# Catalytic Concerted S_N_Ar Reactions of Fluoroarenes by an Organic Superbase

**DOI:** 10.1021/jacs.4c09042

**Published:** 2024-11-08

**Authors:** Masanori Shigeno, Kazutoshi Hayashi, Ozora Sasamoto, Riku Hirasawa, Toshinobu Korenaga, Shintaro Ishida, Kanako Nozawa-Kumada, Yoshinori Kondo

**Affiliations:** † Department of Biophysical Chemistry, Graduate School of Pharmaceutical Science, Tohoku University, Aoba, Sendai 980-8578, Japan; ‡ JST, PRESTO, Kawaguchi, Saitama 332-0012, Japan; § Department of Chemistry and Biological Sciences, Faculty of Science and Engineering, Iwate University, Ueda, Morioka 020-8551, Japan; ∥ Soft-Path Science and Engineering Research Center (SPERC), Iwate University, Ueda, Morioka 020-8551, Japan; ⊥ Department of Chemistry, Graduate School of Science, Tohoku University, Sendai 980-8578, Japan; # Interdisciplinary Research Center for Catalytic Chemistry, National Institute of Advanced Industrial Science and Technology (AIST), Central 5, 1-1-1 Higashi, Tsukuba 305-8565, Ibaraki, Japan

## Abstract

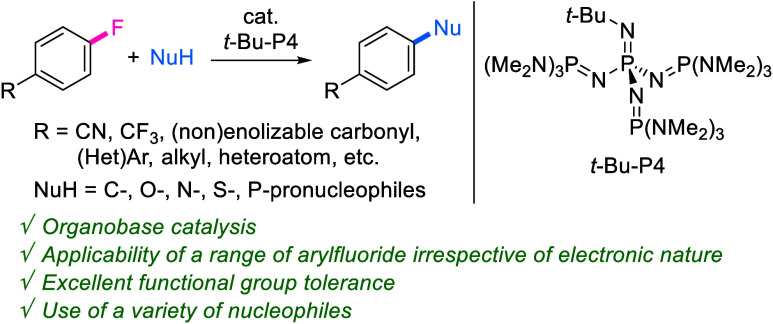

We herein propose that the catalytic concerted S_N_Ar reaction is a powerful method to prepare functionalized aromatic scaffolds. Classic stepwise S_N_Ar reactions involving addition/elimination processes require the use of electron-deficient aromatic halides to stabilize Meisenheimer intermediates, despite their widespread use in medicinal chemistry research. Recent efforts have been made to develop concerted S_N_Ar reactions involving a single transition state, allowing the use of electron-rich substrates based on the use of stoichiometric amounts of strong bases or reactive nucleophiles. This study demonstrates that, without the use of such reagents, the organic superbase *t*-Bu-P4 efficiently catalyzes the concerted S_N_Ar reactions of aryl fluorides regardless of their electronic nature. The key to establishing this system is the dual activation of aryl fluoride and anionic nucleophiles by the *t*-Bu-P4 catalyst. Furthermore, this catalysis allows excellent functional group tolerance, utilization of diverse nucleophiles, and late-stage functionalization of bioactive compound derivatives. These findings make possible diverse applications in chemical synthesis and pharmaceutical development.

## Introduction

**Figure 1:**
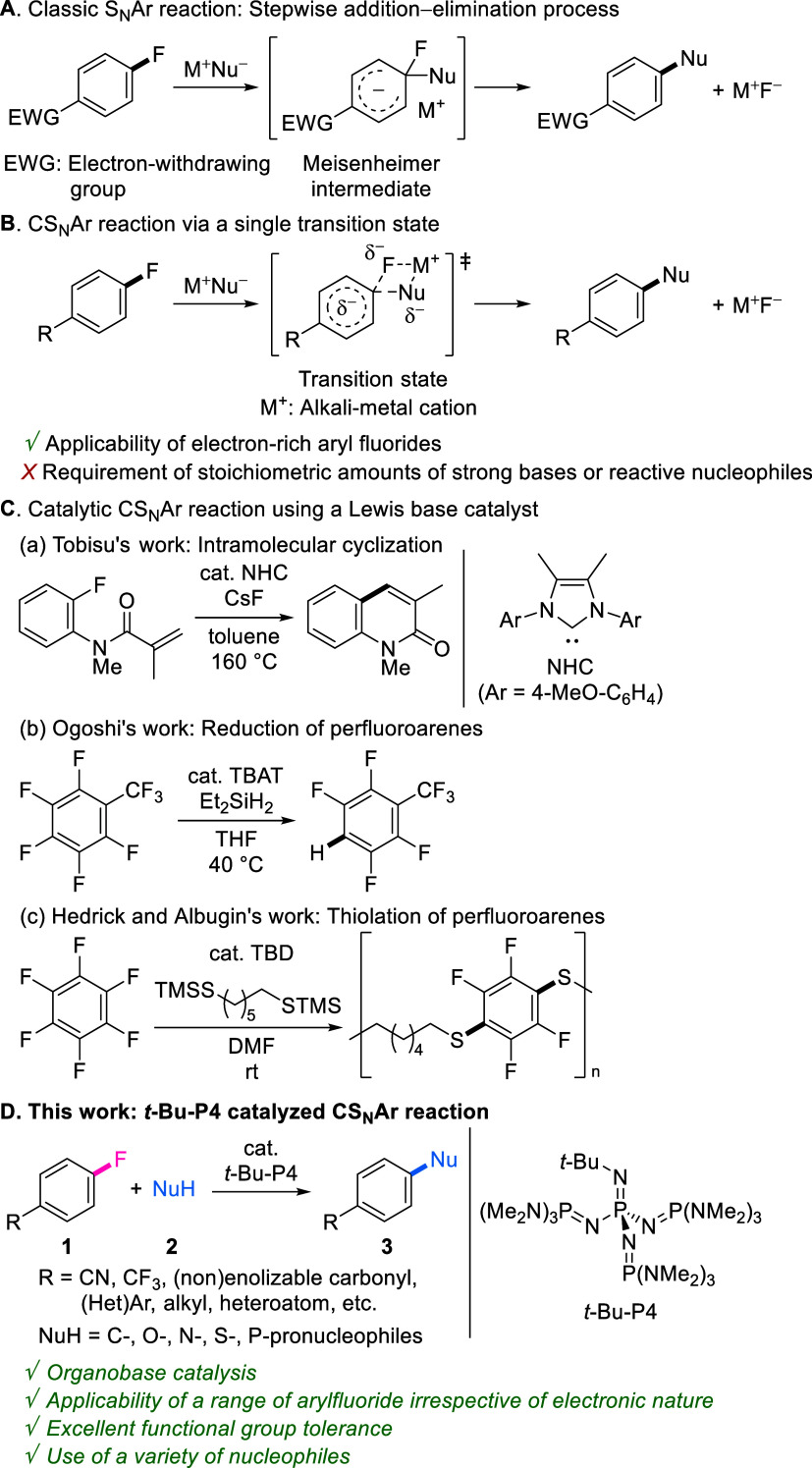
Overview of previous S_N_Ar reactions and this work.

The nucleophilic aromatic substitution (S_N_Ar) reaction of aryl halides involves regioselective *ipso*-substitution and generates functionalized aromatic compounds relevant to pharmaceuticals, agrochemicals, and functional materials.^1^ Early studies on this reaction date back to at least the 1870s.^2^ A 2016 report documented that the S_N_Ar reaction is the second most commonly used reaction in medicinal chemistry research,^3^ appearing at least once in the synthetic route of many best-selling drugs.^4^ Aryl fluorides are suitable substrates for this reaction, in which the fluorine atom of a strong C–F bond with high bonding energy is replaced.^1^ Such C–F bond transformations of aryl fluorides have received considerable attention in organic synthesis due to scientific interest as well as their abundance, ready availability, and structural diversity.^5^ Furthermore, this reaction enables the late-stage functionalization of complex molecules.^6^ The classic S_N_Ar reaction mechanistically involves the addition of a nucleophile to form a Meisenheimer intermediate, followed by the elimination of fluorine along with rearomatization of the aryl ring (Figure 1A). Despite the widespread use of this reaction, an inherent and significant drawback is the need to use electron-deficient substrates possessing an electron-withdrawing group at the *ortho*- or *para*-position to stabilize the Meisenheimer intermediate and compensate for the loss of aromaticity. Although stepwise elimination and addition processes using strong bases via an aryne intermediate have also been reported as alternative pathways, regioselective control of the addition is not facile and affords a mixture of *ipso*- and *ortho*-substituted products.^7^

As a recent prominent advance, the concerted nucleophilic aromatic substitution (CS_N_Ar) reaction through a single transition state has emerged (Figure 1B).^8^ Theoretical and experimental studies by Jacobsen’s group and Murphy and Tuttle’s group highlighted the CS_N_Ar reactions and suggested the potential reaction scope.^9,10^ Here, cation M^+^ (mainly, alkali-metal cations) undergoes a favorable electrostatic interaction with a fluorine atom to stabilize its negative charge in the transition state and assists in fluorine elimination.^10^ Based on these findings, several groups have successfully demonstrated the applicability of nonelectrophilically activated aryl fluorides, even including electron-rich substrates, for these reactions.^11^ This reaction, however, remains sporadically reported and understudied. Moreover, this reaction requires the use of stoichiometric amounts of strong bases {e.g., MHMDS (M = Li, Na, K) and KO-*t*-Bu} or reactive nucleophiles. Thus, the construction of a catalytic CS_N_Ar reaction system without the use of such reagents is attractive and merits investigation. To date, a few nucleophilic Lewis base catalysts have been demonstrated (Figure 1C). For example, Tobisu and co-workers reported that *N*-heterocyclic carbene catalyzes intramolecular cyclization of fluoroarenes bearing an acrylamide moiety to afford quinoline-2-ones (Figure 1C-a).^12^ In addition, catalytic reduction and thiolation reactions of perfluoroarenes were described with silane and silylated thiol nucleophiles, respectively (Figure 1C-b,c).^13,14^ However, these pioneering reactions have a limited scope of aryl fluorides (i.e., intramolecular cyclization substrates and electron-deficient substrates) and nucleophilic components (i.e., special C=C double bonds and organosilanes). Therefore, the development of a powerful and robust catalytic system that is compatible with a range of aryl fluorides, regardless of their electronic nature, and nucleophiles as well as diverse functional groups is highly desirable and is expected to lead to considerable advances in the chemistry of S_N_Ar reactions. In addition, other catalytic S_N_Ar reactions using cationic transition metals (Rh(III) and Ru(II))^15^ or (electro)photocatalysts^16^ proceed through the electrophilic activation of aromatic rings (i.e., formation of a metal *h*^6^-fluoroarene complex or generation of an aryl radical cation intermediate by single-electron arene oxidation, respectively), which mainly focuses on the use of electron-rich aryl fluorides. Thus, based on the different mechanisms, the catalytic CS_N_Ar system proposed herein is complementary to those catalysis.

Our group has developed molecular transformations using the organic superbase *t*-Bu-P4, which was originally prepared by Schwesinger et al.^17,18^ Deprotonation of a pronucleophile by *t*-Bu-P4 forms a reactive anion species because the electrostatic interaction of the anion with the large soft cation [*t*-Bu-P4]^+^H (∼500 Å^3^) is rather weak.^19^ Based on this, we previously showed that *t*-Bu-P4 catalyzes the S_N_Ar reactions of electron-deficient methoxyarenes with alkyl cyanides, alcohols, and amines.^20^ It was also reported that the reactions of electron-deficient aryl fluorides proceed with organosilicon.^21^ In previous studies, we found that when 3-fluoro-4-methoxybenzonitrile was treated with 2-phenylpropionitrile (**2a**), not only methoxy substitution but also fluorine substitution occurred (Scheme S1).^20c^ This result was notable because the electronically insufficiently activated aryl fluoride moiety reacted under the applied conditions, suggesting that *t*-Bu-P4 catalysis could be a potent catalytic S_N_Ar system for aryl fluorides. Here, the developed system based on this finding is reported (Figure 1D).

## Results and Discussion

### Reaction Development

Initially, the reaction conditions were investigated using electron-neutral 4-fluorobiphenyl (**1a**) and 2-phenylpropionitrile (**2a**) as model substrates (Table 1). When the reaction was conducted with *t*-Bu-P4 (20 mol %) at 80 °C, target product **3aa** was obtained in 39% yield (entry 1). Then, molecular sieves were added as a heterogeneous solid base to trap the generated HF, and the 4 Å MS greatly increased the product yield to 91% (entries 2–4).^22^ The use of inorganic bases, such as Na_2_CO_3_, K_2_CO_3_, Cs_2_CO_3_, K_3_PO_4_, and KOH, resulted in modest to good yields (45–88%), while organic bases, such as NEt_3_ and pyridine, were less effective (Table S1). Among the evaluated solvents, 1,4-dioxane and cyclohexane resulted in good yields (81 and 87%, respectively), but these values were inferior to that obtained in toluene (entries 5–7). A reduced amount of *t*-Bu-P4 (10 mol %) still afforded a good yield of 83% (entry 8). Decreasing the reaction temperature decreased the product yield (entry 9). *t*-Bu-P4 was found to be an exceptionally effective base catalyst compared with other catalysts (Table S2). This catalyst was amenable to a scaled-up reaction on the 1.0 mmol scale, furnishing the product in 91% yield (entry 10).

**Table 1:**
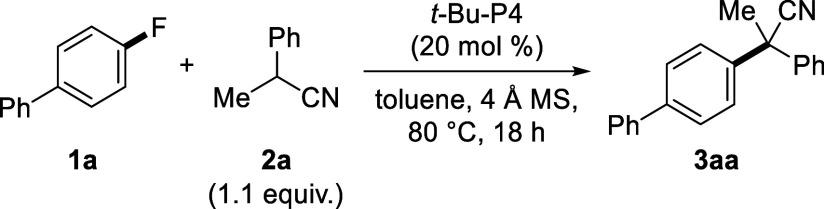
Optimization of the Reaction Conditions of **1a** and **2a**^a^

entry	deviation from standard conditions	**3aa** (%)^b^
1	without 4 Å MS	39
2	3 Å MS instead of 4 Å MS	79
3	none	91
4	5 Å MS instead of 4 Å MS	71
5	1,4-dioxane as the solvent	81
6	cyclohexane as the solvent	87
7	DMF as the solvent	56
8	10 mol % *t*-Bu-P4	83
9	60 °C	55
10	scaled-up reaction^c^	91

a Standard conditions: **1a** (0.20 mmol), **2a** (0.22 mmol), *t*-Bu-P4 (0.04 mmol), 4 Å MS (100 mg), toluene (0.3 mL), 80 °C, and 18 h.

b Isolated yields.

c **1a** (1.0 mmol), **2a** (1.1 mmol), *t*-Bu-P4 (0.20 mmol), 4 Å MS (500 mg), toluene (1.5 mL), 80 °C, and 18 h.

### Substrate Scope with Respect to Fluoroarenes

**Figure 2:**
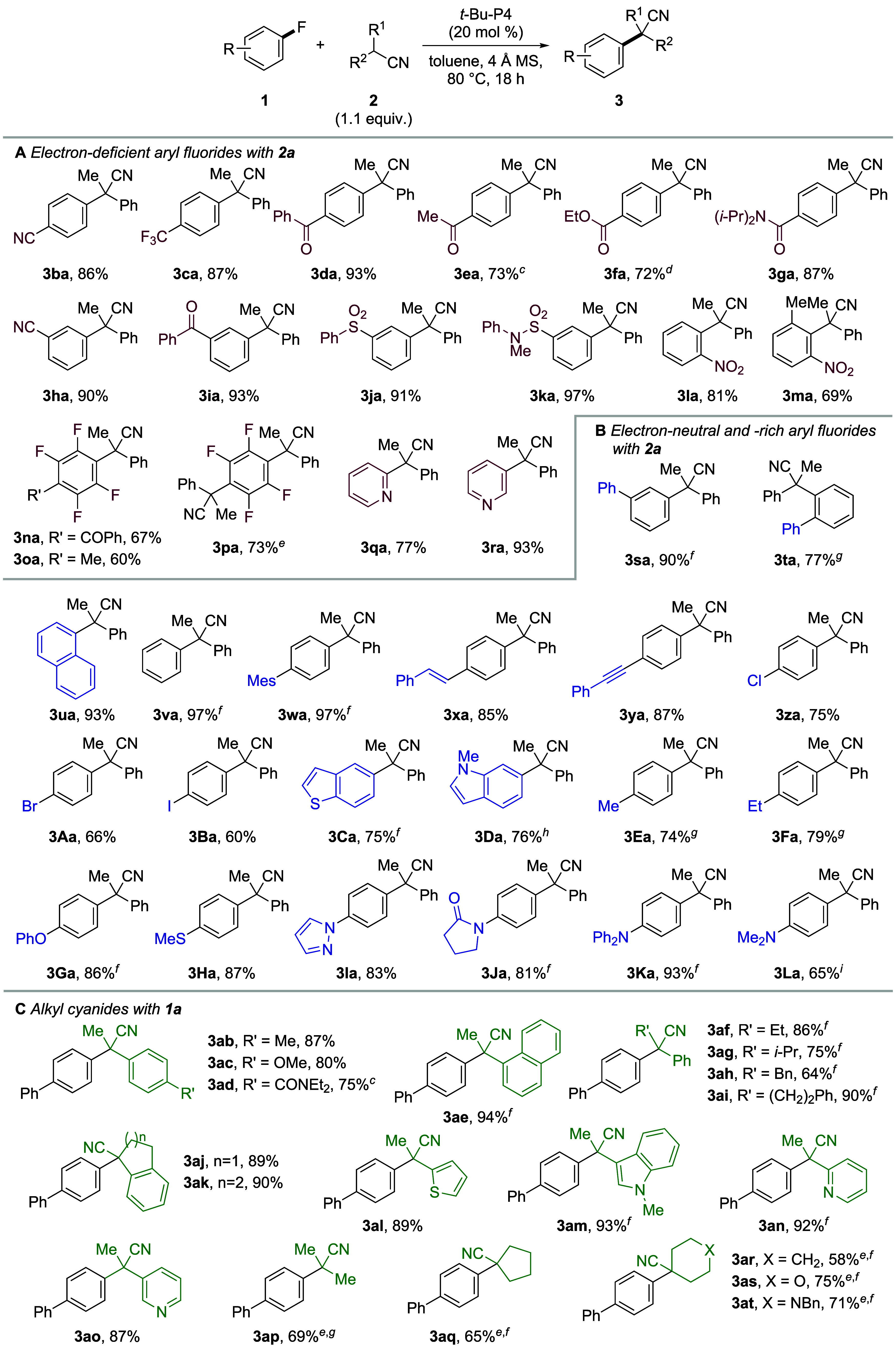
Scope of aryl fluorides and alkyl cyanides.^*a*,*b a*^Reactions were conducted on a 0.2 mmol scale. ^*b*^Isolated yields. ^*c*^Reaction was conducted at 40 °C. ^*d*^Reaction was conducted at 60 °C. ^*e*^**2** (2 equiv) was used. ^*f*^Reaction was conducted at 120 °C. ^*g*^Reaction was conducted at 140 °C. ^*h*^Reaction was conducted in mesitylene at 160 °C. ^*i*^Reaction was conducted in mesitylene at 180 °C.

With the optimized conditions in hand, we examined the substrate scope of fluoroarenes with **2a** (Figure 2). First, electron-deficient substrates were tested (Figure 2A). Electrophilic functional groups (i.e., cyano, trifluoromethyl, (non)enolizable ketone, ester, and amide), which may be susceptible to nucleophilic addition or substitution, at the *para* position of the fluorine atom were well tolerated, and target products **3ba**–**3ga** were furnished in high yields (72–93%). In addition, less electron-deficient *meta*-substituted substrates **1h**–**1k** possessing cyano, benzoyl, sulfonyl, and sulfonamide moieties successfully afforded products **3ha**–**3ka**, despite the potential side reaction, e.g., deprotonation of the relatively acidic C2-proton and subsequent aryne formation. Reactions of nitro-substituted substrates also proceeded smoothly. Perfluoroarenes bearing benzoyl and methyl groups, **1n** and **1o**, provided the monosubstitution products, while hexafluorobenzene (**1p**) gave the disubstitution product. Additionally, not only 2-fluoropyridine (**1q**) but also less electrophilic 3-fluoropyridine (**1r**) underwent reaction to generate products **3qa** and **3ra**, respectively, in high yields.

Next, the scope of electron-neutral and electron-rich substrates was examined (Figure 2B). Regardless of the position of the phenyl group, the reactions proceeded smoothly to form target products **3sa** and **3ta** in high yields of 90 and 77%, respectively. 1-Fluoronaphthalene (**1u**) and fluorobenzene (**1v**) were also suitable substrates, producing the target products in excellent yields. Use of substrate **1w**, bearing a mesityl group at the *para*-position, provided product **3wa** in 97% yield. This is in contrast to previous reports of CS_N_Ar silylation and phosphinylation, which indicated that substrate **1w** and related 4′-fluoro-2,4,6-triisopropyl-1,1′-biphenyl with large torsion angles are unfavorable substrates since their fluorine-*ipso*-carbons are shielded by the distal methyl and isopropyl substituents, respectively.^11f,11h^ Substrates **1x** and **1y** demonstrated the compatibility of alkene and alkyne moieties, respectively. Moreover, chemoselective reactions proceeded successfully in the presence of halogen atoms (Cl, Br, and I), affording products **3za**–**3Ba**. Then, the electron-rich heteroaromatic fluorides 5-fluorobenzothiophene (**1C**) and 6-fluoro-1-methyl-1*H*-indole (**1D**) were tested, resulting in the formation of products **3Ca** and **3 Da**, respectively, in good yields. Substrates with alkyl groups (i.e., Me and Et) or heteroatom substituents (i.e., OPh, SMe, pyrazolyl, and 2-pyrrolidone groups) also generated target products **3Ea**–**3Ja** in high yields (74–87%). Furthermore, substrates with strong electron-donating amino groups (i.e., NPh_2_ and NMe_2_) were suitable for this reaction, affording products **3Ka** and **3La**, respectively. In comparison to fluoroarenes, other (pseudo)halobenzenes such as chlorobenzene, bromobenzene, and nitrobenzene were unsuitable substrates (Table S3).

### Substrate Scope with Respect to Alkyl Cyanides

The scope of alkyl cyanides was investigated with fluoroarene **1a** (Figure 2C). 2-Arylpropionitriles **2b**–**2d** with methyl, methoxy, and amide moieties on the phenyl ring and **2e** with a 1-naphthyl group produced target products **3ab**–**3ae** in high yields (75–94%). In addition, good to high yields were observed for substrates **2f**–**2i**, containing ethyl, isopropyl, benzyl, and phenethyl groups, respectively, at the α-position of the cyano group and **2j** and **2k**, bearing Indane and tetrahydronaphthalene scaffolds, respectively. Heteroaromatic ring-containing substrates **2l**–**2o** afforded products **3al**–**3ao** in excellent yields (87–93%). Furthermore, alkyl cyanides **2p**–**2t** without an aryl group, which have a higher p*K*_a_ value (e.g., propionitrile, 32.5, DMSO)^23a^ than **2a** (23.0, DMSO)^23b^ and are less prone to deprotonation, furnished the corresponding products in good yields (58–75%). The use of carbon nucleophiles bearing ketone, ester, nitro, and trifluoromethyl moieties instead of a cyano group resulted in a low yield or no product formation (Table S4). The high reactivity of alkyl cyanides is considered to be due to the small size of the cyano group^24^ and the formation of a less-stabilized carbanion intermediate.^25,26^

### Extension to Transformations with Heteroatom Nucleophiles

**Figure 3:**
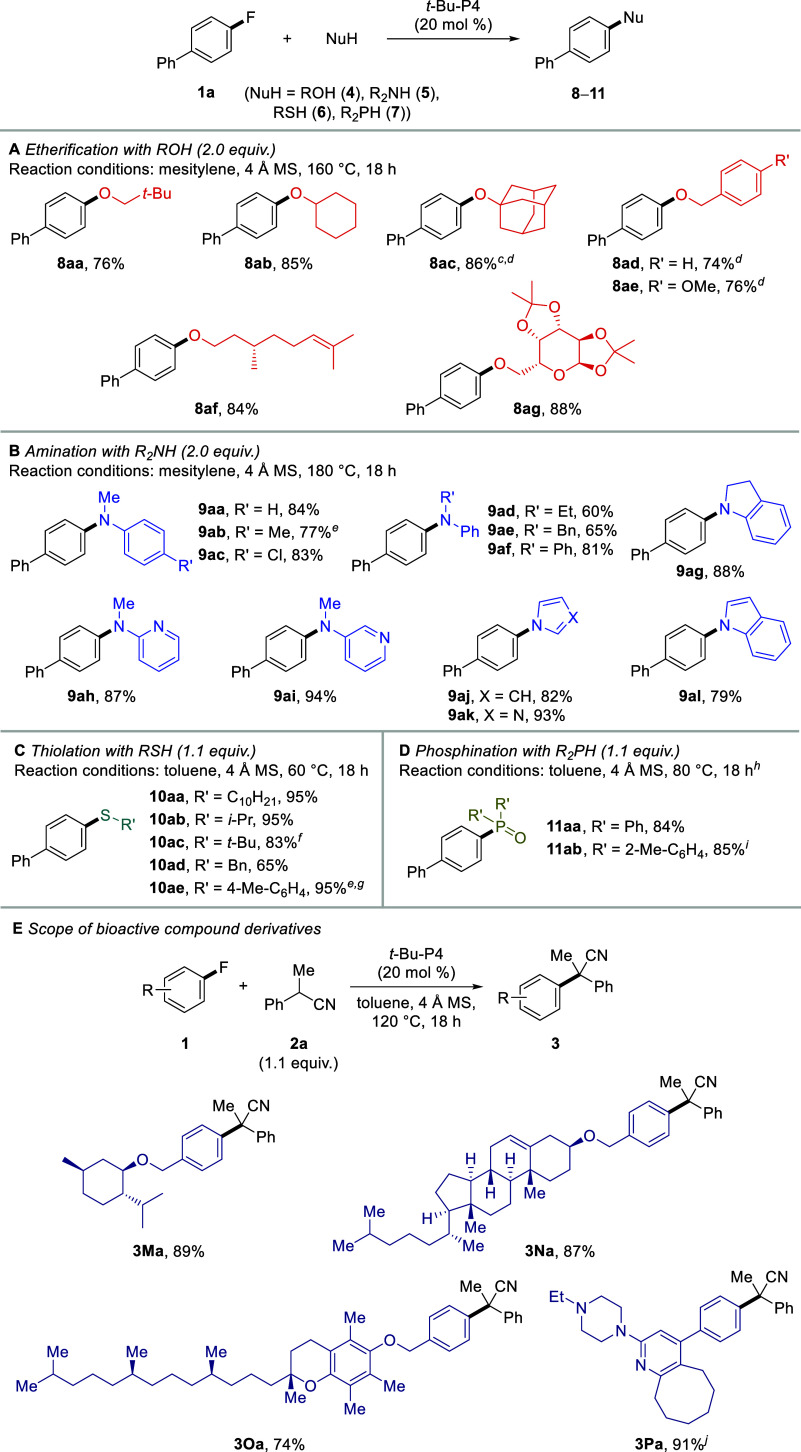
Scope of heteroatom nucleophiles in the reaction of **1a** (A–D) and functionalization of bioactive compound derivatives (E).^*a*,*b a*^Reactions were conducted on a 0.2 mmol scale. ^*b*^Isolated yields. ^*c*^Reaction was conducted at 200 °C. ^*d*^DMI was used as a solvent. ^*e*^Reaction was conducted at 160 °C. ^*f*^THF was used as a solvent. ^*g*^Mesitylene was used as a solvent. ^*h*^Products were isolated after oxidation with H_2_O_2_. ^*i*^Reaction was conducted at 140 °C. ^*j*^Reaction was conducted at 80 °C.

The current system was extended to transformations with heteroatom nucleophiles (Figure 3; for optimization of the reaction conditions, see the Supporting Information). Specifically, etherification reactions of primary, secondary, and tertiary alcohols **4a**–**4c** with fluoroarene **1a** proceeded to provide products **8aa**–**8ac** in high yields (76–86%) (Figure 3A). Benzyl alcohols **4d** and **4e** were also employed to form the target products. Moreover, β-citronellol and diacetone-d-galactose with intricate structures successfully afforded products **8af** and **8ag**, respectively, in excellent yields.

Next, amination reactions using nitrogen nucleophiles were investigated (Figure 3B). The reactions proceeded with various *N*-alkyl aniline derivatives {i.e., *N*-methylaniline (**5a**), its methyl- and chlorine-substituted derivatives **5b** and **5c**, *N*-ethylaniline (**5d**), *N*-benzylaniline (**5e**), diphenylamine (**5f**), and indoline (**5g**)}, producing target products **9aa**–**9ag** in good to high yields (60–88%). 2-(Methylamino)- and 3-(methylamino)pyridines, **5h** and **5i**, were also suitable substrates. Additionally, high yields were obtained with nitrogen-containing heteroarenes, such as pyrrole (**5j**), imidazole (**5k**), and indole (**5l**). Pyrrolidine, which has a large p*K*_a_ value (p*K*_a_ = 44, DMSO;^23c^ cf. **5a**, p*K*_a_ = 29.5, DMSO),^23d^ was also tested in the reaction but did not form the product (data not shown).

Furthermore, thiolation and phosphination reactions were surveyed (Figure 3C,D).^27^ The use of primary, secondary, and tertiary thiols **6a**–**6c**, benzyl mercaptan (**6d**), and *p*-toluenethiol (**6e**) resulted in the formation of the target products in good to excellent yields (65–95%). Phosphination using diphenylphosphine (**7a**) and di-*o*-tolylphosphine (**7b**) was also successful, and the products were isolated as phosphine oxide after oxidation. Thus, versatile nucleophiles were applicable, demonstrating the universality of this catalysis to access biologically important classes of functionalized aromatic compounds.

### Synthetic Applications

We next examined the potential for the late-stage functionalization of bioactive compound derivatives with diverse structures (Figure 3E). Menthol, cholesterol, and α-tocopherol derivatives as well as blonanserin were employed in the reaction with **2a**, successfully providing target products **3Ma**–**3Pa** in excellent yields (74–91%). The results further illustrate the robustness of this catalytic protocol.

### Proposed Mechanism

**Figure 4:**
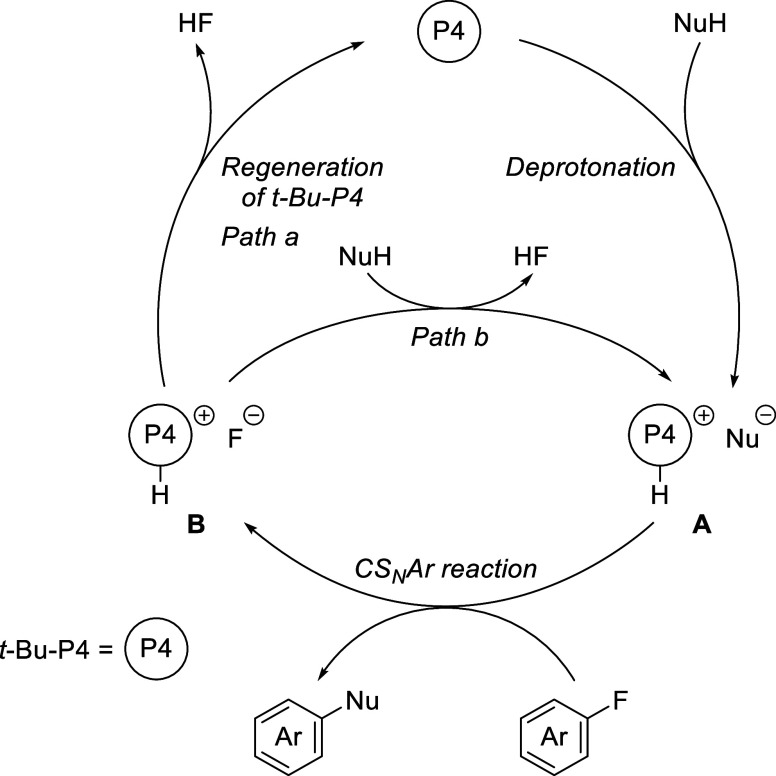
Proposed mechanism.

The reaction mechanism is shown in Figure 4. The *t*-Bu-P4 base initially deprotonates the nucleophile to form ionic intermediate **A**. Subsequently, **A** undergoes aryl fluoride substitution, generating the arylated product and fluoride salt **B**. Then, *t*-BuP4 is regenerated with the release of an HF molecule, which is trapped by the molecular sieves, thus completing the catalytic cycle (path a). Alternatively, it is also plausible that **A** is regenerated through deprotonation of the nucleophile by the fluoride anion in **B** (path b).

### Hammett Analysis

A Hammett plot was constructed from the correlation of the kinetic data for the reactions of substrates **1a**, **1z**, **1B**, and **1K** with σ_p_ parameters (Figures S1 and S2 and Table S12). A positive reaction constant (ρ = 2.9) was obtained, indicating that a partial negative charge is built up on the aromatic ring in the transition state. This constant is within the range of those observed in previously reported CS_N_Ar reactions.^8a^

### Density Functional Theory Calculations

**Figure 5:**
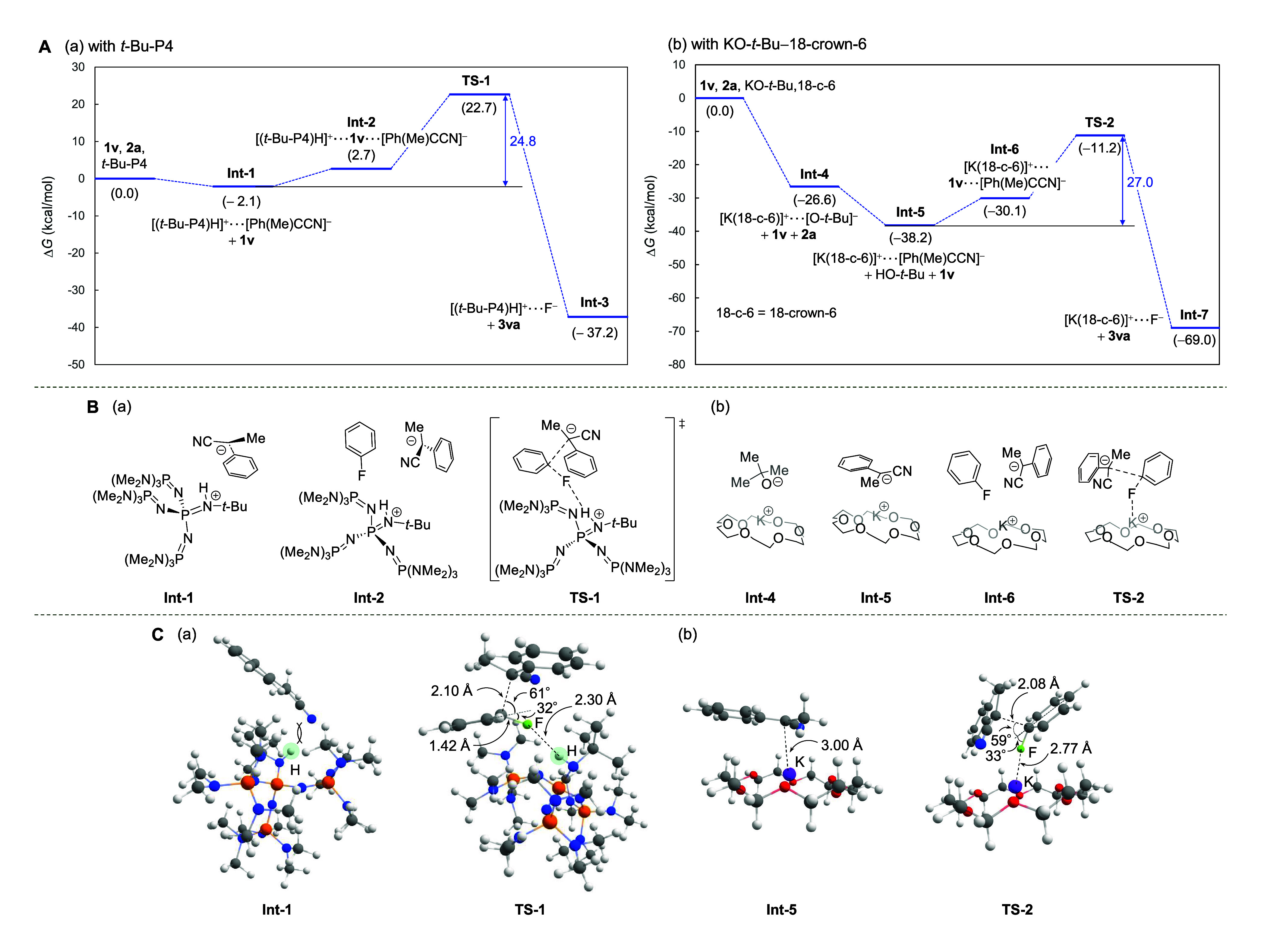
Theoretical mechanistic studies. (A) Energy diagram using Gibbs free energies calculated at the CPCM(toluene)/oniom(ωB97X-D/6-311+G(2d,p):B97-D/6-311G(d,p)) level of theory. (B) Chemical structures of intermediates and transition state. (C) 3D structures of intermediates and transition state.

Next, we conducted density functional theory (DFT) calculations to gain insight into the reaction mechanism (Figure 5; CPCM(toluene)/oniom(ωB97X-D/6-311+G(2d,p):B97-D/6-311G(d,p)) level of theory, and the division of layers is shown in the Supporting Information). The reaction coordinate diagram of PhF (**1v**) and PhCHMeCN (**2a**) with *t*-Bu-P4 was evaluated (Figure 5A-a). The pathway consists of the formation of a deprotonated intermediate of **2a** (**Int-1**), a pretransition state (**Int-2**), and a transition state (**TS-1**) to afford a fluorine-substituted product (**Int-3**). Notably, the intrinsic reaction coordinate (IRC) calculation showed a single barrier of **TS-1** between **Int-2** and **Int-3** and no formation of a Meisenheimer intermediate. In addition, attempts to locate the Meisenheimer intermediate did not afford a stable intermediate.^28^ Thus, nucleophile attack and fluorine elimination occur in a synchronous manner in **TS-1**, supporting the involvement of the CS_N_Ar reaction mechanism.

**Figure 6:**
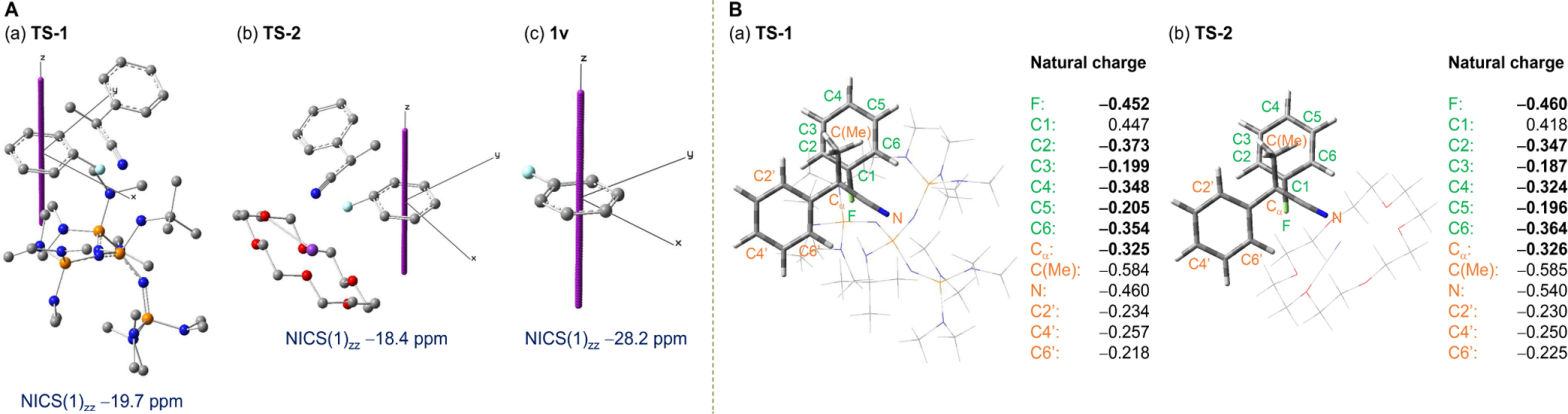
(A) NICS_*zz*_ scan of (a) **TS-1**, (b) **TS-2**, and (c) **1v** calculated at the GIAO/B3LYP/6-311+G(d) level of theory. The *z* axis for the NICS_*zz*_-scan and z values are shown as purple dots. (B) Natural population analysis (NPA) of (a) **TS-1** and (b) **TS-2** calculated at the M06-2*X*/6-311+G(2d,p) level of theory using the optimized structures obtained at the CPCM(toluene)/oniom(ωB97X-D/6-311+G(2d,p):B97-D/6-311G(d,p)) level of theory.

We subsequently focused on the molecular state of **TS-1**. In **TS-1**, the C_aryl_–F bond is bent from the phenyl plane by 32° with hydrogen bond formed between the fluorine of PhF and the proton of [(*t*-Bu-P4)H]^+^, and the carbanion nucleophile approaches the fluorine-*ipso* carbon from the opposite side of the phenyl ring (Figure 5C-a). Nucleus-independent chemical shift (NICS) calculations of **TS-1** yielded NICS(1)_*zz*_ values of −19.7 ppm for the phenyl ring, indicating that the aromaticity is retained, although it is slightly weakened compared to that of **1v** (−28.2 ppm) (Figure 6A). Natural population analysis (NPA) of **TS-1** showed that the negative charges are located not only on the aromatic ring (C2: −0.373; C3: −0.199; C4: −0.348; C5: −0.205; C6: −0.354) but also on the eliminating fluorine atom (−0.452) and the nucleophilic carbon (−0.325) (Figure 6B). These are characteristic features of the transition state of CS_N_Ar reactions, unlike the case of the Meisenheimer structure that loses aromaticity and localizes the negative charge on the aromatic ring, contributing to the applicability of poorly reactive electron-rich aryl fluorides.^8b,29^ Furthermore, natural bond orbital (NBO) analysis showed orbital interactions of the σ(C_ipso_–C_α_) bond orbital with aromatic π* orbitals as well as hydrogen-bond formation between the proton of [(*t*-Bu-P4)H]^+^ and the fluorine of PhF, which aid in stabilizing **TS-1** (Figure S4).

Furthermore, for comparison, calculations were conducted for KO-*t*-Bu-18-crown-6, which is generally considered to generate a reactive anion species by the coordination of 18-crown-6 to potassium cation (Figure 5A-b). A reaction coordinate diagram similar to that for *t*-Bu-P4 was obtained; however, the activation energy of **TS-2** was greater (27.0 kcal/mol) than that of **TS-1** (24.8 kcal/mol). This result is consistent with the high reactivity of *t*-Bu-P4.

**Figure 7:**
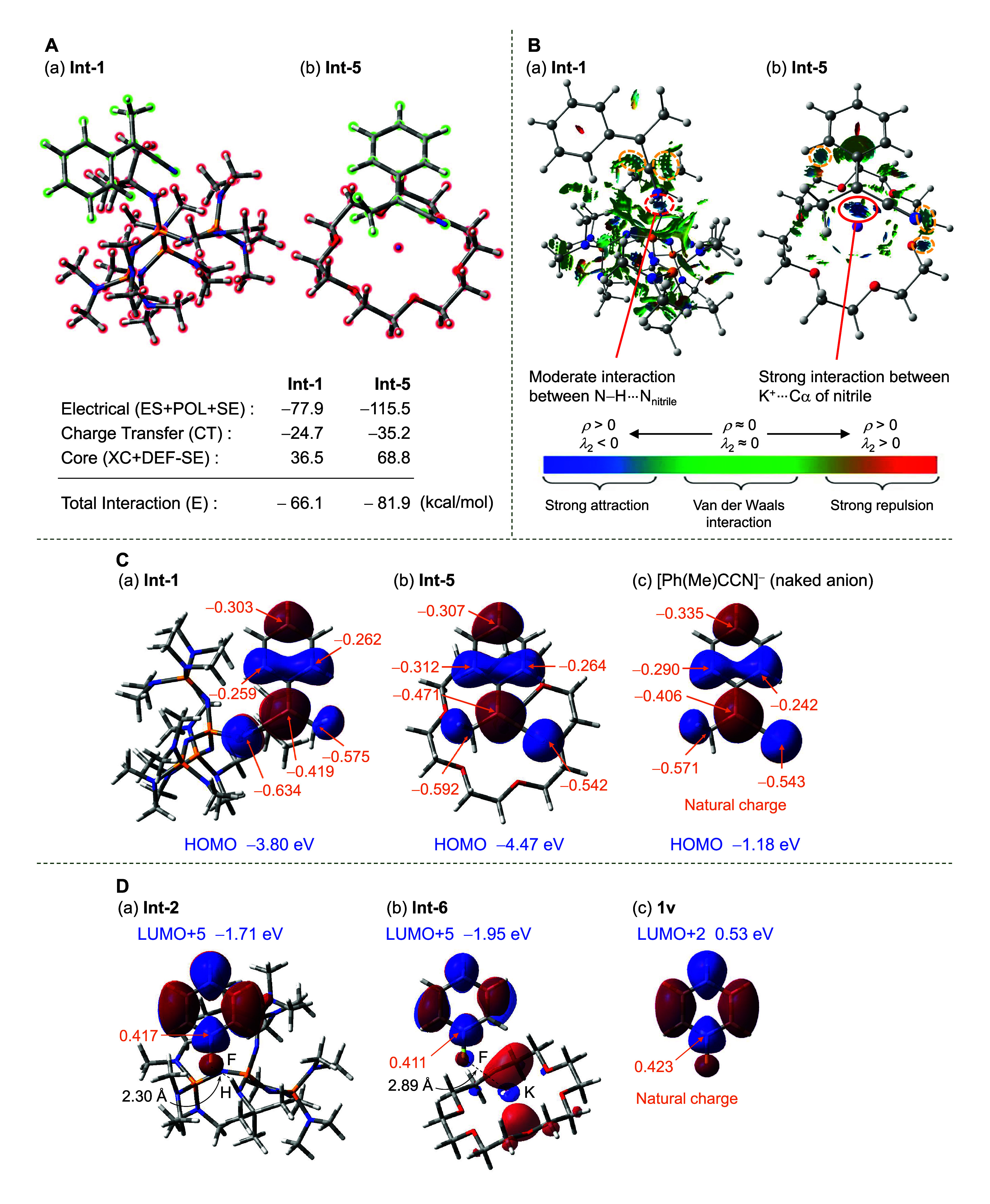
(A) Natural energy decomposition analysis (NEDA) for (a) **Int-1** ([(*t*-Bu-P4)H]^+^···[Ph(Me)CCN]^−^) and (b) **Int-5** ([K(18-crown-6)]^+^···[Ph(Me)CCN]^−^) (kcal/mol). In figures (A–D), all single point calculations were performed at the M06-2*X*/6-311+G(2d,p) level of theory using the optimized structures obtained at the CPCM(toluene)/oniom(ωB97X-D/6-311+G(2d,p):B97-D/6-311G(d,p)) level of theory. (B) Noncovalent interaction analyses of (a) **Int-1** and (b) **Int-5**. The red surface indicates strong repulsive interactions, while the green and blue surfaces show weak and strong attractive interactions, respectively. (C) Molecular states of (a) **Int-1**, (b) **Int-5**, and (c) [Ph(Me)CCN]^−^ (naked anion). The HOMOs of the anionic parts and their energies are shown. The natural charges of the anionic parts are also noted. (D) Molecular states of fluorobenzene moieties in (a) **Int-2** and (b) **Int-6** and the molecular state of (c) **1v**. In molecular states **Int-2** and **Int-6**, the anionic part was omitted for simplicity. The π* orbitals of the aromatic rings (**Int-2**: LUMO+5; **Int-6**: LUMO + 5; **1v**: LUMO + 2) and their energies are shown. The natural charges of the *ipso*-carbons attached to a fluorine atom are also noted.

To understand the high reactivity achieved by the *t*-Bu-P4 base over KO-*t*-Bu-18-crown-6, we conducted the following studies. The nucleophilicity of the deprotonated intermediates (**Int-1** and **Int-5**) was investigated. Specifically, natural energy decomposition analysis (NEDA) was conducted to evaluate the interaction energies between the cationic part ([(*t*-Bu-P4)H]^+^ and [K(18-crown-6)]^+^) and the anionic part in **Int-1** and **Int-5** by calculating the electrical interaction, charge transfer, and core repulsion (Figure 7A). A smaller interaction energy (−66.1 kcal/mol) and thus less stabilization were observed in the case of [(*t*-Bu-P4)H]^+^ than in the case of [K(18-crown-6)]^+^ (−81.9 kcal/mol). A weaker interaction was also observed for [(*t*-Bu-P4)H]^+^ by noncovalent interaction (NCI) analysis because the proton on [(*t*-Bu-P4)H]^+^ is embedded in the pocket of the *t*-Bu-P4 catalyst, surrounded by bulky *t*-Bu and NMe_2_ groups, and thus is insufficient to stabilize the carbanion species, which is in contrast to the case of [K(18-crown-6)]^+^, which accepts anionic coordination (Figure 7B). Furthermore, the HOMO energy level of the anionic species of **2a** is higher for [(*t*-Bu-P4)H]^+^ (−3.80 eV) than for [K(18-crown-6)]^+^ (−4.47 eV) (Figure 7C). Thus, *t*-Bu-P4 significantly enhances the nucleophilicity of the anionic moiety. Then, we also investigated the effect of the catalyst on the ArF reactivity in pretransition states **Int-2** and **Int-6** (Figure 7D). The proton on [(*t*-Bu-P4)H]^+^ forms a hydrogen bond with the fluorine atom in **Int-2** and the LUMO + 5 of the PhF moiety, corresponding to the orbital that is subject to nucleophilic attack, possessing a lower energy level of −1.71 eV than the LUMO + 2 of PhF without a catalyst (0.53 eV), thus demonstrating the activation of PhF by [(*t*-Bu-P4)H]^+^. Nevertheless, this finding cannot sufficiently explain the high reactivity of *t*-Bu-P4 because the LUMO + 5 was lowered by [K(18-crown-6)]^+^ (−1.95 eV). Thus, *t*-Bu-P4 allows the dual activation of anionic nucleophile and ArF components, the former of which is more crucial for its high reactivity.

## Conclusions

In summary, our study revealed a highly efficient method for catalytic CS_N_Ar reactions of aryl fluorides by *t*-Bu-P4. This catalytic reaction exhibited excellent functional group tolerance and allowed the use of diverse nucleophiles, thus making it a versatile tool for synthetic chemists. In addition, this system enabled the late-stage functionalization of bioactive compound derivatives. These findings demonstrate the promise of this approach in the development of new pharmaceuticals and the synthesis of complex molecules, offering exciting opportunities for future research and application.

## Supporting Information Available

Reaction of 3-fluoro-4-methoxybenzonitrile (**12**) and **2a** (Scheme S1); effects of base additives in the reaction conditions of **1a** and **2a** (Table S1); effects of base catalysts in the reaction conditions of **1a** and **2a** (Table S2); reactions of (pseudo)halobenzenes (**16**) and **2a** (Table S3); reactions of **1a** and carbon nucleophiles other than alkyl cyanide (Table S4); optimization of the reaction conditions of **1a** and heteroatom nucleophiles (Tables S5–S11); Hammet analysis (Figures S1 and S2 and Table S12); details for NICS_*zz*_-scan of **1v**, **Int-2**, **TS-1**, **Int-6**, and **TS-2** (Figure S3); natural bond orbital (NBO) analysis of **TS-1** (Figure S4); noncovalent interaction analyses of **Int-1** and **Int-5** (Figure S5); experimental procedures and spectra data for obtained products, and ^1^H, ^13^C, ^19^F, and ^31^P NMR spectra (PDF)
