# Supplementary material for: Catalytic Concerted SNAr Reactions of Fluoroarenes by an Organic Superbase
Source: J Am Chem Soc. 2024 Nov 8;146(47):32452–62. doi: 10.1021/jacs.4c09042 (PMC11613311; doi:10.1021/jacs.4c09042)

Catalytic Concerted S_NAr Reactions of Fluoroarenes by an Organic Superbase

Masanori Shigeno,*[1,2] Kazutoshi Hayashi,[1] Ozora Sasamoto,[1] Riku Hirasawa,[1] Toshinobu Korenaga,*[3,4]
Shintaro Ishida,[5] Kanako Nozawa-Kumada,[1,6] and Yoshinori Kondo[1]

[1]Department of Biophysical Chemistry, Graduate School of Pharmaceutical Science,
Tohoku University, Aoba, Sendai, 980-8578, Japan
[2]JST, PRESTO, Kawaguchi, Saitama 332-0012, Japan
[3]Department of Chemistry and Biological Sciences, Faculty of Science and Engineering,
Iwate University, Ueda, Morioka, 020-8551, Japan
[4]Soft-Path Science and Engineering Research Center (SPERC),
Iwate University, Ueda, Morioka, 020-8551, Japan
[5]Department of Chemistry, Graduate School of Science,
Tohoku University, Sendai 980-8578, Japan
[6]Interdisciplinary Research Center for Catalytic Chemistry, National Institute of Advanced Industrial Science and Technology (AIST), Central 5, 1-1-1 Higashi, Tsukuba 305-8565, Ibaraki, Japan

*E-mail: masanori.shigeno.e5@tohoku.ac.jp, korenaga@iwate-u.ac.jp, Fax: (+81) 22-795-6804.

Supporting Information

Table of Contents

General methods....S2
Reaction of 3-fluoro-4-methoxybenzonitrile (**12**) and **2a** (Scheme S1)....S2
Effects of base additives in the reaction conditions of **1a** and **2a** (Table S1)....S3
Effects of base catalysts in the reaction conditions of **1a** and **2a** (Table S2)....S4
Reactions of (pseudo)halobenzenes (**16**) and **2a** (Table S3)....S5
Reactions of **1a** and carbon nucleophiles other than alkyl cyanide (Table S4)....S5
Optimization of the reaction conditions of **1a** and heteroatom nucleophiles (Tables S5-S11)....S6-S12
Hammet analysis (Figures S1 and S2 and Table S12)....S13-S15
Details for $NICS_{zz}$-scan of **1v**, **Int-2**, **TS-1**, **Int-6**, and **TS-2** (Figure S3)....S16
Natural bond orbital (NBO) analysis of **TS-1** (Figure S4)....S17
Noncovalent interaction analyses of **Int-1** and **Int-5** (Figure S5)....S18
Experimental procedures and spectra data for obtained products....S19-S56
Computational section....S57-S72
References....S73-S75
^{1}H, ^{13}C, ^{19}F, and ^{31}P NMR spectra....S76-S173

General methods. All reactions were carried out under N_2 or Ar atmosphere. Flash column chromatography was performed with Kanto silica gel 60 N (spherical, neutral, 40–50 μm). Preparative thin-layer chromatography was performed with silica gel (Wakogel® B-5F). Melting points (Mp) were determined with a Yazawa micro melting point apparatus without correction. Infrared (IR) data were recorded on a SHIMADZU SensIR ATR (Attenuated Total Reflectance) FT-IR or JASCO FT/IR-400 spectrophotometer, and absorbance frequencies are reported in reciprocal centimeters (cm^{-1}). NMR data were recorded on a JEOL AL400 spectrometer (395.75 MHz for ^{1}H, 99.50 MHz for ^{13}C), a Varian Mercury (399.17 MHz for ^{1}H, 100.38 MHz for ^{13}C), or a JEOL ECA600 spectrometer (597.17 MHz for ^{1}H, 150.91 MHz for ^{13}C, 561.81 MHz for ^{19}F, 242.95 MHz for ^{31}P). Chemical shifts are expressed in δ (parts per million, ppm) values, and coupling constants are expressed in herts (Hz). ^{1}H NMR spectra were referenced to tetramethylsilane (0 ppm) as an internal standard or to a solvent signal ($CDCl_3$: 7.26 ppm, DMSO-d_6: 2.49 ppm). ^{13}C NMR spectra were referenced to a solvent signal ($CDCl_3$: 77.0 ppm, DMSO-d_6: 39.5 ppm, acetone-d_6: 30.3 ppm, acetonitrile-d_3: 1.32 ppm, C_6D_6: 128.06 ppm). ^{19}F NMR spectra were referenced to 4-fluorotoluene ($\delta = -118.0$ ppm) as an internal standard. ^{31}P NMR spectra were referenced to H_3PO_4 (0 ppm) as an external standard. Low and high resolution mass spectra (LRMS and HRMS) were obtained from Mass Spectrometry Resource, Graduate School of Pharmaceutical Sciences, Tohoku University, on a JEOL JMS-DX 303 and JMS-700/JMS-T 100 GC spectrometer, respectively.

Scheme S1. Reaction of 3-fluoro-4-methoxybenzonitrile (**12**) and **2a**[a,b]

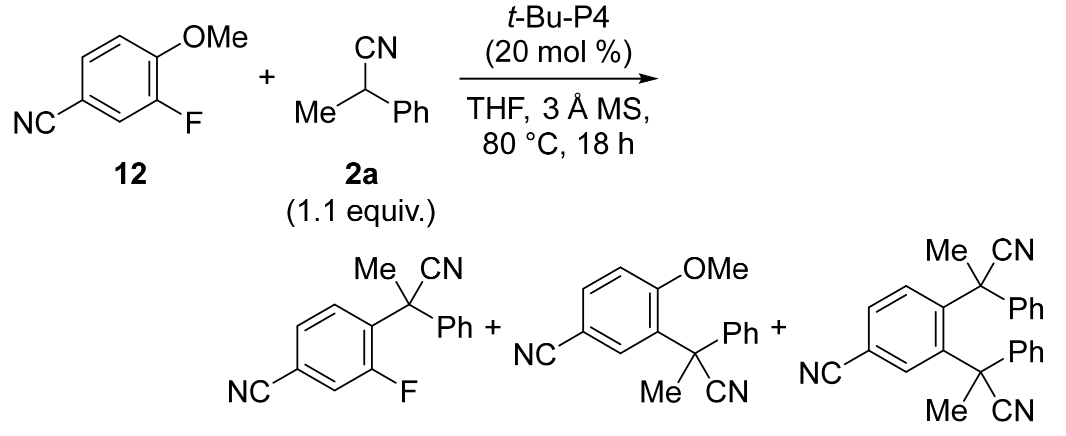

[a]Reaction was conducted on a 0.2 mmol scale. [b]The yields were determined by ^{1}H-NMR using 1,1,2-trichloroethane as an internal standard.

Table S1. Effects of base additives in the reaction conditions of **1a** and **2a**[a]

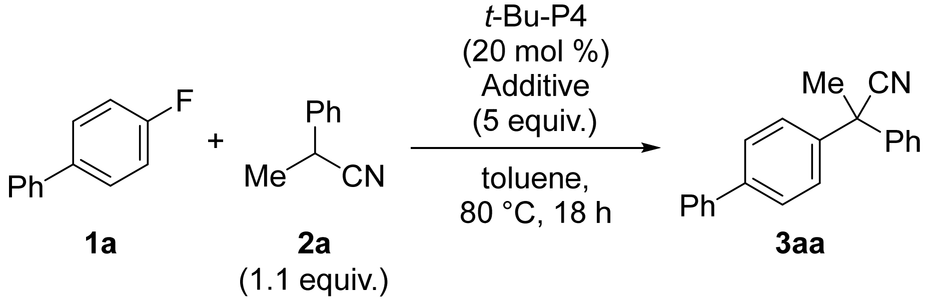

Entry	Additive	**3aa** (%)[b]
1	Na_2CO_3	45
2	K_2CO_3	46
3	Cs_2CO_3	88
4	K_3PO_4	80
5	KOH	63
6	NEt_3	36
7	Pyridine	35

[a]Standard conditions: **1a** (0.20 mmol), **2a** (0.22 mmol), *t*-Bu-P4 (0.04 mmol), additive (1.0 mmol), toluene (0.3 mL), 80 °C, and 18 h. [b]Isolated yields.

Table S2. Effects of base catalysts in the reaction conditions of **1a** and **2a**[a]

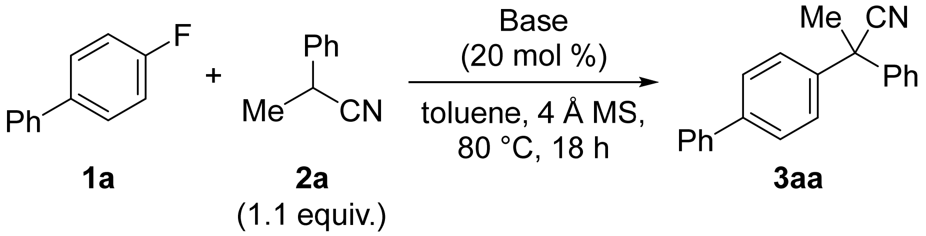

Entry	Base	**3aa** (%)[b]
1	NEt_3	0
2	*i*-Pr_2NEt	0
3	Proton sponge[c]	0
4	DBU[d]	0
5	TBD[e]	0
6	*t*-Bu-P2[f]	0
7	NaH	0
8	LiHMDS[g]	0
9	NaHMDS[g]	0
10	KHMDS[g]	0
11	NaO-*t*-Bu	0
12	KO-*t*-Bu	0
13	KO-*t*-Bu, 18-crown-6	5[h]

[a]**1a** (0.20 mmol), **2a** (0.22 mmol), base (0.04 mmol), 4Å MS (100 mg), toluene (0.3 mL), 80 ºC, 18 h. [b]The yields were determined by ^{1}H-NMR using 1,1,2-trichloroethane as an internal standard. [c]Proton sponge: 1,8-bis-(dimethylamino)naphthalene. [d]DBU: 1,8-diazabicyclo[5.4.0]-7-undecene. [e]TBD: 1,5,7-triazabicyclo-[4.4.0]dec-5-ene. [f]*t*-Bu-P2: 1-(*tert*-butylimino)-1,1,3,3,3-pentakis(dimethylamino)-1λ5, 3λ5-diphosphazene. [g]HMDS: hexamethyldisilazide. [h]Isolated yield.

Table S3. Reactions of (pseudo)halobenzenes (**16**) and **2a**[a]

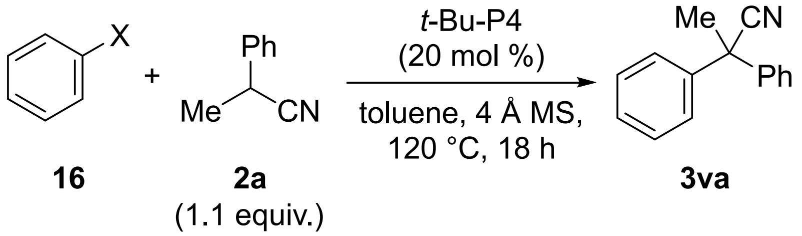

Entry	X	**3va** (%)[b]
1	Cl	5
2	Br	8
3	NO_2	0[c]

[a]**16** (0.20 mmol), **2a** (0.22 mmol), *t*-Bu-P4 (0.04 mmol), 4Å MS (100 mg), toluene (0.3 mL), 120 ºC, 18 h. [b]Isolated yields. [c]The yield was determined by ^{1}H-NMR using 1,1,2-trichloroethane as an internal standard.

Table S4. Reactions of **1a** and carbon nucleophiles other than alkyl cyanide[a]

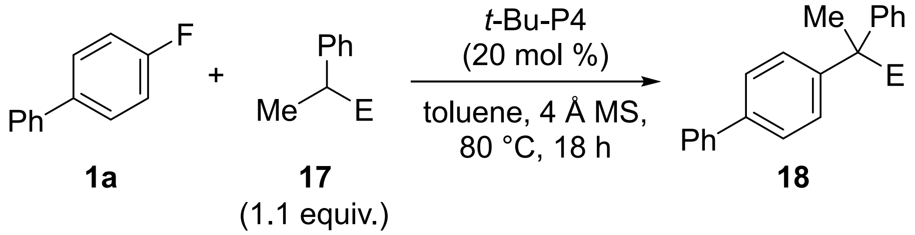

Entry	E	**18** (%)[b]
1	COPh	0
2	CO_2Et	7[c]
3	NO_2	0
4	CF_3	0

[a]**1a** (0.20 mmol), **17** (0.22 mmol), *t*-Bu-P4 (0.04 mmol), 4Å MS (100 mg), toluene (0.3 mL), 80 ºC, 18 h. [b]The yields were determined by ^{1}H-NMR using 1,1,2-trichloroethane as an internal standard. [c]Isolated yield.

Table S5. Optimization of the reaction conditions of **1a** and **4b**[a]

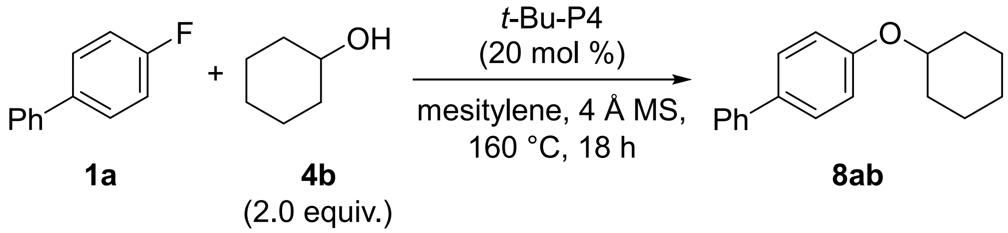

Entry	Deviation from standard conditions	**8ab** (%)[b]
1	without 4Å MS	35
2	3Å MS instead of 4Å MS	69
3	none	85
4	5Å MS instead of 4Å MS	63
5	triglyme as solvent	74
6	DMI as solvent	85
7	10 mol % *t*-Bu-P4	55
8	140 °C	53
9	1.1 equiv. of **4b**	72

[a]Standard conditions: **1a** (0.20 mmol), **4b** (0.40 mmol), *t*-Bu-P4 (0.04 mmol), 4Å MS (100 mg), mesitylene (0.3 mL), 160 °C, and 18 h. [b]Isolated yields.

Table S6. Effects of base catalysts in the reactions of **1a** and **4b**[a]

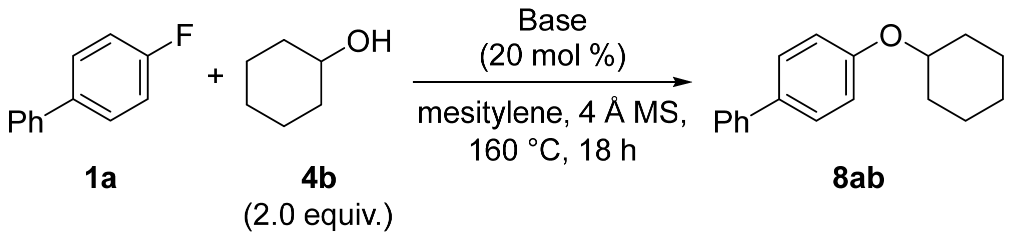

Entry	Base	**8ab** (%)[b]
1	NEt_3	0
2	*i*-Pr_2NEt	0
3	Proton sponge	0
4	DBU	0
5	TBD	0
6	*t*-Bu-P2	0
7	NaH	0
8	LiHMDS	0
9	NaHMDS	0
10	KHMDS	0
11	NaO-*t*-Bu	0
12	KO-*t*-Bu	0
13	KO-*t*-Bu, 18-crown-6	0

[a]**1a** (0.20 mmol), **2a** (0.40 mmol), base (0.04 mmol), 4Å MS (100 mg), mesitylene (0.3 mL), 160 ℃, 18 h. [b]The yields were determined by ^{1}H-NMR using 1,1,2-trichloroethane as an internal standard.

Table S7. Optimization of the reaction conditions of **1a** and **5a**[a]

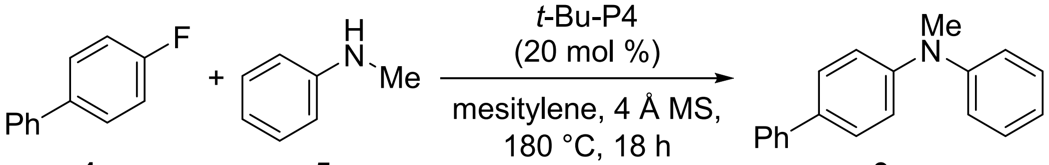

1a **5a** (2.0 equiv.) **9aa**

Entry	Deviation from standard conditions	**9aa** (%)[b]
1	without 4Å MS	36
2	3Å MS instead of 4Å MS	66
3	none	84
4	5Å MS instead of 4Å MS	59
5	triglyme as solvent	77
6	DMI as solvent	74
7	10 mol % *t*-Bu-P4	70
8	160 °C	80

[a]Standard conditions: **1a** (0.20 mmol), **5a** (0.40 mmol), *t*-Bu-P4 (0.04 mmol), 4Å MS (100 mg), mesitylene (0.3 mL), 180 °C, and 18 h. [b]Isolated yields.

Table S8. Effects of base catalysts in the reactions of **1a** and **5a**[a]

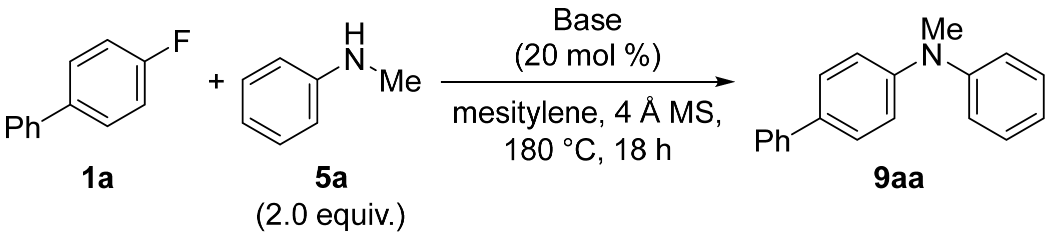

Entry	Base	**9aa** (%)[b]
1	NEt_3	0
2	*i*-Pr_2NEt	0
3	Proton sponge	0
4	DBU	0
5	TBD	0
6	*t*-Bu-P2	0
7	NaH	0
8	LiHMDS	0
9	NaHMDS	0
10	KHMDS	7
11	NaO-*t*-Bu	0
12	KO-*t*-Bu	0
13	KO-*t*-Bu, 18-crown-6	1

[a]**1a** (0.20 mmol), **5a** (0.40 mmol), base (0.04 mmol), 4Å MS (100 mg), mesitylene (0.3 mL), 180 ℃, 18 h. [b]The yields were determined by ^{1}H-NMR using 1,1,2-trichloroethane as an internal standard.

Table S9. Optimization of the reaction conditions of **1a** and **6a**[a]

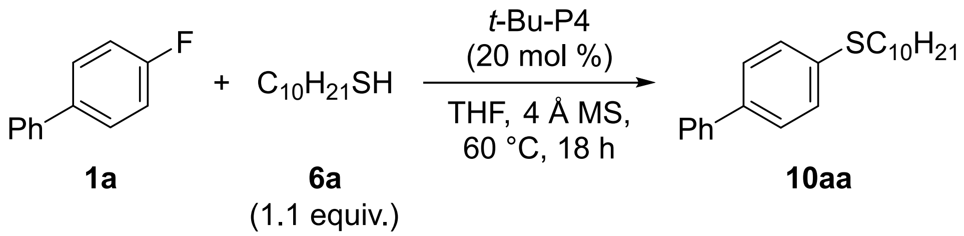

Entry	Deviation from standard conditions	**10aa** (%)[b]
1	without 4Å MS	59
2	3Å MS instead of 4Å MS	91
3	none	91
4	5Å MS instead of 4Å MS	69
5	toluene as solvent	95
6	cyclohexane as solvent	92
7	10 mol % *t*-Bu-P4	84
8	40 °C	87

[a]Standard conditions: **1a** (0.20 mmol), **6a** (0.22 mmol), *t*-Bu-P4 (0.04 mmol), 4Å MS (100 mg), THF (0.3 mL), 60 °C, and 18 h. [b]Isolated yields.

Table S10. Effects of base catalysts in the reactions of **1a** and **6a**[a]

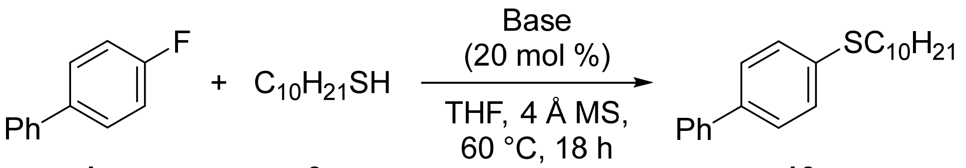

1a **6a** (1.1 equiv.) **10aa**

Entry	Base	**10aa** (%)[b]
1	NEt_3	0
2	*i*-Pr_2NEt	0
3	Proton sponge	0
4	DBU	0
5	TBD	0
6	*t*-Bu-P2	(82)[c]
7	NaH	0
8	LiHMDS	0
9	NaHMDS	0
10	KHMDS	0
11	NaO-*t*-Bu	0
12	KO-*t*-Bu	0
13	KO-*t*-Bu, 18-crown-6	17

[a]**1a** (0.20 mmol), **6a** (0.22 mmol), base (0.04 mmol), 4Å MS (100 mg), THF (0.3 mL), 60 ºC, 18 h. [b]The yields were determined by ^{1}H-NMR using 1,1,2-trichloroethane as an internal standard. [c]Yield in parentheses denotes the isolated yield of **10aa**.

Table S11. Effects of base catalysts in the reactions of **1a** and **7b**[a]

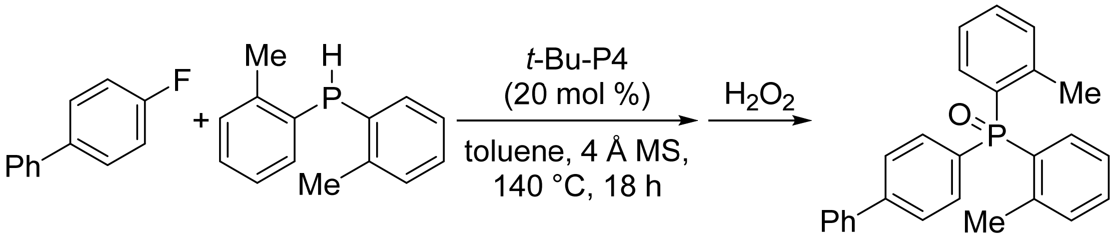

1a **7b** (1.1 equiv.) **11ab**

Entry	Base	**11ab** (%)[b]
1	NEt_3	0
2	*i*-Pr_2NEt	0
3	Proton sponge	0
4	DBU	0
5	TBD	0
6	*t*-Bu-P2	9
7	NaH	trace
8	LiHMDS	10
9	NaHMDS	10
10	KHMDS	9
11	NaO-*t*-Bu	trace
12	KO-*t*-Bu	trace
13	KO-*t*-Bu, 18-crown-6	0

[a]**1a** (0.20 mmol), **7b** (0.22 mmol), base (0.04 mmol), 4Å MS (100 mg), toluene (0.3 mL), 140 ℃, 18 h. The reaction mixtures were treated with 30% H_2O_2 (0.6 mL). [b]The yields were determined by ^{1}H-NMR using 1,1,2-trichloroethane as an internal standard.

Hammet analysis

Procedure for determining the initial rates of substitution reactions of fluoroarenes with 2a.

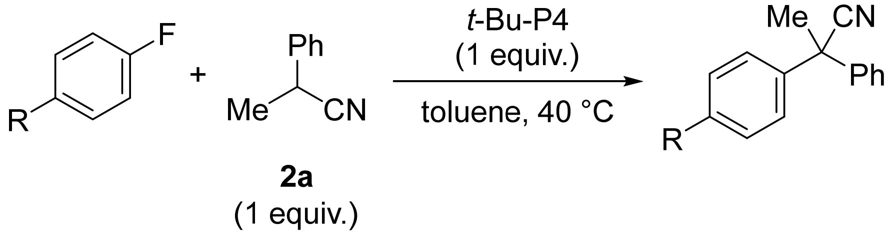

In a glove box under an Ar atmosphere, to a solution of **2a** (0.04 mmol) and **1a**, **1z**, **1B**, or **1K** (0.04 mmol) in toluene (0.6 mL) was added *t*-Bu-P4 solution (50 µL, 0.8 M in hexane, 0.04 mmol) in an oven-dried vial equipped with a stirrer bar. The vial was sealed with a cap containing a Teflon film and taken outside the glove box. Then, the reaction mixture was stirring at 40 °C in a heat block. The yields of products were determined by HPLC analysis to assess the initial rates of the substitution reactions. The Hammett constants (σ_p)[1] of 4-substituted fluoroarenes and the obtained initial rates are shown in Table S12.

Figure S1. Initial rates of the reactions with **2a**

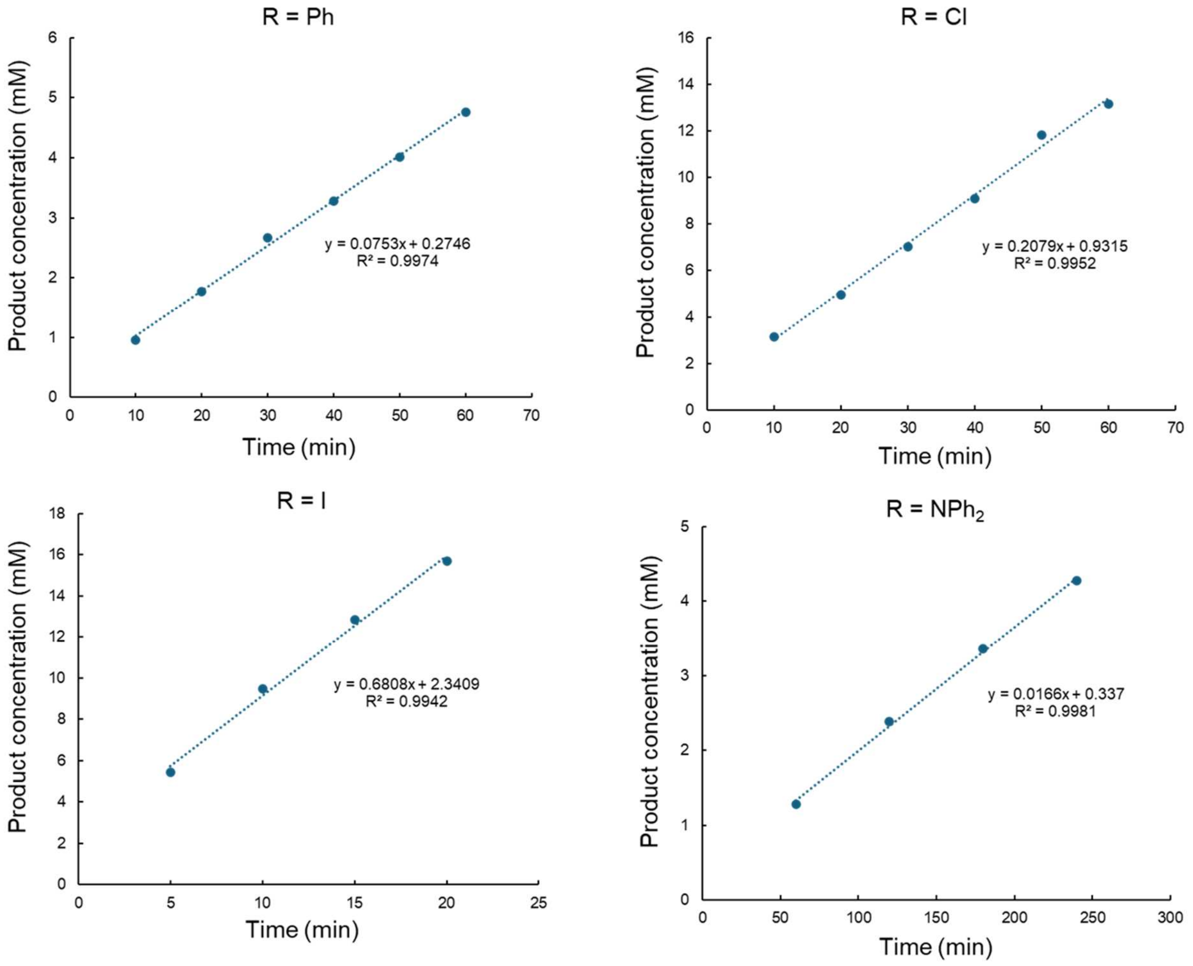

Table S12. Hammett constants and kinetic data

Fluoroarene (R)	Hammett constant (σ_p)	Initial rate (mM/min)	k_R/k_{Ph}	$\log(k_R/k_{Ph})$
**1a** (Ph)	–0.01	0.0753	1.000	0
**1z** (Cl)	0.23	0.2079	2.761	0.441
**1B** (I)	0.28	0.6808	9.041	0.956
**1K** (NPh_2)	–0.22	0.0166	0.221	-0.657

Figure S2. Hammett plot for $\log(k_R/k_{Ph})$ against σ_p

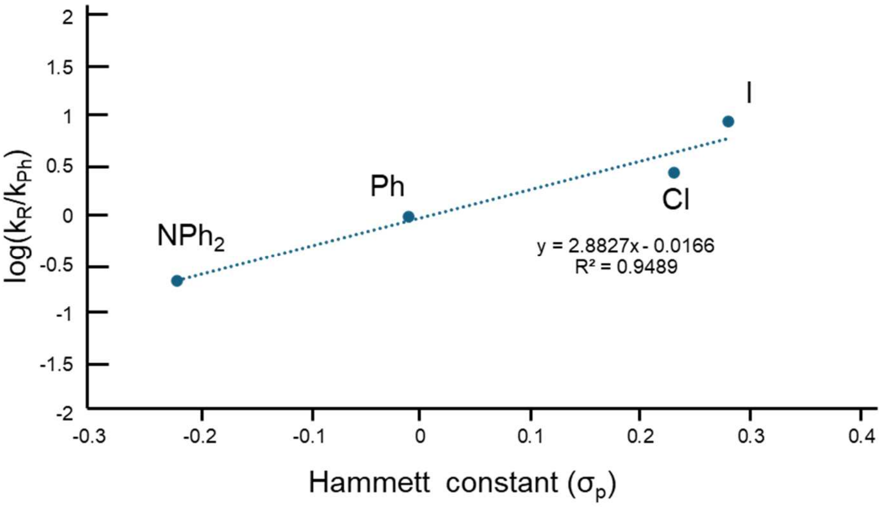

Figure S3. Details for NICS$_{zz}$-scan of (a) **1v**, (b) **Int-2**, (c) **TS-1**, (d) **Int-6**, and (e) **TS-2** calculated at the GIAO/B3LYP/6-311+G(d) level of theory. (A) The z axis for NICS$_{zz}$-scan and z values shown as the purple dots. (B) NICSzz-scan curves with 0.1 Å intervals.

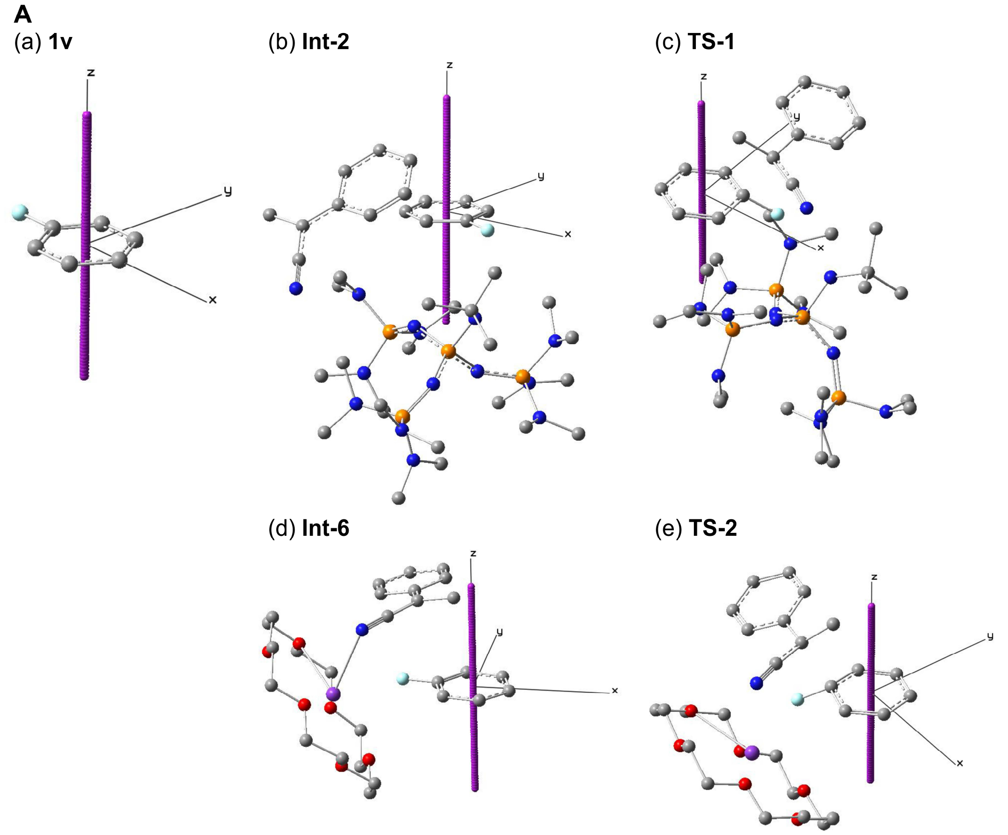

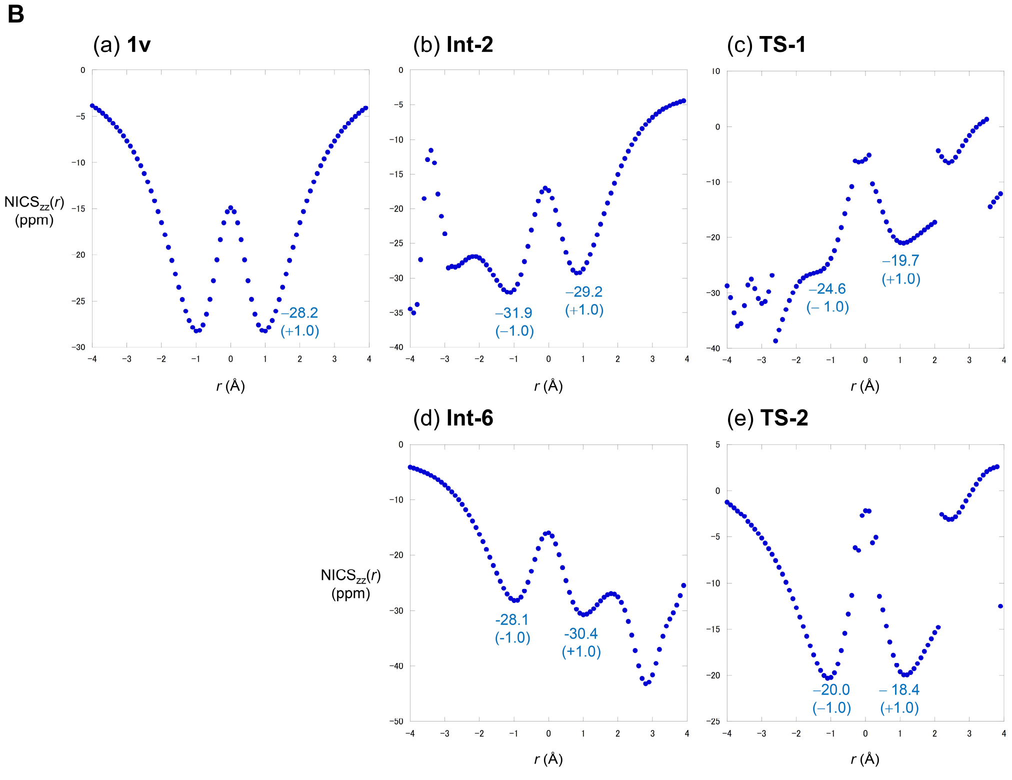

Figure S4. Natural bond orbital (NBO) analysis of **TS-1** calculated at the M06-2X/6-311+g(2d,p) level of theory. The noncovalent stereoelectronic interactions between (a) σ (C1−Cα) bond orbital and π* orbitals, (b) LP (F) orbital and σ* (N−H) bond orbital, and (C) σ (C1−Ca) bond orbital and σ* (C1−F) bond orbital are analyzed. LP refers to lone electron pair. The deletion energies are shown for combinations of each interaction.

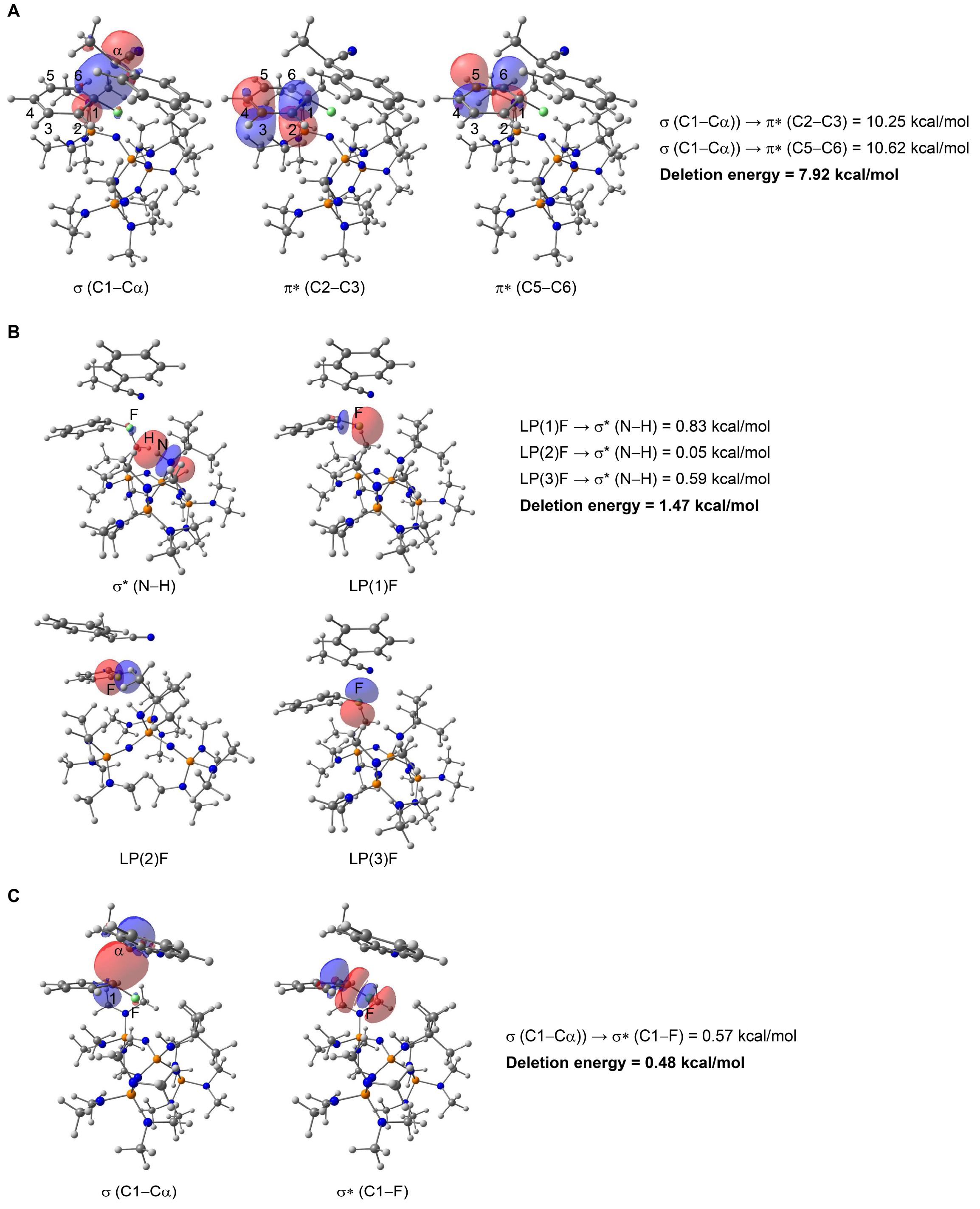

Figure S5. Noncovalent interaction analyses of (a) **Int-1** and (b) **Int-5**. (a) and (b) represent expansion of several representative interactions of Figures 7B-(a) and (b), respectively. Red surface indicates strong repulsive interactions, while green and blue surfaces show weak and strong attractive interactions, respectively.

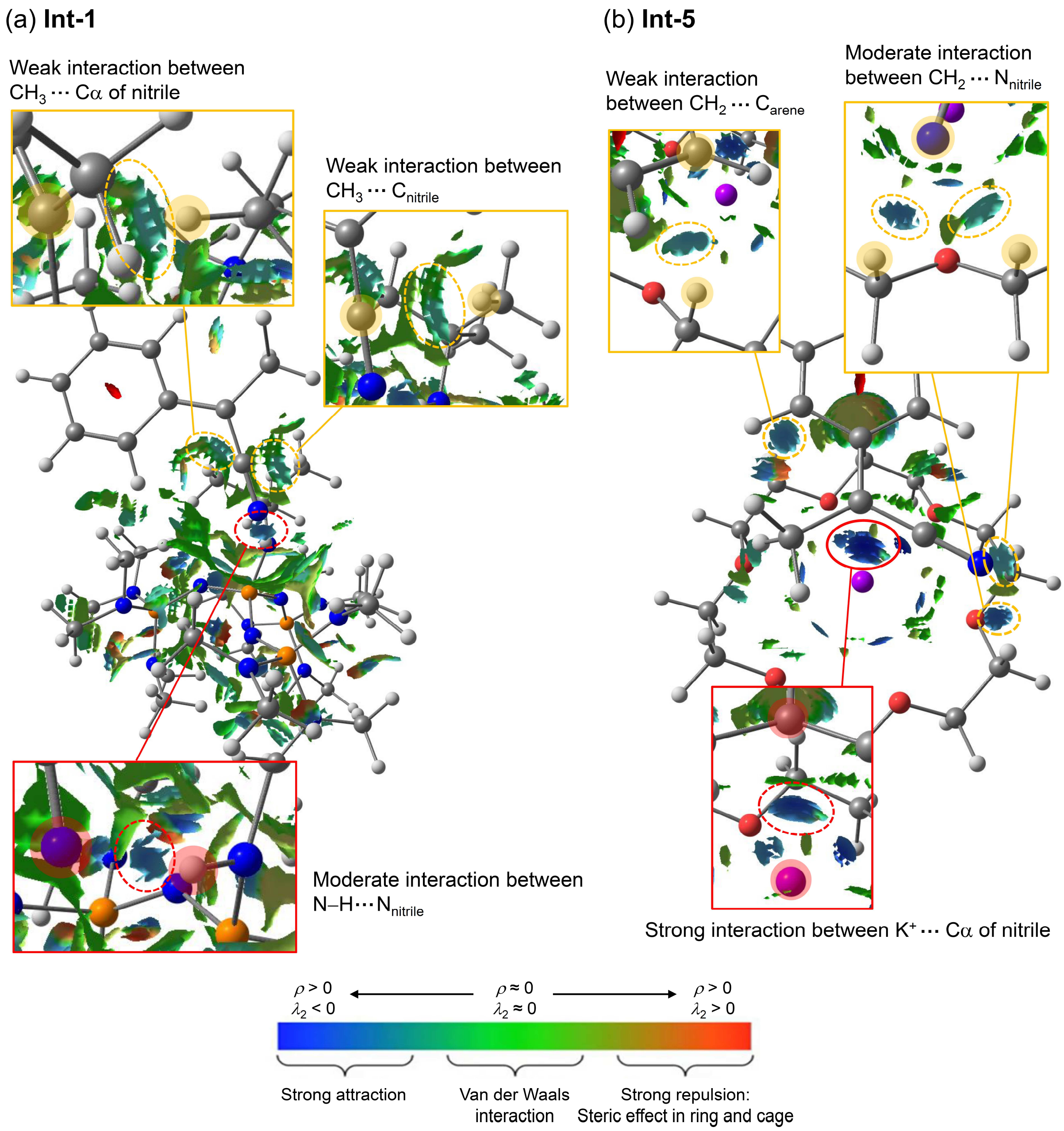

Materials. Unless otherwise noted, materials were purchased from Tokyo Kasei Co., Aldrich Inc. and other commercial suppliers and were used as received. **1i**[2], **1j**[2], **1k**[2], **1x**[3], **1y**[4], **1C**[5], **1D**[6], **1G**[7], **1I**[8], **1J**[9], **1K**[10], **1M**[11], **2b**[12], **2c**[13], **2d**[14], **2e**[15], **2g**[16], **2h**[17], **2i**[18], **2j**[19], **2l**[13], **2m**[20], **2n**[17], **2o**[14], and **2t**[21] were prepared according to the literature procedures. **1s**, **1w**, **1N**, and **1O** were prepared according to the following Schemes S2, S3, S4, and S5, respectively. Flash column chromatography was performed with Kanto silica gel 60 N (spherical, neutral, 70–230 mesh).

Scheme S2. Preparation of **1s**

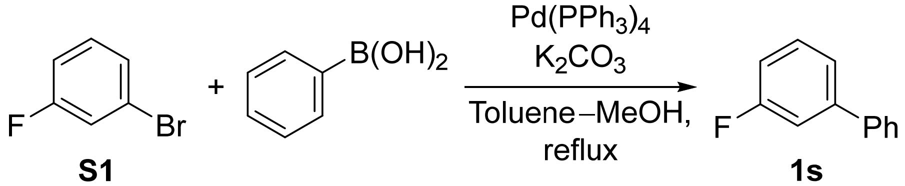

3-Fluoro-1,1'-biphenyl (1s). A solution of $Pd(PPh_3)_4$ (161.5 mg, 0.140 mmol), K_2CO_3 (1.46 g, 10.6 mmol), 3-bromofluorobenzene (**S1**, 888.8 mg, 5.08 mmol), and phenylboronic acid (693.8 mg, 5.69 mmol) in a mixed solvent of toluene (10 mL) and MeOH (5 mL) was refluxed overnight. The mixture was cooled to room temperature, and water was added at 0 °C. The organic materials were extracted with AcOEt, and washed with brine. The organic phase was dried over Na_2SO_4 and concentrated. The residue was purified by silica gel column chromatography (hexane) to give **1s** (840.1 mg, 4.88 mmol, 96 %) as a white solid: Mp 29 °C (EtOH/water) (lit. 26-29 °C[22]). ^{1}H NMR (400 MHz, $CDCl_3$/TMS) δ 7.60-7.55 (m, 2H), 7.45 (t, 2H, J = 7.6 Hz), 7.42-7.34 (m, 3H), 7.32-7.26 (m, 1H), 7.07-7.00 (m, 1H). ^{13}C NMR (100 MHz, $CDCl_3$) δ 163.2 (d, J = 246.9 Hz), 143.5 (d, J = 7.4 Hz), 139.9 (d, J = 1.7 Hz), 130.2 (d, J = 8.2 Hz), 128.8, 127.8, 127.0, 122.7 (d, J = 2.5 Hz), 114.1, 113.9 (d, J = 1.6 Hz). ^{19}F NMR (565 MHz, $CDCl_3$) δ −112.5 - −112.6 (m). LRMS (EI) *m/z*: 172 (M^+). HRMS Calcd. for $C_{12}H_9F$: 172.0688, found: 172.0685. IR (neat): 1610, 1590, 1577, 1481, 1474, 1423, 1260, 1186, 878, 755 cm^{-1}. The spectra data matched those reported in the literature.[22]

Scheme S3. Preparation of **1w**

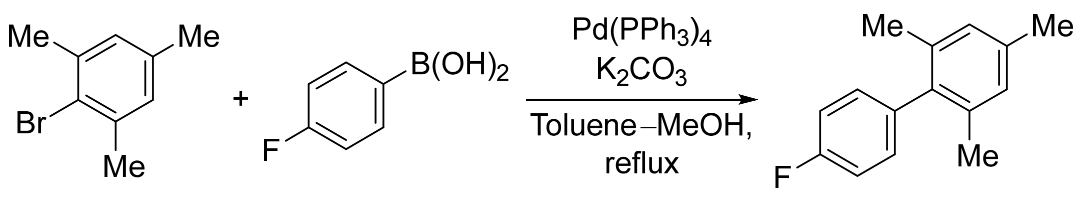

4'-Fluoro-2,4,6-trimethyl-1,1'-biphenyl (1w). A solution of $Pd(PPh_3)_4$ (163.3 mg, 0.141 mmol), K_2CO_3 (1.41 g, 10.2 mmol), 2-bromomesitylene (**S2**, 991.5 mg, 4.98 mmol), and 4-fluorophenylboronic acid (775.3 mg, 5.54 mmol) in a mixed solvent of toluene (10 mL) and MeOH (5 mL) was refluxed overnight. The mixture was cooled to room temperature, and water was added at

0 °C. The organic materials were extracted with AcOEt, and washed with brine. The organic phase was dried over Na_2SO_4 and concentrated. The residue was purified by silica gel column chromatography (hexane) and recrystallization (water/MeOH) to give **1w** (271.1 mg, 1.27 mmol, 25 %) as a white solid: Mp 64 °C (water/MeOH) (lit. 69-71 °C[23]). ^{1}H NMR (400 MHz, $CDCl_3$/TMS) δ 7.12-7.06 (m, 4H), 6.94 (s, 2H), 2.33 (s, 3H), 1.99 (s, 6H). ^{13}C NMR (100 MHz, $CDCl_3$) δ 161.7 (d, J = 244.0 Hz), 138.0, 136.84 (d, J = 3.8 Hz), 136.76, 136.1, 130.8 (d, J = 7.6 Hz), 128.1, 115.3 (d, J = 21.3 Hz), 21.0, 20.7. ^{19}F NMR (565 MHz, $CDCl_3$) δ −115.8 - −115.9 (m). LRMS (EI) *m/z*: 214 (M^+). HRMS Calcd. for $C_{15}H_{15}F$: 214.1158, found: 214.1156. IR (neat): 2927, 2847, 1602, 1479, 1377, 1214, 1087, 1007, 853, 831 cm^{-1}. The spectra data matched those reported in the literature.[23]

Scheme S4. Preparation of **1N**

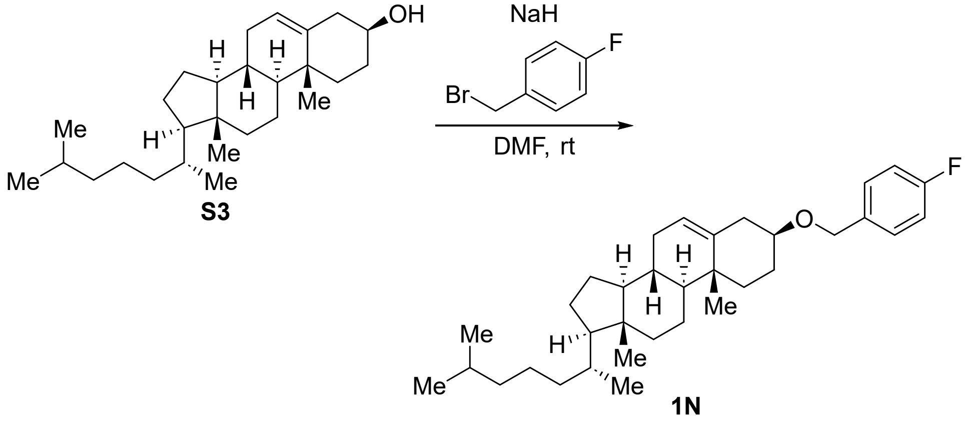

(3*S*,8*S*,9*S*,10*R*,13*R*,14*S*,17*R*)-3-((4-Fluorobenzyl)oxy)-10,13-dimethyl-17-((*R*)-6-methylheptan-2-yl)-2,3,4,7,8,9,10,11,12,13,14,15,16,17-tetradecahydro-1*H*-cyclopenta[*a*]phenanthrene (**1N**). To a solution of cholesterol (1.931 g, 4.99 mmol) in THF (20 mL) was slowly added NaH (60% oil suspension, 311.6 mg, 7.79 mmol) at 0 °C. After stirring at the temperature for 30 min, 4-fluorobenzyl bromide (1.040 g, 5.50 mmol) was added. After stirring at room temperature overnight, the reaction mixture was quenched with saturated NH_4Cl aqueous solution, and the organic materials were extracted with AcOEt. The extract was washed with water and brine, dried over Na_2SO_4, and concentrated. The residue was purified by column chromatography on silica gel (hexane:AcOEt = 10:1) to afford **1N** (664.0 mg, 1.34 mmol, 27%) as a white solid: Mp 137-139 °C: ^{1}H NMR (600 MHz, $CDCl_3$/TMS) δ 7.31 (dd, 2H, J = 5.5, 8.5 Hz), 7.00 (t, 2H, J = 8.5 Hz), 5.37-5.33 (m, 1H), 4.51 (dd, 2H, J = 12.0, 13.1 Hz), 3.26 (sep, 1H, J = 5.3 Hz), 2.43-2.37 (m, 1H), 2.31-2.23 (m, 1H), 2.05-1.76 (m, 5H), 1.61-1.41 (m, 7H), 1.41-1.20 (m, 4H), 1.20-0.93 (m, 12H), 0.93-0.88 (m, 4H), 0.86 (dd, 6H, J = 2.5, 6.5 Hz), 0.68 (s, 3H). ^{13}C NMR (150 MHz, $CDCl_3$) δ 162.2 (d, J = 243.5 Hz), 140.9, 134.8 (d, J = 3.0 Hz), 129.2 (d, J = 8.0 Hz), 121.7, 115.1 (d, J = 21.9 Hz), 78.7, 69.2, 56.8, 56.2, 50.2, 42.3, 39.8, 39.5, 39.1, 37.2, 36.9, 36.2, 35.8, 31.93, 31.88, 28.4, 28.2, 28.0, 24.3, 23.8, 22.8, 22.5, 21.1, 19.4, 18.7, 11.8. ^{19}F NMR (375 MHz, $CDCl_3$) δ −114.6 - −114.7 (m). LRMS (EI) *m/z*: 494 (M^+). HRMS Calcd.

for $C_{34}H_{51}FO$: 494.3924, found: 494.3914. IR (neat): 2926, 2236, 1740, 1456, 1374, 1257, 1087, 757 cm^{-1}.

Scheme S5. Preparation of **1O**

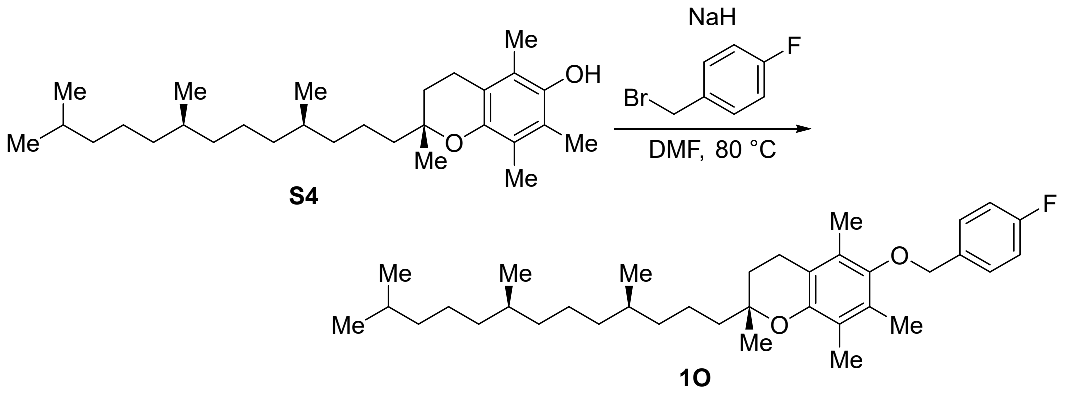

(*R*)-6-((4-Fluorobenzyl)oxy)-2,5,7,8-tetramethyl-2-((4*R*,8*R*)-4,8,12-trimethyltridecyl)chromane (**1O**). The following synthetic procedure of **1O** was referred to the literature reported by Shibata et al.[11] To a solution of D-α-Tocopherol (2.260 g, 5.25 mmol) in DMF (17 mL) was slowly added NaH (60% oil suspension, 244.9 mg, 6.13 mmol) at 0 °C. After stirring at room temperature for 30 min, 4-fluorobenzyl bromide (1.047 g, 5.54 mmol) was added. Then, after stirring at 80 °C overnight, the reaction mixture was quenched with saturated NH_4Cl aqueous solution, and the organic materials were extracted with AcOEt. The extract was washed with water and brine, dried over Na_2SO_4, and concentrated. The residue was purified by column chromatography on silica gel (hexane:AcOEt = 100:1) to afford **1O** (2.613 g, 4.85 mmol, 92%) as a colorless oil. ^{1}H NMR (600 MHz, $CDCl_3$/TMS) δ 7.50 (dd, 2H, J = 8.0, 5.5 Hz), 7.12 (t, 2H, J = 8.5 Hz), 4.71 (s, 2H), 2.65 (t, 2H, J = 6.8 Hz), 2.27 (s, 3H), 2.21 (s, 3H), 2.18 (s, 3H), 1.92-1.80 (m, 2H), 1.68-1.10 (m, 24H), 0.98-0.91 (m, 12H). ^{13}C NMR (150 MHz, $CDCl_3$) δ 162.4 (d, J = 244.5 Hz), 147.98, 147.96, 133.8 (d, J = 3.0 Hz), 129.5 (d, J = 8.0 Hz), 127.8, 125.8, 123.0, 117.6, 115.2 (d, J = 21.9 Hz), 74.8, 73.9, 40.0, 39.4, 37.48, 37.47, 37.4, 37.3, 32.8, 32.7, 31.3, 28.0, 24.8, 24.4, 23.9, 22.7, 22.6, 21.0, 20.7, 19.75, 19.66, 12.8, 12.0, 11.8. ^{19}F NMR (375 MHz, $CDCl_3$) δ −114.0 - −114.1 (m). LRMS (EI) *m/z*: 538 (M^+). HRMS Calcd. for $C_{36}H_{55}FO_2$: 538.4186, found: 538.4192. IR (neat): 2937, 2925, 1512, 1453, 1411, 1258, 1226, 1087, 861 cm^{-1}. The spectra data matched those reported in the literature.[11]

General procedure of *t*-Bu-P4-catalyzed alkylation of fluoroarenes (Table 1 and Figure 2).

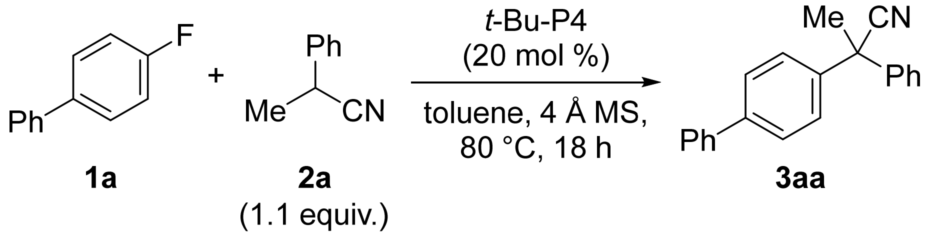

2-([1,1'-Biphenyl]-4-yl)-2-phenylpropanenitrile (3aa). In a glove box under an Ar atmosphere, to a mixture of **1a** (34.5 mg, 0.200 mmol) and **2a** (28.5 mg, 0.217 mmol) in toluene (0.3 mL) were added *t*-Bu-P4 solution (50 µL, 0.8 M in hexane, 0.04 mmol) and 4 Å molecular sieves (100 mg) in an oven-dried vial equipped with a stirrer bar. The vial was sealed with a cap containing an inner Teflon film and taken outside the glove box. After stirring at 80 °C for 18 h, saturated NH_4Cl aqueous solution (1 mL) was added to the reaction mixture at 0 ºC. The mixture was extracted with AcOEt (10 mL × 3). The combined organic layers were collected, washed with brine (10 mL), dried over Na_2SO_4, and concentrated. The crude material was purified by preparative thin-layer chromatography of silica gel (toluene), to afford **3aa** (51.4 mg, 0.181 mmol, 91%) as a white solid: Mp 69 °C (hexane/CH_2Cl_2). ^{1}H NMR (400 MHz, $CDCl_3$/TMS) δ 7.62-7.54 (m, 4H), 7.48-7.30 (m, 10H), 2.13 (s, 3H). ^{13}C NMR (100 MHz, DMSO-*d*6) δ 141.0, 140.2, 139.7, 139.2, 129.0, 128.9, 127.9, 127.7, 127.2, 126.8, 126.7, 126.3, 123.3, 45.6, 27.0. LRMS (EI) *m/z*: 283 (M^+). HRMS Calcd. for $C_{21}H_{17}N$: 283.1361, found: 283.1363. IR (neat): 3032, 2998, 2234, 1489, 1404, 1069, 1006, 840, 764, 728 cm^{-1}.

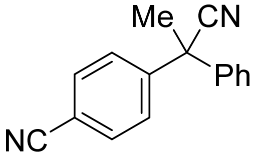

4-(1-Cyano-1-phenylethyl)benzonitrile (3ba). According to the general procedure analogous to that described for **3aa**, except that the crude material was purified by column chromatography on silica gel (hexane:CH_2Cl_2 =1:2), **3ba** (41.0 mg, 0.177 mmol, 86%) was obtained from **1b** (24.8 mg, 0.205 mmol) as a colorless oil. ^{1}H NMR (400 MHz, $CDCl_3$/TMS) δ 7.67 (d, 2H, J = 8.8 Hz), 7.50 (d, 2H, J = 8.8 Hz), 7.43-7.32 (m, 5H), 2.11 (s, 3H). ^{13}C NMR (100 MHz, $CDCl_3$) δ 146.5, 139.7, 132.7, 129.2, 128.5, 127.4, 126.5, 122.3, 118.0, 112.1, 46.2, 27.7. LRMS (EI) *m/z*: 232 (M^+). HRMS Calcd. for $C_{16}H_{12}N_2$: 232.1000, found: 232.1002. IR (neat): 3065, 2991, 2943, 2229, 1609, 1494, 1448, 1409, 1208, 1070, 1019, 825, 752 cm^{-1}. The spectra data matched those reported in the literature.[14]

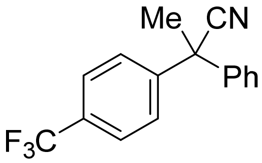

2-Phenyl-2-(4-(trifluoromethyl)phenyl)propanenitrile (3ca). According to the general procedure analogous to that described for **3aa**, **3ca** (47.3 mg, 0.172 mmol, 87%) was obtained from **1c** (32.3 mg, 0.197 mmol) as a colorless oil. ^{1}H NMR (400 MHz, $CDCl_3$/TMS) δ 7.63 (d, 2H, J = 8.3 Hz), 7.51 (d, 2H, J = 8.3 Hz), 7.43-7.30 (m, 5H), 2.12 (s, 3H). ^{13}C NMR (100 MHz, $CDCl_3$) δ 145.3, 140.2, 130.2 (q, J = 32.9 Hz), 129.1, 128.3, 127.1, 126.5, 125.9 (q, J = 3.8 Hz), 123.8 (q, J = 273.5 Hz), 122.7, 46.1, 27.8. ^{19}F NMR (565 MHz, $CDCl_3$) δ –62.1 (s). LRMS (EI) *m/z*: 275 (M^+). HRMS Calcd. for $C_{16}H_{12}F_3N$: 275.0922, found: 275.0931. IR (neat): 3078, 2992, 2238, 1619, 1449, 1327, 1171, 1126, 1072,

1017, 760 cm^{-1}.

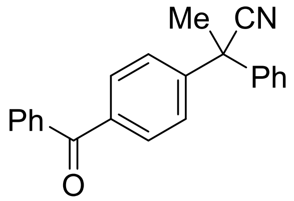

2-(4-Benzoylphenyl)-2-phenylpropanenitrile (3da). According to the general procedure analogous to that described for **3aa**, except that the crude material was purified by column chromatography on silica gel (hexane:AcOEt =10:1) **3da** (57.3 mg, 0.184 mmol, 93%) was obtained from **1d** (39.7 mg, 0.198 mmol) as a white solid: Mp 57 °C. ^{1}H NMR (400 MHz, $CDCl_3$/TMS) δ7.83-7.78 (m, 4H), 7.61 (t, 1H, J = 7.4 Hz), 7.53-7.46 (m, 4H), 7.42-7.32 (m. 5H), 2.14 (s, 3H). ^{13}C NMR (100 MHz, DMSO-d_6) δ195.0, 145.3, 140.4, 136.7, 136.5, 132.8, 130.2, 129.5, 129.1, 128.5, 128.1, 126.5, 126.3, 122.9, 45.9, 26.8. LRMS (EI) *m/z*: 311 (M^+). HRMS Calcd. for $C_{22}H_{17}NO$: 311.1310, found: 311.1314. IR (neat): 3061, 2988, 2238, 1659, 1598, 1447, 1317, 1278, 939, 761 cm^{-1}. The spectra data matched those reported in the literature.[14]

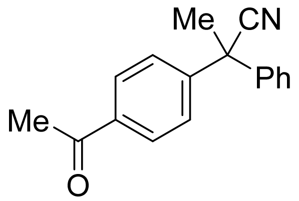

2-(4-Acetylphenyl)-2-phenylpropanenitrile (3ea). According to the general procedure analogous to that described for **3aa**, except that the reaction was conducted at 40 °C and the crude material was purified by column chromatography on silica gel (hexane:AcOEt =5:1), **3ea** (36.4 mg, 0.146 mmol, 73%) was obtained from **1e** (27.8 mg, 0.201 mmol) as a colorless oil. ^{1}H NMR (400 MHz, $CDCl_3$/TMS) δ7.95 (d, 2H, J = 8.8 Hz), 7.49 (d, 2H, J = 8.3 Hz), 7.42-7.30 (m, 5H), 2.60 (s, 3H), 2.12 (s, 3H). ^{13}C NMR (150 MHz, $CDCl_3$) δ197.1, 146.2, 140.3, 136.5, 129.0, 128.8, 128.2, 126.8, 126.5, 122.7, 46.1, 27.7, 26.5. LRMS (EI) *m/z*: 249 (M^+). HRMS Calcd. for $C_{17}H_{15}NO$: 249.1154, found: 249.1150. IR (neat): 3001, 2237, 1684, 1607, 1407, 1359, 1268, 959, 822, 764 cm^{-1}. The spectra data matched those reported in the literature.[14]

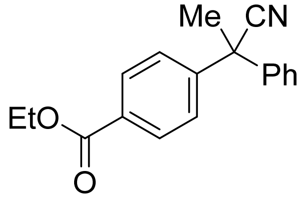

Ethyl 4-(1-cyano-1-phenylethyl)benzoate (3fa). According to the general procedure analogous to

that described for **3aa**, except that the reaction was conducted at 60 °C and the crude material was purified by preparative thin-layer chromatography of silica gel (toluene:AcOEt = 10:1) and GPC ($CHCl_3$), **3fa** (42.9 mg, 0.154 mmol, 72%) was obtained from **1f** (35.7 mg, 0.212 mmol) as a colorless oil. ^{1}H NMR (400 MHz, $CDCl_3$/TMS) δ 8.03 (d, 2H, J = 8.8 Hz), 7.46 (d, 2H, J = 8.8 Hz), 7.42-7.28 (m, 5H), 4.38 (q, 2H, J = 7.1 Hz), 2.11 (s, 3H), 1.39 (t, 3H, J = 7.3 Hz). ^{13}C NMR (100 MHz, $CDCl_3$) δ 165.8, 145.9, 140.4, 130.13, 130.06, 129.0, 128.1, 126.6, 126.5, 122.8, 61.1, 46.1, 27.8, 14.2. LRMS (EI) *m/z*: 279 (M^+). HRMS Calcd. for $C_{18}H_{17}NO_2$: 279.1259, found: 279.1242. IR (neat): 2998, 2944, 2238, 1718, 1610, 1276, 1107, 1018, 855, 756 cm^{-1}. The spectra data matched those reported in the literature.[24]

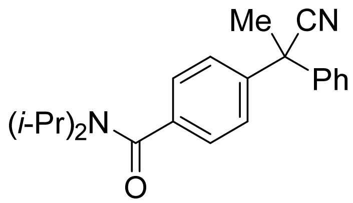

4-(1-Cyano-1-phenylethyl)-*N*,*N*-diisopropylbenzamide (3ga). According to the general procedure analogous to that described for **1aa**, except that the crude material was purified by column chromatography on silica gel (hexane:AcOEt =3:1), **3ga** (57.3 mg, 0.171 mmol, 87%) was obtained from **1g** (44.1 mg, 0.197 mmol) as a colorless oil. ^{1}H NMR (400 MHz, $CDCl_3$/TMS) δ 7.41-7.29 (m, 9H), 3.83 (brs, 1H), 3.53 (brs, 1H), 2.09 (s, 3H), 1.52 (brs, 6H), 1.16 (brs, 6H). ^{13}C NMR (150 MHz, $CDCl_3$) δ 170.1, 141.6, 140.7, 138.5, 128.9, 128.0, 126.8, 126.6, 126.2, 123.1, 50.8 (brs), 46.0, 45.8 (brs), 28.0, 20.6. LRMS (EI) *m/z*: 334.2 (M^+). HRMS Calcd. for $C_{22}H_{26}N_2O$: 334.2045, found: 334.2035. IR (neat): 2971, 2933, 2238, 1623, 1441, 1371, 1340, 1212, 1162, 1037, 1018, 917, 758 cm^{-1}.

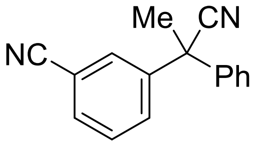

3-(1-Cyano-1-phenylethyl)benzonitrile (3ha). According to the general procedure analogous to that described for **3aa**, except that the crude material was purified by preparative thin-layer chromatography of silica gel (toluene:AcOEt = 4:1), **3ha** (41.0 mg, 0.177 mmol, 90%) was obtained from **1h** (23.7 mg, 0.196 mmol) as a white solid: Mp 70 °C (hexane/CH_2Cl_2). ^{1}H NMR (400 MHz, $CDCl_3$/TMS) δ 7.67 (d, 1H, J = 7.8 Hz), 7.65-7.60 (m, 2H), 7.51 (t, 1H, J = 7.8 Hz), 7.44-7.33 (m, 5H), 2.11 (s, 3H). ^{13}C NMR (100 MHz, $CDCl_3$) δ 143.0, 139.6, 131.6, 131.1, 130.0, 129.8, 129.2, 128.4, 126.4, 122.3, 118.1, 113.1, 45.8, 27.7. LRMS (EI) *m/z*: 232 (M^+). HRMS Calcd. for $C_{16}H_{12}N_2$: 232.1000, found: 232.0998. IR (neat): 3067, 2252, 2231, 1484, 1447, 1182, 1076, 1029, 799, 750 cm^{-1}.

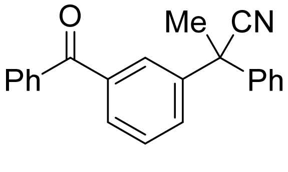

2-(3-Benzoylphenyl)-2-phenylpropanenitrile (3ia). According to the general procedure analogous to that described for **3aa**, except that the crude material was purified by column chromatography on silica gel (hexane:AcOEt =10:1), **3ia** (58.0 mg, 0.186 mmol, 93%) was obtained from **1i** (39.8 mg, 0.199 mmol) as a yellow oil. ^{1}H NMR (400 MHz, $CDCl_3$/TMS) δ 7.81 (s, H), 7.77-7.71 (m, 3H), 7.64 (d, 1H, J = 8.3 Hz), 7.59 (t, 1H, J = 7.3 Hz), 7.52-7.42 (m, 3H), 7.41-7.30 (m, 5H), 2.13 (s, 3H). ^{13}C NMR (100 MHz, $CDCl_3$) δ 195.7, 141.7, 140.6, 138.0, 136.9, 132.6, 130.6, 129.9, 129.6, 128.9, 128.8, 128.3, 128.0, 127.8, 126.4, 122.8, 46.0, 27.8. LRMS (EI) *m/z*: 311 (M^+). HRMS Calcd. for $C_{22}H_{17}NO$: 311.1310, found: 311.1314. IR (neat): 3066, 2990, 2237, 1660, 1597, 1448, 1317, 1278, 970, 764 cm^{-1}.

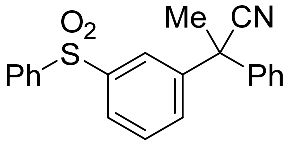

2-Phenyl-2-(3-(phenylsulfonyl)phenyl)propanenitrile (3ja). According to the general procedure analogous to that described for **3aa**, except that the crude material was purified by column chromatography on silica gel (hexane:AcOEt =5:1), **3ja** (63.0 mg, 0.183 mmol, 91%) was obtained from **1j** (47.1 mg, 0.199 mmol) as a white solid: Mp 128 °C (hexane/CH_2Cl_2). ^{1}H NMR (400 MHz, $CDCl_3$/TMS) δ 7.95 (s, 1H), 7.90 (d, 2H, J = 7.3 Hz), 7.87 (d, 1H, J = 7.8 Hz), 7.61-7.56 (m, 2H), 7.54-7.47 (m, 3H), 7.40-7.29 (m, 5H), 2.11 (s, 3H). ^{13}C NMR (100 MHz, $CDCl_3$) δ 143.1, 142.4, 140.9, 139.8, 133.4, 131.6, 129.9, 129.3, 129.0, 128.3, 127.5, 127.2, 126.4, 125.2, 122.4, 45.9, 27.8. LRMS (EI) *m/z*: 347 (M^+). HRMS Calcd. for $C_{21}H_{17}NO_2S$: 347.0980, found: 347.0963. IR (neat): 2960, 2236, 1598, 1448, 1303, 1152, 1104, 800, 744, 701 cm^{-1}.

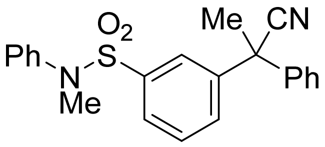

3-(1-Cyano-1-phenylethyl)-*N*-methyl-*N*-phenylbenzenesulfonamide (3ka). According to the general procedure analogous to that described for **3aa**, except that the crude material was purified by column chromatography on silica gel (hexane:AcOEt =5:1), **3ka** (73.6 mg, 0.195 mmol, 97%) was obtained from **1k** (53.2 mg, 0.200 mmol) as a white solid: Mp 102 °C (hexane/CH_2Cl_2). ^{1}H NMR (400 MHz, $CDCl_3$/TMS) δ 7.65 (d, 1H, J = 7.8 Hz), 7.59 (d, 1H, J = 7.8 Hz), 7.49 (t, 1H, J = 7.9 Hz), 7.41-

7.24 (m, 9H), 7.06-7.02 (m, 2H), 3.14 (s, 3H), 1.97 (s, 3H). ^{13}C NMR (100 MHz, $CDCl_3$) δ 142.3, 141.0, 140.0, 137.0, 131.2, 129.4, 129.0, 128.8, 128.2, 127.4, 127.1, 126.3, 126.2, 125.4, 122.3, 45.8, 37.9, 27.6. LRMS (EI) *m/z*: 376 (M^+). HRMS Calcd. for $C_{22}H_{20}N_2O_2S$: 376.1245, found: 376.1244. IR (neat): 2936, 2238, 1597, 1495, 1446, 1349, 1150, 1064, 855, 767 cm^{-1}.

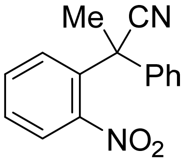

2-(2-Nitrophenyl)-2-phenylpropanenitrile (3la). According to the general procedure analogous to that described for **3aa**, except that the crude material was purified by column chromatography on silica gel (hexane:AcOEt =10:1), **3la** (42.0 mg, 0.166 mmol, 81%) was obtained from **1l** (29.0 mg, 0.206 mmol) as a yellow oil. ^{1}H NMR (400 MHz, $CDCl_3$/TMS) δ 7.88 (dd, 1H, *J* = 8.1, 1.2 Hz), 7.76 (dd, 1H, *J* = 8.3, 1.5 Hz), 7.71 (td, 1H, *J* = 7.8, 1.5 Hz), 7.56 (td, 1H, *J* = 7.8, 1.0 Hz), 7.40-7.20 (m, 5H), 2.24 (s, 3H). ^{13}C NMR (150 MHz, $CDCl_3$) δ 149.7, 139.2, 133.3, 132.7, 129.7, 129.3, 128.9, 128.2, 126.0, 125.9, 121.4, 44.9, 29.6. LRMS (EI) *m/z*: 252 (M^+). HRMS Calcd. for $C_{15}H_{12}N_2O_2$: 252.0899, found: 252.0917. IR (neat): 3062, 2992, 2241, 1531, 1449, 1359, 1200, 1032, 859, 746 cm^{-1}.

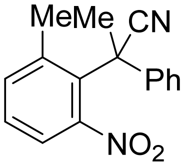

2-(2-Methyl-6-nitrophenyl)-2-phenylpropanenitrile (3ma). According to the general procedure analogous to that described for **3aa**, **3ma** (36.0 mg, 0.135 mmol, 69%) was obtained from **1m** (30.6 mg, 0.197 mmol) as a yellow oil. ^{1}H NMR (400 MHz, $CDCl_3$/TMS) δ 7.42-7.30 (m, 8H), 2.27 (s, 3H), 2.17 (s, 3H). ^{13}C NMR (100 MHz, $CDCl_3$) δ 152.1, 141.0, 140.6, 135.3, 129.1, 129.0, 128.8, 128.2, 126.1, 122.4, 121.7, 45.6, 26.8, 22.4. LRMS (EI) *m/z*: 266 (M^+). HRMS Calcd. for $C_{16}H_{14}N_2O_2$: 266.1055, found: 266.1032. IR (neat): 3067, 2997, 2937, 2234, 1533, 1449, 1364, 1031, 796, 763 cm^{-1}.

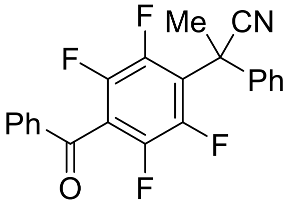

2-(4-Benzoyl-2,3,5,6-tetrafluorophenyl)-2-phenylpropanenitrile (3na). According to the general procedure analogous to that described for **3aa**, except that the reaction was conducted at 60 °C and the crude material was purified by preparative thin-layer chromatography of silica gel (toluene:AcOEt =

3:1), **3na** (50.9 mg, 0.133 mmol, 67%) was obtained from **1n** (54.1 mg, 0.199 mmol) as a colorless oil. ^{1}H NMR (400 MHz, $CDCl_3$/TMS) δ7.87 (d, 2H, J = 7.8 Hz), 7.70 (t, 1H, J = 7.6 Hz), 7.55 (t, 2H, J = 8.0 Hz), 7.50-7.35 (m, 5H), 2.31 (t, 3H, J = 2.9 Hz). ^{13}C NMR (150 MHz, $CDCl_3$) δ 185.4, 145.8-144.0 (m), 144.7-142.9 (m), 139.6, 135.6, 135.1, 129.7, 129.3, 129.1, 128.6, 125.2, 120.6 (t, J_{CF} = 11.5 Hz), 120.2, 119.3 (t, J_{CF} = 20.5 Hz), 43.0, 28.9 (t, J_{CF} = 5.5 Hz). ^{19}F NMR (565 MHz, $CDCl_3$) δ−134.86 - −134.93 (m), −138.5 - −138.6 (m). LRMS (EI) *m/z*: 383 (M^+). HRMS Calcd. for $C_{22}H_{13}F_4NO$: 383.0933, found: 383.0938. IR (neat): 3061, 2242, 1679, 1597, 1472, 1298, 1235, 975, 825, 751 cm^{-1}.

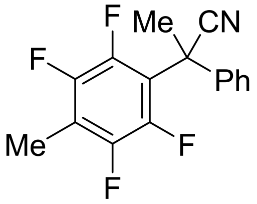

2-Phenyl-2-(2,3,5,6-tetrafluoro-4-methylphenyl)propanenitrile (3oa). According to the general procedure analogous to that described for **3aa**, except that the reaction was conducted at 60 °C and the crude material was purified by preparative thin-layer chromatography of silica gel (toluene:AcOEt = 3:1), **3oa** (34.9 mg, 0.119 mmol, 60%) was obtained from **1o** (36.2 mg, 0.199 mmol) as a colorless oil. ^{1}H NMR (400 MHz, $CDCl_3$/TMS) δ7.50-7.26 (m, 5H), 2.28 (t, 3H, J = 1.7 Hz), 2.24 (t, 3H, J = 3.2 Hz). ^{13}C NMR (150 MHz, $CDCl_3$) δ 146.5-144.7 (m), 145.6-143.8 (m), 140.4, 129.1, 128.3, 125.1, 117.1 (t, J_{CF} = 19.2 Hz), 115.7 (t, J_{CF} = 11.8 Hz), 42.6, 29.1 (t, J_{CF} = 5.4 Hz), 7.6. ^{19}F NMR (565 MHz, $CDCl_3$) δ−138.5 - −138.6 (m), −141.67 - −142.73(m). LRMS (EI) *m/z*: 293 (M^+). HRMS Calcd. for $C_{16}H_{11}F_4N$: 293.0828, found: 293.0802. IR (neat): 3031, 2953, 2244, 1483, 1473, 1283, 1125, 1072, 913, 758 cm^{-1}.

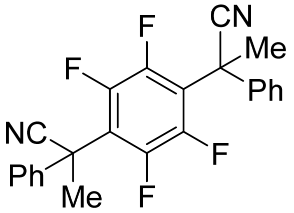

2,2'-(Perfluoro-1,4-phenylene)bis(2-phenylpropanenitrile) (3pa). According to the general procedure analogous to that described for **3aa**, except that the reaction was conducted with **2a** (54.9 mg, 0.419 mmol) and the crude material was purified by column chromatography on silica gel (hexane:AcOEt = 88:12 to 67:33), a diastereomeric mixture of **3pa** (60.2 mg, 0.147 mmol, 73%) was obtained from **1p** (37.4 mg, 0.201 mmol) as white solid. The ratio of diastereomers was determined to be Isomer A:Isomer B = 46:54 by the ^{19}F-NMR analysis of the product. The diastereomers were separable by recycling preparative HPLC (toluene).

Isomer A (26.3 mg, 0.0644 mmol, 32%) white solid: Mp 183-184 °C. ^{1}H NMR (600 MHz, $CDCl_3$/TMS) δ 7.45-7.39 (m, 8H), 7.39-7.33 (m, 2H), 2.25 (s, 6H). ^{13}C NMR (150 MHz, $CDCl_3$) δ

146.4-144.4 (m), 139.5, 129.3, 128.6, 125.2, 120.2, 119.5-119.2 (m), 42.8, 28.8. ^{19}F NMR (376 MHz, $CDCl_3$) δ −135.4. LRMS (EI) *m/z* 408 (M^+). HRMS Calcd. for $C_{24}H_{16}F_4N_2$: 408.1250, found: 408.1239. IR (neat): 2963, 2920, 2857, 2238, 1960, 1881, 1806, 1599, 1481, 1465, 1450, 1381, 1336, 1315, 1288, 1201, 1191, 1150, 1093, 1063, 1026, 958, 916, 871, 844, 764, 746 cm^{-1}.

Isomer B: (21.7 mg, 0.0531 mmol, 26%) colorless oil. ^{1}H NMR (600 MHz, $CDCl_3$/TMS) δ 7.48-7.30 (m, 10H), 2.26 (s, 6H). ^{13}C NMR (150 MHz, $CDCl_3$) δ 146.4-144.4 (m), 139.5, 129.3, 128.6, 125.2, 120.2, 119.5-119.2 (m), 42.8, 28.8. ^{19}F NMR (376 MHz, $CDCl_3$) δ −135.5. LRMS (EI) *m/z* 408 (M^+). HRMS Calcd. for $C_{24}H_{16}F_4N_2$: 408.1250, found: 408.1243. IR (neat): 3064, 3030, 3001, 2242, 1954, 1884, 1804, 1735, 1600, 1494, 1473, 1449, 1387, 1336, 1285, 1266, 1244, 1206, 1153, 1065, 1027, 993, 962, 874, 763, 737 cm^{-1}.

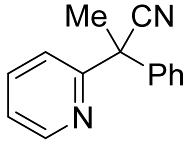

2-Phenyl-2-(pyridin-2-yl)propanenitrile (3qa). According to the general procedure analogous to that described for **3aa**, except that the crude material was purified by column chromatography on silica gel (hexane:AcOEt =10:1), **3qa** (34.3 mg, 0.165 mmol, 77%) was obtained from **1q** (20.9 mg, 0.215 mmol) as a yellow oil. ^{1}H NMR (400 MHz, $CDCl_3$/TMS) δ 8.63 (d, 1H, *J* = 4.9 Hz), 7.67 (dt, 1H, *J* = 1.9, 7.8 Hz), 7.49-7.41 (m, 3H), 7.40-7.26 (m, 3H), 7.23 (dd, 1H, *J* = 6.8, 4.9 Hz), 2.18 (s, 3H). ^{13}C NMR (100 MHz, $CDCl_3$) δ 159.1, 149.3, 140.4, 137.1, 128.9, 127.9, 126.3, 123.0, 122.7, 121.6, 48.7, 26.8. LRMS (EI) *m/z*: 208 (M^+). HRMS Calcd. for $C_{14}H_{12}N_2$: 208.1000, found: 208.0985. IR (neat): 3062, 2992, 2939, 2238, 1587, 1469, 1431, 1075, 933, 746 cm^{-1}. The spectra data matched those reported in the literature.[24]

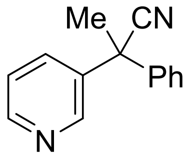

2-Phenyl-2-(pyridin-3-yl)propanenitrile (3ra). According to the general procedure analogous to that described for **3aa**, except that the crude material was purified by column chromatography on silica gel (hexane:AcOEt =5:1), **3ra** (40.7 mg, 0.195 mmol, 93%) was obtained from **1r** (20.3 mg, 0.209 mmol) as a colorless oil. ^{1}H NMR (400 MHz, $CDCl_3$/TMS) δ 8.66 (s, 1H), 8.59 (d, 1H, *J* = 4.3 Hz), 7.68 (dt, 1H, *J* = 8.0, 1.8 Hz), 7.43-7.27 (m, 6H), 2.13 (s, 3H). ^{13}C NMR (100 MHz, $CDCl_3$) δ 149.2, 147.8, 139.8, 137.0, 134.2, 129.0, 128.2, 126.4, 123.4, 122.3, 44.4, 27.7. LRMS (EI) *m/z*: 208 (M^+). HRMS Calcd. for $C_{14}H_{12}N_2$: 208.1000, found: 208.0996. IR (neat): 3030, 2989, 2237, 1576, 1496, 1419, 1075, 1021, 806, 760 cm^{-1}.

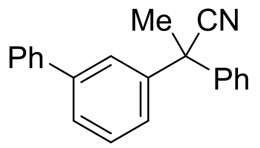

2-([1,1'-Biphenyl]-3-yl)-2-phenylpropanenitrile (3sa). According to the general procedure analogous to that described for **3aa**, except that the reaction was conducted in toluene at 120 °C, **3sa** (50.6 mg, 0.179 mmol, 90%) was obtained from **1s** (34.2 mg, 0.199 mmol) as a colorless oil. ^{1}H NMR (400 MHz, DMSO-d_6) δ 7.65-7.57 (m, 4H), 7.54-7.32 (m, 10H), 2.17 (s, 3H). ^{13}C NMR (100 MHz, DMSO-d_6) δ 141.9, 141.03, 140.95, 139.6, 129.6, 129.01, 128.99, 127.9, 127.8, 126.9, 126.4, 126.3, 125.5, 124.4, 123.4, 46.0, 27.0. LRMS (EI) *m/z*: 283 (M$^+$). HRMS Calcd. for $C_{21}H_{17}N$: 283.1361, found: 283.1350. IR (neat): 3064, 2988, 2944, 2237, 1598, 1479, 1414, 1190, 1077, 757 cm^{-1}.

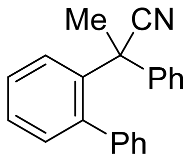

2-([1,1'-Biphenyl]-2-yl)-2-phenylpropanenitrile (3ta). According to the general procedure analogous to that described for **3aa**, except that the reaction was conducted in toluene at 140 °C and the crude material was purified by preparative thin-layer chromatography of silica gel (hexane:AcOEt = 10:1), **3ta** (42.1 mg, 0.149 mmol, 77%) was obtained from **1t** (33.1 mg, 0.192 mmol) as a white solid: Mp 86 °C (hexane). ^{1}H NMR (400 MHz, DMSO-d_6) δ 7.80 (d, 1H, J = 7.8 Hz), 7.55 (t, 1H, J = 7.8 Hz), 7.45 (t, 1H, J = 7.6 Hz), 7.30-6.30 (m, 11H), 1.97 (s, 3H). ^{13}C NMR (100 MHz, acetone-d_6) δ 143.9, 143.8, 141.4, 138.5, 133.7, 130.6, 129.4, 129.0, 128.7, 128.0, 127.9, 127.8, 127.6, 126.8, 123.5, 46.3, 30.9. LRMS (EI) *m/z*: 285 (M$^+$). HRMS Calcd. for $C_{15}H_{12}BrN$: 285.0153, found: 285.0156. IR (neat): 3063, 2984, 2238, 1601, 1495, 1441, 1378, 1073, 1011, 762 cm^{-1}.

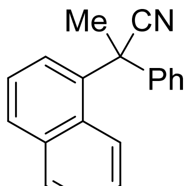

2-(Naphthalen-1-yl)-2-phenylpropanenitrile (3ua). According to the general procedure analogous to that described for **3aa**, **3ua** (46.8 mg, 0.182 mmol, 93%) was obtained from **1u** (28.7 mg, 0.196 mmol) as a white solid: Mp 148 °C (hexane/CH_2Cl_2) (lit. 143 °C[25]). ^{1}H NMR (400 MHz, $CDCl_3$/TMS) δ 7.92 (d, 1H, J = 8.3 Hz), 7.87 (d, 1H, J = 7.8Hz), 7.80-7.74 (m, 2H), 7.56 (t, 1H, J = 7.8 Hz), 7.42 (t, 1H, J = 7.6 Hz), 7.38-7.22 (m, 6H), 2.24 (s, 3H). ^{13}C NMR (150 MHz, CD_3CN) δ 143.9, 135.7, 135.3, 131.0, 130.9, 130.23, 130.15, 128.7, 127.0, 126.8, 126.50, 126.48, 126.3, 126.2, 124.1, 46.0, 31.0. LRMS (EI) *m/z*: 257 (M$^+$). HRMS Calcd. for $C_{19}H_{15}N$: 257.1204, found: 257.1199. IR (neat):

3086, 2993, 2238, 1599, 1511, 1492, 1025, 912, 801, 782 cm^{-1}.

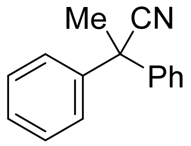

2,2-Diphenylpropanenitrile (3va). According to the general procedure analogous to that described for **3aa**, except that the reaction was conducted at 120 °C, **3va** (45.4 mg, 0.219 mmol, 97%) was obtained from **1v** (21.6 mg, 0.225 mmol) as a colorless oil. ^{1}H NMR (400 MHz, $CDCl_3$/TMS) δ 7.40-7.28 (m, 10H), 2.09 (s, 3H). ^{13}C NMR (100 MHz, $CDCl_3$) δ 141.2, 128.8, 127.9, 126.6, 123.4, 46.1, 28.1. LRMS (EI) *m/z*: 207 (M^+). HRMS Calcd. for $C_{15}H_{13}N$: 207.1048, found: 207.1054. IR (neat): 3066, 2989, 2237, 1602, 1492, 1447, 1206, 1069, 1027, 754 cm^{-1}. The spectra data matched those reported in the literature.[26]

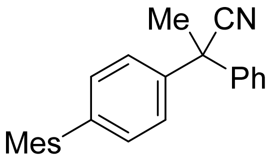

2-Phenyl-2-(2',4',6'-trimethyl-[1,1'-biphenyl]-4-yl)propanenitrile (3wa). According to the general procedure analogous to that described for **3aa**, except that the reaction was conducted at 120 °C, **3wa** (63.2 mg, 0.194 mmol, 97%) was obtained from **1w** (42.8 mg, 0.200 mmol) as a white solid: Mp 147 °C (hexane/CH_2Cl_2). ^{1}H NMR (400 MHz, $CDCl_3$/TMS) δ 7.46-7.30 (m, 7H), 7.14 (d, 2H, J = 8.3 Hz), 6.93 (s, 2H), 2.32 (s, 3H), 2.14 (s, 3H), 1.98 (s, 6H). ^{13}C NMR (150 MHz, $CDCl_3$, 59 ℃) δ 141.5, 141.0, 139.5, 138.1, 136.7, 135.8, 129.9, 128.9, 128.1, 127.9, 126.7, 126.6, 123.5, 46.1, 28.3, 20.9, 20.6. LRMS (EI) *m/z*: 325 (M^+). HRMS Calcd. for $C_{24}H_{23}N$: 325.1830, found: 325.1828. IR (neat): 2924, 2850, 2238, 1608, 1480, 1447, 1072, 1029, 846, 763 cm^{-1}.

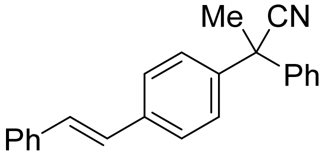

(*E*)-2-Phenyl-2-(4-styrylphenyl)propanenitrile (3xa). According to the general procedure analogous to that described for **3aa**, except that the crude material was purified by column chromatography on silica gel (hexane:AcOEt =10:1 and toluene), **3xa** (52.1 mg, 0.168 mmol, 85%) was obtained from **1x** (39.3 mg, 0.198 mmol) as a white solid: Mp 93 °C (hexane/CH_2Cl_2). ^{1}H NMR (400 MHz, $CDCl_3$/TMS) δ 7.53-7.47 (m, 4H), 7.42-7.26 (m, 10H), 7.12 (d, 1H, J = 16.1 Hz), 7.07 (d, 1H, J = 16.6 Hz), 2.10 (s, 3H). ^{13}C NMR (100 MHz, DMSO-d_6) δ 141.1, 140.2, 136.81, 136.76, 129.3, 129.0, 128.7, 127.9, 127.8, 127.4, 127.0, 126.7, 126.6, 126.2, 123.3, 45.6, 27.0. LRMS (EI) *m/z*: 309 (M^+). HRMS Calcd. for

$C_{23}H_{19}N$: 309.1517, found: 309.1503. IR (neat): 3026, 2239, 1598, 1494, 1449, 1188, 1072, 967, 820, 754 cm^{-1}.

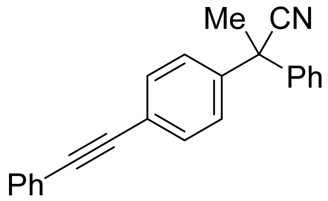

2-Phenyl-2-(4-(phenylethynyl)phenyl)propanenitrile (3ya). According to the general procedure analogous to that described for **3aa**, except that the reaction was conducted at 60 °C and the crude material was purified by column chromatography on silica gel (hexane:AcOEt =20:1), **3ya** (52.7 mg, 0.171 mmol, 87%) was obtained from **1y** (38.8 mg, 0.198 mmol) as a white solid: Mp 138 °C (hexane/CH_2Cl_2). ^{1}H NMR (400 MHz, $CDCl_3$/TMS) δ 7.56-7.49 (m, 4H), 7.40-7.28 (m, 10H), 2.10 (s, 3H). ^{13}C NMR (100 MHz, $CDCl_3$) δ 141.1, 140.8, 132.0, 131.6, 128.9, 128.4, 128.3, 128.0, 126.6, 126.5, 123.1, 123.0, 122.9, 90.2, 88.5, 46.0, 27.9. LRMS (EI) *m/z*: 307 (M^+). HRMS Calcd. for $C_{23}H_{17}N$: 307.1361, found: 307.1336. IR (neat): 3063, 2944, 2235, 1601, 1514, 1448, 1206, 1071, 814, 759 cm^{-1}.

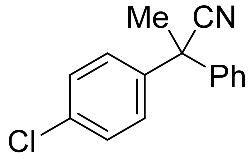

2-(4-Chlorophenyl)-2-phenylpropanenitrile (3za). According to the general procedure analogous to that described for **3aa**, except that the crude material was purified by preparative thin-layer chromatography of silica gel (hexane:AcOEt = 10:1), **3za** (35.8 mg, 0.148 mmol, 75%) was obtained from **1z** (25.7 mg, 0.197 mmol) as a colorless oil. ^{1}H NMR (400 MHz, $CDCl_3$/TMS) δ 7.41-7.28 (m, 9H), 2.07 (s, 3H). ^{13}C NMR (100 MHz, $CDCl_3$) δ 140.7, 139.9, 134.0, 129.02, 128.99, 128.13, 128.05, 126.5, 123.0, 45.7, 28.1. LRMS (EI) *m/z*: 241 (M^+). HRMS Calcd. for $C_{15}H_{12}ClN$: 241.0658, found: 241.0681. IR (neat): 3068, 2989, 2238, 1600, 1492, 1403, 1096, 1014, 816, 759 cm^{-1}.

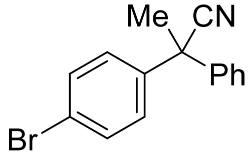

2-(4-Bromophenyl)-2-phenylpropanenitrile (3Aa). According to the general procedure analogous to that described for **3aa**, except that the crude material was purified by preparative thin-layer chromatography of silica gel (hexane:AcOEt = 9:1), **3Aa** (41.2 mg, 0.144 mmol, 66%) was obtained from **1A** (38.2 mg, 0.218 mmol) as a colorless oil. ^{1}H NMR (400 MHz, $CDCl_3$/TMS) δ 7.49 (d, 2H, *J*

= 8.8 Hz), 7.41-7.30 (m, 5H), 7.28-7.23 (m, 2H), 2.07 (s, 3H). ^{13}C NMR (100 MHz, $CDCl_3$) δ 140.6, 140.4, 132.0, 129.0, 128.4, 128.1, 126.5, 122.9, 122.1, 45.8, 28.0. LRMS (EI) *m/z*: 285 (M^+). HRMS Calcd. for $C_{15}H_{12}BrN$: 285.0153, found: 285.0156. IR (neat): 3067, 2988, 2238, 1600, 1490, 1397, 1079, 1010, 813, 758 cm^{-1}. The spectra data matched those reported in the literature.[26]

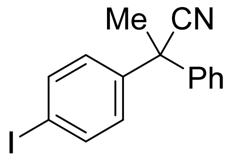

2-(4-Iodophenyl)-2-phenylpropanenitrile (3Ba). According to the general procedure analogous to that described for **3aa**, except that the crude material was purified by preparative thin-layer chromatography of silica gel (hexane:AcOEt = 9:1), **3Ba** (41.6 mg, 0.125 mmol, 60%) was obtained from **1B** (45.9 mg, 0.207 mmol) as a colorless oil. ^{1}H NMR (400 MHz, $CDCl_3$/TMS) δ 7.69 (d, 2H, *J* = 8.3 Hz), 7.42-7.30 (m, 5H), 7.12 (d, 2H, *J* = 8.3 Hz), 2.06 (s, 3H). ^{13}C NMR (100 MHz, $CDCl_3$) δ 141.1, 140.5, 137.9, 128.9, 128.5, 128.1, 126.5, 122.8, 93.7, 45.8, 27.8. LRMS (EI) *m/z*: 333 (M^+). HRMS Calcd. for $C_{15}H_{12}IN$: 333.0014, found: 332.9994. IR (neat): 3061, 2988, 2238, 1599, 1486, 1395, 1072, 1005, 809, 757 cm^{-1}.

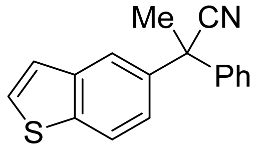

2-(Benzo[*b*]thiophen-5-yl)-2-phenylpropanenitrile (3Ca). According to the general procedure analogous to that described for **3aa**, **3Ca** (38.7 mg, 0.147 mmol, 75%) was obtained from **1C** (29.9 mg, 0.196 mmol) as a colorless oil. ^{1}H NMR (400 MHz, $CDCl_3$/TMS) δ 7.91 (d, 1H, *J* = 2.0 Hz), 7.84 (d, 1H, *J* = 8.8 Hz), 7.50 (d, 1H, *J* = 5.4 Hz), 7.44-7.26 (m, 7H), 2.16 (s, 3H). ^{13}C NMR (100 MHz, $CDCl_3$) δ 141.4, 139.7, 139.2, 137.6, 128.9, 127.9, 127.7, 126.6, 123.9, 123.5, 123.1, 123.0, 121.4, 46.1, 28.3. LRMS (EI) *m/z*: 263 (M^+). HRMS Calcd. for $C_{17}H_{13}NS$: 263.0769, found: 263.0759. IR (neat): 3061, 2986, 2238, 1600, 1493, 1441, 1160, 1073, 809, 752 cm^{-1}.

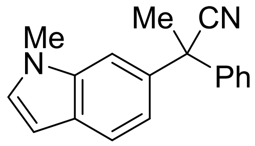

2-(1-Methyl-1*H*-indol-6-yl)-2-phenylpropanenitrile (3Da). According to the general procedure analogous to that described for **3aa**, except that the reaction was conducted in mesitylene at 160 °C and the crude material was purified by column chromatography on silica gel (hexane: AcOEt = 10:1), **3Da** (41.4 mg, 0.159 mmol, 76%) was obtained from **1D** (31.0 mg, 0.208 mmol) as a orange solid: Mp

82.5-83 °C. ^{1}H NMR (600 MHz, $CDCl_3$/TMS) δ 7.57 (d, 1H, J = 8.5 Hz), 7.43-7.39 (m, 3H), 7.34 (t, 2H, J = 7.5 Hz), 7.28 (t, 1H, J = 7.3 Hz), 7.08 (d, 1H, J = 3.0 Hz), 7.00 (d, 1H, J = 8.0 Hz), 6.46 (d, 1H, J = 3.0 Hz), 3.78 (s, 3H), 2.16 (s, 3H). ^{13}C NMR (150 MHz, $CDCl_3$) δ 142.1, 136.4, 134.6, 130.0, 128.7, 127.8, 127.7, 126.7, 124.0, 121.3, 118.2, 107.4, 100.8, 46.4, 32.9, 28.6. LRMS (EI) *m/z*: 260 (M^+). HRMS Calcd. for $C_{18}H_{16}N_2$: 260.1313, found: 260.1322. IR (neat): 3058, 2984, 2940, 2235, 1718, 1623, 1445, 1340, 1248, 1196, 1078, 1025, 929, 755 cm^{-1}.

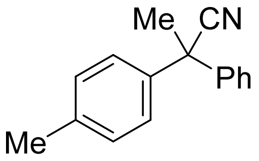

2-Phenyl-2-(*p*-tolyl)propanenitrile (3Ea). According to the general procedure analogous to that described for **3aa**, except that the reaction was conducted at 140 °C and the crude material was purified by preparative thin-layer chromatography of silica gel (toluene:AcOEt = 10:1), **3Ea** (32.5 mg, 0.147 mmol, 74%) was obtained from **1E** (21.9 mg, 0.199 mmol) as a colorless oil. ^{1}H NMR (400 MHz, $CDCl_3$/TMS) δ 7.40-7.20 (m, 7H), 7.15 (d, 2H, J = 8.6 Hz), 2.34 (s, 3H), 2.07 (s, 3H). ^{13}C NMR (100 MHz, $CDCl_3$) δ 141.5, 138.3, 137.7, 129.5, 128.8, 127.8, 126.52, 126.48, 123.5, 45.8, 28.1, 20.9. LRMS (EI) *m/z*: 221 (M^+). HRMS Calcd. for $C_{16}H_{15}N$: 221.1204, found: 221.1211. IR (neat): 3033, 2991, 2236, 1600, 1512, 1448, 1190, 1019, 809, 754 cm^{-1}. The spectra data matched those reported in the literature.[27]

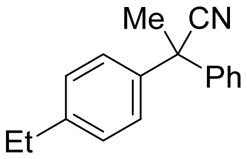

2-(4-Ethylphenyl)-2-phenylpropanenitrile (3Fa). According to the general procedure analogous to that described for **3aa**, except that the reaction was conducted at 140 °C and the crude material was purified by preparative thin-layer chromatography of silica gel (hexane:AcOEt = 10:1), **3Fa** (36.7 mg, 0.156 mmol, 79%) was obtained from **1F** (24.5 mg, 0.197 mmol) as a colorless oil. ^{1}H NMR (400 MHz, DMSO-d_6) δ 7.42-7.27 (m, 7H), 7.24 (d, 2H, J = 8.3 Hz), 2.58 (q, 2H, J = 7.6 Hz), 2.06 (s, 3H), 1.15 (t, 3H, J = 7.8 Hz). ^{13}C NMR (100 MHz, DMSO-d_6) δ 143.5, 141.3, 138.4, 128.9, 128.3, 127.8, 126.17, 126.15, 123.5, 45.5, 27.5, 27.1, 15.2. LRMS (EI) *m/z*: 235 (M^+). HRMS Calcd. for $C_{17}H_{17}N$: 235.1361, found: 235.1354. IR (neat): 3061, 3032, 2967, 2938, 2362, 2337, 2238, 1495, 1414, 1089, 860, 823, 754 cm^{-1}.

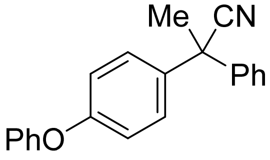

2-(4-Phenoxyphenyl)-2-phenylpropanenitrile (3Ga). According to the general procedure analogous to that described for **3aa**, except that the reaction was conducted at 60 °C and the crude material was purified by column chromatography on silica gel (hexane:AcOEt =20:1), **3Ga** (53.0 mg, 0.177 mmol, 86%) was obtained from **1G** (38.7 mg, 0.206 mmol) as a colorless oil. ^{1}H NMR (400 MHz, $CDCl_3$/TMS) δ7.42-7.29 (m, 9H), 7.13 (t, 1H, J = 7.6 Hz), 7.02 (d, 2H, J = 7.8 Hz), 6.97 (d, 2H, J = 8.8 Hz), 2.08 (s, 3H). ^{13}C NMR (100 MHz, $CDCl_3$) δ157.1, 156.5, 141.3, 135.7, 129.8, 128.9, 128.1, 127.9, 126.5, 123.8, 123.4, 119.3, 118.6, 45.6, 28.3. LRMS (EI) *m/z*: 299 (M^+). HRMS Calcd. for $C_{21}H_{17}NO$: 299.1310, found: 299.1302. IR (neat): 3066, 2988, 2236, 1587, 1506, 1489, 1240, 1174, 872, 749 cm^{-1}.

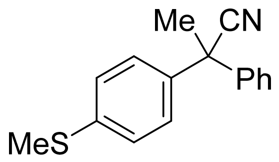

2-(4-(Methylthio)phenyl)-2-phenylpropanenitrile (3Ha). According to the general procedure analogous to that described for **3aa**, **3Ha** (44.6 mg, 0.176 mmol, 87%) was obtained from **1H** (28.7 mg, 0.202 mmol) as a colorless oil. ^{1}H NMR (400 MHz, $CDCl_3$/TMS) δ7.38-7.26 (m, 7H), 7.23 (d, 2H, J = 8.3 Hz), 2.48 (s, 3H), 2.07 (s, 3H). ^{13}C NMR (100 MHz, $CDCl_3$) δ141.1, 138.6, 137.8, 128.8, 127.9, 127.0, 126.50, 126.49, 123.2, 45.7, 28.0, 15.5. LRMS (EI) *m/z*: 253 (M^+). HRMS Calcd. for $C_{16}H_{15}NS$: 253.0925, found: 253.0904. IR (neat): 2987, 2917, 2236, 1599, 1494, 1447, 1099, 1014, 813, 760 cm^{-1}.

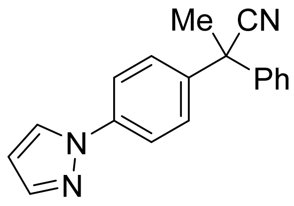

2-(4-(1*H*-pyrazol-1-yl)phenyl)-2-phenylpropanenitrile (3Ia). According to the general procedure analogous to that described for **3aa**, except that the crude material was purified by column chromatography on silica gel (hexane:AcOEt = 5:1), **3Ia** (46.1 mg, 0.169 mmol, 83%) was obtained from **1I** (32.8 mg, 0.202 mmol) as a yellow oil. ^{1}H NMR (400 MHz, $CDCl_3$/TMS) δ7.92 (d, 1H, J = 2.4 Hz), 7.75-7.68 (m, 3H), 7.47 (d, 2H, J = 8.8 Hz), 7.43-7.30 (m, 5H), 6.48 (t, 1H, J = 2.0 Hz), 2.12 (s, 3H). ^{13}C NMR (150 MHz, $CDCl_3$, 40 °C) δ141.2, 140.9, 139.6, 139.2, 128.9, 128.0, 127.7, 126.6, 126.4, 123.0, 119.2, 107.8, 45.7, 28.0. LRMS (EI) *m/z*: 273 (M^+). HRMS Calcd. for $C_{18}H_{15}N_3$: 273.1266, found: 273.1255. IR (neat): 3145, 2988, 2238, 1611, 1525, 1394, 1199, 1031, 936, 758 cm^{-1}.

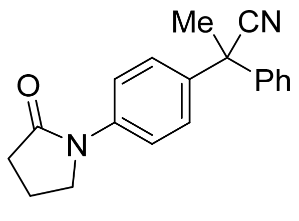

2-(4-(2-Oxopyrrolidin-1-yl)phenyl)-2-phenylpropanenitrile (3Ja). According to the general procedure analogous to that described for **3aa**, except that the reaction was conducted at 120 °C and the crude material was purified by column chromatography on silica gel (hexane:AcOEt =1:1), **3Ja** (49.0 mg, 0.169 mmol, 81%) was obtained from **1J** (37.2 mg, 0.208 mmol) as a white solid: Mp 129 °C (hexane/CH_2Cl_2). ^{1}H NMR (400 MHz, $CDCl_3$/TMS) δ 7.62 (dt, 2H, J = 8.8, 2.4 Hz), 7.40-7.26 (m, 7H), 3.85 (t, 2H, J = 7.1 Hz), 2.61 (t, 2H, J = 8.0 Hz), 2.17 (quint, 2H, J = 7.6 Hz), 2.08 (s, 3H). ^{13}C NMR (150 MHz, $CDCl_3$, 40 °C) δ 174.2, 141.2, 139.1, 137.0, 128.8, 127.9, 127.1, 126.5, 123.2, 119.9, 48.5, 45.7, 32.6, 28.1, 17.9. LRMS (EI) *m/z*: 290 (M^+). HRMS Calcd. for $C_{19}H_{18}N_2O$: 290.1419, found: 290.1391. IR (neat): 2977, 2888, 2241, 1684, 1515, 1397, 1306, 1228, 848, 764 cm^{-1}.

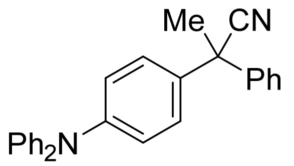

2-(4-(Diphenylamino)phenyl)-2-phenylpropanenitrile (3Ka). According to the general procedure analogous to that described for **3aa**, except that the reaction was conducted at 120 °C, **3Ka** (69.7 mg, 0.186 mmol, 93%) was obtained from **1K** (52.8 mg, 0.201 mmol) as a colorless oil. ^{1}H NMR (400 MHz, $CDCl_3$/TMS) δ 7.44-7.35 (m, 4H), 7.33-7.22 (m, 5H), 7.19 (d, 2H, J = 7.3 Hz), 7.10-6.98 (m, 8H), 2.07 (s, 3H). ^{13}C NMR (100 MHz, $CDCl_3$) δ 147.4, 147.3, 141.4, 134.3, 129.3, 128.8, 127.8, 127.4, 126.5, 124.7, 123.5, 123.3, 122.8, 45.6, 28.2. LRMS (EI) *m/z*: 374 (M^+). HRMS Calcd. for $C_{27}H_{22}N_2$: 374.1783, found: 374.1792. IR (neat): 3066, 3034, 2944, 2235, 1588, 1507, 1487, 1327, 1274, 753 cm^{-1}.

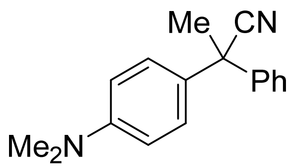

2-(4-(Dimethylamino)phenyl)-2-phenylpropanenitrile (3La). According to the general procedure analogous to that described for **3aa**, except that the reaction was conducted in mesitylene at 180 °C and the crude material was purified by preparative thin-layer chromatography of silica gel (toluene), **3La** (33.1 mg, 0.132 mmol, 65%) was obtained from **1L** (28.4 mg, 0.204 mmol) as a brown oil. ^{1}H NMR (400 MHz, $CDCl_3$/TMS) δ 7.41-7.26 (m, 5H), 7.21 (d, 2H, J = 8.8 Hz), 6.68 (d, 2H, J = 8.8 Hz), 2.95 (s, 6H), 2.04 (s, 3H). ^{13}C NMR (100 MHz, $CDCl_3$) δ 149.9, 142.2, 128.7, 128.5, 127.6, 127.4,

126.5, 123.9, 112.3, 45.3, 40.4, 28.3. LRMS (EI) *m/z*: 250 (M^+). HRMS Calcd. for $C_{17}H_{18}N_2$: 250.1470, found: 250.1477. IR (neat): 3063, 2886, 2234, 1612, 1520, 1356, 1169, 947, 812, 764 cm^{-1}.

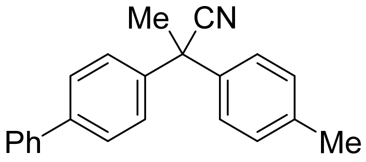

2-([1,1'-Biphenyl]-4-yl)-2-(*p*-tolyl)propanenitrile (3ab). According to the general procedure analogous to that described for **3aa**, **3ab** (52.0 mg, 0.175 mmol, 87%) was obtained from **1a** (34.5 mg, 0.200 mmol) as a colorless oil. ^1H NMR (400 MHz, $CDCl_3$/TMS) δ 7.60-7.54 (m, 4H), 7.47-7.41 (m, 4H), 7.36 (t, 1H, J = 7.3 Hz), 7.31 (d, 2H, J = 8.3 Hz), 7.19 (d, 2H, J = 8.3 Hz), 2.35 (s, 3H), 2.11 (s, 3H). ^{13}C NMR (150 MHz, $CDCl_3$) δ 140.7, 140.5, 140.1, 138.2, 137.8, 129.5, 128.8, 127.55, 127.47, 127.03, 126.97, 126.5, 123.5, 45.6, 28.1, 20.9. LRMS (EI) *m/z*: 297 (M^+). HRMS Calcd. for $C_{22}H_{19}N$: 297.1517, found: 297.1523. IR (neat): 3031, 2919, 2235, 1600, 1511, 1486, 1072, 1008, 764, 731 cm^{-1}.

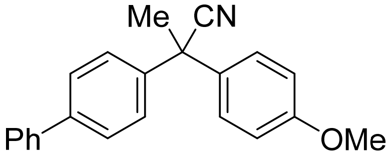

2-([1,1'-Biphenyl]-4-yl)-2-(4-methoxyphenyl)propanenitrile (3ac). According to the general procedure analogous to that described for **3aa**, except that the crude material was purified by preparative thin-layer chromatography of silica gel (toluene:AcOEt = 30:1), **3ac** (49.7 mg, 0.159 mmol, 80%) was obtained from **1a** (34.0 mg, 0.197 mmol) as a white solid: Mp 91 °C (hexane/CH_2Cl_2). ^1H NMR (400 MHz, $CDCl_3$/TMS) δ 7.60-7.53 (m, 4H), 7.47-7.40 (m, 4H), 7.38-7.30 (m, 3H), 6.90 (d, 2H, J = 8.8 Hz), 3.81 (s, 3H), 2.10 (s, 3H). ^{13}C NMR (150 MHz, $CDCl_3$) δ 159.1, 140.64, 140.60, 140.1, 133.1, 128.8, 127.8, 127.5, 127.4, 127.0, 126.9, 123.5, 114.1, 55.2, 45.2, 28.2. LRMS (EI) *m/z*: 313 (M^+). HRMS Calcd. for $C_{22}H_{19}NO$: 313.1467, found: 313.1461. IR (neat): 2960, 2836, 2244, 1516, 1250, 1190, 1029, 839, 771, 701 cm^{-1}.

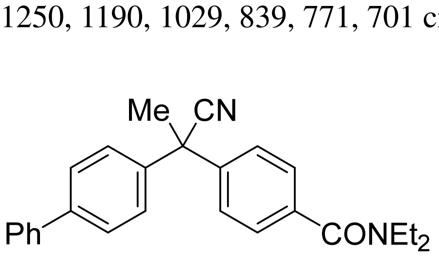

4-(1-([1,1'-Biphenyl]-4-yl)-1-cyanoethyl)-*N*,*N*-diethylbenzamide (3ad). According to the general procedure analogous to that described for **3aa**, except that the reaction was conducted at 120 °C and the crude material was purified by preparative thin-layer chromatography of silica gel (toluene:AcOEt = 3:1), **3ad** (56.6 mg, 0.148 mmol, 75%) was obtained from **1a** (34.2 mg, 0.199 mmol) as a colorless

oil. ^{1}H NMR (400 MHz, $CDCl_3$/TMS) δ 7.62-7.55 (m, 4H), 7.48-7.34 (m, 9H), 3.54 (brs, 2H), 3.27 (brs, 2H), 2.13 (s, 2H), 1.25 (brs, 3H), 1.13 (brs, 3H). ^{13}C NMR (100 MHz, $CDCl_3$) δ 170.4, 142.1, 141.0, 139.95, 139.62, 136.9, 128.8, 127.6, 127.5, 127.02, 127.01, 126.9, 126.8, 123.1, 45.8, 43.2, 39.2, 28.0, 14.2, 12.8. LRMS (EI) *m/z*: 382 (M^+). HRMS Calcd. for $C_{26}H_{26}N_2O$: 382.2045, found: 382.2022. IR (neat): 2986, 2943, 2236, 1627, 1487, 1428, 1289, 1098, 837, 764 cm^{-1}.

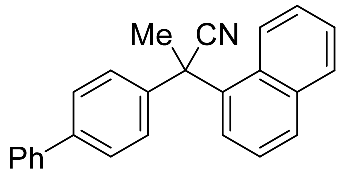

2-([1,1'-Biphenyl]-4-yl)-2-(naphthalen-1-yl)propanenitrile (3ae). According to the general procedure analogous to that described for **3aa**, except that the reaction was conducted at 120 °C, **3ae** (63.3 mg, 0.190 mmol, 94%) was obtained from **1e** (34.8 mg, 0.202 mmol) as a white solid: Mp 117 °C (hexane). ^{1}H NMR (400 MHz, $CDCl_3$/TMS) δ 7.94 (d, 1H, *J* = 8.3 Hz), 7.88 (d, 1H, *J* = 7.8 Hz), 7.83 (d, 1H, *J* = 8.8 Hz), 7.80 (d, 1H, *J* = 7.3 Hz), 7.58 (d, 1H, *J* = 7.8 Hz), 7.56-7.51 (m, 4H), 7.47-7.26 (m, 7H), 2.28 (s, 3H). ^{13}C NMR (150 MHz, $CDCl_3$) δ 141.6, 140.4, 140.0, 134.7, 134.2, 130.3, 130.1, 129.0, 128.8, 127.7, 127.5, 126.9, 126.3, 126.1, 125.8, 125.6, 124.9, 124.8, 123.1, 44.8, 30.6. LRMS (EI) *m/z*: 333 (M^+). HRMS Calcd. for $C_{25}H_{19}N$: 333.1517, found: 333.1512. IR (neat): 3025, 2929, 2235, 1600, 1485, 1401, 1008, 838, 776, 764 cm^{-1}.

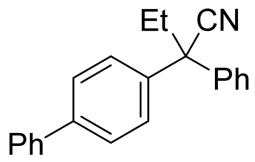

2-([1,1'-Biphenyl]-4-yl)-2-phenylbutanenitrile (3af). According to the general procedure analogous to that described for **3aa**, except that the reaction was conducted at 120 °C, **3af** (52.0 mg, 0.175 mmol, 86%) was obtained from **1a** (35.0 mg, 0.203 mmol) as a colorless oil. ^{1}H NMR (400 MHz, $CDCl_3$/TMS) δ 7.60-7.55 (m, 4H), 7.48-7.26 (m, 10H), 2.47 (q, 2H, *J* = 7.5 Hz), 1.10 (t, 3H, *J* = 7.6 Hz). ^{13}C NMR (100 MHz, $CDCl_3$) δ 140.6, 140.02, 139.97, 139.1, 128.79, 128.76, 127.8, 127.5, 127.4, 127.3, 126.95, 126.87, 122.1, 52.3, 32.7, 10.1. LRMS (EI) *m/z*: 297 (M^+). HRMS Calcd. for $C_{22}H_{19}N$: 297.1517, found: 297.1501. IR (neat): 3031, 2982, 2882, 2235, 1599, 1486, 1385, 1008, 833, 760 cm^{-1}.

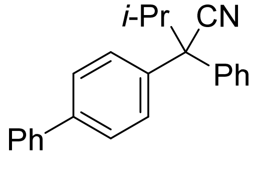

2-([1,1'-Biphenyl]-4-yl)-3-methyl-2-phenylbutanenitrile (3ag). According to the general procedure analogous to that described for **3aa**, except that the reaction was conducted at 120 °C, **3ag** (46.6 mg, 0.150 mmol, 75%) was obtained from **1a** (34.2 mg, 0.199 mmol) as a colorless oil. ^{1}H NMR (400 MHz, $CDCl_3$/TMS) δ 7.58-7.50 (m, 8H), 7.42 (t, 2H, J = 7.6 Hz), 7.38-7.30 (m, 3H), 7.28-7.22 (m, 1H), 2.94 (sept, 1H, J = 6.6 Hz), 1.12 (d, 3H, J = 6.4 Hz), 1.09 (d, 3H, J = 6.8 Hz). ^{13}C NMR (150 MHz, $CDCl_3$) δ 140.4, 140.1, 139.7, 138.8, 128.9, 128.8, 127.6, 127.5, 127.4, 127.1, 127.0, 126.6, 120.7, 58.9, 34.8, 19.1, 19.0. LRMS (EI) *m/z*: 311 (M^+). HRMS Calcd. for $C_{23}H_{21}N$: 311.1674, found: 311.1659. IR (neat): 3041, 2978, 2877, 2238, 1599, 1487, 1391, 1171, 1008, 760 cm^{-1}. The spectra data matched those reported in the literature.[28]

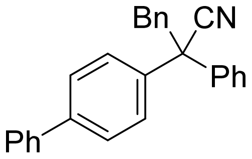

2-([1,1'-Biphenyl]-4-yl)-2,3-diphenylpropanenitrile (3ah). According to the general procedure analogous to that described for **3aa**, except that the reaction was conducted at 120 °C, **3ah** (45.3 mg, 0.126 mmol, 64%) was obtained from **1a** (34.1 mg, 0.198 mmol) as a white solid: Mp 147 °C (hexane/CH_2Cl_2). ^{1}H NMR (400 MHz, $CDCl_3$/TMS) δ 7.60-7.54 (m, 4H), 7.47-7.28 (m, 10H), 7.23-7.12 (m, 3H), 6.92 (dd, 2H, J = 8.1, 1.3 Hz), 3.71 (d, 1H, J = 13.1 Hz), 3.68 (d, 1H, J = 13.3 Hz). ^{13}C NMR (150 MHz, $CDCl_3$) δ 140.8, 140.0, 139.9, 139.0, 134.6, 130.5, 128.8, 128.7, 128.0, 127.9, 127.8, 127.6, 127.4, 127.29, 127.28, 127.0, 121.8, 52.7, 45.3. LRMS (EI) *m/z*: 359 (M^+). HRMS Calcd. for $C_{27}H_{21}N$: 359.1674, found: 359.1692. IR (neat): 3079, 3033, 2233, 1583, 1494, 1077, 1009, 834, 766, 730 cm^{-1}.

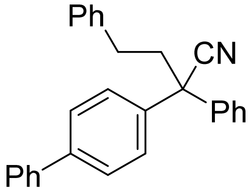

2-([1,1'-Biphenyl]-4-yl)-2,4-diphenylbutanenitrile (3ai). According to the general procedure analogous to that described for **3aa**, except that the reaction was conducted at 120 °C, **3ai** (67.4 mg, 0.180 mmol, 90%) was obtained from **1a** (34.5 mg, 0.200 mmol) as a colorless oil. ^{1}H NMR (400 MHz, $CDCl_3$/TMS) δ 7.63-7.54 (m, 4H), 7.52-7.26 (m, 12H), 7.25-7.15 (m, 3H), 2.81-2.66 (m, 4H). ^{13}C NMR (150 MHz, $CDCl_3$) δ 140.8, 140.6, 140.0, 139.9, 138.9, 129.0, 128.8, 128.6, 128.3, 128.0, 127.61, 127.58, 127.3, 127.0, 126.8, 126.3, 122.1, 51.5, 41.7, 32.1. LRMS (EI) *m/z*: 373 (M^+). HRMS Calcd. for $C_{28}H_{23}N$: 373.1830, found: 373.1820. IR (neat): 3026, 2932, 2870, 2237, 1601, 1487, 1448, 1008, 834, 761 cm^{-1}.

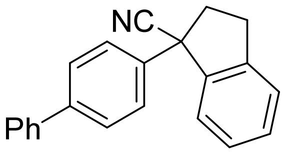

1-([1,1'-Biphenyl]-4-yl)-2,3-dihydro-1*H*-indene-1-carbonitrile (3aj). According to the general procedure analogous to that described for **3aa**, **3aj** (53.1 mg, 0.180 mmol, 89%) was obtained from **1a** (34.8 mg, 0.202 mmol) as a white solid: Mp 98 °C (hexane/CH_2Cl_2). ^{1}H NMR (400 MHz, $CDCl_3$/TMS) δ7.60-7.55 (m 4H), 7.44 (t, 2H, J = 7.3 Hz), 7.41-7.26 (m, 7H), 3.25-3.16 (m, 1H), 3.15-3.06 (m, 1H), 3.04-2.95 (m, 1H), 2.61-2.51 (m, 1H). ^{13}C NMR (150 MHz, $CDCl_3$) δ 143.5, 142.1, 140.9, 140.2, 139.1, 129.0, 128.8, 127.7, 127.55, 127.49, 127.0, 126.8, 125.12, 125.05, 122.4, 52.3, 43.6, 30.6. LRMS (EI) *m/z*: 295 (M^+). HRMS Calcd. for $C_{22}H_{17}N$: 295.1361, found: 295.1369. IR (neat): 3033, 2975, 2867, 2233, 1597, 1489, 1403, 1006, 836, 761 cm^{-1}.

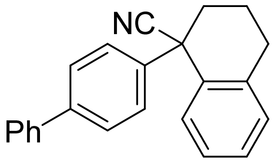

1-([1,1'-Biphenyl]-4-yl)-1,2,3,4-tetrahydronaphthalene-1-carbonitrile (3ak). According to the general procedure analogous to that described for **3aa**, except that the reaction was conducted at 120 °C, **3ak** (55.7 mg, 0.180 mmol, 90%) was obtained from **1a** (34.6 mg, 0.201 mmol) as a colorless oil. ^{1}H NMR (400 MHz, $CDCl_3$/TMS) δ7.59-7.53 (m, 4H), 7.44 (t, 2H, J = 7.8 Hz), 7.38-7.32 (m, 3H), 7.30-7.15 (m, 4H), 2.95 (t, 2H, J = 6.4 Hz), 2.58-2.48 (m, 1H), 2.30-2.21 (m, 1H), 2.10-1.96 (m, 1H), 1.94-1.82 (m, 1H). ^{13}C NMR (100 MHz, $CDCl_3$) δ141.5, 140.6, 140.2, 137.1, 134.3, 130.3, 129.6, 128.8, 128.2, 127.7, 127.5, 127.2, 127.0, 126.8, 123.5, 46.5, 39.3, 28.9, 19.4. LRMS (EI) *m/z*: 309 (M^+). HRMS Calcd. for $C_{23}H_{19}N$: 309.1517, found: 309.1490. IR (neat): 3033, 2943, 2880, 2238, 1599, 1486, 1447, 1008, 838, 760 cm^{-1}.

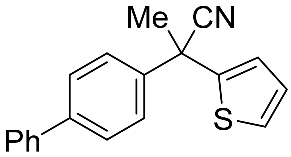

2-([1,1'-Biphenyl]-4-yl)-2-(thiophen-2-yl)propanenitrile (3al). According to the general procedure analogous to that described for **3aa**, **3al** (50.7 mg, 0.175 mmol, 89%) was obtained from **1a** (34.0 mg, 0.197 mmol) as a white solid: Mp 61 °C (hexane/CH_2Cl_2). ^{1}H NMR (400 MHz, $CDCl_3$/TMS) δ7.63-7.56 (m, 4H), 7.54 (d, 2H, J = 8.8 Hz), 7.45 (t, 2H, J = 7.6 Hz), 7.37 (t, 1H, J = 7.3 Hz), 7.31 (d, 1H, J = 4.9 Hz), 7.11 (d, 1H, J = 2.4 Hz), 7.00 (dd, 1H, J = 5.1, 3.7 Hz), 2.21 (s, 3H). ^{13}C NMR (150 MHz, $CDCl_3$) δ 145.1, 141.1, 139.9, 139.8, 128.8, 127.6, 127.5, 127.0, 126.8, 126.3, 126.1, 126.0, 122.3, 43.0, 29.6. LRMS (EI) *m/z*: 289 (M^+). HRMS Calcd. for $C_{19}H_{15}NS$: 289.0925, found: 289.0898. IR

(neat): 3112, 3005, 2235, 1582, 1488, 1404, 1241, 1006, 839, 764 cm^{-1}.

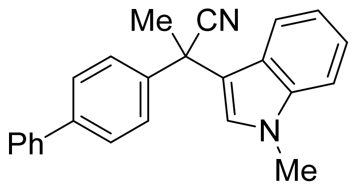

2-([1,1'-Biphenyl]-4-yl)-2-(1-methyl-1*H*-indol-3-yl)propanenitrile (3am). According to the general procedure analogous to that described for **3aa**, **3am** (61.3 mg, 0.182 mmol, 93%) was obtained from **1a** (33.8 mg, 0.196 mmol) as a white solid: Mp 151 °C (hexane/CH_2Cl_2). ^{1}H NMR (400 MHz, $CDCl_3$/TMS) δ 7.60-7.51 (m, 6H), 7.43 (t, 2H, J = 7.6 Hz), 7.37-7.30 (m, 3H), 7.22 (d, 1H, J = 8.3 Hz), 7.09 (s, 1H), 7.03 (t, 1H, J = 7.8 Hz), 3.82 (s, 3H), 2.19 (s, 3H). ^{13}C NMR (150 MHz, $CDCl_3$) δ 140.5, 140.2, 140.1, 137.7, 128.8, 127.44, 127.36, 127.0, 126.9, 126.6, 125.2, 123.0, 122.3, 120.2, 119.7, 114.5, 109.5, 40.3, 32.8, 28.5. LRMS (EI) *m/z*: 336 (M^+). HRMS Calcd. for $C_{24}H_{20}N_2$: 336.1626, found: 336.1662. IR (neat): 3033, 2941, 2232, 1546, 1483, 1335, 1224, 1093, 832, 746 cm^{-1}.

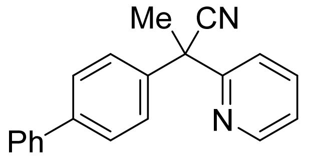

2-([1,1'-Biphenyl]-4-yl)-2-(pyridin-2-yl)propanenitrile (3an). According to the general procedure analogous to that described for **3aa**, except that the reaction was conducted at 120 °C, and the crude material was purified by preparative thin-layer chromatography of silica gel (toluene:AcOEt = 10:1), **3an** (51.5 mg, 0.181 mmol, 92%) was obtained from **1a** (33.8 mg, 0.196 mmol) as a white solid: Mp 53 °C (hexane). ^{1}H NMR (400 MHz, $CDCl_3$/TMS) δ 8.65 (d, 1H, J = 4.4 Hz), 7.70 (td, 1H, J = 7.8, 2.0 Hz), 7.60-7.47 (m, 8H), 7.44 (t, 2H, J = 7.6 Hz), 7.35 (t, 1H, J = 7.3 Hz), 2.22 (s, 3H). ^{13}C NMR (150 MHz, DMSO-d_6) δ 158.8, 149.4, 149.3, 139.9, 139.4, 139.3, 138.0, 129.0, 127.3, 126.9, 126.8, 123.4, 122.9, 121.0, 48.4, 26.0. LRMS (EI) *m/z*: 284 (M^+). HRMS Calcd. for $C_{20}H_{16}N_2$: 284.1313, found: 284.1314. IR (neat): 3033, 2953, 2246, 1587, 1485, 1467, 1078, 1007, 836, 767 cm^{-1}.

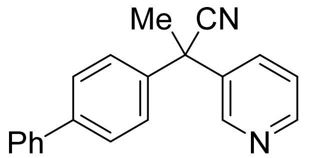

2-([1,1'-Biphenyl]-4-yl)-2-(pyridin-3-yl)propanenitrile (3ao). According to the general procedure analogous to that described for **3aa**, except that the crude material was purified by preparative thin-layer chromatography of silica gel (toluene:AcOEt = 3:1), **3ao** (50.0 mg, 0.176 mmol, 87%) was obtained from **1a** (34.9 mg, 0.203 mmol) as a colorless oil. ^{1}H NMR (400 MHz, $CDCl_3$/TMS) δ 8.71 (d, 1H, J = 2.0 Hz), 8.60 (d, 1H, J = 4.9 Hz), 7.74 (d, 1H, J = 8.3 Hz), 7.61 (d, 2H, J = 8.3 Hz), 7.57

(d, 2H, J = 7.3 Hz), 7.48-7.42 (m, 4H), 7.38 (d, 1H, J = 6.8 Hz), 7.33 (dd, 1H, J = 8.0, 4.6 Hz), 2.17 (s, 3H). ^{13}C NMR (150 MHz, $CDCl_3$) δ 149.3, 147.9, 141.3, 139.8, 138.8, 137.0, 134.3, 128.8, 127.8, 127.7, 127.0, 126.9, 123.5, 122.3, 44.3, 27.8. LRMS (EI) *m/z*: 284 (M^+). HRMS Calcd. for $C_{20}H_{16}N_2$: 284.1313, found: 284.1315. IR (neat): 3032, 2990, 2237, 1575, 1486, 1419, 1077, 1021, 828, 765 cm^{-1}.

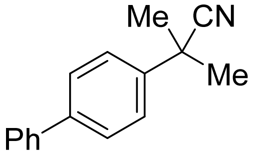

2-([1,1'-Biphenyl]-4-yl)-2-methylpropanenitrile (3ap). According to the general procedure analogous to that described for **3aa**, except that the reaction was conducted with **2p** (27.6 mg, 0.399 mmol) at 140 °C and the crude material was purified by column chromatography on silica gel (hexane:AcOEt =10:1), **3ap** (30.5mg, 0.138 mmol, 69%) was obtained from **1a** (34.6 mg, 0.201 mmol) as a white solid: Mp 85 °C (hexane/CH_2Cl_2). ^{1}H NMR (400 MHz, $CDCl_3$/TMS) δ 7.66-7.52 (m, 6H), 7.43 (t, 2H, J = 7.6 Hz), 7.37 (t, 1H, J = 7.1 Hz), 1.77 (s, 6H). ^{13}C NMR (100 MHz, $CDCl_3$) δ 140.8, 140.4, 140.2, 128.8, 127.6, 127.5, 127.0, 125.5, 124.4, 36.9, 29.1. LRMS (EI) *m/z*: 221 (M^+). HRMS Calcd. for $C_{16}H_{15}N$: 221.1204, found: 221.1201. IR (neat): 2982, 2888, 2233, 1600, 1485, 1407, 1269, 1006, 840, 766 cm^{-1}. The spectra data matched those reported in the literature.[29]

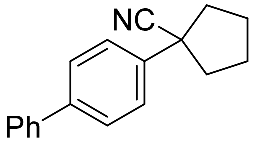

1-([1,1'-Biphenyl]-4-yl)cyclopentane-1-carbonitrile (3aq). According to the general procedure analogous to that described for **3aq**, except that the reaction was conducted with **2q** (39.8 mg, 0.418 mmol) at 120 °C, **3aq** (31.9 mg, 0.129 mmol, 65%) was obtained from **1a** (34.0 mg, 0.197 mmol) as a white solid: Mp 91 °C (hexane) (lit. 91-93 °C[30]). ^{1}H NMR (400 MHz, $CDCl_3$/TMS) δ 7.63-7.56 (m, 4H), 7.53 (d, 2H, J = 8.3 Hz), 7.45 (t, 2H, J = 7.6 Hz), 7.36 (t, 1H, J = 7.3 Hz), 2.58-2.46 (m, 2H), 2.20-1.90 (m, 6H). ^{13}C NMR (150 MHz, $CDCl_3$) δ 140.7, 140.2, 138.8, 128.8, 127.51, 127.48, 127.0, 126.4, 124.3, 47.5, 40.5, 24.2. LRMS (EI) *m/z*: 247 (M^+). HRMS Calcd. for $C_{18}H_{17}N$: 247.1361, found: 247.1349. IR (neat): 2986, 2953, 2878, 2231, 1598, 1485, 1452, 1006, 841, 766 cm^{-1}.

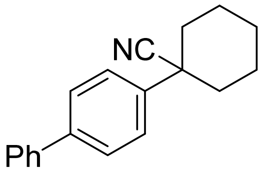

1-([1,1'-Biphenyl]-4-yl)cyclohexane-1-carbonitrile (3ar). According to the general procedure analogous to that described for **3aa**, except that the reaction was conducted with **2r** (43.1 mg, 0.395 mmol) at 120 °C, **3ar** (29.9 mg, 0.114 mmol, 58%) was obtained from **1a** (34.1 mg, 0.198 mmol) as a white solid: Mp 110 °C (hexane). ^{1}H NMR (400 MHz, $CDCl_3$/TMS) δ 7.64-7.54 (m, 6H), 7.45 (t, 2H, J = 7.6 Hz), 7.36 (t, 1H, J = 7.6 Hz), 2.20 (d, 2H, J = 11.7 Hz), 1.94-1.75 (m, 7H), 1.38-1.22 (m, 1H). ^{13}C NMR (150 MHz, $CDCl_3$) δ 140.7, 140.4, 140.3, 128.8, 127.52, 127.51, 127.0, 126.0, 122.7, 44.1, 37.4, 25.0, 23.6. LRMS (EI) *m/z*: 261 (M^+). HRMS Calcd. for $C_{19}H_{19}N$: 261.1517, found: 261.1518. IR (neat): 2944, 2931, 2229, 1597, 1486, 1407, 1126, 1006, 840, 766 cm^{-1}.

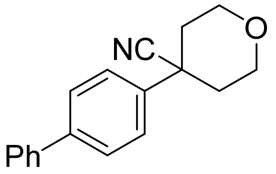

4-([1,1'-Biphenyl]-4-yl)tetrahydro-2*H*-pyran-4-carbonitrile (3as). According to the general procedure analogous to that described for **3aa**, except that the reaction was conducted with **2s** (45.7 mg, 0.411 mmol) at 120 °C and the crude material was purified by preparative thin-layer chromatography of silica gel (toluene:AcOEt = 8:1), **3as** (39.0 mg, 0.148 mmol, 75%) was obtained from **1a** (33.9 mg, 0.197 mmol) as a white solid: Mp 129 °C (hexane/CH_2Cl_2). ^{1}H NMR (400 MHz, $CDCl_3$/TMS) δ 7.65 (d, 2H, J = 8.3 Hz), 7.60-7.54 (m, 4H), 7.46 (t, 2H, J = 7.6 Hz), 7.37 (t, 1H, J = 7.6 Hz), 4.15-4.08 (m, 2H), 4.00-3.90 (m, 2H), 2.23-2.13 (m, 2H), 2.12-2.07 (m, 2H). ^{13}C NMR (150 MHz, $CDCl_3$) δ 141.2, 140.0, 138.7, 128.8, 127.8, 127.7, 127.0, 125.9, 121.7, 65.0, 41.6, 36.7. LRMS (EI) *m/z*: 263 (M^+). HRMS Calcd. for $C_{18}H_{17}NO$: 263.1310, found: 263.1305. IR (neat): 2960, 2860, 2233, 1583, 1485, 1241, 1128, 1031, 835, 763 cm^{-1}.

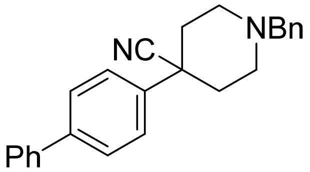

4-([1,1'-Biphenyl]-4-yl)-1-benzylpiperidine-4-carbonitrile (3at). According to the general procedure analogous to that described for **3aa**, except that the reaction was conducted with **2t** (80.7 mg, 0.403 mmol) at 120 °C and the crude material was purified by preparative thin-layer chromatography of silica gel (toluene:AcOEt = 10:1), **3at** (49.8 mg, 0.141 mmol, 71%) was obtained from **1a** (34.3 mg, 0.199 mmol) as a white solid: Mp 104 °C (hexane/CH_2Cl_2). ^{1}H NMR (400 MHz, $CDCl_3$/TMS) δ 7.65-7.55 (m, 6H), 7.45 (t, 2H, J = 7.6 Hz), 7.40-7.26 (m, 6H), 3.62 (s, 2H), 3.03 (d, 2H, J = 12.2 Hz), 2.58-2.50 (m, 2H), 2.20-2.08 (m, 4H). ^{13}C NMR (150 MHz, $CDCl_3$) δ 140.9, 140.1, 139.2, 138.1, 129.0, 128.8, 128.3, 127.6, 127.5, 127.2, 127.0, 126.0, 122.1, 62.9, 50.7, 42.5, 36.6. LRMS (EI) *m/z*: 352 (M^+). HRMS Calcd. for $C_{25}H_{24}N_2$: 352.1939, found: 352.1928. IR (neat): 2950, 2769, 2235, 1600, 1486, 1452, 1119, 990, 835, 766 cm^{-1}.

Procedure of *t*-Bu-P4-catalyzed reaction of 1a with 2a on a 1.0 mmol scale (Table 1, entry 10).

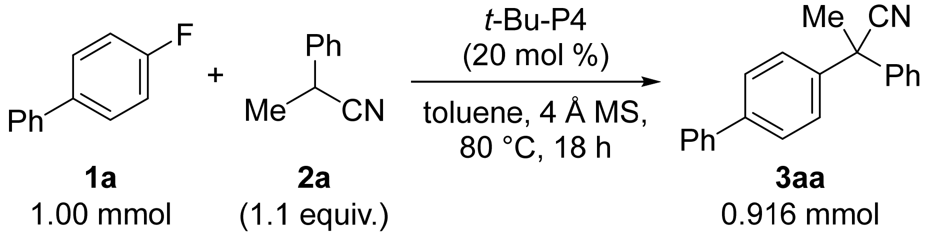

In a glove box under an Ar atmosphere, to a mixture of **1a** (172.6 mg, 1.002 mmol) and **2a** (145.0 mg, 1.105 mmol) in toluene (1.5 mL) were added *t*-Bu-P4 solution (250 µL, 0.8 M in hexane, 0.20 mmol) and 4 Å molecular sieves (500 mg) in an oven-dried vial equipped with a stirrer bar. The vial was sealed with a cap containing an inner Teflon film and taken outside the glove box. After stirring at 80 °C for 18 h, saturated NH_4Cl aqueous solution (5 mL) was added to the reaction mixture at 0 ºC. The mixture was extracted with AcOEt (30 mL × 3). The combined organic layers were collected, washed with brine (30 mL), dried over Na_2SO_4, and concentrated. The crude material was purified by preparative thin-layer chromatography of silica gel (toluene), to afford **3aa** (259.6 mg, 0.916 mmol, 91%).

General procedure of *t*-Bu-P4-catalyzed etherification of fluoroarenes (Table S3 and Figure 3A).

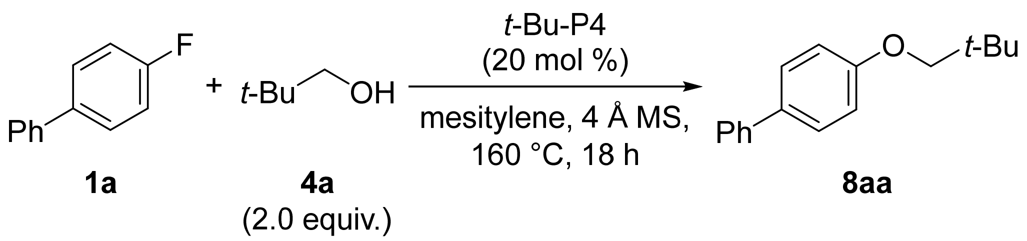

4-(Neopentyloxy)-1,1'-biphenyl (8aa). In a glove box under an Ar atmosphere, to a mixture of **1a** (35.1 mg, 0.204 mmol) and **4a** (35.2 mg, 0.399 mmol) in mesitylene (0.3 mL) were added *t*-Bu-P4 solution (50 µL, 0.8 M in hexane, 0.04 mmol) and 4 Å molecular sieves (100 mg) in an oven-dried vial equipped with a stirrer bar. The vial was sealed with a cap containing an inner Teflon film, and taken outside the glove box. After stirring at 160 °C for 18 h, saturated NH_4Cl aqueous solution (1 mL) was added to the reaction mixture at 0 ºC. The mixture was extracted with AcOEt (10 mL × 3). The combined organic layers were collected, washed with brine (10 mL), dried over Na_2SO_4, and concentrated. The crude material was purified by column chromatography on silica gel (hexane:AcOEt =20:1) to give **8aa** (37.3 mg, 0.155 mmol, 76%) as a white solid: Mp 68 °C (hexane). ^1H NMR (400 MHz, $CDCl_3$/TMS) δ 7.58-7.49 (m, 4H), 7.41 (t, 2H, J = 7.8 Hz), 7.29 (t, 1H, J = 7.3 Hz), 6.97 (d, 2H, J = 8.8 Hz), 3.63 (s, 3H), 1.05 (s, 9H). ^{13}C NMR (150 MHz, $CDCl_3$) δ 159.2, 140.9, 133.4, 128.7, 128.0, 126.7, 126.5, 114.8, 77.9, 31.9, 26.6. LRMS (EI) *m/z*: 240 (M^+). HRMS Calcd. for $C_{17}H_{20}O$: 240.1514, found: 240.1510. IR (neat): 2957, 2912, 2862, 1606, 1522, 1478, 1251, 1056, 832, 761 cm^-

[1].

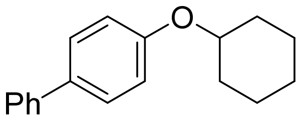

4-(Cyclohexyloxy)-1,1'-biphenyl (8ab). According to the general procedure analogous to that described for **8aa**, except that the crude material was purified by column chromatography on silica gel (hexane:AcOEt =75:1), **8ab** (43.1 mg, 0.171 mmol, 85%) was obtained from **1a** (34.5 mg, 0.200 mmol) as a white solid: Mp 61°C (hexane). ^{1}H NMR (400 MHz, $CDCl_3$/TMS) δ 7.57-7.48 (m, 4H), 7.41 (t, 2H, J = 7.8 Hz), 7.32-7.26 (m, 1H), 6.97 (d, 2H, J = 8.8 Hz), 4.35-4.24 (m, 1H), 2.08-1.98 (m, 2H), 1.88-1.78 (m, 2H), 1.63-1.48 (m, 3H), 1.45-1.24 (m, 3H). ^{13}C NMR (150 MHz, $CDCl_3$) δ 157.4, 140.9, 133.5, 128.7, 128.1, 126.7, 126.5, 116.2, 75.5, 31.8, 25.6, 23.8. LRMS (EI) *m/z*: 252 (M^+). HRMS Calcd. for $C_{18}H_{20}O$: 252.1514, found: 252.1515. IR (neat): 2933, 2854, 1604, 1488, 1239, 1172, 1049, 968, 832, 760 cm^{-1}. The spectra data matched those reported in the literature.[31]

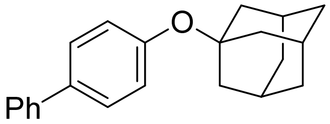

1-([1,1'-Biphenyl]-4-yloxy)adamantane (8ac). According to the general procedure analogous to that described for **8aa**, except that the reaction was conducted in DMI at 200 °C and the crude material was purified by column chromatography on silica gel (hexane:CH_2Cl_2 = 2:1), **8ac** (52.3 mg, 0.172 mmol, 86%) was obtained from **1a** (34.2 mg, 0.199 mmol) as a white solid: Mp 105 °C (hexane). ^{1}H NMR (400 MHz, $CDCl_3$/TMS) δ 7.57 (d, 2H, J = 7.3 Hz), 7.48 (d, 2H, J = 8.8 Hz), 7.42 (d, 2H, J = 7.8 Hz), 7.31 (t, 1H, J = 7.6 Hz), 7.05 (d, 2H, J = 8.3 Hz), 2.19 (s, 3H), 1.97-1.87 (m, 6H), 1.70-1.58 (m, 6H). ^{13}C NMR (100 MHz, $CDCl_3$) δ 153.6, 140.7, 136.4, 128.6, 127.3, 126.80, 126.78, 125.1, 77.8, 42.8, 36.1, 30.8. LRMS (EI) *m/z*: 304 (M^+). HRMS Calcd. for $C_{22}H_{24}O$: 304.1827, found: 304.1827. IR (neat): 2910, 2853, 1596, 1486, 1354, 1230, 1062, 923, 856, 728 cm^{-1}.

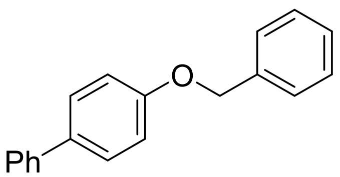

4-(Benzyloxy)-1,1'-biphenyl (8ad). According to the general procedure analogous to that described for **8aa**, except that the reaction was conducted in DMI and the crude material was purified by column chromatography on silica gel (hexane:CH_2Cl_2 = 10:1), **8ad** (38.1 mg, 0.146 mmol, 74%) was obtained from **1a** (33.9 mg, 0.197 mmol) as a white solid: Mp 137 °C (hexane/CH_2Cl_2) (lit. 132.8-134.0 °C[31]).

^{1}H NMR (400 MHz, $CDCl_3$/TMS) δ 7.57-7.51 (m, 4H), 7.46 (d, 2H, J = 7.3 Hz), 7.44-7.37 (m, 4H), 7.36-7.26 (m, 2H), 7.05 (d, 2H, J = 8.8 Hz), 5.12 (s, 2H). ^{13}C NMR (150 MHz, $CDCl_3$) δ 158.4, 140.8, 137.0, 134.0, 128.7, 128.6, 128.2, 128.0, 127.5, 126.72, 126.67, 115.1, 70.1. LRMS (EI) *m/z*: 260 (M^+). HRMS Calcd. for $C_{19}H_{16}O$: 260.1201, found: 260.1207. IR (neat): 3039, 2908, 2866, 1608, 1523, 1487, 1246, 1021, 826, 750 cm^{-1}. The spectra data matched those reported in the literature.[32]

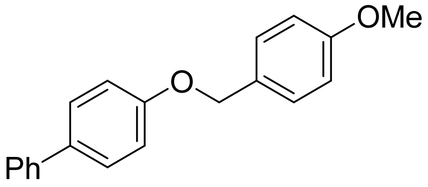

4-((4-Methoxybenzyl)oxy)-1,1'-biphenyl (8ae). According to the general procedure analogous to that described for **8aa**, except that the reaction was conducted in DMI and the crude material was purified by column chromatography on silica gel (hexane:CH_2Cl_2 = 3:1), **8ae** (44.0 mg, 0.152 mmol, 76%) was obtained from **1a** (34.4 mg, 0.200 mmol) as a white solid: Mp 158 °C (hexane/CH_2Cl_2). ^{1}H NMR (400 MHz, $CDCl_3$/TMS) δ 7.57-7.50 (m, 4H), 7.44-7.36 (m, 4H), 7.33-7.26 (m, 1H), 7.04 (d, 2H, J = 8.8 Hz), 6.93 (d, 2H, J = 8.3 Hz), 5.04 (s, 2H), 3.83 (s, 3H). ^{13}C NMR (150 MHz, $CDCl_3$) δ 159.5, 158.4, 140.8, 133.9, 129.2, 129.0, 128.7, 128.1, 126.7, 126.6, 115.1, 114.0, 69.9, 55.3. LRMS (EI) *m/z*: 290 (M^+). HRMS Calcd. for $C_{20}H_{18}O_2$: 290.1307, found: 290.1305. IR (neat): 2971, 2880, 1604, 1516, 1240, 1177, 1007, 810, 763 cm^{-1}. The spectra data matched those reported in the literature.[33]

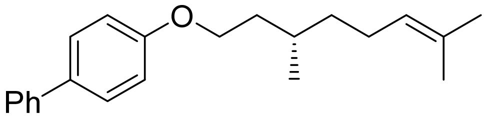

(*S*)-4-((3,7-Dimethyloct-6-en-1-yl)oxy)-1,1'-biphenyl (8af). According to the general procedure analogous to that described for **8aa**, except that the crude material was purified by column chromatography on silica gel (hexane:CH_2Cl_2 = 10:1), **8af** (51.3 mg, 0.166 mmol, 84%) was obtained from **1a** (33.9 mg, 0.197 mmol) as a colorless oil. ^{1}H NMR (400 MHz, $CDCl_3$/TMS) δ 7.57-7.49 (m, 4H), 7.41 (t, 2H, J =7.8 Hz), 7.29 (t, 1H, J = 7.1 Hz), 6.97 (d, 2H, J = 8.8 Hz), 5.11 (t, 1H, J = 6.6 Hz), 4.09-3.98 (m, 2H), 2.12-1.94 (m, 2H), 1.92-1.80 (m, 1H), 1.77-1.56 (m, 8H), 1.46-1.35 (m, 1H), 1.29-1.18 (m, 1H), 0.97 (d, 3H, J = 6.3 Hz). ^{13}C NMR (150 MHz, $CDCl_3$) δ 158.7, 140.9, 133.5, 131.3, 128.7, 128.1, 126.7, 126.6, 124.7, 114.8, 66.3, 37.1, 36.2, 29.5, 25.7, 25.5, 19.6, 17.7. LRMS (EI) *m/z*: 308 (M^+). HRMS Calcd. for $C_{22}H_{28}O$: 308.2140, found: 308.2145. IR (neat): 3038, 2912, 2867, 1609, 1520, 1488, 1246, 1176, 831, 761 cm^{-1}.

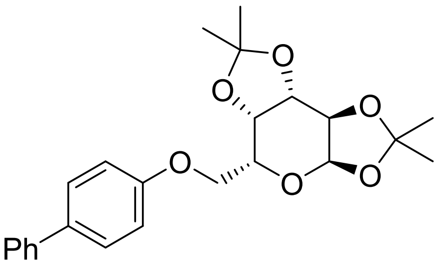

(3a*R*,5*R*,5a*S*,8a*S*,8b*R*)-5-(([1,1'-Biphenyl]-4-yloxy)methyl)-2,2,7,7-tetramethyltetrahydro-5*H*-bis([1,3]dioxolo)[4,5-*b*:4',5'-*d*]pyran (8ag). According to the general procedure analogous to that described for **8aa**, except that the crude material was purified by preparative thin-layer chromatography of silica gel (hexane:AcOEt = 5:1), **8ag** (71.6 mg, 0.174 mmol, 88%) was obtained from **1a** (34.0 mg, 0.197 mmol) as a white solid: Mp 121 °C (hexane/CH_2Cl_2). ^{1}H NMR (400 MHz, $CDCl_3$/TMS) δ 7.59-7.49 (m, 4H), 7.41 (t, 2H, J = 7.6 Hz), 7.33-7.26 (m, 1H), 7.02 (d, 2H, J = 8.8 Hz), 5.59 (d, 1H, J = 4.9 Hz), 4.66 (dd, 1H, J = 7.8, 2.4 Hz), 4.42-4.31 (m, 2H), 4.29-4.13 (m, 3H), 1.53 (s, 3H), 1.48 (s, 3H), 1.37 (s, 3H), 1.35 (s, 3H). ^{13}C NMR (150 MHz, $CDCl_3$, 40 °C) δ 158.3, 140.9, 134.1, 128.7, 128.1, 126.7, 126.6, 115.2, 109.5, 108.7, 96.4, 71.0, 70.8, 70.7, 66.8, 66.2, 26.04, 26.01, 24.9, 24.5. LRMS (EI) *m/z*: 412 (M^+). HRMS Calcd. for $C_{24}H_{28}O_6$: 412.1886, found: 412.1868. IR (neat): 2983, 2896, 1607, 1491, 1452, 1381, 1251, 1069, 1005, 772 cm^{-1}.

General procedure of *t*-Bu-P4-catalyzed amination of fluoroarenes (Table S5 and Figure 3B).

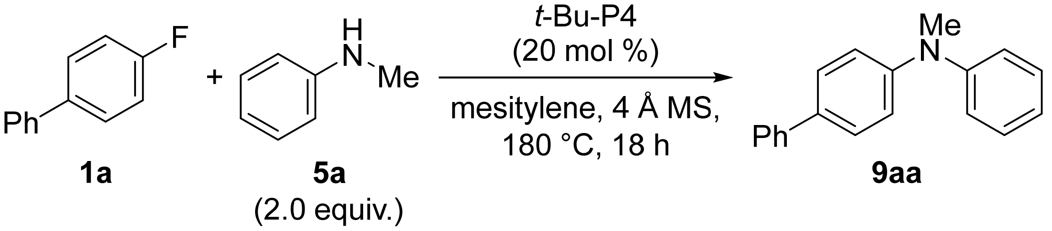

***N*-Methyl-*N*-phenyl-[1,1'-biphenyl]-4-amine (9aa)**. In a glove box under an Ar atmosphere, to a mixture of **1a** (34.3 mg, 0.199 mmol) and **5a** (46.0 mg, 0.429 mmol) in mesitylene (0.3 mL) were added *t*-Bu-P4 solution (50 µL, 0.8 M in hexane, 0.04 mmol) and 4 Å molecular sieves (100 mg) in an oven-dried vial equipped with a stirrer bar. The vial was sealed with a cap containing an inner Teflon film, and taken outside the glove box. After stirring at 180 °C for 18 h, saturated NH_4Cl aqueous solution (1 mL) was added to the reaction mixture at 0 ºC. The mixture was extracted with AcOEt (10 mL × 3). The combined organic layers were collected, washed with brine (10 mL), dried over Na_2SO_4, and concentrated. The crude material was purified by column chromatography on silica gel (hexane:CH_2Cl_2 =10:1) to give **9aa** (43.5 mg, 0.168 mmol, 84%) as a white solid: Mp 101 °C (hexane/CH_2Cl_2). ^{1}H NMR (400 MHz, $CDCl_3$/TMS) δ 7.57 (d, 2H, J = 7.8 Hz), 7.50 (d, 2H, J = 8.8 Hz), 7.41 (t, 2H, J = 7.8 Hz), 7.35-7.26 (m, 3H), 7.11 (d, 2H, J = 8.8 Hz), 7.06 (d, 2H, J = 8.8 Hz), 7.01 (t, 1H, J = 7.6 Hz), 3.36 (s, 3H). ^{13}C NMR (150 MHz, DMSO-d_6, 40 °C) δ 148.3, 148.0, 139.8, 132.1, 129.3, 128.8, 127.3, 126.5, 125.9, 121.9, 121.3, 119.2, 39.9. LRMS (EI) *m/z*: 259 (M^+). HRMS

Calcd. for $C_{19}H_{17}N$: 259.1361, found: 259.1341. IR (neat): 2953, 1588, 1483, 1343, 1254, 1208, 1116, 868, 834 cm^{-1}. The spectra data matched those reported in the literature.[34]

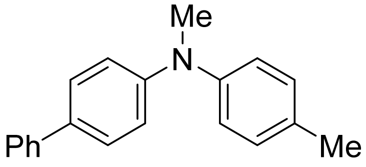

***N*-Methyl-*N*-(*p*-tolyl)-[1,1'-biphenyl]-4-amine (9ab)**. According to the general procedure analogous to that described for **9aa**, except that the reaction was conducted at 160 °C and the crude material was purified by column chromatography on silica gel (hexane:AcOEt = 30:1), **9ab** (43.1 mg, 0.158 mmol, 77%) was obtained from **1a** (35.2 mg, 0.204 mmol) as a white solid: Mp 124 °C (hexane/CH_2Cl_2). ^1H NMR (400 MHz, $CDCl_3$/TMS) δ 7.55 (d, 2H, J = 7.8 Hz), 7.47 (d, 2H, J = 8.8 Hz), 7.40 (t, 2H, J = 7.6 Hz), 7.30-7.26 (m, 1H), 7.15 (d. 2H, J = 8.3 Hz), 7.06 (d, 2H, J = 8.3 Hz), 6.96 (d, 2H, J = 8.8 Hz), 3.33 (s, 3H), 2.34 (s, 3H). ^{13}C NMR (150 MHz, $CDCl_3$) δ 148.6, 146.3, 140.9, 132.6, 132.0, 130.0, 128.6, 127.5, 126.4, 126.3, 123.3, 117.5, 40.2, 20.7. LRMS (EI) *m/z*: 273 (M^+). HRMS Calcd. for $C_{20}H_{19}N$: 273.1517, found: 273.1504. IR (neat): 2942, 1513, 1345, 1261, 1207, 1141, 1078, 872, 819, 762 cm^{-1}.

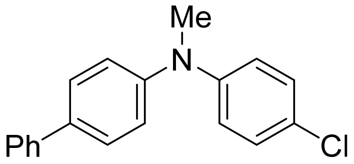

***N*-(4-Chlorophenyl)-*N*-methyl-[1,1'-biphenyl]-4-amine (9ac)**. According to the general procedure analogous to that described for **9aa**, **9ac** (48.3 mg, 0.164 mmol, 83%) was obtained from **1a** (34.0 mg, 0.197 mmol) as a white solid: Mp 139 °C (hexane/CH_2Cl_2). ^1H NMR (400 MHz, $CDCl_3$/TMS) δ 7.57 (d, 2H, J = 8.5 Hz), 7.52 (d, 2H, J = 8.8 Hz), 7.42 (t, 2H, J = 7.6 Hz), 7.31 (t, 1H, J = 7.3 Hz), 7.26-7.22 (m, 2H), 7.08 (d, 2H, J = 8.8 Hz), 6.99 (d, 2H, J = 9.0 Hz), 3.34 (s, 3H). ^{13}C NMR (100 MHz, $CDCl_3$) δ 147.9, 147.4, 140.7, 134.4, 129.2, 128.7, 127.9, 126.7, 126.6, 126.2, 121.6, 120.7, 40.3. LRMS (EI) *m/z*: 293 (M^+). HRMS Calcd. for $C_{19}H_{16}ClN$: 293.0971, found: 293.0969. IR (neat): 2954, 2920, 1587, 1483, 1335, 1252, 1143, 1100, 823, 763 cm^{-1}.

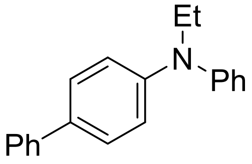

***N*-Ethyl-*N*-phenyl-[1,1'-biphenyl]-4-amine (9ad)**. According to the general procedure analogous to that described for **9aa**, **9ad** (32.9 mg, 0.120 mmol, 60%) was obtained from **1a** (34.3 mg, 0.199 mmol) as a white solid: Mp 62 °C (hexane/CH_2Cl_2). ^1H NMR (400 MHz, $CDCl_3$/TMS) δ 7.56 (d, 2H, J = 7.3

Hz), 7.49 (d, 2H, J = 8.8 Hz), 7.41 (t, 2H, J = 7.8 Hz), 7.34-7.26 (m, 3H), 7.08 (d, 2H, J = 7.8 Hz), 7.04-6.97 (m, 3H), 3.82 (q, 2H, J = 7.2 Hz) 1.26 (t, 3H, J = 7.1 Hz). ^{13}C NMR (150 MHz, $CDCl_3$) δ 147.4, 147.1, 140.9, 133.1, 129.3, 128.7, 127.8, 126.50, 126.46, 122.2, 122.0, 119.8, 46.5, 12.7. LRMS (EI) m/z: 273 (M^+). HRMS Calcd. for $C_{20}H_{19}N$: 273.1517, found: 273.1523. IR (neat): 1591, 1485, 1371, 1243, 1062, 975, 844, 789, 760, 746 cm^{-1}.

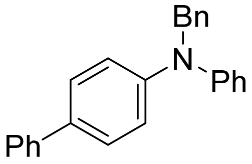

***N*-Benzyl-*N*-phenyl-[1,1'-biphenyl]-4-amine (9ae)**. According to the general procedure analogous to that described for **9aa**, **9ae** (43.8 mg, 0.131 mmol, 65%) was obtained from **1a** (34.7 mg, 0.202 mmol) as a white solid: Mp 140 °C (hexane/CH_2Cl_2). ^{1}H NMR (400 MHz, $CDCl_3$/TMS) δ 7.60-7.14 (m, 16H), 7.09 (d, 2H, J = 8.8 Hz), 6.99 (t, 1H, J = 7.1 Hz), 5.05 (s, 2H). ^{13}C NMR (150 MHz, $CDCl_3$) δ 147.8, 147.4, 140.8, 139.0, 133.5, 129.3, 128.7, 128.6, 127.8, 126.8, 126.52, 126.50, 126.48, 122.1, 121.7, 119.8, 56.3. LRMS (EI) m/z: 335 (M^+). HRMS Calcd. for $C_{25}H_{21}N$: 335.1674, found: 335.1688. IR (neat): 1588, 1493, 1376, 1269, 1233, 1198, 1020, 825, 760, 701 cm^{-1}.

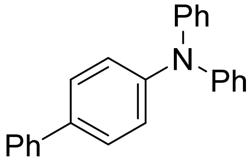

***N*,*N*-Diphenyl-[1,1'-biphenyl]-4-amine (9af)**. According to the general procedure analogous to that described for **9aa**, **9af** (51.6 mg, 0.161 mmol, 81%) was obtained from **1a** (34.2 mg, 0.199 mmol) as a white solid: Mp 111 °C (hexane/CH_2Cl_2) (lit. 115-116 °C[35]). ^{1}H NMR (400 MHz, $CDCl_3$/TMS) δ 7.57 (d, 2H, J = 7.3 Hz), 7.47 (d, 2H, J = 8.8 Hz), 7.42 (t, 2H, J = 7.8 Hz), 7.34-7.23 (m, 5H), 7.17-7.10 (m, 6H), 7.03 (t, 2H, J = 7.3 Hz). ^{13}C NMR (150 MHz, $CDCl_3$) δ 147.7, 147.2, 140.6, 135.1, 129.3, 128.7, 127.7, 126.8, 126.6, 124.4, 123.9, 122.9. LRMS (EI) m/z: 321 (M^+). HRMS Calcd. for $C_{24}H_{19}N$: 321.1517, found: 321.1521. IR (neat): 1737, 1728, 1588, 1484, 1367, 1281, 1217, 899, 747, 700 cm^{-1}. The spectra data matched those reported in the literature.[35]

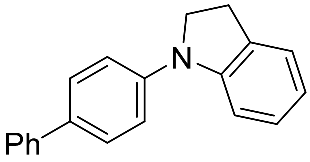

1-([1,1'-Biphenyl]-4-yl)indoline (9ag). According to the general procedure analogous to that described for **9aa**, except that the crude material was purified by column chromatography on silica gel (hexane:AcOEt = 10:1), **9ag** (46.9 mg, 0.173 mmol, 88%) was obtained from **1a** (33.7 mg, 0.196

mmol) as a white solid: Mp 138 °C (hexane/CH_2Cl_2). ^{1}H NMR (400 MHz, $CDCl_3$/TMS) δ 7.61-7.57 (m, 4H), 7.43 (t, 2H, J = 7.8 Hz), 7.33-7.27 (m, 3H), 7.19 (t, 2H, J = 6.6 Hz), 7.10 (t, 1H, J = 7.6 Hz), 6.77 (t, 1H, J = 7.6 Hz), 4.01 (t, 2H, J = 8.5 Hz), 3.16 (t, 2H, J = 8.3 Hz). ^{13}C NMR (100 MHz, $CDCl_3$) δ 146.7, 143.4, 140.8, 133.4, 131.3, 128.7, 127.7, 127.1, 126.6, 126.5, 125.1, 119.0, 117.6, 108.3, 52.0, 28.1. LRMS (EI) *m/z*: 271 (M^+). HRMS Calcd. for $C_{20}H_{17}N$: 271.1361, found: 271.1354. IR (neat): 1534, 1483, 1334, 1170, 1059, 878, 834, 759, 739, 719 cm^{-1}. The spectra data matched those reported in the literature.[36]

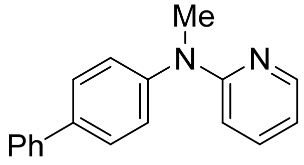

***N*-([1,1'-Biphenyl]-4-yl)-*N*-methylpyridin-2-amine (9ah)**. According to the general procedure analogous to that described for **9aa**, except that the crude material was purified by column chromatography on silica gel (hexane:AcOEt = 10:1), **9ah** (45.5 mg, 0.175 mmol, 87%) was obtained from **1a** (34.5 mg, 0.200 mmol) as a white solid: Mp 86 °C (hexane/CH_2Cl_2). ^{1}H NMR (400 MHz, $CDCl_3$/TMS) δ 8.26 (s, 1H), 7.65-7.57 (m, 4H), 7.45 (t, 2H, J = 7.6 Hz), 7.38-7.30 (m, 4H), 6.69-6.61 (m, 2H), 3.52 (s, 3H). ^{13}C NMR (150 MHz, $CDCl_3$) δ 158.7, 147.8, 146.0, 140.5, 138.0, 136.6, 128.8, 128.2, 127.2, 126.9, 126.1, 113.4, 109.4, 38.4. LRMS (EI) *m/z*: 260 (M^+). HRMS Calcd. for $C_{18}H_{16}N_2$: 260.1313, found: 260.1309. IR (neat): 1557, 1485, 1439, 1325, 1155, 1072, 980, 887, 767, 742 cm^{-1}. The spectra data matched those reported in the literature.[37]

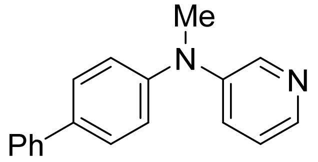

***N*-([1,1'-Biphenyl]-4-yl)-*N*-methylpyridin-3-amine (9ai)**. According to the general procedure analogous to that described for **9aa**, the crude material was purified by column chromatography on silica gel (hexane:AcOEt = 10:1), **9ai** (49.0 mg, 0.188 mmol, 94%) was obtained from **1a** (34.5 mg, 0.200 mmol) as a white solid: Mp 98 °C (hexane/CH_2Cl_2). ^{1}H NMR (400 MHz, $CDCl_3$/TMS) δ 8.40 (s, 1H), 8.19 (s, 1H), 7.61-7.52 (m, 4H), 7.43 (t, 2H, J = 7.6 Hz), 7.35-7.28 (m, 2H), 7.22-7.12 (m, 3H), 3.39 (s, 3H). ^{13}C NMR (100 MHz, $CDCl_3$) δ 147.2, 144.9, 141.7, 141.5, 140.5, 135.5, 128.7, 128.1, 126.9, 126.7, 125.7, 123.5, 121.7, 40.0. LRMS (EI) *m/z*: 260 (M^+). HRMS Calcd. for $C_{18}H_{16}N_2$: 260.1313, found: 260.1343. IR (neat): 1580, 1531, 1476, 1343, 1264, 1147, 1120, 838, 762, 721 cm^{-1}.

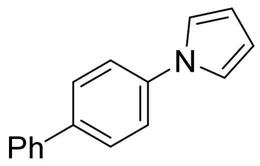

1-([1,1'-Biphenyl]-4-yl)-1*H*-pyrrole (9aj). According to the general procedure analogous to that described for **9aa**, **9aj** (36.5 mg, 0.166 mmol, 82%) was obtained from **1a** (35.1 mg, 0.204 mmol) as a white solid: Mp 196 °C (hexane/CH_2Cl_2) (lit. 185-186 °C[38]). ^{1}H NMR (400 MHz, $CDCl_3$/TMS) δ 7.65 (d, 2H, J = 8.8 Hz), 7.61 (d, 2H, J = 7.3 Hz), 7.50-7.42 (m, 4H), 7.38-7.33 (m, 1H), 7.15-7.12 (m, 2H), 6.38-6.36 (m, 2H). ^{13}C NMR (150 MHz, $CDCl_3$) δ 140.2, 139.9, 138.5, 128.8, 128.1, 127.4, 126.9, 120.7, 119.2, 110.5. LRMS (EI) *m/z*: 219 (M^+). HRMS Calcd. for $C_{16}H_{13}N$: 219.1048, found: 219.1036. IR (neat): 1607, 1531, 1409, 1327, 1250, 1068, 920, 829, 763, 719 cm^{-1}. The spectra data matched those reported in the literature.[38]

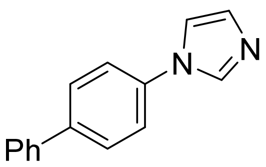

1-([1,1'-Biphenyl]-4-yl)-1*H*-imidazole (9ak). According to the general procedure analogous to that described for **9aa**, except that the crude material was purified by column chromatography on silica gel (hexane:AcOEt = 1:1), **9ak** (40.4 mg, 0.183 mmol, 93%) was obtained from **1a** (33.8 mg, 0.196 mmol) as a white solid: Mp 157 °C (hexane/CH_2Cl_2) (lit. 150-152 °C[39]). ^{1}H NMR (400 MHz, $CDCl_3$/TMS) δ 7.90 (s, 1H), 7.70 (d, 2H, J = 8.8 Hz), 7.61 (d, 2H, J = 7.8 Hz), 7.51-7.45 (m, 4H), 7.39 (t, 1H, J = 7.3 Hz), 7.26-7.22 (m, 2H). ^{13}C NMR (150 MHz, $CDCl_3$) δ 140.3, 139.6, 136.3, 135.4, 130.4, 128.8, 128.3, 127.7, 126.8, 121.5, 118.0. LRMS (EI) *m/z*: 220 (M^+). HRMS Calcd. for $C_{15}H_{12}N_2$: 220.1000, found: 220.1017. IR (neat): 1531, 1488, 1307, 1268, 1060, 962, 905, 839, 766, 742 cm^{-1}. The spectra data matched those reported in the literature.[40]

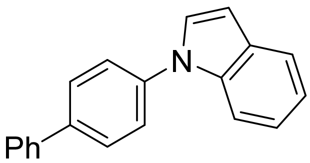

1-([1,1'-Biphenyl]-4-yl)-1*H*-indole (9al). According to the general procedure analogous to that described for **9aa**, **9al** (44.1 mg, 0.164 mmol, 79%) was obtained from **1a** (35.7 mg, 0.207 mmol) as a white solid: Mp 137 °C (hexane/CH_2Cl_2). ^{1}H NMR (400 MHz, $CDCl_3$/TMS) δ 7.74 (d, 2H, J = 8.3 Hz), 7.70 (d, 1H, J = 7.8 Hz), 7.67-7.56 (m, 5H), 7.48 (t, 2H, J = 7.6 Hz), 7.42-7.36 (m, 2H), 7.26-7.22 (m, 1H), 7.18 (t, 1H, J = 7.3 Hz), 6.71 (d, 1H, J = 3.4 Hz). ^{13}C NMR (100 MHz, $CDCl_3$) δ 140.2, 139.3, 139.0, 135.8, 129.4, 128.9, 128.2, 127.8, 127.5, 127.0, 124.5, 122.4, 121.2, 120.4, 110.6, 103.7. LRMS (EI) *m/z*: 269 (M^+). HRMS Calcd. for $C_{20}H_{15}N$: 269.1204, found: 269.1211. IR (neat): 1531, 1453, 1335, 1213, 1134, 977, 872, 839, 750, 724 cm^{-1}. The spectra data matched those reported in the literature.[41]

General procedure of *t*-Bu-P4-catalyzed thiolation of fluoroarenes (Table S7 and Figure 3C).

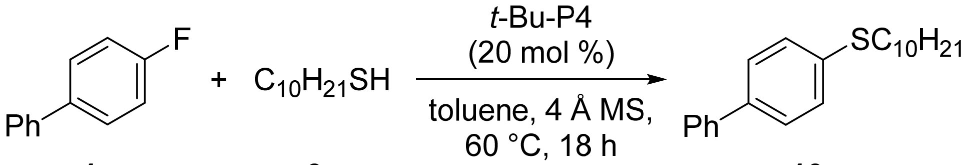

1a **6a** (1.1 equiv.) **10aa**

[1,1'-Biphenyl]-4-yl(decyl)sulfane (10aa). In a glove box under an Ar atmosphere, to a solution of **1a** (34.5 mg, 0.200 mmol) in toluene (0.3 mL) were added *t*-Bu-P4 solution (50 µL, 0.8 M in hexane, 0.04 mmol) and 4 Å molecular sieves (100 mg) in an oven-dried vial equipped with a stirrer bar. The vial was sealed with a cap containing an inner Teflon film, and taken outside the glove box. **6a** (45.7 µL, 0.220 mmol) was added to the mixture under a nitrogen atmosphere. After stirring at 60 °C for 18 h, saturated NH_4Cl aqueous solution (1 mL) was added to the reaction mixture at 0 ºC. The mixture was extracted with AcOEt (10 mL × 3). The combined organic layers were collected, washed with brine (10 mL), dried over Na_2SO_4, and concentrated. The crude material was purified by column chromatography on silica gel (hexane:CH_2Cl_2 =20:1) to give **10aa** (62.4 mg, 0.191 mmol, 95%) as a white solid: Mp 85 °C (hexane) (lit. 87.2-87.5 °C[42]). ^{1}H NMR (400 MHz, $CDCl_3$/TMS) δ 7.57 (d, 2H, J = 8.3 Hz), 7.51 (d, 2H, J = 8.3 Hz), 7.46-7.30 (m, 5H), 2.95 (t, 2H, J = 7.3 Hz), 1.68 (quint, 2H, J = 7.6 Hz), 1.47-1.38 (m, 2H), 1.34-1.19 (m, 12H), 0.88 (t, 3H, J = 6.3 Hz). ^{13}C NMR (150 MHz, C_6D_6) δ 141.0, 139.0, 137.0, 129.5, 129.1, 128.3, 127.5, 127.2, 33.7, 32.3, 29.99, 29.96, 29.8, 29.6, 29.5, 29.2, 23.1, 14.4. LRMS (EI) *m/z*: 326 (M^+). HRMS Calcd. for $C_{22}H_{30}S$: 326.2068, found: 326.2053. IR (neat): 2916, 2849, 1598, 1479, 1380, 1245, 1101, 1003, 824, 752 cm^{-1}.

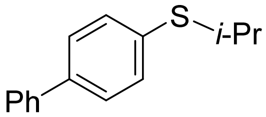

[1,1'-Biphenyl]-4-yl(isopropyl)sulfane (10ab). According to the general procedure analogous to that described for **10aa**, **10ab** (43.2 mg, 0.189 mmol, 95%) was obtained from **1a** (34.4 mg, 0.200 mmol) as a white solid: Mp 49 °C (lit. 50-51 °C[43]). ^{1}H NMR (400 MHz, $CDCl_3$/TMS) δ 7.60-7.56 (m, 2H), 7.55-7.51 (m, 2H), 7.48-7.41 (m, 4H), 7.34 (t, 1H, J = 7.3 Hz), 3.42 (sept, 1H, J = 6.7 Hz), 1.33 (d, 6H, J = 6.8 Hz). ^{13}C NMR (150 MHz, $CDCl_3$) δ 140.4, 139.5, 134.6, 132.0, 128.8, 127.4, 127.3, 126.9, 38.2, 23.1. LRMS (EI) *m/z*: 228 (M^+). HRMS Calcd. for $C_{15}H_{16}S$: 228.0973, found: 228.0970. IR (neat): 2956, 2916, 2850, 1597, 1479, 1381, 1245, 1100, 825, 760 cm^{-1}.

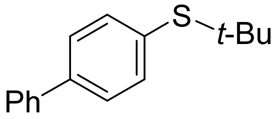

[1,1'-Biphenyl]-4-yl(*tert*-butyl)sulfane (10ac). According to the general procedure analogous to that

described for **10aa**, except that the reaction was conducted in THF, **10ac** (40.1 mg, 0.165 mmol, 83%) was obtained from **1a** (34.3 mg, 0.199 mmol) as a white solid: Mp 107 °C (hexane/CH_2Cl_2) (lit. 106-108 °C[43]). ^1H NMR (400 MHz, $CDCl_3$/TMS) δ 7.63-7.54 (m, 6H), 7.45 (t, 2H, J = 7.6 Hz), 7.36 (t, 1H, J = 7.3 Hz), 1.33 (s, 9H). ^{13}C NMR (150 MHz, $CDCl_3$) δ 141.5, 140.3, 137.8, 131.7, 128.8, 127.6, 127.08, 127.06, 46.0, 31.0. LRMS (EI) *m/z*: 242 (M^+). HRMS Calcd. for $C_{16}H_{18}S$: 242.1129, found: 242.1145. IR (neat): 2959, 2929, 2867, 1484, 1377, 1246, 1176, 1023, 830, 760 cm^{-1}. The spectra data matched those reported in the literature.[44]

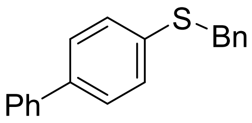

[1,1'-Biphenyl]-4-yl(benzyl)sulfane (10ad). According to the general procedure analogous to that described for **10aa**, except that the crude material was purified by preparative thin-layer chromatography of silica gel (hexane:CH_2Cl_2 =5:1) and GPC ($CHCl_3$), **10ad** (36.6 mg, 0.132 mmol, 65%) was obtained from **1a** (34.9 mg, 0.203 mmol) as a white solid: Mp 127-129 °C (lit. 127.5-128.5 °C[45]). ^1H NMR (400 MHz, $CDCl_3$/TMS) δ 7.56 (d, 2H, J = 7.2 Hz), 7.49 (d, 2H, J = 8.4 Hz), 7.43 (t, 2H, J = 7.6 Hz), 7.38-7.23 (m, 8H), 4.16 (s, 2H). ^{13}C NMR (100 MHz, $CDCl_3$) δ 140.4, 139.2, 137.4, 135.5, 130.0, 128.82, 128.78, 128.5, 127.5, 127.3, 127.2, 126.9, 39.0. LRMS (EI) *m/z*: 276 (M^+). HRMS Calcd. for $C_{19}H_{16}S$: 276.0973, found: 276.0977. IR (neat): 3058, 3036, 2921, 1596, 1479, 823, 754 cm^{-1}. The spectra data matched those reported in the literature.[46]

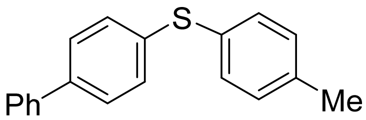

[1,1'-Biphenyl]-4-yl(*p*-tolyl)sulfane (10ae). According to the general procedure analogous to that described for **10aa**, except that the reaction was conducted in mesitylene at 160 °C and the crude material was purified by column chromatography on silica gel (hexane:AcOEt =40:1), **10ae** (52.5 mg, 0.190 mmol, 95%) was obtained from **1a** (34.6 mg, 0.201 mmol) as a white solid: Mp 105-107 °C (lit. 102-104 °C[47]). ^1H NMR (400 MHz, $CDCl_3$/TMS) δ 7.56 (d, 2H, J = 7.2 Hz), 7.50 (d, 2H, J = 8.4 Hz), 7.43 (t, 2H, J = 7.8 Hz), 7.37-7.30 (m, 5H), 7.16 (d, 2H, J = 8.0 Hz), 2.36 (s, 3H). ^{13}C NMR (100 MHz, $CDCl_3$) δ 140.4, 139.4, 137.7, 136.3, 132.4, 131.2, 130.11, 130.05, 128.8, 127.7, 127.4, 126.9, 21.1. LRMS (EI) *m/z*: 276 (M^+). HRMS Calcd. for $C_{19}H_{16}S$: 276.0973, found: 276.0975. IR (neat): 3038, 2916, 2861, 1909, 1591, 1478, 1393, 1179, 1085, 1003, 811, 755 cm^{-1}. The spectra data matched those reported in the literature.[47]

General procedure of *t*-Bu-P4-catalyzed phosphination of fluoroarenes (Figures 3D).

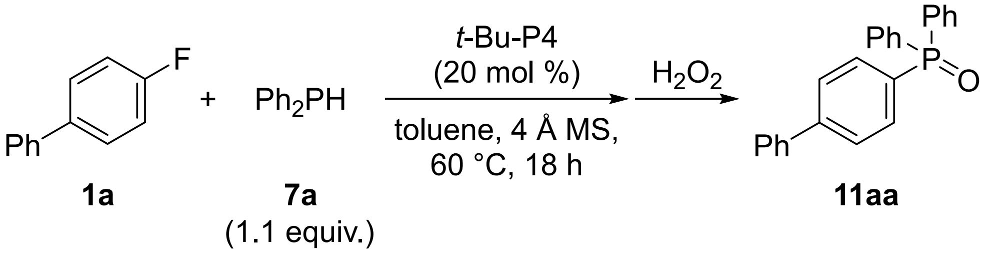

[1,1'-Biphenyl]-4-yldiphenylphosphine oxide (11aa). In a glove box under an Ar atmosphere, to a mixture of **1a** (34.2 mg, 0.199 mmol) and **7a** (41.9 mg, 0.225 mmol) in toluene (0.3 mL) were added *t*-Bu-P4 solution (50 µL, 0.8 M in hexane, 0.04 mmol) and 4 Å molecular sieves (100 mg) in an oven-dried vial equipped with a stirrer bar. The vial was sealed with a cap containing an inner Teflon film, and taken outside the glove box. After stirring at 80 °C for 18 h, 30% hydrogen peroxide (0.6 mL) was added to the reaction mixture at 0 ºC. After stirring at room temperature for 20 min, saturated $Na_2S_2O_3$ solution (6.0 mL) was added. The mixture was extracted with CH_2Cl_2 (10 mL × 3). The combined organic layers were collected, washed with brine (10 mL), dried over Na_2SO_4, and concentrated. The crude material was purified by preparative thin-layer chromatography of silica gel (CH_2Cl_2:AcOEt = 4:1), to afford **11aa** (58.8 mg, 0.166 mmol, 84%) as a white solid: Mp 148-150 °C (lit. 156.0-156.8 °C[48]). ^{1}H NMR (400 MHz, $CDCl_3$/TMS) δ 7.79-7.66 (m, 8H), 7.63-7.54 (m, 4H), 7.52-7.44 (m, 6H), 7.39 (t, 1H, *J* = 7.2 Hz). ^{13}C NMR (150 MHz, $CDCl_3$) δ 144.7 (d, *J* = 3.0 Hz), 140.0, 132.6 (d, *J* = 103.8 Hz), 132.5 (d, *J* = 10.0 Hz), 132.1 (d, *J* = 10.0 Hz), 131.9 (d, *J* = 2.0 Hz), 131.1 (d, *J* = 104.8 Hz), 128.9, 128.5 (d, *J* = 12.0 Hz), 128.1, 127.2, 127.1 (d, *J* = 12.0 Hz). ^{31}P NMR (243 MHz, $CDCl_3$) δ 28.9. LRMS (EI) *m/z*: 353 (M^+). HRMS Calcd. for $C_{24}H_{19}OP$: 354.1174, found: 354.1175. IR (neat): 3058, 3029, 2924, 2333, 1966, 1821, 1599, 1484, 1436, 1393, 1192, 1119, 1007, 840, 749, 730 cm^{-1}. The spectra data matched those reported in the literature.[49]

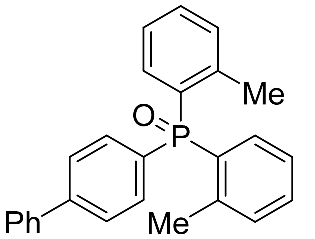

[1,1'-Biphenyl]-4-yldi-o-tolylphosphine oxide (11ab). According to the general procedure analogous to that described for **11aa**, except that the reaction was conducted at 140 °C and the crude material was purified by preparative thin-layer chromatography of silica gel (CH_2Cl_2:AcOEt = 5:1), **11ab** (65.3 mg, 0.171 mmol, 85%) was obtained from **1a** (34.5 mg, 0.200 mmol) as a white solid: Mp 213-214 °C. ^{1}H NMR (400 MHz, $CDCl_3$/TMS) δ 7.72-7.67 (m, 3H), 7.67-7.61 (m, 3H), 7.49-7.36 (m, 5H), 7.33-7.28 (m, 2H), 7.18-7.04 (m, 4H), 2.54 (s, 6H). ^{13}C NMR (150 MHz, $CDCl_3$) δ 144.4 (d, *J* = 3.0 Hz), 143.4 (d, *J* = 8.0 Hz), 139.8, 132.9 (d, *J* = 13.0 Hz), 132.7 (d, *J* = 10.0 Hz), 132.0 (d, *J* = 10.0 Hz), 131.9 (d, *J* = 2.0 Hz), 131.3 (d, *J* = 103.8 Hz), 130.9 (d, *J* = 101.8 Hz), 128.9, 128.1, 127.2, 127.1 (d, *J* = 12.0

Hz), 125.3 (d, J = 13.0 Hz), 21.9 (d, J = 5.0 Hz). ^{31}P NMR (243 MHz, $CDCl_3$) δ 34.9. LRMS (EI) *m/z*: 381 (M^+). HRMS Calcd. for $C_{26}H_{23}OP$: 382.1487, found: 382.1515. IR (neat): 3060, 2930, 2327, 1950, 1825, 1599, 1453, 1393, 1277, 1186, 1111, 1007, 841, 806, 755 cm^{-1}.

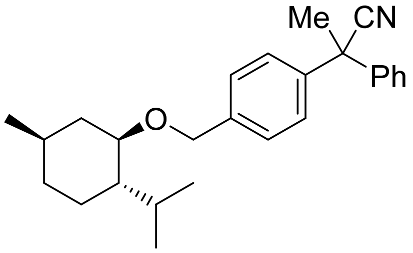

2-(4-((((1*R*,2*S*,5*R*)-2-Isopropyl-5-methylcyclohexyl)oxy)methyl)phenyl)-2-phenylpropanenitrile (3Ma). According to the general procedure analogous to that described for **3aa**, except that the reaction was conducted at 120 °C and the crude material was purified by preparative thin-layer chromatography of silica gel (hexane:toluene = 1:1), **3Ma** (66.7 mg, 0.178 mmol, 89%) was obtained from **1M** (52.2 mg, 0.197 mmol) as a colorless oil. ^{1}H NMR (400 MHz, $CDCl_3$/TMS) δ 7.38-7.27 (m, 9H), 4.64 (d, 1H, J = 11.7 Hz), 4.38 (d, 1H, J = 11.7 Hz), 3.20-3.13 (m, 1H), 2.30-2.16 (m, 2H), 2.08 (s, 3H), 1.70-1.58 (m, 2H), 1.42-1.23 (m, 2H), 1.03-0.78 (m, 9H), 0.70 (dd, 3H, J = 6.8, 1.9 Hz). ^{13}C NMR (100 MHz, DMSO-d_6) δ 141.3, 140.1, 139.1, 129.0, 128.1, 127.9, 126.24, 126.18, 123.4, 78.0, 68.8, 47.8, 45.6, 34.1, 30.9, 27.1, 25.2, 22.9, 22.3, 20.9, 16.0. LRMS (EI) *m/z*: 375 (M^+). HRMS Calcd. for $C_{26}H_{33}NO$: 375.2562, found: 375.2565. IR (neat): 2953, 2925, 2867, 2236, 1508, 1493, 1448, 1370, 1084, 1018, 921, 811, 756 cm^{-1}.

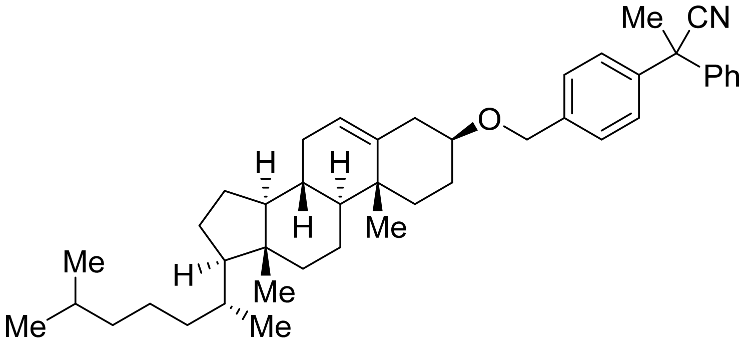

2-(4-((((3*S*,8*S*,9*S*,10*R*,13*R*,14*S*,17*R*)-10,13-Dimethyl-17-((*R*)-6-methylheptan-2-yl)-2,3,4,7,8,9,10,11,12,13,14,15,16,17-tetradecahydro-1*H*-cyclopenta[*a*]phenanthren-3-yl)oxy)methyl)phenyl)-2-phenylpropanenitrile (3Na). According to the general procedure analogous to that described for **3aa**, except that the reaction was conducted at 120 °C and the crude material was purified by preparative thin-layer chromatography of silica gel (toluene), **3Na** (105.3 mg, 0.174 mmol, 87%) was obtained from **1N** (98.8 mg, 0.200 mmol) as a white solid: Mp 128-130 °C. ^{1}H NMR (400 MHz, $CDCl_3$/TMS) δ 7.41-7.26 (m, 9H), 5.35 (d, 1H, J = 4.5 Hz), 4.54 (s, 2H), 3.34-3.21 (m, 1H), 2.4-2.36 (m, 1H), 2.33-2.22 (m, 1H), 2.08 (s, 3H), 2.05-1.91 (m, 3H), 1.91-1.76 (m, 2H),

1.70-0.93 (m, 24H), 0.91 (d, 3H, J = 6.4 Hz), 0.87 (d, 6H, J = 6.6 Hz), 0.68 (s, 3H). ^{13}C NMR (100 MHz, $CDCl_3$/TMS) δ 141.2, 140.8, 140.3, 138.9, 128.8, 127.9, 127.8, 126.60, 126.55, 123.3, 121.6, 78.8, 69.3, 56.7, 56.1, 50.1, 45.9, 42.3, 39.7, 39.5, 39.1, 37.2, 36.8, 36.2, 35.7, 31.9, 31.8, 28.4, 28.2, 28.1, 27.9, 24.2, 23.8, 22.8, 22.5, 21.0, 19.3, 18.7, 11.8. LRMS (EI) *m/z*: 606 (M^+). HRMS Calcd. for $C_{43}H_{59}NO$: 605.4597, found: 605.4606. IR (neat): 2925, 2866, 2236, 1741, 1456, 1374, 1257, 1157, 1087, 1016, 811, 757 cm^{-1}.

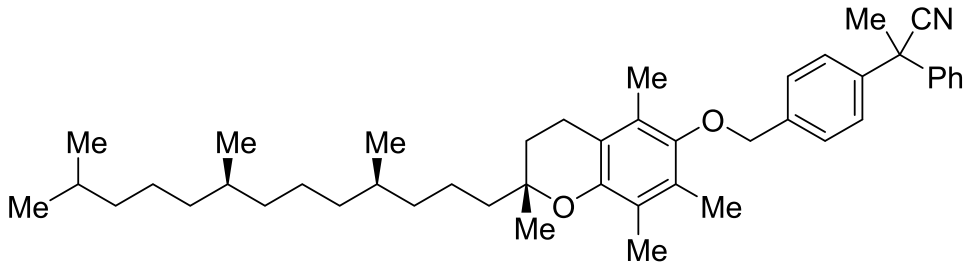

2-Phenyl-2-(4-((((*R*)-2,5,7,8-tetramethyl-2-((4*R*,8*R*)-4,8,12-trimethyltridecyl)chroman-6-yl)oxy)methyl)phenyl)propanenitrile (3Oa). According to the general procedure analogous to that described for **3aa**, except that the reaction was conducted at 120 °C and the crude material was purified by column chromatography on silica gel (hexane: AcOEt = 20:1) and GPC ($CHCl_3$), **3Oa** (96.5 mg, 0.148 mmol, 74%) was obtained from **1O** (108.0 mg, 0.200 mmol) as a colorless oil. ^{1}H NMR (400 MHz, $CDCl_3$/TMS) δ 7.49 (d, 2H, J = 8.2 Hz), 7.43-7.28 (m, 7H), 4.68 (s, 2H), 2.58 (t, 2H, J = 6.7 Hz), 2.20 (s, 3H), 2.15 (s, 3H), 2.10 (s, 3H), 2.09 (s, 3H), 1.87-1.71 (m, 2H), 1.61-1.01 (m, 24H), 0.87-0.84 (m, 12H). ^{13}C NMR (100 MHz, $CDCl_3$) δ 147.9, 147.8, 141.2, 140.7, 137.8, 128.8, 128.0, 127.9, 127.8, 126.7, 126.6, 125.8, 123.3, 122.9, 117.6, 74.8, 73.9, 45.9, 39.9, 39.3, 37.40, 37.36, 37.2, 32.7, 32.6, 31.2, 28.1, 27.9, 24.8, 24.4, 23.8, 22.7, 22.6, 21.0, 20.6, 19.7, 19.6, 12.8, 11.9, 11.8. LRMS (EI) *m/z*: 649 (M^+). HRMS Calcd. for $C_{45}H_{63}NO_2$: 649.4859, found: 649.4871. IR (neat): 2972, 2934, 2364, 1512, 1457, 1216, 1091, 814, 757 cm^{-1}.

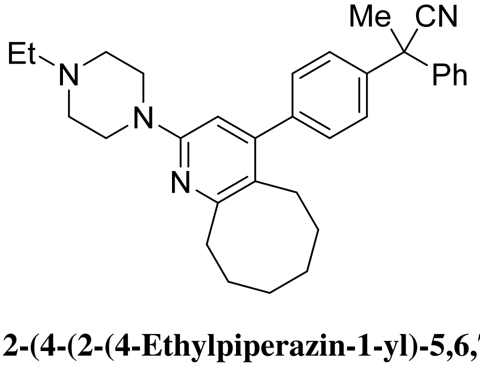

2-(4-(2-(4-Ethylpiperazin-1-yl)-5,6,7,8,9,10-hexahydrocycloocta[*b*]pyridin-4-yl)phenyl)-2-phenylpropanenitrile (3Pa). According to the general procedure analogous to that described for **3aa**, except that the crude material was purified by preparative thin-layer chromatography of silica gel (CH_2Cl_2:AcOEt = 1:2, TEA:1%), **3Pa** (43.4 mg, 0.091 mmol, 91%) was obtained from 1**P** (36.7 mg, 0.100 mmol) as a brown oil. ^{1}H NMR (600 MHz, $CDCl_3$/TMS) δ 7.45-7.36 (m, 6H), 7.33 (t, 1H, J =

7.3 Hz), 7.27-7.23 (m, 2H), 6.29 (s, 1H), 3.58-3.52 (m, 4H), 2.88 (t, 2H, *J* = 6.0 Hz), 2.61-2.55 (m, 6H), 2.49 (q, 2H, *J* = 7.2 Hz), 2.13 (s, 3H), 1.82-1.75 (m, 2H), 1.47-1.32 (m, 6H), 1.14 (t, 3H, *J* = 7.3 Hz). ^{13}C NMR (100 MHz, $CDCl_3$) δ 160.0, 157.2, 150.4, 141.2, 141.1, 140.2, 129.0, 128.9, 128.0, 126.6, 126.3, 123.4, 122.7, 105.8, 52.7, 52.4, 46.0, 45.4, 35.5, 31.6, 30.6, 28.2, 26.6, 26.5, 25.8, 11.8. LRMS (EI) *m/z*: 479 (M^+). HRMS Calcd. for $C_{32}H_{38}N_4$: 478.3096, found: 478.3085. IR (neat): 2924, 2851, 2237, 1588, 1540, 1448, 1410, 1244, 1166, 999, 830, 754 cm^{-1}.

Computational section.

Calculation methods.

All calculations were performed with the Gaussian 16 (revision C.01) program.[50] Geometries were fully optimized and characterized by frequency calculation using ONIOM method,[51] combiniang ωB97X-D density functional theory (DFT) with 6-311+g(2d,p) basis set in the high level layer (Figures S4-S6, red structure) and B97D[52] density functional theory (DFT) with 6-311g(d,p) basis set[53] in the low level layer (Figures S4-S6, blue structure), with the SCRF method based on CPCM (toluene).[54] The intrinsic reaction coordinate (IRC) calculations[55] were performed at same level of theory. The calculations of the natural atomic charges[56] in the optimized structures were performed by using NBO7 (version 7.0.10) program at the M06-2X/6-311+g(2d,p). The calculations of the natural energy decomposition analysis[57] in the optimized structures were performed by using NBO7 (version 7.0.10) program at the M06-2X/6-311+g(2d,p). The calculations of the non-covalent interaction (NCI) analysis[58] were performed by using Multiwfn (version 3.6) program with PROAIMS wavefunction files written at the M06-2X/6-311+g(2d,p),[59] and the graphics were depicted by using VMD (version 1.9.4a53) program.[60] Nucleus independent chemical shifts (NICS) were calculated using the gauge invariant atomic orbital (GIAO) approach at the GIAO-B3LYP/6-311+G(2d,p) level of theory and performed with the Aroma package (1.0) of Stanger and coworkers using cphf = grid = fine and integral = grid = ultrafine keywords.[61]

Figure S4. Molecules used in the calculations

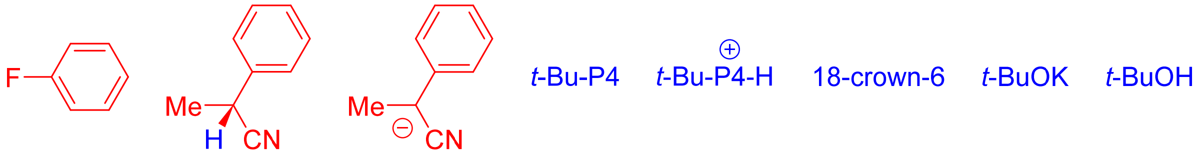

A conformational search of the transition-state with *t*-Bu-P4 gave six conformers, with **TS-1** being the most stable conformer (Figure S5).

Figure S5. Conformers of the transition states obtained by the calculations with *t*-Bu-P4

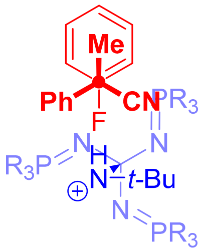

TS-1
+0.00 kcal/mol

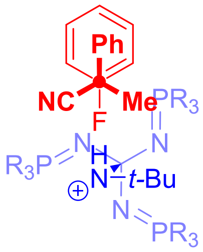

TS-1-a
+2.78 kcal/mol

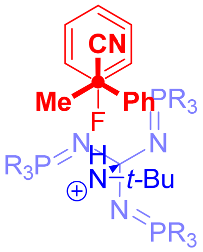

TS-1-b
+4.07 kcal/mol

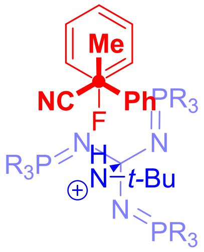

TS-1-c
+0.31 kcal/mol

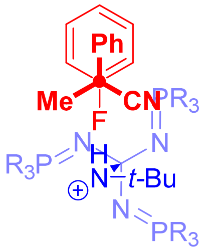

TS-1-d
+3.42 kcal/mol

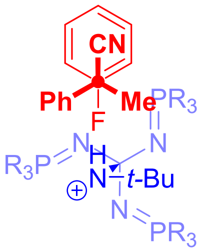

TS-1-e
+5.51 kcal/mol

From the IRC calculation of **TS-1**, the structures before (**Int-2**) and after (**Int-3**) **TS-1** were found.

To search for the Meisenheimer complex structure, calculations were performed using 4-fluoronitrobenzene with an electron-withdrawing group instead of **1a**, employing the same calculation method. However, the Meisenheimer complex structure could not be found.

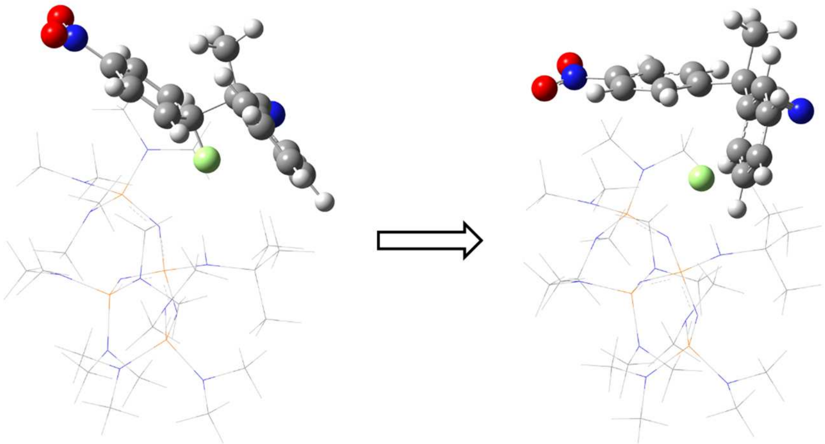

input structure   optimized structure

A similar transition state conformational search was performed with KO-*t*-Bu–18-crown-6 and the most stable conformer **TS-2** was found (Figure S6).

Figure S6. Conformers of the transition states obtained by the calculations with KO-*t*-Bu–18-crown-6

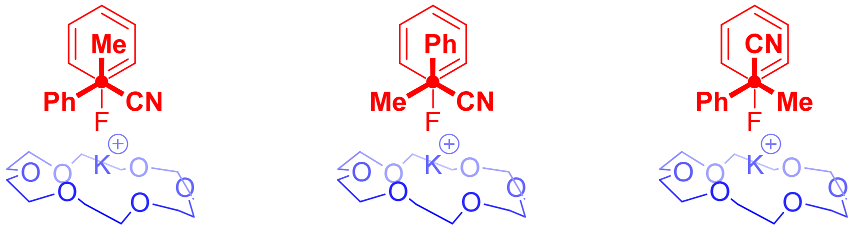

TS-2 +0.00 kcal/mol | **TS-2-a** +2.86 kcal/mol | **TS-2-b** +7.84 kcal/mol

From the IRC calculation of **TS-2**, the structures before (**Int-6**) and after (**Int-7**) the transition state were found.

Cartesian coordinates.

t-Bu-P4

Sum of electronic and thermal Free Energies= -2952.137423

Center Number	Atomic Number	Atomic Type	Coordinates (Angstroms) X	Y	Z
1	15	0	-0.139306	-0.072739	0.766116
2	7	0	0.884962	-1.362055	0.468105
3	7	0	-1.272880	0.015624	-0.448955
4	7	0	0.835758	1.269631	0.596129
5	15	0	-2.761088	-0.503321	-0.525626
6	15	0	0.919848	2.635039	-0.143782
7	15	0	2.011052	-1.932999	-0.444954
8	7	0	1.379126	2.738633	-1.790358
9	7	0	2.212097	3.499772	0.564492
10	7	0	-0.540111	3.501292	-0.162931
11	7	0	-2.818435	-2.046645	-1.239679
12	7	0	-3.635270	0.552413	-1.521760
13	7	0	-3.781861	-0.584107	0.825502
14	6	0	-0.261792	-0.557061	3.441605
15	6	0	-1.269305	-0.182622	4.553862
16	1	0	-0.884594	-0.462701	5.547484
17	1	0	-1.458509	0.900788	4.536804
18	1	0	-2.223509	-0.701087	4.383925
19	6	0	1.068689	0.184057	3.737019
20	1	0	0.908424	1.269783	3.700721
21	1	0	1.454894	-0.086117	4.733280
22	1	0	1.824130	-0.077193	2.984455
23	6	0	0.004386	-2.085006	3.523294
24	1	0	0.416344	-2.367237	4.506799
25	1	0	-0.937652	-2.630950	3.365765
26	1	0	0.707311	-2.375728	2.734074
27	6	0	-3.958232	0.654050	1.608582
28	1	0	-4.890726	0.565775	2.188257
29	1	0	-3.099634	0.800065	2.280451
30	1	0	-4.045715	1.510980	0.931260
31	6	0	-3.696342	-1.772545	1.689911
32	1	0	-2.821026	-1.703530	2.352517
33	1	0	-4.619381	-1.827143	2.288041
34	1	0	-3.626523	-2.681361	1.082883
35	6	0	-4.095108	-2.621171	-1.662908
36	1	0	-4.588220	-3.192600	-0.853930
37	1	0	-4.778032	-1.836219	-2.000711
38	1	0	-3.915358	-3.307741	-2.505072
39	6	0	-1.792613	-3.049204	-0.941327
40	1	0	-0.869971	-2.562671	-0.617249
41	1	0	-2.122062	-3.748751	-0.150096
42	1	0	-1.587144	-3.634056	-1.852375
43	6	0	-2.996653	1.070402	-2.731162
44	1	0	-3.334031	0.518456	-3.628446
45	1	0	-3.260225	2.132932	-2.861452
46	1	0	-1.911658	0.988673	-2.640072
47	6	0	-5.092917	0.665717	-1.548076
48	1	0	-5.377634	1.731147	-1.557032
49	1	0	-5.516683	0.195875	-2.455711
50	1	0	-5.525688	0.188542	-0.664690
51	6	0	2.503289	4.861927	0.109590
52	1	0	3.558652	5.090442	0.325439
53	1	0	2.341592	4.939973	-0.970503
54	1	0	1.879128	5.617891	0.622123
55	6	0	2.489834	3.307254	1.991009
56	1	0	1.866447	3.964637	2.627451
57	1	0	2.301779	2.266557	2.265383
58	1	0	3.547032	3.552236	2.179853
59	6	0	-1.533281	3.280689	0.892713
60	1	0	-1.465724	4.063752	1.671420
61	1	0	-2.540280	3.322841	0.448487
62	1	0	-1.401610	2.298778	1.357633
63	6	0	-0.681680	4.800026	-0.824240
64	1	0	-1.682931	4.866924	-1.281467
65	1	0	-0.576668	5.635706	-0.108783
66	1	0	0.070372	4.909648	-1.611510
67	6	0	2.718422	2.217260	-2.122100
68	1	0	2.749239	1.114755	-2.092734
69	1	0	2.980723	2.559890	-3.134118
70	1	0	3.453804	2.607587	-1.411972
71	6	0	0.388294	2.351437	-2.805058
72	1	0	0.282724	1.257333	-2.877502
73	1	0	-0.587411	2.779513	-2.564489
74	1	0	0.720189	2.739040	-3.780314
75	6	0	2.144208	-3.427581	-2.819309
76	1	0	2.708864	-2.984693	-3.660818
77	1	0	2.823693	-4.031822	-2.211629
78	1	0	1.362567	-4.079689	-3.244282
79	6	0	3.763411	-0.024088	0.490509
80	1	0	2.885148	0.257058	1.074443
81	1	0	4.527379	-0.493033	1.138202
82	1	0	4.188011	0.894606	0.060227
83	6	0	1.580963	-4.457318	0.363683
84	1	0	0.968902	-4.234396	1.254777
85	1	0	0.921178	-4.518403	-0.508820
86	1	0	2.078236	-5.428749	0.502150
87	6	0	0.655375	-1.484041	-2.754250
88	1	0	-0.074952	-2.077335	-3.327118
89	1	0	0.094074	-0.830064	-2.077133

90	1	0	1.241457	-0.873703	-3.465690
91	7	0	1.517444	-2.392264	-1.996656
92	6	0	4.459274	-1.273816	-1.499580
93	1	0	4.084383	-1.813139	-2.374424
94	1	0	4.951767	-0.352653	-1.847958
95	1	0	5.218608	-1.901044	-0.995331
96	7	0	3.358906	-0.909753	-0.607209
97	6	0	3.547298	-3.337217	1.268843
98	1	0	3.976244	-4.334002	1.445106
99	1	0	4.364416	-2.649122	1.028893
100	1	0	3.053845	-2.999909	2.198778
101	7	0	2.612171	-3.428339	0.137856
102	7	0	-0.883150	-0.191621	2.170375

PhF (**1v**)

Sum of electronic and thermal Free Energies= -331.409694

Center Number	Atomic Number	Atomic Type	Coordinates (Angstroms) X	Y	Z
1	6	0	-0.259702	-1.211502	-0.000011
2	6	0	1.128647	-1.201869	0.000013
3	6	0	1.824431	0.000002	-0.000004
4	6	0	1.128618	1.201888	0.000008
5	6	0	-0.259701	1.211497	-0.000007
6	6	0	-0.922646	-0.000019	-0.000043
7	1	0	-0.823706	-2.135369	0.000007
8	1	0	1.666740	-2.142090	0.000022
9	1	0	2.907251	0.000027	0.000007
10	1	0	1.666729	2.142099	0.000019
11	1	0	-0.823765	2.135329	0.000013
12	9	0	-2.270126	0.000002	0.000022

PhCHMeCN (**2a**)

Sum of electronic and thermal Free Energies= -402.970142

Center Number	Atomic Number	Atomic Type	Coordinates (Angstroms) X	Y	Z
1	6	0	2.069915	-0.864530	0.911133
2	6	0	0.686398	-0.759134	0.942920
3	6	0	0.015510	0.000669	-0.008367
4	6	0	0.746263	0.653882	-0.994952
5	6	0	2.128806	0.547688	-1.028930
6	6	0	2.794344	-0.210724	-0.075266
7	1	0	2.580906	-1.462583	1.656190
8	1	0	0.124245	-1.274879	1.714344
9	1	0	0.233645	1.242546	-1.748382
10	1	0	2.687359	1.056727	-1.805250
11	1	0	3.874004	-0.295217	-0.104102
12	6	0	-1.492399	0.156552	0.093051
13	1	0	-1.894487	-0.703194	0.635643
14	6	0	-1.883194	1.439912	0.846188
15	1	0	-2.966613	1.523634	0.938649
16	1	0	-1.443786	1.413657	1.843743
17	1	0	-1.504417	2.319306	0.323586
18	6	0	-2.127949	0.134005	-1.229970
19	7	0	-2.633400	0.135551	-2.260569

Int-1

Sum of electronic and thermal Free Energies= -3355.110918

Center Number	Atomic Number	Atomic Type	Coordinates (Angstroms) X	Y	Z
1	6	0	5.406274	-0.418357	-0.277649
2	6	0	5.733418	0.305096	0.896379
3	6	0	3.951853	1.905221	0.548542
4	6	0	5.425415	2.269260	2.517196
5	6	0	6.771007	-0.238854	1.688953
6	6	0	5.051159	1.505116	1.272970
7	6	0	6.051427	-1.590644	-0.616180
8	6	0	7.412965	-1.416686	1.336844
9	6	0	7.065037	-2.113191	0.186289
10	1	0	4.630179	-0.032003	-0.926857
11	1	0	5.763047	-2.106741	-1.526570
12	1	0	7.072389	0.269593	2.597072
13	1	0	8.201460	-1.797335	1.978235
14	1	0	7.570345	-3.032768	-0.082737
15	7	0	-1.898939	0.060748	1.329786
16	15	0	-0.577361	0.044507	0.368498
17	7	0	0.650139	0.686013	1.308339
18	7	0	-0.773208	0.980257	-0.951482
19	7	0	-0.247799	-1.502314	-0.046217
20	15	0	-0.573008	2.512141	-1.322918
21	15	0	-0.141792	-2.318468	-1.386988
22	15	0	-3.392124	-0.385215	1.456207
23	7	0	-1.366982	-2.268157	-2.555004
24	7	0	-0.167491	-3.957754	-0.937423
25	7	0	1.217597	-1.875557	-2.293049
26	7	0	-1.936568	3.231889	-2.048968
27	7	0	0.538613	2.653499	-2.558208
28	7	0	-0.181902	3.484911	-0.018984
29	6	0	1.282551	0.046673	2.501894
30	6	0	1.902149	1.189875	3.328933
31	1	0	2.430297	0.776995	4.200473
32	1	0	2.621248	1.749785	2.722186
33	1	0	1.114181	1.872143	3.678303
34	6	0	2.383092	-0.944960	2.070064
35	1	0	3.161490	-0.429429	1.496764
36	1	0	2.855029	-1.396267	2.955902
37	1	0	1.941395	-1.739379	1.456449
38	6	0	0.247538	-0.689175	3.370631
39	1	0	0.751291	-1.049931	4.278080
40	1	0	-0.575975	-0.021239	3.647473
41	1	0	-0.174465	-1.550657	2.839696
42	6	0	0.812466	4.556305	-0.090852
43	1	0	0.346860	5.539450	-0.288430
44	1	0	1.341448	4.598545	0.871155
45	1	0	1.560650	4.325500	-0.851476
46	6	0	-0.920549	3.411215	1.245935
47	1	0	-0.219191	3.193575	2.063261
48	1	0	-1.438983	4.365467	1.447256
49	1	0	-1.658061	2.602457	1.222457
50	6	0	-2.929363	3.947720	-1.243440
51	1	0	-3.606452	3.275378	-0.688079
52	1	0	-2.424998	4.606087	-0.529560
53	1	0	-3.536210	4.570954	-1.916315
54	6	0	-2.461535	2.639898	-3.283861
55	1	0	-1.634327	2.229238	-3.873768
56	1	0	-3.191759	1.836052	-3.095037
57	1	0	-2.955530	3.427445	-3.873435
58	6	0	1.517630	1.604681	-2.863279
59	1	0	1.562122	1.483432	-3.958602
60	1	0	2.509579	1.867903	-2.472238
61	1	0	1.193295	0.660793	-2.420748
62	6	0	0.728834	3.892244	-3.315961
63	1	0	1.723735	4.322392	-3.116930
64	1	0	0.652072	3.678377	-4.395030
65	1	0	-0.039224	4.624636	-3.048571
66	6	0	-0.016933	-4.981396	-1.979789
67	1	0	-0.497363	-5.908908	-1.633524
68	1	0	-0.507259	-4.656076	-2.902995
69	1	0	1.043836	-5.202303	-2.193810
70	6	0	0.434541	-4.365450	0.339677
71	1	0	1.515780	-4.566983	0.235305
72	1	0	0.289201	-3.582487	1.087420
73	1	0	-0.058582	-5.288915	0.679813
74	6	0	2.431704	-1.458853	-1.572546
75	1	0	3.025238	-0.820619	-2.240032
76	1	0	2.174497	-0.880924	-0.683075
77	1	0	3.053572	-2.319266	-1.274659
78	6	0	1.517048	-2.504323	-3.586610
79	1	0	2.033827	-1.769620	-4.222864
80	1	0	2.174812	-3.382854	-3.470066
81	1	0	0.592775	-2.813730	-4.083652
82	6	0	-2.678330	-2.829285	-2.180839
83	1	0	-3.228771	-2.170446	-1.490735
84	1	0	-3.267148	-2.962351	-3.099669
85	1	0	-2.544579	-3.803300	-1.700728
86	6	0	-1.492283	-1.073374	-3.411519
87	1	0	-2.045959	-0.271137	-2.905299
88	1	0	-0.503208	-0.686737	-3.668561
89	1	0	-2.020840	-1.365068	-4.331661
90	6	0	-5.874963	0.744360	0.813169
91	1	0	-6.553573	0.088106	0.238569
92	1	0	-6.047964	0.586184	1.880875
93	1	0	-6.114707	1.788058	0.555582

94	6	0	-2.617598	-3.024916	1.511904
95	1	0	-1.641286	-2.546282	1.594756
96	1	0	-2.881088	-3.522602	2.462152
97	1	0	-2.549105	-3.783041	0.719275
98	6	0	-3.793506	1.321066	3.485286
99	1	0	-2.742627	1.496289	3.772512
100	1	0	-4.065278	2.031960	2.696640
101	1	0	-4.440233	1.494688	4.357240
102	6	0	-4.186834	0.520204	-0.952206
103	1	0	-4.613352	1.442185	-1.370642
104	1	0	-3.105570	0.536650	-1.128334
105	1	0	-4.637600	-0.338736	-1.481577
106	7	0	-4.468913	0.481188	0.484855
107	6	0	-4.973268	-2.580894	0.994648
108	1	0	-5.655966	-1.836551	0.575240
109	1	0	-4.932617	-3.438892	0.306821
110	1	0	-5.379273	-2.931190	1.961218
111	7	0	-3.625189	-2.026783	1.132140
112	6	0	-3.709757	-1.057945	4.049507
113	1	0	-4.317009	-0.823269	4.934670
114	1	0	-3.983767	-2.058681	3.699779
115	1	0	-2.645159	-1.057055	4.343211
116	7	0	-4.005999	-0.058808	3.010380
117	1	0	1.338220	1.225600	0.764042
118	1	0	4.804476	3.160449	2.624488
119	1	0	5.295559	1.672385	3.430327
120	1	0	6.470845	2.601968	2.500380
121	7	0	2.989136	2.214407	-0.049783

Int-2

Sum of electronic and thermal Free Energies= -3686.513031

Center Number	Atomic Number	Atomic Type	Coordinates (Angstroms) X	Y	Z
1	6	0	3.051942	-4.741675	0.818466
2	6	0	3.770845	-3.761349	1.490900
3	6	0	3.837034	-2.474816	0.975968
4	6	0	3.187597	-2.146729	-0.204115
5	6	0	2.488061	-3.144670	-0.842643
6	6	0	2.398467	-4.436945	-0.367145
7	1	0	2.998707	-5.748656	1.214017
8	1	0	4.408814	-1.710445	1.483827
9	1	0	3.242004	-1.149976	-0.621688
10	1	0	1.836284	-5.180411	-0.917067
11	9	0	1.841448	-2.847160	-2.000616
12	6	0	5.241553	2.349836	-0.168509
13	6	0	6.149648	2.418788	1.029829
14	1	0	5.948438	1.616200	1.757061
15	1	0	7.209764	2.335499	0.758219
16	1	0	6.024944	3.367087	1.555742
17	1	0	4.284432	-4.002723	2.413428
18	15	0	-0.992104	0.026965	-0.377623
19	7	0	-1.669900	1.476321	-0.645477
20	7	0	-2.091746	-1.009148	0.236767
21	7	0	0.337480	0.030637	0.554858
22	15	0	-3.061877	-2.140372	-0.275366
23	15	0	0.598962	-0.108899	2.114984
24	15	0	-2.085380	2.873167	-0.063510
25	7	0	-0.141062	0.981958	3.161292
26	7	0	2.249802	0.039871	2.333810
27	7	0	0.043715	-1.582642	2.710270
28	7	0	-4.527489	-1.475067	-0.806054
29	7	0	-3.340805	-3.174081	1.023860
30	7	0	-2.575125	-3.230630	-1.470675
31	6	0	-0.203195	0.289332	-3.125868
32	6	0	0.489649	-0.657731	-4.125143
33	1	0	0.692543	-0.116407	-5.058634
34	1	0	1.442570	-1.017999	-3.717615
35	1	0	-0.150038	-1.522485	-4.349741
36	6	0	0.743207	1.467603	-2.822922
37	1	0	1.667362	1.102492	-2.360241
38	1	0	1.005540	1.989268	-3.754945
39	1	0	0.266413	2.181006	-2.145264
40	6	0	-1.522996	0.789325	-3.736854
41	1	0	-1.309229	1.375835	-4.641735
42	1	0	-2.161867	-0.061581	-4.009436
43	1	0	-2.056515	1.417093	-3.018960
44	6	0	-1.352414	-4.016043	-1.237122
45	1	0	-1.433179	-4.965446	-1.787688
46	1	0	-0.459360	-3.481123	-1.588998
47	1	0	-1.244283	-4.234788	-0.170497
48	6	0	-2.766492	-2.892361	-2.888501
49	1	0	-1.965869	-2.229257	-3.247794
50	1	0	-2.759461	-3.826078	-3.470007
51	1	0	-3.732749	-2.401841	-3.037974
52	6	0	-5.690312	-2.334336	-1.048385
53	1	0	-5.709102	-2.733704	-2.078891
54	1	0	-5.705013	-3.172572	-0.346688
55	1	0	-6.603823	-1.741068	-0.893665
56	6	0	-4.552065	-0.230192	-1.585587
57	1	0	-3.673847	0.380645	-1.363193
58	1	0	-4.583587	-0.427847	-2.671595
59	1	0	-5.454376	0.337741	-1.312049
60	6	0	-3.376488	-2.633393	2.387006
61	1	0	-4.393533	-2.316594	2.678414
62	1	0	-3.046759	-3.420362	3.081378
63	1	0	-2.684813	-1.790593	2.465318
64	6	0	-3.936182	-4.507492	0.899931
65	1	0	-3.358324	-5.218074	1.512141
66	1	0	-4.980943	-4.519003	1.257699
67	1	0	-3.910546	-4.836507	-0.142880
68	6	0	2.864196	-0.348440	3.602036
69	1	0	2.964150	0.502745	4.299114
70	1	0	2.284016	-1.137944	4.089741
71	1	0	3.870563	-0.741633	3.398583
72	6	0	3.077242	0.931611	1.505376
73	1	0	4.043821	0.450972	1.315844
74	1	0	2.591247	1.112505	0.546361
75	1	0	3.263254	1.896745	2.003072
76	6	0	0.179730	-2.792923	1.893360
77	1	0	1.013842	-3.417165	2.250163
78	1	0	-0.757654	-3.365680	1.937397
79	1	0	0.368271	-2.521994	0.853998
80	6	0	-0.201260	-1.862373	4.125350
81	1	0	-1.161305	-2.390857	4.232818
82	1	0	0.591569	-2.505492	4.547451
83	1	0	-0.243705	-0.930009	4.696366
84	6	0	0.538139	2.103216	3.803532
85	1	0	0.115563	3.052865	3.442763
86	1	0	0.405637	2.054773	4.898004
87	1	0	1.607540	2.091493	3.578303
88	6	0	-1.600128	1.039481	3.245646
89	1	0	-1.956810	2.006351	2.861850
90	1	0	-2.044232	0.243550	2.640257
91	1	0	-1.931344	0.917201	4.291304
92	6	0	-4.379899	3.992684	1.041795
93	1	0	-4.503164	4.149606	2.126433
94	1	0	-3.937731	4.887020	0.594655
95	1	0	-5.379264	3.837141	0.600528
96	6	0	0.546198	3.364796	0.569306
97	1	0	0.693052	2.409958	0.064939
98	1	0	0.951731	4.174323	-0.053246
99	1	0	1.121694	3.347629	1.502322
100	6	0	-3.588830	3.768122	-2.143449
101	1	0	-3.286644	3.179075	-3.025415
102	1	0	-4.374621	3.224220	-1.608197
103	1	0	-3.997026	4.729907	-2.488230
104	6	0	-4.129982	1.568578	1.261515
105	1	0	-5.132246	1.447141	0.818373
106	1	0	-3.517542	0.711253	0.974382
107	1	0	-4.235723	1.585371	2.358436
108	7	0	-3.537652	2.820417	0.785971
109	6	0	-1.156650	4.809514	1.603744
110	1	0	-2.151226	4.788844	2.060583
111	1	0	-0.415200	4.929621	2.405537
112	1	0	-1.083753	5.685735	0.935398
113	7	0	-0.881591	3.546658	0.907403
114	6	0	-1.352235	4.733817	-1.922681
115	1	0	-1.755033	5.649495	-2.378686
116	1	0	-0.581516	5.020153	-1.202739
117	1	0	-0.890149	4.120740	-2.714554
118	7	0	-2.451981	4.029280	-1.247855
119	7	0	-0.498857	-0.540664	-1.908263
120	1	0	0.262125	-1.197951	-1.755444
121	6	0	4.327114	3.371178	-0.373505
122	7	0	3.545999	4.226019	-0.543598
123	6	0	5.230959	1.215393	-1.028179
124	6	0	4.371414	1.114678	-2.154997
125	6	0	6.075869	0.098785	-0.799906
126	6	0	4.380594	0.011416	-2.984317
127	1	0	3.707354	1.941508	-2.379223

128	6	0	6.074996	-1.000831	-1.644050
129	1	0	6.753178	0.106242	0.046279
130	6	0	5.234388	-1.066551	-2.748146
131	1	0	3.708118	-0.009599	-3.835940
132	1	0	6.742668	-1.829003	-1.427103
133	1	0	5.235043	-1.931000	-3.400807

TS-1

Sum of electronic and thermal Free Energies= -3686.481138

Center Number	Atomic Number	Atomic Type	Coordinates (Angstroms) X	Y	Z
1	6	0	4.772175	0.607907	2.480053
2	6	0	4.578894	1.961493	2.741685
3	6	0	3.933902	2.729976	1.767826
4	6	0	3.515782	2.184474	0.575788
5	6	0	3.826336	0.838765	0.256925
6	6	0	4.377604	0.035649	1.284077
7	1	0	5.223326	-0.028210	3.235445
8	1	0	3.724370	3.776526	1.961941
9	1	0	2.979386	2.778120	-0.151584
10	1	0	4.515522	-1.022781	1.103673
11	9	0	2.840874	0.173833	-0.523034
12	6	0	5.193852	0.898302	-1.337870
13	6	0	6.291071	1.743541	-0.721625
14	1	0	6.511621	1.395132	0.291075
15	1	0	7.216586	1.713176	-1.306078
16	1	0	5.969706	2.782250	-0.639397
17	1	0	4.887998	2.398555	3.682568
18	15	0	-1.172253	-0.271077	-0.281047
19	7	0	-2.576749	-0.412477	-1.090231
20	7	0	-1.247952	-0.935002	1.202507
21	7	0	-0.655179	1.258894	-0.191905
22	15	0	-0.895192	-2.328528	1.862202
23	15	0	-0.516069	2.412778	0.879302
24	15	0	-4.030640	0.148057	-1.258273
25	7	0	-1.929697	3.205522	1.374265
26	7	0	0.455321	3.583496	0.176865
27	7	0	0.095745	1.896163	2.346734
28	7	0	-2.192065	-3.405505	1.702460
29	7	0	-0.623493	-2.041761	3.497023
30	7	0	0.494825	-3.171433	1.416830
31	6	0	0.048402	-1.248495	-2.679939
32	6	0	1.402296	-1.920934	-2.968212
33	1	0	1.571043	-1.967087	-4.052336
34	1	0	2.219287	-1.350596	-2.513006
35	1	0	1.422857	-2.943020	-2.566169
36	6	0	0.028930	0.149376	-3.331454
37	1	0	0.829578	0.775436	-2.919057
38	1	0	0.179513	0.056993	-4.416932
39	1	0	-0.935834	0.637426	-3.153878
40	6	0	-1.080862	-2.124075	-3.248677
41	1	0	-0.936000	-2.244567	-4.331930
42	1	0	-1.061366	-3.115990	-2.776107
43	1	0	-2.055039	-1.665393	-3.062475
44	6	0	1.806006	-2.511032	1.550574
45	1	0	2.572977	-3.286155	1.693465
46	1	0	2.071832	-1.920759	0.662423
47	1	0	1.810066	-1.848917	2.420330
48	6	0	0.443932	-4.132463	0.304784
49	1	0	0.539457	-3.622570	-0.664600
50	1	0	1.272289	-4.845072	0.429001
51	1	0	-0.497334	-4.690328	0.323860
52	6	0	-2.232356	-4.634715	2.499521
53	1	0	-1.732441	-5.479874	1.992295
54	1	0	-1.757364	-4.478768	3.472337
55	1	0	-3.284062	-4.909726	2.670184
56	6	0	-2.973329	-3.482661	0.462865
57	1	0	-2.921289	-2.537631	-0.082822
58	1	0	-2.617041	-4.294962	-0.195927
59	1	0	-4.024655	-3.684635	0.720808
60	6	0	-1.409512	-1.022108	4.198225
61	1	0	-2.277421	-1.465534	4.717356
62	1	0	-0.767185	-0.533264	4.946253
63	1	0	-1.749212	-0.264192	3.486818
64	6	0	0.088496	-2.956464	4.392629
65	1	0	0.826279	-2.387142	4.980170
66	1	0	-0.606354	-3.447957	5.096397
67	1	0	0.611454	-3.722318	3.813227
68	6	0	1.031396	4.656844	0.982163
69	1	0	0.462666	5.599338	0.882287
70	1	0	1.062605	4.371827	2.038265
71	1	0	2.064248	4.835412	0.649849
72	6	0	0.575370	3.768210	-1.272643
73	1	0	1.636209	3.887276	-1.538260
74	1	0	0.190924	2.885616	-1.789370
75	1	0	0.021675	4.664211	-1.607970
76	6	0	1.116374	0.837546	2.400456
77	1	0	2.061683	1.241830	2.784165
78	1	0	0.757862	0.026713	3.047114
79	1	0	1.301994	0.434677	1.405899
80	6	0	-0.071377	2.594659	3.621983
81	1	0	-0.408686	1.879564	4.389794
82	1	0	0.888420	3.026229	3.954442
83	1	0	-0.814369	3.392129	3.528627
84	6	0	-2.417315	4.433857	0.751721
85	1	0	-3.248945	4.222507	0.058921
86	1	0	-2.787749	5.120812	1.530224
87	1	0	-1.614421	4.931158	0.199542
88	6	0	-2.994954	2.394816	1.971285
89	1	0	-3.743553	2.120840	1.210143
90	1	0	-2.574260	1.477415	2.396092
91	1	0	-3.488869	2.962791	2.776022
92	6	0	-6.534662	-0.321454	-0.132452
93	1	0	-7.007263	0.434197	0.518023
94	1	0	-6.837436	-0.147899	-1.168676
95	1	0	-6.893627	-1.316071	0.180624
96	6	0	-2.962329	2.489487	-2.219396
97	1	0	-2.008178	2.028702	-1.958816
98	1	0	-3.112167	2.460874	-3.312189
99	1	0	-2.928105	3.541901	-1.908939
100	6	0	-5.006397	-1.990833	-2.585915
101	1	0	-4.088470	-2.507122	-2.912571
102	1	0	-5.265520	-2.335374	-1.579116
103	1	0	-5.825221	-2.253159	-3.271242
104	6	0	-4.598039	-0.534845	1.350368
105	1	0	-4.989747	-1.509496	1.683366
106	1	0	-3.506834	-0.576860	1.385259
107	1	0	-4.956112	0.240937	2.046971
108	7	0	-5.074509	-0.267777	-0.009585
109	6	0	-5.336398	2.527263	-1.557969
110	1	0	-6.047547	2.100642	-0.844127
111	1	0	-5.175772	3.581835	-1.291658
112	1	0	-5.778820	2.488379	-2.569794
113	7	0	-4.049253	1.827683	-1.478726
114	6	0	-4.542874	0.009049	-3.924174
115	1	0	-5.295659	-0.375691	-4.626062
116	1	0	-4.610520	1.100710	-3.917918
117	1	0	-3.543274	-0.289682	-4.284707
118	7	0	-4.843405	-0.526203	-2.588155
119	7	0	-0.056614	-1.189025	-1.182621
120	1	0	0.862899	-0.961809	-0.805398
121	6	0	4.440500	1.587873	-2.323103
122	7	0	3.774617	2.189027	-3.057499
123	6	0	5.503110	-0.503952	-1.631157
124	6	0	4.850862	-1.219466	-2.647980
125	6	0	6.430983	-1.209161	-0.849481
126	6	0	5.080386	-2.568237	-2.845957
127	1	0	4.151558	-0.699775	-3.291803
128	6	0	6.660011	-2.564559	-1.050845
129	1	0	6.980203	-0.694497	-0.071824
130	6	0	5.983648	-3.258716	-2.042450
131	1	0	4.552474	-3.086310	-3.639029
132	1	0	7.380763	-3.079377	-0.424685
133	1	0	6.166222	-4.315204	-2.199759

TS-1-a

Sum of electronic and thermal Free Energies= -3686.476715

Center Number	Atomic Number	Atomic Type	Coordinates (Angstroms) X	Y	Z
1	6	0	4.686350	-0.016658	-2.011159
2	6	0	4.924737	-1.387604	-2.127096
3	6	0	4.520114	-2.209899	-1.079059
4	6	0	3.912321	-1.697050	0.051140
5	6	0	3.750667	-0.301564	0.204832
6	6	0	4.075607	0.521283	-0.900356
7	1	0	4.971245	0.649205	-2.819679

8	1	0	4.665753	-3.283548	-1.148064
9	1	0	3.589266	-2.356086	0.846576
10	1	0	3.902441	1.586706	-0.827699
11	9	0	2.550299	0.057400	0.891371
12	6	0	4.912835	0.333621	1.849781
13	1	0	5.396202	-1.799397	-3.010048
14	15	0	-1.412782	0.168302	0.393609
15	7	0	-2.885289	0.146920	1.084132
16	7	0	-1.354289	1.086742	-0.942503
17	7	0	-0.880600	-1.334912	0.076463
18	15	0	-1.015694	2.586263	-1.319974
19	15	0	-0.675621	-2.245783	-1.198463
20	15	0	-4.328229	-0.461515	1.016341
21	7	0	-2.027428	-2.858741	-2.006061
22	7	0	0.126491	-3.636988	-0.684958
23	7	0	0.102637	-1.432200	-2.453498
24	7	0	-2.406407	3.546850	-1.176871
25	7	0	-0.488174	2.577854	-2.914054
26	7	0	0.222459	3.421455	-0.550025
27	6	0	-0.245352	0.565293	2.981143
28	6	0	0.911451	1.440386	3.501944
29	1	0	1.112571	1.195655	4.553703
30	1	0	1.824479	1.268791	2.920257
31	1	0	0.648753	2.504650	3.433915
32	6	0	0.122249	-0.922599	3.158203
33	1	0	1.026313	-1.154075	2.583913
34	1	0	0.303937	-1.139430	4.221061
35	1	0	-0.691669	-1.564938	2.800311
36	6	0	-1.518715	0.900947	3.775660
37	1	0	-1.329360	0.728409	4.844816
38	1	0	-1.786981	1.955999	3.628107
39	1	0	-2.360112	0.282882	3.452417
40	6	0	1.606350	2.923534	-0.643862
41	1	0	2.286649	3.780100	-0.761548
42	1	0	1.912126	2.378267	0.258559
43	1	0	1.713577	2.262247	-1.507740
44	6	0	-0.034536	4.196392	0.671394
45	1	0	-0.032560	3.549614	1.561432
46	1	0	0.758333	4.951008	0.772345
47	1	0	-0.996442	4.713140	0.603081
48	6	0	-2.442085	4.881819	-1.781706
49	1	0	-2.029952	5.659163	-1.112705
50	1	0	-1.882895	4.893730	-2.721347
51	1	0	-3.489033	5.138047	-2.002511
52	6	0	-3.307943	3.403495	-0.027087
53	1	0	-3.248370	2.392790	0.383485
54	1	0	-3.072923	4.128125	0.772869
55	1	0	-4.339948	3.587000	-0.363774
56	6	0	-0.982807	1.567479	-3.855067
57	1	0	-1.883129	1.912434	-4.393773
58	1	0	-0.192891	1.366769	-4.593842
59	1	0	-1.195240	0.639197	-3.317782
60	6	0	0.208982	3.698590	-3.551229
61	1	0	1.099563	3.318952	-4.076457
62	1	0	-0.437404	4.204955	-4.289372
63	1	0	0.527897	4.423975	-2.797417
64	6	0	0.817506	-4.437658	-1.703104
65	1	0	0.868534	-5.481367	-1.359261
66	1	0	0.264551	-4.417829	-2.649078
67	1	0	1.844217	-4.073998	-1.879490
68	6	0	0.716470	-3.750585	0.654719
69	1	0	1.774561	-3.446475	0.647398
70	1	0	0.173077	-3.112769	1.355092
71	1	0	0.648629	-4.800585	0.980364
72	6	0	1.241875	-0.556888	-2.125703
73	1	0	2.199519	-1.074748	-2.278303
74	1	0	1.202845	0.331093	-2.771372
75	1	0	1.188674	-0.229632	-1.088594
76	6	0	0.167394	-1.938107	-3.828013
77	1	0	0.130253	-1.082878	-4.520047
78	1	0	1.107960	-2.485935	-4.010044
79	1	0	-0.680720	-2.597871	-4.034603
80	6	0	-2.630154	-4.162658	-1.734967
81	1	0	-3.537033	-4.050718	-1.118065
82	1	0	-2.918896	-4.636630	-2.687450
83	1	0	-1.919195	-4.809210	-1.213295
84	6	0	-2.984090	-1.893687	-2.556620
85	1	0	-3.854521	-1.805841	-1.887747
86	1	0	-2.520449	-0.907426	-2.653779
87	1	0	-3.325307	-2.227255	-3.549974
88	6	0	-6.755375	0.245022	-0.152018
89	1	0	-7.178276	-0.355747	-0.975412
90	1	0	-7.123933	-0.138682	0.803105
91	1	0	-7.099849	1.285267	-0.274682
92	6	0	-3.310445	-2.950179	1.568670
93	1	0	-2.378054	-2.387087	1.641542
94	1	0	-3.651560	-3.253085	2.573574
95	1	0	-3.112497	-3.857993	0.983654
96	6	0	-5.348868	1.329711	2.742006
97	1	0	-4.441413	1.692847	3.252324
98	1	0	-5.502337	1.920172	1.831752
99	1	0	-6.215159	1.470273	3.404358
100	6	0	-4.728131	0.735167	-1.433942
101	1	0	-5.107756	1.755975	-1.595980
102	1	0	-3.637575	0.780879	-1.386546
103	1	0	-5.030479	0.111229	-2.290770
104	7	0	-5.289827	0.208642	-0.186697
105	6	0	-5.579140	-2.857694	0.613352
106	1	0	-6.209339	-2.288613	-0.077127
107	1	0	-5.350449	-3.827256	0.148319
108	1	0	-6.145906	-3.046709	1.543140
109	7	0	-4.318705	-2.147916	0.856052
110	6	0	-5.122159	-0.963910	3.579141
111	1	0	-5.942181	-0.720908	4.269230
112	1	0	-5.220032	-2.014561	3.291603
113	1	0	-4.166001	-0.822905	4.112818
114	7	0	-5.245969	-0.099278	2.397947
115	7	0	-0.418227	0.939491	1.537943
116	1	0	0.503856	1.027148	1.113717
117	6	0	6.270136	0.109385	1.340862
118	6	0	6.930279	1.064310	0.553409
119	6	0	6.906854	-1.125472	1.524129
120	6	0	8.156905	0.793584	-0.028119
121	1	0	6.469412	2.032895	0.396617
122	6	0	8.134498	-1.395241	0.937707
123	1	0	6.434350	-1.891468	2.125935
124	6	0	8.767578	-0.441755	0.152884
125	1	0	8.639856	1.553473	-0.632601
126	1	0	8.598481	-2.362935	1.094291
127	1	0	9.727031	-0.653643	-0.304170
128	6	0	4.447028	-0.515789	3.015932
129	1	0	4.317447	-1.559306	2.712923
130	1	0	5.165716	-0.507124	3.843154
131	1	0	3.488015	-0.165027	3.394510
132	6	0	4.517493	1.692704	1.932553
133	7	0	4.147425	2.791607	1.934806

TS-1-b

Sum of electronic and thermal Free Energies= -3686.474646

Center Number	Atomic Number	Atomic Type	Coordinates (Angstroms) X	Y	Z
1	6	0	-4.612143	2.917210	0.985468
2	6	0	-4.820259	2.216707	2.172711
3	6	0	-4.496633	0.860311	2.195115
4	6	0	-3.997863	0.215415	1.083506
5	6	0	-3.897040	0.897604	-0.151317
6	6	0	-4.126881	2.295072	-0.147711
7	1	0	-4.819386	3.981965	0.944768
8	1	0	-4.602531	0.297413	3.116751
9	1	0	-3.694706	-0.819681	1.132051
10	1	0	-3.954381	2.858449	-1.056853
11	9	0	-2.772132	0.507544	-0.945392
12	6	0	-5.189089	-0.025938	-1.540023
13	6	0	-6.445059	0.334665	-0.973828
14	7	0	-7.440465	0.671686	-0.491656
15	1	0	-5.203748	2.714241	3.054134
16	15	0	1.164490	0.036303	-0.372877
17	7	0	2.481305	-0.809408	-0.829612
18	7	0	1.564464	1.481279	0.260271
19	7	0	0.206428	-0.782979	0.636950
20	15	0	1.706747	2.967372	-0.260922
21	15	0	-0.223534	-0.769601	2.159061
22	15	0	3.644795	-1.717353	-0.294759
23	7	0	0.902118	-1.325771	3.297204
24	7	0	-1.549562	-1.783514	2.276156
25	7	0	-0.554307	0.759622	2.746833
26	7	0	3.263070	3.228852	-0.875466
27	7	0	1.461389	3.980054	1.058606
28	7	0	0.626503	3.599239	-1.391467
29	6	0	0.223075	-0.577067	-3.010311

30	6	0	-0.948902	-0.043803	-3.855818
31	1	0	-1.074797	-0.666568	-4.751364
32	1	0	-1.876693	-0.073150	-3.272356
33	1	0	-0.762138	0.992316	-4.169104
34	6	0	-0.057638	-2.045921	-2.631061
35	1	0	-0.970826	-2.116966	-2.027695
36	1	0	-0.192533	-2.648620	-3.541371
37	1	0	0.784395	-2.455226	-2.063234
38	6	0	1.524443	-0.469170	-3.823064
39	1	0	1.444009	-1.086126	-4.729669
40	1	0	1.698064	0.574416	-4.119444
41	1	0	2.372656	-0.812438	-3.224883
42	6	0	-0.813841	3.588479	-1.078688
43	1	0	-1.300430	4.386799	-1.657649
44	1	0	-1.289314	2.630624	-1.333380
45	1	0	-0.971363	3.782778	-0.014688
46	6	0	0.903029	3.450171	-2.827538
47	1	0	0.575120	2.466520	-3.193121
48	1	0	0.362473	4.242480	-3.366021
49	1	0	1.972817	3.566483	-3.025771
50	6	0	3.737507	4.592368	-1.128219
51	1	0	3.495867	4.937246	-2.150127
52	1	0	3.301016	5.291752	-0.409426
53	1	0	4.831081	4.613197	-1.008127
54	6	0	3.948671	2.209655	-1.678208
55	1	0	3.566003	1.213812	-1.442007
56	1	0	3.826228	2.390960	-2.761243
57	1	0	5.024166	2.240654	-1.443881
58	6	0	1.920546	3.569415	2.388920
59	1	0	2.914850	3.989372	2.621839
60	1	0	1.202462	3.934357	3.138639
61	1	0	1.954988	2.478131	2.446779
62	6	0	1.136467	5.405331	0.961556
63	1	0	0.295328	5.631514	1.635953
64	1	0	1.995258	6.031856	1.260553
65	1	0	0.847943	5.658611	-0.062324
66	6	0	-2.397196	-1.774427	3.465728
67	1	0	-2.166119	-2.615839	4.144151
68	1	0	-2.281967	-0.834518	4.014181
69	1	0	-3.448210	-1.855860	3.152935
70	6	0	-1.809662	-2.884072	1.342549
71	1	0	-2.870074	-2.876744	1.055375
72	1	0	-1.209400	-2.751393	0.439582
73	1	0	-1.574014	-3.862272	1.800840
74	6	0	-1.188578	1.770784	1.886002
75	1	0	-2.187397	2.021556	2.266043
76	1	0	-0.553942	2.665789	1.859578
77	1	0	-1.304714	1.391012	0.871760
78	6	0	-0.537369	1.140089	4.160207
79	1	0	0.047194	2.066235	4.282618
80	1	0	-1.562441	1.332147	4.520946
81	1	0	-0.082236	0.350261	4.764897
82	6	0	0.944383	-2.710065	3.762421
83	1	0	1.732966	-3.279095	3.241795
84	1	0	1.166073	-2.728462	4.841897
85	1	0	-0.018221	-3.202181	3.594868
86	6	0	2.205353	-0.657181	3.350353
87	1	0	2.948502	-1.213180	2.755918
88	1	0	2.122938	0.357287	2.946546
89	1	0	2.550952	-0.595899	4.394804
90	6	0	6.230460	-1.359842	0.674926
91	1	0	6.469036	-1.524464	1.739344
92	1	0	6.376258	-2.292046	0.122527
93	1	0	6.926965	-0.601167	0.280439
94	6	0	1.791926	-3.637257	0.378021
95	1	0	1.078652	-2.897049	0.014442
96	1	0	1.892935	-4.459188	-0.351321
97	1	0	1.395870	-4.058196	1.311321
98	6	0	5.211691	-1.508979	-2.486465
99	1	0	4.516689	-1.130716	-3.254708
100	1	0	5.631896	-0.657091	-1.941357
101	1	0	6.030016	-2.048734	-2.984585
102	6	0	4.602378	0.389171	1.206461
103	1	0	5.321245	1.140107	0.840629
104	1	0	3.593090	0.755369	1.004372
105	1	0	4.738166	0.278251	2.294639
106	7	0	4.851227	-0.884325	0.524992
107	6	0	4.043275	-3.862234	1.346578
108	1	0	4.889194	-3.281053	1.725766
109	1	0	3.543969	-4.336639	2.203535
110	1	0	4.424309	-4.658660	0.682058
111	7	0	3.077594	-2.984912	0.675569
112	6	0	3.980481	-3.608623	-2.215838
113	1	0	4.765145	-4.060243	-2.838462
114	1	0	3.667368	-4.349229	-1.474267
115	1	0	3.121528	-3.358045	-2.862629
116	7	0	4.545565	-2.427767	-1.547226
117	7	0	0.301129	0.324983	-1.814337
118	1	0	-0.644989	0.573264	-1.527241
119	6	0	-4.944692	0.685952	-2.857997
120	1	0	-5.553517	0.277524	-3.672896
121	1	0	-5.192589	1.745449	-2.761767
122	1	0	-3.898552	0.622883	-3.155986
123	6	0	-4.875728	-1.454386	-1.437397
124	6	0	-3.961272	-2.076908	-2.302010
125	6	0	-5.423255	-2.247823	-0.414114
126	6	0	-3.604893	-3.405823	-2.143558
127	1	0	-3.516817	-1.513082	-3.111113
128	6	0	-5.068786	-3.579509	-0.262614
129	1	0	-6.147874	-1.810976	0.263039
130	6	0	-4.152927	-4.172394	-1.121635
131	1	0	-2.892782	-3.848588	-2.831048
132	1	0	-5.516717	-4.159892	0.536747
133	1	0	-3.877619	-5.213667	-1.003319

TS-1-c

Sum of electronic and thermal Free Energies= -3686.480644

Center Number	Atomic Number	Atomic Type	Coordinates (Angstroms) X	Y	Z
1	6	0	-4.560643	2.940414	1.026621
2	6	0	-4.868846	2.262206	2.207937
3	6	0	-4.604675	0.895017	2.263245
4	6	0	-4.072037	0.216322	1.185679
5	6	0	-3.855461	0.881859	-0.042690
6	6	0	-4.031575	2.287520	-0.066778
7	1	0	-4.721827	4.012070	0.963840
8	1	0	-4.786240	0.347121	3.182658
9	1	0	-3.818244	-0.830165	1.259630
10	1	0	-3.790660	2.826385	-0.975483
11	9	0	-2.717136	0.427950	-0.761180
12	6	0	-5.171711	0.020361	-1.438765
13	6	0	-6.497918	0.531112	-0.915388
14	1	0	-6.598264	1.598120	-1.117463
15	1	0	-7.352948	0.013343	-1.362925
16	1	0	-6.546939	0.403299	0.169898
17	6	0	-4.764529	0.650517	-2.642015
18	7	0	-4.405500	1.233117	-3.577556
19	1	0	-5.273721	2.788150	3.063075
20	15	0	1.127378	0.044131	-0.360748
21	7	0	2.432981	-0.796916	-0.856035
22	7	0	1.543032	1.463762	0.318014
23	7	0	0.184680	-0.803070	0.642721
24	15	0	1.690001	2.965532	-0.153939
25	15	0	-0.241489	-0.825666	2.163563
26	15	0	3.584386	-1.743285	-0.366529
27	7	0	0.875168	-1.442040	3.283089
28	7	0	-1.591877	-1.812042	2.257978
29	7	0	-0.536956	0.690475	2.807884
30	7	0	3.250120	3.240597	-0.753904
31	7	0	1.443515	3.933662	1.199263
32	7	0	0.616947	3.639555	-1.265884
33	6	0	0.150207	-0.469297	-3.010014
34	6	0	-1.008100	0.125246	-3.832513
35	1	0	-1.162154	-0.473598	-4.740076
36	1	0	-1.940924	0.133655	-3.257094
37	1	0	-0.781992	1.157835	-4.130989
38	6	0	-0.165165	-1.941429	-2.674433
39	1	0	-1.068929	-2.007035	-2.057184
40	1	0	-0.331856	-2.509791	-3.601412
41	1	0	0.674184	-2.392657	-2.134620
42	6	0	1.451280	-0.372043	-3.824981
43	1	0	1.343380	-0.947638	-4.755637
44	1	0	1.657500	0.676374	-4.080751
45	1	0	2.292266	-0.767612	-3.249208
46	6	0	-0.824783	3.620274	-0.960543
47	1	0	-1.305446	4.451679	-1.496150
48	1	0	-1.302447	2.681789	-1.274707
49	1	0	-0.987048	3.752836	0.112255
50	6	0	0.898405	3.535640	-2.705762
51	1	0	0.578186	2.561358	-3.101225

52	1	0	0.352073	4.338926	-3.221162
53	1	0	1.967685	3.667132	-2.897213
54	6	0	3.731153	4.609444	-0.960624
55	1	0	3.490194	4.990702	-1.969673
56	1	0	3.299014	5.285841	-0.217620
57	1	0	4.824980	4.620604	-0.841214
58	6	0	3.929385	2.245843	-1.592148
59	1	0	3.540397	1.244834	-1.390408
60	1	0	3.807522	2.465184	-2.668123
61	1	0	5.005161	2.262117	-1.357405
62	6	0	1.898682	3.474185	2.514914
63	1	0	2.903417	3.863753	2.756415
64	1	0	1.192161	3.834474	3.277759
65	1	0	1.906363	2.380921	2.540390
66	6	0	1.131412	5.364051	1.148558
67	1	0	0.291662	5.575445	1.829574
68	1	0	1.994985	5.973892	1.468010
69	1	0	0.845429	5.652397	0.133221
70	6	0	-2.401448	-1.852926	3.472793
71	1	0	-2.165420	-2.733346	4.098163
72	1	0	-2.251526	-0.946922	4.068138
73	1	0	-3.463555	-1.900707	3.191242
74	6	0	-1.893650	-2.862784	1.280443
75	1	0	-2.962639	-2.833838	1.029157
76	1	0	-1.322913	-2.689193	0.365638
77	1	0	-1.652931	-3.864913	1.681090
78	6	0	-1.197639	1.722611	1.993317
79	1	0	-2.209571	1.920775	2.371240
80	1	0	-0.595114	2.639635	2.022265
81	1	0	-1.283748	1.394407	0.958837
82	6	0	-0.514248	1.018173	4.234463
83	1	0	0.062893	1.944461	4.385989
84	1	0	-1.538100	1.188493	4.610634
85	1	0	-0.048157	0.210457	4.806044
86	6	0	0.914903	-2.858246	3.644653
87	1	0	1.683586	-3.394503	3.063463
88	1	0	1.164222	-2.955250	4.713587
89	1	0	-0.057012	-3.328574	3.468188
90	6	0	2.193267	-0.801422	3.342713
91	1	0	2.906382	-1.323240	2.683975
92	1	0	2.118575	0.242785	3.022054
93	1	0	2.571594	-0.828645	4.376967
94	6	0	6.174898	-1.461499	0.616131
95	1	0	6.416808	-1.670297	1.672023
96	1	0	6.307119	-2.372810	0.026718
97	1	0	6.878708	-0.696344	0.247600
98	6	0	1.708019	-3.669588	0.218359
99	1	0	0.998128	-2.900811	-0.088326
100	1	0	1.791966	-4.444126	-0.563154
101	1	0	1.318079	-4.145714	1.127604
102	6	0	5.148964	-1.445541	-2.546795
103	1	0	4.453448	-1.018363	-3.288429
104	1	0	5.583891	-0.628894	-1.960736
105	1	0	5.956812	-1.968761	-3.078485
106	6	0	4.574056	0.290740	1.213639
107	1	0	5.297532	1.044536	0.862677
108	1	0	3.566845	0.674412	1.034874
109	1	0	4.720514	0.145221	2.296318
110	7	0	4.800716	-0.964946	0.492506
111	6	0	3.961400	-3.974447	1.160304
112	1	0	4.817728	-3.425381	1.563342
113	1	0	3.461218	-4.486506	1.994878
114	1	0	4.327112	-4.740024	0.452301
115	7	0	3.003643	-3.050191	0.543140
116	6	0	3.897400	-3.543159	-2.378494
117	1	0	4.678273	-3.976371	-3.018836
118	1	0	3.569848	-4.313388	-1.674268
119	1	0	3.045632	-3.248821	-3.016220
120	7	0	4.477021	-2.404099	-1.652095
121	7	0	0.257751	0.388536	-1.782679
122	1	0	-0.681259	0.649192	-1.484851
123	6	0	-4.905382	-1.417224	-1.345740
124	6	0	-5.564069	-2.208701	-0.392601
125	6	0	-3.935042	-2.052112	-2.140177
126	6	0	-5.261379	-3.556692	-0.239041
127	1	0	-6.329172	-1.769948	0.234549
128	6	0	-3.635875	-3.391746	-1.983847
129	1	0	-3.407207	-1.475401	-2.889926
130	6	0	-4.295949	-4.160870	-1.028471
131	1	0	-5.792496	-4.137427	0.507423
132	1	0	-2.878902	-3.843295	-2.615244
133	1	0	-4.062561	-5.212197	-0.908884

TS-1-d

Sum of electronic and thermal Free Energies= -3686.475685

Center Number	Atomic Number	Atomic Type	Coordinates (Angstroms) X	Y	Z
1	6	0	-4.931054	1.272558	1.722308
2	6	0	-4.690342	0.015573	2.272022
3	6	0	-3.892568	-0.872896	1.546026
4	6	0	-3.384175	-0.542021	0.313335
5	6	0	-3.738760	0.681827	-0.301422
6	6	0	-4.430101	1.633525	0.485444
7	1	0	-5.506177	2.003135	2.282423
8	1	0	-3.637489	-1.838365	1.969002
9	1	0	-2.743621	-1.220080	-0.230415
10	1	0	-4.604125	2.626502	0.088403
11	9	0	-2.698877	1.245341	-1.107688
12	6	0	-4.892248	0.255325	-1.988060
13	6	0	-5.001406	1.564462	-2.746777
14	1	0	-4.081975	1.773709	-3.292424
15	1	0	-5.831312	1.556102	-3.462209
16	1	0	-5.172540	2.399643	-2.060791
17	6	0	-4.084480	-0.719859	-2.625702
18	7	0	-3.381477	-1.527014	-3.070542
19	1	0	-5.084997	-0.253940	3.243231
20	15	0	1.311037	0.083592	-0.343511
21	7	0	2.629769	-0.593123	-1.010177
22	7	0	1.705274	1.305054	0.662393
23	7	0	0.379751	-0.995288	0.428533
24	15	0	1.851061	2.877733	0.563330
25	15	0	-0.015747	-1.396512	1.905868
26	15	0	3.843208	-1.565999	-0.832922
27	7	0	1.155228	-1.839205	3.055064
28	7	0	-0.855964	-2.853860	1.767495
29	7	0	-0.815724	-0.163248	2.706342
30	7	0	3.436349	3.289817	0.136119
31	7	0	1.517283	3.516558	2.083603
32	7	0	0.835793	3.793296	-0.420248
33	6	0	0.422870	0.215101	-3.068677
34	6	0	-0.749644	0.929265	-3.768378
35	1	0	-0.785505	0.619685	-4.821435
36	1	0	-1.699961	0.660738	-3.294552
37	1	0	-0.618288	2.019606	-3.724905
38	6	0	0.205663	-1.309637	-3.156311
39	1	0	-0.754963	-1.584334	-2.704055
40	1	0	0.183965	-1.619858	-4.211594
41	1	0	1.022052	-1.837946	-2.652841
42	6	0	1.737861	0.611024	-3.762519
43	1	0	1.699980	0.299582	-4.816239
44	1	0	1.878011	1.699648	-3.721688
45	1	0	2.585845	0.127080	-3.273149
46	6	0	-0.618721	3.756375	-0.187660
47	1	0	-1.043541	4.729337	-0.477685
48	1	0	-1.103577	2.967690	-0.776725
49	1	0	-0.828414	3.584138	0.871983
50	6	0	1.177541	4.000537	-1.833560
51	1	0	0.801796	3.171941	-2.450514
52	1	0	0.720715	4.944399	-2.167293
53	1	0	2.261958	4.076781	-1.959898
54	6	0	3.882890	4.681363	0.242485
55	1	0	3.699445	5.251024	-0.686640
56	1	0	3.373977	5.186141	1.069127
57	1	0	4.964341	4.691767	0.445487
58	6	0	4.207806	2.490912	-0.821579
59	1	0	3.840296	1.462818	-0.841918
60	1	0	4.152339	2.909036	-1.843214
61	1	0	5.263626	2.485586	-0.508968
62	6	0	1.958756	2.800982	3.282239
63	1	0	2.841166	3.290799	3.730726
64	1	0	1.146048	2.797026	4.026060
65	1	0	2.204345	1.767503	3.030153
66	6	0	1.145565	4.908985	2.343084
67	1	0	0.267744	4.934452	3.008617
68	1	0	1.969443	5.451254	2.840473
69	1	0	0.896465	5.418304	1.408452
70	6	0	-1.377489	-3.546319	2.947115
71	1	0	-1.290823	-4.633167	2.790344
72	1	0	-0.796239	-3.280858	3.835766
73	1	0	-2.439932	-3.307364	3.125610

74	6	0	-1.430508	-3.340707	0.509529
75	1	0	-2.528284	-3.253530	0.515258
76	1	0	-1.032404	-2.768814	-0.331391
77	1	0	-1.165009	-4.403503	0.383780
78	6	0	-1.250742	1.078574	2.055525
79	1	0	-2.323479	1.224869	2.229596
80	1	0	-0.674224	1.928810	2.453627
81	1	0	-1.096812	1.022783	0.979963
82	6	0	-1.346871	-0.318261	4.063209
83	1	0	-1.052053	0.551490	4.674623
84	1	0	-2.446422	-0.370628	4.034089
85	1	0	-0.945637	-1.218447	4.538459
86	6	0	1.941459	-3.066104	2.864433
87	1	0	2.827365	-2.887965	2.233441
88	1	0	2.268107	-3.431390	3.850105
89	1	0	1.324169	-3.834811	2.390945
90	6	0	1.880942	-0.826279	3.827584
91	1	0	2.804630	-0.510578	3.317522
92	1	0	1.251864	0.050936	3.988990
93	1	0	2.152955	-1.254983	4.804877
94	6	0	6.455151	-1.389257	0.139883
95	1	0	6.692856	-1.992012	1.034010
96	1	0	6.655908	-1.982602	-0.756345
97	1	0	7.112520	-0.504871	0.139848
98	6	0	2.048222	-3.650915	-0.610002
99	1	0	1.314128	-2.851663	-0.718840
100	1	0	2.085015	-4.261619	-1.529236
101	1	0	1.727510	-4.294565	0.220479
102	6	0	5.369372	-0.673789	-2.899900
103	1	0	4.676128	-0.109134	-3.544650
104	1	0	5.765949	-0.000504	-2.133508
105	1	0	6.203139	-1.040398	-3.516593
106	6	0	4.700003	-0.206657	1.370106
107	1	0	5.403755	0.631010	1.493179
108	1	0	3.691721	0.210902	1.290462
109	1	0	4.766268	-0.850440	2.264282
110	7	0	5.055783	-0.950043	0.158822
111	6	0	4.375760	-4.103132	0.025334
112	1	0	5.286839	-3.640924	0.415760
113	1	0	3.976383	-4.778927	0.795884
114	1	0	4.635074	-4.704351	-0.864540
115	7	0	3.362635	-3.086024	-0.271961
116	6	0	4.122896	-2.750535	-3.250106
117	1	0	4.906478	-3.041739	-3.963632
118	1	0	3.758146	-3.655025	-2.753810
119	1	0	3.291675	-2.287433	-3.808771
120	7	0	4.710884	-1.829301	-2.265815
121	7	0	0.426143	0.718624	-1.651040
122	1	0	-0.535449	0.850227	-1.338863
123	6	0	-6.115796	-0.280330	-1.373722
124	6	0	-7.171724	0.571546	-1.026122
125	6	0	-6.225915	-1.628849	-1.004339
126	6	0	-8.275288	0.101422	-0.329339
127	1	0	-7.128678	1.619650	-1.294204
128	6	0	-7.328039	-2.095907	-0.309671
129	1	0	-5.430029	-2.316954	-1.264789
130	6	0	-8.359979	-1.233070	0.040910
131	1	0	-9.074031	0.787681	-0.070076
132	1	0	-7.381076	-3.143790	-0.035366
133	1	0	-9.222107	-1.599247	0.585992

TS-1-e

Sum of electronic and thermal Free Energies= -3686.472360

Center Number	Atomic Number	Atomic Type	Coordinates (Angstroms) X	Y	Z
1	6	0	4.591004	1.941096	1.809293
2	6	0	4.568113	3.285013	1.440055
3	6	0	4.165360	3.598594	0.142734
4	6	0	3.809104	2.617789	-0.761569
5	6	0	3.900966	1.250343	-0.408197
6	6	0	4.220394	0.940617	0.934665
7	1	0	4.873933	1.665881	2.820532
8	1	0	4.113826	4.636709	-0.170168
9	1	0	3.486590	2.878785	-1.762044
10	1	0	4.220718	-0.092709	1.251135
11	9	0	2.861249	0.455324	-0.981670
12	6	0	5.316205	0.282641	-1.664011
13	6	0	4.900933	0.645551	-3.077178
14	1	0	4.975467	1.725110	-3.223837
15	1	0	5.530302	0.165453	-3.835548
16	1	0	3.865979	0.360465	-3.270239
17	6	0	6.492616	0.971557	-1.257068
18	7	0	7.416220	1.572704	-0.906257
19	1	0	4.848950	4.061345	2.139897
20	15	0	-1.198740	-0.206254	-0.346796
21	7	0	-2.652253	-0.637890	-0.939341
22	7	0	-0.949388	-0.742348	1.161118
23	7	0	-0.959222	1.399747	-0.434604
24	15	0	-0.312432	-2.006785	1.871155
25	15	0	-0.980185	2.622615	0.568824
26	15	0	-4.193753	-0.352693	-0.909962
27	7	0	-2.444605	3.148061	1.219951
28	7	0	-0.440993	3.967422	-0.299348
29	7	0	-0.095803	2.304307	1.969058
30	7	0	-1.479435	-3.228899	2.028178
31	7	0	0.191043	-1.501634	3.391210
32	7	0	1.071345	-2.747094	1.272939
33	6	0	-0.142199	-1.114893	-2.840742
34	6	0	1.268523	-1.531484	-3.290089
35	1	0	1.291420	-1.653240	-4.381129
36	1	0	1.999591	-0.767162	-3.000871
37	1	0	1.559205	-2.479786	-2.819842
38	6	0	-0.517643	0.216191	-3.523424
39	1	0	0.180220	1.006322	-3.219257
40	1	0	-0.473788	0.100827	-4.616217
41	1	0	-1.532906	0.516675	-3.242726
42	6	0	-1.144539	-2.215538	-3.230701
43	1	0	-1.140580	-2.346768	-4.322503
44	1	0	-0.860319	-3.165742	-2.758012
45	1	0	-2.151542	-1.946126	-2.901474
46	6	0	2.319035	-1.967140	1.166161
47	1	0	3.172347	-2.627543	1.370463
48	1	0	2.455759	-1.552425	0.161031
49	1	0	2.312275	-1.145290	1.887361
50	6	0	0.970565	-3.807323	0.260018
51	1	0	0.828984	-3.383363	-0.745849
52	1	0	1.905521	-4.383763	0.277782
53	1	0	0.138652	-4.480215	0.489950
54	6	0	-1.236370	-4.359185	2.929493
55	1	0	-0.675565	-5.175809	2.439144
56	1	0	-0.681953	-4.033555	3.814104
57	1	0	-2.206638	-4.758687	3.260608
58	6	0	-2.394308	-3.549004	0.926383
59	1	0	-2.539570	-2.678368	0.282886
60	1	0	-2.020988	-4.387922	0.312121
61	1	0	-3.368147	-3.840660	1.349379
62	6	0	-0.515716	-0.419626	4.084622
63	1	0	-1.310041	-0.807057	4.747072
64	1	0	0.211093	0.130235	4.700870
65	1	0	-0.938029	0.272633	3.350800
66	6	0	1.106199	-2.261707	4.247086
67	1	0	1.890314	-1.587464	4.626246
68	1	0	0.576492	-2.696225	5.113002
69	1	0	1.579370	-3.065311	3.675893
70	6	0	0.029625	5.115730	0.489068
71	1	0	-0.048938	6.022707	-0.127570
72	1	0	-0.595722	5.251254	1.378989
73	1	0	1.080205	4.993589	0.803113
74	6	0	0.280534	3.803279	-1.572343
75	1	0	1.354219	3.618433	-1.407174
76	1	0	-0.144159	2.964156	-2.129362
77	1	0	0.160335	4.729106	-2.155165
78	6	0	1.217576	1.651241	1.820384
79	1	0	2.027426	2.393728	1.810884
80	1	0	1.370179	0.961679	2.661463
81	1	0	1.264536	1.075900	0.896136
82	6	0	-0.162306	3.143800	3.169912
83	1	0	-0.029947	2.502382	4.054497
84	1	0	0.637727	3.903768	3.174187
85	1	0	-1.133289	3.643433	3.237224
86	6	0	-3.281522	4.201463	0.647732
87	1	0	-4.172095	3.765186	0.166608
88	1	0	-3.619988	4.878766	1.449233
89	1	0	-2.717063	4.774495	-0.092995
90	6	0	-3.195912	2.215431	2.065682
91	1	0	-4.029143	1.778804	1.493288
92	1	0	-2.546487	1.405945	2.412080
93	1	0	-3.600113	2.746445	2.942745
94	6	0	-6.392459	-1.188537	0.581049
95	1	0	-6.909837	-0.489600	1.260111

96	1	0	-6.856701	-1.143749	-0.407779
97	1	0	-6.510469	-2.208308	0.983725
98	6	0	-3.721240	2.073529	-2.113087
99	1	0	-2.670679	1.807622	-1.981294
100	1	0	-4.016979	1.947395	-3.168776
101	1	0	-3.839037	3.132791	-1.850801
102	6	0	-4.925291	-2.714842	-1.991280
103	1	0	-3.980350	-3.078548	-2.428122
104	1	0	-4.973755	-3.031548	-0.944069
105	1	0	-5.766500	-3.165992	-2.536883
106	6	0	-4.278217	-0.934573	1.785711
107	1	0	-4.428089	-1.937532	2.214930
108	1	0	-3.204063	-0.770924	1.672559
109	1	0	-4.689341	-0.190422	2.486952
110	7	0	-4.965694	-0.859951	0.493364
111	6	0	-5.948607	1.725797	-1.117457
112	1	0	-6.462902	1.233722	-0.286342
113	1	0	-5.957767	2.809256	-0.931391
114	1	0	-6.507529	1.529560	-2.050275
115	7	0	-4.552514	1.279389	-1.192409
116	6	0	-5.026346	-0.760482	-3.464594
117	1	0	-5.782144	-1.318867	-4.034129
118	1	0	-5.292391	0.300017	-3.495514
119	1	0	-4.046082	-0.902451	-3.952228
120	7	0	-5.041624	-1.248229	-2.078042
121	7	0	-0.078001	-1.003329	-1.346485
122	1	0	0.851801	-0.697300	-1.063069
123	6	0	5.271322	-1.124233	-1.255343
124	6	0	4.509178	-2.072507	-1.955928
125	6	0	5.935257	-1.571983	-0.099525
126	6	0	4.391559	-3.378381	-1.511453
127	1	0	3.994879	-1.783543	-2.861503
128	6	0	5.816602	-2.880546	0.341351
129	1	0	6.561420	-0.880233	0.451901
130	6	0	5.038444	-3.796784	-0.354358
131	1	0	3.790292	-4.079719	-2.079854
132	1	0	6.344192	-3.188015	1.237714
133	1	0	4.950618	-4.820953	-0.011541

Int-3

Sum of electronic and thermal Free Energies= -3686.553308

Center Number	Atomic Number	Atomic Type	Coordinates (Angstroms) X	Y	Z
1	6	0	-4.604860	-1.808248	-2.492673
2	6	0	-4.076557	-1.043302	-3.521935
3	6	0	-4.167483	0.338664	-3.462492
4	6	0	-4.785862	0.950936	-2.382487
5	6	0	-5.313306	0.190260	-1.345735
6	6	0	-5.217691	-1.195345	-1.410553
7	1	0	-4.528554	-2.888255	-2.522631
8	1	0	-3.751028	0.947296	-4.255771
9	1	0	-4.842935	2.032564	-2.346895
10	1	0	-5.608572	-1.802212	-0.602705
11	9	0	-1.179523	-0.348634	2.461855
12	6	0	-6.070510	0.845215	-0.178530
13	6	0	-7.558320	0.956430	-0.571614
14	1	0	-7.960164	-0.039164	-0.761703
15	1	0	-8.141231	1.437117	0.214998
16	1	0	-7.652469	1.541647	-1.486333
17	1	0	-3.590396	-1.521818	-4.363047
18	15	0	1.706744	-0.108365	0.670289
19	7	0	3.337463	-0.312036	0.568493
20	7	0	0.936681	-0.860838	-0.565243
21	7	0	1.412235	1.498841	0.505902
22	15	0	0.269828	-2.268504	-0.793576
23	15	0	0.358035	2.493621	-0.095214
24	15	0	4.582972	0.158741	-0.252415
25	7	0	1.077531	3.744076	-1.008871
26	7	0	-0.473485	3.287590	1.133790
27	7	0	-0.740504	1.835082	-1.210574
28	7	0	1.422014	-3.520910	-0.827782
29	7	0	-0.592166	-2.113391	-2.253491
30	7	0	-0.890847	-2.945151	0.216919
31	6	0	1.993585	-0.905825	3.342841
32	6	0	0.996192	-1.264108	4.464386
33	1	0	1.534341	-1.380960	5.416199
34	1	0	0.234163	-0.482236	4.560666
35	1	0	0.483788	-2.206004	4.226593
36	6	0	2.752974	0.378575	3.751731
37	1	0	2.046111	1.210656	3.873060
38	1	0	3.281625	0.224364	4.705423
39	1	0	3.485888	0.644269	2.982061
40	6	0	2.999127	-2.070851	3.195295
41	1	0	3.544465	-2.227842	4.139546
42	1	0	2.458060	-2.993348	2.941781
43	1	0	3.713514	-1.849361	2.396083
44	6	0	-2.263890	-2.406135	0.236846
45	1	0	-2.949156	-3.224227	0.511564
46	1	0	-2.341819	-1.603129	0.979545
47	1	0	-2.540119	-2.032871	-0.750880
48	6	0	-0.497237	-3.464527	1.541094
49	1	0	-0.608242	-2.676869	2.296061
50	1	0	-1.150968	-4.318145	1.785252
51	1	0	0.542659	-3.801774	1.525321
52	6	0	1.055421	-4.931845	-0.980816
53	1	0	1.743886	-5.548706	-0.380924
54	1	0	0.034741	-5.099501	-0.626048
55	1	0	1.129232	-5.259608	-2.033645
56	6	0	2.781765	-3.234387	-1.288964
57	1	0	3.044837	-2.197852	-1.077433
58	1	0	3.485088	-3.887603	-0.748938
59	1	0	2.894469	-3.430628	-2.372513
60	6	0	-0.254606	-1.054541	-3.210050
61	1	0	0.568817	-1.352377	-3.886722
62	1	0	-1.146843	-0.847555	-3.820278
63	1	0	0.028229	-0.148456	-2.666410
64	6	0	-1.115385	-3.326573	-2.883759
65	1	0	-1.975078	-3.055757	-3.512986
66	1	0	-0.362714	-3.827344	-3.521053
67	1	0	-1.456578	-4.030901	-2.117497
68	6	0	-1.699168	4.017754	0.793842
69	1	0	-1.812569	4.870530	1.480764
70	1	0	-1.637773	4.410871	-0.228058
71	1	0	-2.598268	3.386662	0.873974
72	6	0	-0.403699	2.811383	2.524026
73	1	0	-1.135010	2.013920	2.720912
74	1	0	0.593049	2.402663	2.714316
75	1	0	-0.583545	3.668673	3.191727
76	6	0	-1.696282	0.867143	-0.648800
77	1	0	-2.657782	1.370888	-0.454739
78	1	0	-1.870321	0.059982	-1.369370
79	1	0	-1.328089	0.426708	0.283547
80	6	0	-1.306985	2.570242	-2.343906
81	1	0	-1.540309	1.851521	-3.144372
82	1	0	-2.245778	3.083246	-2.065024
83	1	0	-0.597494	3.312666	-2.717621
84	6	0	1.569360	4.940596	-0.315779
85	1	0	2.532232	4.763074	0.196756
86	1	0	1.716830	5.736127	-1.061448
87	1	0	0.835598	5.273053	0.424201
88	6	0	2.019000	3.263770	-2.035419
89	1	0	2.962055	2.904498	-1.588897
90	1	0	1.568872	2.438767	-2.599478
91	1	0	2.230221	4.088323	-2.732547
92	6	0	5.786315	-0.517274	-2.691468
93	1	0	5.961434	0.313970	-3.398647
94	1	0	6.662538	-0.625910	-2.046737
95	1	0	5.651572	-1.439912	-3.279441
96	6	0	4.451758	2.595988	1.024327
97	1	0	3.514355	2.197322	1.416875
98	1	0	5.228829	2.598458	1.810041
99	1	0	4.279008	3.635925	0.710911
100	6	0	5.996545	-2.075868	0.262127
101	1	0	5.373014	-2.494563	1.070150
102	1	0	5.607260	-2.427840	-0.698588
103	1	0	7.028186	-2.437501	0.381807
104	6	0	3.397148	0.005515	-2.694444
105	1	0	3.255778	-0.814676	-3.415287
106	1	0	2.500111	0.055211	-2.070572
107	1	0	3.514092	0.949212	-3.255338
108	7	0	4.586396	-0.278485	-1.884684
109	6	0	5.976858	2.417667	-0.895445
110	1	0	6.139408	1.886597	-1.838160
111	1	0	5.732981	3.465267	-1.127466
112	1	0	6.917258	2.400823	-0.314221
113	7	0	4.857068	1.827260	-0.158252
114	6	0	6.562347	-0.082401	1.550929
115	1	0	7.557192	-0.524557	1.701052
116	1	0	6.674818	1.005137	1.495711
117	1	0	5.929796	-0.335555	2.420343

118	7	0	6.011843	-0.601798	0.288670
119	7	0	1.200227	-0.739774	2.102669
120	6	0	-5.573389	2.220423	0.021395
121	7	0	-5.208525	3.298504	0.172386
122	6	0	-5.837901	0.085638	1.136336
123	6	0	-4.546109	0.021267	1.651693
124	6	0	-6.861195	-0.568046	1.809991
125	6	0	-4.270204	-0.684023	2.809379
126	1	0	-3.729884	0.515450	1.140823
127	6	0	-6.590463	-1.276862	2.976133
128	1	0	-7.877608	-0.539836	1.439707
129	6	0	-5.299379	-1.337814	3.476492
130	1	0	-3.240869	-0.721309	3.149078
131	1	0	-7.399107	-1.783141	3.490453
132	1	0	-5.093453	-1.894989	4.383008
133	1	0	0.067357	-0.553575	2.273656

18-crown-6

Sum of electronic and thermal Free Energies= -922.329277

Center Number	Atomic Number	Atomic Type	Coordinates (Angstroms) X	Y	Z
1	8	0	0.491492	2.831729	0.263744
2	6	0	1.752092	3.194244	-0.288869
3	6	0	2.830028	2.294796	0.289031
4	8	0	2.699355	0.989787	-0.263960
5	6	0	3.643785	0.079696	0.288876
6	6	0	3.403642	-1.303680	-0.288615
7	8	0	2.207700	-1.842249	0.264159
8	6	0	1.891042	-3.115011	-0.288749
9	6	0	0.572851	-3.598402	0.288812
10	8	0	-0.491492	-2.831729	-0.263745
11	6	0	-1.752091	-3.194244	0.288869
12	6	0	-2.830028	-2.294796	-0.289032
13	8	0	-2.699355	-0.989786	0.263960
14	6	0	-3.643784	-0.079696	-0.288876
15	6	0	-3.403641	1.303680	0.288615
16	8	0	-2.207699	1.842250	-0.264159
17	6	0	-1.891041	3.115011	0.288749
18	6	0	-0.572851	3.598402	-0.288812
19	1	0	1.993827	4.247644	-0.045335
20	1	0	1.737073	3.090272	-1.389779
21	1	0	3.823120	2.721499	0.045899
22	1	0	2.730121	2.261037	1.389904
23	1	0	3.546337	0.041110	1.389804
24	1	0	4.676871	0.397057	0.045148
25	1	0	3.324869	-1.234394	-1.389537
26	1	0	4.269391	-1.950631	-0.044961
27	1	0	1.808712	-3.049730	-1.389655
28	1	0	2.682117	-3.851426	-0.045279
29	1	0	0.445238	-4.671575	0.044964
30	1	0	0.593548	-3.495780	1.389751
31	1	0	-1.737073	-3.090272	1.389779
32	1	0	-1.993827	-4.247644	0.045335
33	1	0	-2.730121	-2.261037	-1.389905
34	1	0	-3.823120	-2.721499	-0.045899
35	1	0	-3.546337	-0.041110	-1.389804
36	1	0	-4.676871	-0.397057	-0.045148
37	1	0	-4.269391	1.950631	0.044961
38	1	0	-3.324869	1.234394	1.389537
39	1	0	-2.682117	3.851426	0.045279
40	1	0	-1.808712	3.049730	1.389655
41	1	0	-0.445238	4.671575	-0.044964
42	1	0	-0.593548	3.495781	-1.389751

KO-*t*-Bu

Sum of electronic and thermal Free Energies= -832.938017

Center Number	Atomic Number	Atomic Type	Coordinates (Angstroms) X	Y	Z
1	6	0	0.917498	0.000007	0.143624
2	6	0	1.047016	-1.261615	1.051609
3	1	0	0.215981	-1.272242	1.774160
4	1	0	0.971217	-2.164830	0.426119
5	1	0	1.999483	-1.295508	1.607729
6	6	0	2.117360	0.000068	-0.853132
7	1	0	2.052681	-0.892058	-1.495493
8	1	0	2.052710	0.892425	-1.495208
9	1	0	3.097345	-0.000065	-0.345967
10	6	0	1.047639	1.261394	1.051950
11	1	0	0.972338	2.164909	0.426800
12	1	0	0.216772	1.272347	1.774699
13	1	0	2.000220	1.294613	1.607922
14	8	0	-0.278974	0.000203	-0.527813
15	19	0	-2.286806	0.000527	-1.638971

HO-*t*-Bu

Sum of electronic and thermal Free Energies= -233.489035

Center Number	Atomic Number	Atomic Type	Coordinates (Angstroms) X	Y	Z
1	6	0	-1.756440	0.540381	0.000003
2	6	0	-1.269813	1.268744	1.265615
3	1	0	-1.640280	2.305365	1.273041
4	1	0	-1.645867	0.755526	2.160847
5	1	0	-0.171428	1.297043	1.305323
6	6	0	-1.292455	-0.920152	0.000107
7	1	0	-1.671990	-1.435252	0.892890
8	1	0	-1.671851	-1.435347	-0.892682
9	1	0	-0.195429	-0.970553	0.000194
10	6	0	-1.269681	1.268600	-1.265641
11	1	0	-1.645688	0.755297	-2.160843
12	1	0	-1.640104	2.305237	-1.273212
13	1	0	-0.171292	1.296847	-1.305267
14	8	0	-3.205171	0.480778	-0.000071
15	1	0	-3.518157	1.395662	-0.000102

Int-4

Sum of electronic and thermal Free Energies= -1755.309644

Center Number	Atomic Number	Atomic Type	Coordinates (Angstroms) X	Y	Z
1	8	0	2.197461	0.755773	-1.414471
2	6	0	2.944889	-0.310319	-2.005689
3	6	0	2.652698	-1.584759	-1.237727
4	8	0	1.350715	-2.067756	-1.609188
5	6	0	0.998091	-3.218847	-0.823945
6	6	0	-0.439876	-3.597669	-1.123852
7	8	0	-1.296654	-2.653125	-0.479741
8	6	0	-2.680151	-2.853942	-0.731513
9	6	0	-3.458324	-1.924433	0.181849
10	8	0	-3.204305	-0.566641	-0.194981
11	6	0	-3.766584	0.354505	0.741648
12	6	0	-3.553718	1.769953	0.242934
13	8	0	-2.163847	2.093955	0.299239
14	6	0	-1.898591	3.389099	-0.242089
15	6	0	-0.406082	3.653105	-0.228069
16	8	0	0.236382	2.779330	-1.159753
17	6	0	1.634928	3.062333	-1.269366
18	6	0	2.299583	1.989550	-2.112257
19	1	0	4.025514	-0.085194	-1.949184
20	1	0	2.666748	-0.430055	-3.068985
21	1	0	3.419184	-2.341374	-1.490847
22	1	0	2.656529	-1.384778	-0.151699
23	1	0	1.112844	-2.977101	0.246358
24	1	0	1.656428	-4.067380	-1.089662
25	1	0	-0.617212	-3.597754	-2.215180
26	1	0	-0.641042	-4.612928	-0.735669
27	1	0	-2.919691	-2.649262	-1.791501
28	1	0	-2.972519	-3.895808	-0.503310
29	1	0	-4.538686	-2.144358	0.103205
30	1	0	-3.138474	-2.093747	1.225026
31	1	0	-3.295236	0.224715	1.732478
32	1	0	-4.853101	0.179885	0.847213
33	1	0	-3.922274	1.856707	-0.795120
34	1	0	-4.133177	2.465007	0.877905
35	1	0	-2.277658	3.451038	-1.278181
36	1	0	-2.404453	4.165140	0.361226
37	1	0	-0.236976	4.708071	-0.512152
38	1	0	0.003165	3.491875	0.784497

39	1	0	1.787349	4.050600	-1.740536
40	1	0	2.096996	3.074473	-0.267043
41	1	0	3.359203	2.268376	-2.261758
42	1	0	1.815631	1.916893	-3.103838
43	19	0	-0.248781	-0.039914	-0.208706
44	8	0	1.137363	-1.091468	1.634114
45	6	0	1.726279	-0.278137	2.564989
46	6	0	3.283570	-0.406085	2.534204
47	1	0	3.650426	-0.120117	1.536241
48	1	0	3.783917	0.226904	3.287880
49	1	0	3.560391	-1.456000	2.715282
50	6	0	1.382136	1.230782	2.304950
51	1	0	1.733231	1.508371	1.299558
52	1	0	0.289016	1.370257	2.346963
53	1	0	1.843996	1.915198	3.037074
54	6	0	1.248268	-0.623803	4.010810
55	1	0	1.485559	-1.677464	4.224183
56	1	0	1.716580	0.009999	4.784744
57	1	0	0.155026	-0.500385	4.067245

Int-5

Sum of electronic and thermal Free Energies= -1924.809279

Center Number	Atomic Number	Atomic Type	Coordinates (Angstroms) X	Y	Z
1	6	0	-0.761249	-0.024877	-2.253080
2	6	0	-0.075771	1.269133	-2.619335
3	1	0	0.960977	1.085885	-2.908884
4	1	0	-0.560865	1.779609	-3.461900
5	1	0	-0.061883	1.983564	-1.786388
6	6	0	-0.078607	-1.216320	-2.428783
7	7	0	0.505532	-2.225287	-2.541062
8	6	0	-2.122857	-0.051510	-1.814274
9	6	0	-2.871991	1.139043	-1.660836
10	6	0	-2.805581	-1.256824	-1.509108
11	6	0	-4.191817	1.119938	-1.232579
12	1	0	-2.411564	2.092801	-1.889161
13	6	0	-4.119388	-1.261314	-1.087976
14	1	0	-2.278212	-2.198274	-1.611357
15	6	0	-4.835785	-0.073283	-0.936780
16	1	0	-4.724400	2.059838	-1.130344
17	1	0	-4.598158	-2.209899	-0.868407
18	1	0	-5.866693	-0.083367	-0.605683
19	8	0	-1.481758	0.895519	2.094297
20	6	0	-1.927417	2.197898	1.700861
21	6	0	-0.770085	3.170473	1.781237
22	8	0	0.182750	2.853018	0.761650
23	6	0	1.298489	3.743015	0.772588
24	6	0	2.293080	3.305535	-0.283969
25	8	0	2.847203	2.044519	0.099973
26	6	0	3.821960	1.577469	-0.837512
27	6	0	4.398724	0.269821	-0.332258
28	8	0	3.382310	-0.727952	-0.373362
29	6	0	3.837722	-1.993475	0.115234
30	6	0	2.714201	-3.001808	-0.004060
31	8	0	1.683366	-2.672505	0.939907
32	6	0	0.549748	-3.538944	0.783956
33	6	0	-0.508472	-3.160767	1.800640
34	8	0	-1.023965	-1.871099	1.474586
35	6	0	-2.033982	-1.431764	2.381405
36	6	0	-2.535697	-0.071391	1.944313
37	1	0	-2.729423	2.543724	2.379289
38	1	0	-2.326278	2.164703	0.673920
39	1	0	-1.153816	4.196248	1.634410
40	1	0	-0.289494	3.115597	2.774819
41	1	0	1.781329	3.736141	1.766531
42	1	0	0.967954	4.774325	0.552284
43	1	0	1.793881	3.220213	-1.264414
44	1	0	3.091787	4.065468	-0.360113
45	1	0	3.357583	1.428467	-1.828095
46	1	0	4.637351	2.316256	-0.938774
47	1	0	5.249050	-0.021073	-0.975810
48	1	0	4.770872	0.398492	0.700609
49	1	0	4.162808	-1.897148	1.167389
50	1	0	4.696430	-2.343317	-0.485814
51	1	0	2.299606	-2.986304	-1.024180
52	1	0	3.114877	-4.008815	0.215000
53	1	0	0.153770	-3.445625	-0.240035
54	1	0	0.850128	-4.588334	0.959798
55	1	0	-1.317745	-3.913007	1.770930
56	1	0	-0.076195	-3.152068	2.818252
57	1	0	-2.884085	-2.137786	2.375431
58	1	0	-1.625882	-1.382671	3.407909
59	1	0	-3.393615	0.208958	2.582515
60	1	0	-2.869459	-0.107545	0.898347
61	19	0	0.703686	0.023666	0.359728

Int-6

Sum of electronic and thermal Free Energies= -2256.206025

Center Number	Atomic Number	Atomic Type	Coordinates (Angstroms) X	Y	Z
1	8	0	-3.389132	2.039077	0.216063
2	6	0	-2.703186	3.203279	0.683211
3	6	0	-2.298059	2.978814	2.126362
4	8	0	-1.305569	1.955437	2.180600
5	6	0	-0.984773	1.597471	3.527044
6	6	0	0.102548	0.542492	3.525408
7	8	0	-0.414449	-0.666380	2.963983
8	6	0	0.606416	-1.668417	2.849759
9	6	0	-0.018421	-2.958385	2.357254
10	8	0	-0.501209	-2.764123	1.029666
11	6	0	-1.086622	-3.941812	0.473630
12	6	0	-1.583591	-3.630607	-0.923797
13	8	0	-2.693730	-2.725717	-0.838249
14	6	0	-3.188610	-2.396300	-2.143848
15	6	0	-4.282600	-1.354972	-2.014700
16	8	0	-3.693691	-0.111914	-1.625944
17	6	0	-4.643306	0.941169	-1.504286
18	6	0	-3.907816	2.206668	-1.106160
19	1	0	-3.367302	4.084957	0.631524
20	1	0	-1.811394	3.400708	0.062654
21	1	0	-1.899983	3.921906	2.543473
22	1	0	-3.185205	2.683318	2.715429
23	1	0	-1.886209	1.214602	4.039231
24	1	0	-0.618926	2.481823	4.080045
25	1	0	0.967151	0.895651	2.942997
26	1	0	0.427232	0.367807	4.567910
27	1	0	1.390077	-1.336369	2.151265
28	1	0	1.065454	-1.856562	3.837625
29	1	0	0.749329	-3.752675	2.372279
30	1	0	-0.848283	-3.259985	3.022049
31	1	0	-1.922357	-4.286039	1.109707
32	1	0	-0.333708	-4.748464	0.415719
33	1	0	-0.780375	-3.172910	-1.523364
34	1	0	-1.901772	-4.574360	-1.404048
35	1	0	-2.366574	-2.000606	-2.760335
36	1	0	-3.609584	-3.298935	-2.624223
37	1	0	-4.788975	-1.244362	-2.990876
38	1	0	-5.031305	-1.672398	-1.265955
39	1	0	-5.157479	1.112017	-2.468087
40	1	0	-5.404067	0.691571	-0.742091
41	1	0	-4.609210	3.060025	-1.134907
42	1	0	-3.086269	2.402682	-1.818091
43	19	0	-1.489644	-0.254891	0.208221
44	6	0	1.595044	3.778695	-1.459685
45	6	0	2.909532	4.107503	-1.152951
46	6	0	3.639315	3.304930	-0.286523
47	6	0	3.061879	2.179704	0.287201
48	6	0	1.751794	1.891831	-0.028211
49	6	0	1.001054	2.659886	-0.893739
50	1	0	1.027134	4.391005	-2.149557
51	1	0	4.670087	3.547098	-0.058579
52	1	0	3.617300	1.519159	0.940121
53	1	0	-0.012807	2.371157	-1.136918
54	9	0	1.176032	0.814544	0.546832
55	6	0	2.360354	-0.384730	-2.222961
56	6	0	2.956407	0.633905	-3.161171
57	1	0	2.200529	1.010533	-3.852759
58	1	0	3.770433	0.215285	-3.766802
59	1	0	3.366712	1.500331	-2.625113
60	6	0	1.003946	-0.612918	-2.271896
61	7	0	-0.156593	-0.794367	-2.301474
62	1	0	3.368272	4.980572	-1.599843
63	6	0	3.145396	-1.020625	-1.212621
64	6	0	4.518008	-0.716839	-1.048249
65	6	0	2.605913	-1.965572	-0.302562
66	6	0	5.280148	-1.304155	-0.049897

67	1	0	4.987761	0.000438	-1.710664
68	6	0	3.378048	-2.544291	0.684631
69	1	0	1.555401	-2.223383	-0.365951
70	6	0	4.727761	-2.223575	0.833150
71	1	0	6.328331	-1.035994	0.038187
72	1	0	2.920295	-3.263733	1.356414
73	1	0	5.328150	-2.683205	1.608874

TS-2

Sum of electronic and thermal Free Energies= -2256.175963

Center Number	Atomic Number	Atomic Type	Coordinates (Angstroms) X	Y	Z
1	8	0	-3.469310	1.718233	0.362071
2	6	0	-2.857148	2.902067	0.889211
3	6	0	-2.439446	2.630065	2.320761
4	8	0	-1.388927	1.667503	2.311853
5	6	0	-0.943406	1.323798	3.623253
6	6	0	0.254743	0.402924	3.508371
7	8	0	-0.164092	-0.851569	2.958486
8	6	0	0.955628	-1.714763	2.721286
9	6	0	0.451364	-3.046180	2.201752
10	8	0	-0.110971	-2.860825	0.901955
11	6	0	-0.668207	-4.062173	0.367692
12	6	0	-1.244232	-3.774895	-1.004436
13	8	0	-2.387630	-2.920151	-0.869739
14	6	0	-2.955250	-2.614702	-2.151012
15	6	0	-4.106216	-1.644360	-1.973354
16	8	0	-3.587864	-0.370872	-1.583797
17	6	0	-4.605821	0.615730	-1.431872
18	6	0	-3.963579	1.908034	-0.964977
19	1	0	-3.577329	3.739978	0.875803
20	1	0	-1.974525	3.176496	0.286233
21	1	0	-2.091682	3.573093	2.780653
22	1	0	-3.302470	2.254420	2.900821
23	1	0	-1.757890	0.832027	4.186401
24	1	0	-0.637750	2.232669	4.172996
25	1	0	1.012844	0.864974	2.858111
26	1	0	0.686853	0.248417	4.514274
27	1	0	1.641969	-1.255315	1.993837
28	1	0	1.504146	-1.893227	3.664572
29	1	0	1.296341	-3.756258	2.152401
30	1	0	-0.311402	-3.456221	2.888129
31	1	0	-1.456369	-4.445371	1.040977
32	1	0	0.115463	-4.835375	0.270956
33	1	0	-0.488826	-3.286237	-1.639947
34	1	0	-1.542117	-4.732670	-1.469606
35	1	0	-2.186215	-2.167512	-2.799202
36	1	0	-3.338722	-3.538997	-2.621164
37	1	0	-4.647542	-1.556007	-2.932910
38	1	0	-4.810756	-2.018277	-1.208127
39	1	0	-5.117481	0.791600	-2.395782
40	1	0	-5.357041	0.283121	-0.692562
41	1	0	-4.719212	2.714364	-0.980224
42	1	0	-3.140455	2.188439	-1.646134
43	19	0	-1.414115	-0.417347	0.293526
44	6	0	0.526175	3.757966	-1.296925
45	6	0	1.655705	4.493362	-0.935173
46	6	0	2.678515	3.831082	-0.261083
47	6	0	2.606698	2.479659	0.023261
48	6	0	1.509210	1.712636	-0.434456
49	6	0	0.422126	2.410516	-1.021236
50	1	0	-0.303045	4.251589	-1.793931
51	1	0	3.549543	4.385165	0.073722
52	1	0	3.401197	1.981157	0.565326
53	1	0	-0.459680	1.857506	-1.324331
54	9	0	1.125825	0.672127	0.459066
55	6	0	2.224560	0.332804	-1.823988
56	6	0	2.692590	1.254400	-2.931103
57	1	0	1.837382	1.716572	-3.425762
58	1	0	3.290279	0.732707	-3.685085
59	1	0	3.295929	2.063362	-2.510173
60	6	0	1.032930	-0.360139	-2.134140
61	7	0	-0.000592	-0.847500	-2.339537
62	1	0	1.719997	5.553339	-1.143738
63	6	0	3.237260	-0.454874	-1.103710
64	6	0	4.565943	-0.018507	-1.035915
65	6	0	2.904212	-1.622284	-0.397333
66	6	0	5.510764	-0.704864	-0.281753

67	1	0	4.870257	0.868397	-1.576628
68	6	0	3.848352	-2.304509	0.347124
69	1	0	1.883600	-1.985580	-0.414467
70	6	0	5.162439	-1.849573	0.416900
71	1	0	6.530521	-0.338032	-0.247133
72	1	0	3.557656	-3.203129	0.880429
73	1	0	5.901342	-2.386113	0.999948

TS-2-a

Sum of electronic and thermal Free Energies= -2256.171380

Center Number	Atomic Number	Atomic Type	Coordinates (Angstroms) X	Y	Z
1	6	0	-3.479284	2.480649	-0.352222
2	6	0	-3.466958	2.255535	1.022592
3	6	0	-2.507134	1.381244	1.529146
4	6	0	-1.607407	0.733387	0.708702
5	6	0	-1.707330	0.859681	-0.700760
6	6	0	-2.598027	1.840488	-1.200855
7	1	0	-4.182142	3.191253	-0.774911
8	1	0	-2.452742	1.203980	2.598696
9	1	0	-0.903017	0.029270	1.136283
10	1	0	-2.604771	2.053025	-2.263001
11	9	0	-0.457416	0.828846	-1.404155
12	6	0	-2.233274	-0.990987	-1.474448
13	6	0	-2.204746	-0.830303	-2.982867
14	1	0	-1.179547	-0.815176	-3.351613
15	1	0	-2.742104	-1.638524	-3.490040
16	1	0	-2.675619	0.110859	-3.280825
17	6	0	-1.170954	-1.770228	-0.960680
18	7	0	-0.266426	-2.315588	-0.479650
19	1	0	-4.161595	2.763103	1.678793
20	6	0	-3.539821	-1.230048	-0.837949
21	6	0	-4.718826	-0.793223	-1.452887
22	6	0	-3.645061	-1.786134	0.443777
23	6	0	-5.943200	-0.894036	-0.809081
24	1	0	-4.681380	-0.357223	-2.443020
25	6	0	-4.868522	-1.888527	1.083766
26	1	0	-2.752060	-2.139697	0.946529
27	6	0	-6.027419	-1.436299	0.465279
28	1	0	-6.838459	-0.540562	-1.308153
29	1	0	-4.917214	-2.321367	2.076706
30	1	0	-6.984470	-1.514026	0.967341
31	8	0	1.450935	1.630496	2.649192
32	6	0	0.818938	2.885392	2.358251
33	6	0	1.645864	3.619010	1.322415
34	8	0	1.586785	2.892410	0.097229
35	6	0	2.266803	3.549337	-0.968965
36	6	0	2.123250	2.712810	-2.224696
37	8	0	2.866910	1.498989	-2.058815
38	6	0	2.760327	0.649749	-3.204344
39	6	0	3.597439	-0.592889	-2.970407
40	8	0	2.985479	-1.371914	-1.944518
41	6	0	3.754417	-2.522616	-1.589919
42	6	0	3.006009	-3.310264	-0.533056
43	8	0	2.992582	-2.567200	0.693499
44	6	0	2.218562	-3.242380	1.694879
45	6	0	2.228394	-2.425289	2.970554
46	8	0	1.465814	-1.230512	2.777957
47	6	0	1.480199	-0.391756	3.933423
48	6	0	0.724832	0.886533	3.630355
49	1	0	0.761613	3.499708	3.275296
50	1	0	-0.198729	2.712091	1.972992
51	1	0	1.227680	4.633290	1.187438
52	1	0	2.693950	3.714423	1.661595
53	1	0	3.335842	3.682949	-0.720264
54	1	0	1.822917	4.545515	-1.149536
55	1	0	1.060470	2.482693	-2.398609
56	1	0	2.517668	3.282611	-3.085666
57	1	0	1.705890	0.370920	-3.374520
58	1	0	3.135861	1.172046	-4.103147
59	1	0	3.656712	-1.174195	-3.908635
60	1	0	4.621628	-0.302361	-2.673531
61	1	0	4.746266	-2.214401	-1.211598
62	1	0	3.903288	-3.168224	-2.474591
63	1	0	1.972928	-3.494767	-0.865127
64	1	0	3.519140	-4.277699	-0.380155
65	1	0	1.186968	-3.377159	1.335529
66	1	0	2.662284	-4.232270	1.908860

67	1	0	1.787524	-3.028301	3.785006
68	1	0	3.266559	-2.168276	3.249085
69	1	0	0.996346	-0.904605	4.784490
70	1	0	2.521589	-0.152563	4.215492
71	1	0	0.631145	1.473027	4.562686
72	1	0	-0.288156	0.655853	3.261498
73	19	0	1.662052	-0.016678	0.185853

TS-2-b

Sum of electronic and thermal Free Energies= -2256.163462

Center Number	Atomic Number	Atomic Type	Coordinates (Angstroms) X	Y	Z
1	6	0	-1.065393	-3.967024	0.047759
2	6	0	0.078064	-4.250368	-0.698763
3	6	0	0.787400	-3.180978	-1.237713
4	6	0	0.384125	-1.871062	-1.053615
5	6	0	-0.834471	-1.595240	-0.387562
6	6	0	-1.503264	-2.672442	0.238665
7	1	0	-1.621755	-4.776867	0.508309
8	1	0	1.688346	-3.371795	-1.812146
9	1	0	0.963799	-1.063149	-1.484112
10	1	0	-2.383372	-2.463428	0.834665
11	9	0	-0.826038	-0.404731	0.396357
12	6	0	-2.132821	-0.608743	-1.758628
13	1	0	0.409372	-5.269785	-0.846863
14	8	0	3.655403	-1.099945	0.710432
15	6	0	3.237717	-2.226780	1.498498
16	6	0	2.760271	-1.737108	2.852067
17	8	0	1.554974	-0.998641	2.665114
18	6	0	0.965097	-0.546858	3.883767
19	6	0	-0.349087	0.137043	3.558178
20	8	0	-0.090244	1.373889	2.880387
21	6	0	-1.312460	1.991955	2.449311
22	6	0	-0.991520	3.268294	1.697902
23	8	0	-0.393907	2.942935	0.437892
24	6	0	0.014858	4.101806	-0.288424
25	6	0	0.518680	3.674733	-1.653529
26	8	0	1.698319	2.877251	-1.492833
27	6	0	2.145184	2.326962	-2.737271
28	6	0	3.450900	1.589211	-2.515646
29	8	0	3.213099	0.431318	-1.711134
30	6	0	4.420488	-0.281495	-1.420836
31	6	0	4.094673	-1.506317	-0.589978
32	1	0	4.087174	-2.917884	1.644797
33	1	0	2.422458	-2.759723	0.981719
34	1	0	2.576273	-2.611812	3.501491
35	1	0	3.531776	-1.103857	3.327641
36	1	0	1.645828	0.149555	4.407044
37	1	0	0.763370	-1.405272	4.549353
38	1	0	-0.954402	-0.520970	2.915357
39	1	0	-0.898243	0.330455	4.497458
40	1	0	-1.879360	1.306137	1.803155
41	1	0	-1.932947	2.249215	3.327070
42	1	0	-1.926410	3.832669	1.534105
43	1	0	-0.302676	3.895750	2.291967
44	1	0	0.807030	4.638270	0.265094
45	1	0	-0.838250	4.789600	-0.428869
46	1	0	-0.261487	3.096974	-2.174632
47	1	0	0.749352	4.574663	-2.251274
48	1	0	1.381439	1.641943	-3.142600
49	1	0	2.318791	3.134698	-3.470552
50	1	0	3.865080	1.291988	-3.495510
51	1	0	4.177321	2.255063	-2.016085
52	1	0	4.902157	-0.607327	-2.359766
53	1	0	5.123689	0.374412	-0.876698
54	1	0	5.003616	-2.129305	-0.505438
55	1	0	3.308431	-2.097468	-1.084404
56	19	0	1.539876	0.842786	0.552755
57	6	0	-3.292491	-0.221273	-0.950932
58	6	0	-4.308244	-1.141794	-0.644273
59	6	0	-3.397416	1.059786	-0.384682
60	6	0	-5.353878	-0.806732	0.201482
61	1	0	-4.273074	-2.133529	-1.080861
62	6	0	-4.446731	1.390471	0.457465
63	1	0	-2.632747	1.799757	-0.582122
64	6	0	-5.433032	0.460597	0.764760
65	1	0	-6.118598	-1.544390	0.418372
66	1	0	-4.495103	2.389117	0.879089

67	1	0	-6.254136	0.722677	1.421248
68	6	0	-2.369649	-1.665082	-2.678263
69	7	0	-2.508491	-2.553328	-3.406405
70	6	0	-1.267985	0.490497	-2.342654
71	1	0	-1.826137	1.152731	-3.015860
72	1	0	-0.841710	1.106018	-1.549925
73	1	0	-0.443999	0.061024	-2.916298

Int-7

Sum of electronic and thermal Free Energies= -2256.268039

Center Number	Atomic Number	Atomic Type	Coordinates (Angstroms) X	Y	Z
1	8	0	-2.857822	2.452469	-0.070660
2	6	0	-2.588238	3.127518	1.160615
3	6	0	-3.209690	2.332998	2.292686
4	8	0	-2.530492	1.090897	2.381628
5	6	0	-2.956156	0.290099	3.486822
6	6	0	-2.136664	-0.982935	3.503565
7	8	0	-2.578406	-1.843401	2.439344
8	6	0	-1.692505	-2.968237	2.315917
9	6	0	-2.120140	-3.802768	1.127093
10	8	0	-1.749315	-3.122034	-0.074529
11	6	0	-2.236483	-3.763221	-1.246259
12	6	0	-1.749366	-2.997660	-2.460256
13	8	0	-2.361747	-1.704265	-2.475354
14	6	0	-1.969889	-0.961882	-3.631891
15	6	0	-2.651189	0.389490	-3.608290
16	8	0	-2.134263	1.172146	-2.529781
17	6	0	-2.777715	2.442559	-2.457046
18	6	0	-2.334832	3.154224	-1.195923
19	1	0	-3.022924	4.144136	1.141806
20	1	0	-1.499108	3.210182	1.319952
21	1	0	-3.101196	2.906239	3.233052
22	1	0	-4.288145	2.178074	2.101890
23	1	0	-4.035189	0.063040	3.398975
24	1	0	-2.790151	0.839614	4.432224
25	1	0	-1.070876	-0.745821	3.339115
26	1	0	-2.280135	-1.489123	4.477724
27	1	0	-0.664618	-2.588594	2.179707
28	1	0	-1.749349	-3.594106	3.227413
29	1	0	-1.620043	-4.788164	1.169156
30	1	0	-3.213843	-3.965130	1.147820
31	1	0	-3.341889	-3.796022	-1.234402
32	1	0	-1.858824	-4.801550	-1.306443
33	1	0	-0.652915	-2.891026	-2.427133
34	1	0	-2.027483	-3.559905	-3.371598
35	1	0	-0.876427	-0.837361	-3.647817
36	1	0	-2.283745	-1.495107	-4.549521
37	1	0	-2.466706	0.899133	-4.572535
38	1	0	-3.742085	0.259280	-3.487234
39	1	0	-2.515329	3.058912	-3.337324
40	1	0	-3.875229	2.314419	-2.436970
41	1	0	-2.718287	4.191433	-1.216859
42	1	0	-1.233048	3.192747	-1.148896
43	19	0	-1.368503	-0.294524	0.105821
44	6	0	3.211620	3.152412	1.606434
45	6	0	2.087932	3.904216	1.299705
46	6	0	1.289822	3.531173	0.228485
47	6	0	1.610928	2.414761	-0.528287
48	6	0	2.736013	1.655451	-0.222239
49	6	0	3.533834	2.035140	0.849648
50	1	0	3.839783	3.428846	2.444414
51	1	0	0.410024	4.111934	-0.018041
52	1	0	0.968875	2.129230	-1.355893
53	1	0	4.407640	1.450427	1.108775
54	9	0	0.384653	-0.738605	1.864198
55	6	0	3.139058	0.463222	-1.105841
56	6	0	1.923111	-0.222508	-1.580856
57	7	0	0.983997	-0.759966	-1.963074
58	1	0	1.831381	4.772225	1.894615
59	6	0	3.864678	1.010127	-2.353308
60	1	0	4.751108	1.564135	-2.043779
61	1	0	4.157377	0.204771	-3.028062
62	1	0	3.207174	1.695144	-2.888265
63	6	0	3.953813	-0.582526	-0.330110
64	6	0	5.282129	-0.848691	-0.635370
65	6	0	3.344252	-1.262893	0.724106
66	6	0	5.999154	-1.783887	0.101987

67	1	0	5.778319	-0.333512	-1.446723
68	6	0	4.062093	-2.196247	1.454095
69	1	0	2.311398	-1.058139	0.997306
70	6	0	5.391893	-2.460201	1.145796
71	1	0	7.035081	-1.980352	-0.147118
72	1	0	3.583022	-2.721533	2.271082
73	1	0	5.949651	-3.190186	1.719886

References

1. (a) C. Hansch, A. Leo, R. W. A. Taft, *Chem. Rev.* **1991**, *91*, 165. (b) J. A. Dean, Ed. *Lange's Handbook of Chemistry*, McGraw-Hill, New York, 1998. (c) L. P. Hammett, *J. Am. Chem. Soc.* **1937**, *59*, 96.

2. M. Shigeno, K. Hanasaka, I. Tohara, K. Izumi, H. Yamakoshi, E. Kwon, K. Nozawa-Kumada, Y. Kondo, *Org. Lett.* **2022**, *24*, 809.

3. H. Yang, W. Dong, W.Wang, T. Li, W. Zhao, *Synthesis* **2020**, *52*, 2833.

4. M. Rubin, A. Trofimov, V. Gevorgyan, *J. Am. Chem. Soc.* **2005**, *127*, 10243.

5. B. Février, G. Dupas, J. Bourguignon, G. Quéguiner, J. *Heterocyclic Chem.* **1993**, *30*, 1085.

6. É. Rochette, V. Desrosiers, Y. Soltani, F.-G. Fontaine, *J. Am. Chem. Soc.* **2019**, *141*, 12305.

7. J. W. W. Chang, S. Chee, S. Mak, P. Buranaprasertsuk, W. Chavasiri, P. Wai, H. Chan, *Tetrahedron Lett.* **2008**, *49*, 2018.

8. T. Niwa, H. Ochiai, Y. Watanabe, T. Hosoya, *J. Am. Chem. Soc.* **2015**, *137*, 14313.

9. F.-F. Yong, Y.-C. Teo, G.-L. Chua, G. S. Lim, Y. Lin, *Tetrahedron Lett.* **2011**, *52*, 1169.

10. A. C. Hernandez-Perez, A. Caron, S. K. Collins, *Chem. Eur. J.* **2015**, *21*, 16673.

11. J. Zhou, B. Jiang, Y. Fujihira, Z. Zhao, T. Imai, N. Shibata, *Nat. Commun.* **2021**, 12, 3749.

12. K. Kodama, H. Kanai, Y. Shimomura, T. Hirose, *Eur. J. Org. Chem.* **2018**, *14*, 1726.

13. J. Guin, G. Varseev, B. List, *J. Am. Chem. Soc,* **2013**, *135*, 2100.

14. M. Shigeno, K. Hayashi, K. Nozawa-Kumada, Y. Kondo, *Org. Lett.* **2020**, *22*, 9107.

15. T. Patra, S. Agasti, Akanksha, D. Maiti, *Chem. Commun.* **2013**, *49*, 69.

16. K. Kodama, H. Kanai, Y. Shimomura, T. Hirose, *Eur. J. Org. Chem.*, **2018**, 1726.

17. M. Shigeno, Y. Shishido, A. Soga, K. Nozawa-Kumada, Y. Kondo, *J. Org. Chem.* **2023**, *88*, 1796.

18. P. Kozikowski, W. Tückmantel, C. George, *J. Org. Chem.*, **2000**, *65*, 5371.

19. L. Crombie, P. Tuchinda, M. J. Powell, *J. Chem. Soc. Perkin Trans. 1*, **1982**, 1477.

20. R. Bellemin, J. Decerprit, D. Festal, *Eur. J. Med. Chem.* **1996**, *31*, 123.

21. K. W. Shimkin, P. G. Gildner, D. A. Watson, *Org. Lett.* **2016**, *18*, 988.

22. J. Malineni, R. L. Jezorek, N. Zhang, V. Percec, *Synthesis* **2016**, *48*, 2795.

23. T. Niwa, H. Ochiai, Y. Watanabe, T. Hosoya, *J. Am. Chem. Soc.* **2015**, *137*, 14313.

24. L. R. Mills, P. Patel, S. A. L. Rousseaux, *Org. Biomol. Chem.,* **2022,** *20,* 5933.

25. M. Makosza, M. Ludwikow, A. Urniaz, *Rocz Chem.* **1975**, *49*, 297.

26. P. Orecchia, W. Yuan, M. Oestreich, *Angew. Chem. Int. Ed.* **2019**, *58*, 3579.

27. L. R. Mills, R. K. Edjoc, S. A. L. Rousseaux, *J. Am. Chem. Soc.* **2021**, *143*, 10422.

28. Z. Jiao, K. W. Chee, J. S. Zhou, *J. Am. Chem. Soc.* **2016**, *138*, 16240.

29. S. Tang, R. Guillot, L. Grimaud, M. R. Vitale, G. Vincent, *Org. Lett.* **2022**, *24*, 2125.

30. P. Morand, J. F. Bagli, M. Kraml, J. Dubuc, *J. Med. Chem.* **1964**, *7*, 504.

31. T. K. Salvador, C. H. Arnett, S. Kundu, N. G. Sapiezynski, J. A. Bertke, M. R. Boroujeni, T. H. Warren, *J. Am. Chem. Soc.* **2016**, *138*, 16580.

32. Z. Song, X. Huang, S. Jiang, C. He, L. Tang, Q. Ni, M. Ma, B. Chen, Y. Ma, *Org. Lett.* **2022**, *24*,

5573.
33. R. A. Escobar, J. W. Johannes, *Chem. Eur. J.* **2020**, *26*, 5168.
34. Q. Lemesre, T. Wiesner, R. Wiechert, E. Rodrigo, S. Triebel, H. Geneste, *Green Chem.* **2022**, *24*, 5502.
35. Y. Monguchi, K. Kitamoto, T. Ikawa, T. Maegawa, H. Sajiki, *Adv. Synth. Catal.* **2008**, *350*, 2767.
36. B. H. Lipshutz, D. M. Nihan, E. Vinogradova, B. R. Taft, Ž. V. Bošković, *Org. Lett.* **2008**, *10*, 4279.
37. J. Liu, X. Jia, X. Chen, H. Sun, Y. Li, S. Kramer, Z. Lian, *J. Org. Chem.* **2020**, *85*, 5702.
38. G. A. Molander, L. Iannazzo, *J. Org. Chem.* **2011**, *76*, 9182.
39. H. Zhang, Q. Cai, D. Ma, *J. Org. Chem.* **2005**, *70*, 5164.
40. H. Yang, C. Xi, Z. Miao, R. Chen, *Eur. J. Org. Chem.* **2011**, 3353.
41. Z. Li, F. Meng, J. Zhang, J. Xiea, B. Dai, *Org. Biomol. Chem.* **2016**, *14*, 10861.
42. C. T. Lester, G. F. Rodgers, E. E. Reid, *J. Am. Chem. Soc.* **1944**, *66*, 1674.
43. M. Tiecco, L. Testaferri, M. Tingoli, E. Wenkert, *Tetrahedron* **1983**, *39*, 2289.
44. B. A. Hopkins, B. Zavesky, D. White, *J. Org. Chem.* **2022**, *87*, 7547.
45. D. S. Tarbell, D. P. Harnish, *J. Am. Chem. Soc.* **1952**, *74*, 1862.
46. G. Sun, X. Liu, J. Li, J.-X. Yang, J.-K. Xie, X. Wen, H. Sun, Q.-L. Xu, *New J. Chem.* **2023**, *47*, 4746.
47. L. Le, S. Li, D. Zhang, S.-F. Yin, N. Kambe, R. Qiu, *Org. Lett.* **2022**, *24*, 6159.
48. D.-L. Zhu, S. Jiang, Q. Wu, H. Wang, L.-L. Chai, H.-Y. Li, H.-X. Li, *Org. Lett.* **2021**, *23*, 160.
49. Z. You, K. Higashida, T. Iwai, M. Sawamura, *Angew. Chem. Int. Ed.* **2021**, *60*, 5778.
50. Gaussian 16, Revision C.01, M. J. Frisch, G. W. Trucks, H. B. Schlegel, G. E. Scuseria, M. A. Robb, J. R. Cheeseman, G. Scalmani, V. Barone, G. A. Petersson, H. Nakatsuji, X. Li, M. Caricato, A. V. Marenich, J. Bloino, B. G. Janesko, R. Gomperts, B. Mennucci, H. P. Hratchian, J. V. Ortiz, A. F. Izmaylov, J. L. Sonnenberg, D. Williams-Young, F. Ding, F. Lipparini, F. Egidi, J. Goings, B. Peng, A. Petrone, T. Henderson, D. Ranasinghe, V. G. Zakrzewski, J. Gao, N. Rega, G. Zheng, W. Liang, M. Hada, M. Ehara, K. Toyota, R. Fukuda, J. Hasegawa, M. Ishida, T. Nakajima, Y. Honda, O. Kitao, H. Nakai, T. Vreven, K. Throssell, J. A. Montgomery, Jr., J. E. Peralta, F. Ogliaro, M. J. Bearpark, J. J. Heyd, E. N. Brothers, K. N. Kudin, V. N. Staroverov, T. A. Keith, R. Kobayashi, J. Normand, K. Raghavachari, A. P. Rendell, J. C. Burant, S. S. Iyengar, J. Tomasi, M. Cossi, J. M. Millam, M. Klene, C. Adamo, R. Cammi, J. W. Ochterski, R. L. Martin, K. Morokuma, O. Farkas, J. B. Foresman, and D. J. Fox, Gaussian, Inc., Wallingford CT, 2019.
51. S. Dapprich, I. Komáromi, K. S. Byun, K. Morokuma, M. J. Frisch, *J. Mol. Struct. (Theochem)* **1999**, *462*, 1.
52. S. Grimme, *J. Comput. Chem.* **2006**, *27*, 1787.
53. For Gaussian basis sets: (a) M. J. Frisch, J. A. Pople, J. S. Binkley, *J. Chem. Phys.* **1984**, *80*, 3265. (b) W. J. Hehre, L. Radom, P. v. R. Schleyer, J. A. Pople in *Ab initio Molecular Orbital Theory*; John Wiley: New York, USA, **1986**, and references cited therein.
54. V. Barone, M. Cossi, *J. Phys. Chem. A* **1998**, *102*, 1995.

55. K. Fukui, *Acc. Chem. Res.* **1981**, *14*, 363.

56. Nuclear charge minus summed natural populations of NAOs on the atom.

57. G. K. Schenter, E. D. Glendening, *J. Phys. Chem*. **1996**, *100*, 17152.

58. E. R. Johnson, S. Keinan, P. Mori-Sánchez, J. Contreras-García, A. J. Cohen, W. Yang, *J. Am. Chem. Soc*. **2010**, 132, 6498.

59. J. Contreras-Garcia, E. R. Johnson, S. Keinan, R. Chaudret, J. P. Piquemal, D. N. Beratan, W. T. Yang, *J. Chem. Theory Comput.* **2011**, *7*, 625.

60. W. Humphrey, A. Dalke, K. Schulten, *J. Mol. Graph*. **1996**, *14*, 27.

61. (a) A. Rahalkar, A. Stanger, Aroma. http://chemistry.technion.ac.il/ members/amnon-stanger/. (b) A. Stanger, *J. Org. Chem.* **2006**, *71*, 883. (c) A. Stanger, *J. Org. Chem.* **2010**, *75*, 2281. (d) R. Gershoni-Poranne, A. Stanger, *Chem. Eur. J.* **2014**, *20*, 5673.

^{1}H NMR spectra of **1s** ($CDCl_3$, 400 MHz)

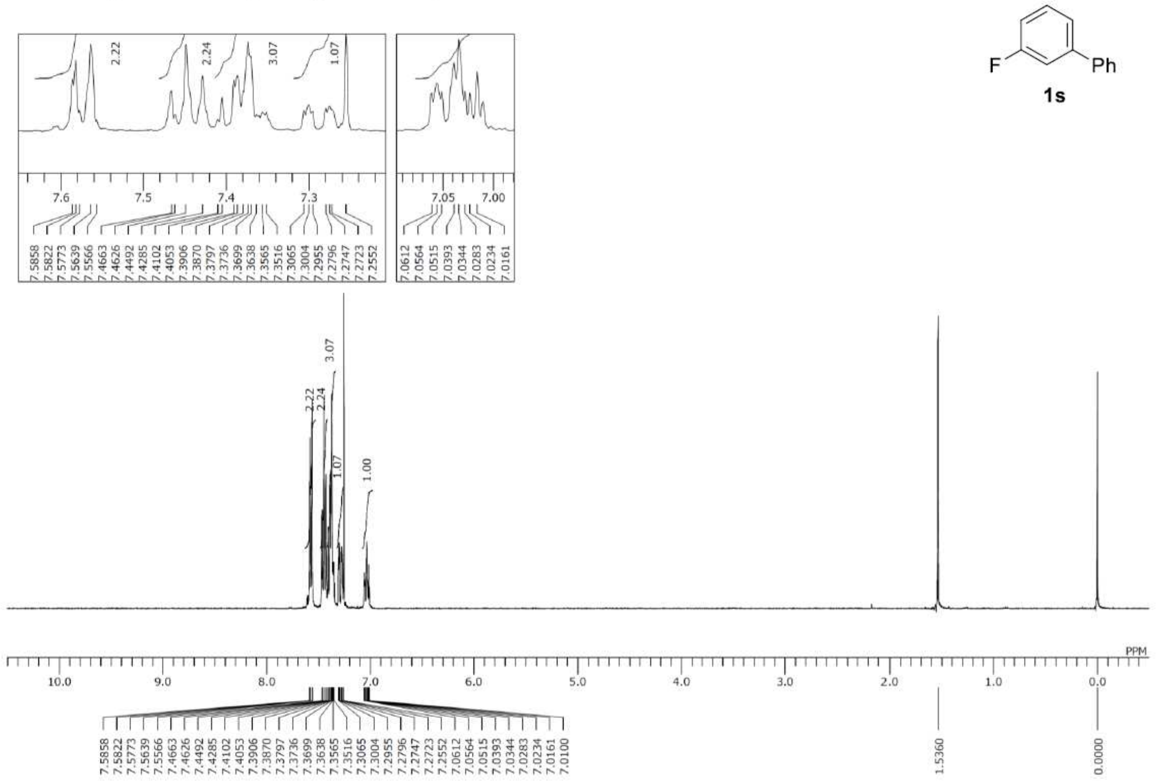

^{13}C NMR spectra of **1s** ($CDCl_3$, 100 MHz)

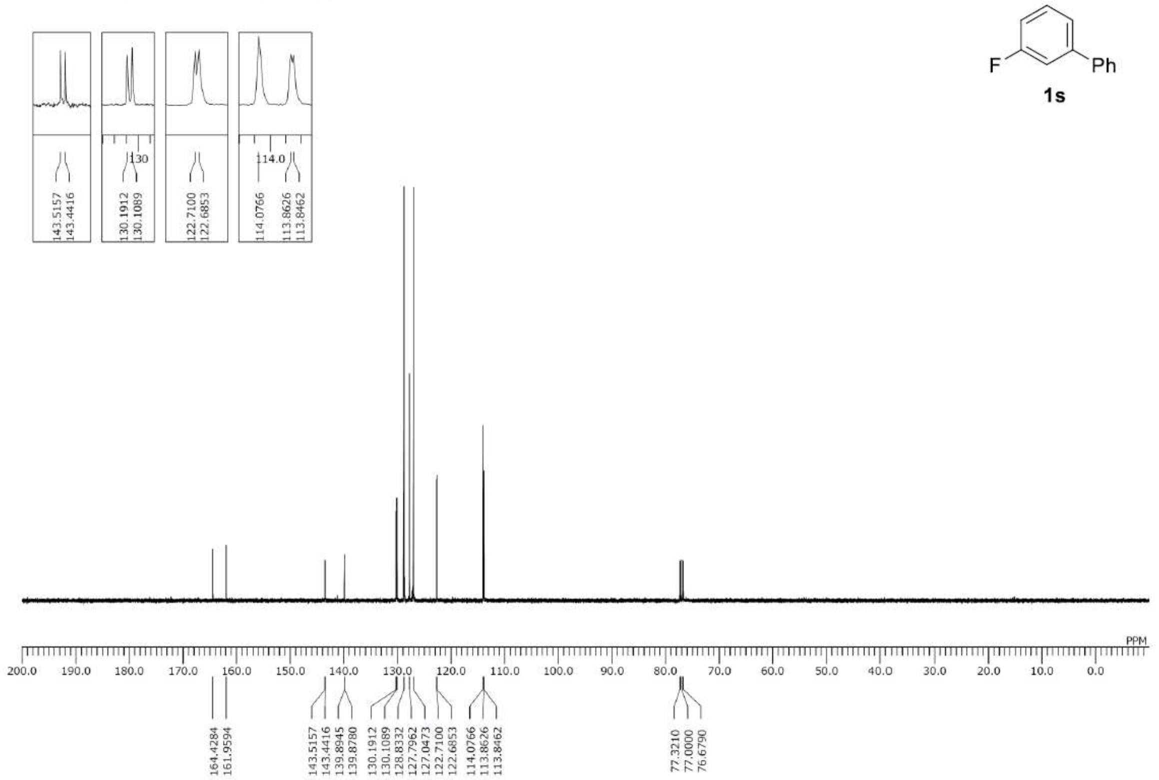

^{19}F NMR spectra of **1s** ($CDCl_3$, 375 MHz)

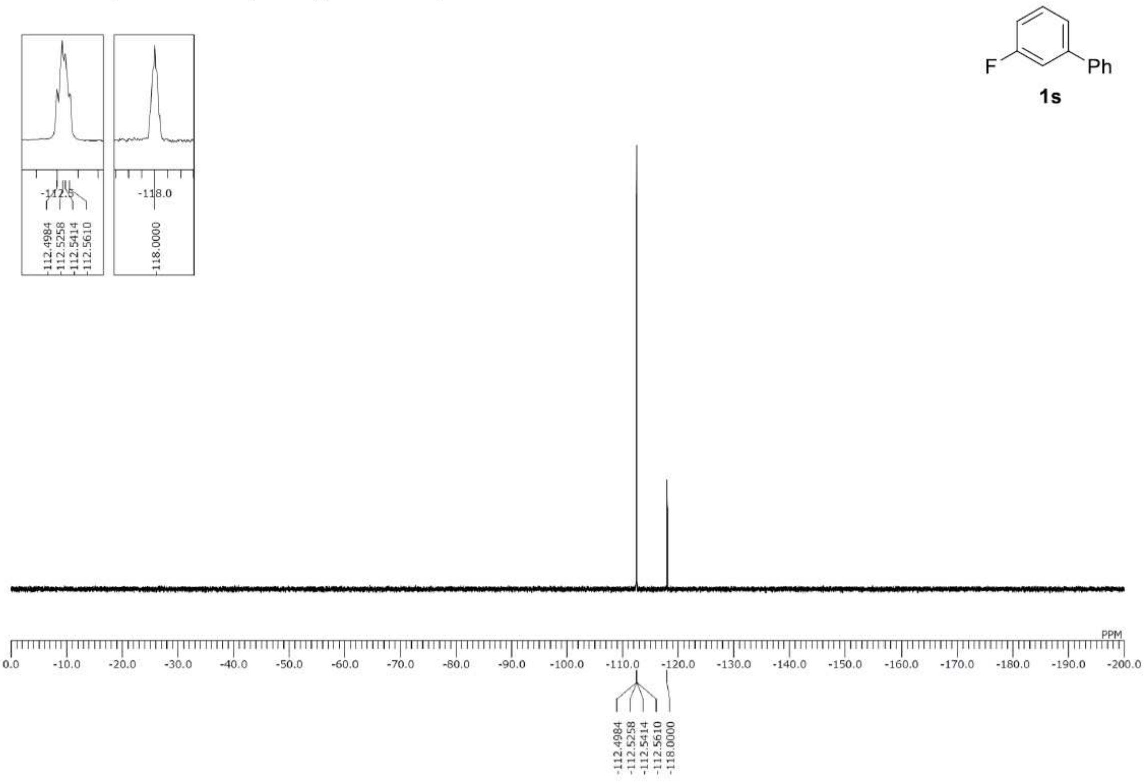

^{1}H NMR spectra of **1w** ($CDCl_3$, 400 MHz)

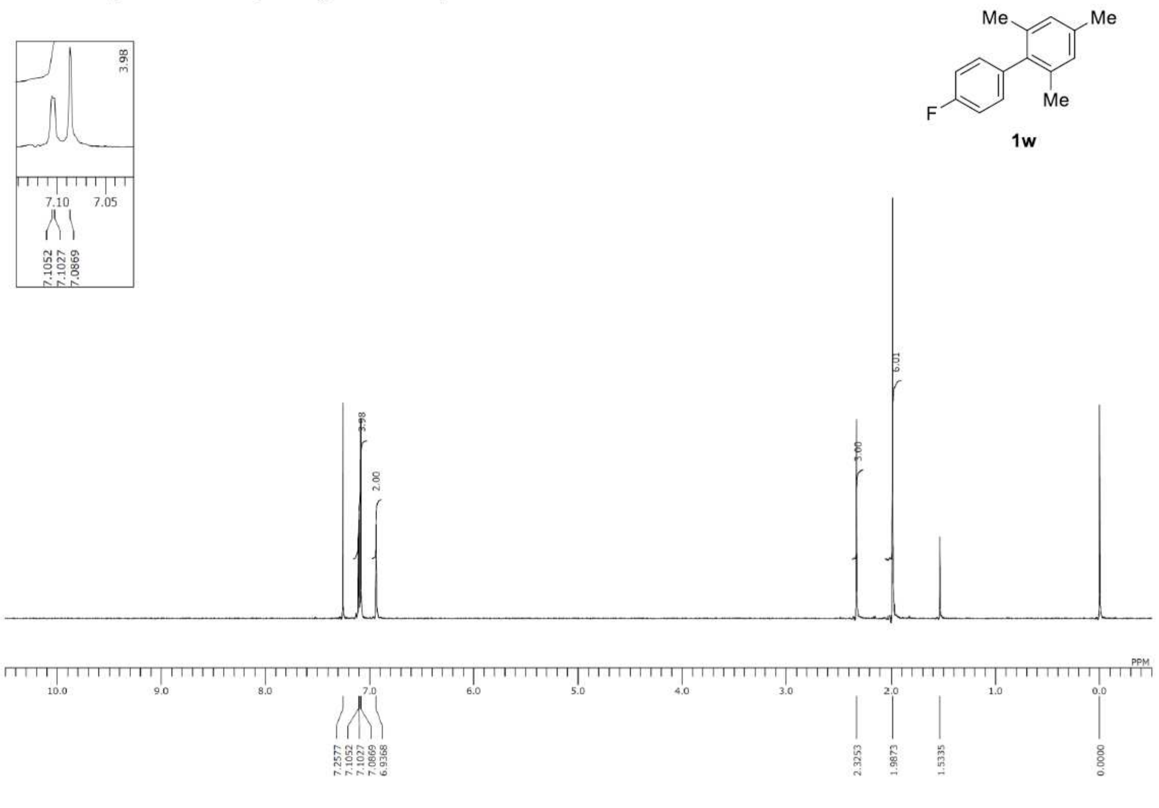

^{13}C NMR spectra of **1w** ($CDCl_3$, 100 MHz)

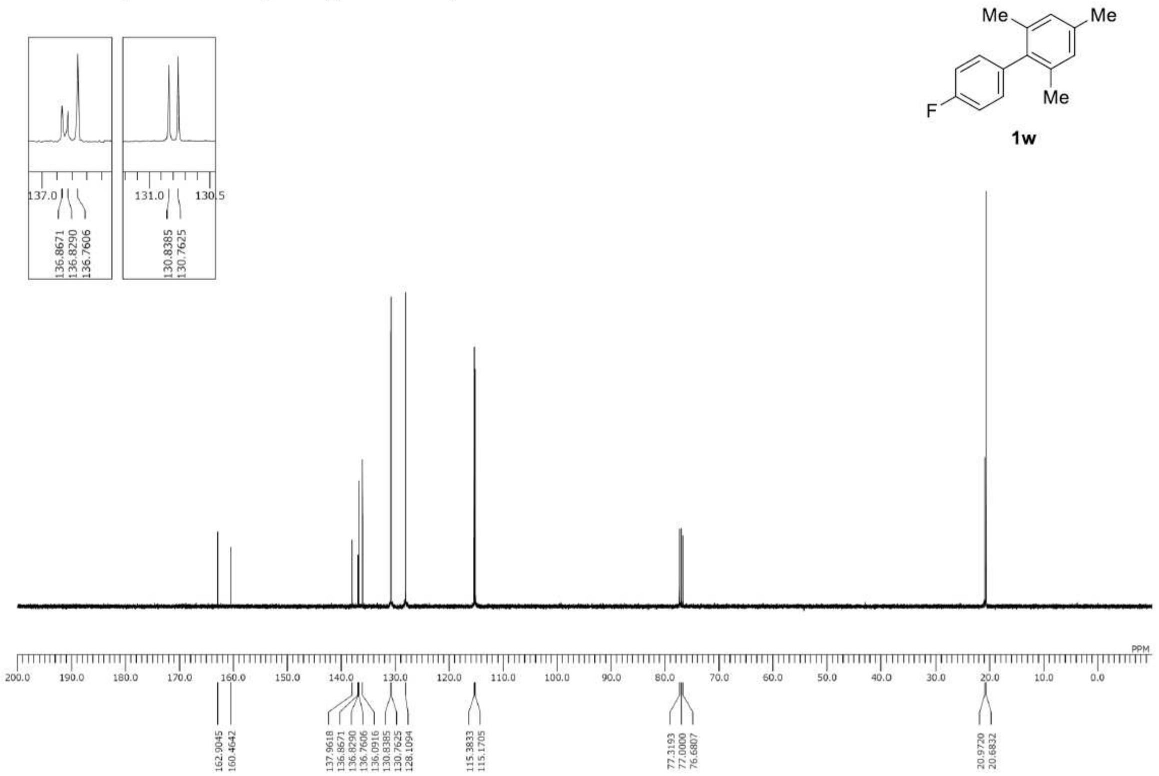

^{19}F NMR spectra of **1w** ($CDCl_3$, 375 MHz)

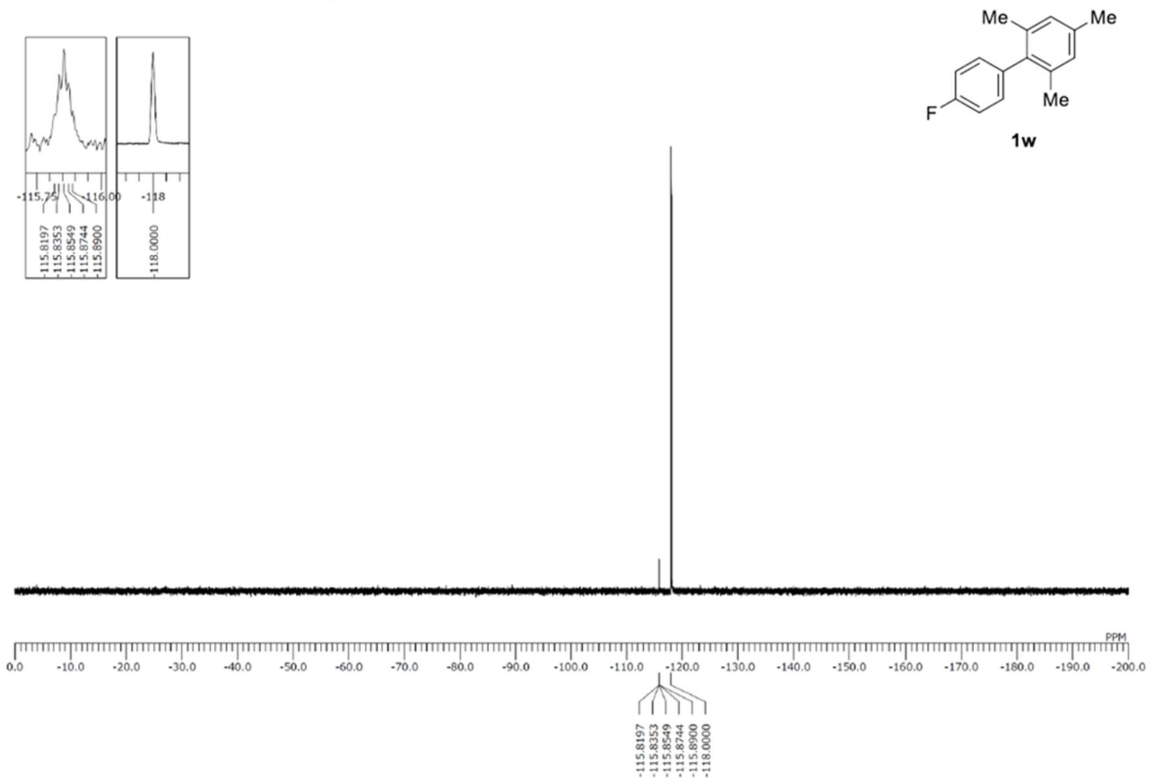

^{1}H NMR spectra of **1N** ($CDCl_3$, 600 MHz)

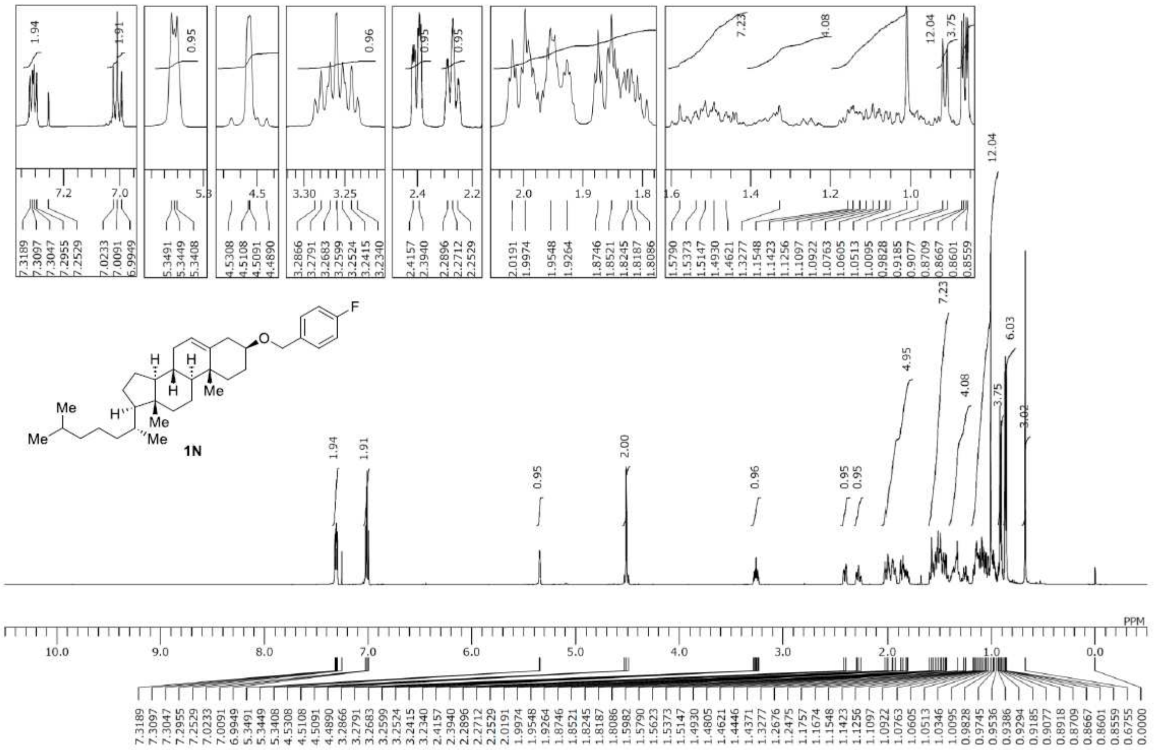

^{13}C NMR spectra of **1N** ($CDCl_3$, 150 MHz)

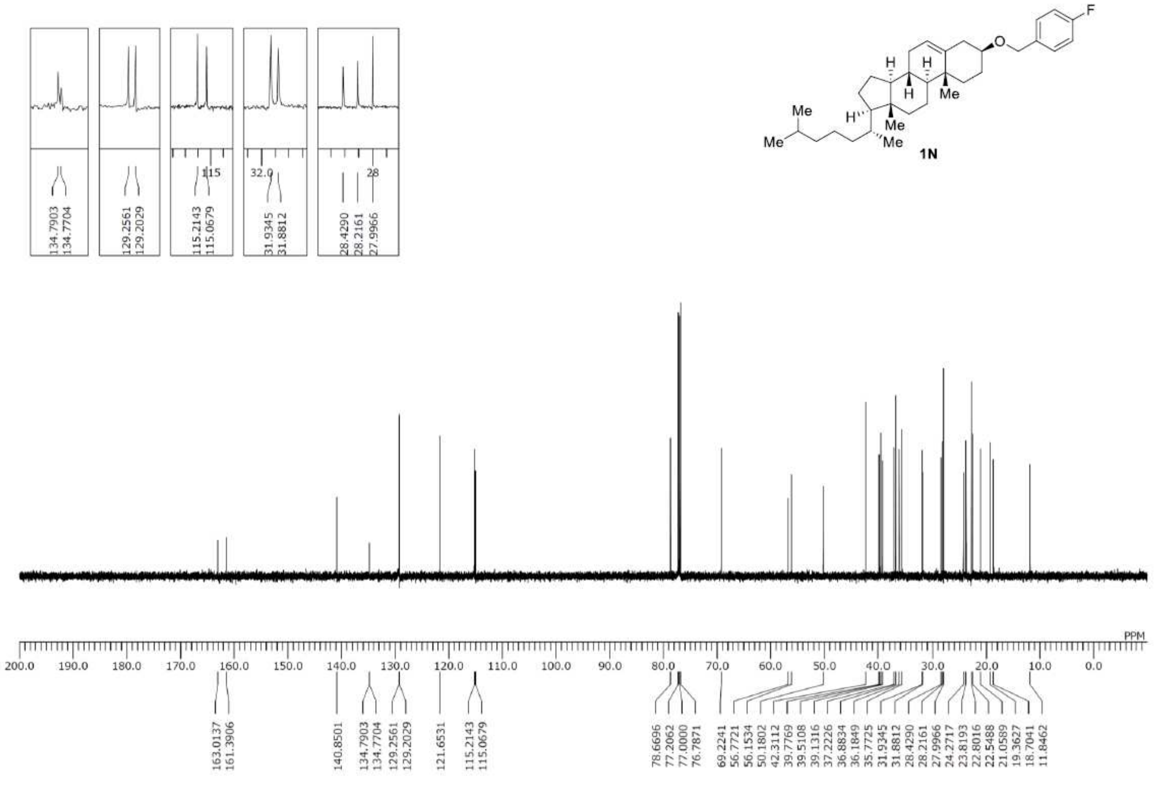

^{19}F NMR spectra of **1N** ($CDCl_3$, 375 MHz)

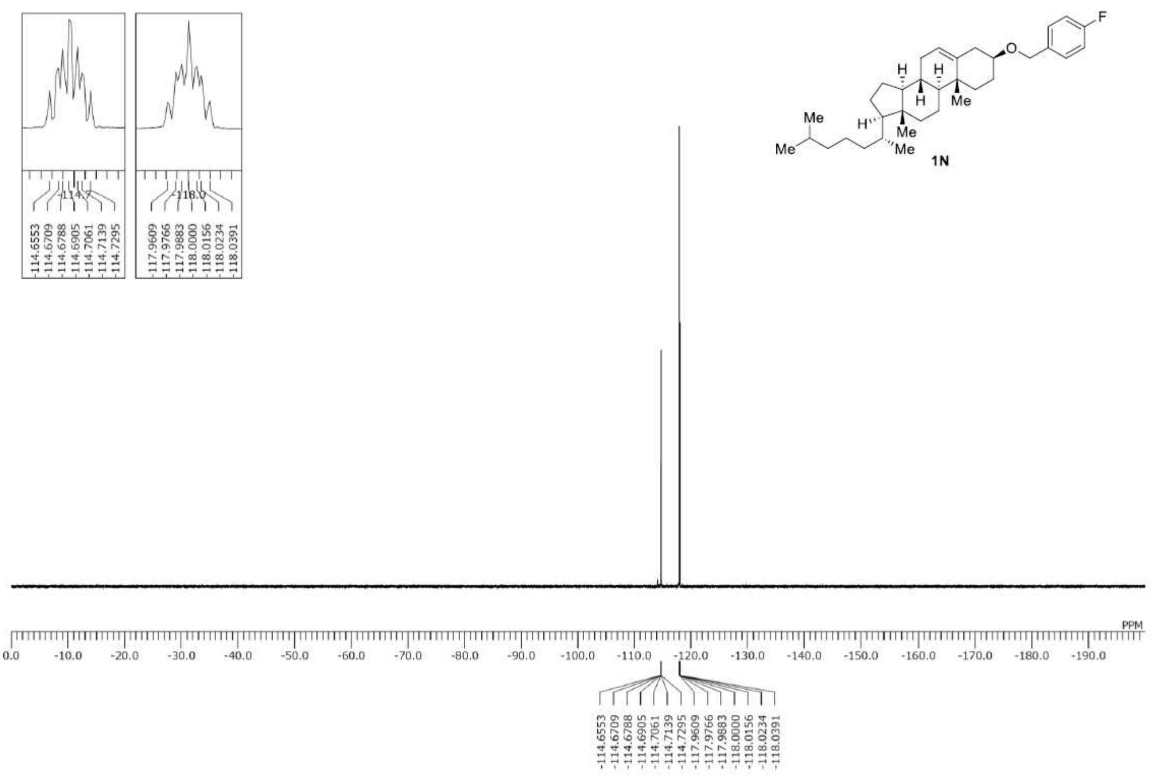

^{1}H NMR spectra of **1O** ($CDCl_3$, 600 MHz)

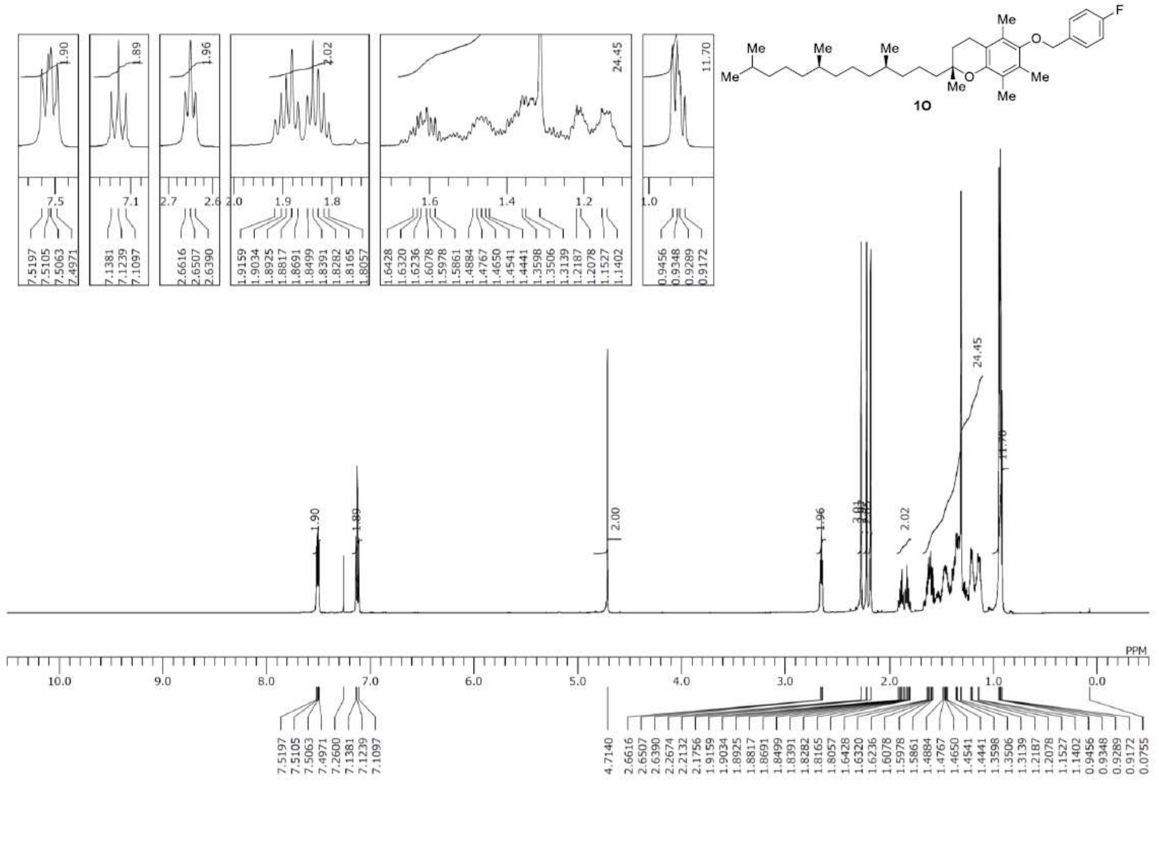

^{13}C NMR spectra of **1O** ($CDCl_3$, 150 MHz)

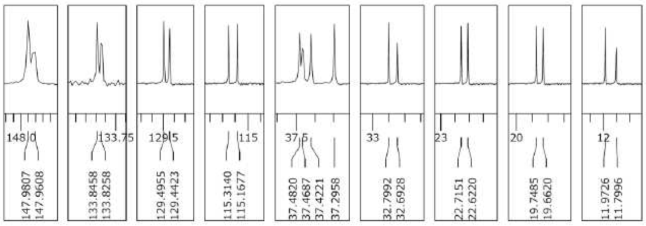

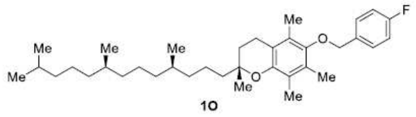

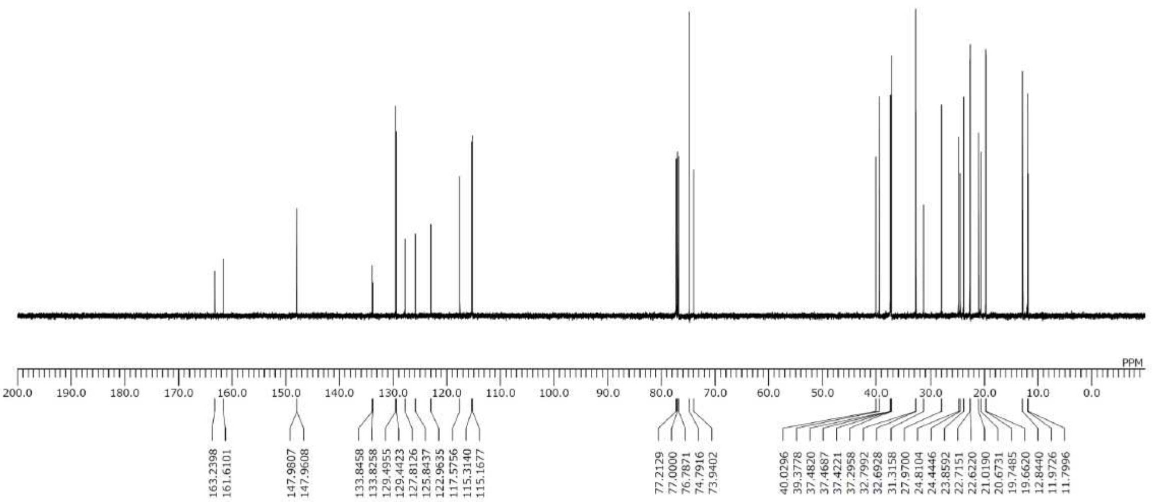

^{19}F NMR spectra of **1O** ($CDCl_3$, 375 MHz)

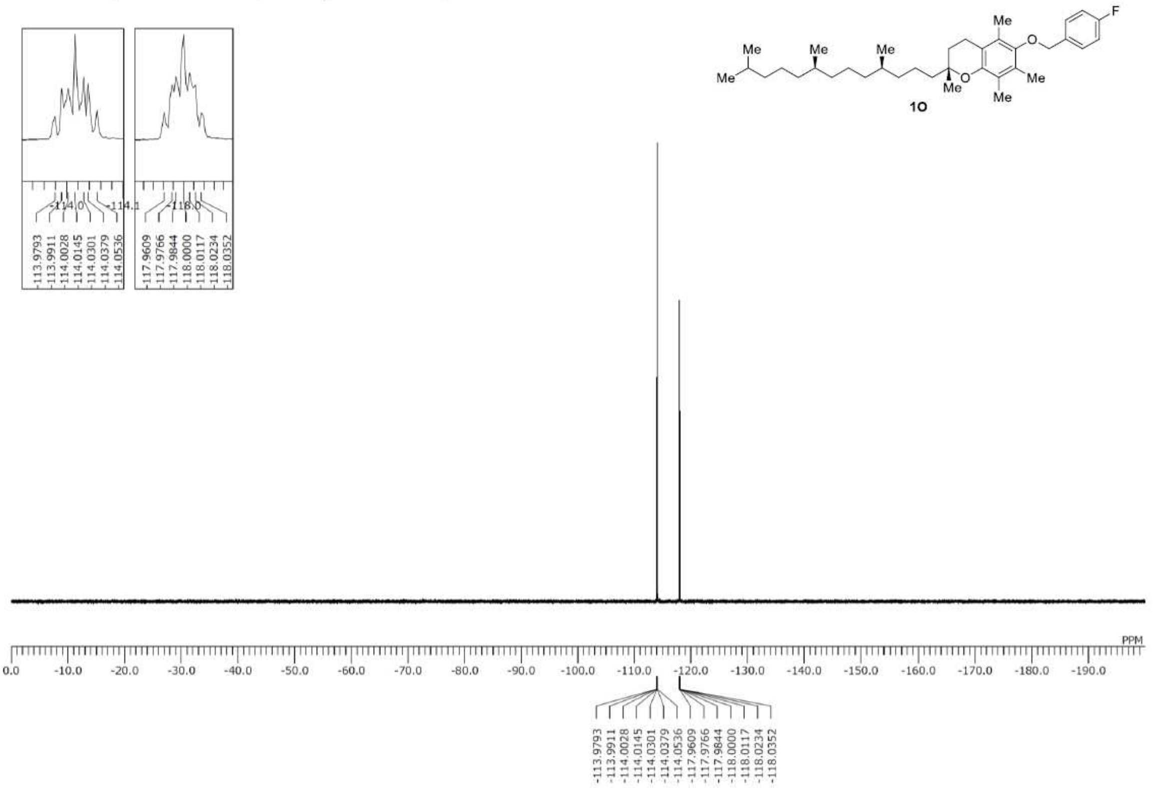

^{1}H NMR spectra of **3aa** ($CDCl_3$, 400 MHz)

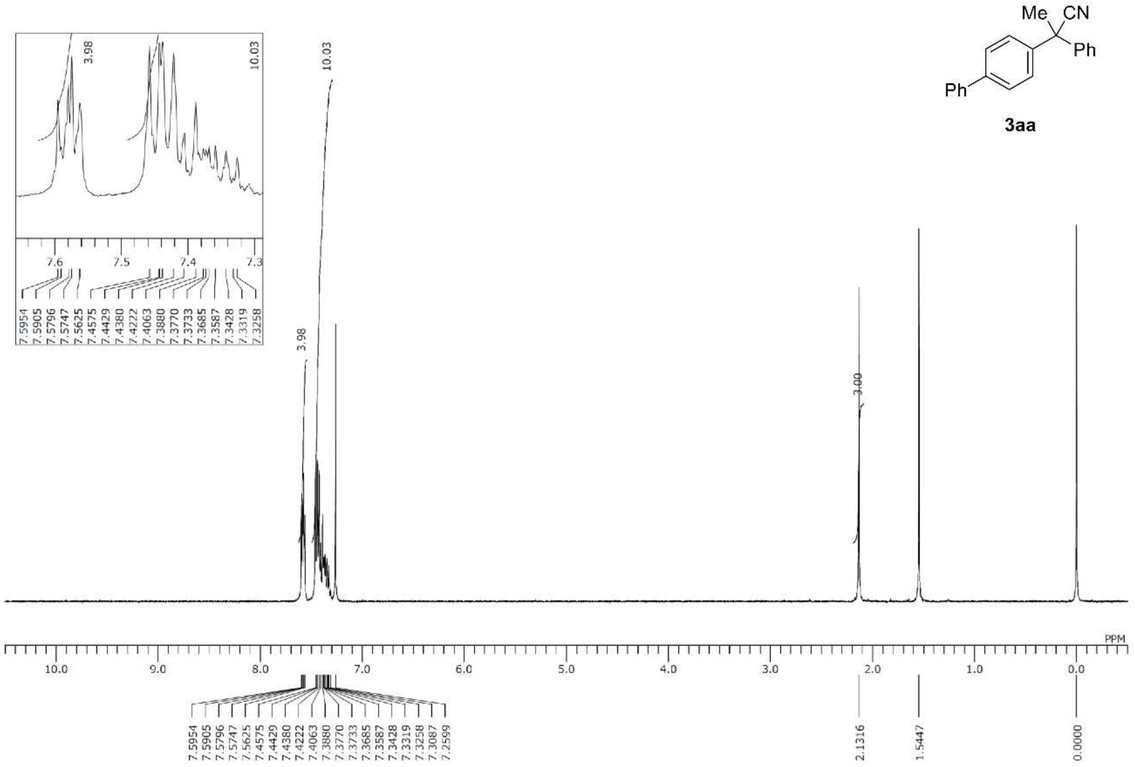

^{13}C NMR spectra of **3aa** (DMSO-d_6, 100 MHz)

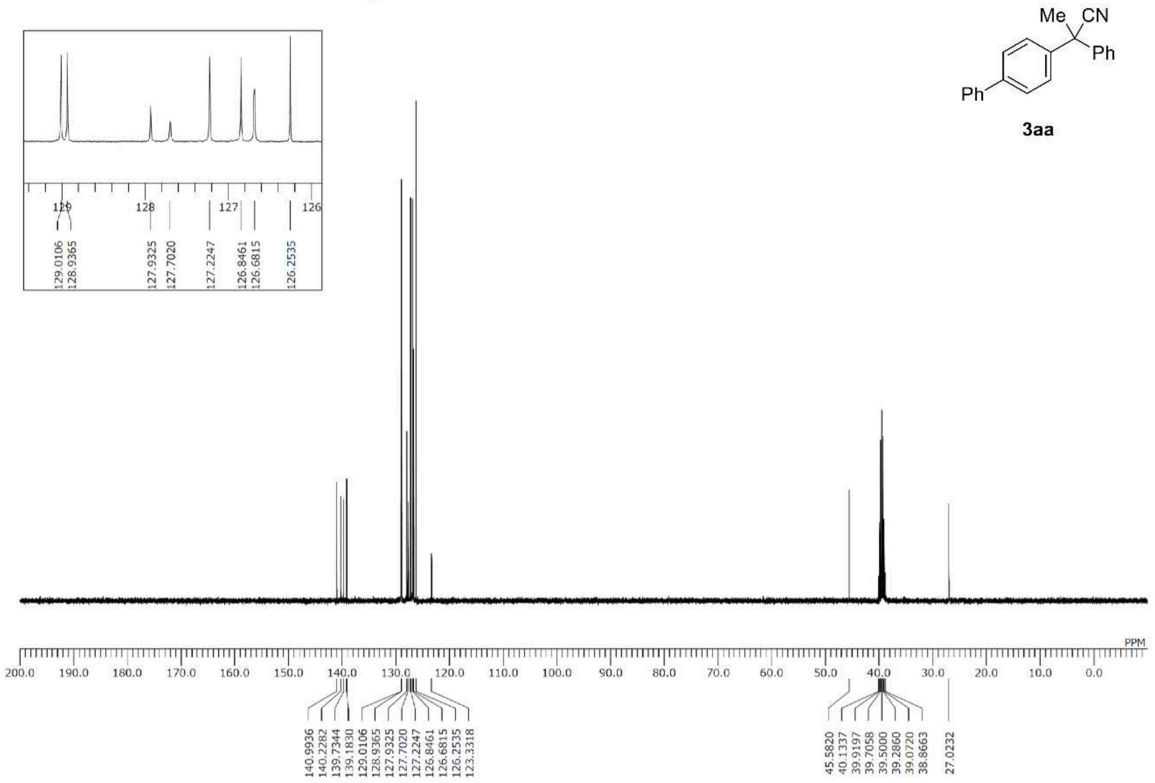

^{1}H NMR spectra of **3ba** ($CDCl_3$, 400 MHz)

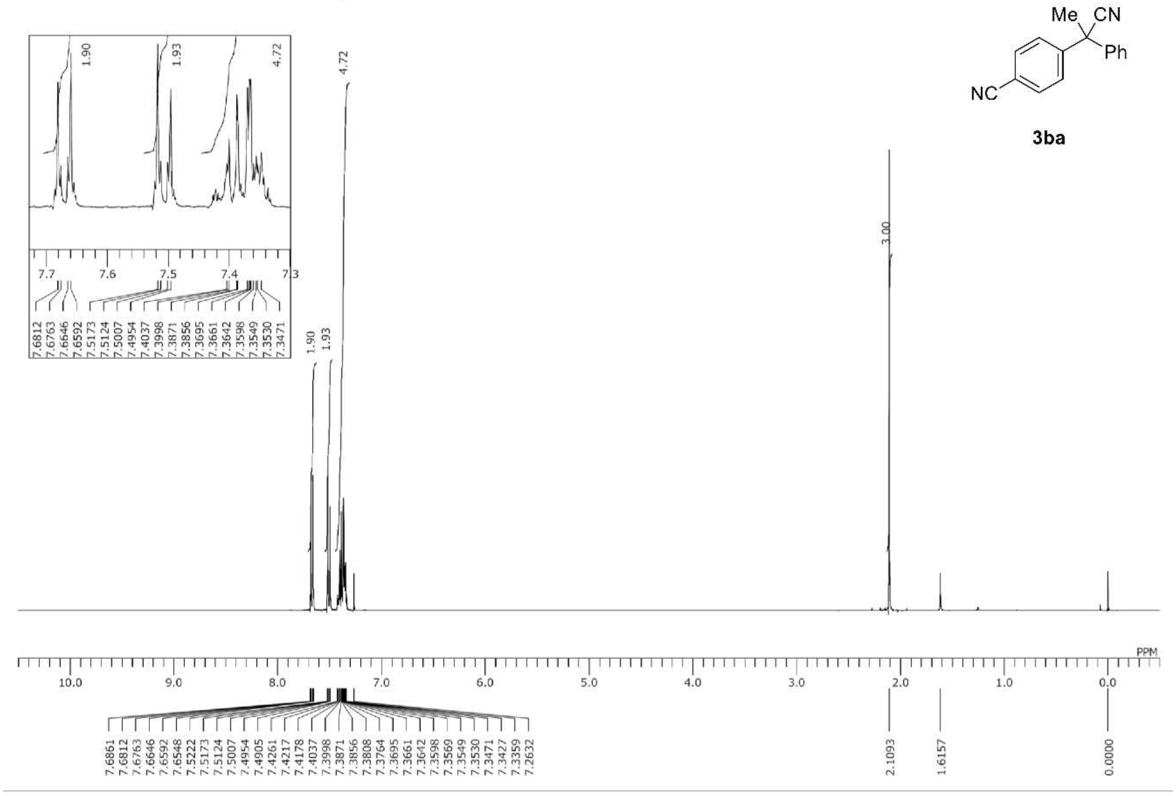

^{13}C NMR spectra of **3ba** ($CDCl_3$, 100 MHz)

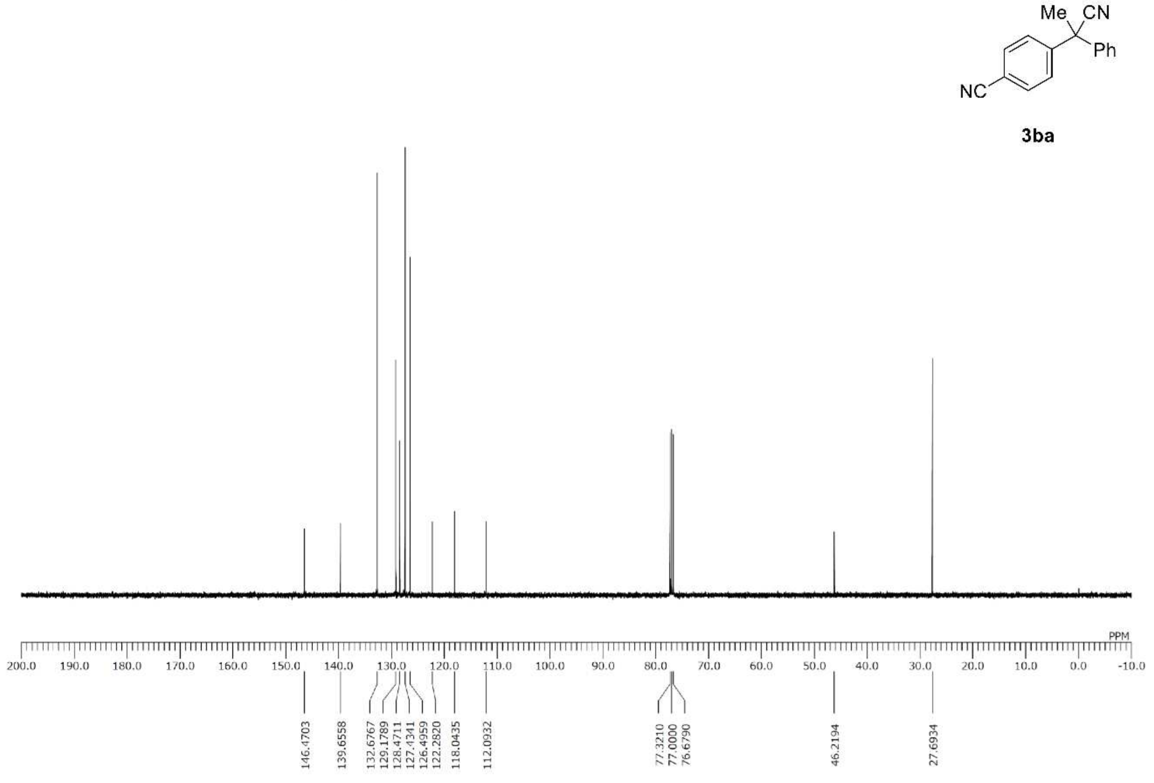

^{1}H NMR spectra of **3ca** ($CDCl_3$, 400 MHz)

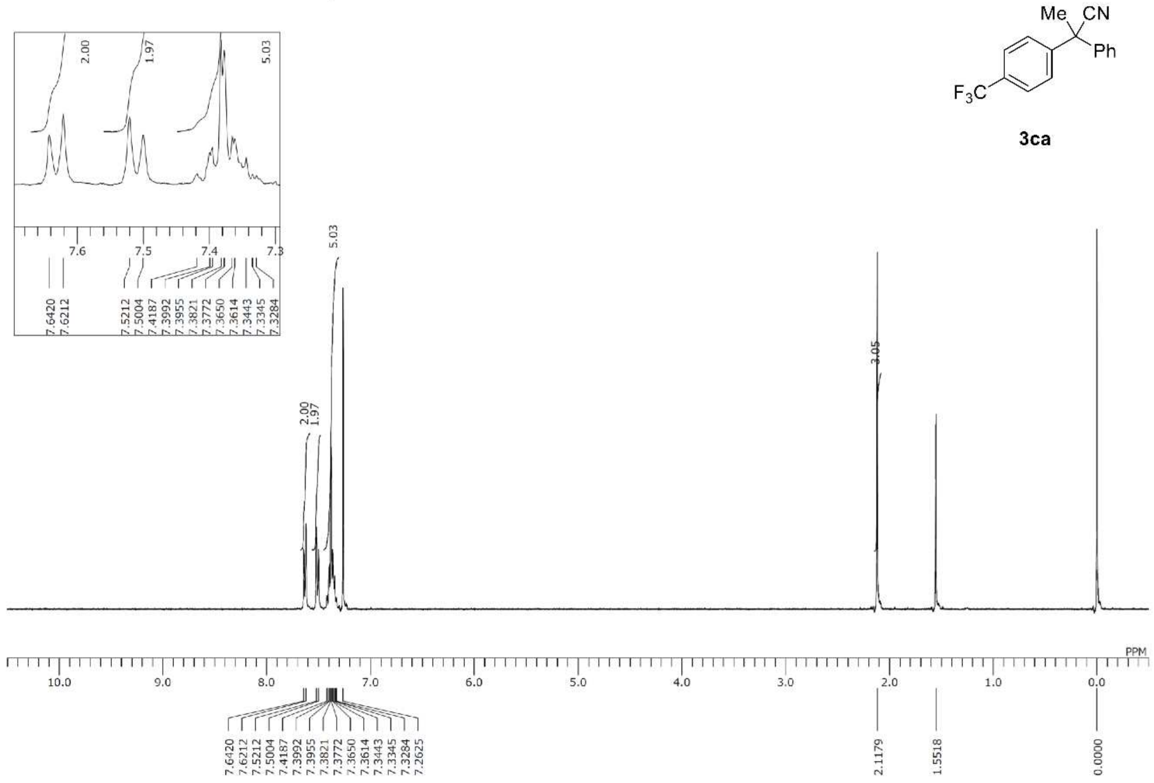

^{13}C NMR spectra of **3ca** ($CDCl_3$, 100 MHz)

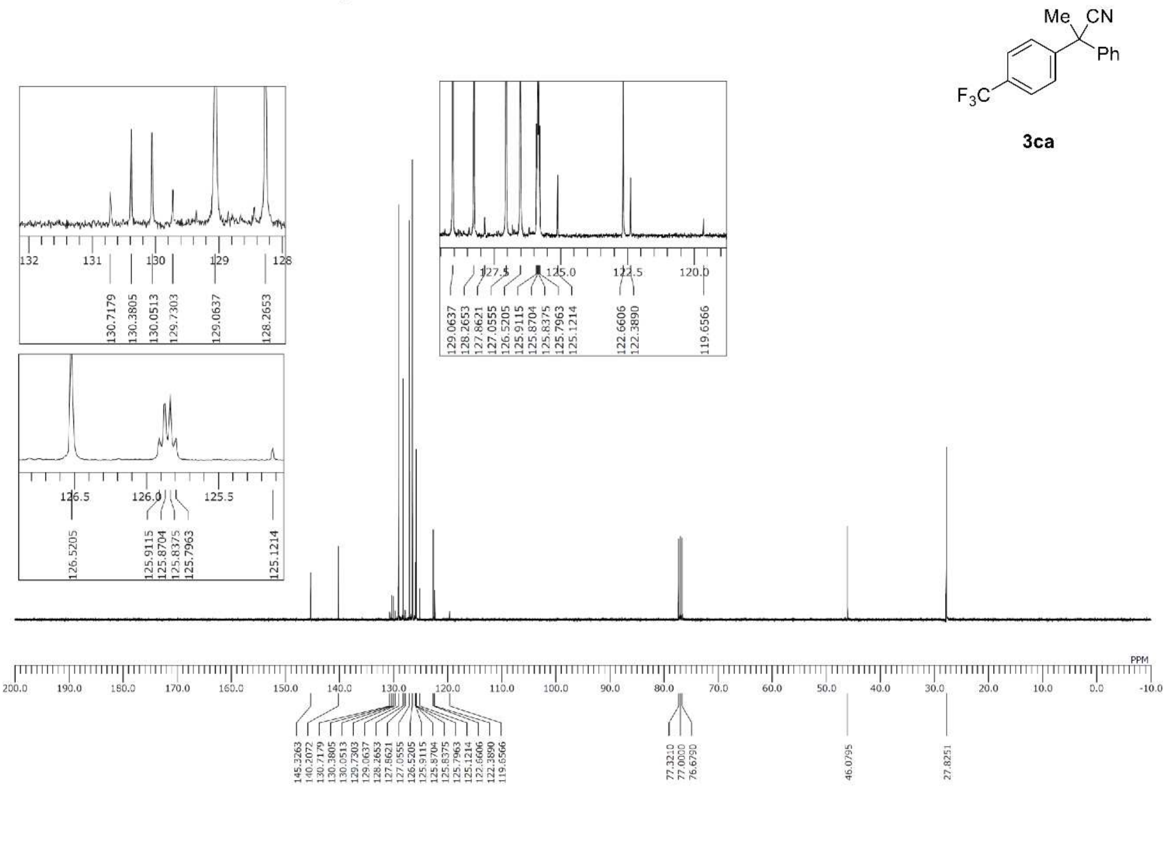

^{19}F NMR spectra of **3ca** ($CDCl_3$, 565 MHz)

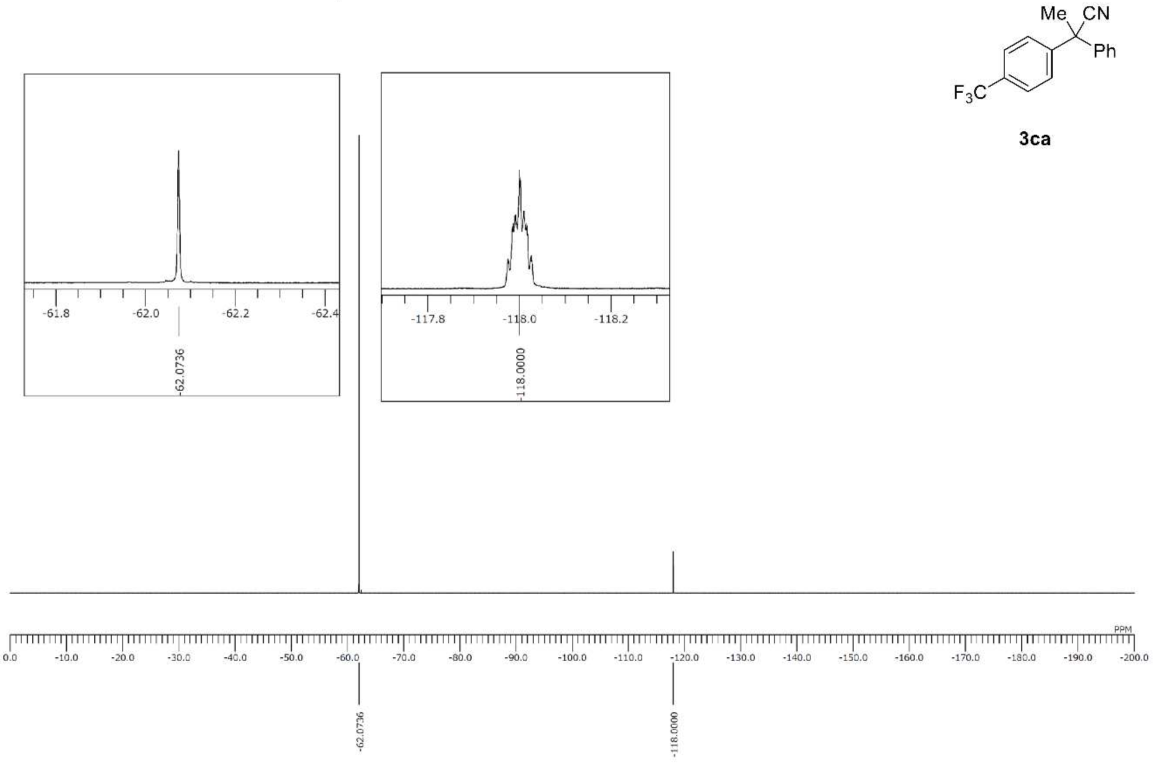

^{1}H NMR spectra of **3da** ($CDCl_3$, 400 MHz)

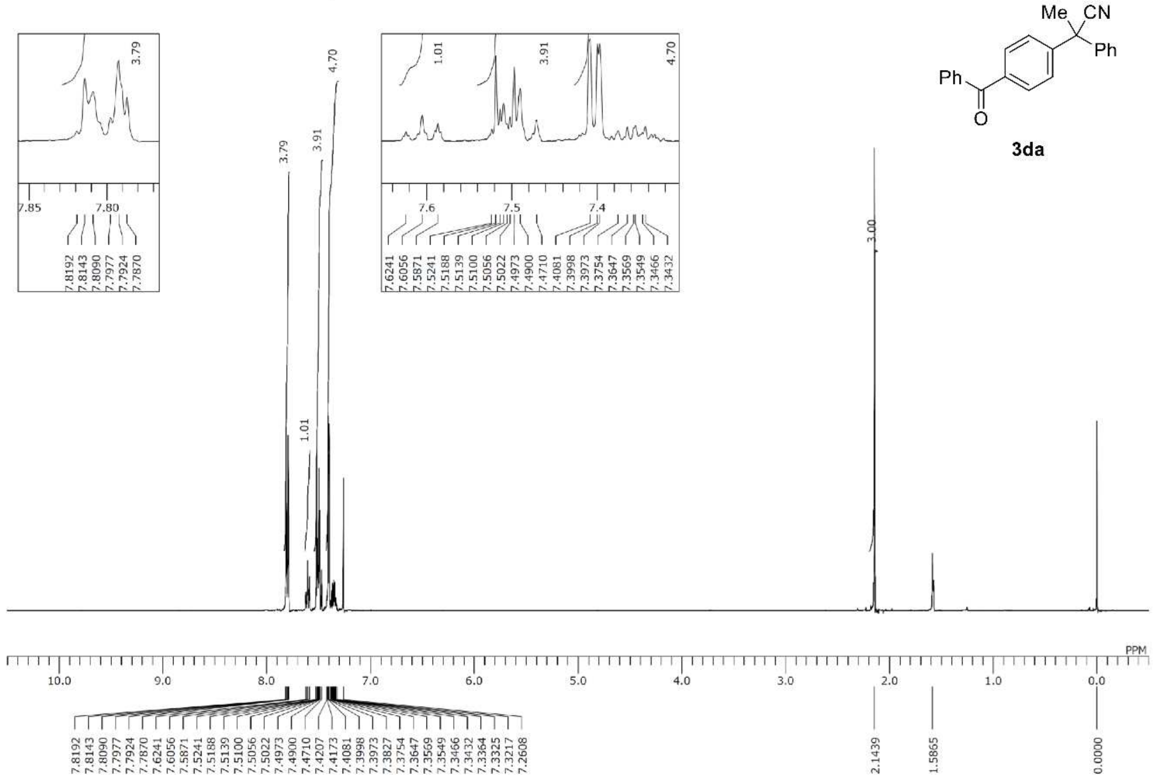

^{13}C NMR spectra of **3da** (DMSO-d_6, 100 MHz)

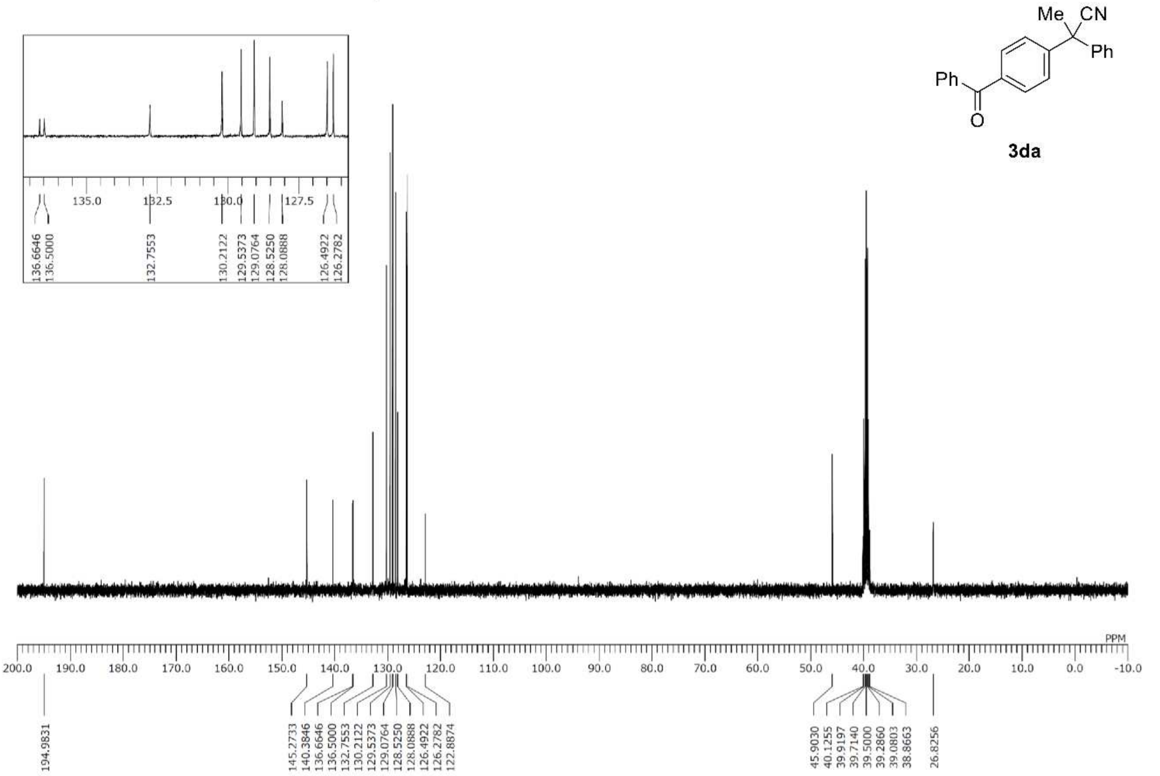

^{1}H NMR spectra of **3ea** ($CDCl_3$, 400 MHz)

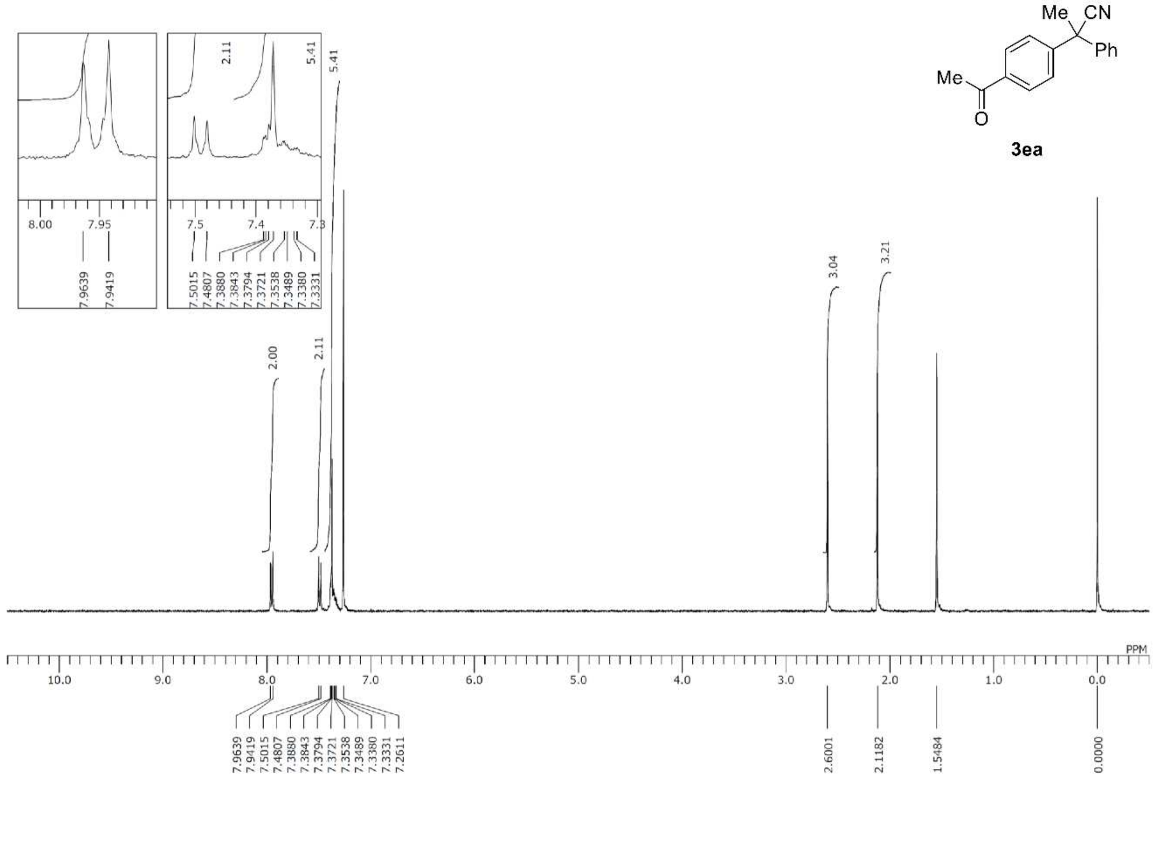

^{13}C NMR spectra of **3ea** ($CDCl_3$, 150 MHz)

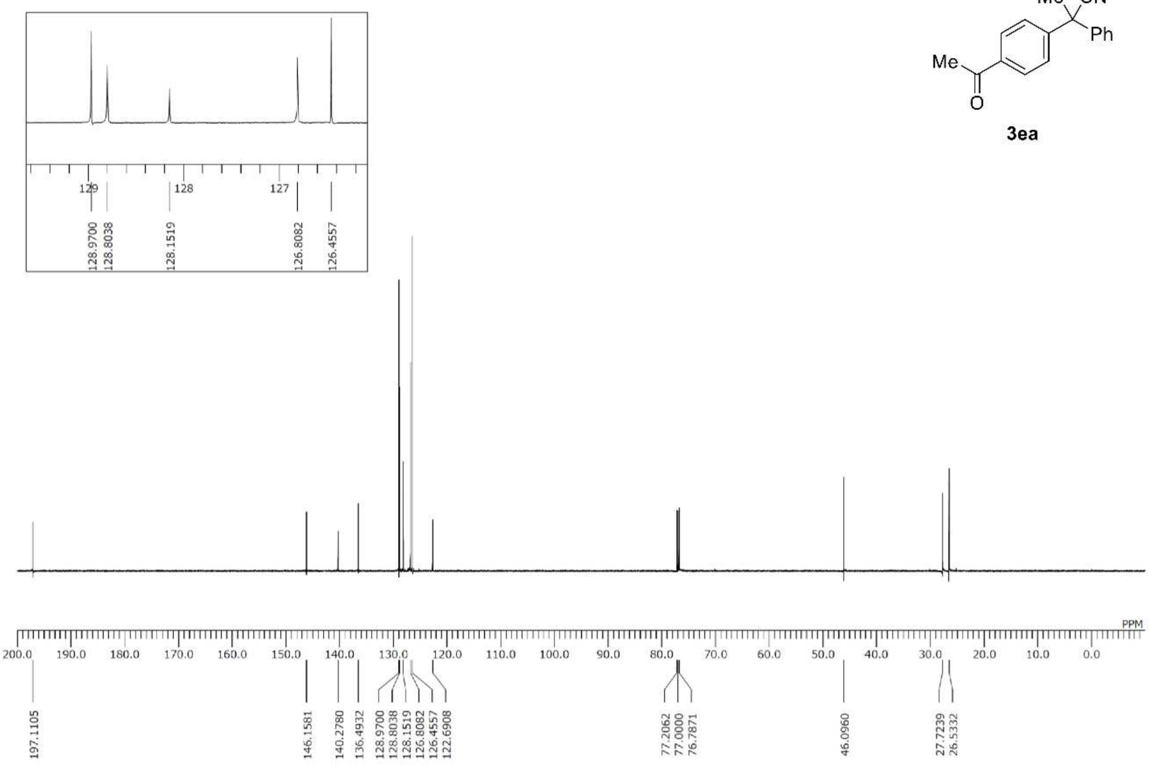

^{1}H NMR spectra of **3fa** ($CDCl_3$, 400 MHz)

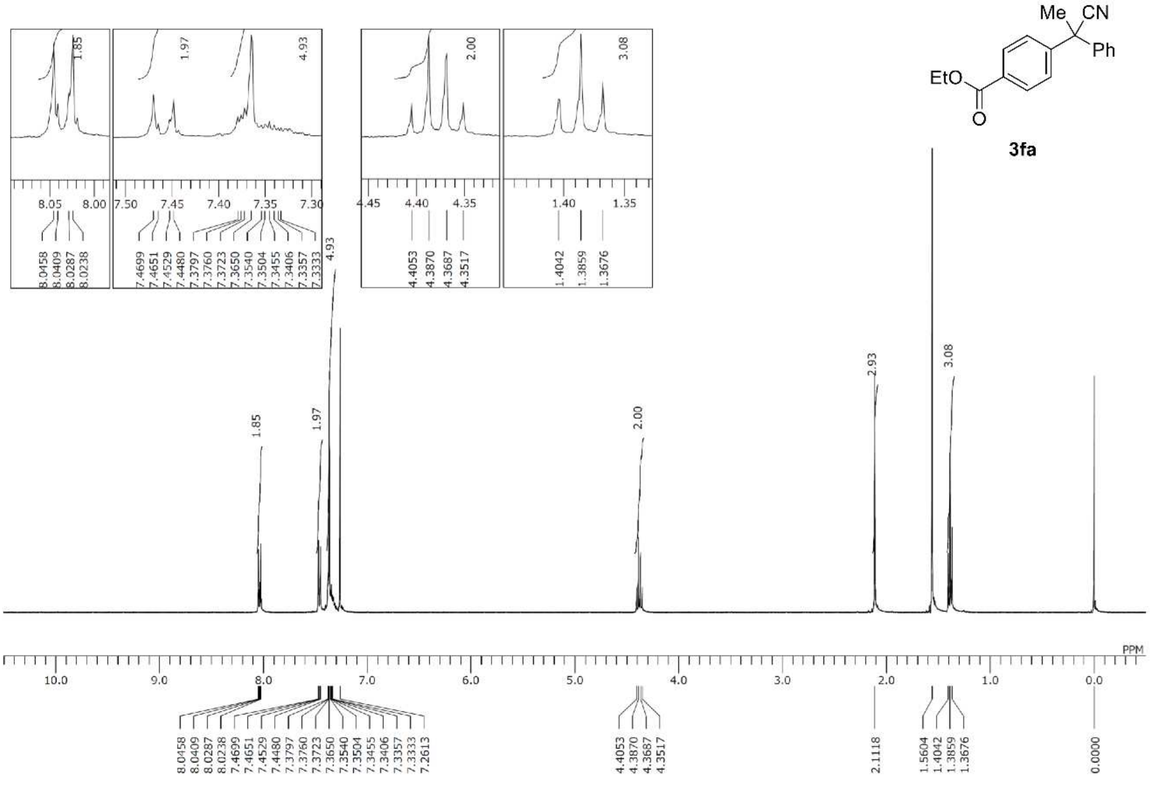

^{13}C NMR spectra of **3fa** ($CDCl_3$, 100 MHz)

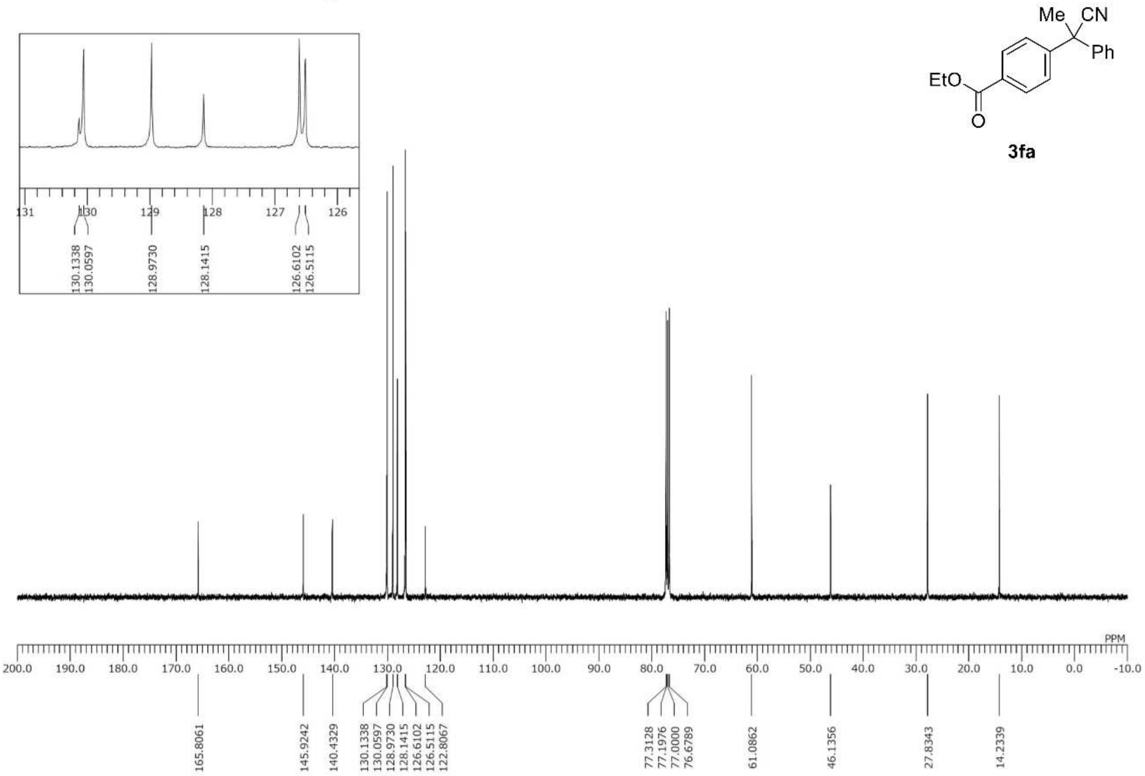

^{1}H NMR spectra of **3ga** ($CDCl_3$, 400 MHz)

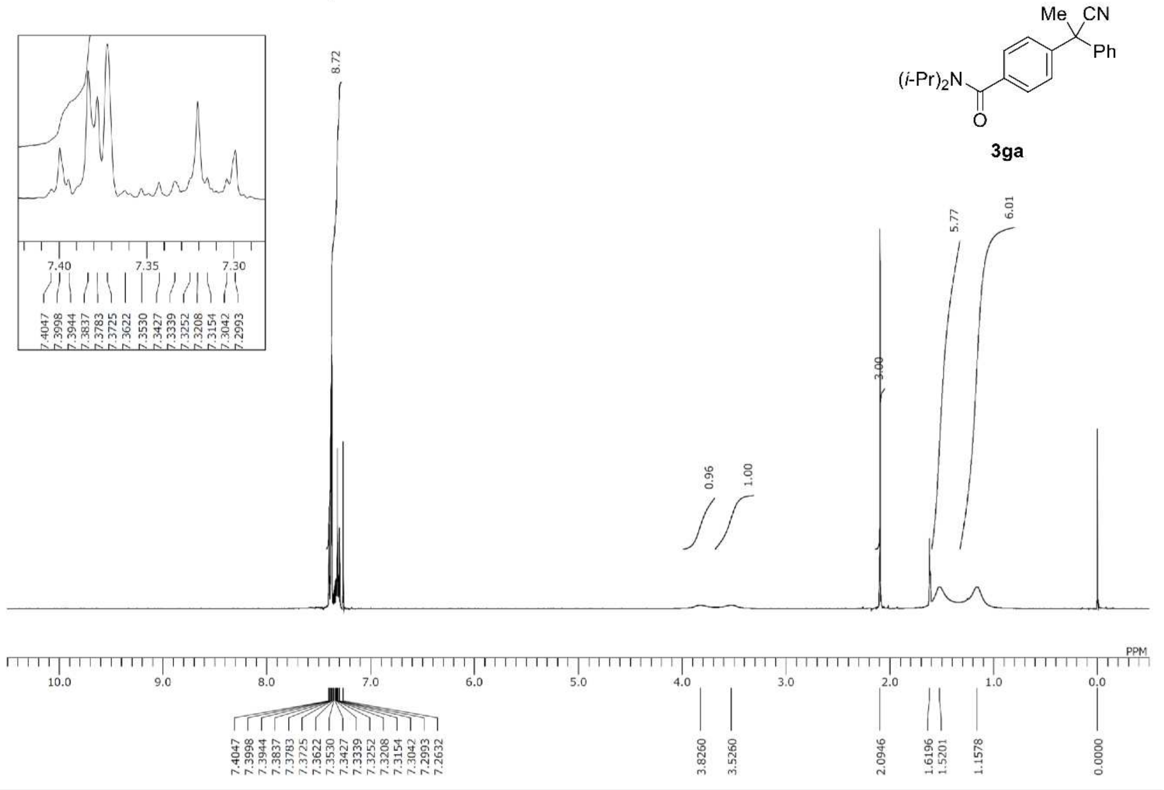

^{13}C NMR spectra of **3ga** ($CDCl_3$, 150 MHz)

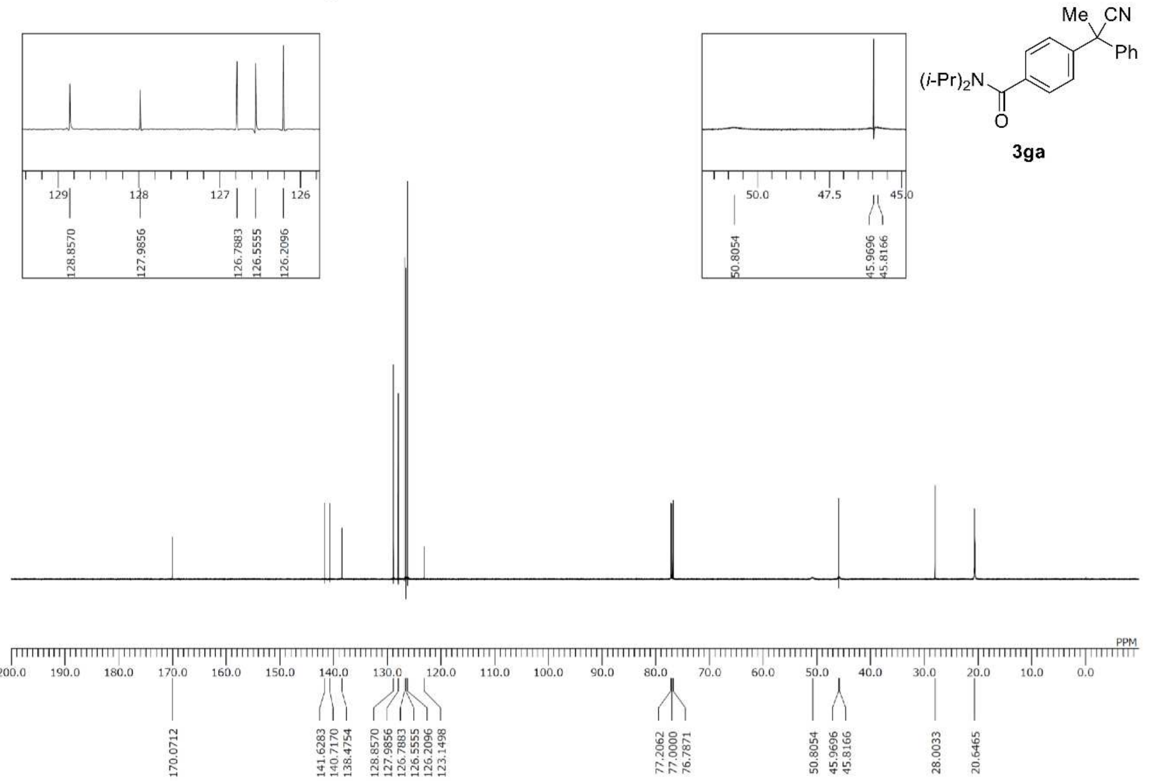

^{1}H NMR spectra of **3ha** ($CDCl_3$, 400 MHz)

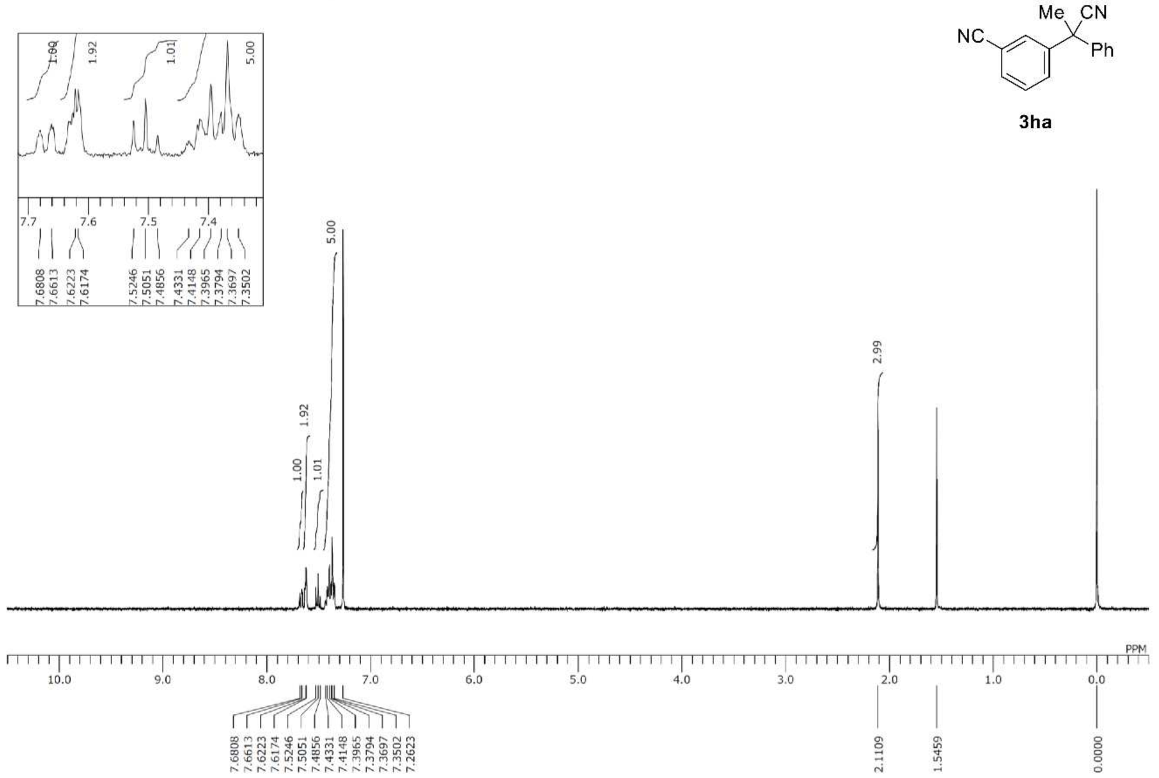

^{13}C NMR spectra of **3ha** ($CDCl_3$, 100 MHz)

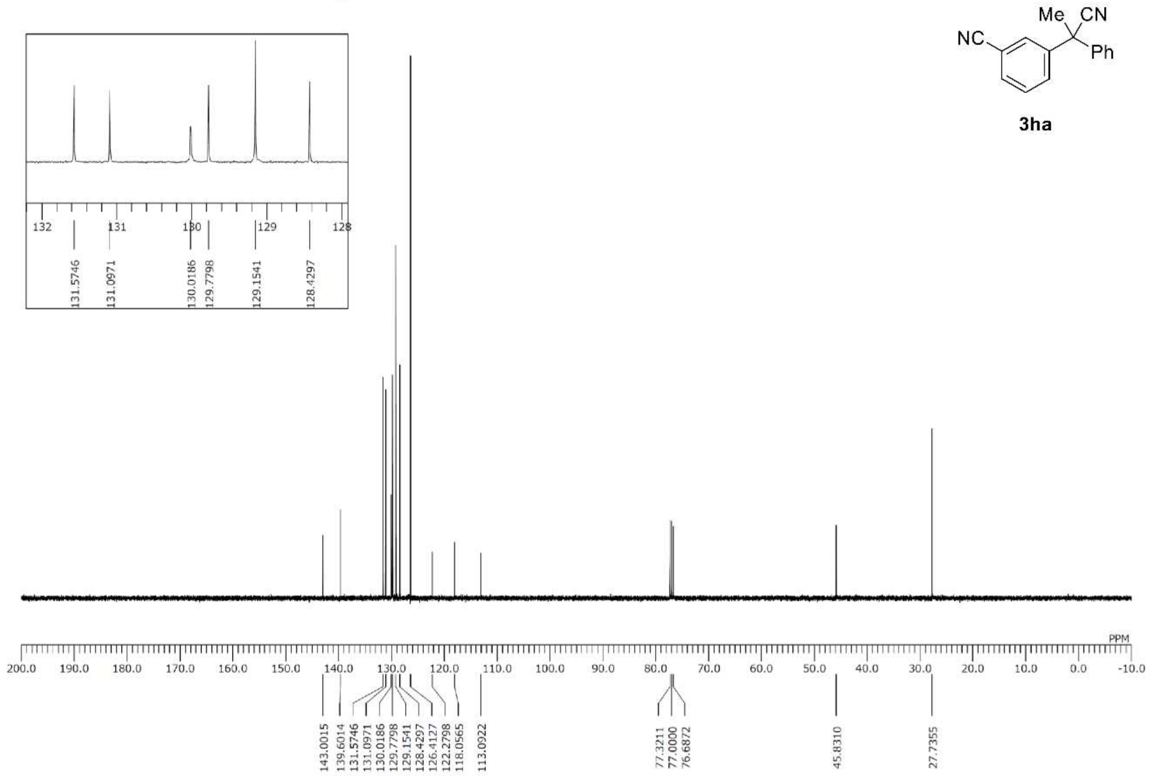

^{1}H NMR spectra of **3ia** ($CDCl_3$, 400 MHz)

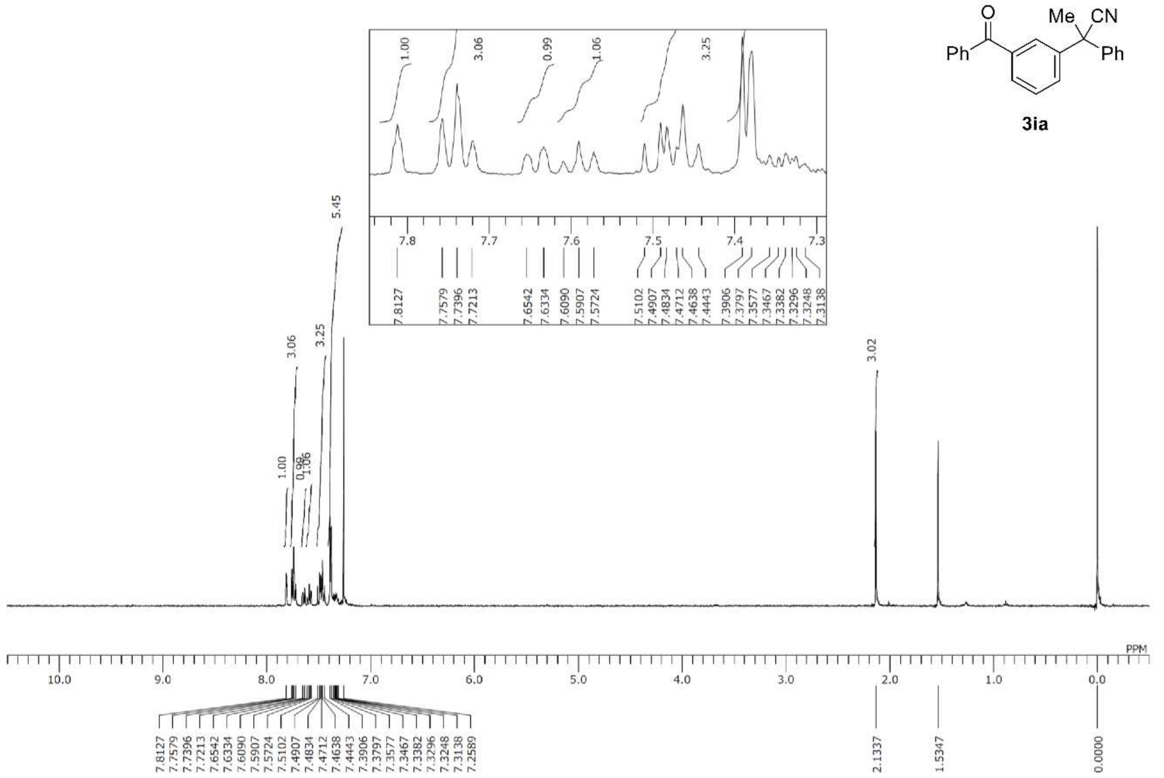

^{13}C NMR spectra of **3ia** ($CDCl_3$, 100 MHz)

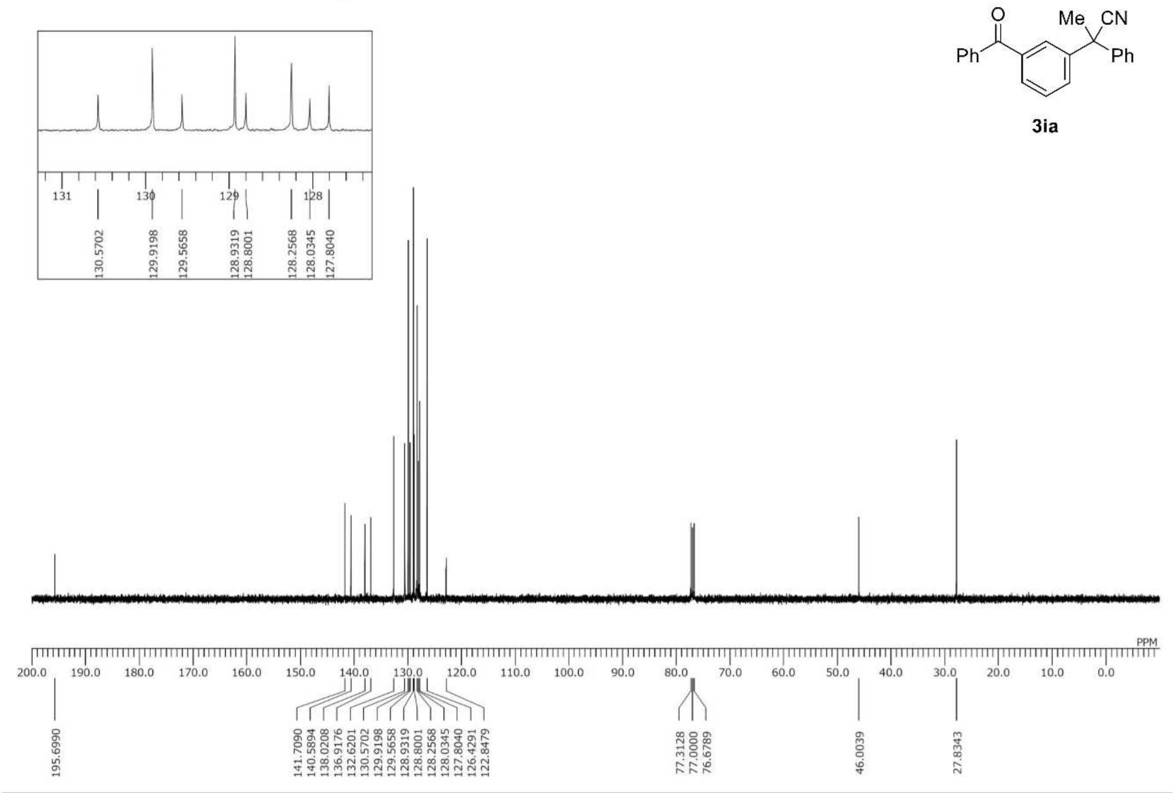

^{1}H NMR spectra of **3ja** ($CDCl_3$, 400 MHz)

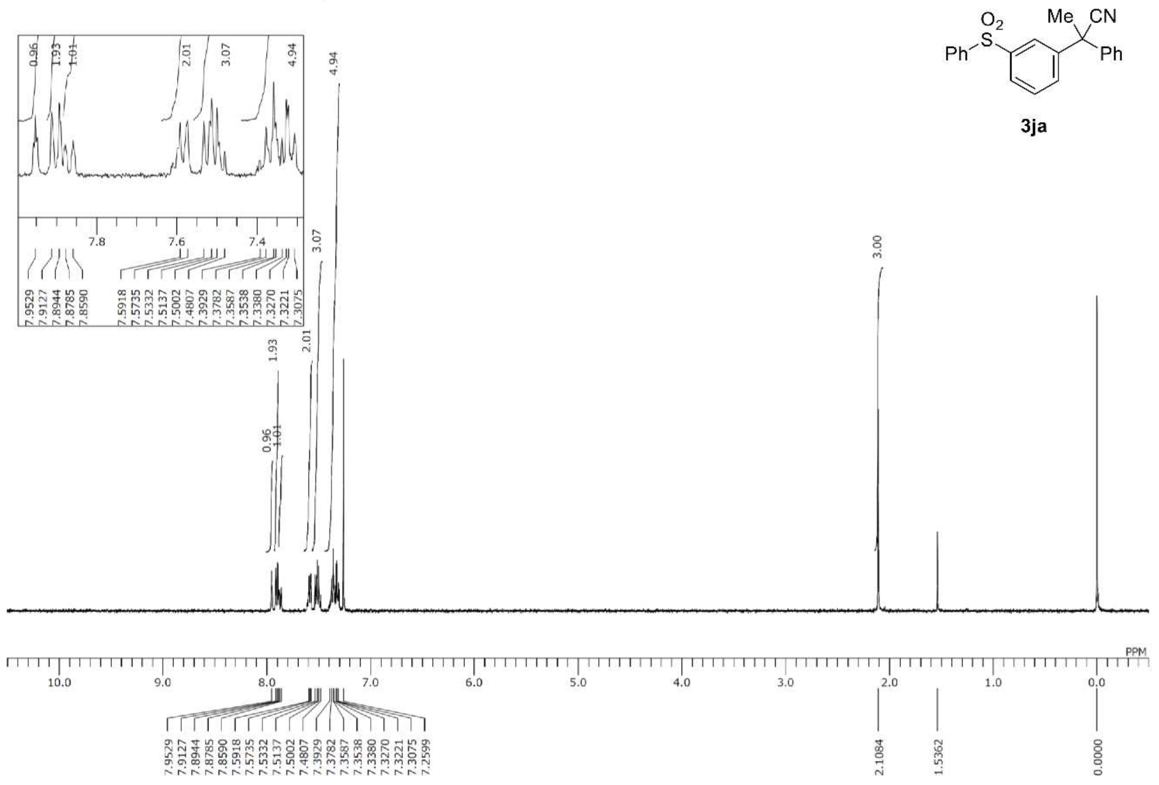

^{13}C NMR spectra of **3ja** ($CDCl_3$, 100 MHz)

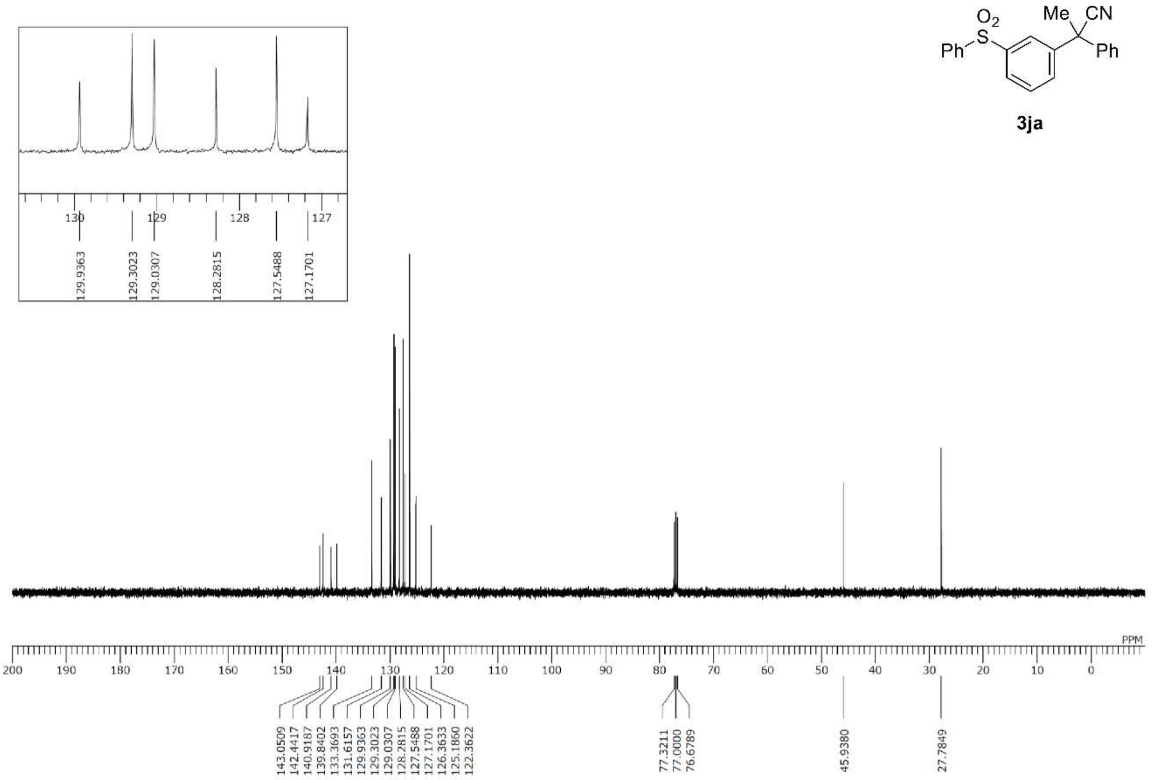

^{1}H NMR spectra of **3ka** ($CDCl_3$, 400 MHz)

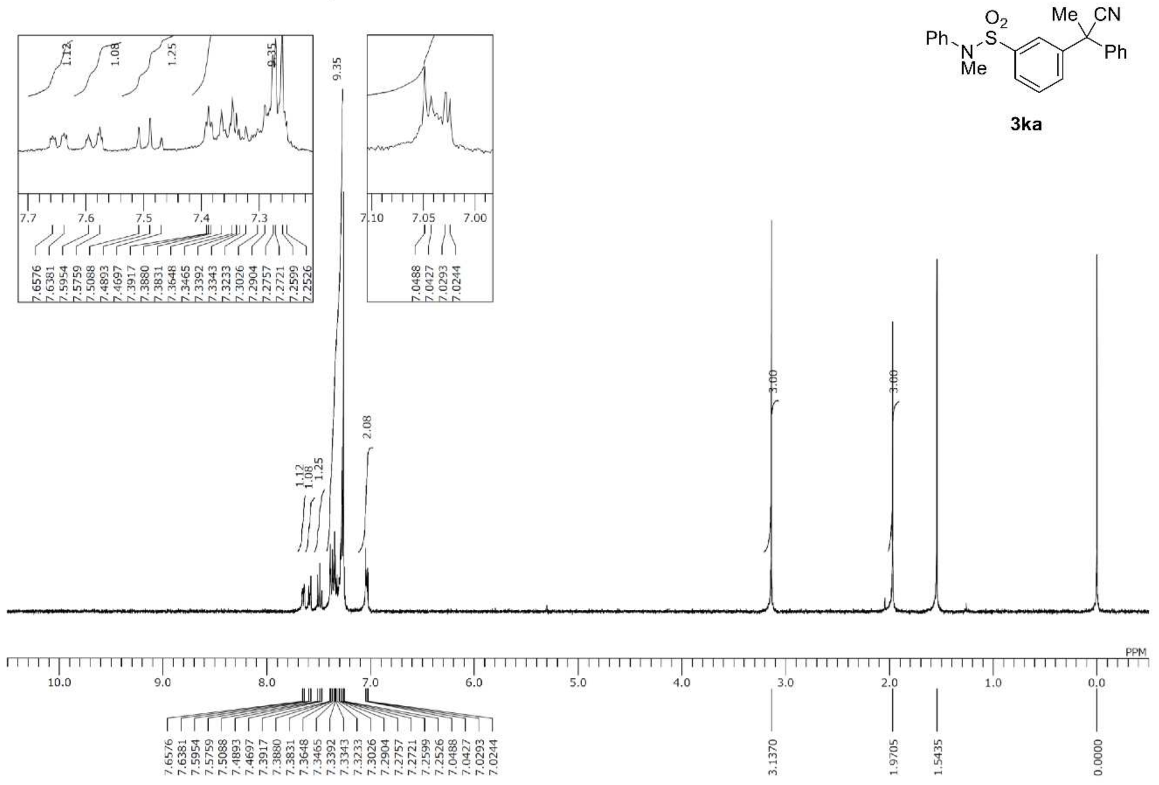

^{13}C NMR spectra of **3ka** ($CDCl_3$, 100 MHz)

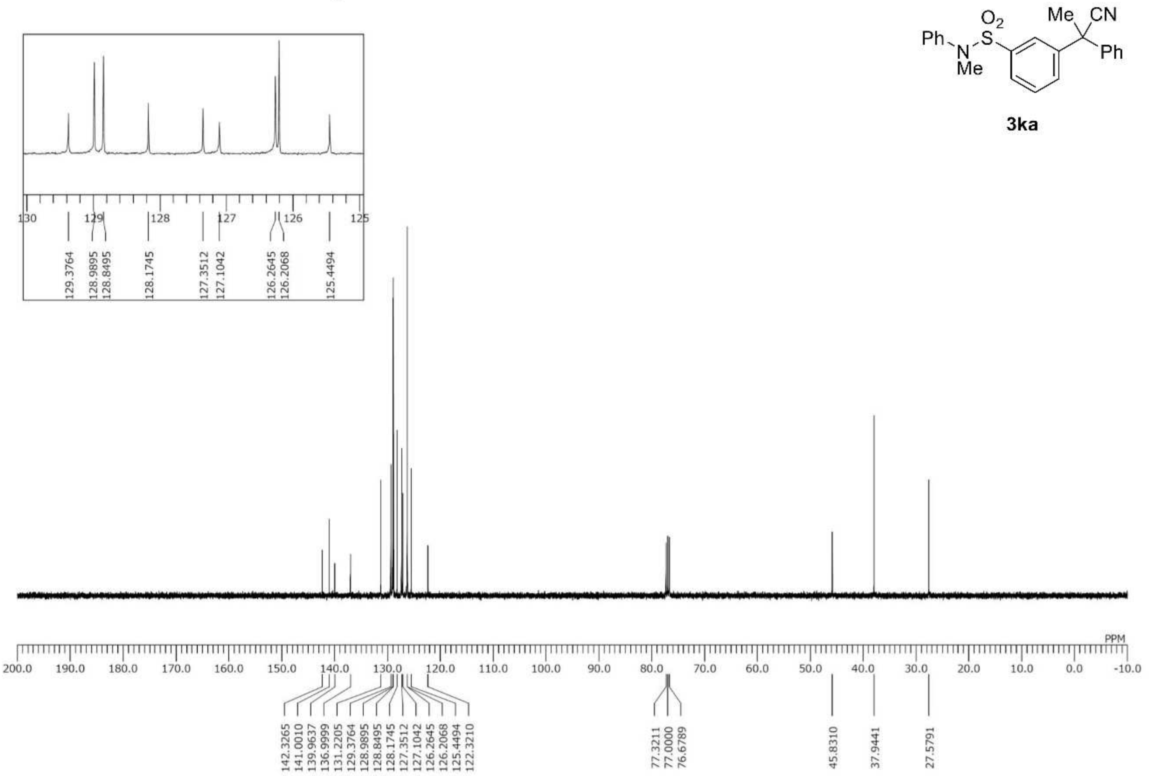

^{1}H NMR spectra of **3la** ($CDCl_3$, 400 MHz)

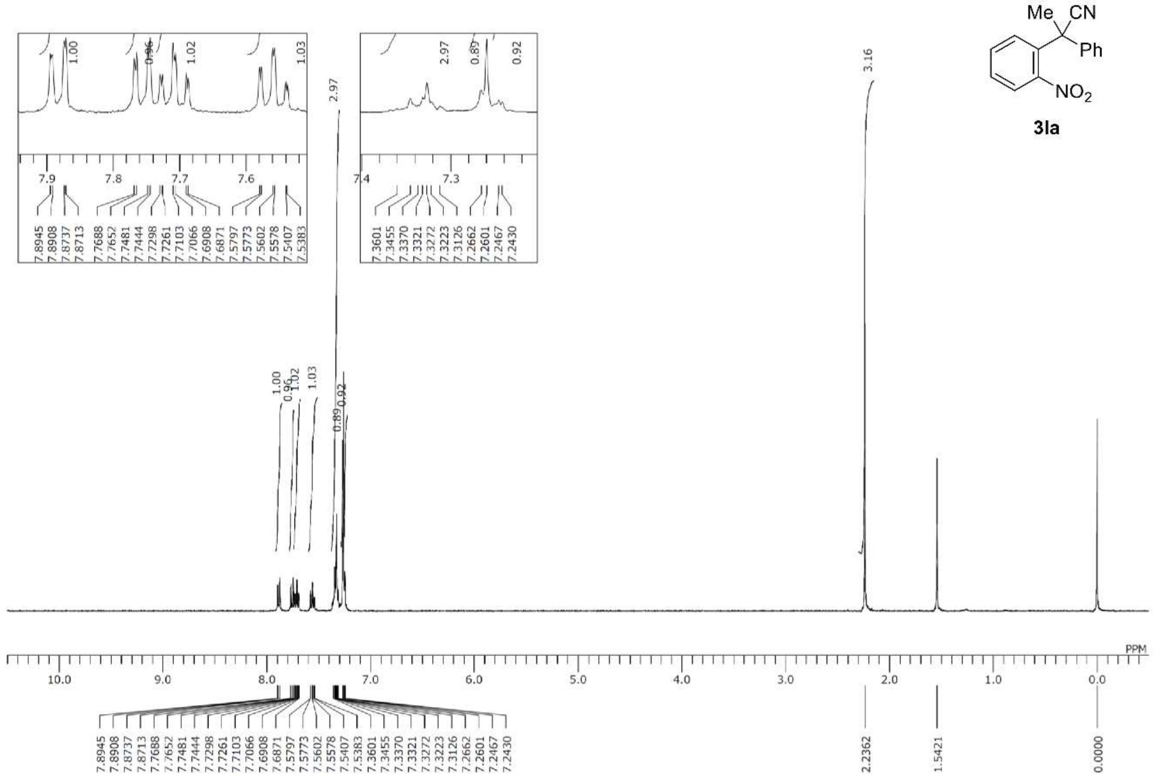

^{13}C NMR spectra of **3la** ($CDCl_3$, 150 MHz)

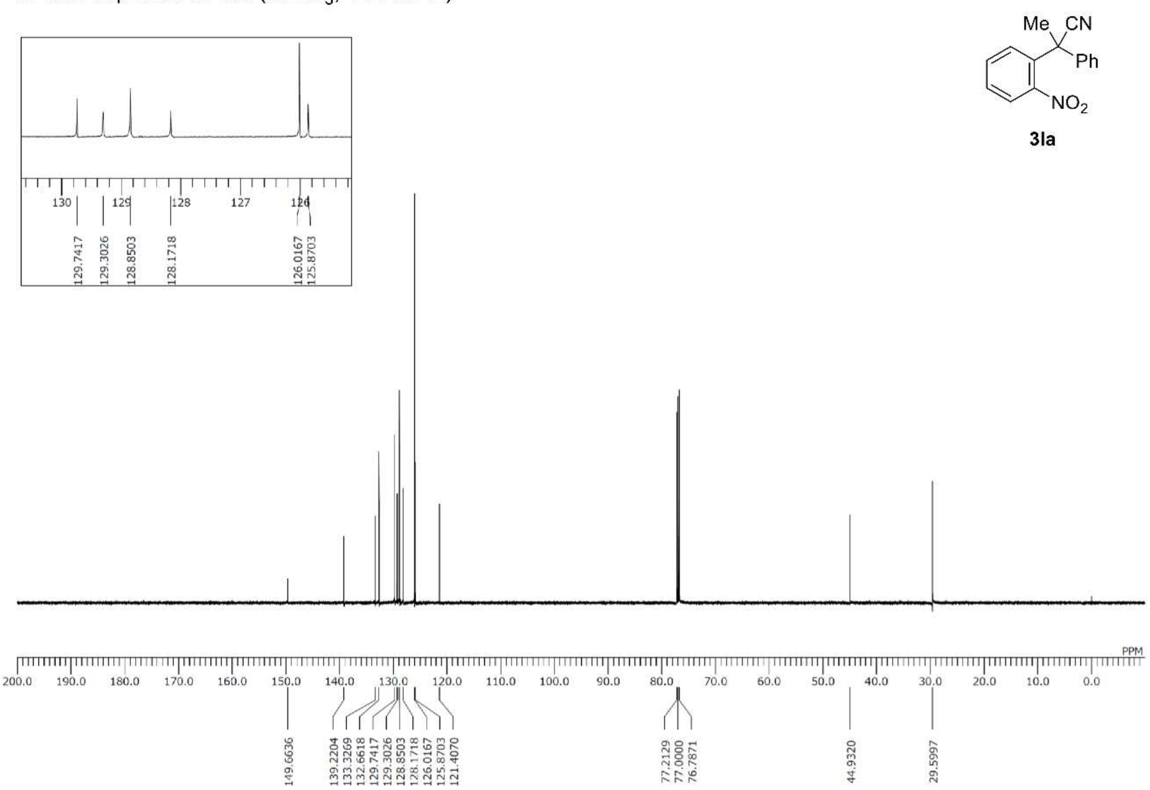

^{1}H NMR spectra of **3ma** ($CDCl_3$, 400 MHz)

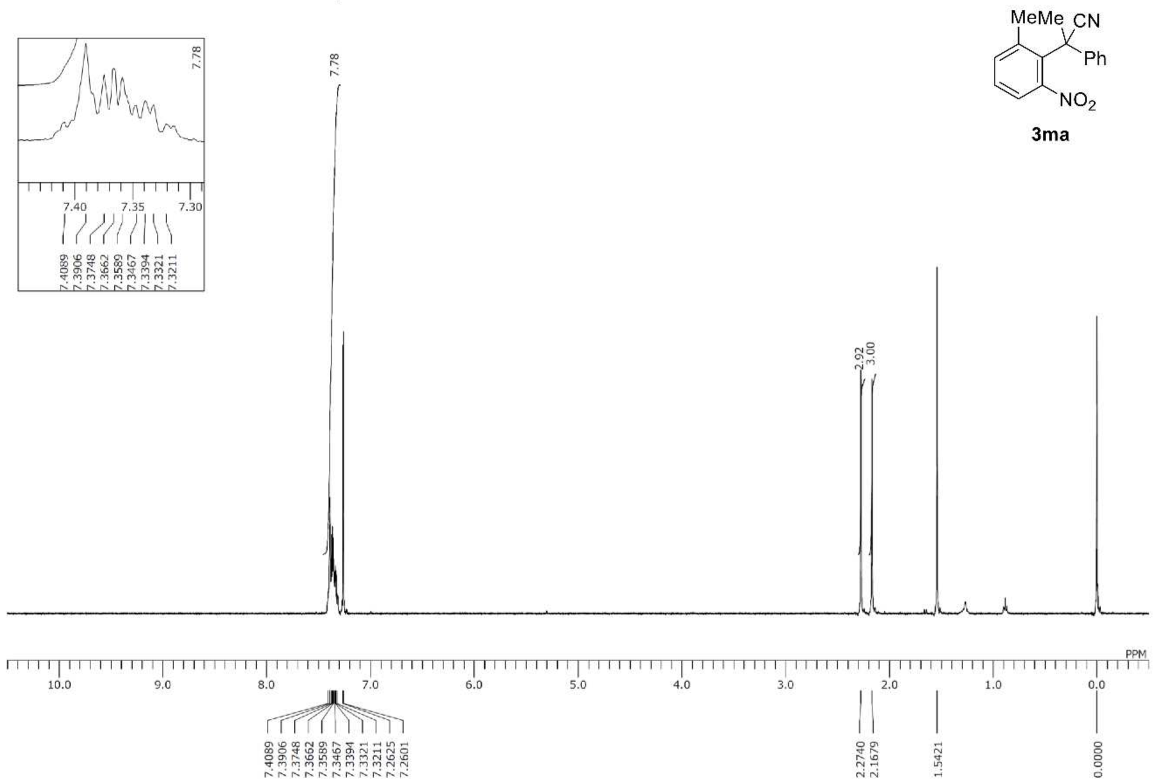

^{13}C NMR spectra of **3ma** ($CDCl_3$, 100 MHz)

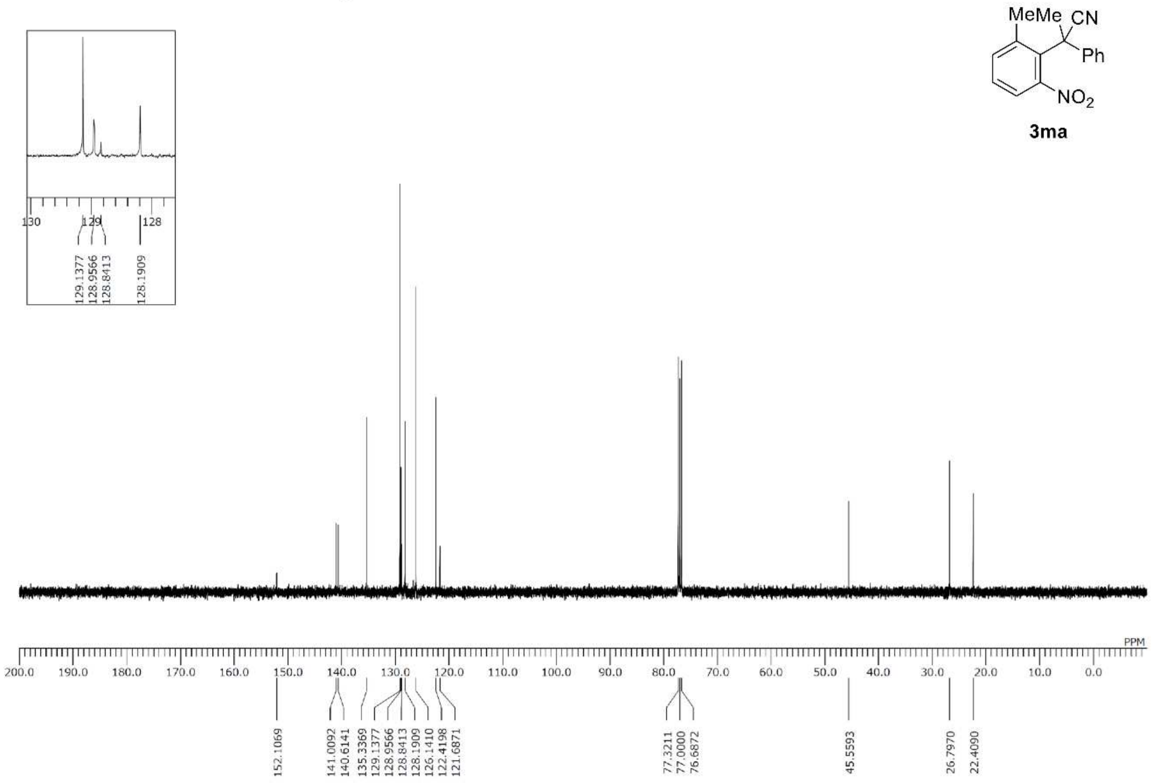

^{1}H NMR spectra of **3na** ($CDCl_3$, 400 MHz)

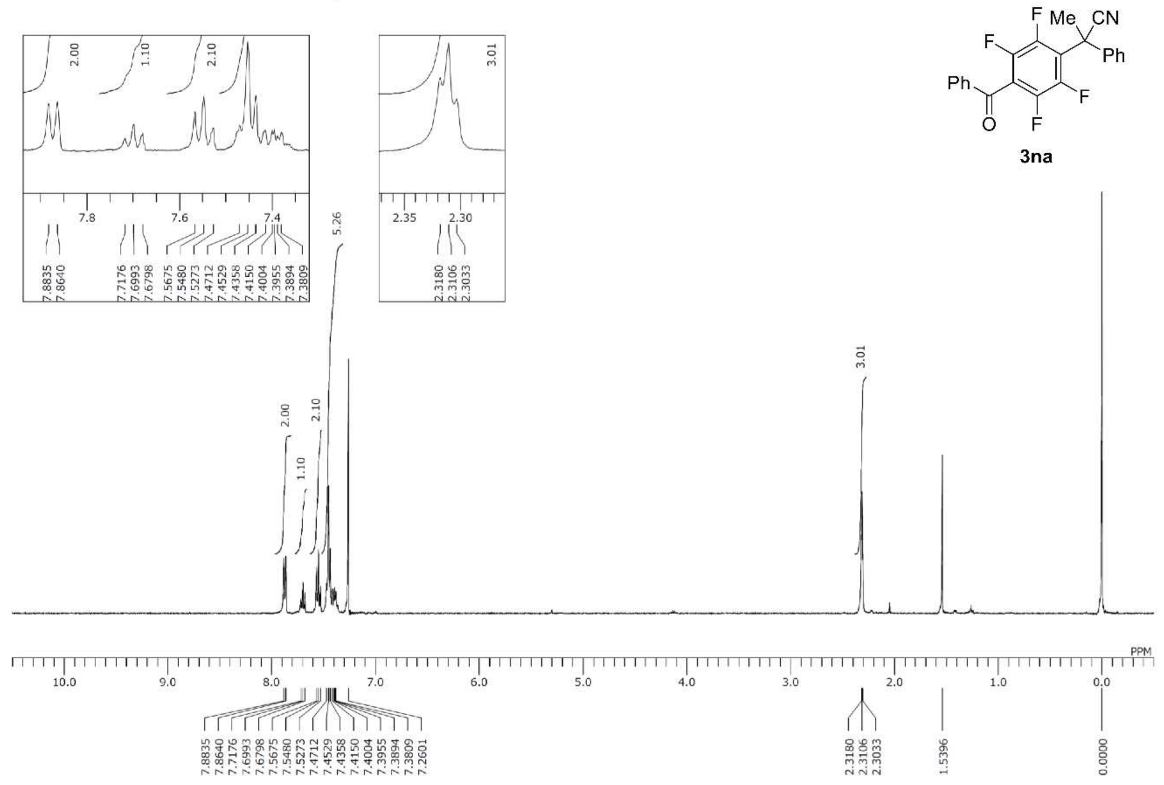

^{13}C NMR spectra of **3na** ($CDCl_3$, 150 MHz)

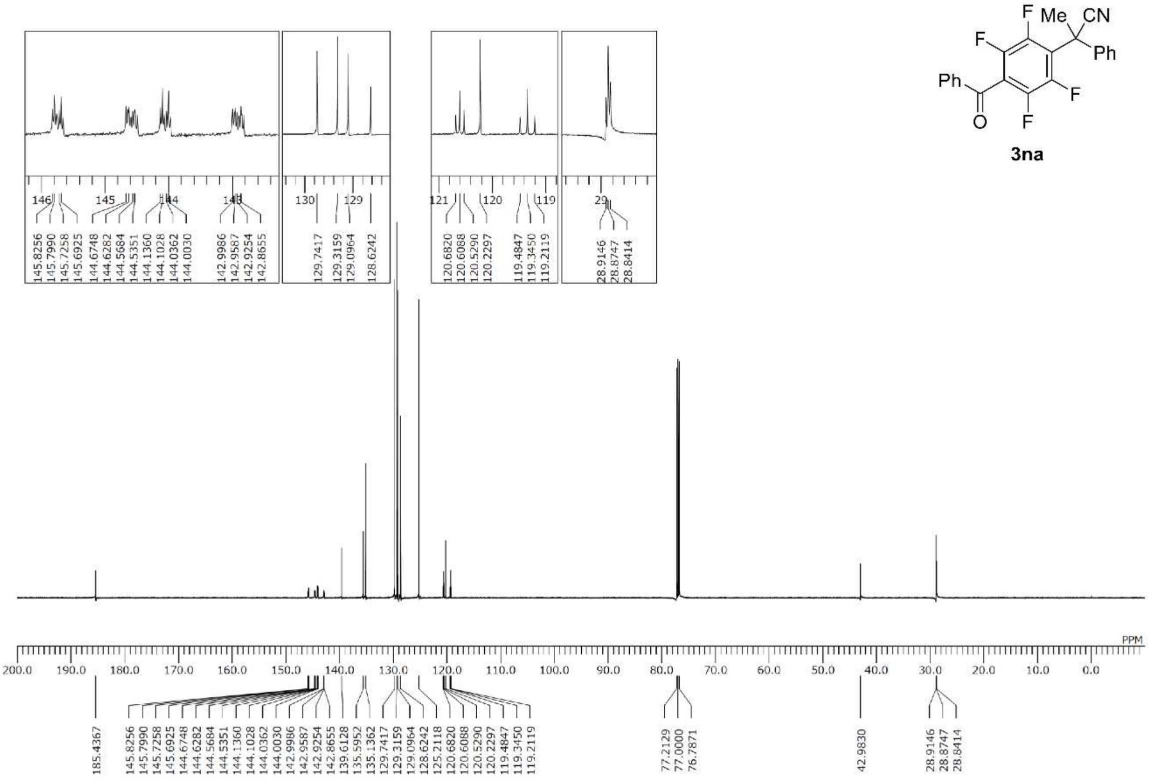

^{19}F NMR spectra of **3na** ($CDCl_3$, 565 MHz)

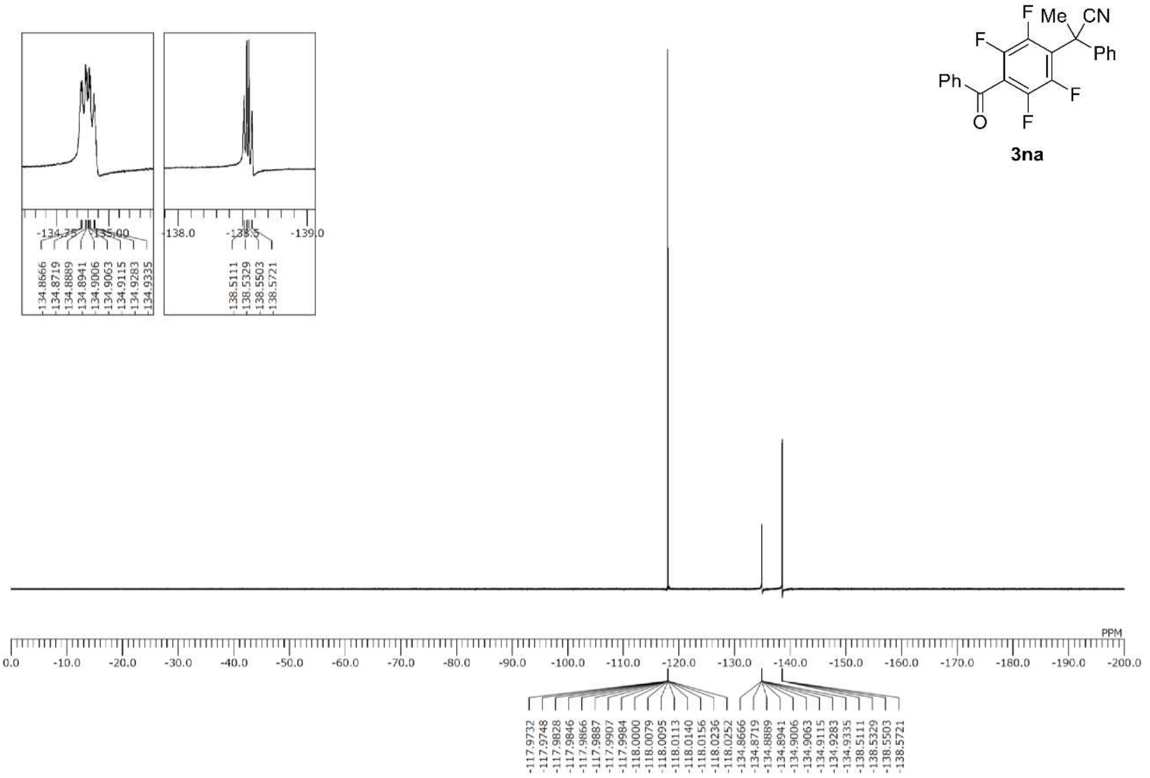

^{1}H NMR spectra of **3oa** ($CDCl_3$, 400 MHz)

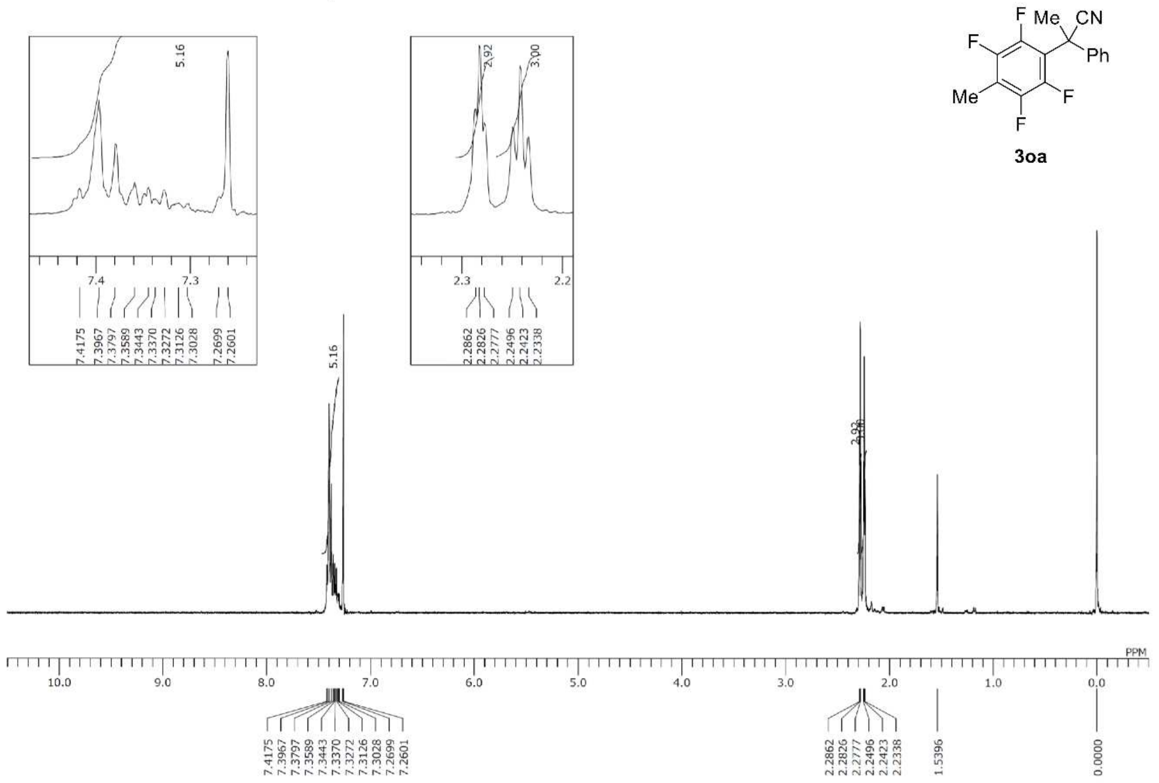

^{13}C NMR spectra of **3oa** ($CDCl_3$, 150 MHz)

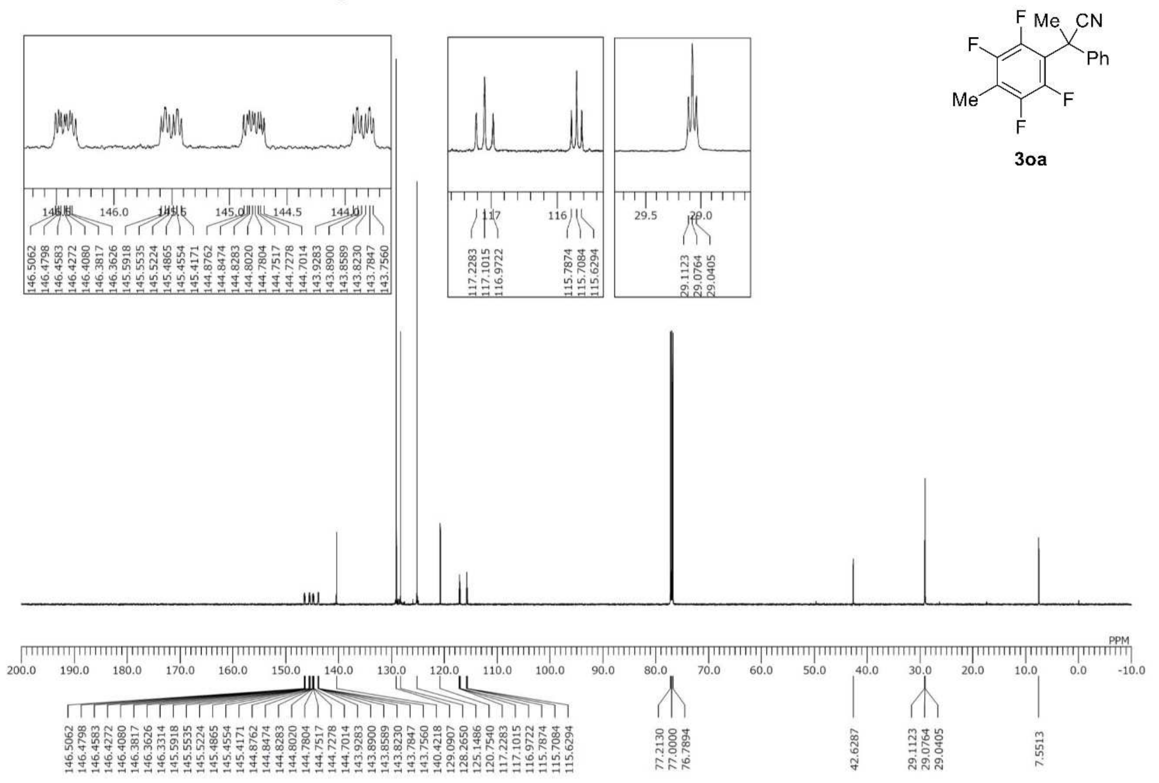

^{19}F NMR spectra of **3oa** ($CDCl_3$, 565 MHz)

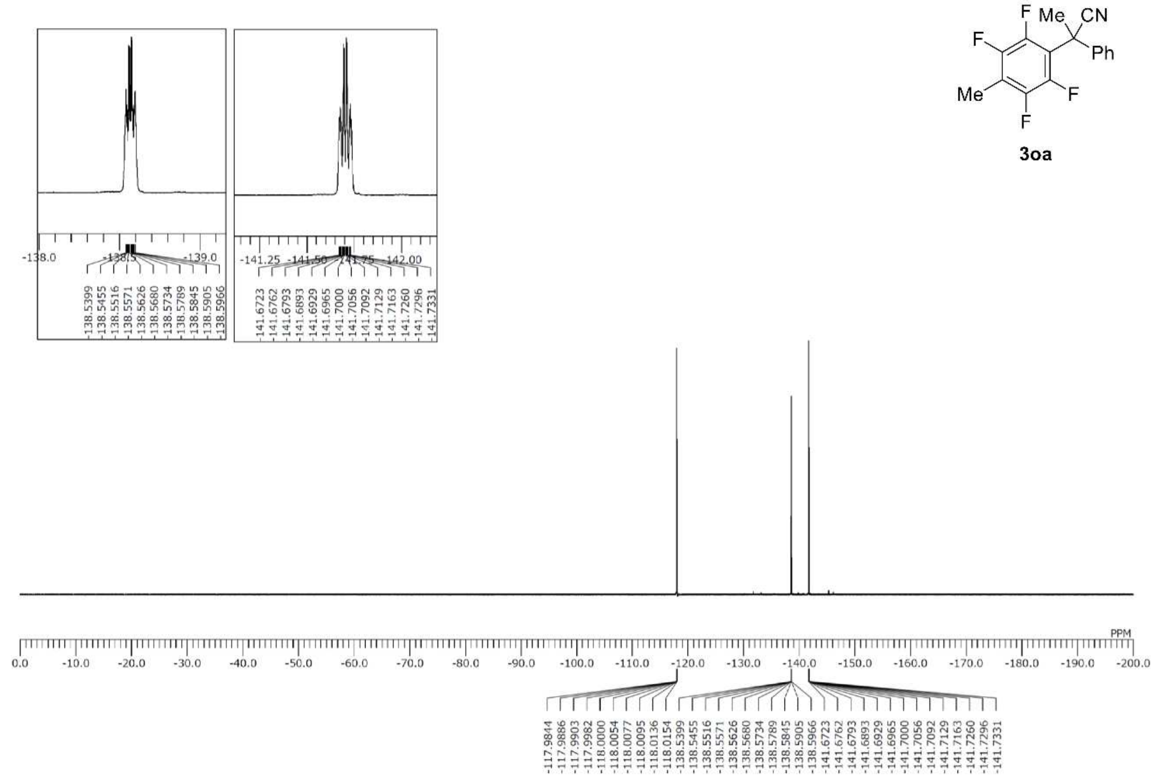

^{19}F NMR spectra of a diastereomeric mixture of **3pa** ($CDCl_3$, 376 MHz)

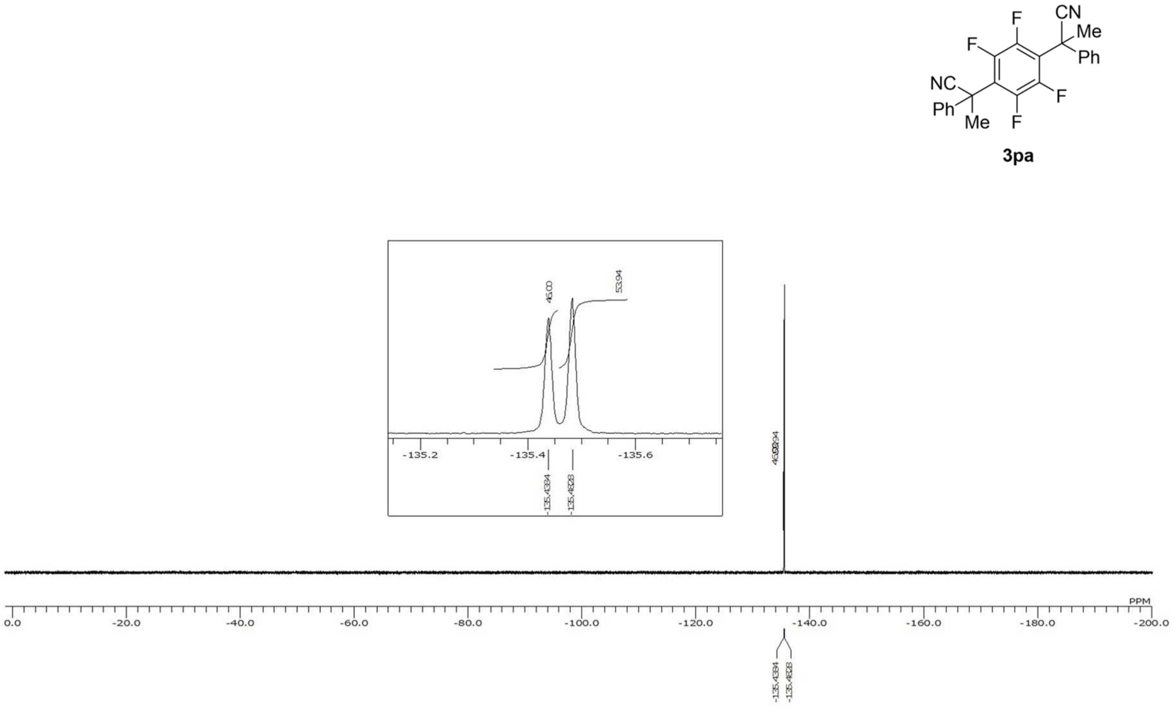

^{1}H NMR spectra of **3pa (Isomer A)** ($CDCl_3$, 600 MHz)

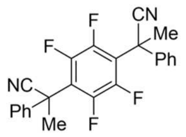

3pa

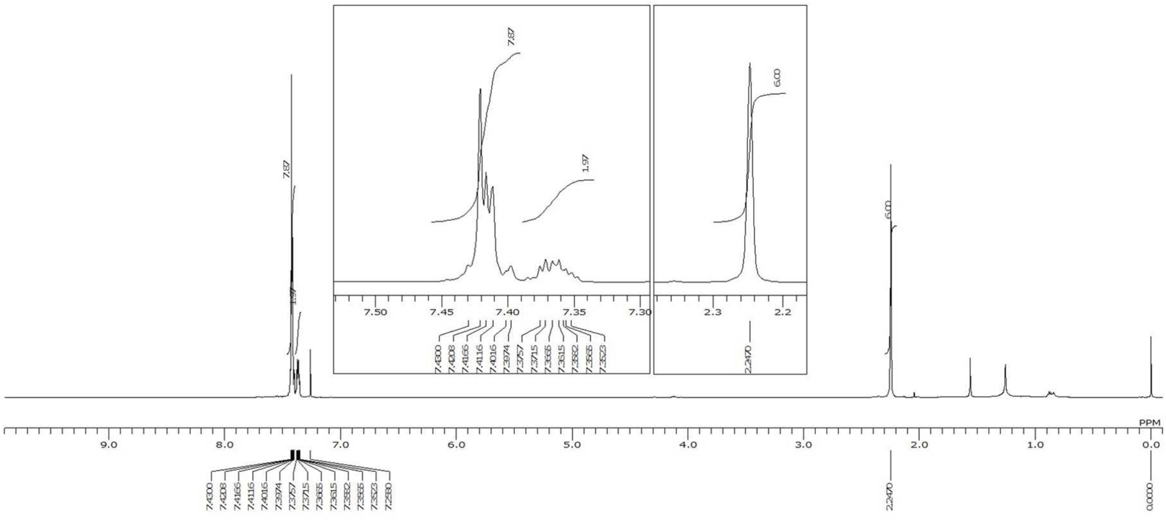

^{13}C NMR spectra of **3pa (Isomer A)** ($CDCl_3$, 150 MHz)

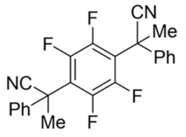

3pa

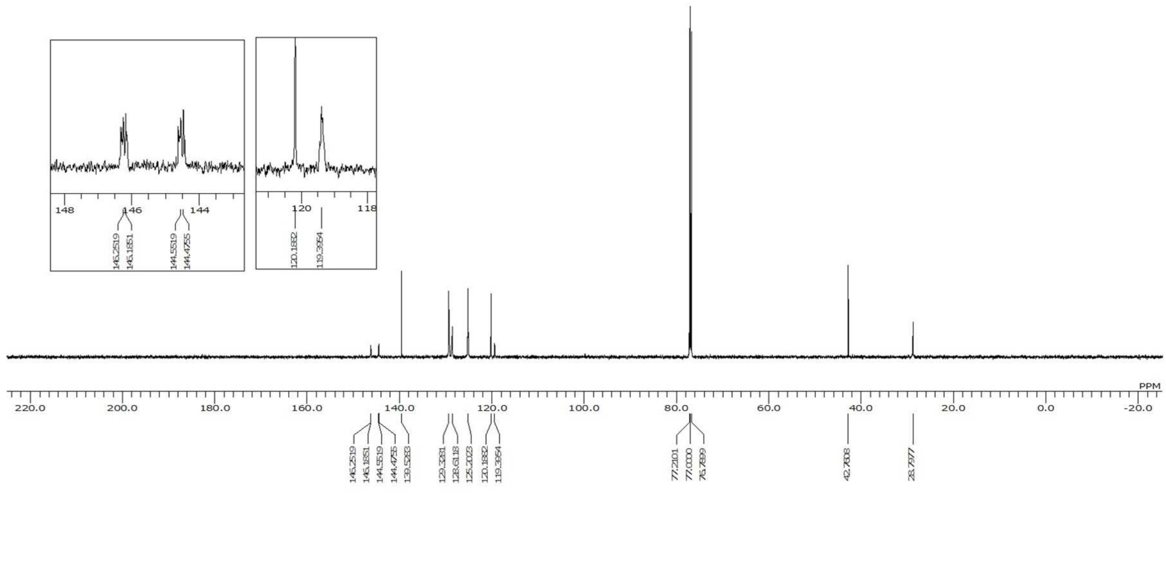

^{19}F NMR spectra of **3pa (Isomer A)** (CDCl$_3$, 376 MHz)

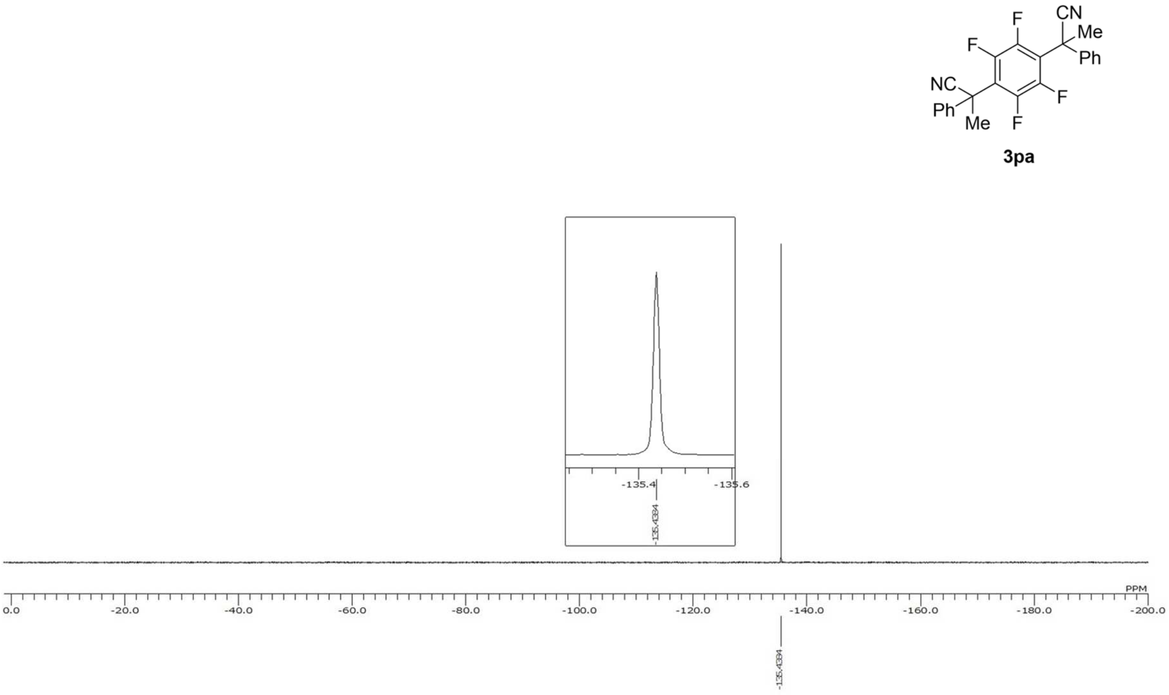

^{1}H NMR spectra of **3pa (Isomer B)** (CDCl$_3$, 600 MHz)

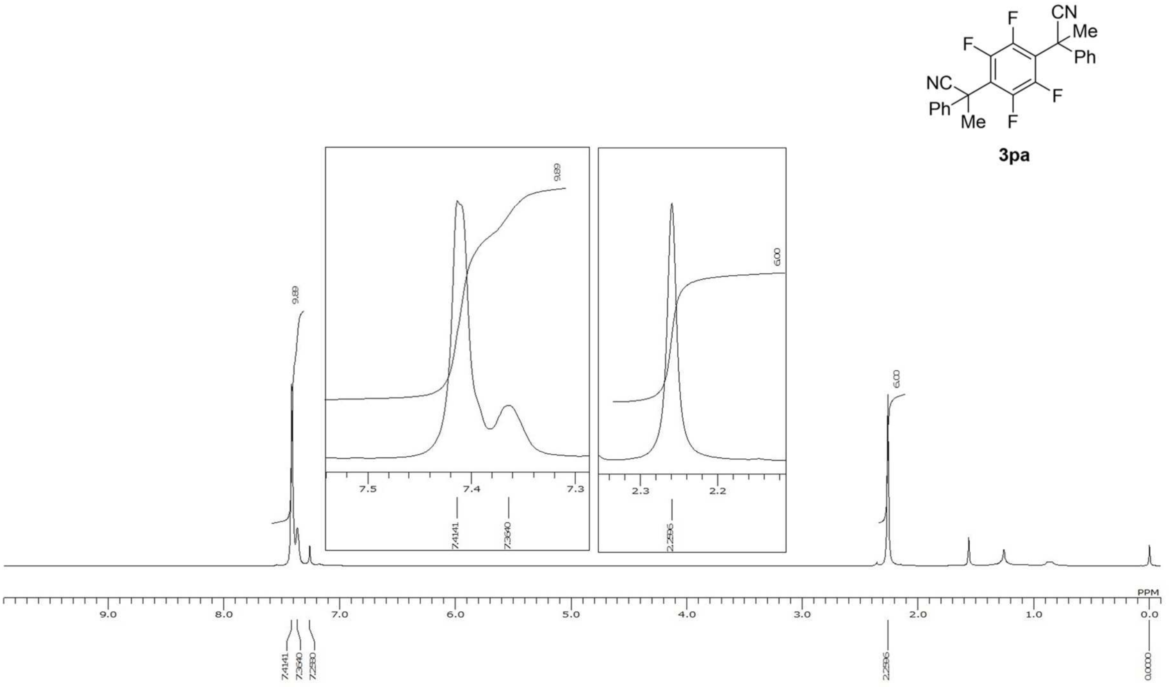

^{13}C NMR spectra of **3pa (Isomer B)** ($CDCl_3$, 150 MHz)

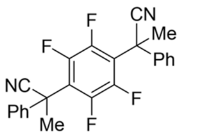

3pa

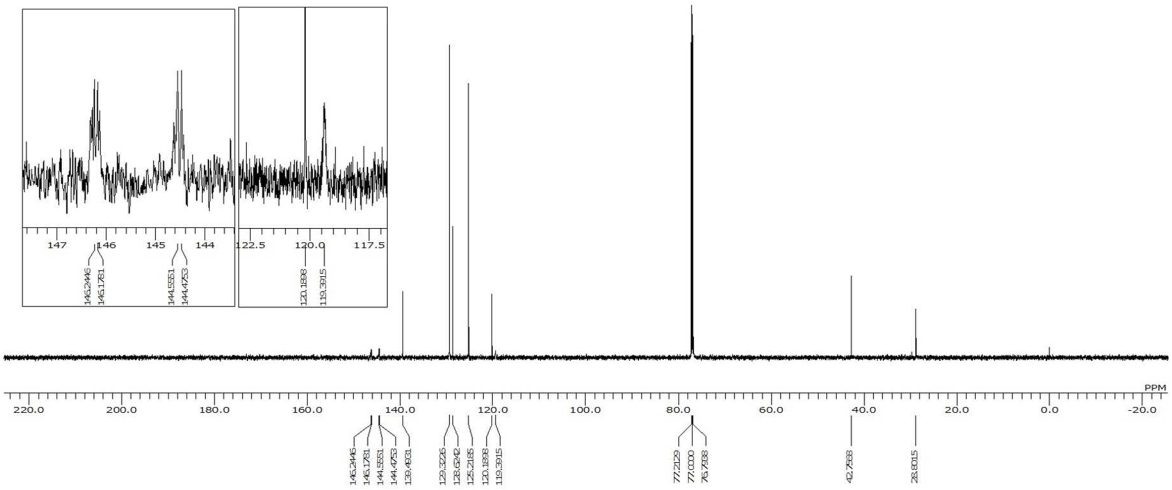

^{19}F NMR spectra of **3pa (Isomer B)** ($CDCl_3$, 376 MHz)

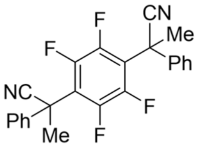

3pa

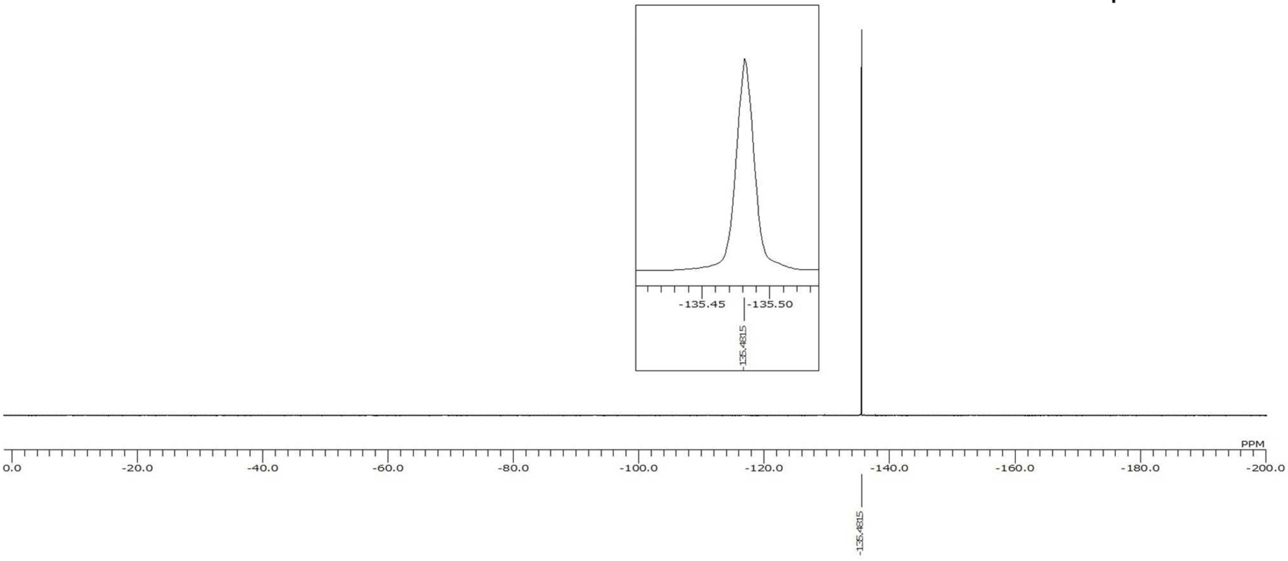

^{1}H NMR spectra of **3qa** ($CDCl_3$, 400 MHz)

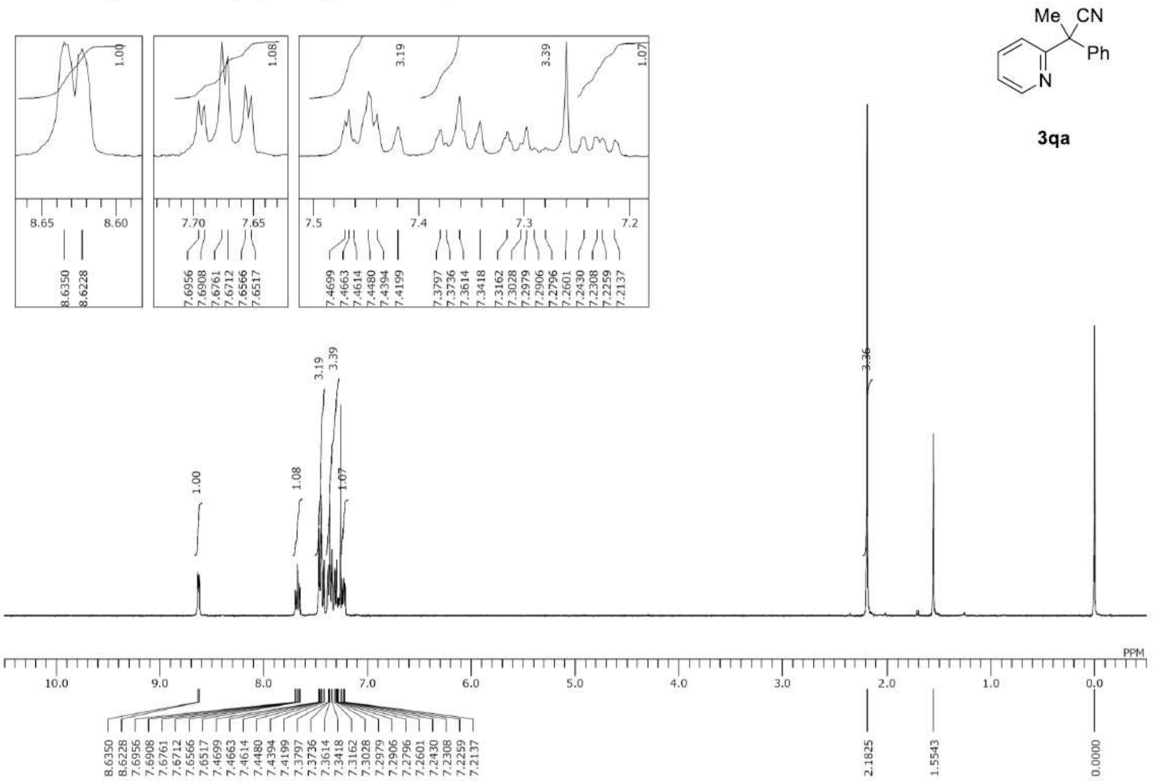

^{13}C NMR spectra of **3qa** ($CDCl_3$, 100 MHz)

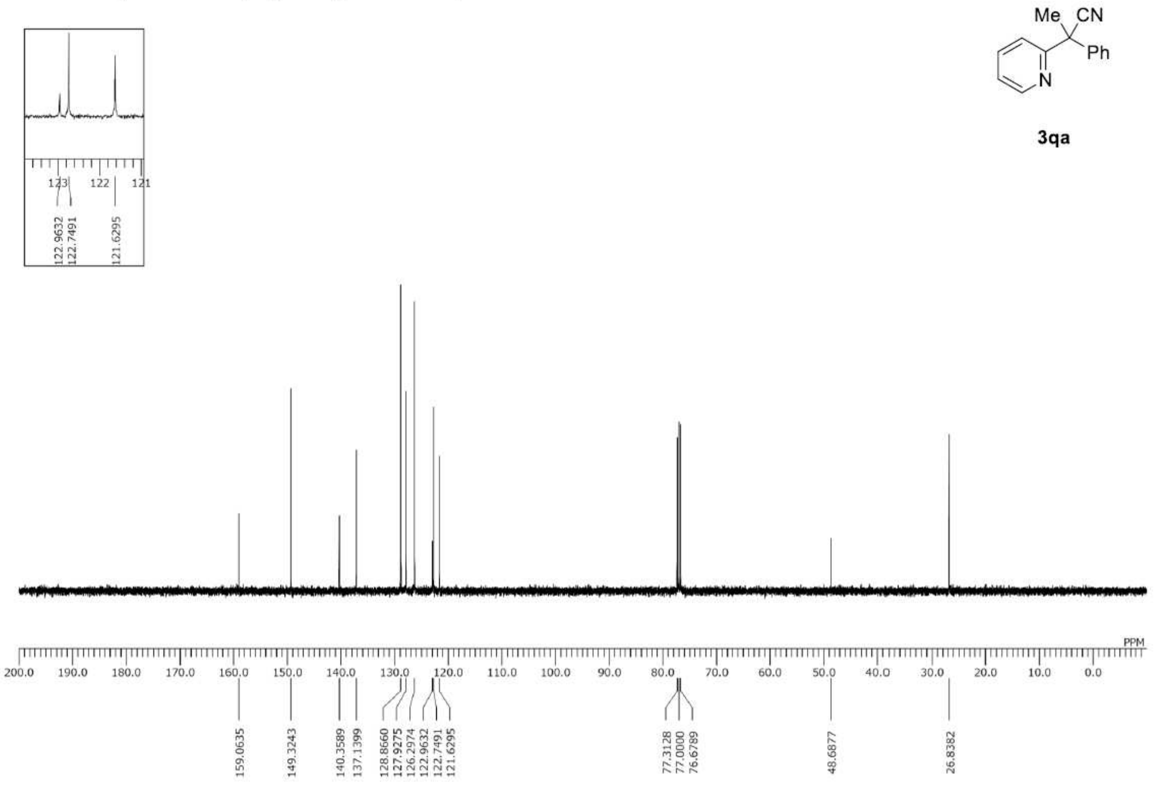

^{1}H NMR spectra of **3ra** ($CDCl_3$, 400 MHz)

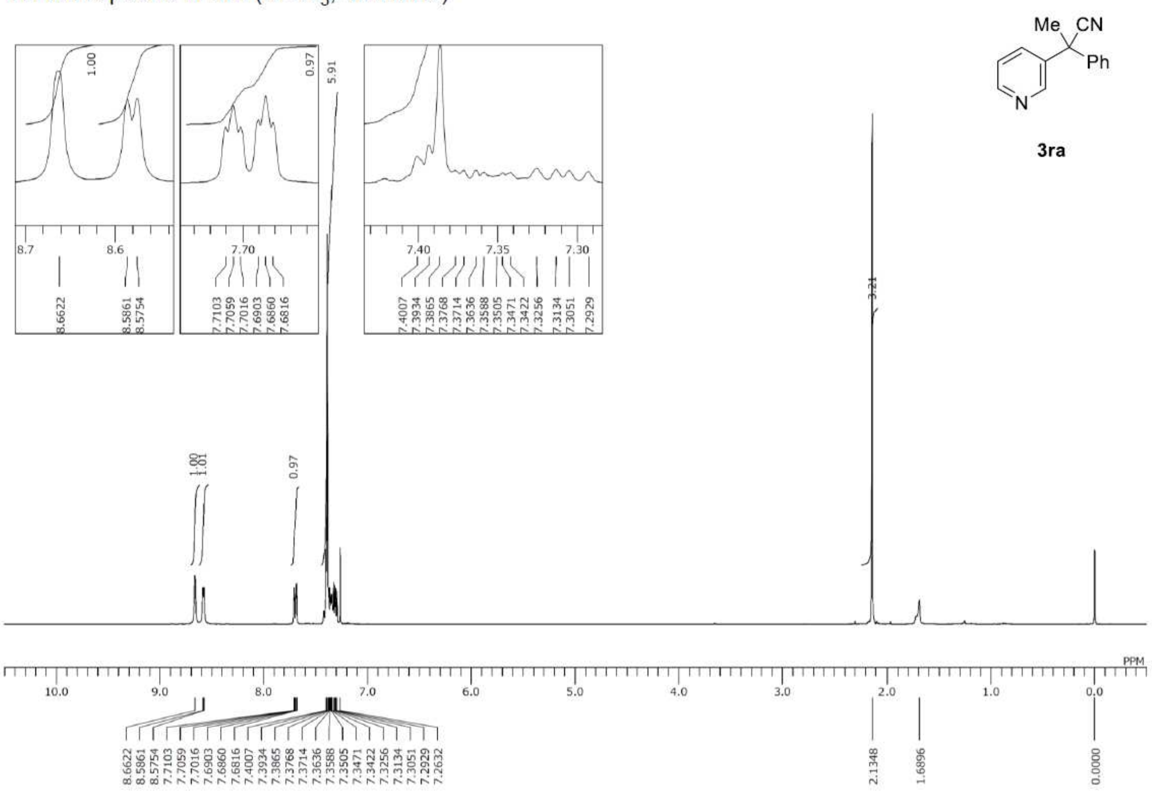

^{13}C NMR spectra of **3ra** ($CDCl_3$, 100 MHz)

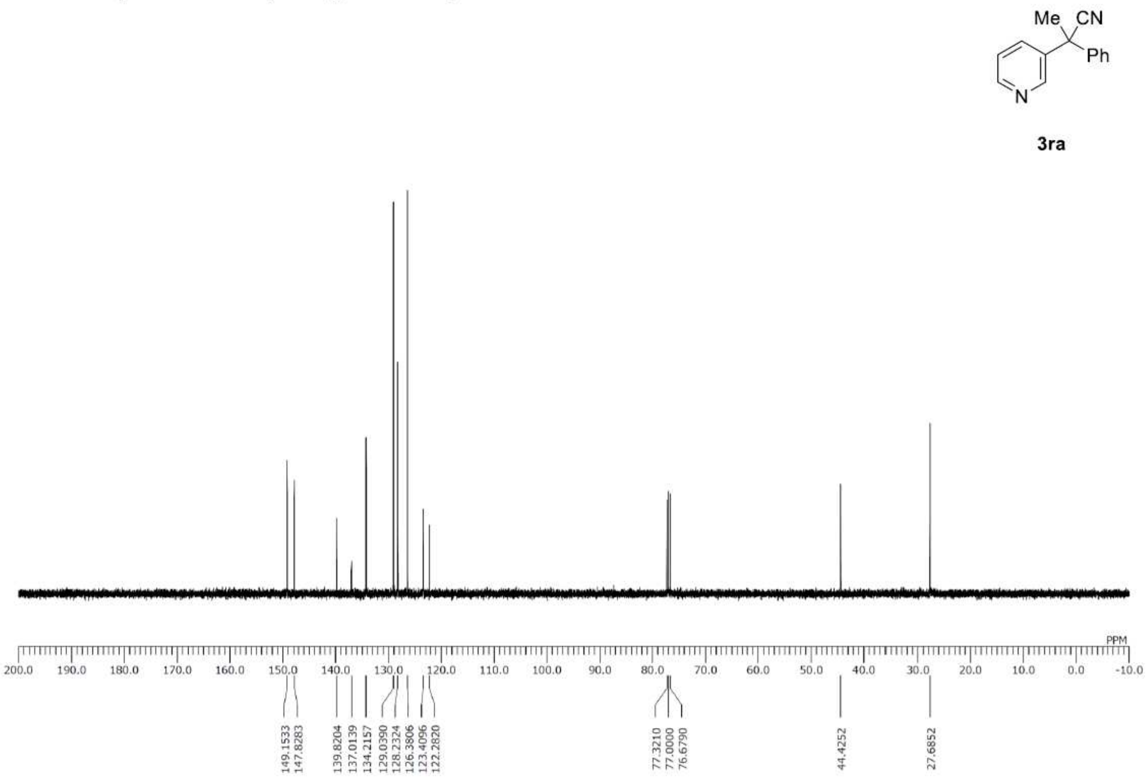

^{1}H NMR spectra of **3sa** (DMSO-d_6, 400 MHz)

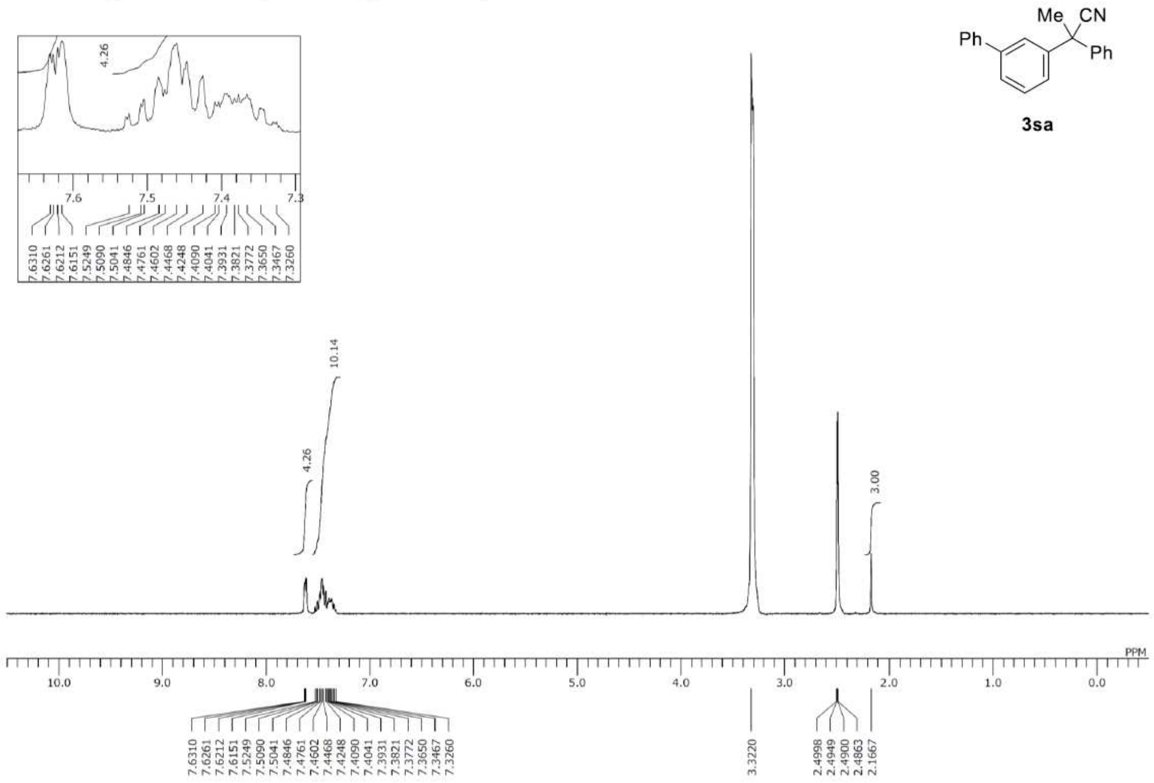

^{13}C NMR spectra of **3sa** (DMSO-d_6, 100 MHz)

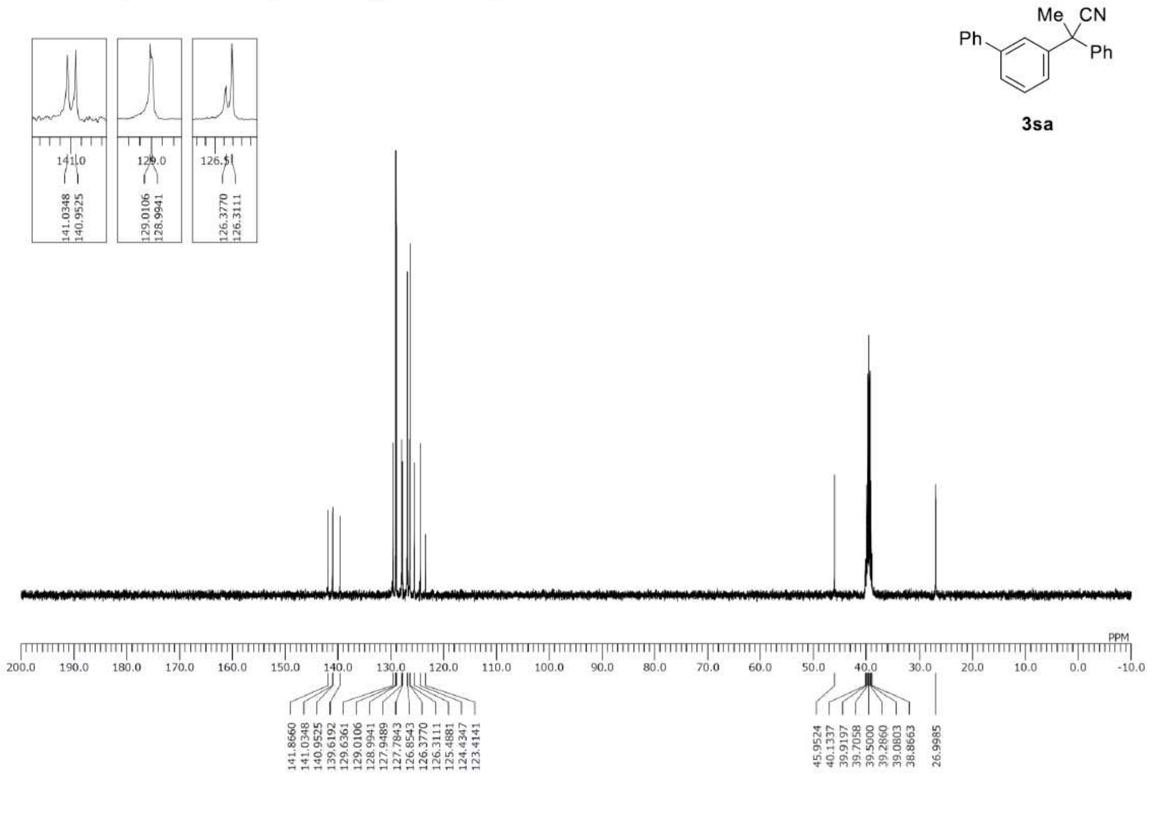

^{1}H NMR spectra of **3ta** (DMSO-d_6, 400 MHz)

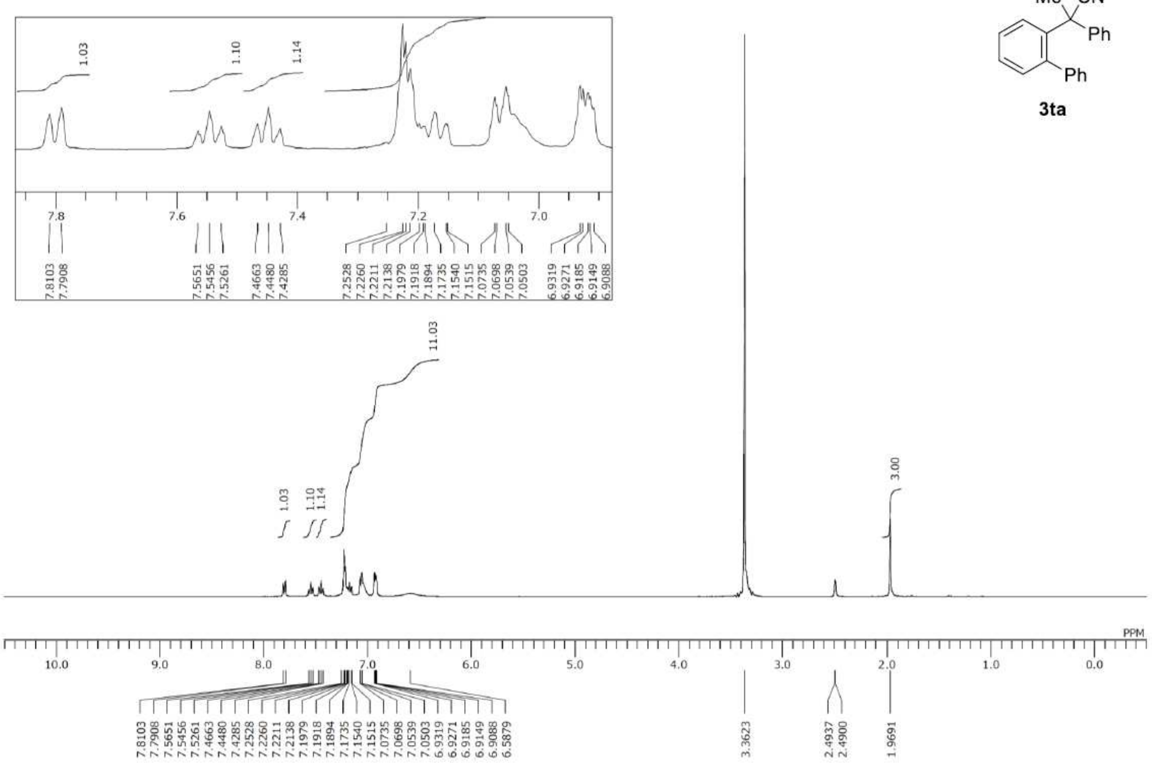

3ta

^{13}C NMR spectra of **3ta** (acetone-d_6, 100 MHz)

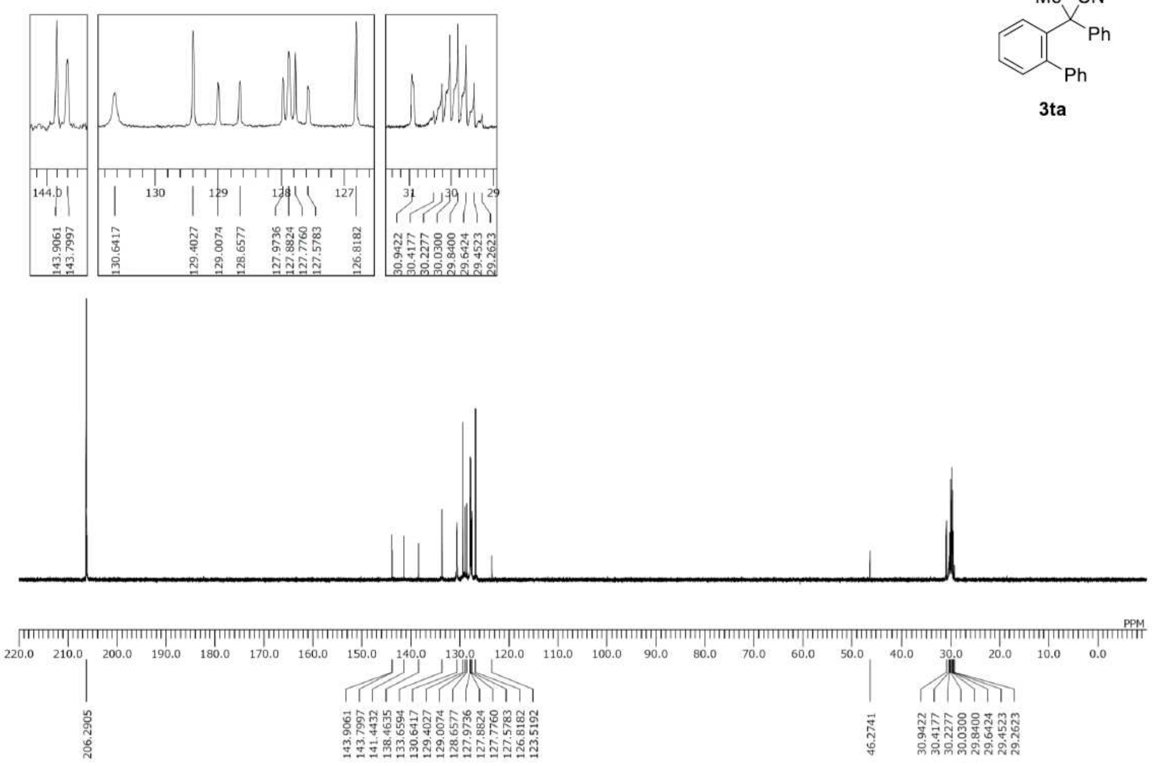

3ta

^{1}H NMR spectra of **3ua** ($CDCl_3$, 400 MHz)

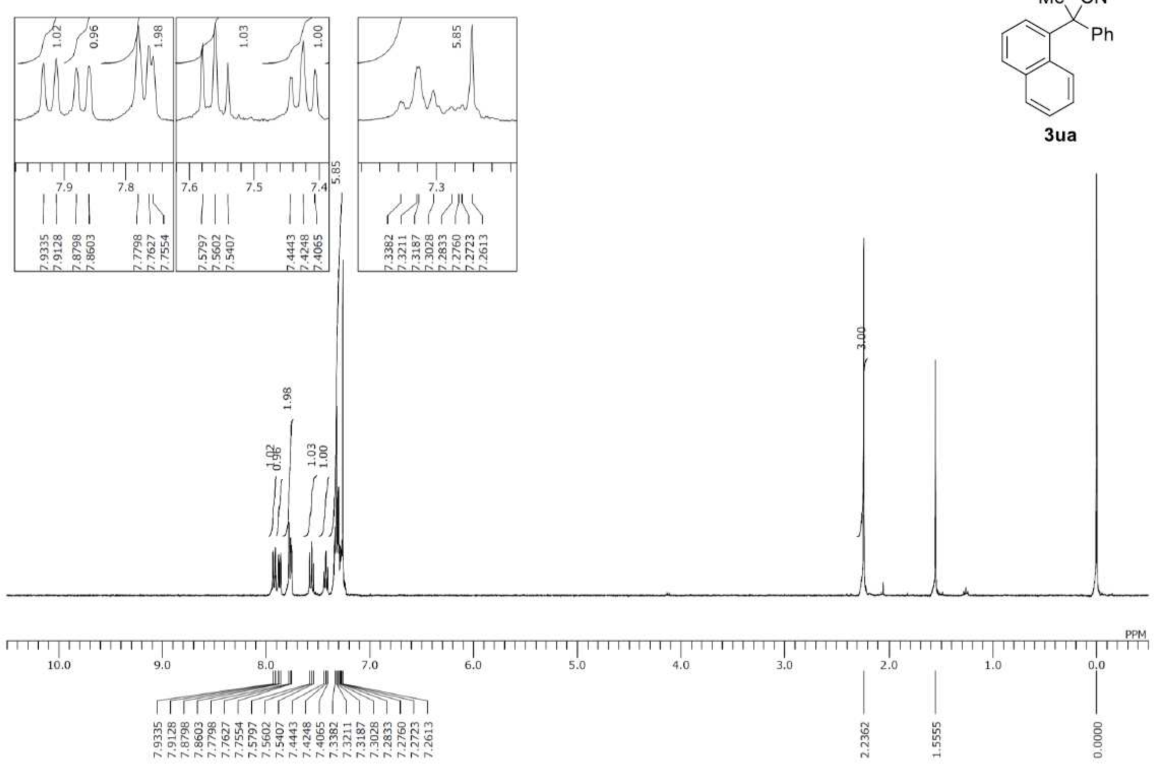

^{13}C NMR spectra of **3ua** (CD_3CN, 150 MHz)

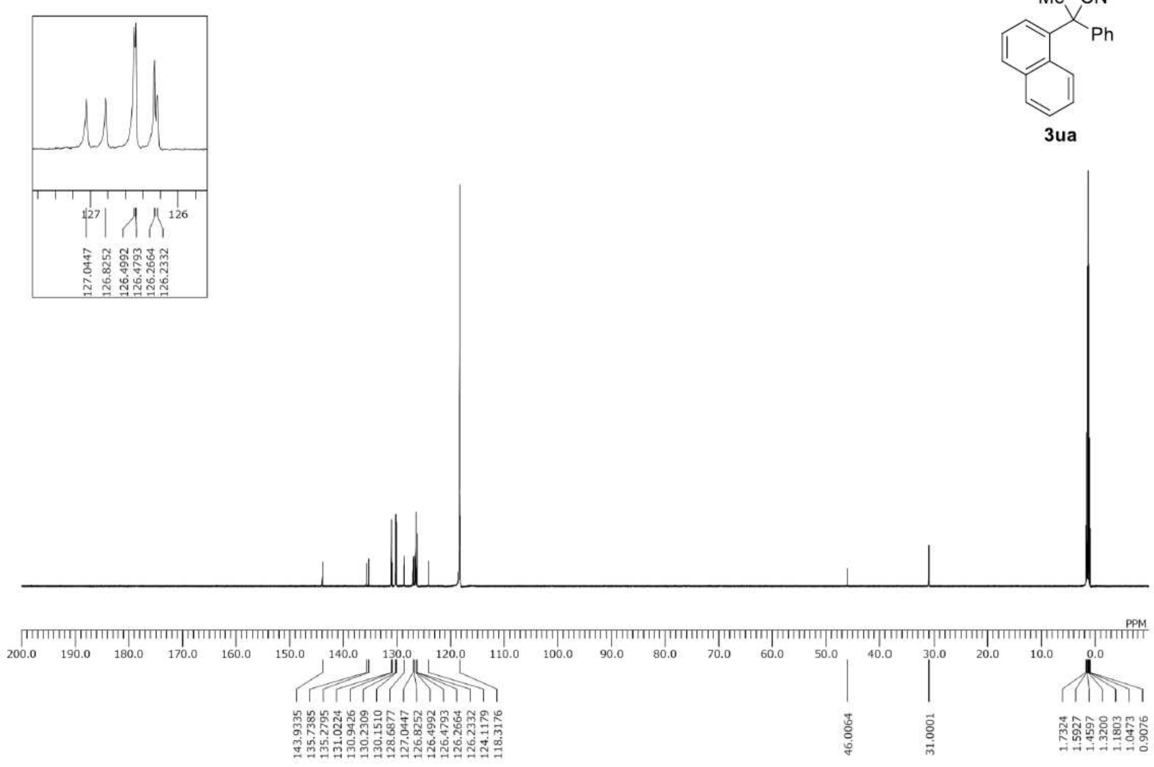

^{1}H NMR spectra of **3va** ($CDCl_3$, 400 MHz)

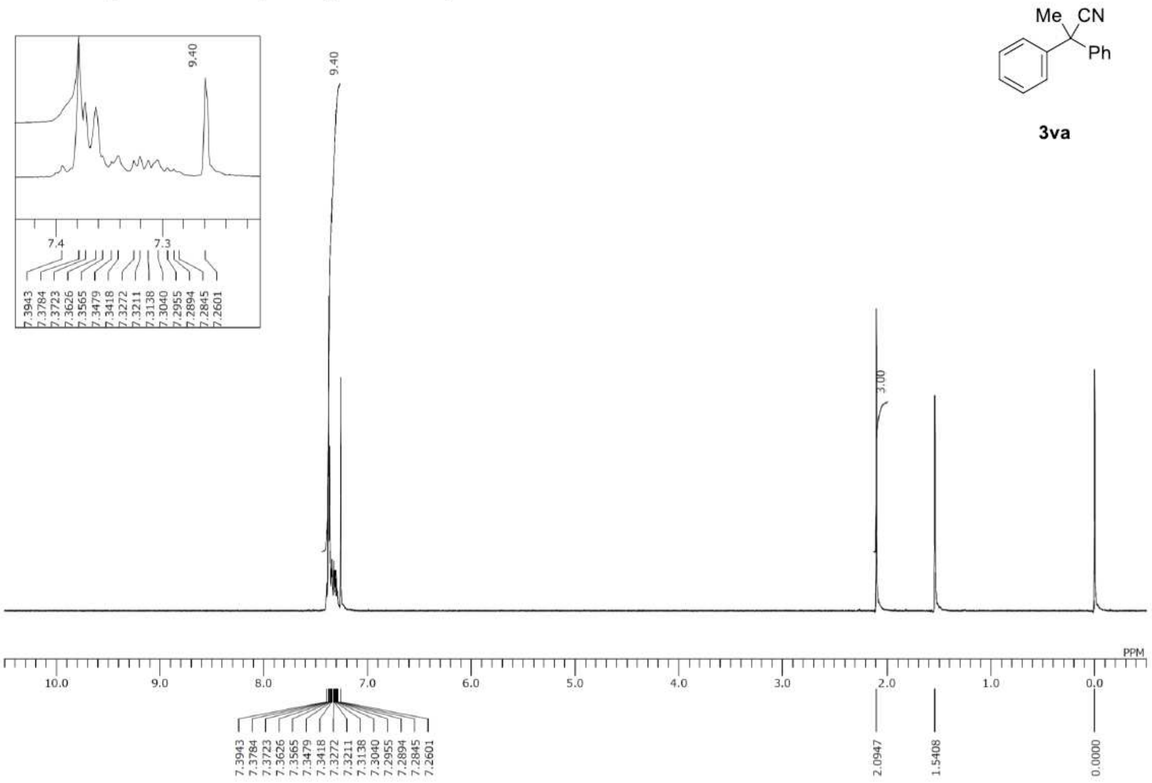

^{13}C NMR spectra of **3va** ($CDCl_3$, 100 MHz)

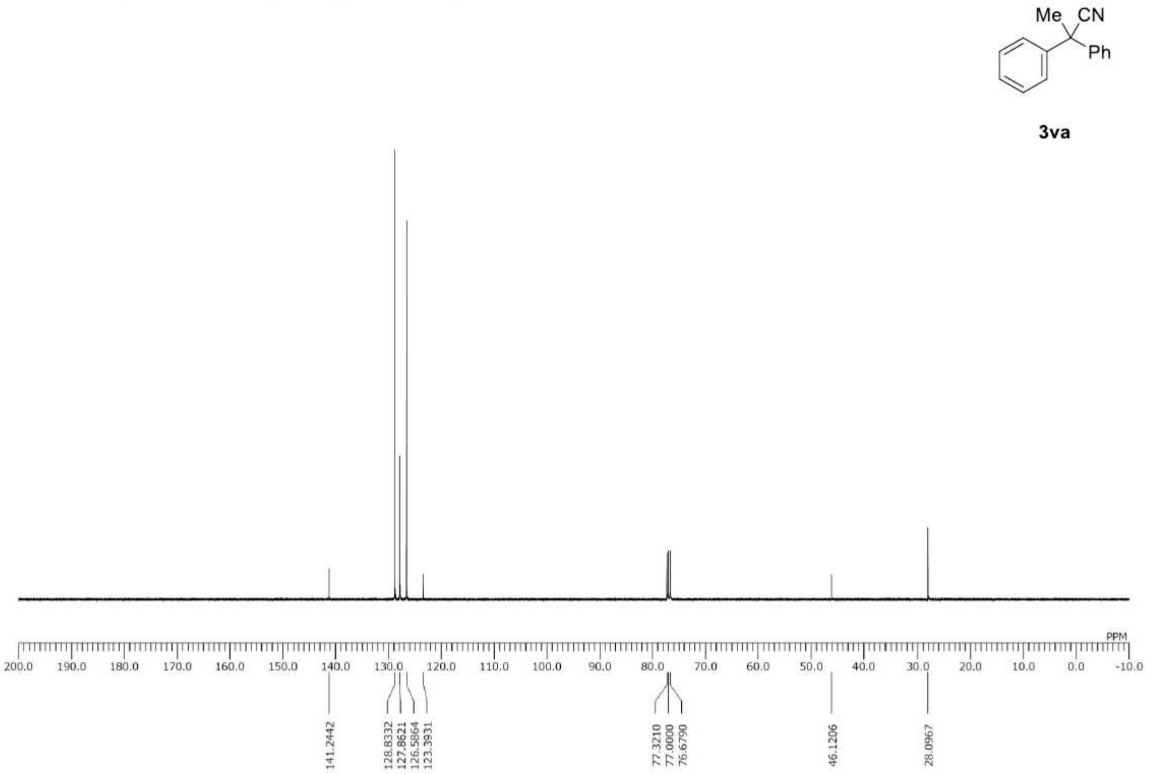

^{1}H NMR spectra of **3wa** ($CDCl_3$, 400 MHz)

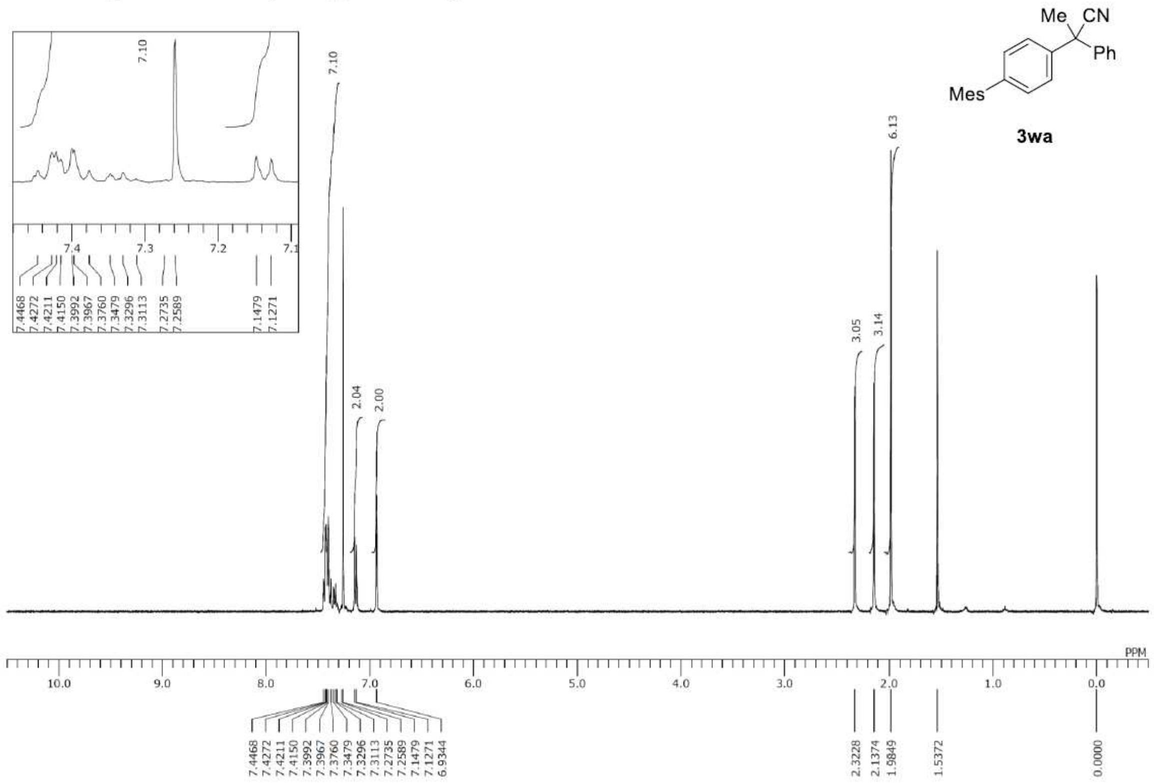

^{13}C NMR spectra of **3wa** ($CDCl_3$, 150 MHz, 59 °C)

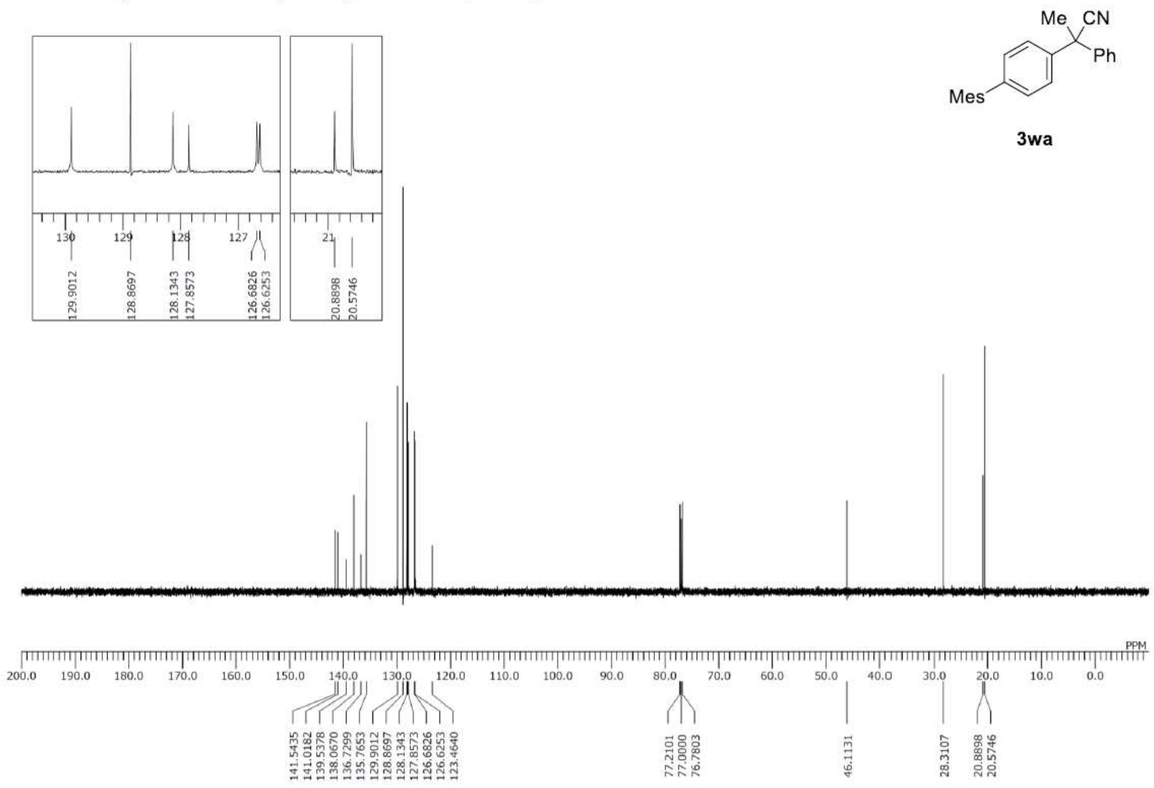

^{1}H NMR spectra of **3xa** ($CDCl_3$, 400 MHz)

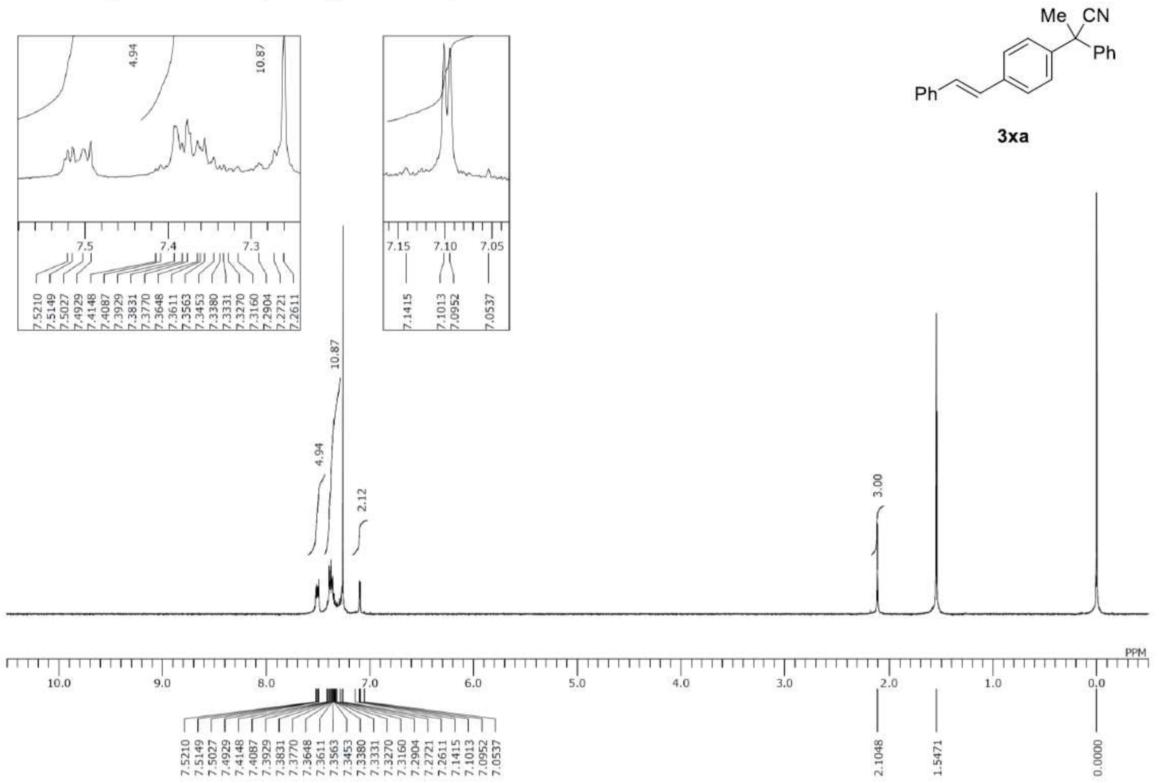

^{13}C NMR spectra of **3xa** (DMSO-d_6, 150 MHz)

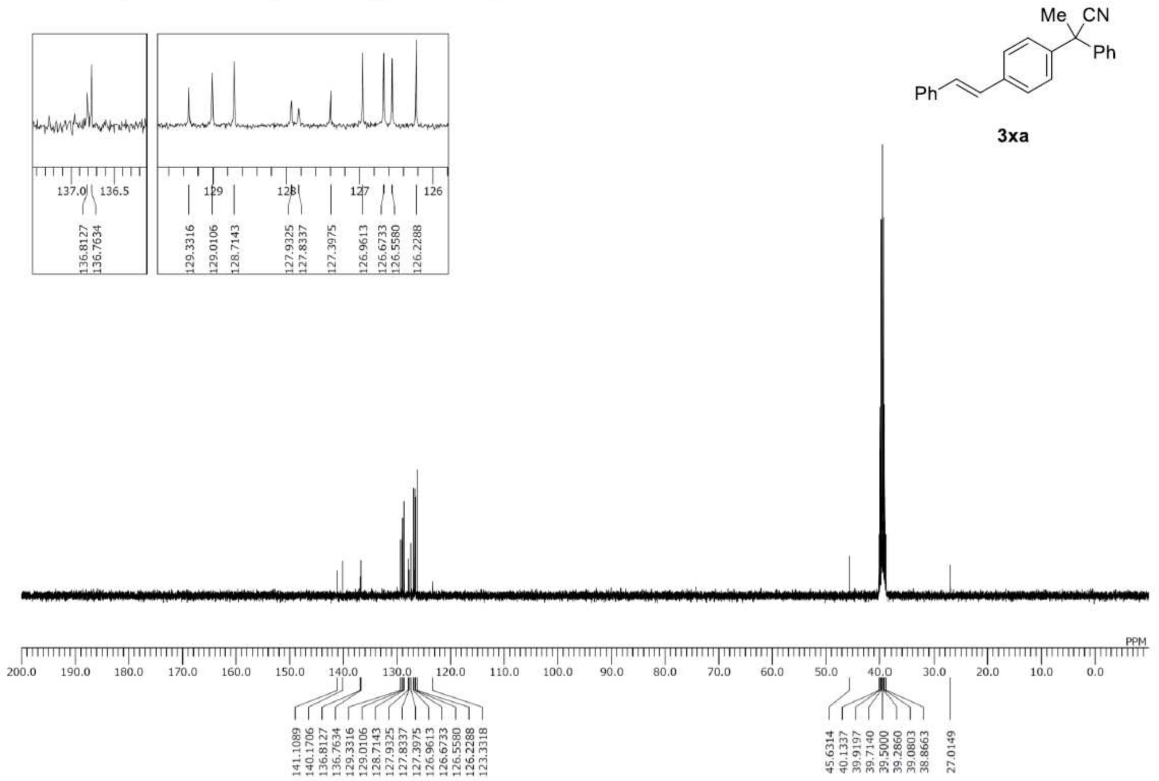

^{1}H NMR spectra of **3ya** ($CDCl_3$, 400 MHz)

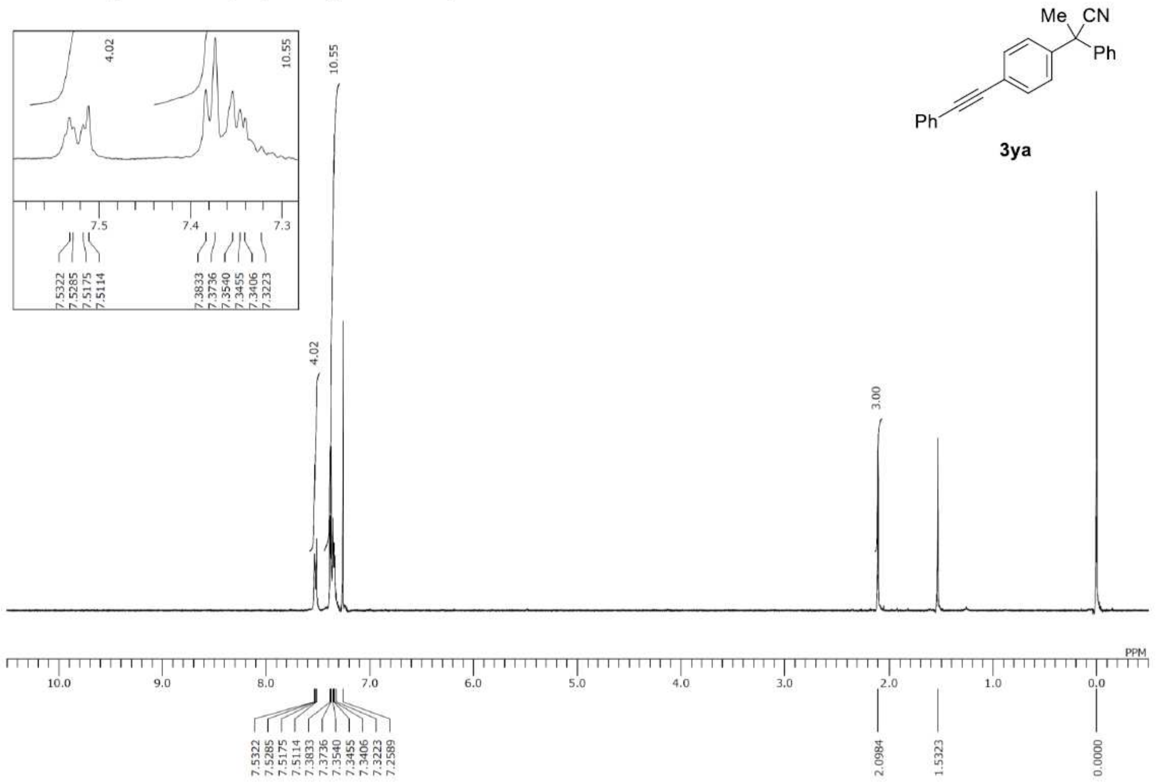

^{13}C NMR spectra of **3ya** ($CDCl_3$, 100 MHz)

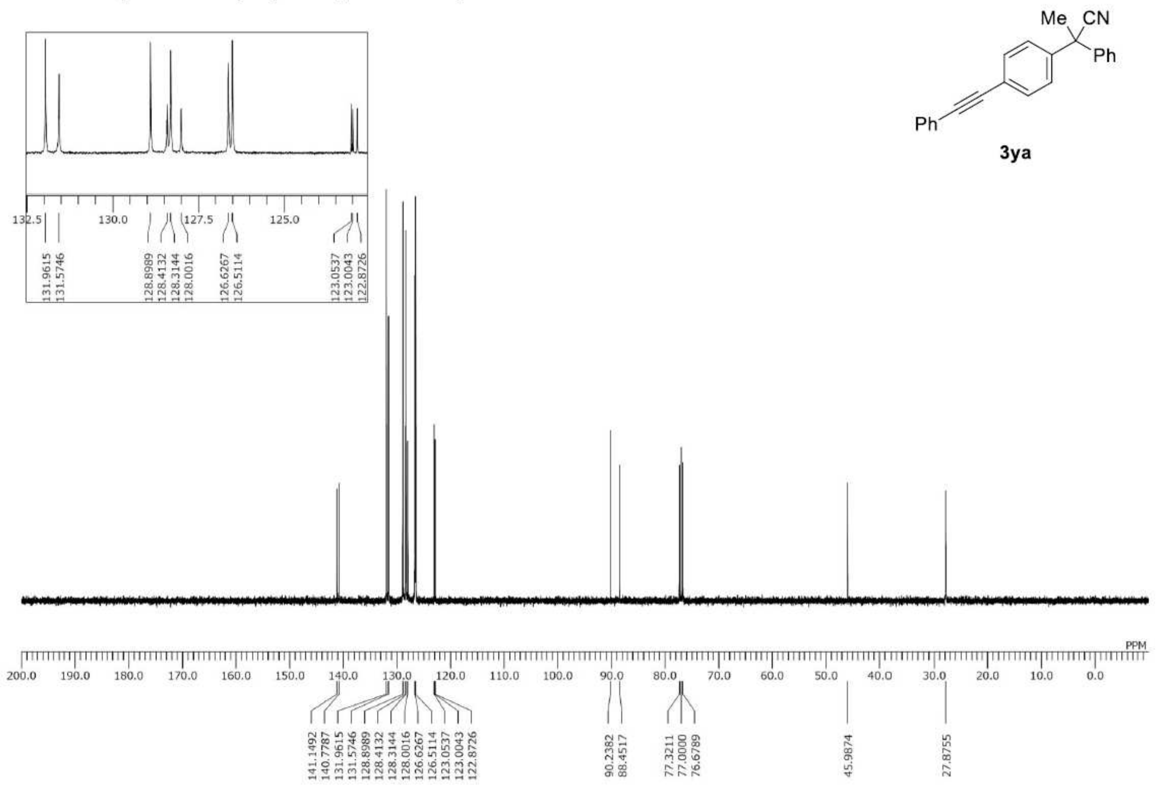

^{1}H NMR spectra of **3za** ($CDCl_3$, 400 MHz)

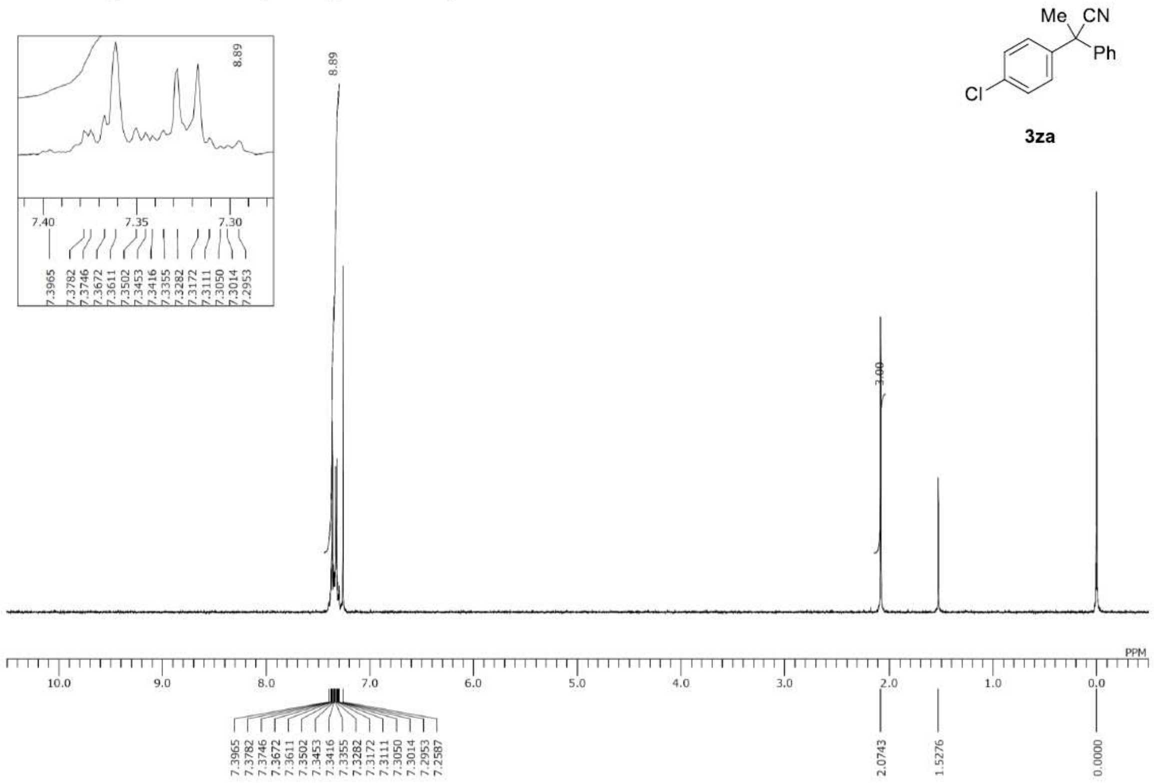

^{13}C NMR spectra of **3za** ($CDCl_3$, 100 MHz)

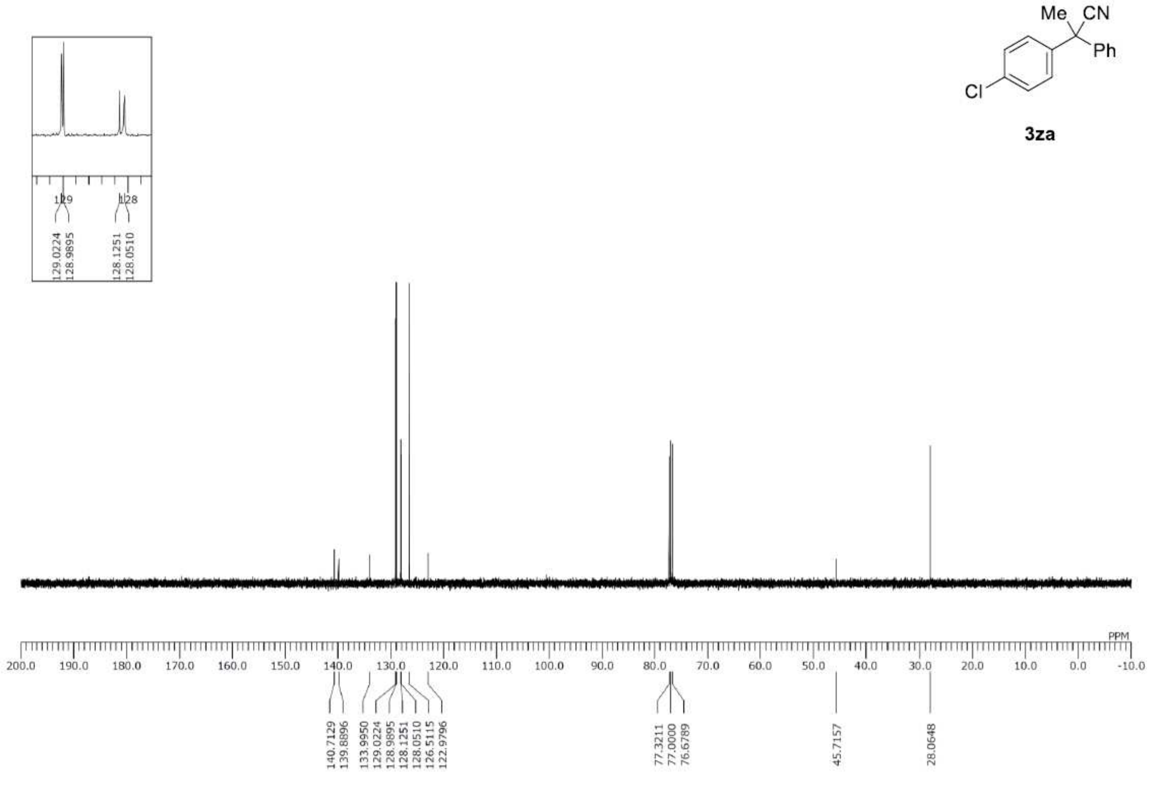

^1H NMR spectra of **3Aa** ($CDCl_3$, 400 MHz)

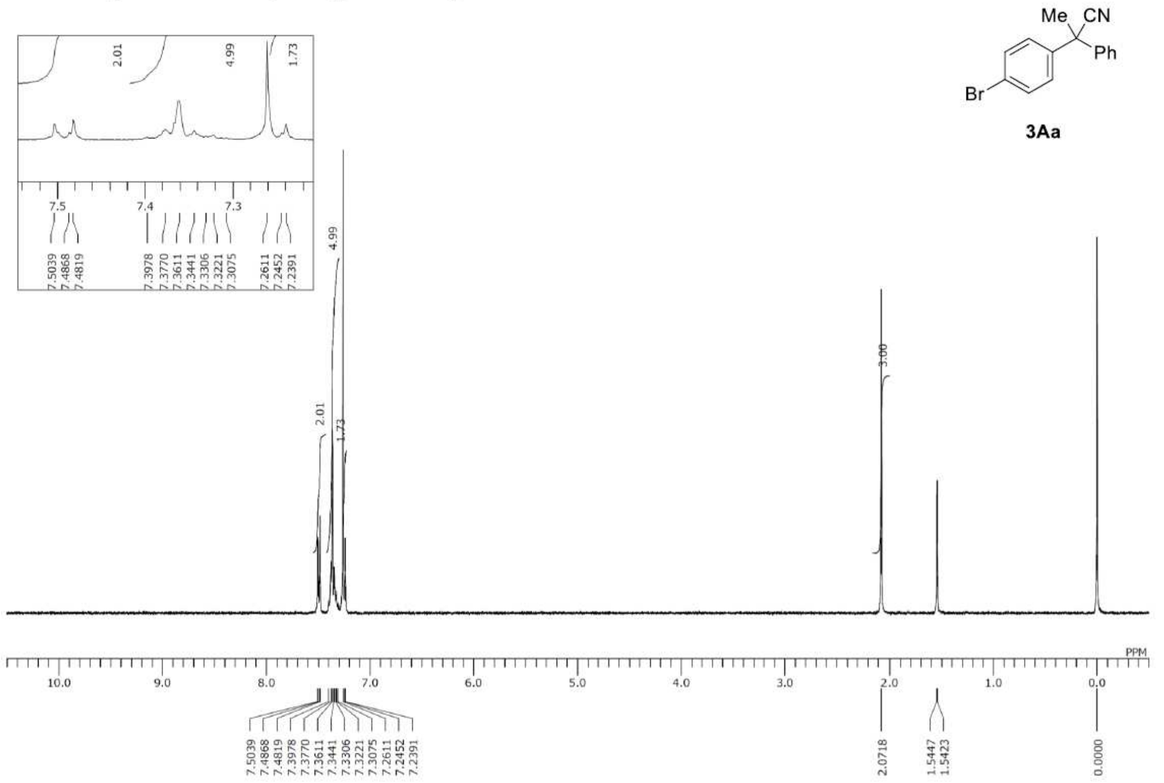

^{13}C NMR spectra of **3Aa** ($CDCl_3$, 100 MHz)

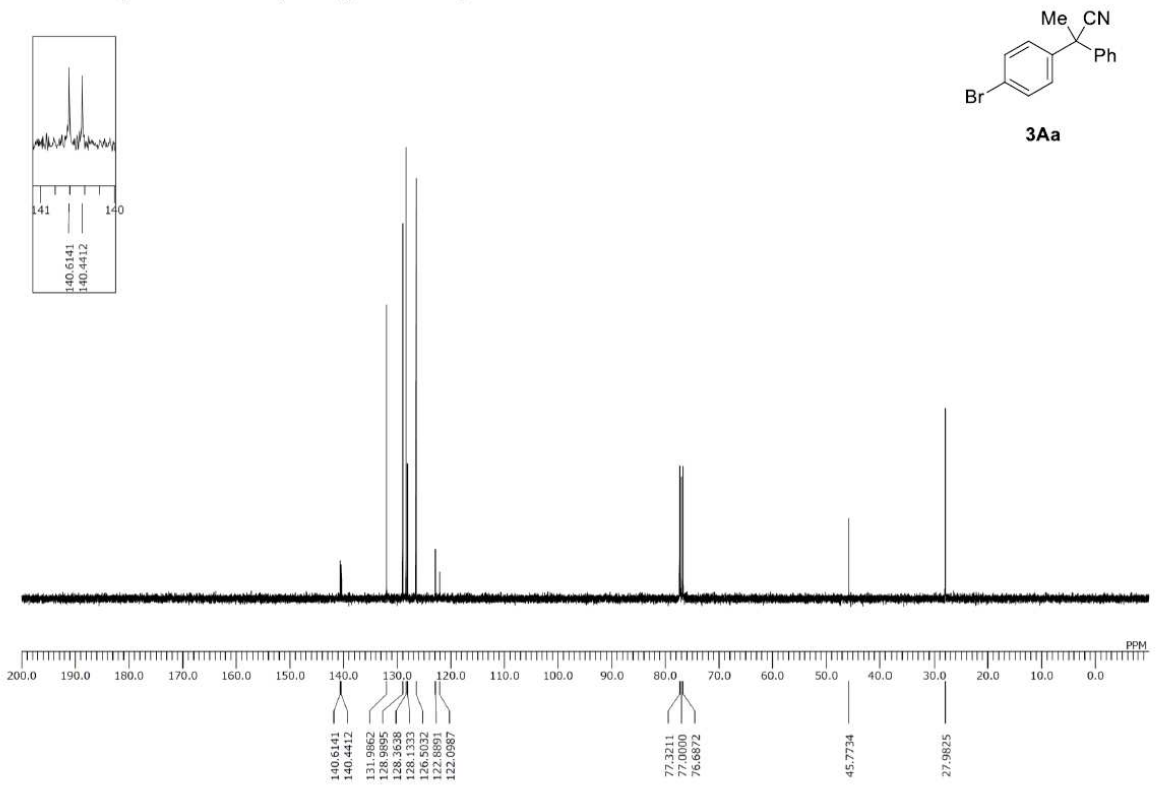

^{1}H NMR spectra of **3Ba** ($CDCl_3$, 400 MHz)

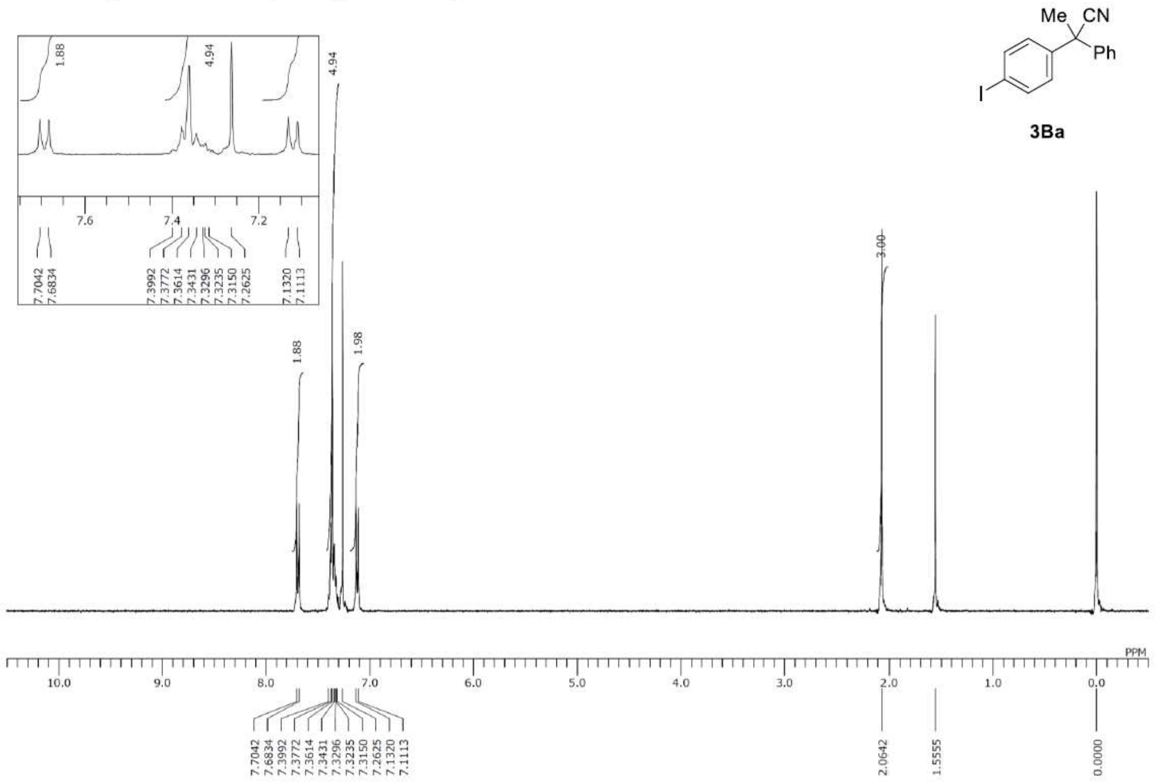

^{13}C NMR spectra of **3Ba** ($CDCl_3$, 100 MHz)

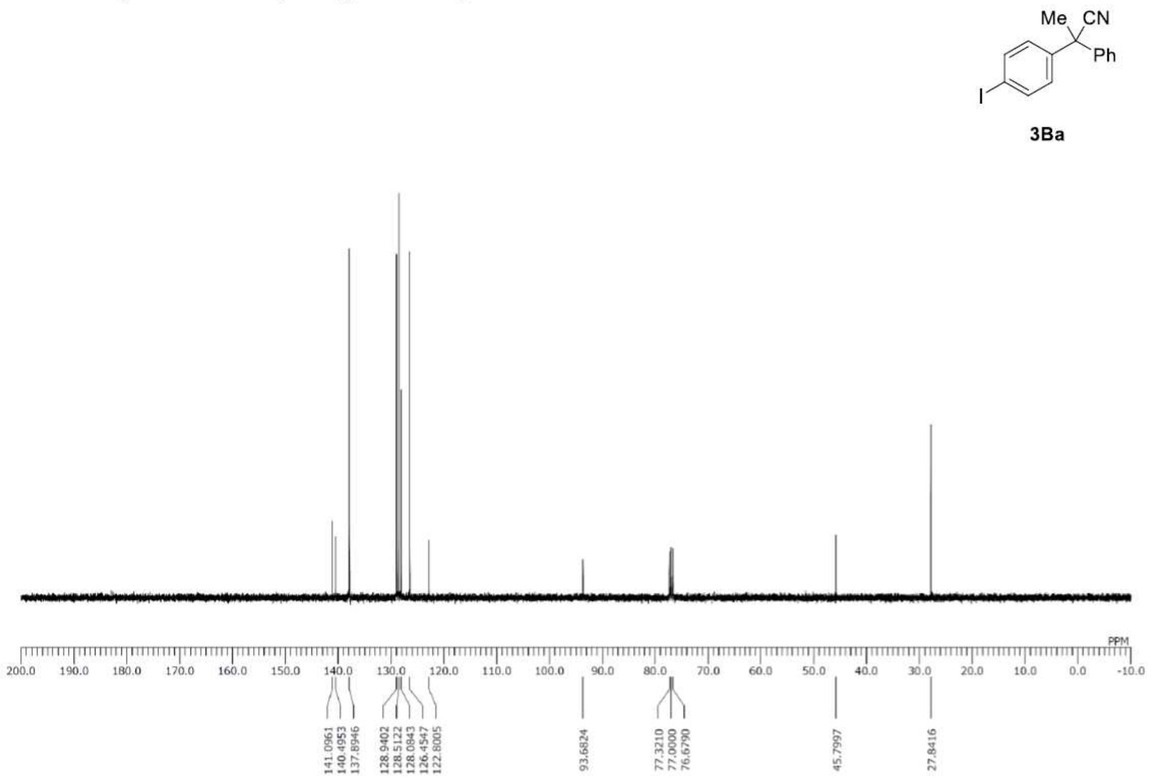

^{1}H NMR spectra of **3Ca** ($CDCl_3$, 400 MHz)

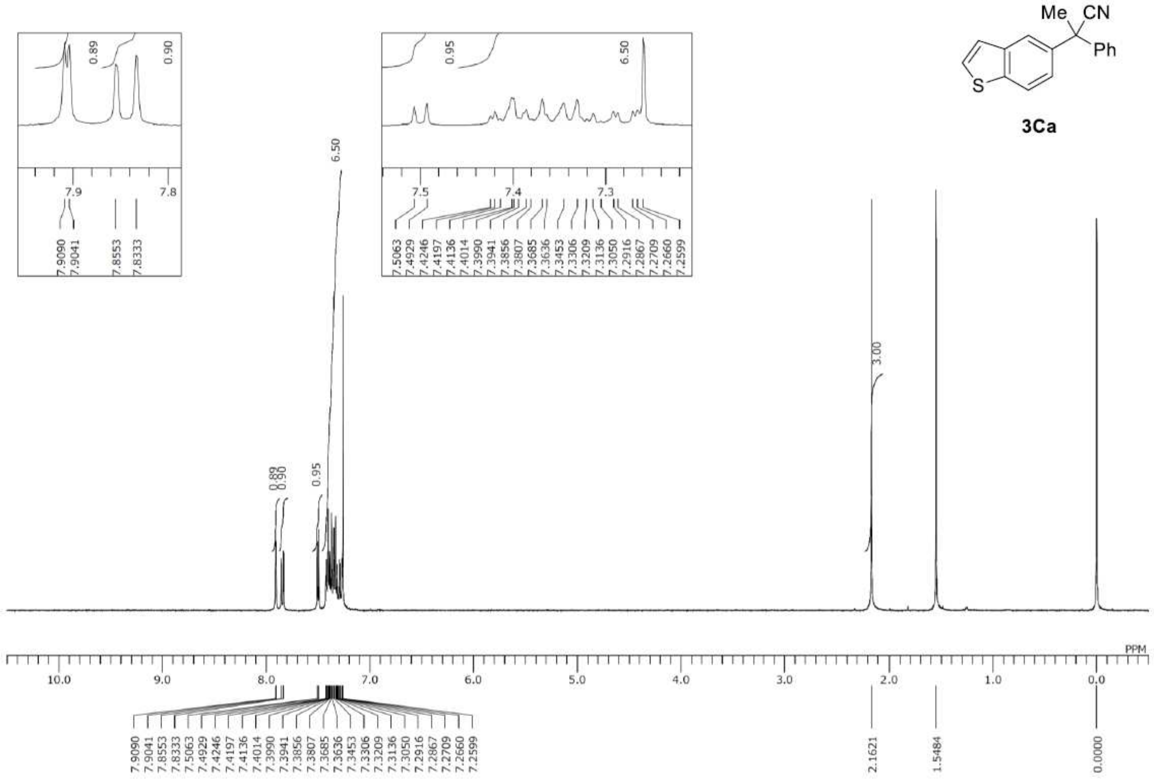

^{13}C NMR spectra of **3Ca** ($CDCl_3$, 100 MHz)

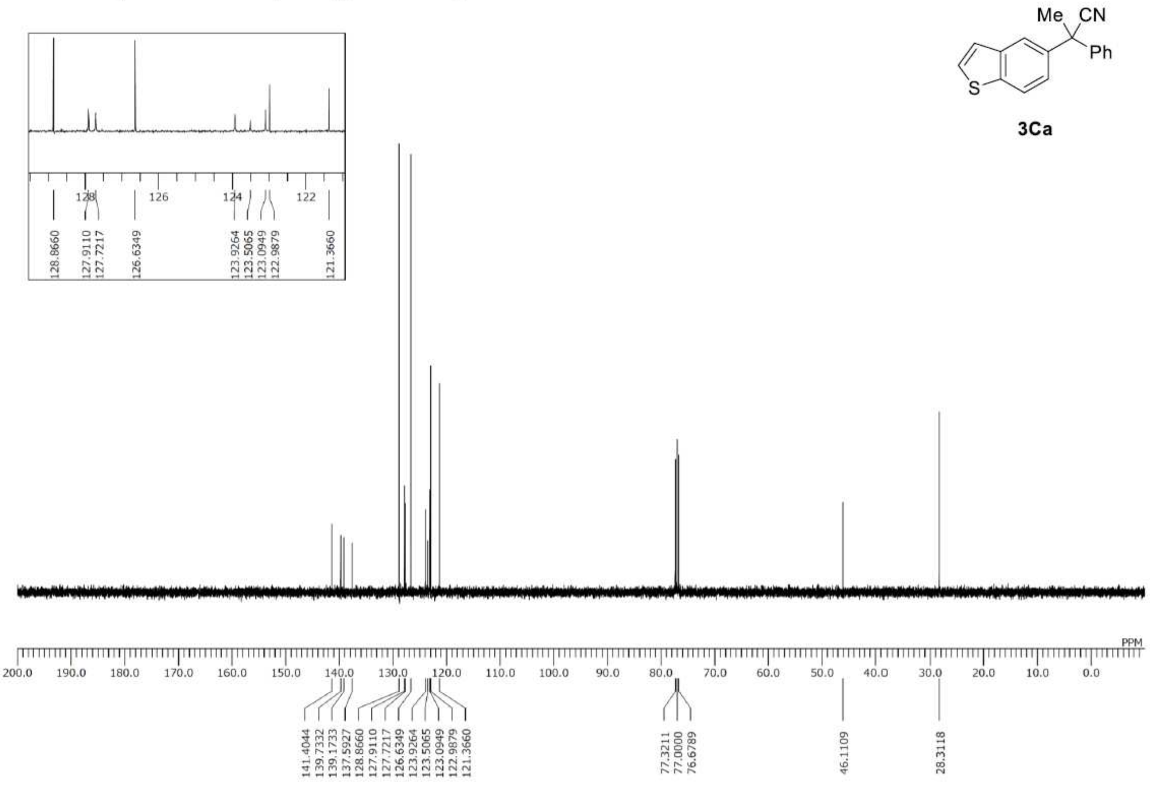

^{1}H NMR spectra of **3Da** ($CDCl_3$, 600 MHz)

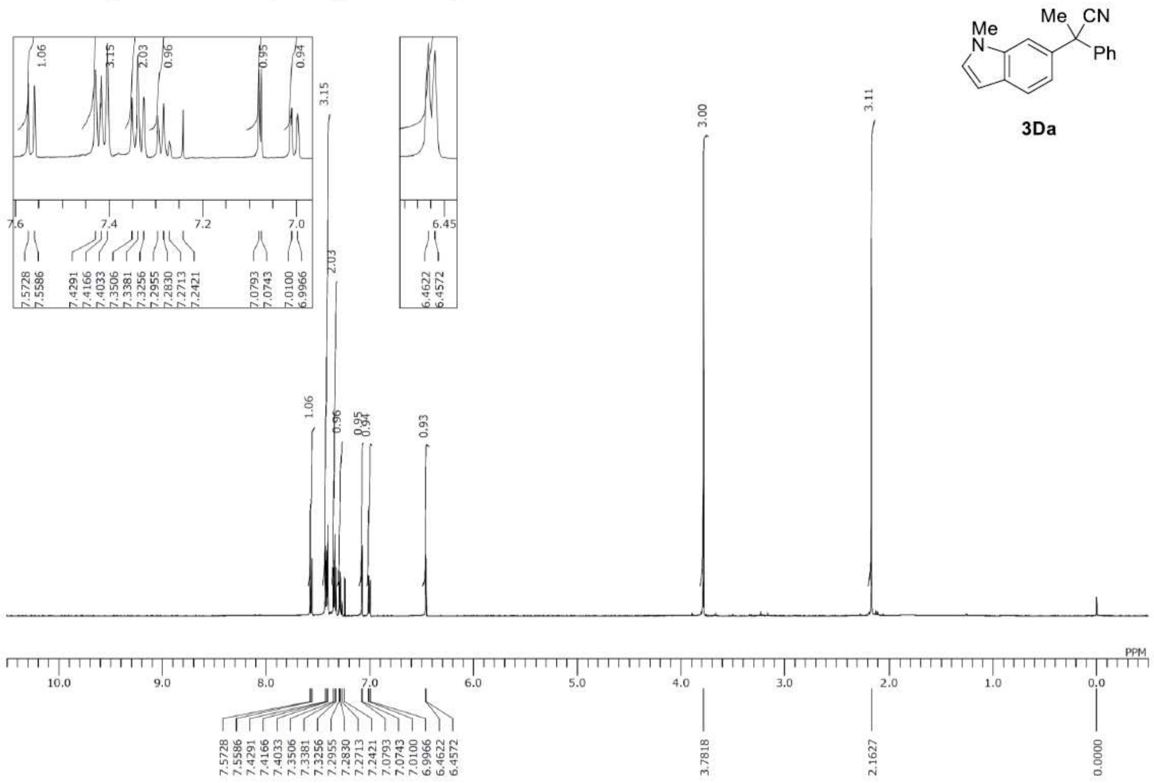

^{13}C NMR spectra of **3Da** ($CDCl_3$, 150 MHz)

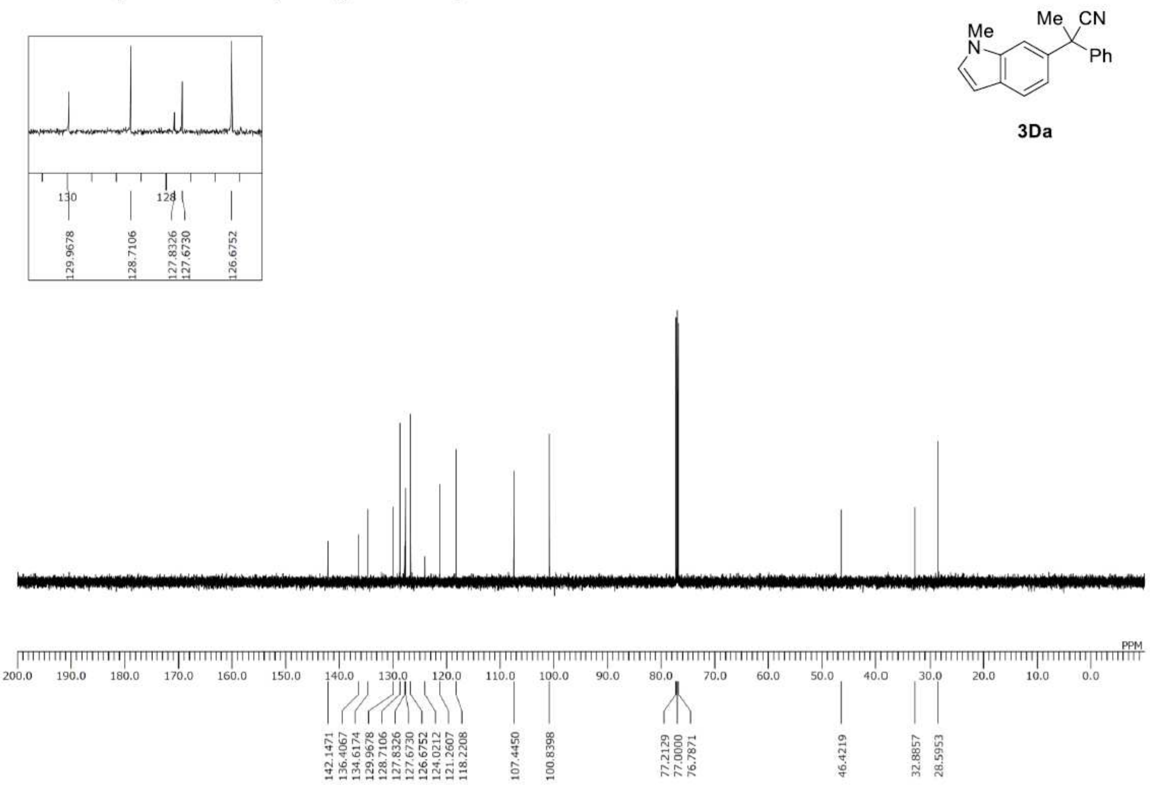

^{1}H NMR spectra of **3Ea** ($CDCl_3$, 400 MHz)

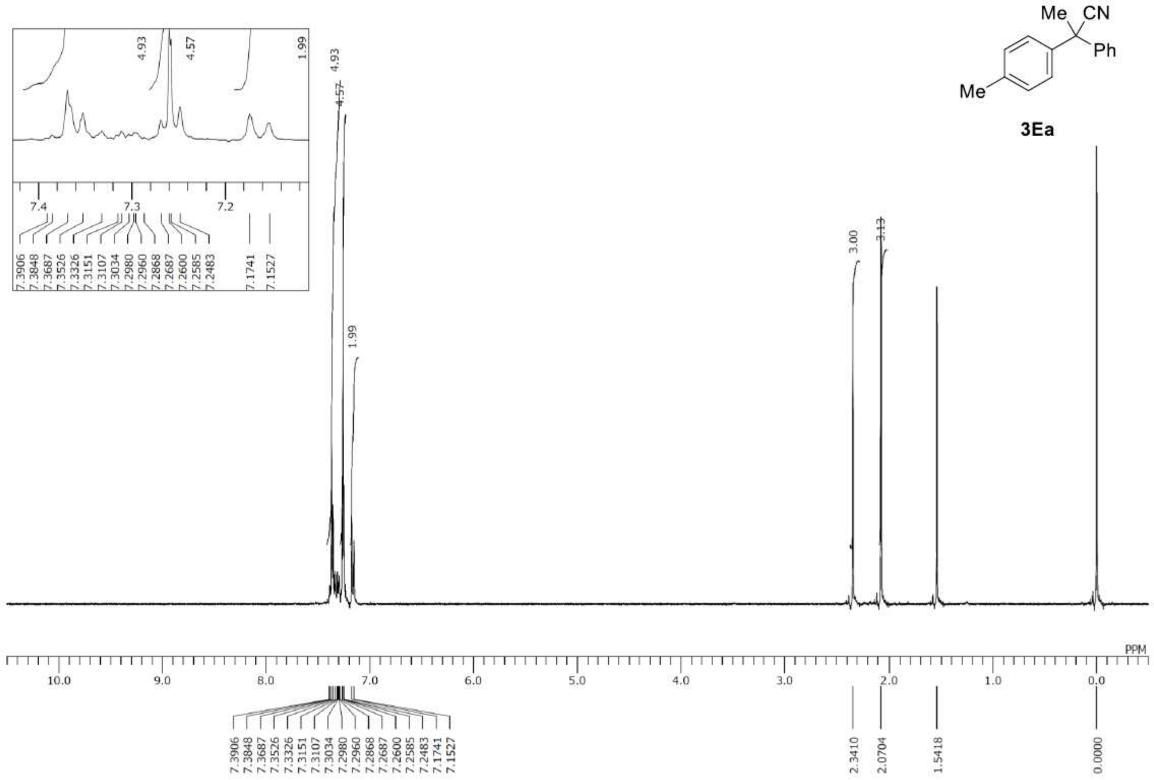

^{13}C NMR spectra of **3Ea** ($CDCl_3$, 100 MHz)

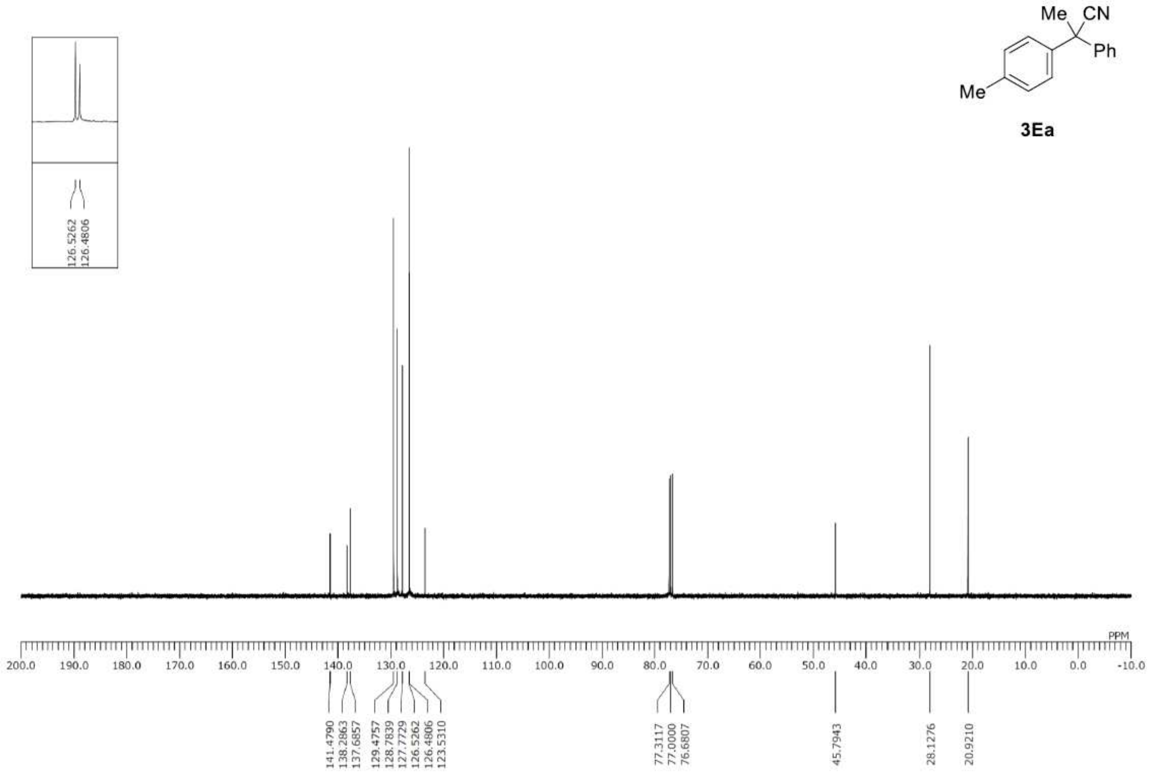

^{1}H NMR spectra of **3Fa** (DMSO-d_6, 400 MHz)

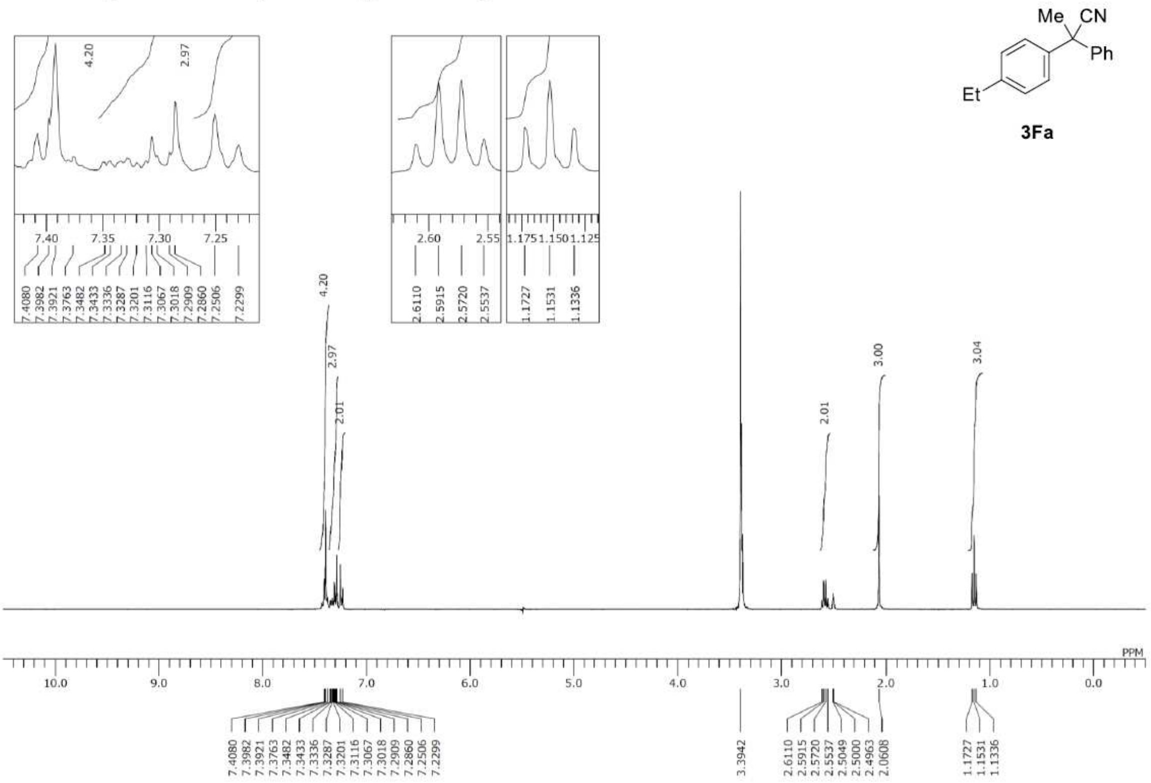

^{13}C NMR spectra of **3Fa** (DMSO-d_6, 100 MHz)

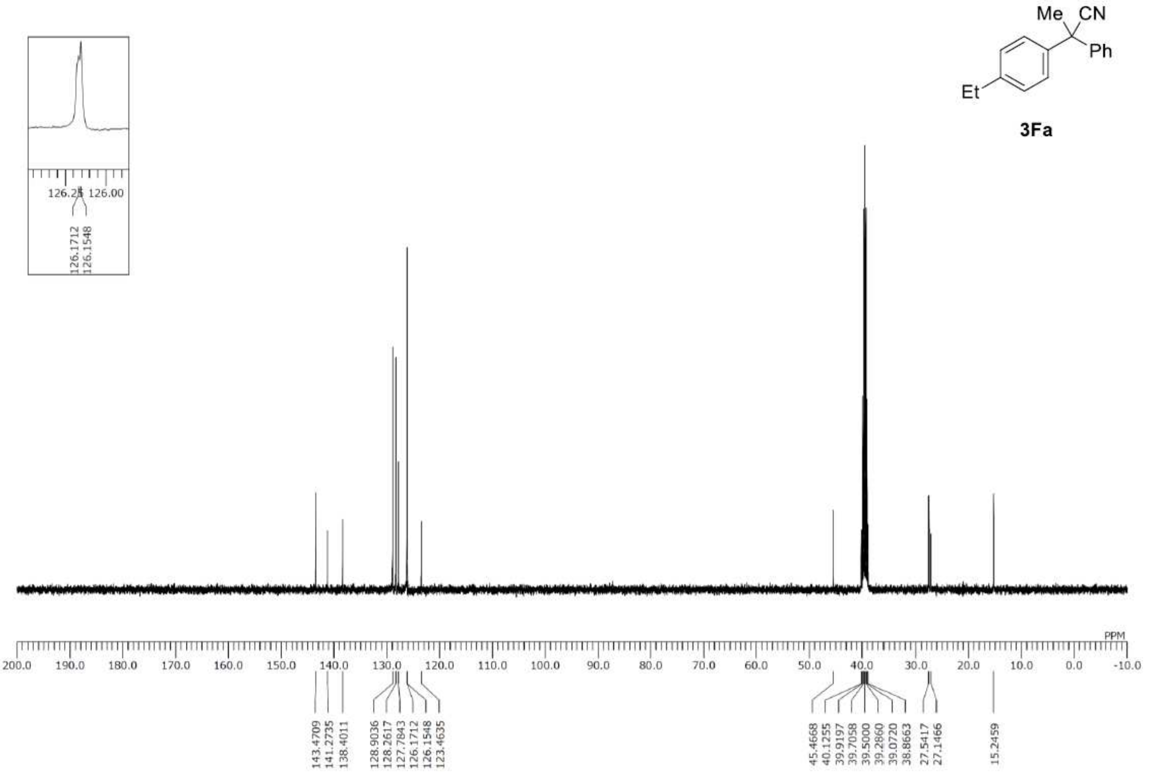

^{1}H NMR spectra of **3Ga** ($CDCl_3$, 400 MHz)

3Ga

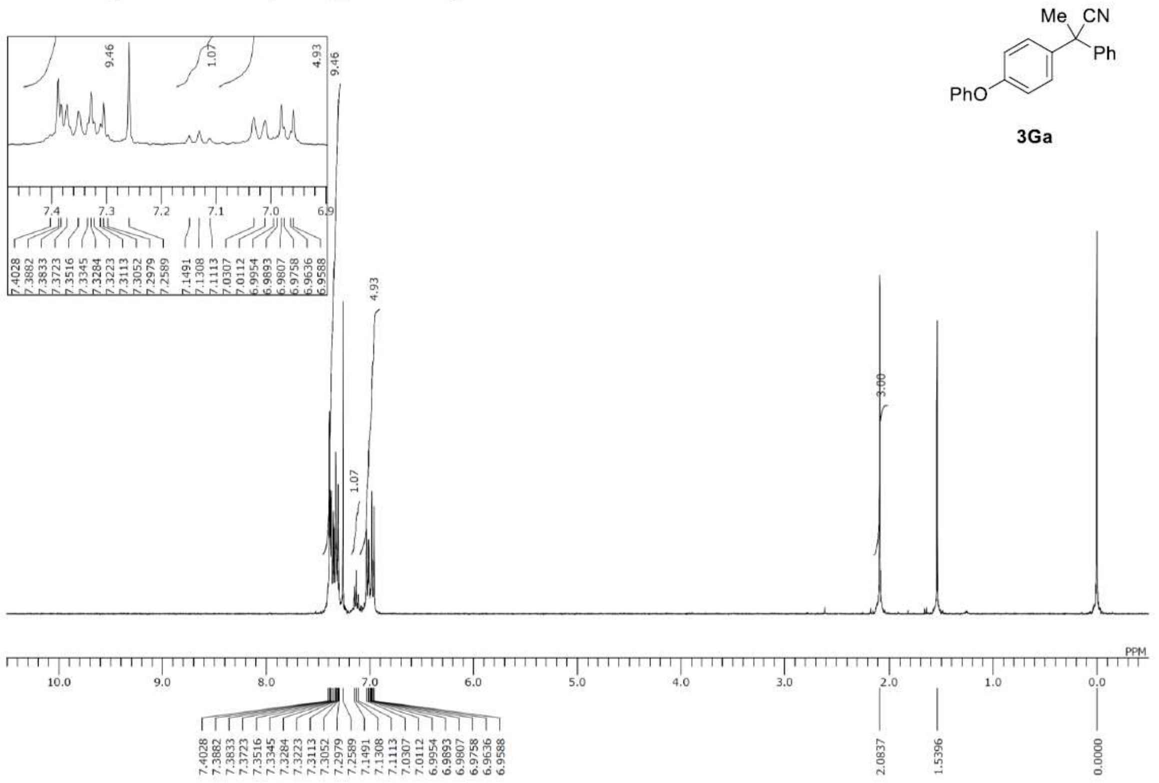

^{13}C NMR spectra of **3Ga** ($CDCl_3$, 100 MHz)

3Ga

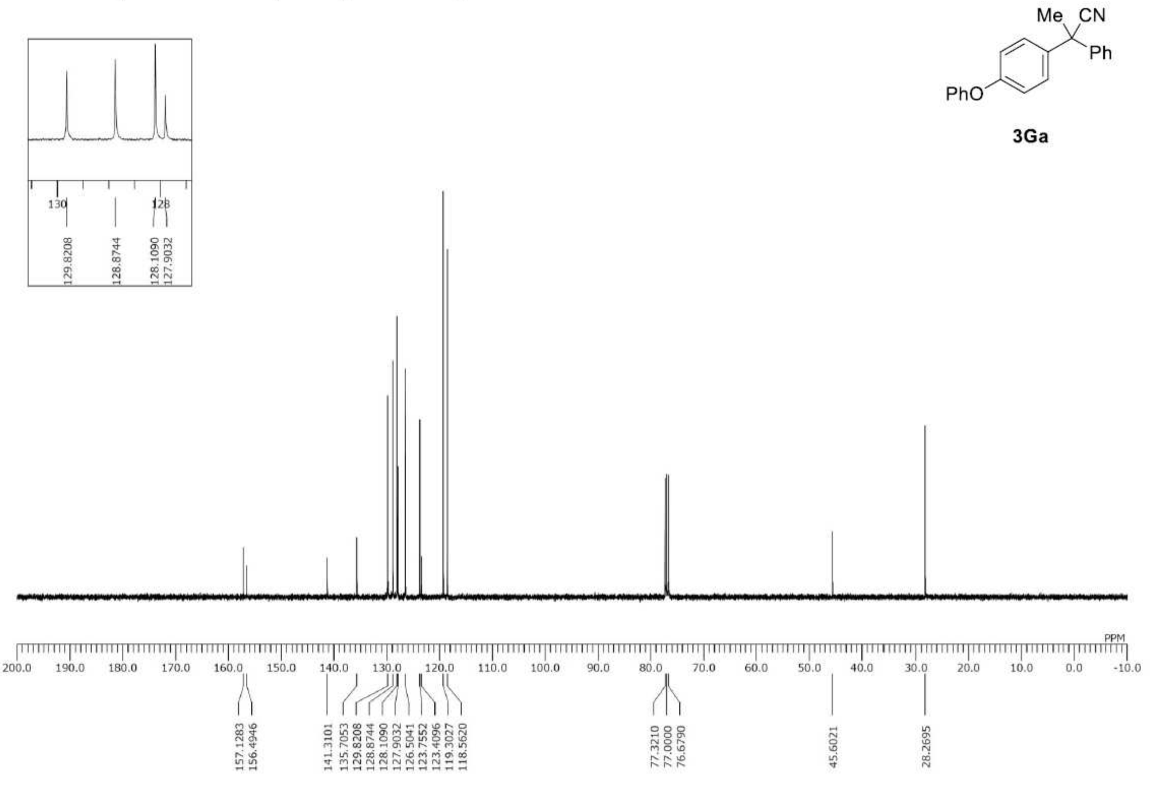

^{1}H NMR spectra of **3Ha** ($CDCl_3$, 400 MHz)

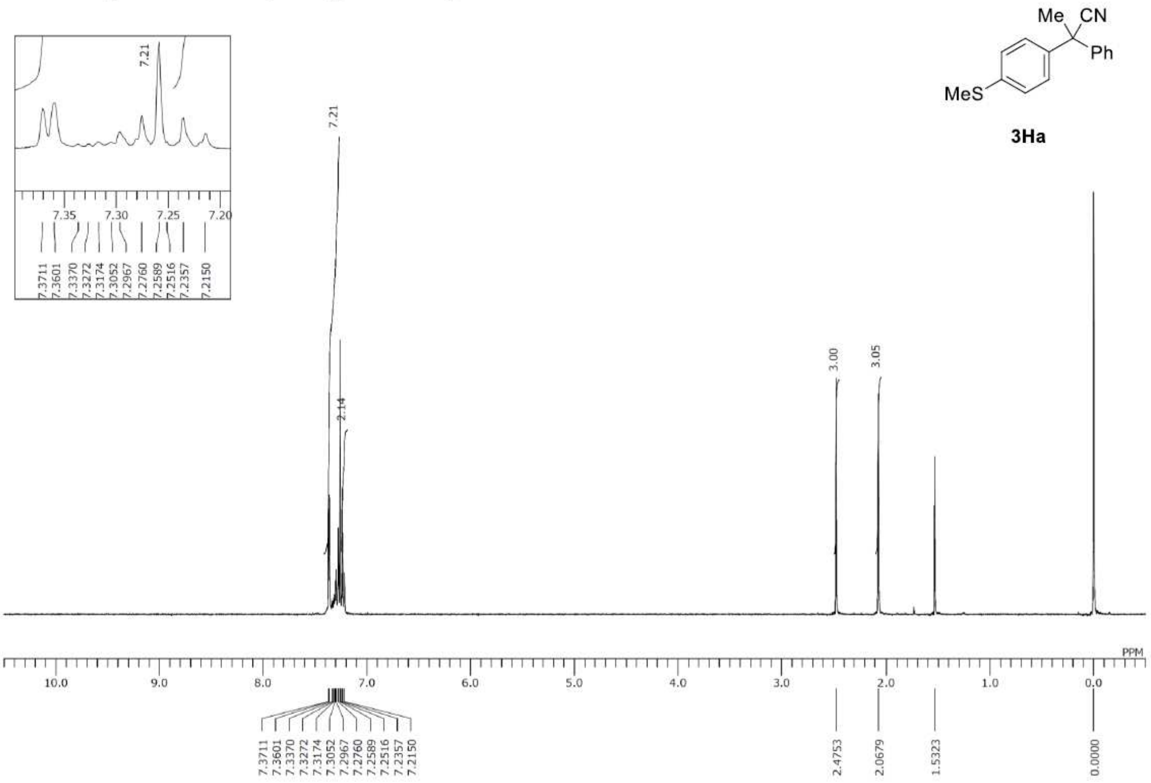

^{13}C NMR spectra of **3Ha** ($CDCl_3$, 100 MHz)

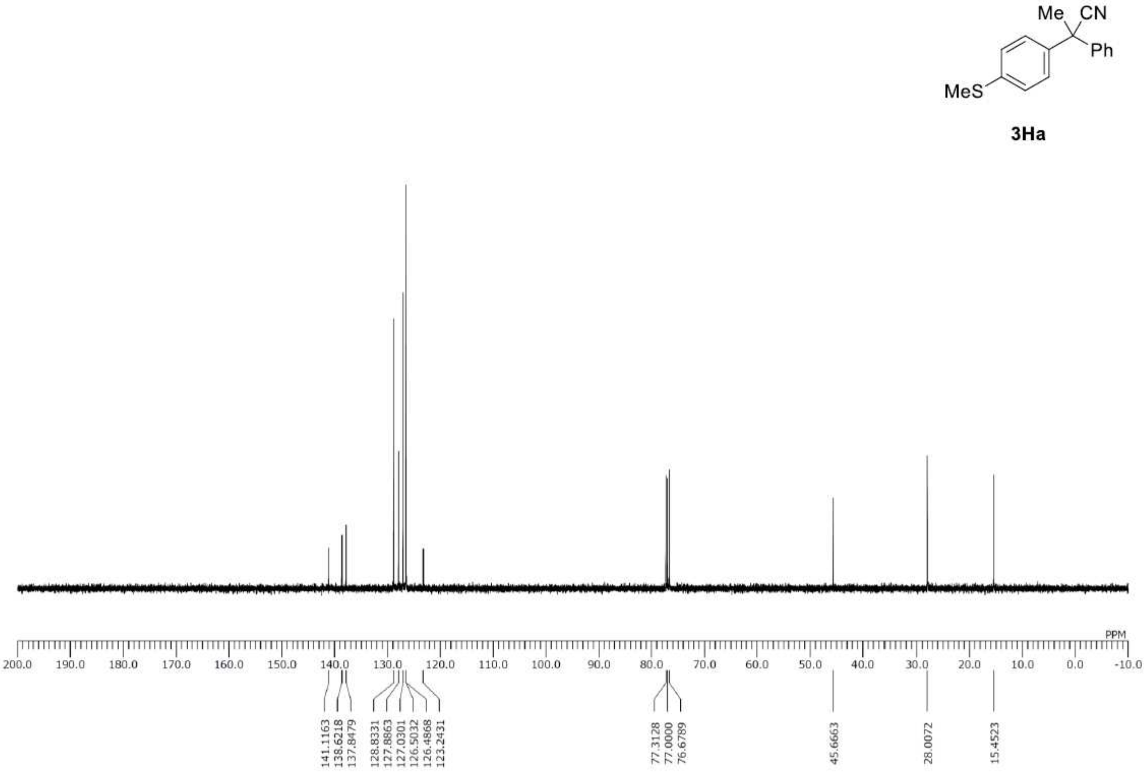

^{1}H NMR spectra of **3la** ($CDCl_3$, 400 MHz)

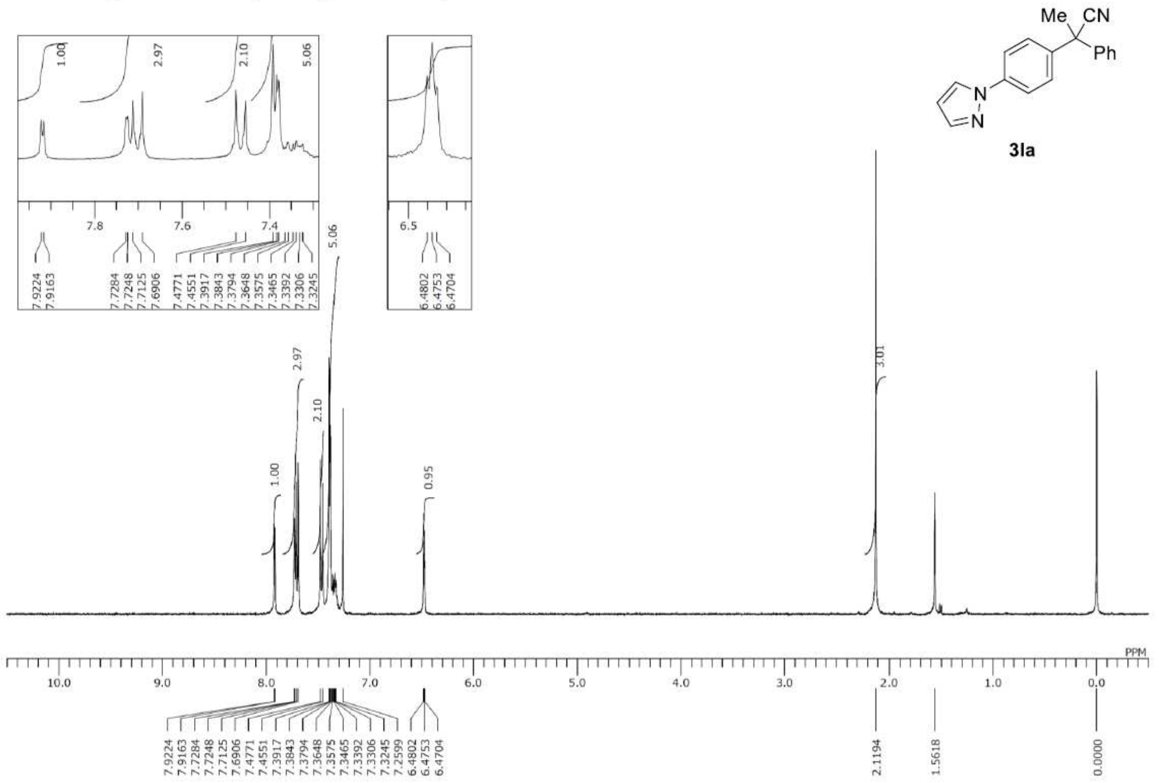

^{13}C NMR spectra of **3la** ($CDCl_3$, 150 MHz, 40 °C)

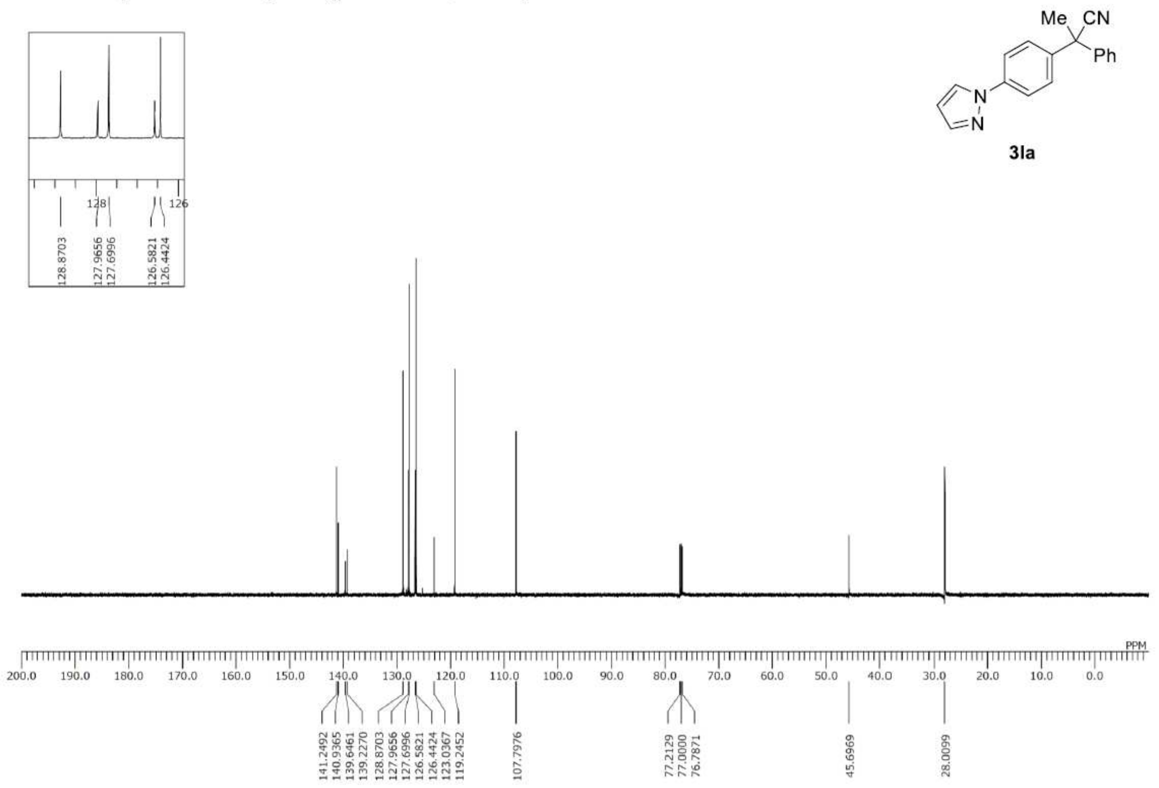

^{1}H NMR spectra of **3Ja** ($CDCl_3$, 400 MHz)

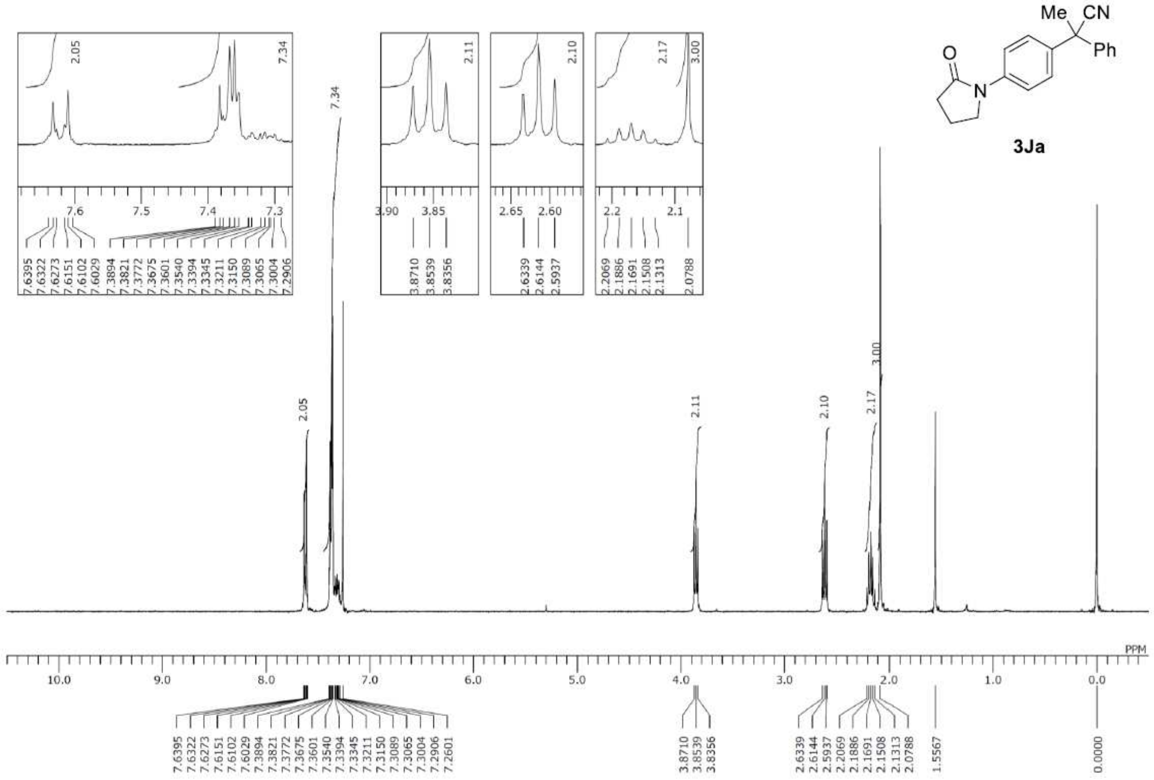

^{13}C NMR spectra of **3Ja** ($CDCl_3$, 150 MHz, 40 °C)

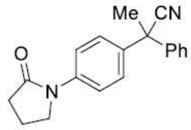

3Ja

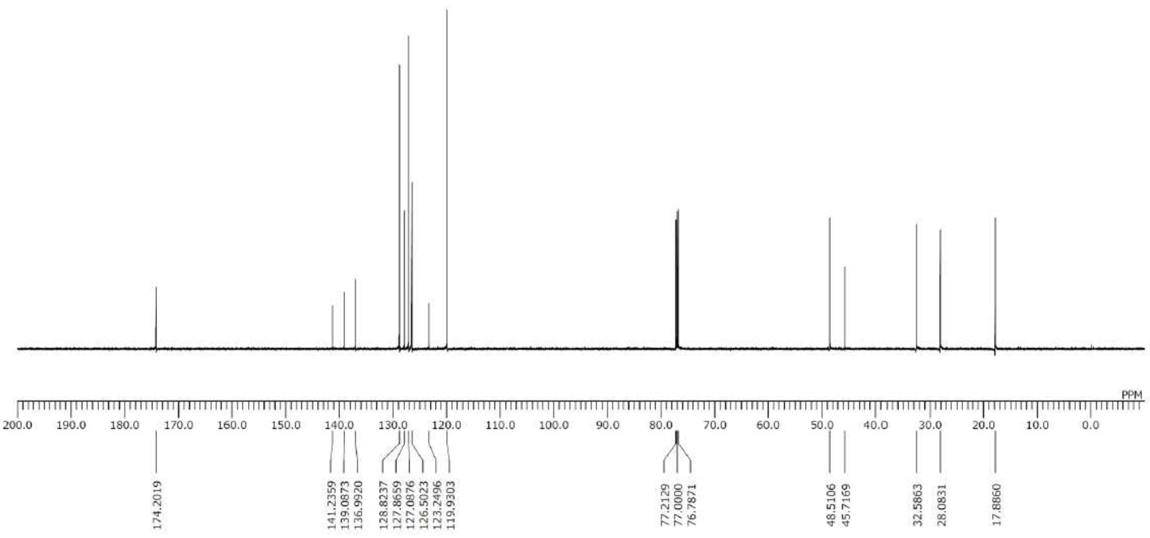

^{1}H NMR spectra of **3Ka** ($CDCl_3$, 400 MHz)

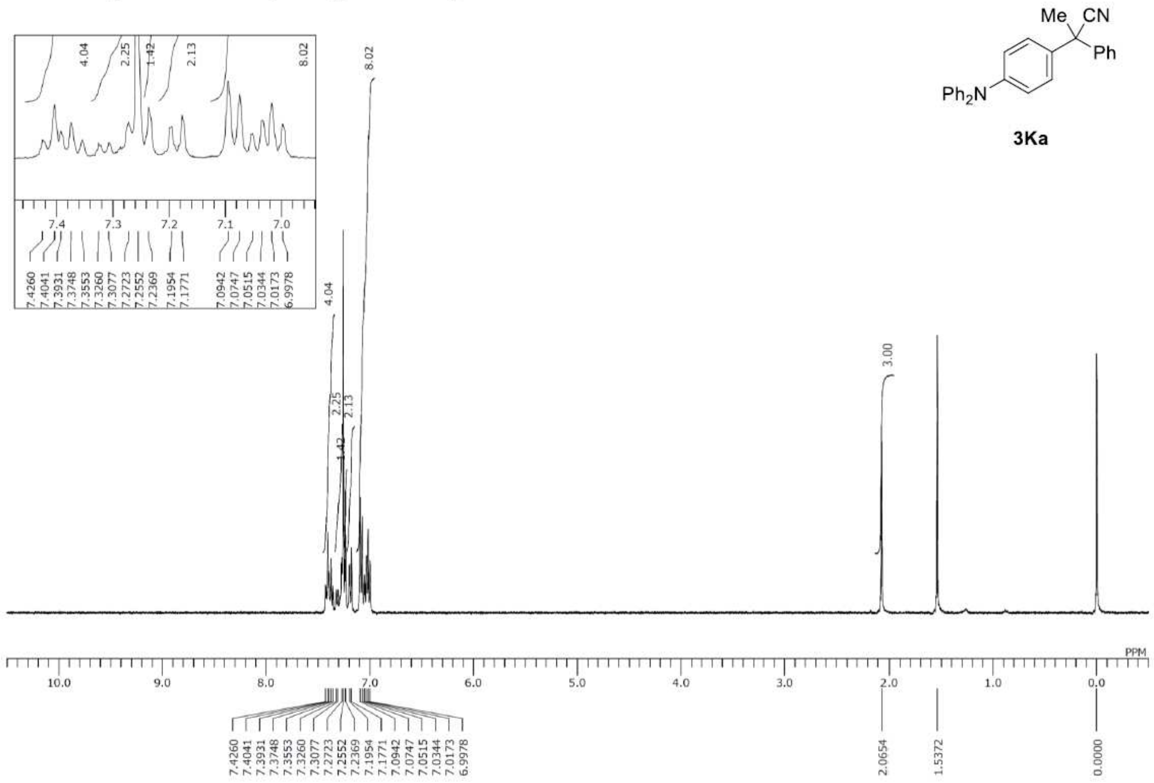

^{13}C NMR spectra of **3Ka** ($CDCl_3$, 100 MHz)

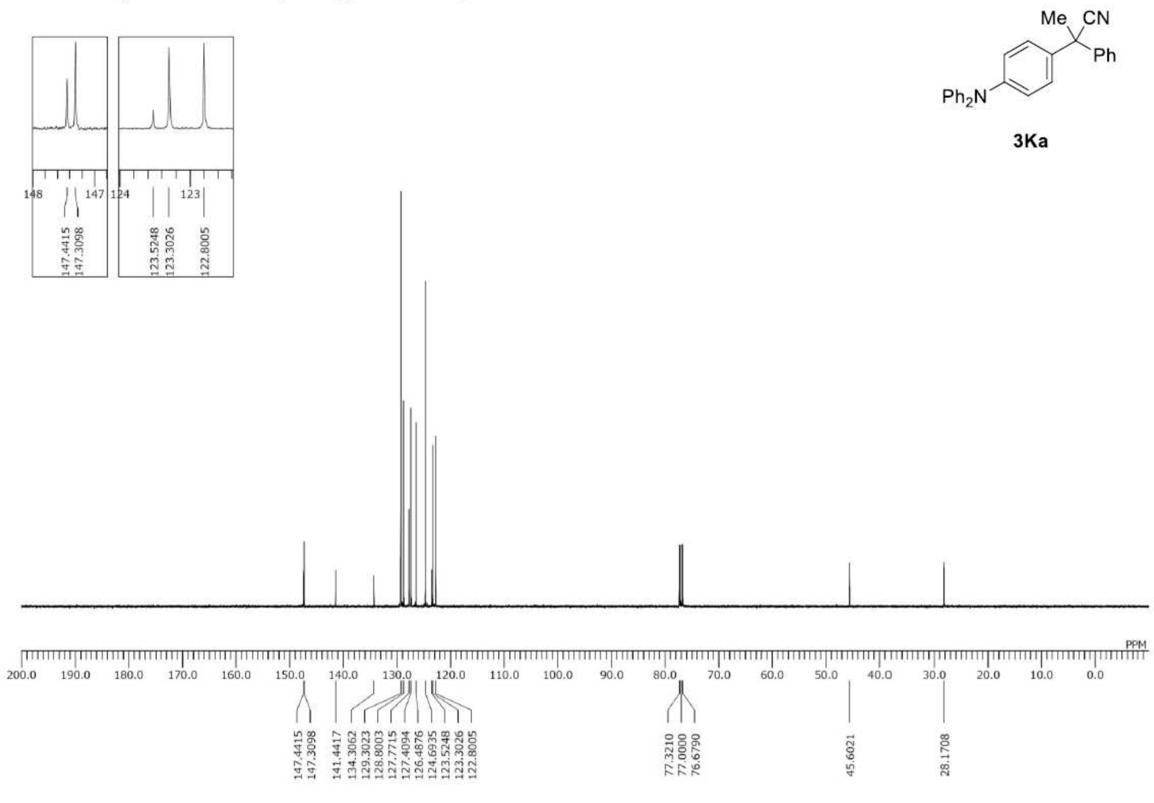

^{1}H NMR spectra of **3La** ($CDCl_3$, 400 MHz)

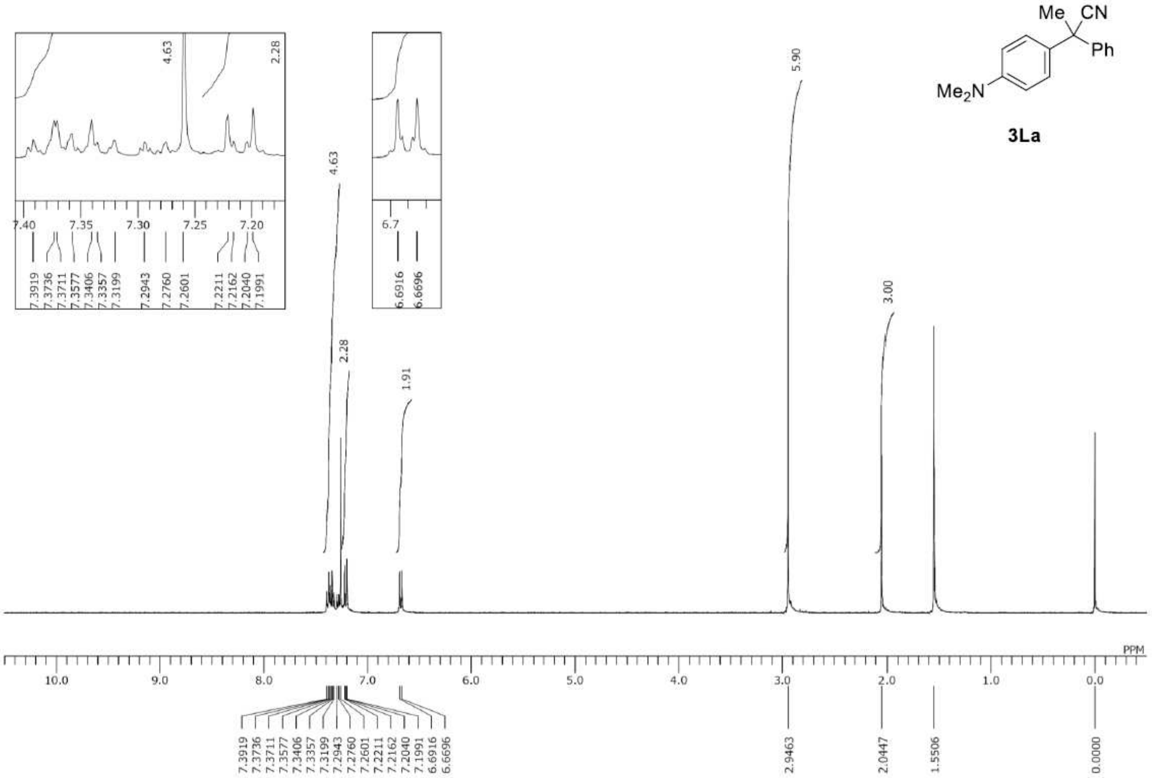

^{13}C NMR spectra of **3La** ($CDCl_3$, 100 MHz)

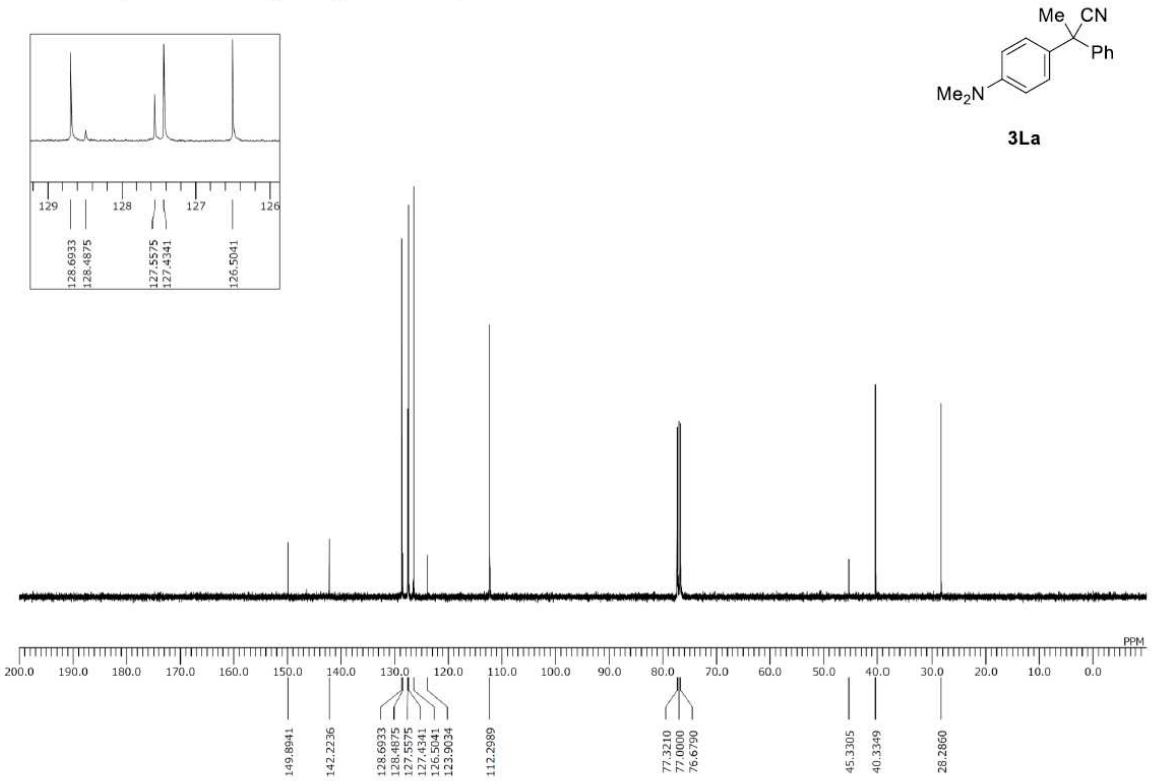

^{1}H NMR spectra of **3ab** ($CDCl_3$, 400 MHz)

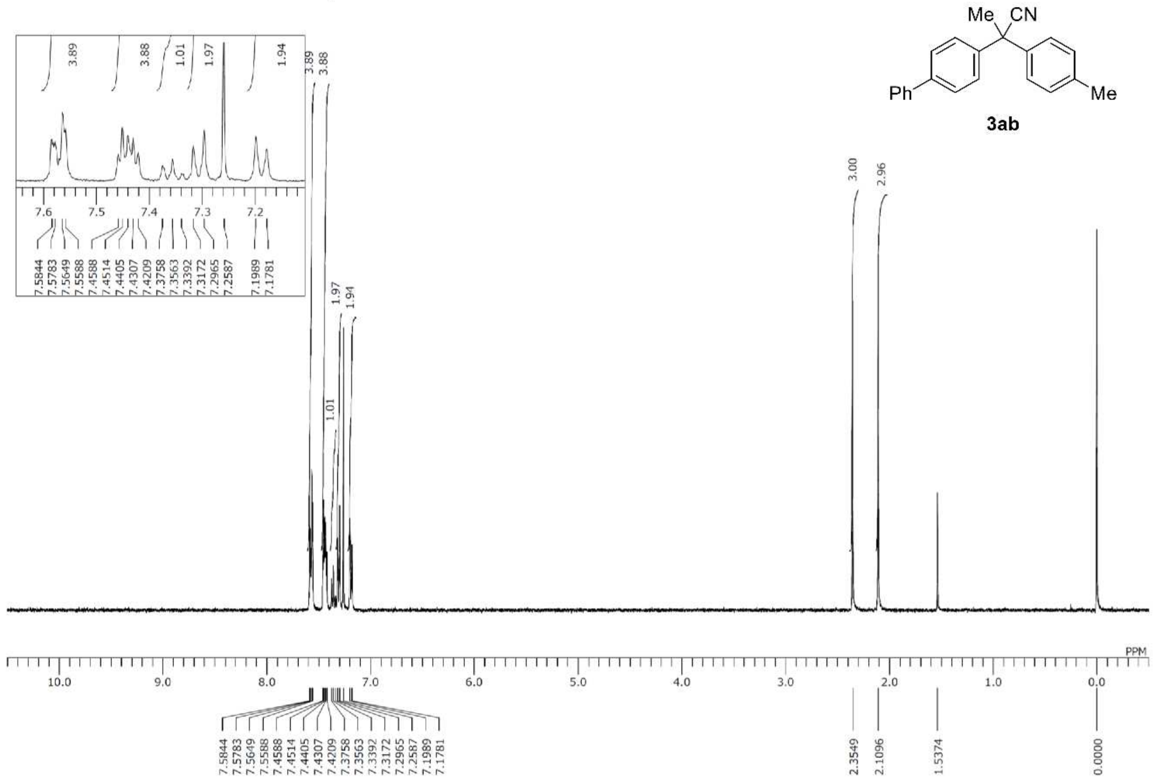

^{13}C NMR spectra of **3ab** ($CDCl_3$, 150 MHz)

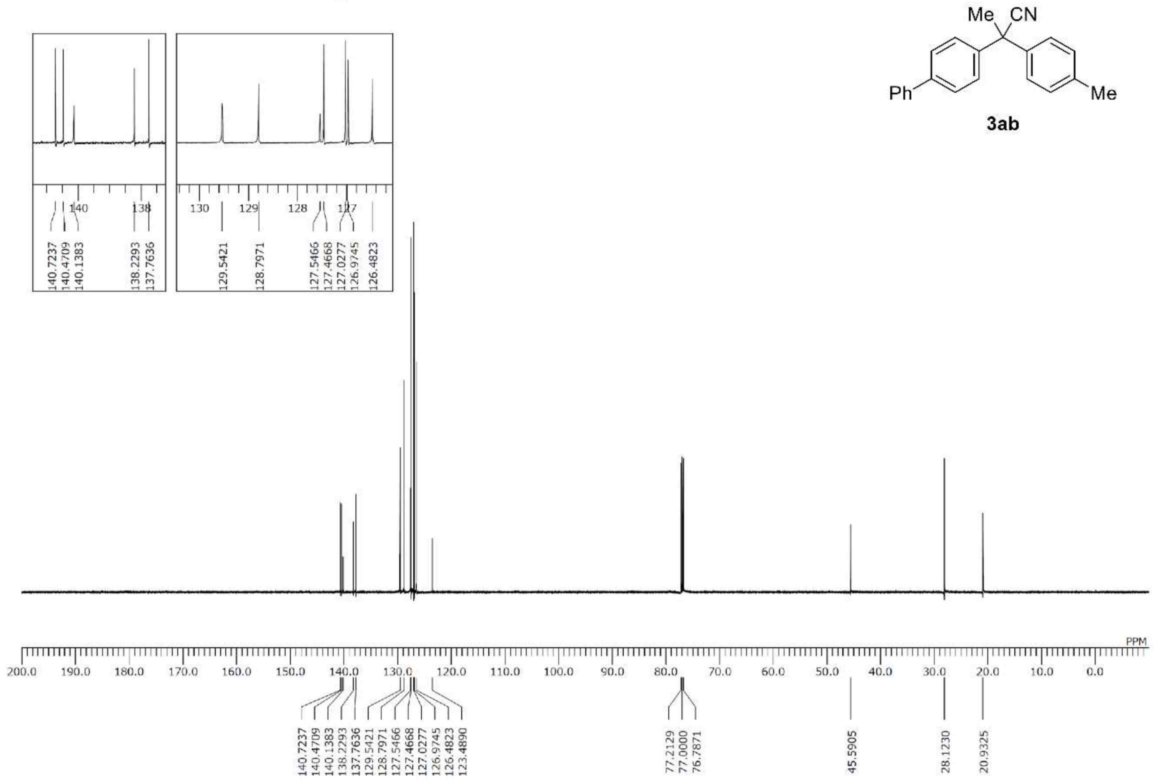

^{1}H NMR spectra of **3ac** ($CDCl_3$, 400 MHz)

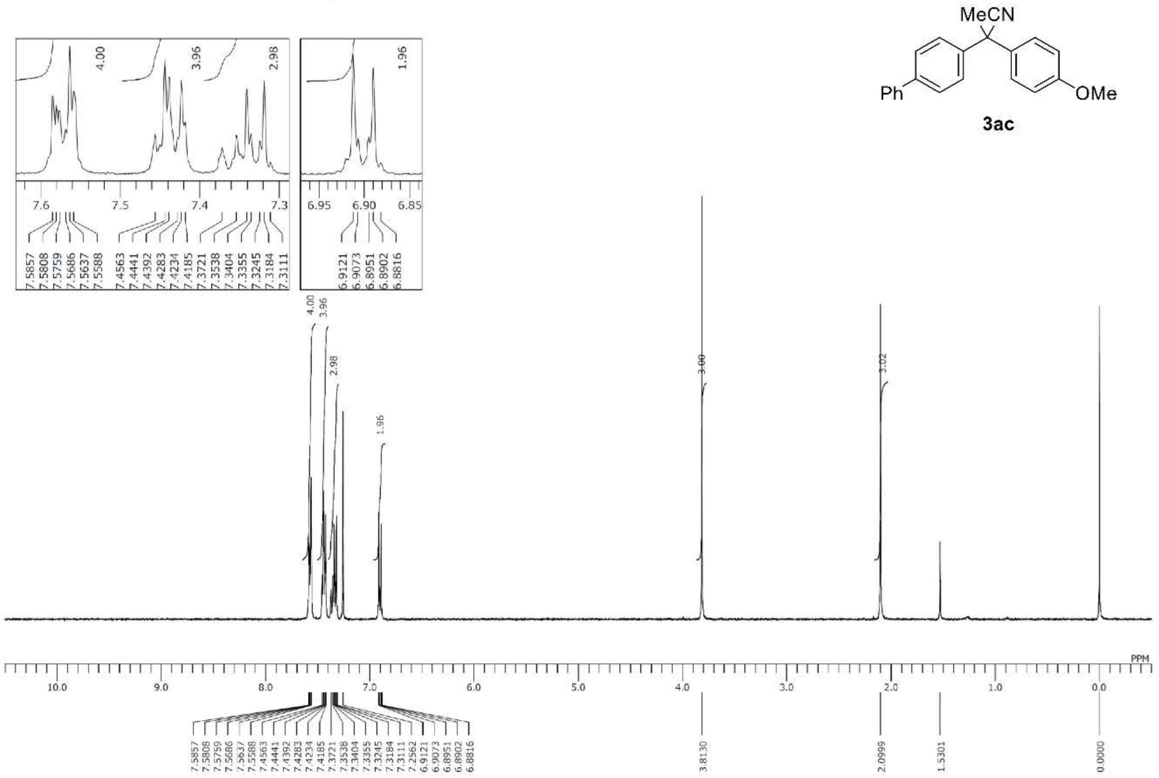

^{13}C NMR spectra of **3ac** ($CDCl_3$, 150 MHz)

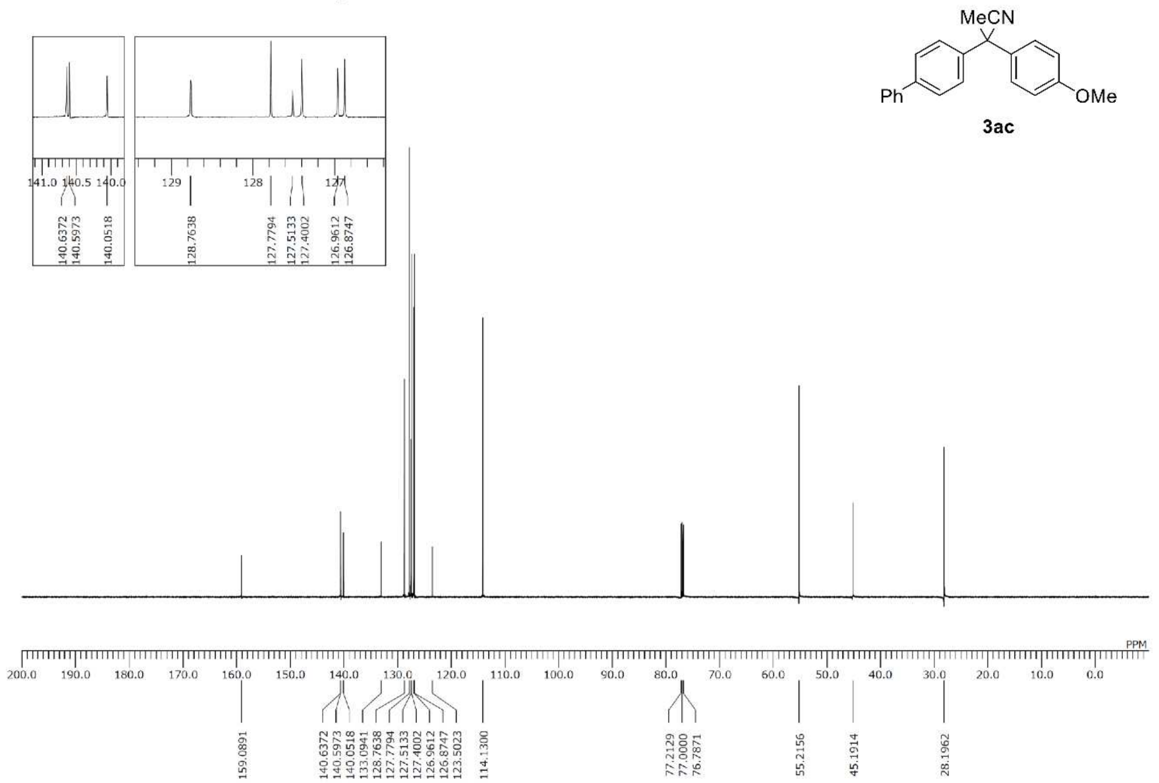

^{1}H NMR spectra of **3ad** ($CDCl_3$, 400 MHz)

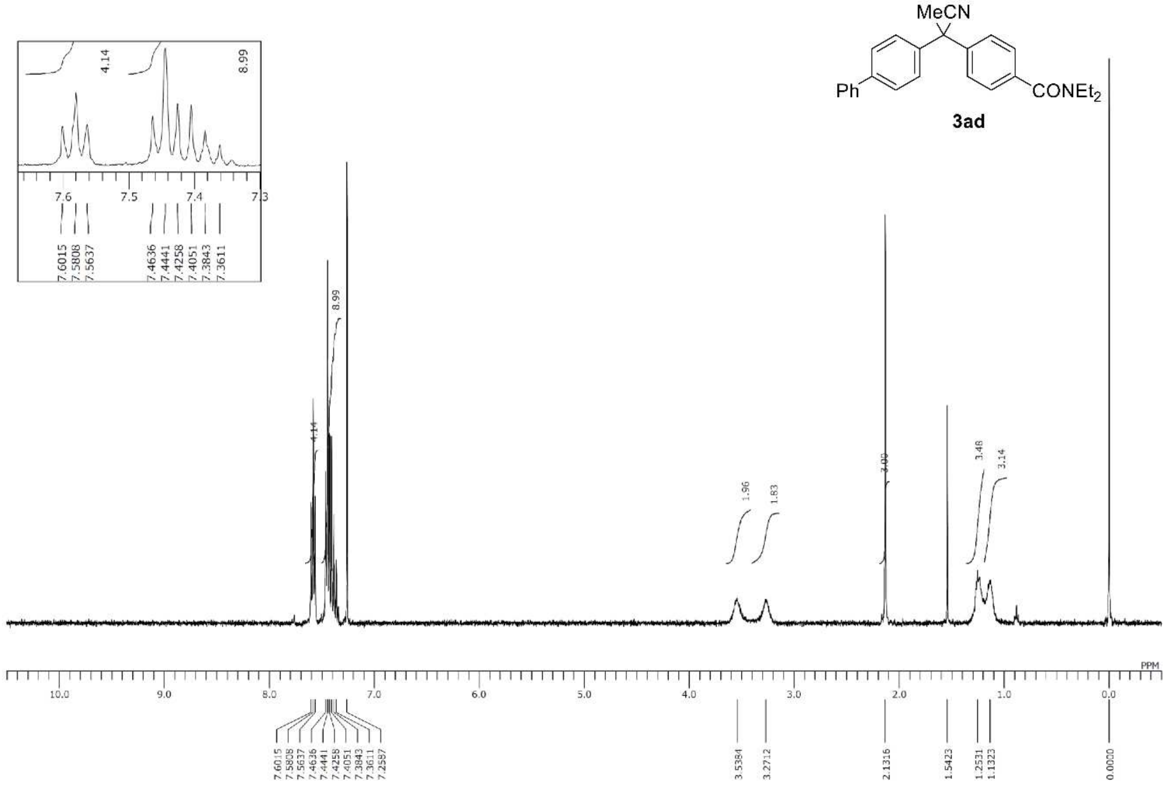

^{13}C NMR spectra of **3ad** ($CDCl_3$, 100 MHz)

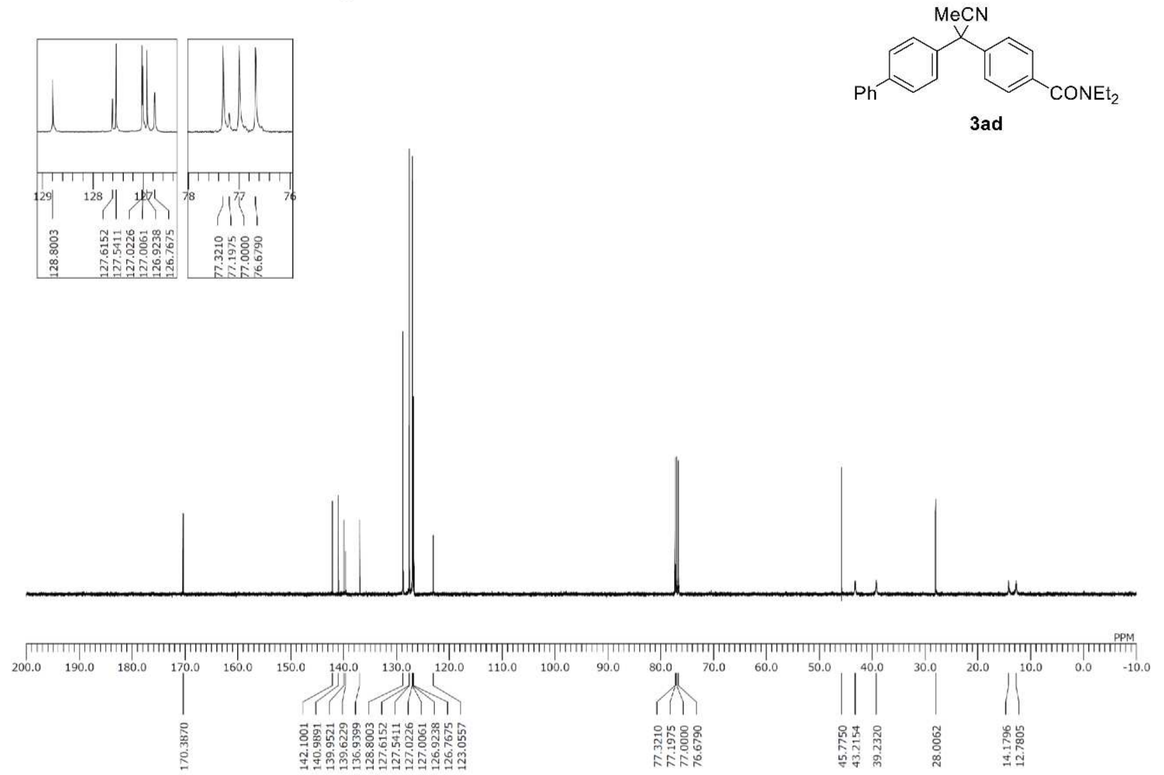

^{1}H NMR spectra of **3ae** ($CDCl_3$, 400 MHz)

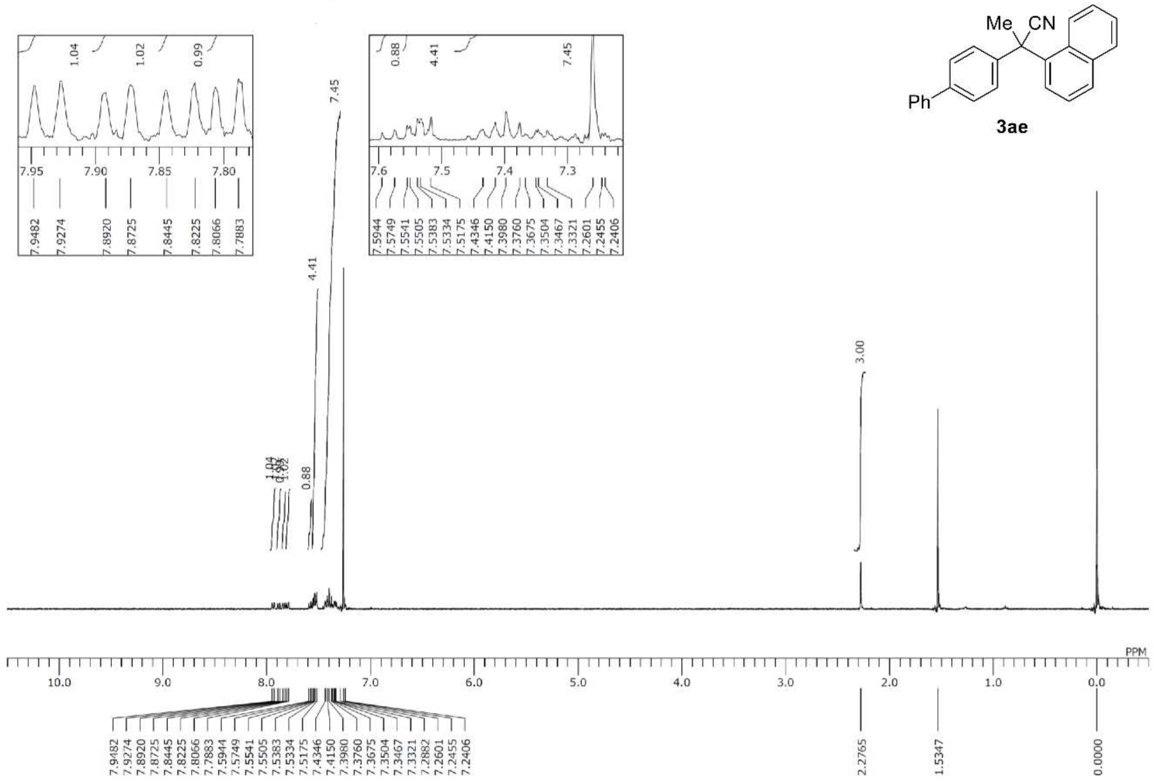

^{13}C NMR spectra of **3ae** ($CDCl_3$, 150 MHz)

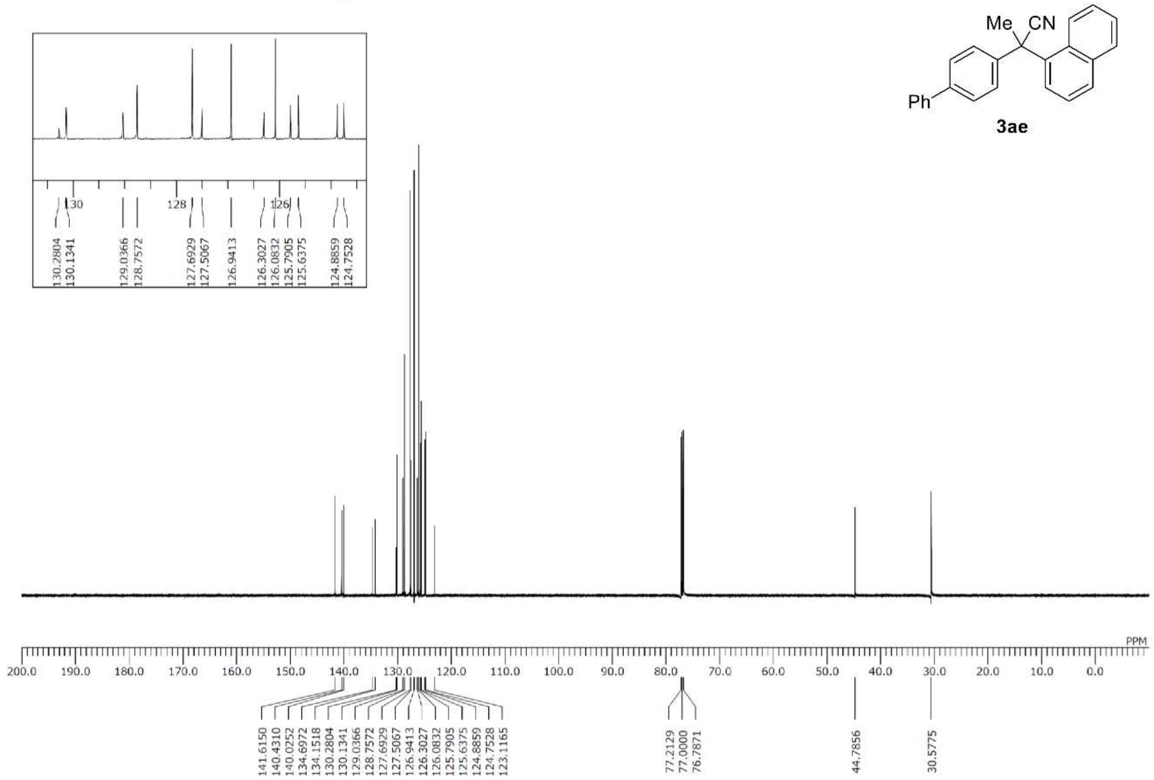

^{1}H NMR spectra of **3af** ($CDCl_3$, 400 MHz)

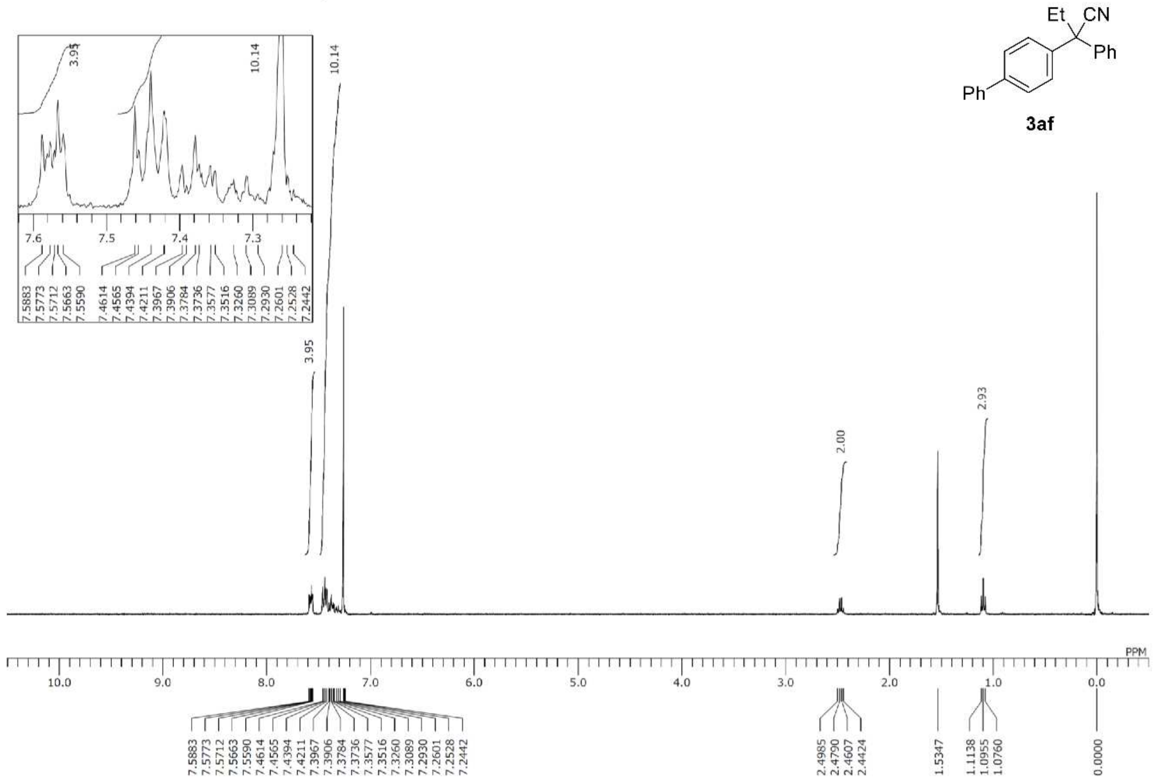

^{13}C NMR spectra of **3af** ($CDCl_3$, 100 MHz)

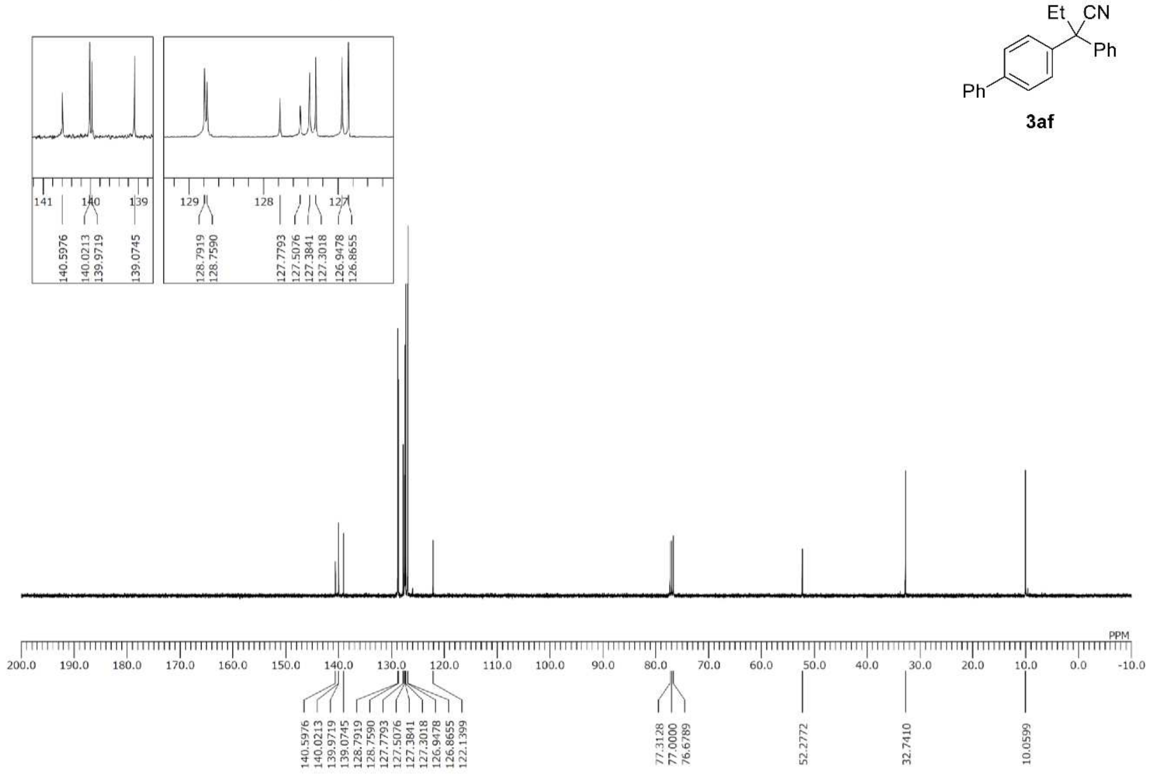

^{1}H NMR spectra of **3ag** ($CDCl_3$, 400 MHz)

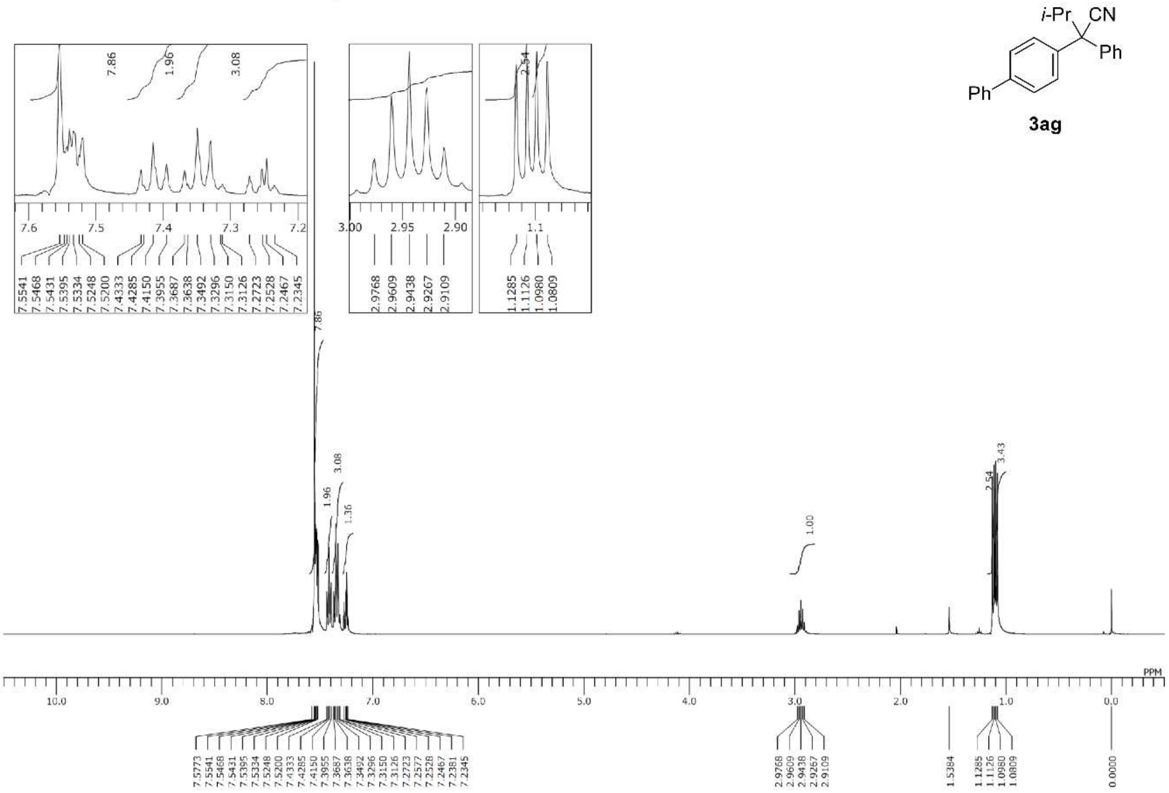

^{13}C NMR spectra of **3ag** ($CDCl_3$, 150 MHz)

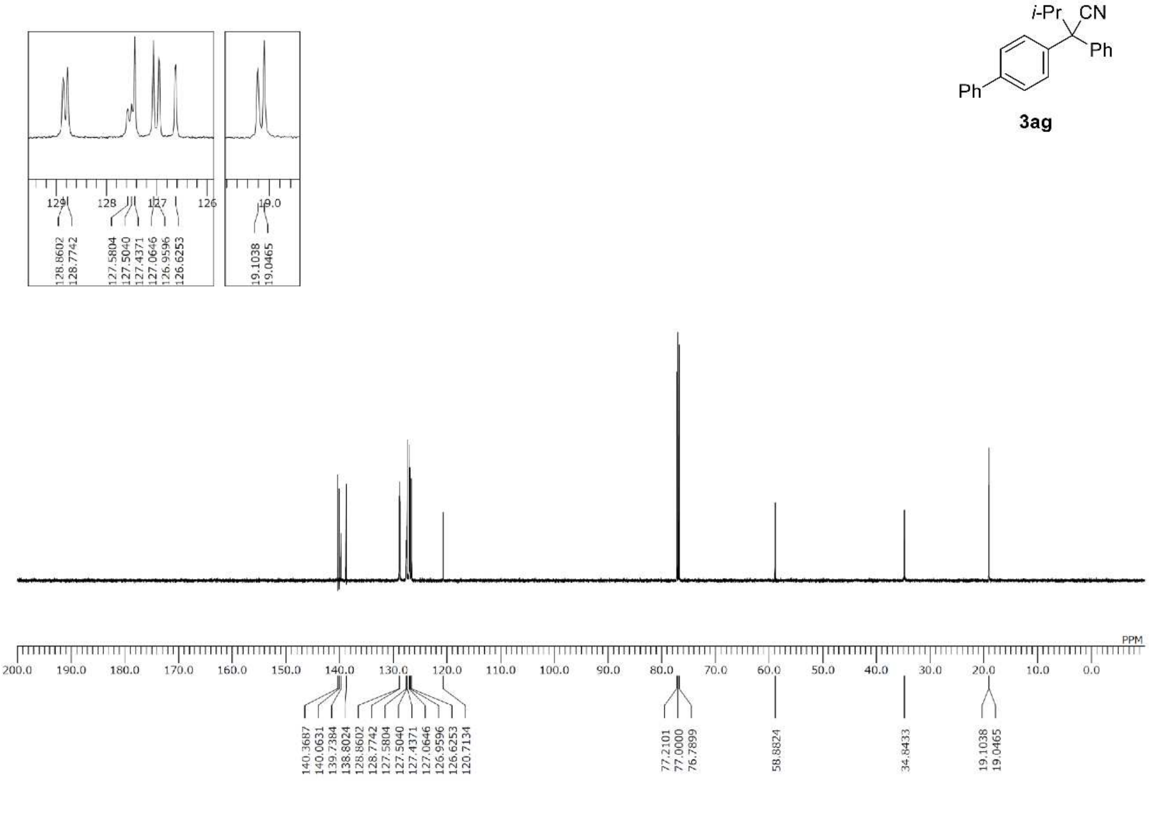

^{1}H NMR spectra of **3ah** ($CDCl_3$, 400 MHz)

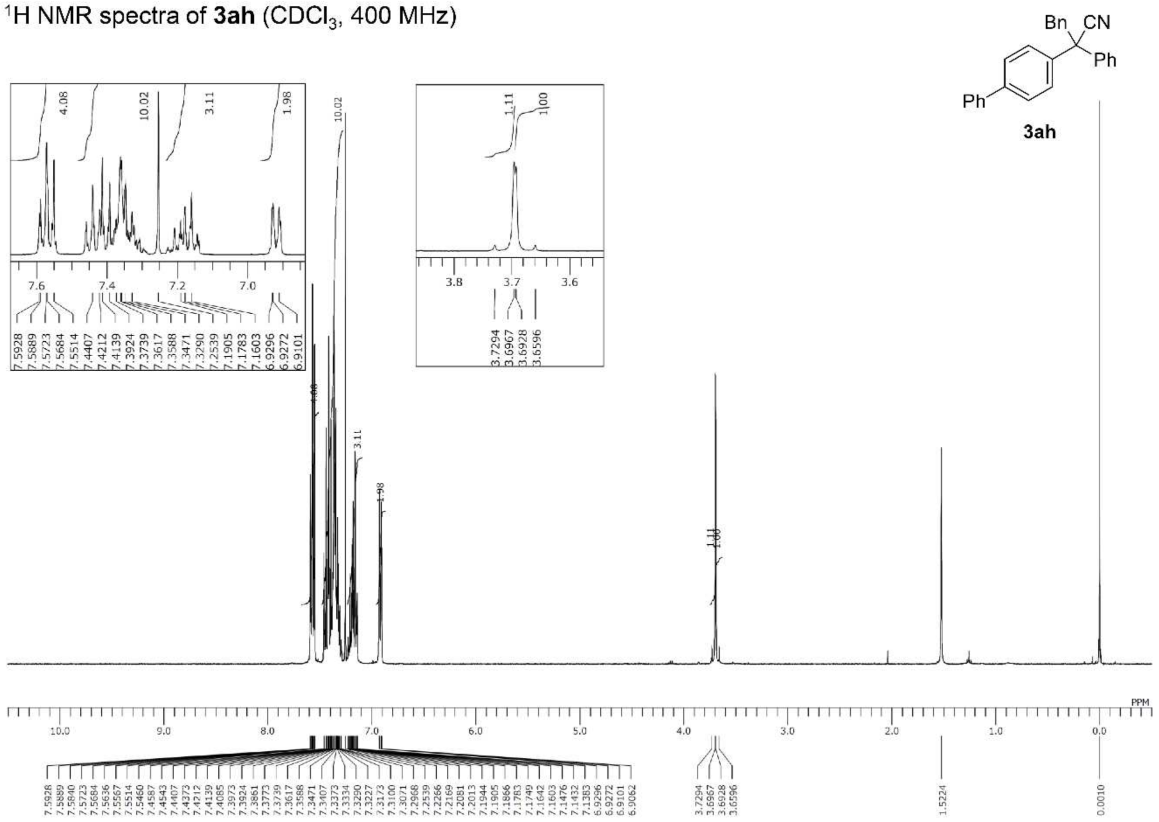

^{13}C NMR spectra of **3ah** ($CDCl_3$, 150 MHz)

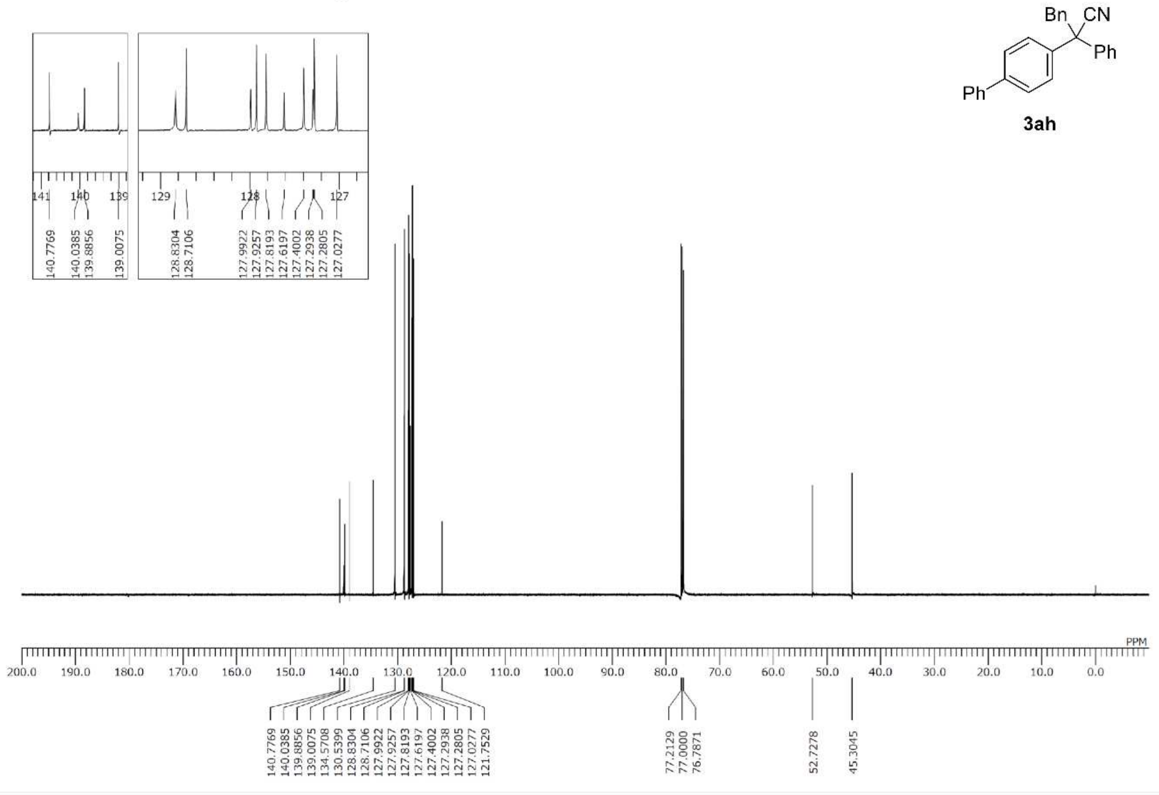

^{1}H NMR spectra of **3ai** ($CDCl_3$, 400 MHz)

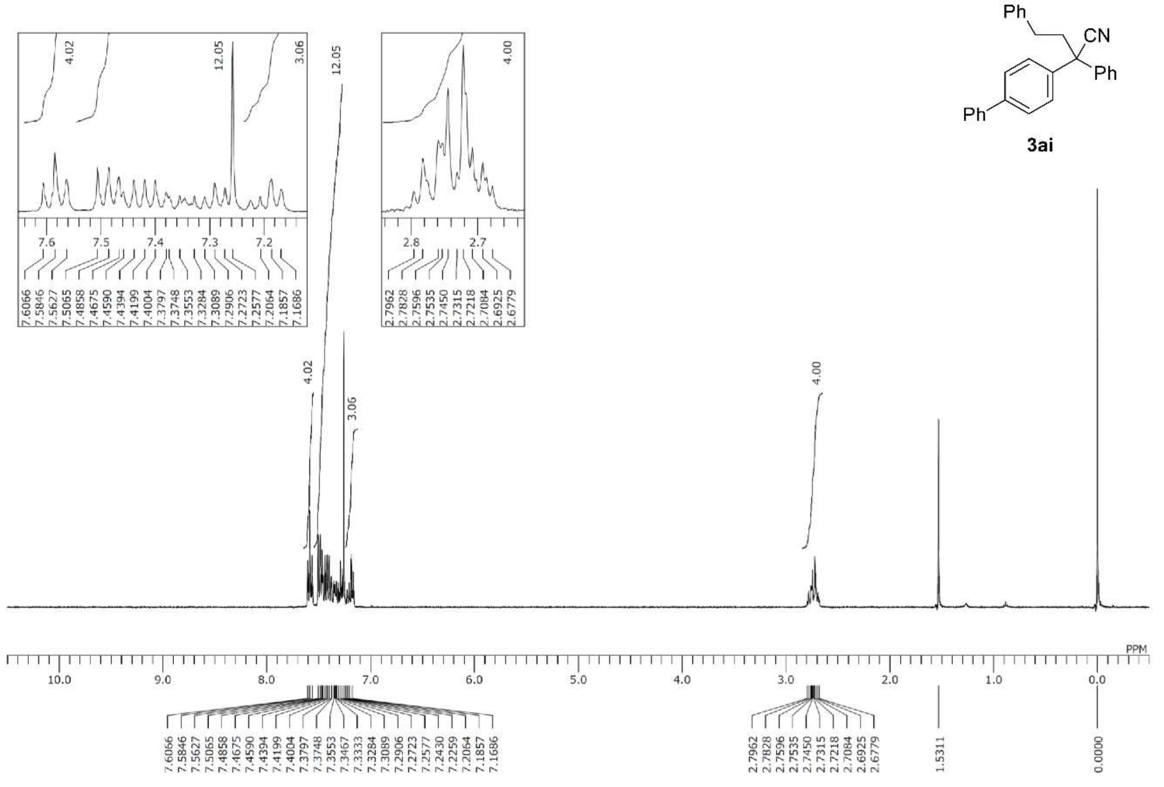

^{13}C NMR spectra of **3ai** ($CDCl_3$, 150 MHz)

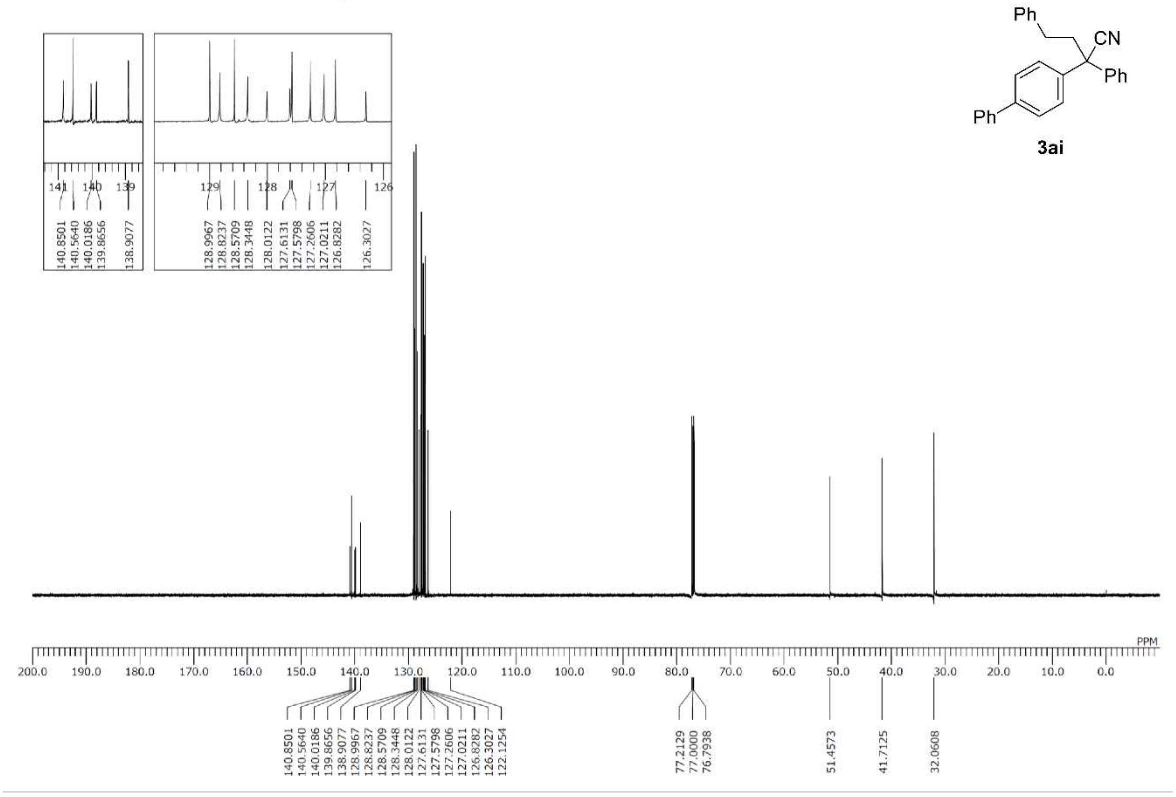

^{1}H NMR spectra of **3aj** ($CDCl_3$, 400 MHz)

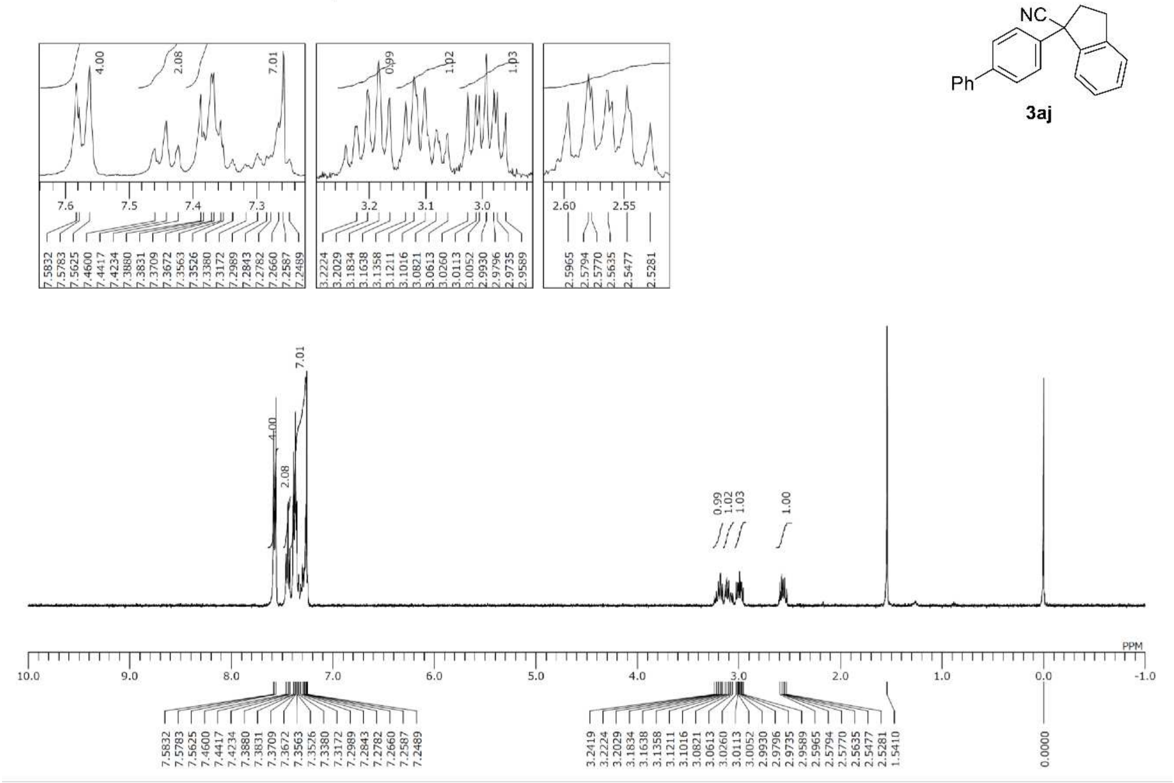

^{13}C NMR spectra of **3aj** ($CDCl_3$, 150 MHz)

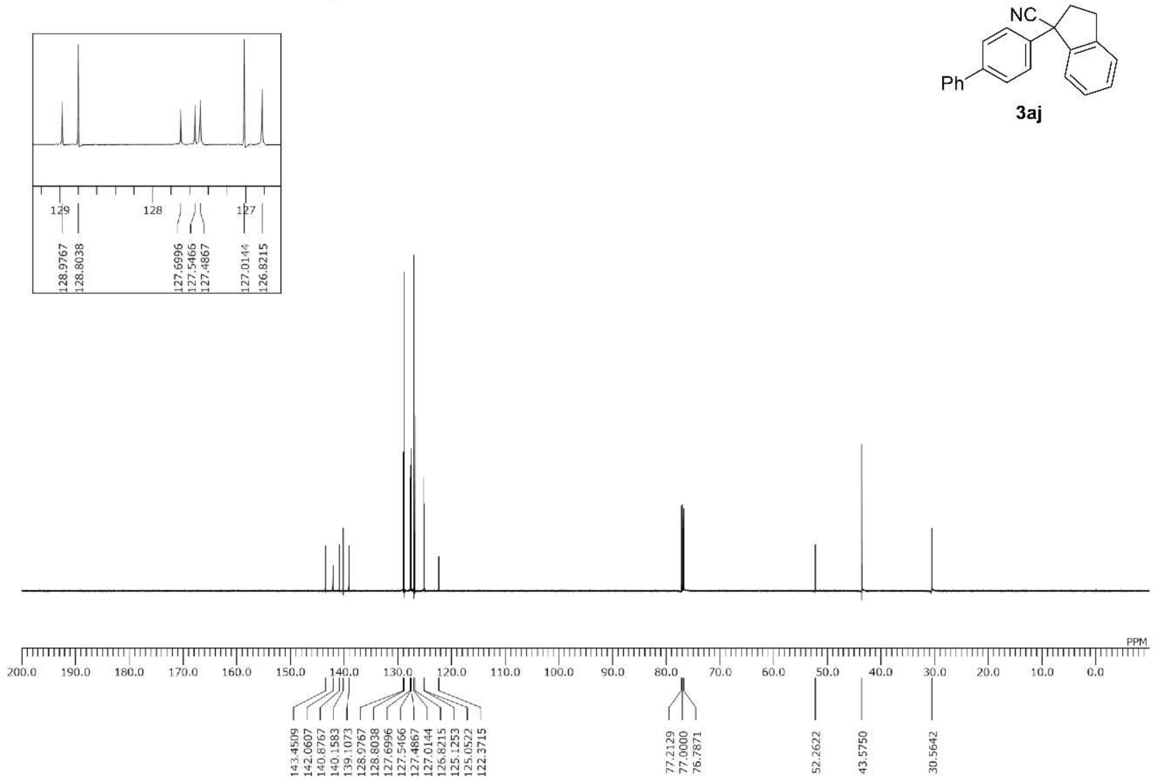

^{1}H NMR spectra of **3ak** ($CDCl_3$, 400 MHz)

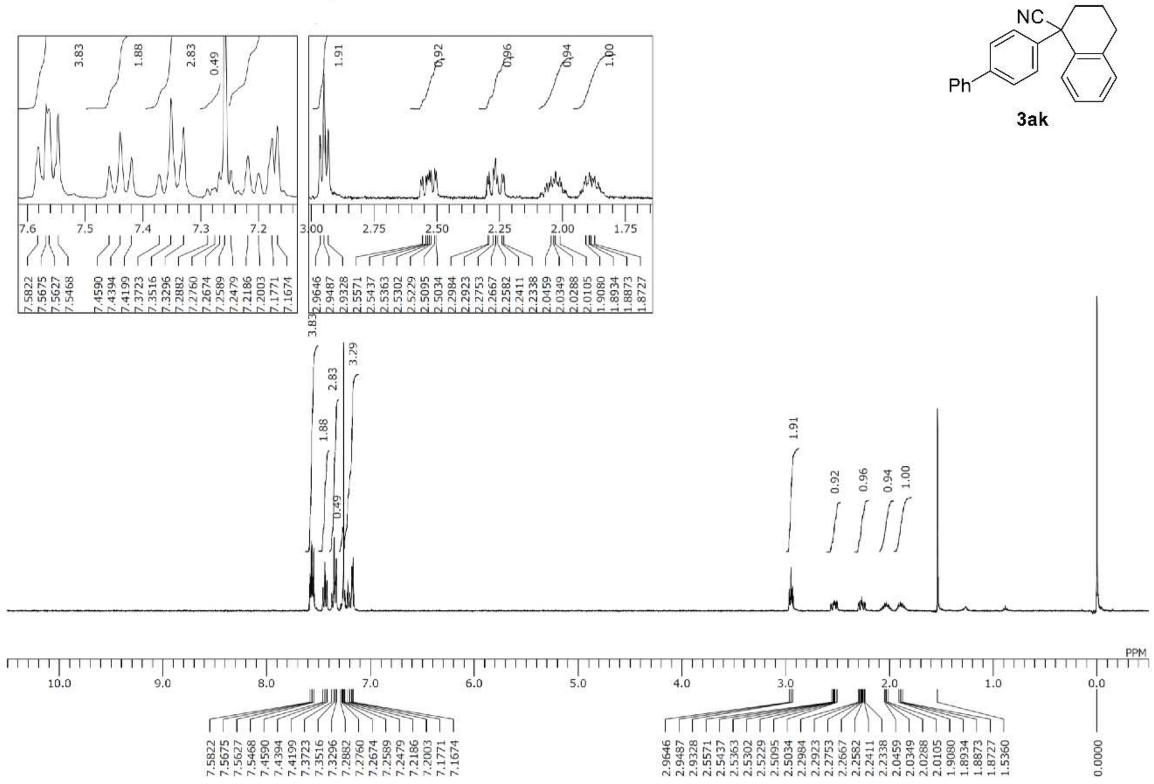

^{13}C NMR spectra of **3ak** ($CDCl_3$, 100 MHz)

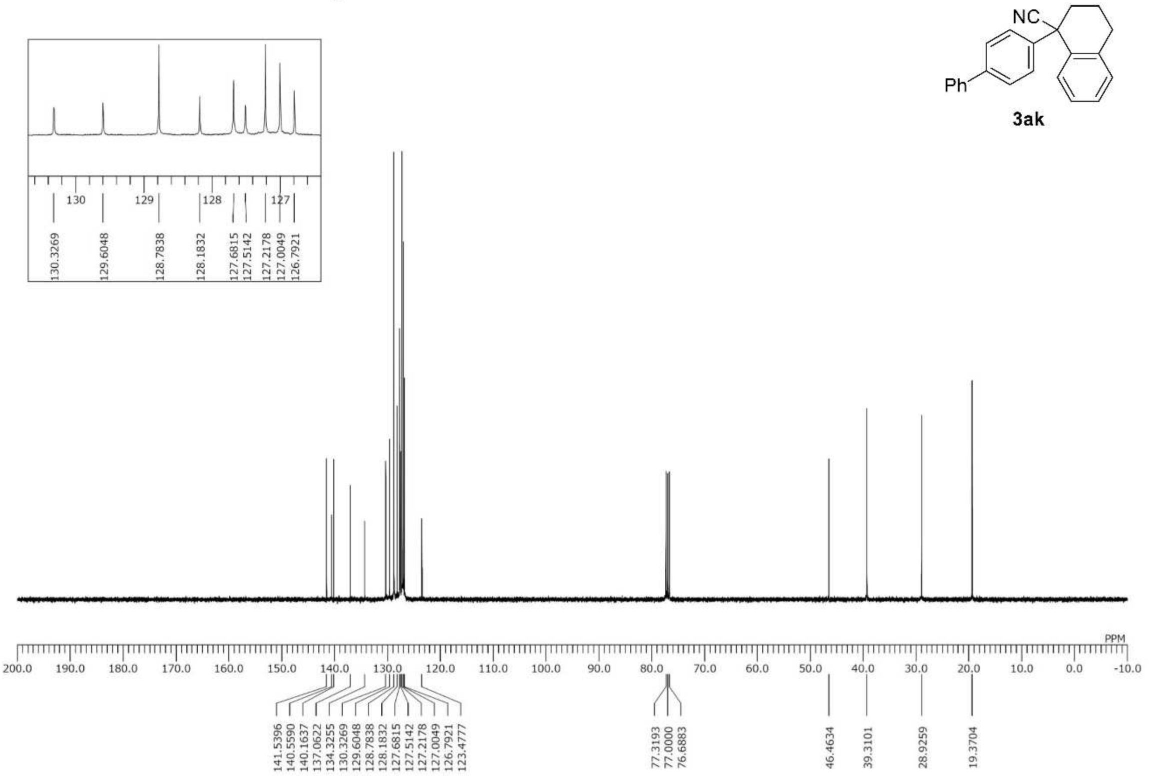

^{1}H NMR spectra of **3al** ($CDCl_3$, 400 MHz)

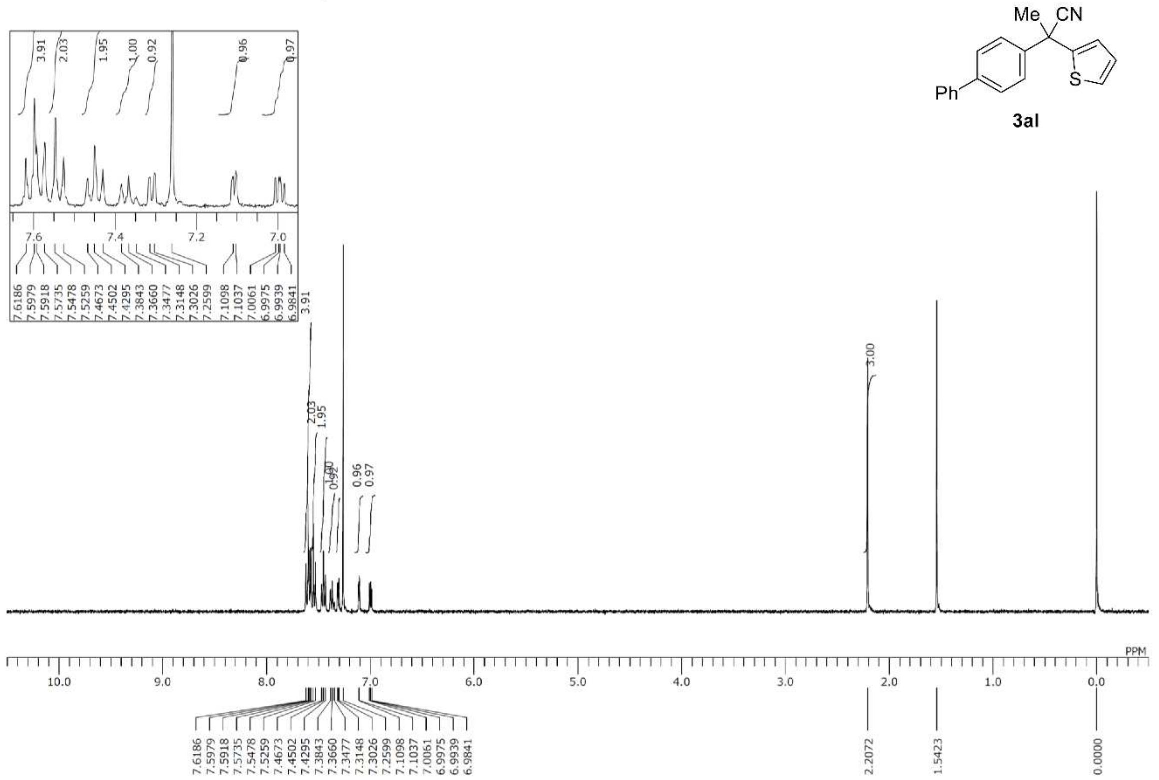

3al

^{13}C NMR spectra of **3al** ($CDCl_3$, 150 MHz)

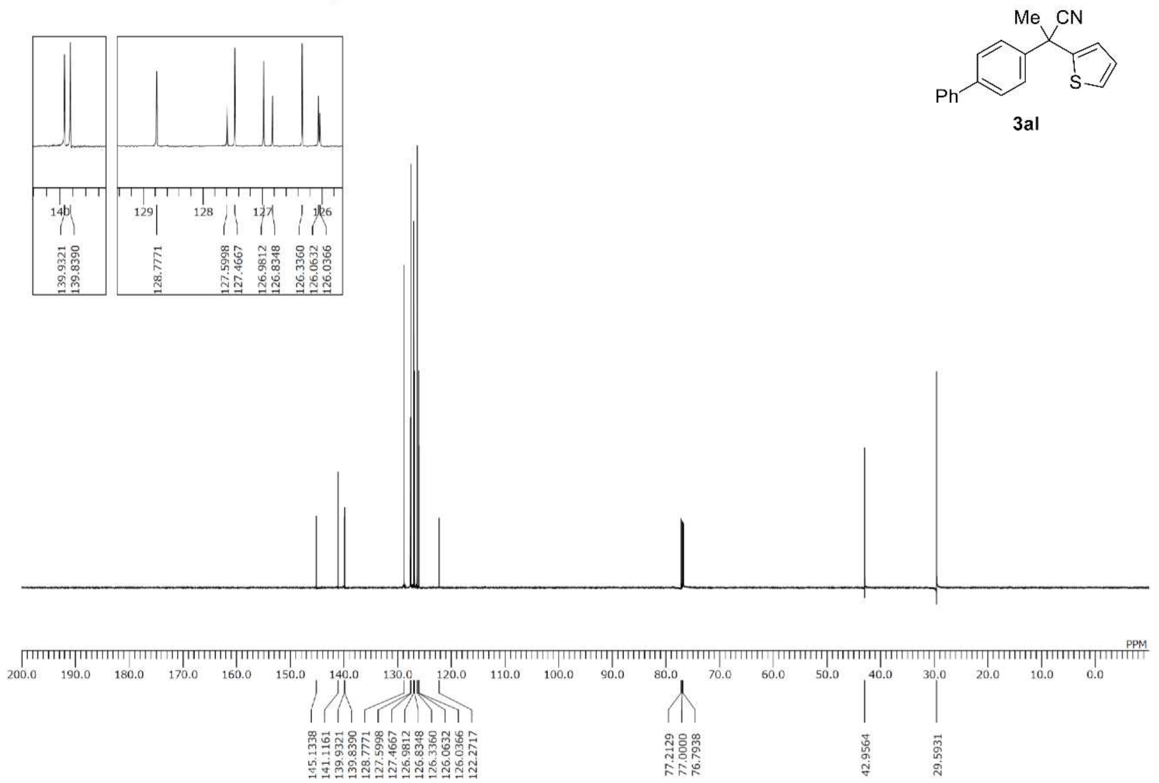

3al

^{1}H NMR spectra of **3am** ($CDCl_3$, 400 MHz)

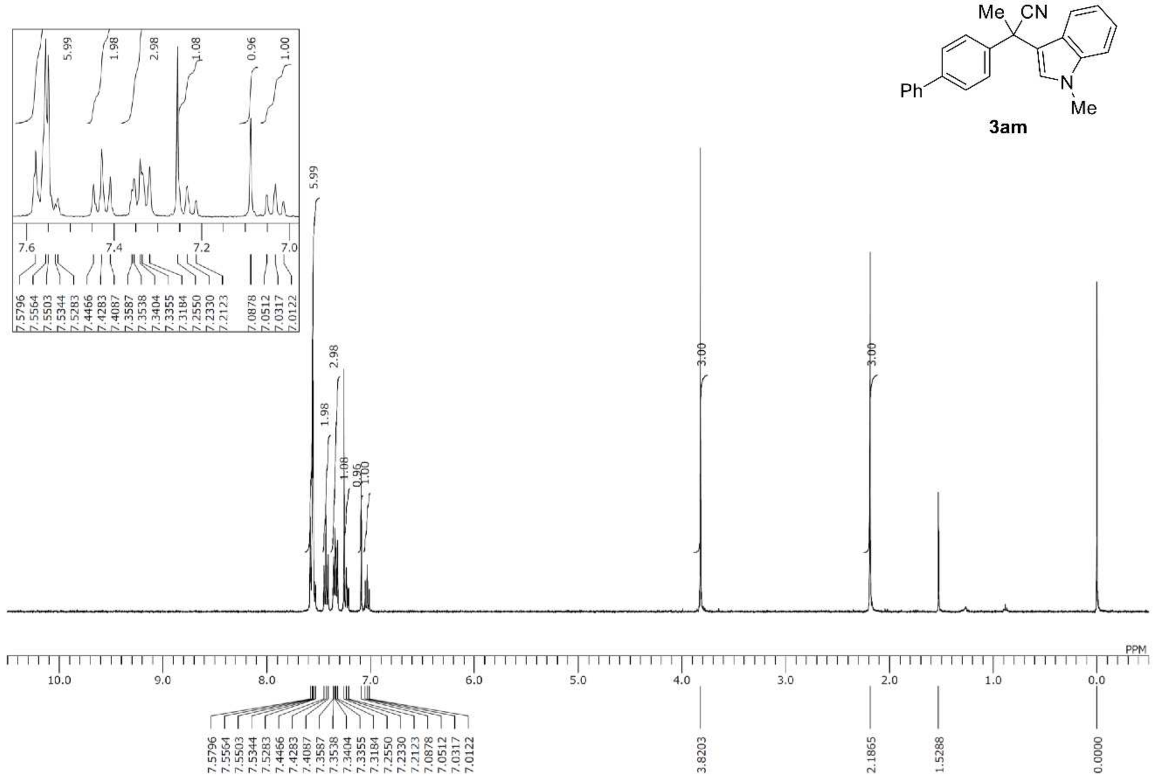

^{13}C NMR spectra of **3am** ($CDCl_3$, 150 MHz)

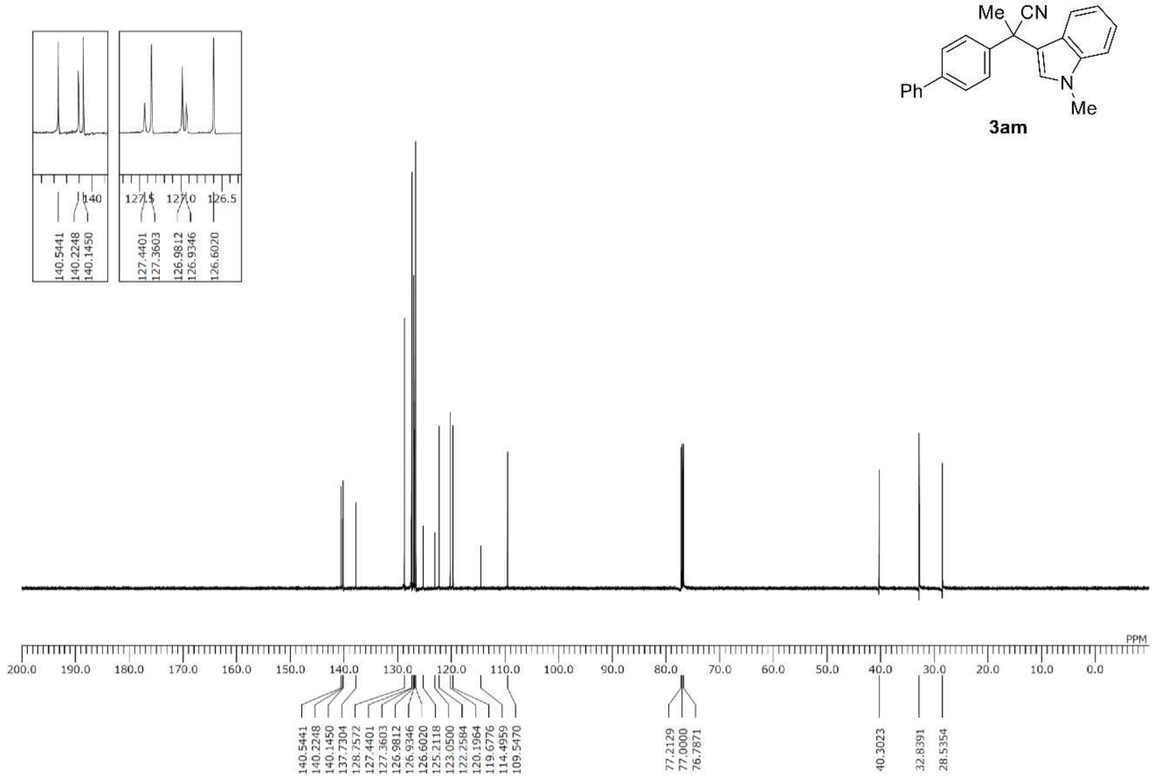

^{1}H NMR spectra of **3an** ($CDCl_3$, 400 MHz)

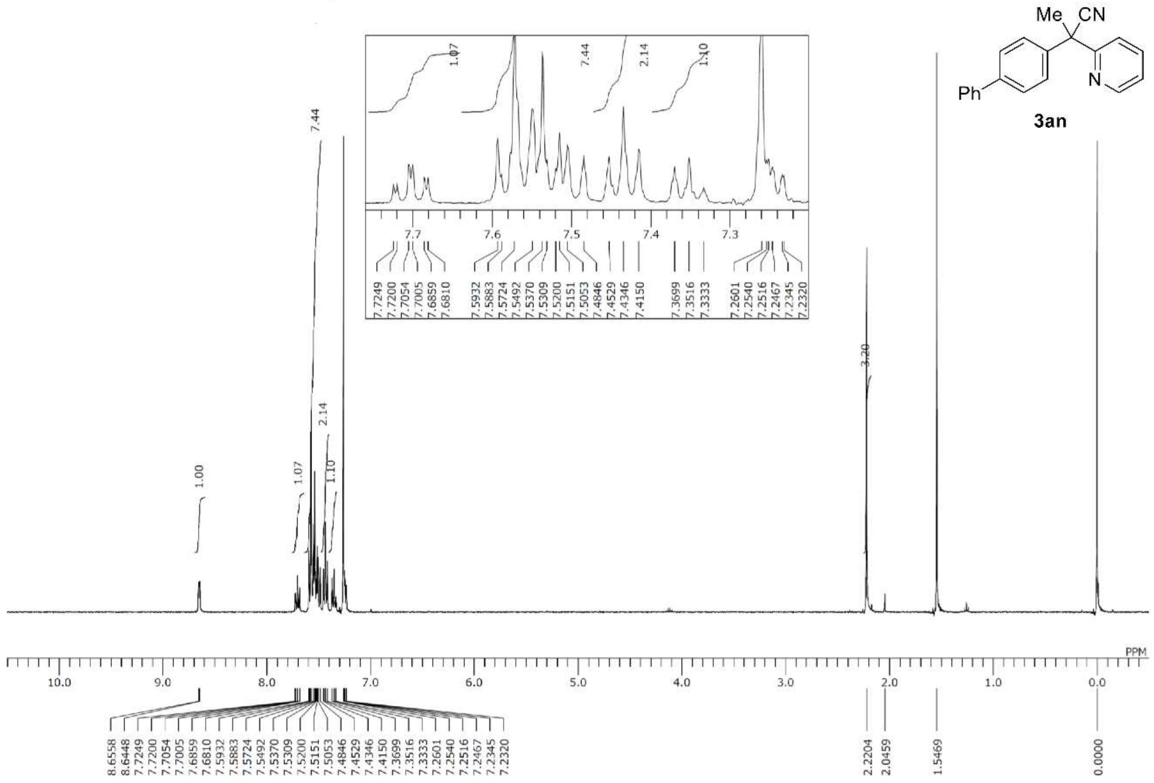

^{13}C NMR spectra of **3an** (DMSO-d_6, 100 MHz)

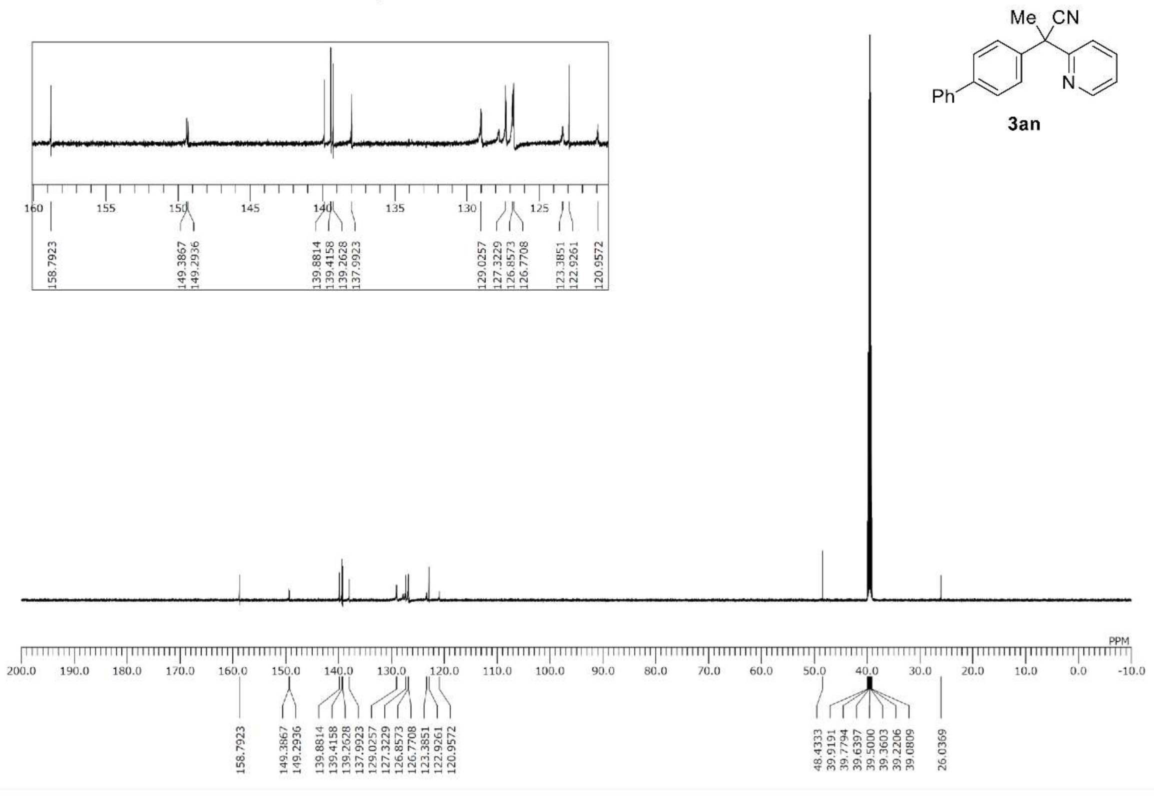

^{1}H NMR spectra of **3ao** ($CDCl_3$, 400 MHz)

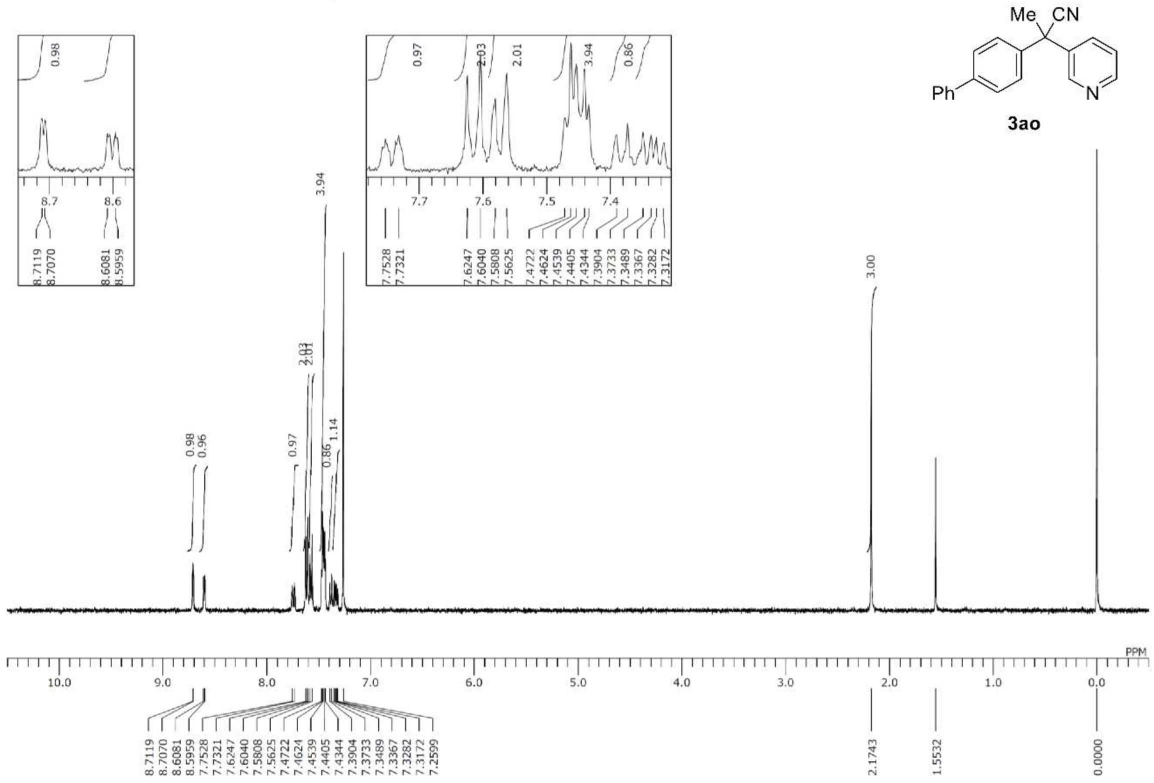

^{13}C NMR spectra of **3ao** ($CDCl_3$, 150 MHz)

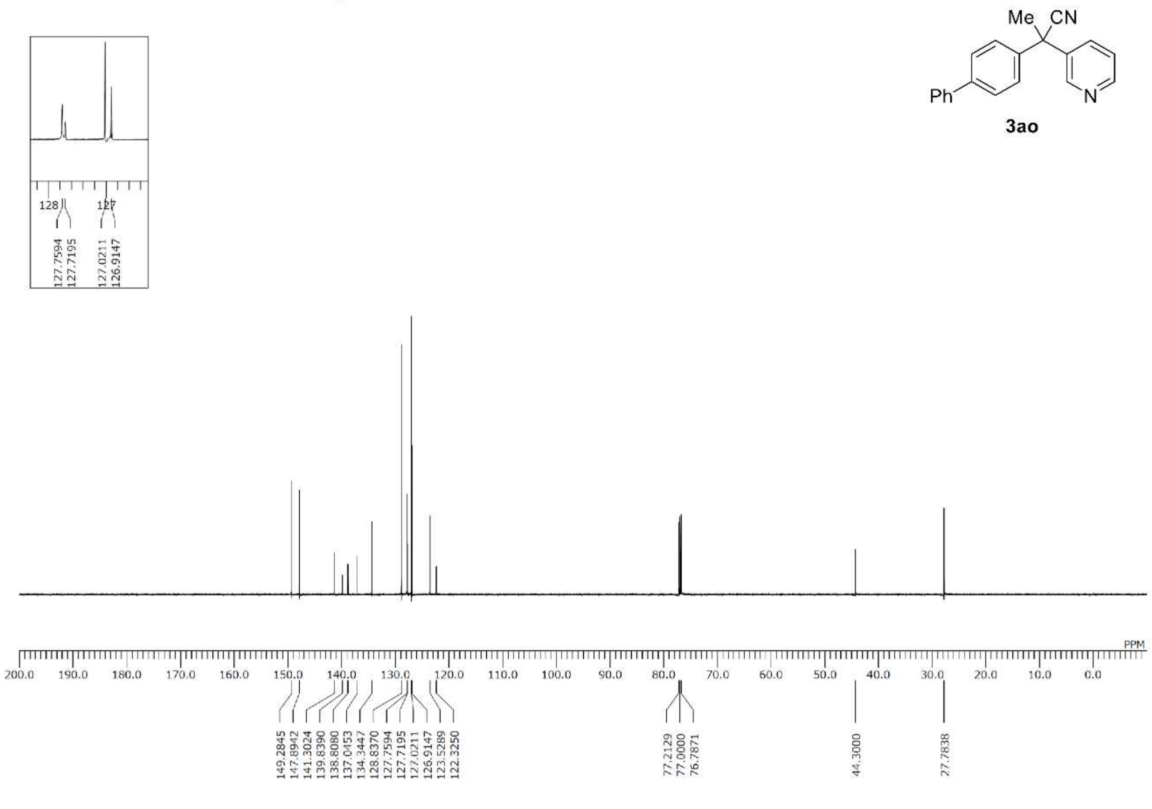

^{1}H NMR spectra of **3ap** ($CDCl_3$, 400 MHz)

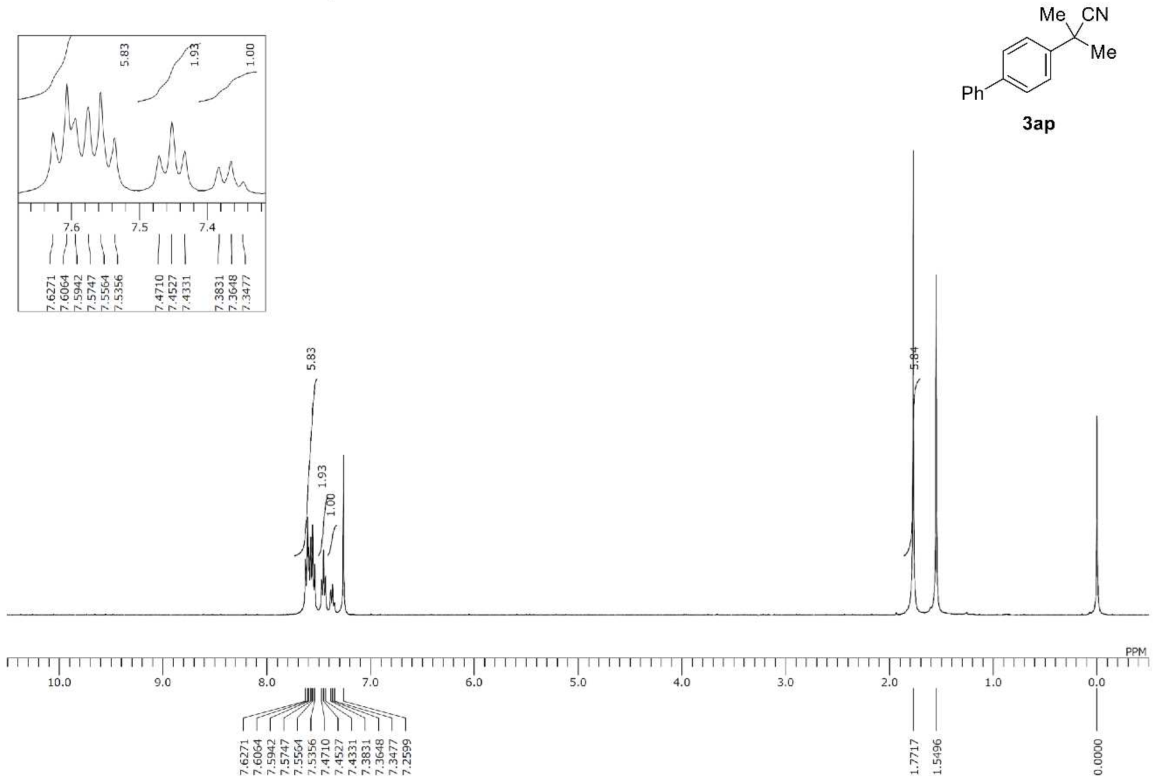

^{13}C NMR spectra of **3ap** ($CDCl_3$, 100 MHz)

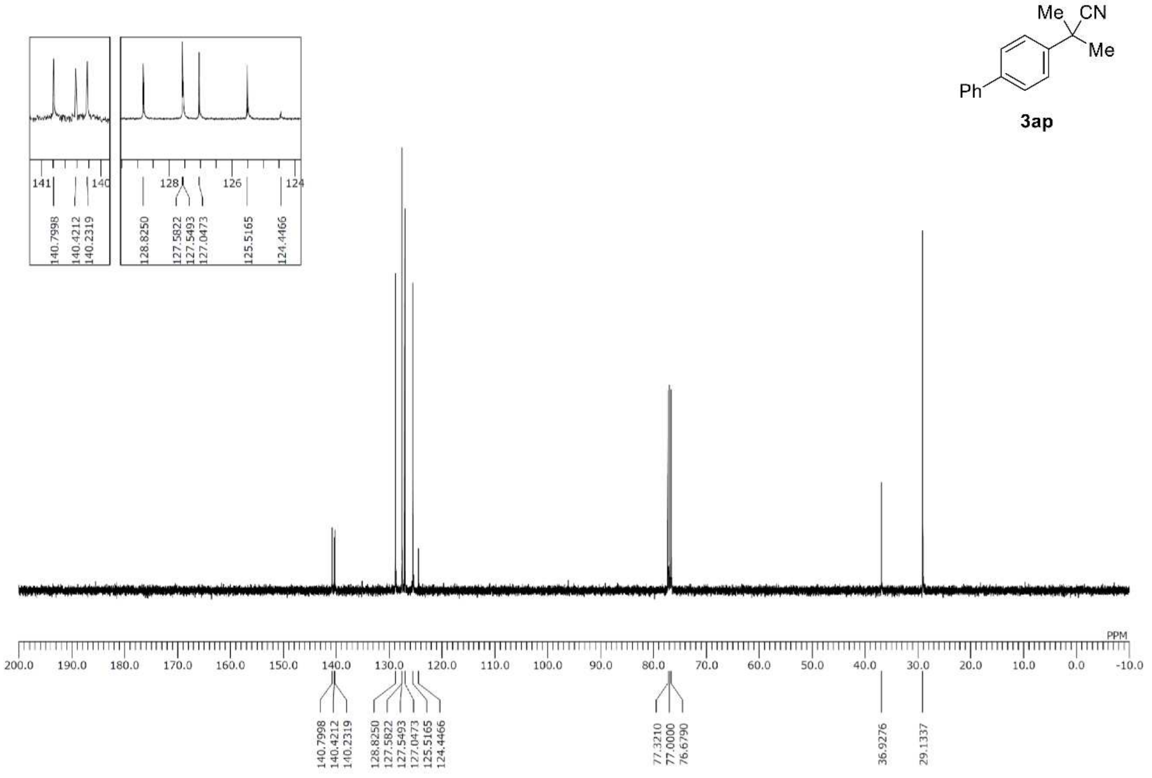

^{1}H NMR spectra of **3aq** ($CDCl_3$, 400 MHz)

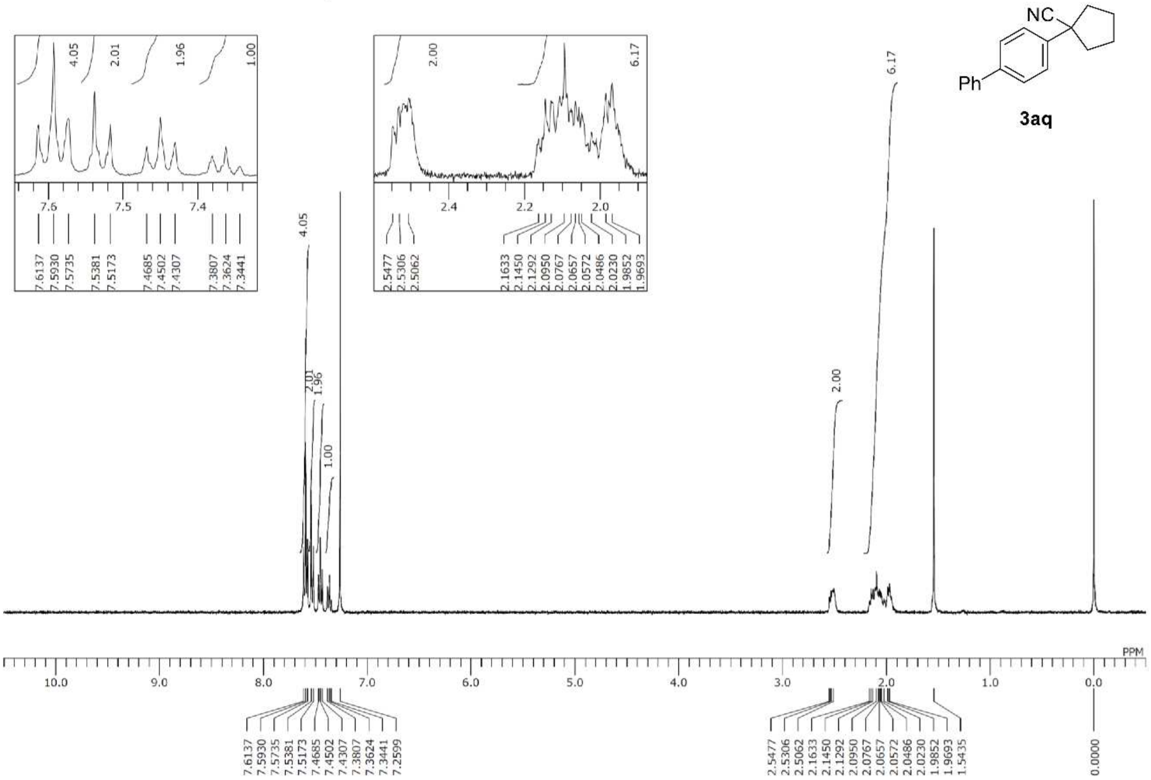

^{13}C NMR spectra of **3aq** ($CDCl_3$, 150 MHz)

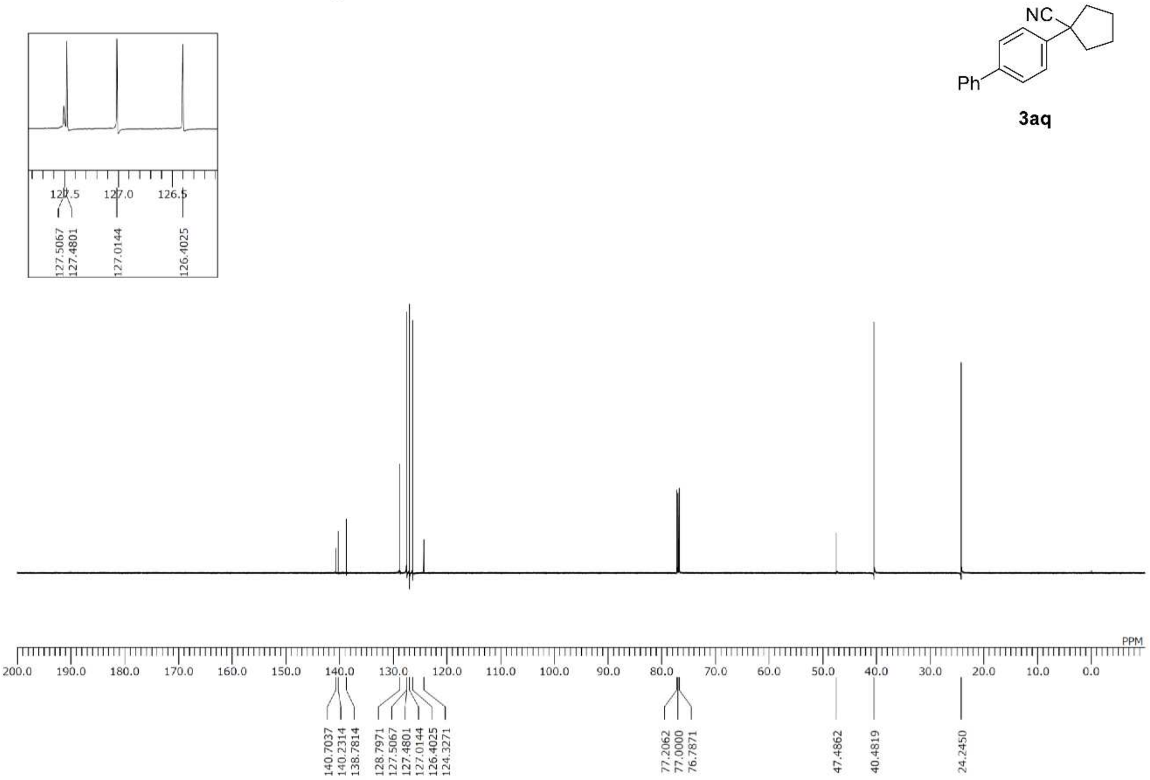

^{1}H NMR spectra of **3ar** ($CDCl_3$, 400 MHz)

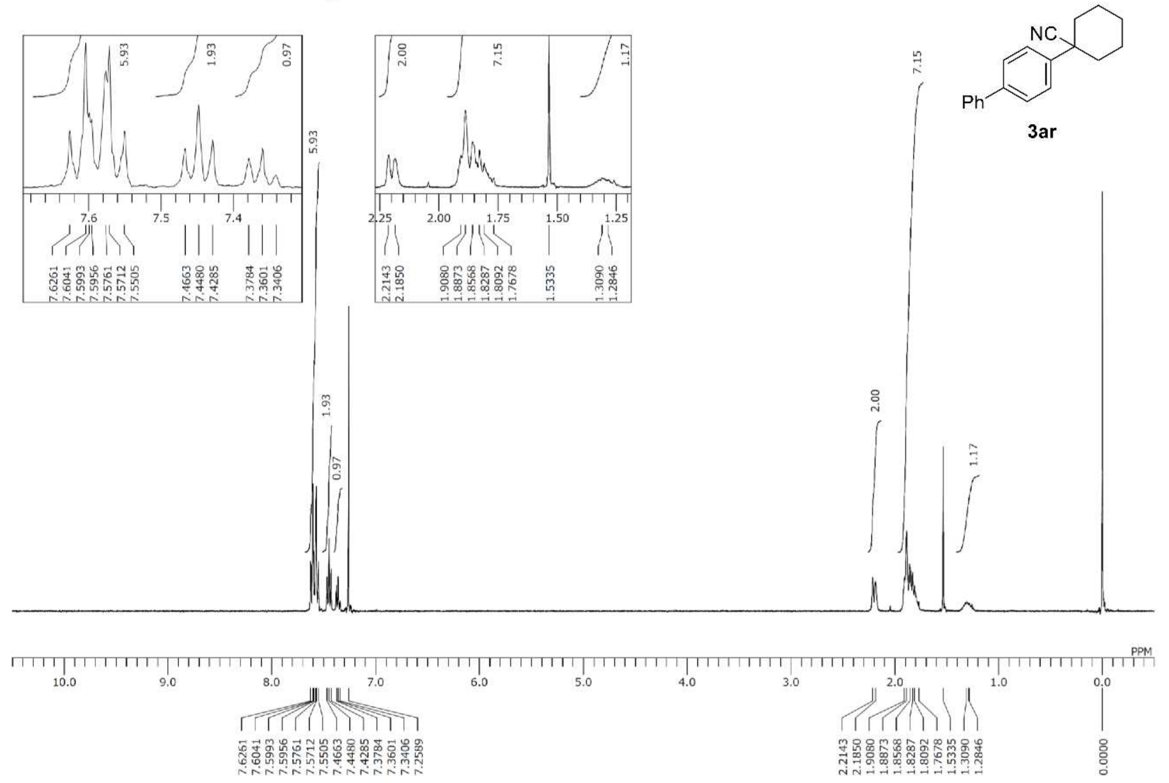

^{13}C NMR spectra of **3ar** ($CDCl_3$, 150 MHz)

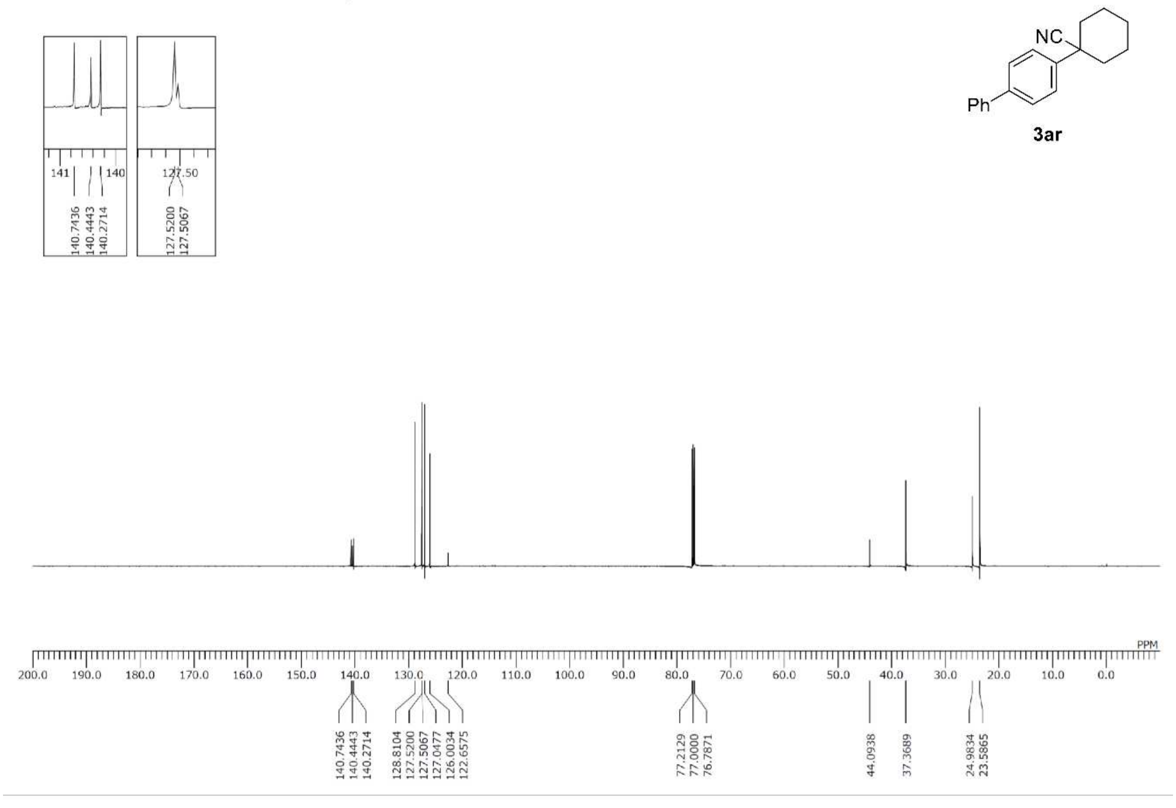

^{1}H NMR spectra of **3as** ($CDCl_3$, 400 MHz)

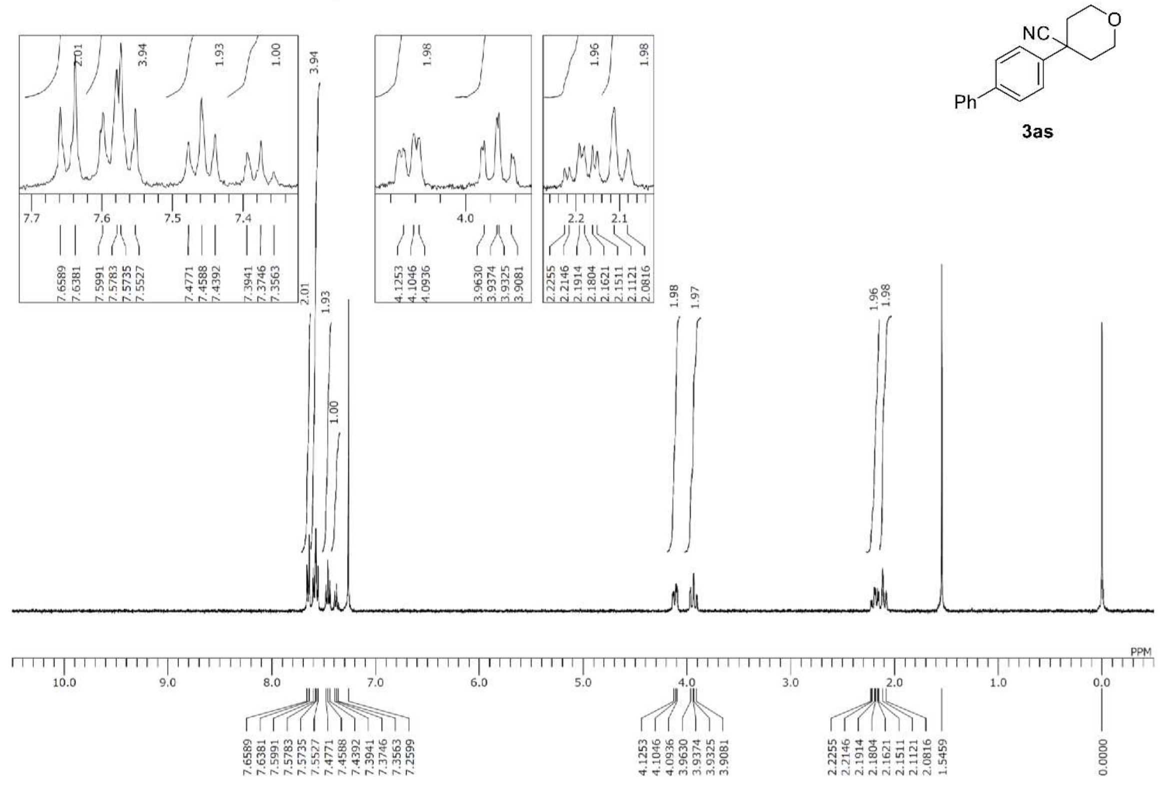

^{13}C NMR spectra of **3as** ($CDCl_3$, 150 MHz)

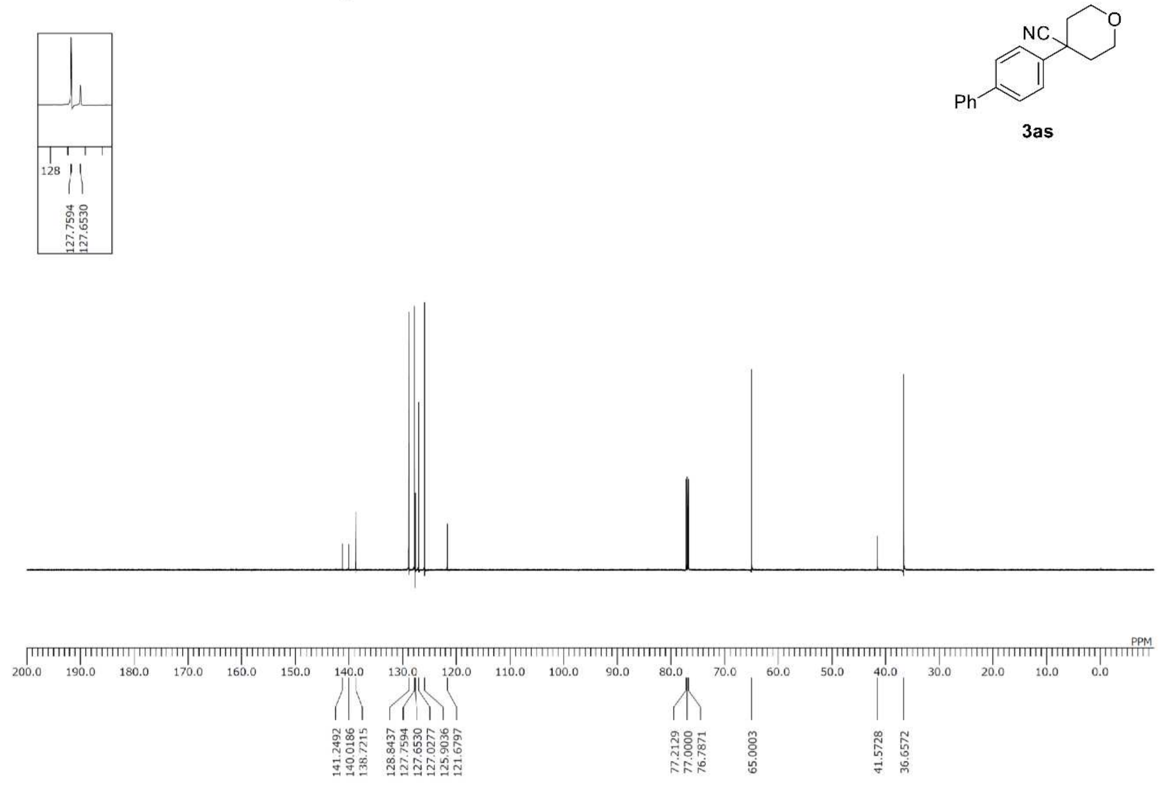

^{1}H NMR spectra of **3at** ($CDCl_3$, 400 MHz)

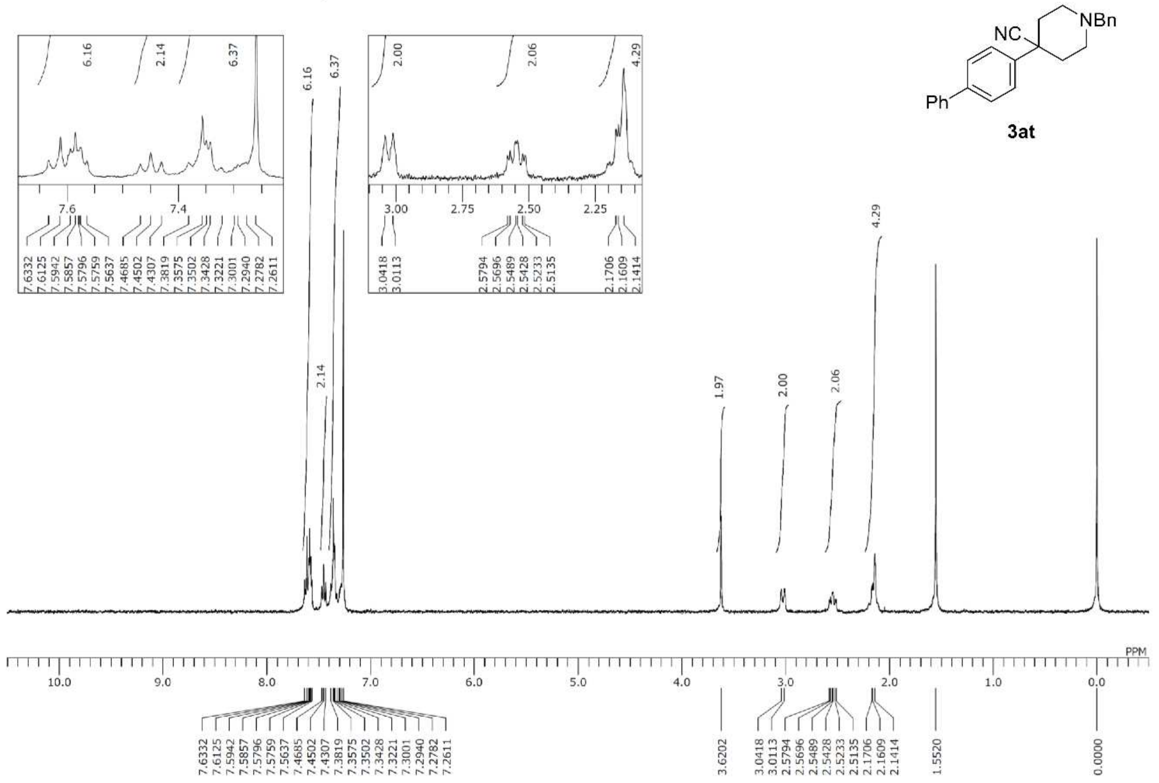

^{13}C NMR spectra of **3at** ($CDCl_3$, 150 MHz)

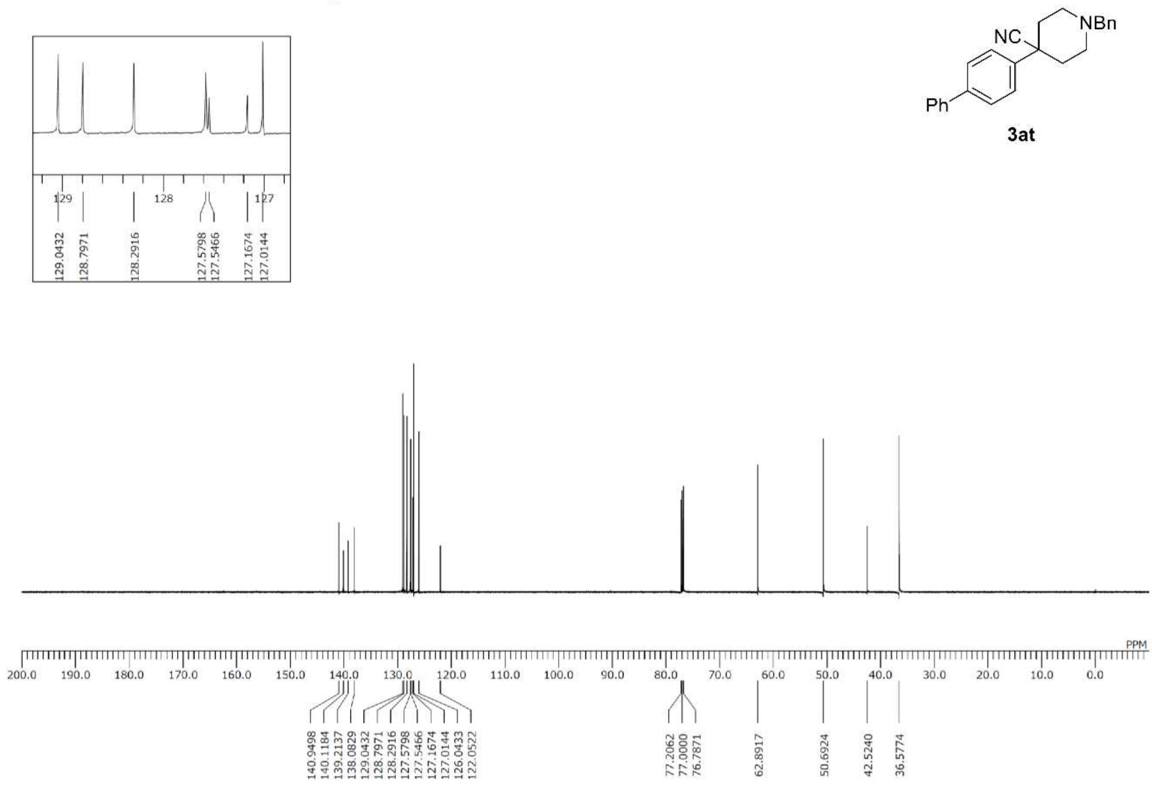

^{1}H NMR spectra of **8aa** ($CDCl_3$, 400 MHz)

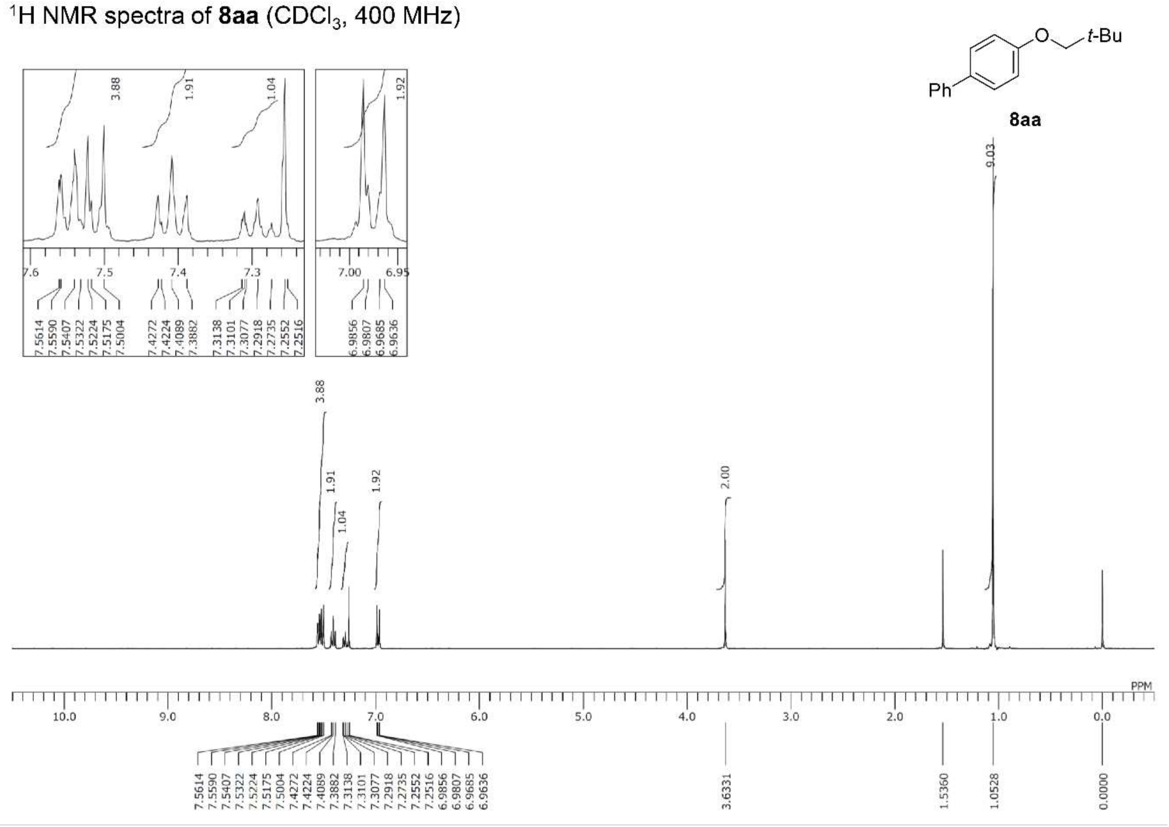

^{13}C NMR spectra of **8aa** ($CDCl_3$, 150 MHz)

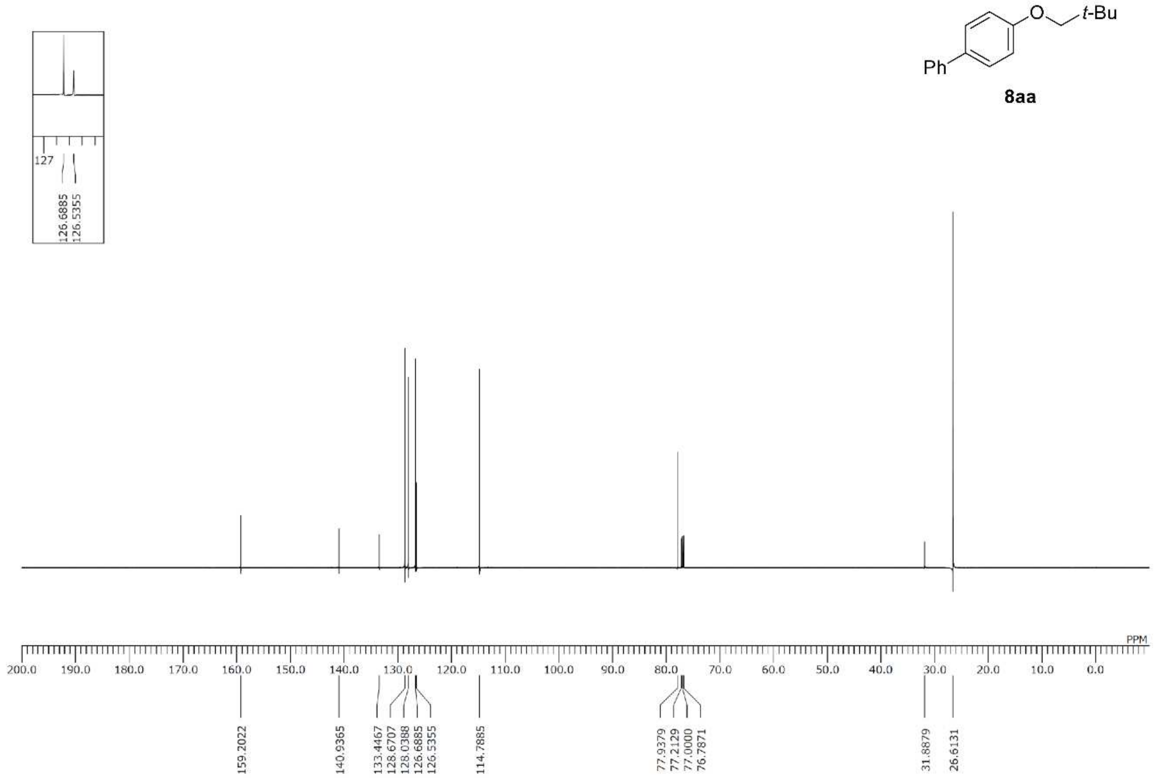

^{1}H NMR spectra of **8ab** ($CDCl_3$, 400 MHz)

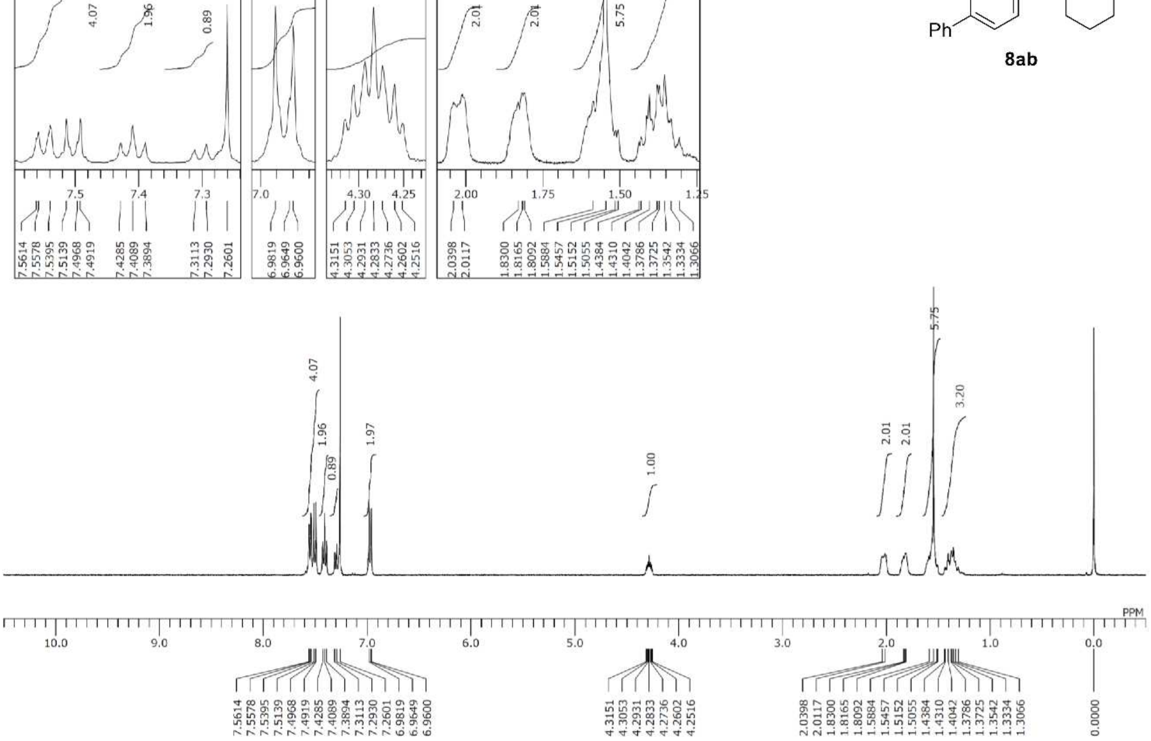

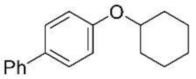

8ab

^{13}C NMR spectra of **8ab** ($CDCl_3$, 150 MHz)

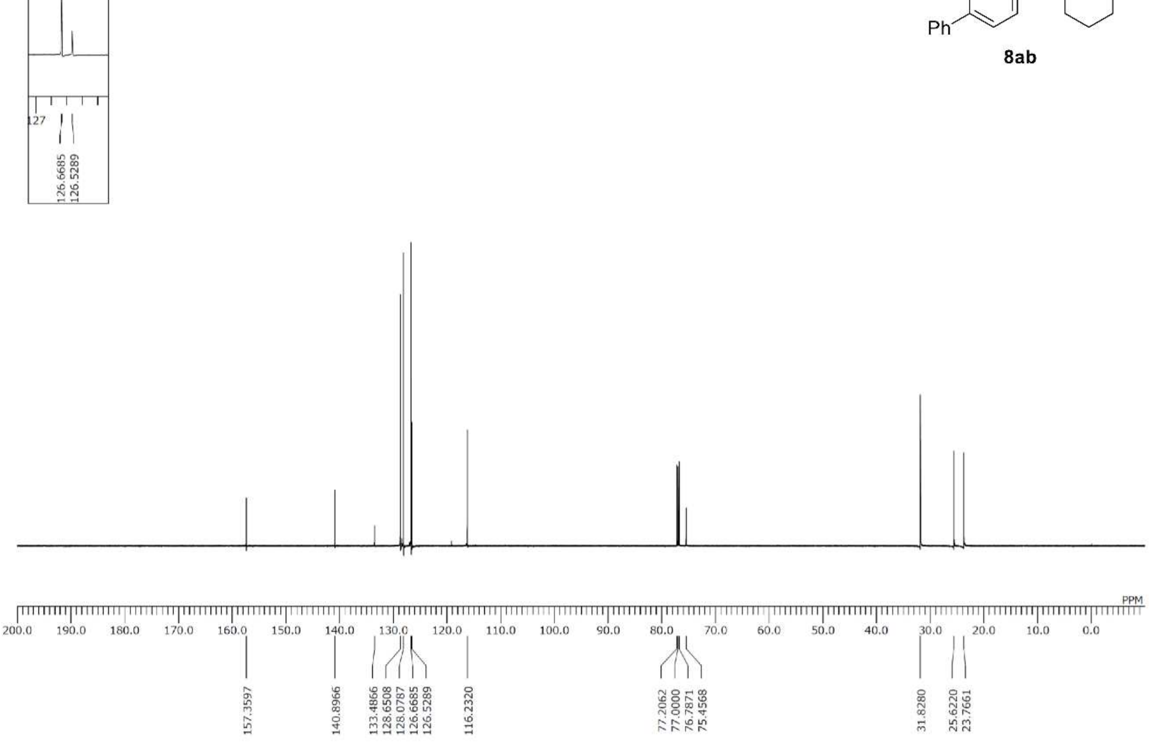

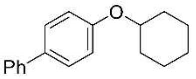

8ab

^{1}H NMR spectra of **8ac** ($CDCl_3$, 400 MHz)

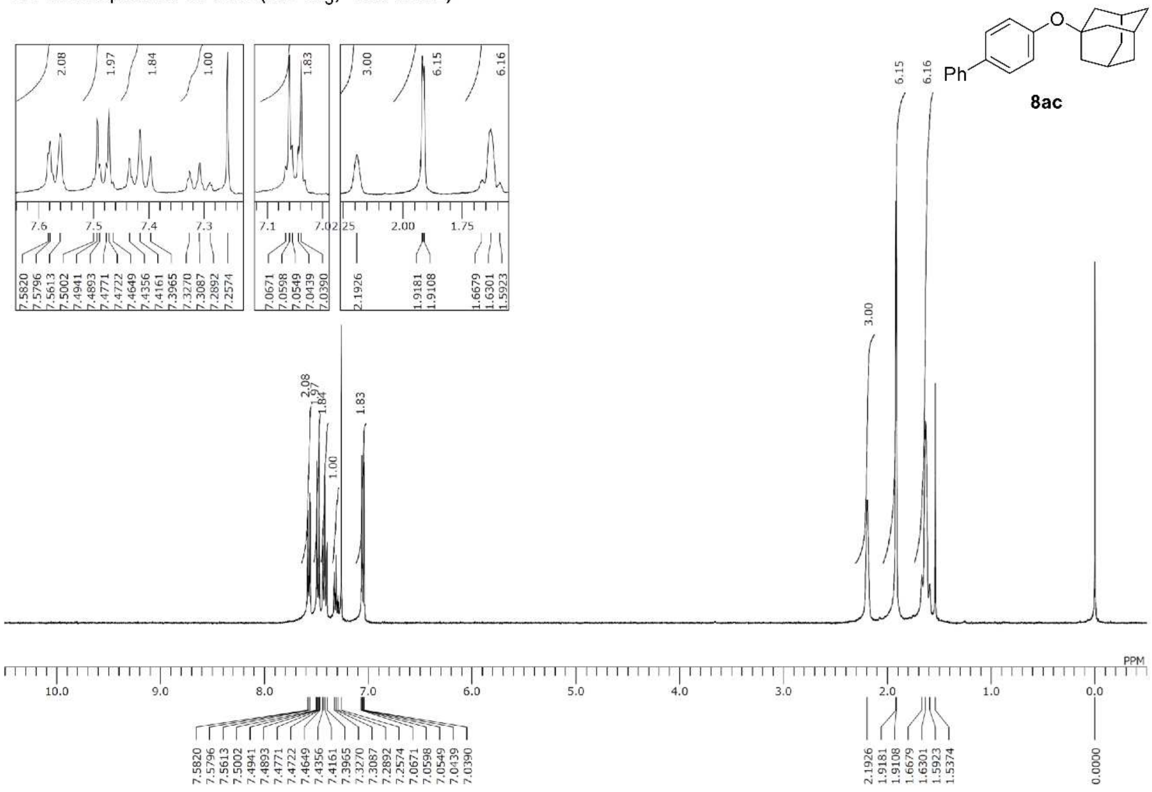

^{13}C NMR spectra of **8ac** ($CDCl_3$, 100 MHz)

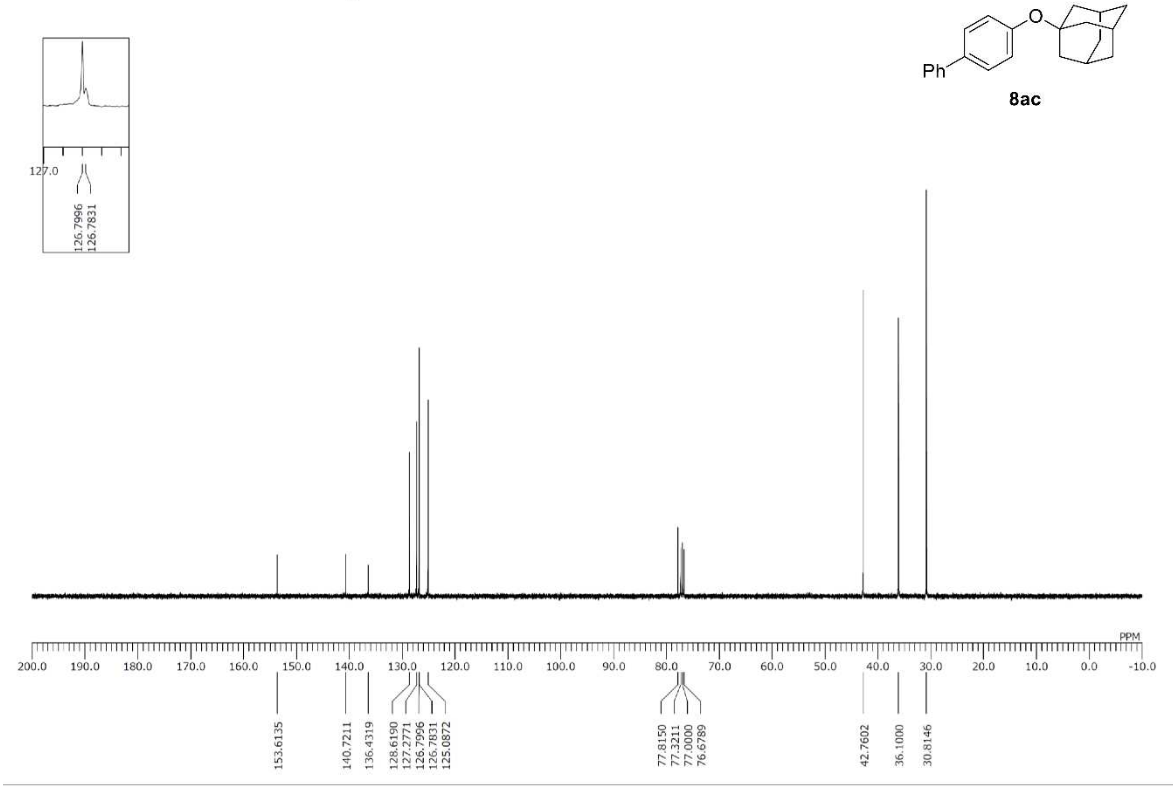

^{1}H NMR spectra of **8ad** ($CDCl_3$, 400 MHz)

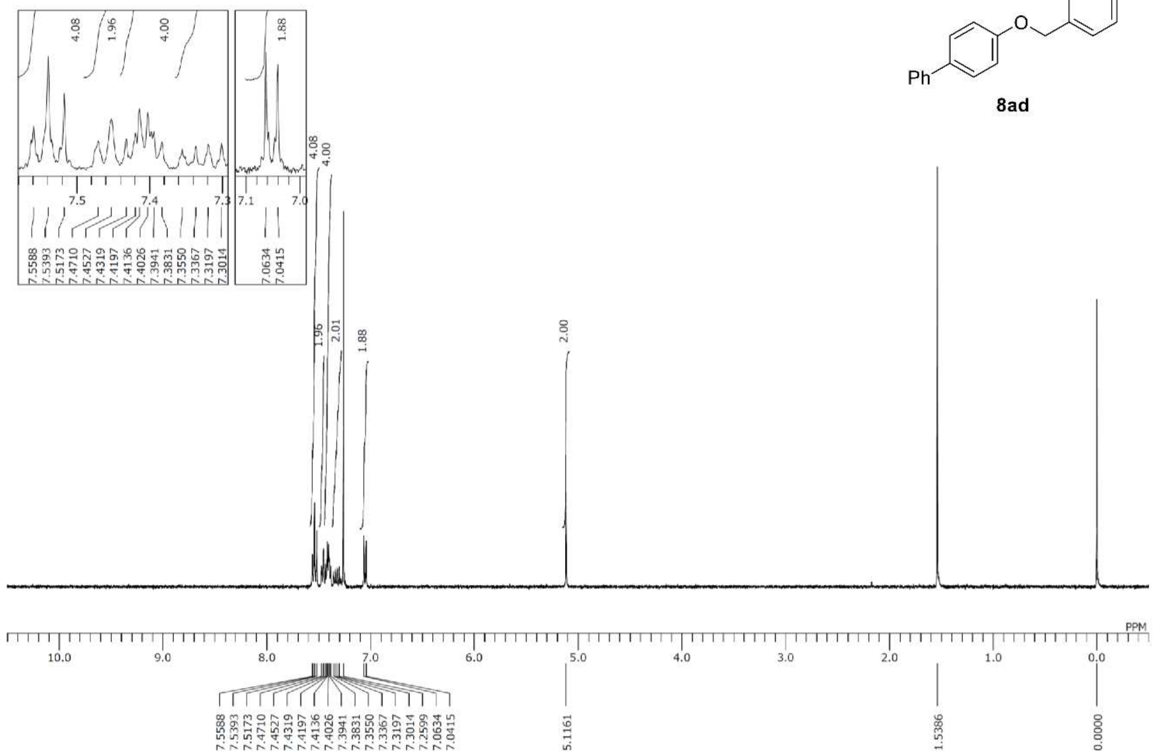

^{13}C NMR spectra of **8ad** ($CDCl_3$, 150 MHz)

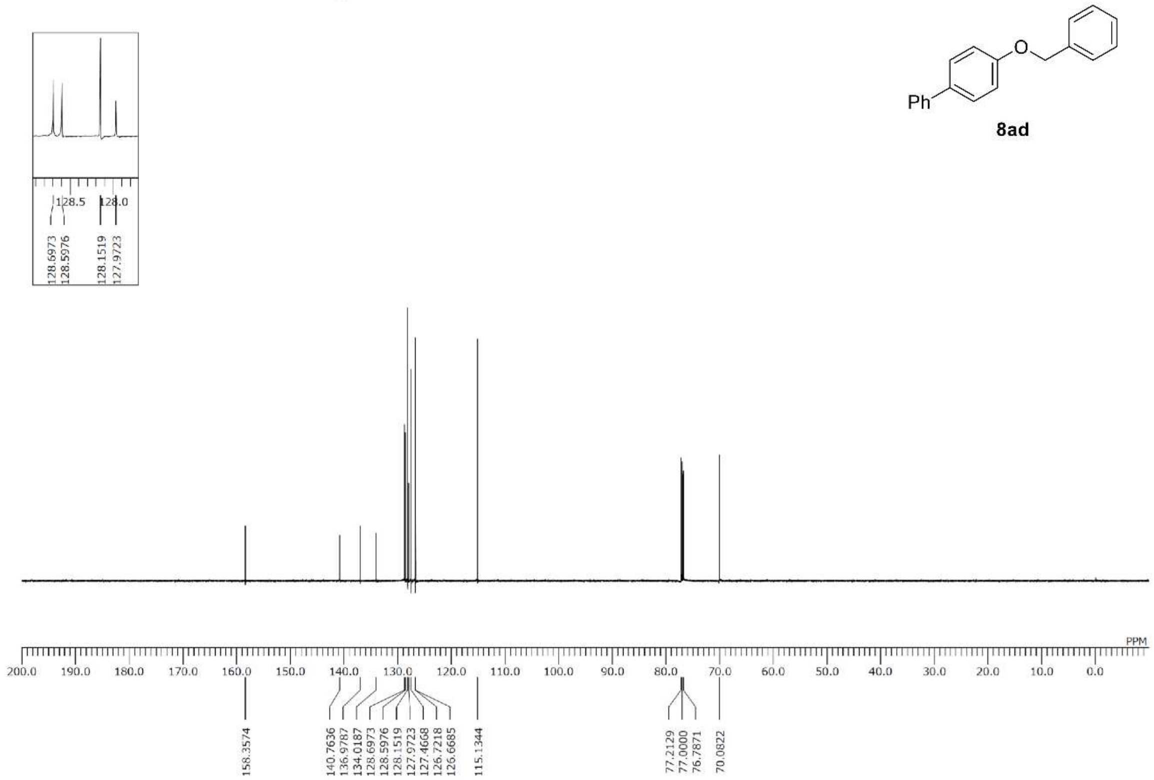

^{1}H NMR spectra of **8ae** ($CDCl_3$, 400 MHz)

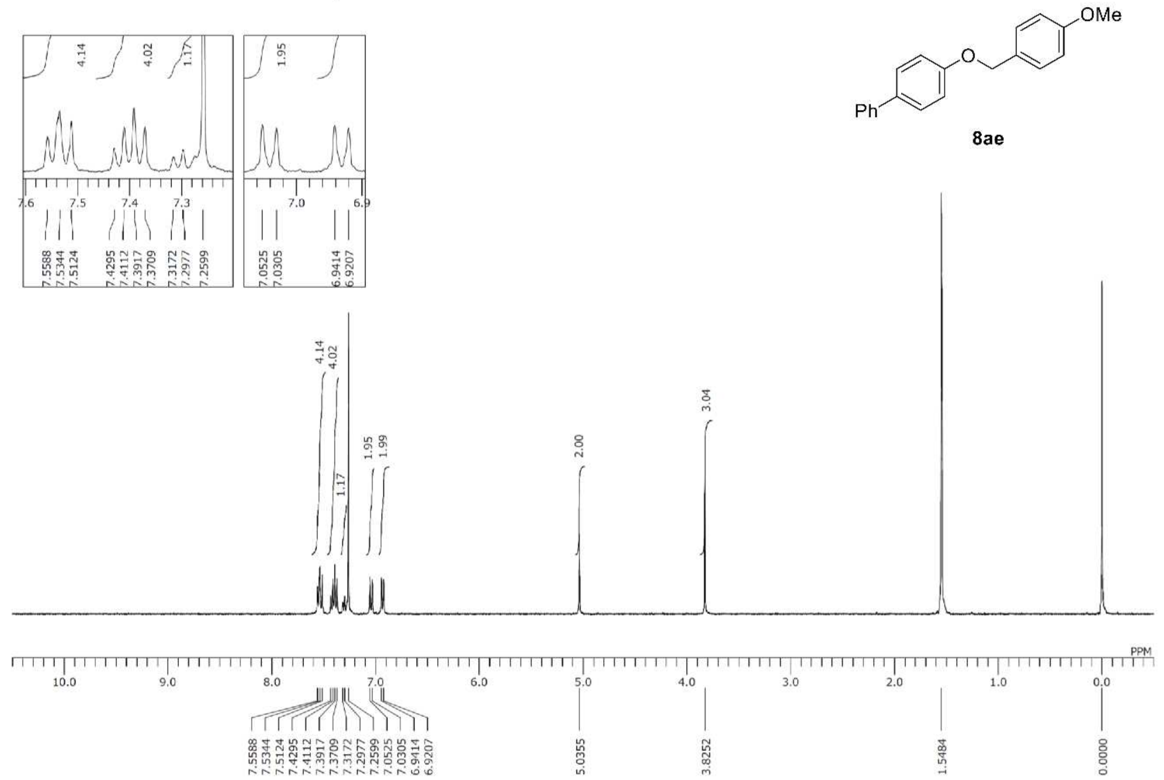

^{13}C NMR spectra of **8ae** ($CDCl_3$, 150 MHz)

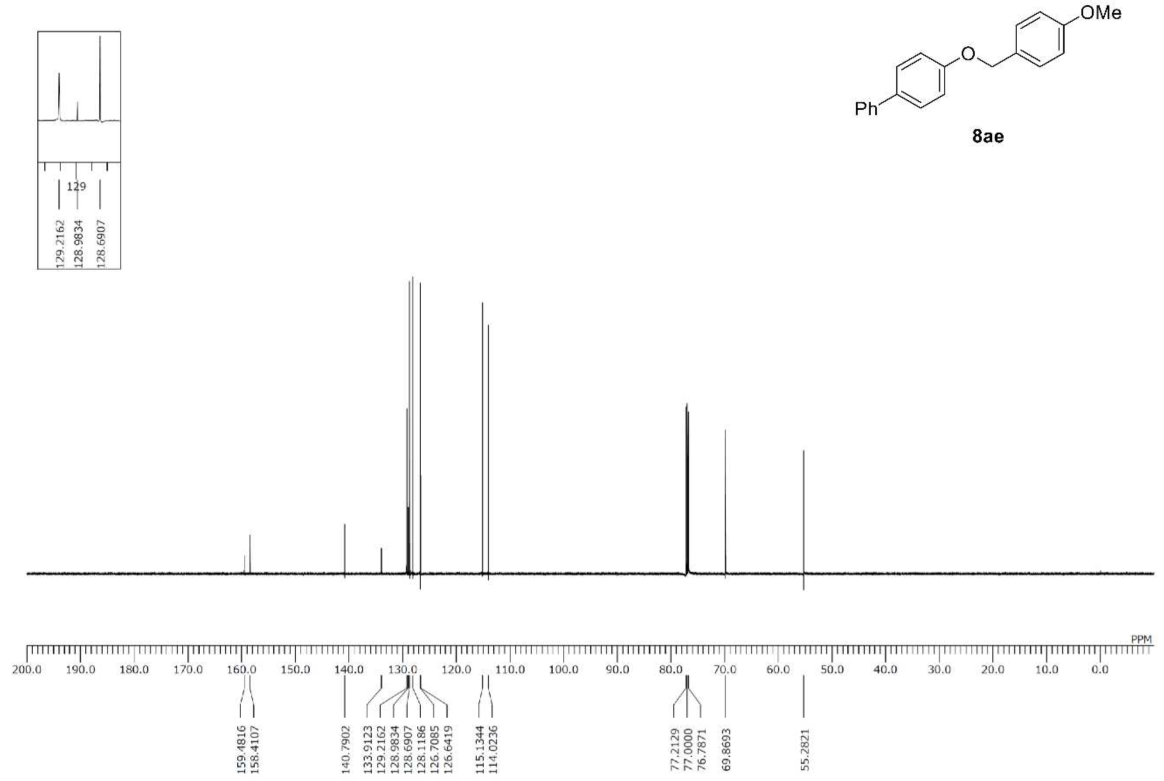

^{1}H NMR spectra of **8af** ($CDCl_3$, 400 MHz)

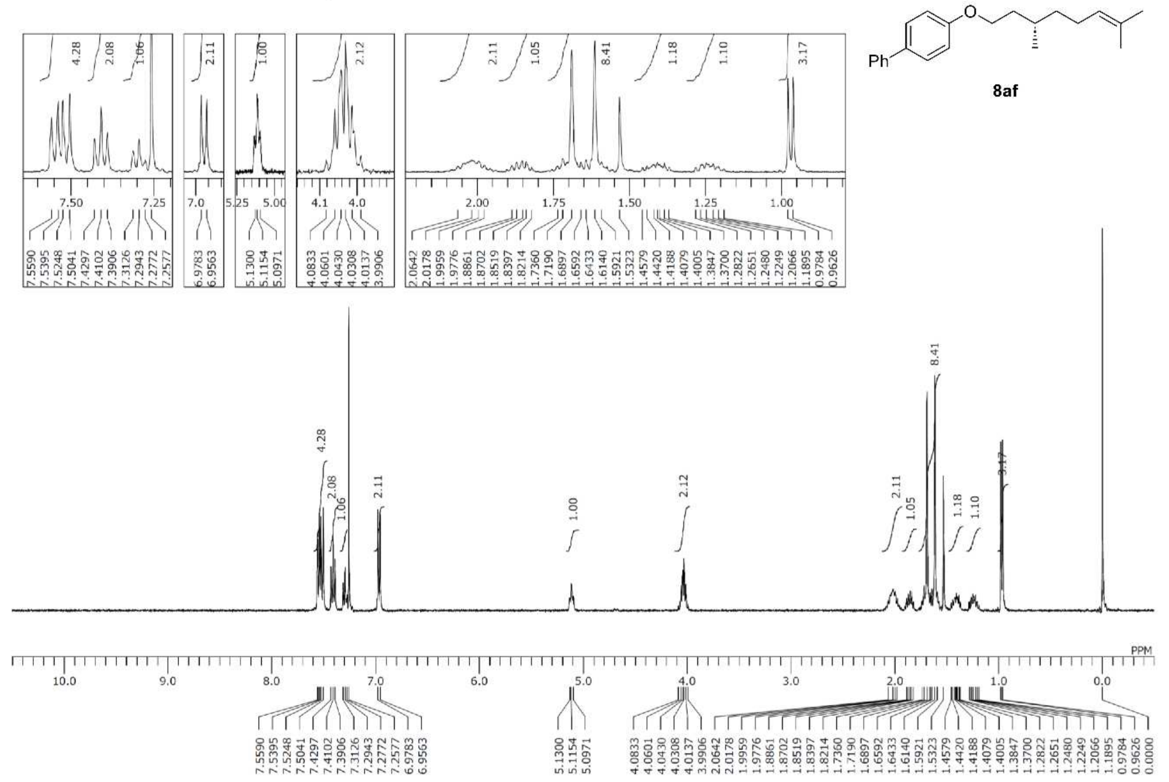

^{13}C NMR spectra of **8af** ($CDCl_3$, 150 MHz)

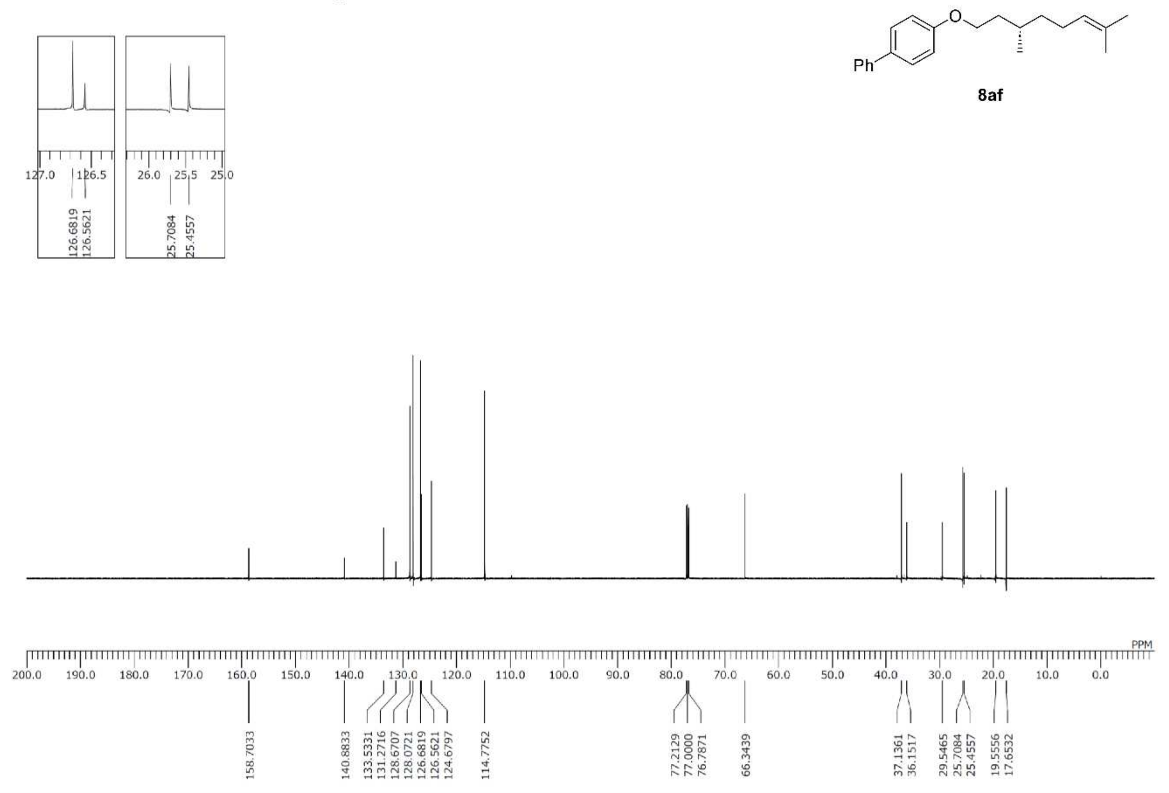

^{1}H NMR spectra of **8ag** ($CDCl_3$, 400 MHz)

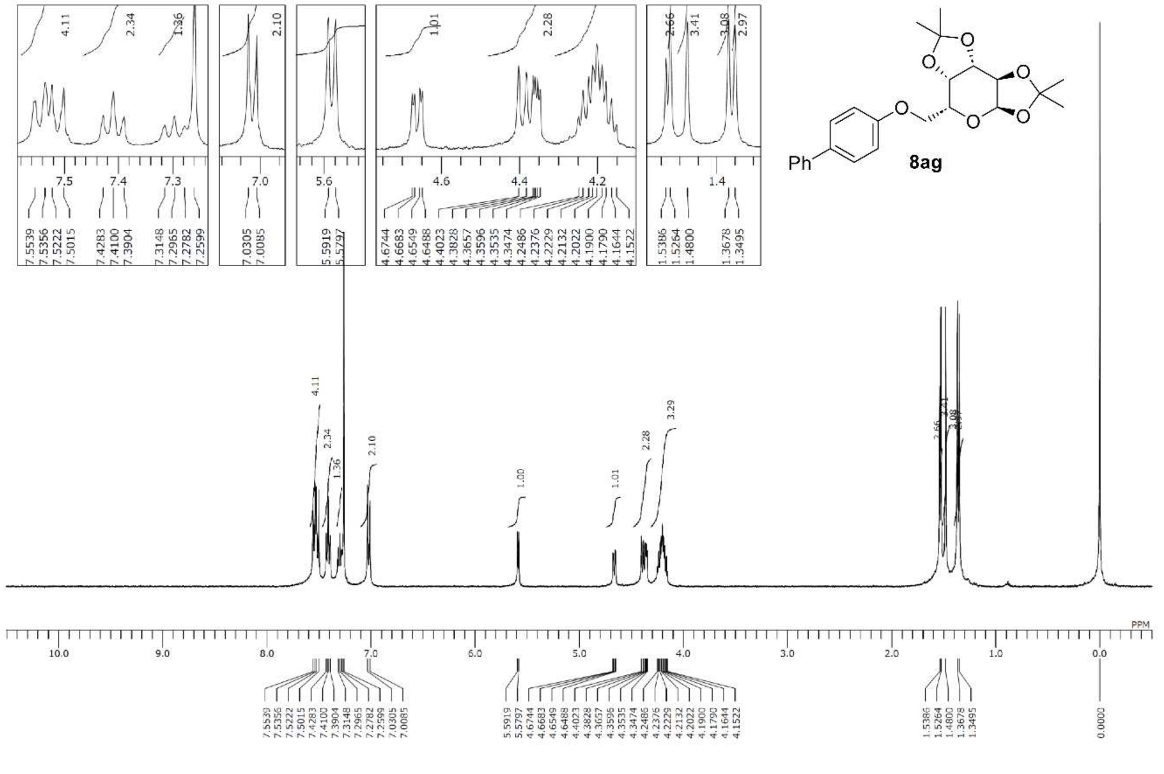

^{13}C NMR spectra of **8ag** ($CDCl_3$, 150 MHz, 40 °C)

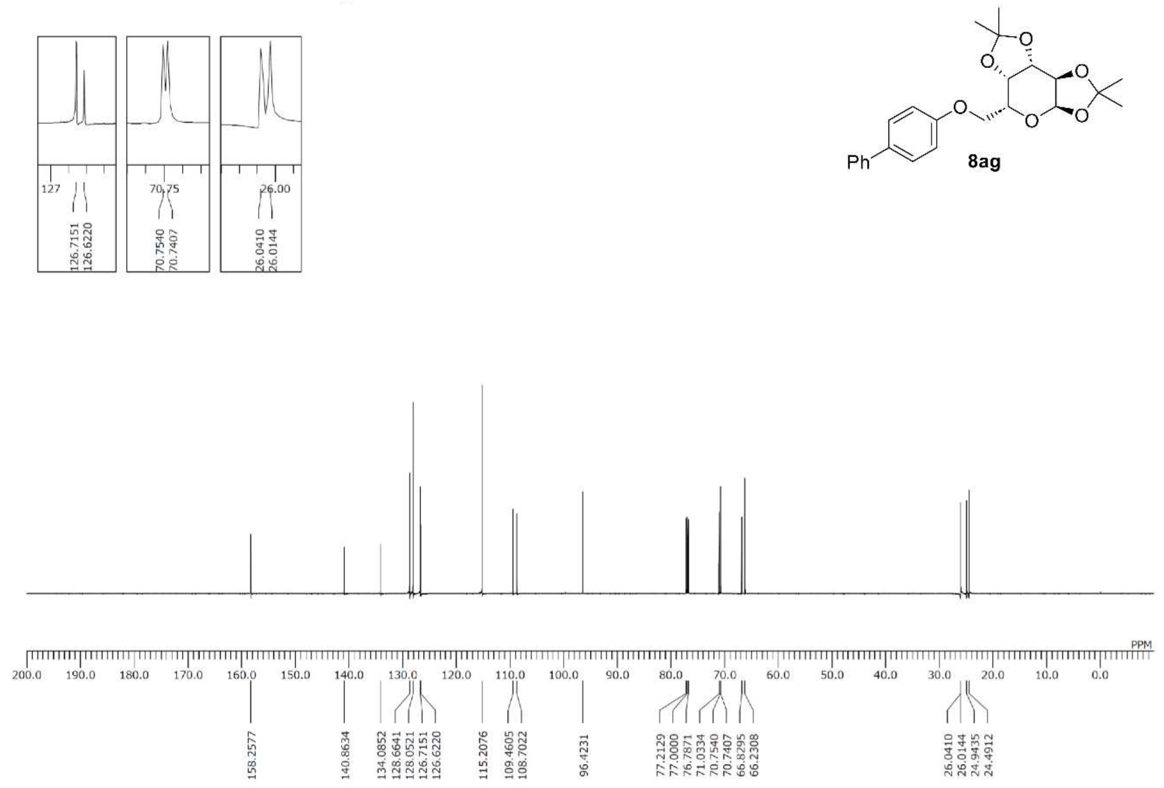

^{1}H NMR spectra of **9aa** ($CDCl_3$, 400 MHz)

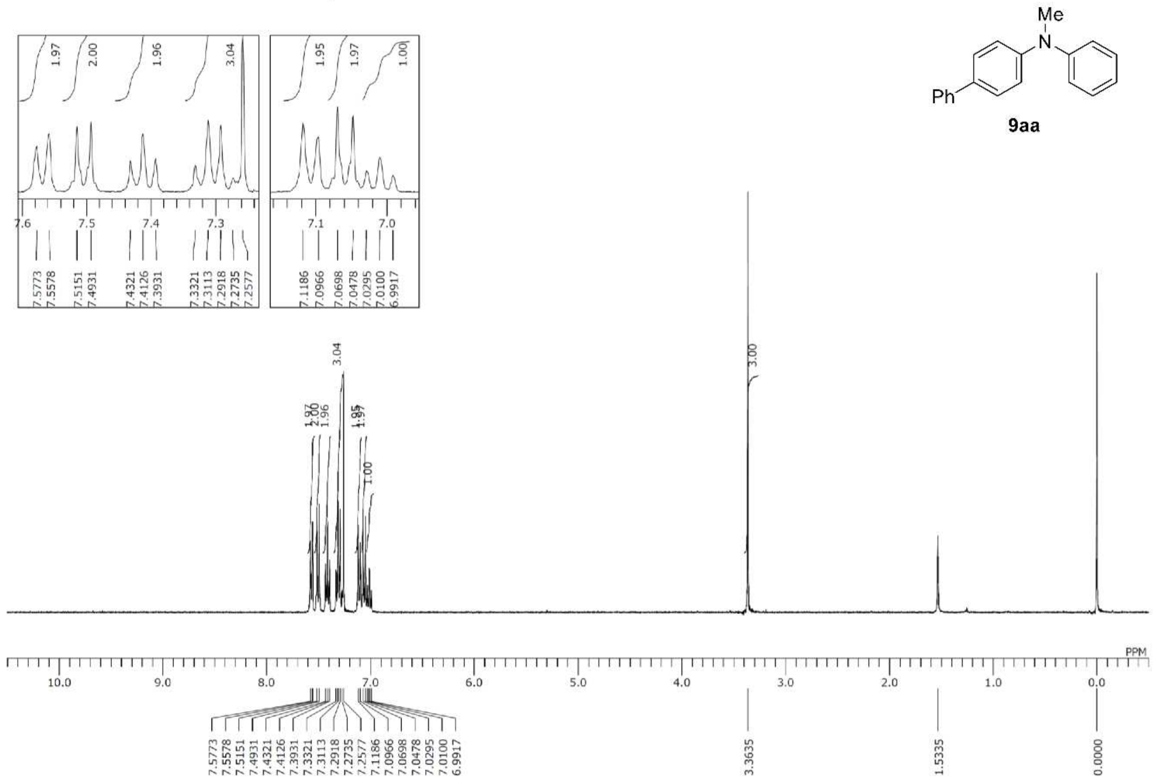

^{13}C NMR spectra of **9aa** (DMSO-d_6, 150 MHz, 40 °C)

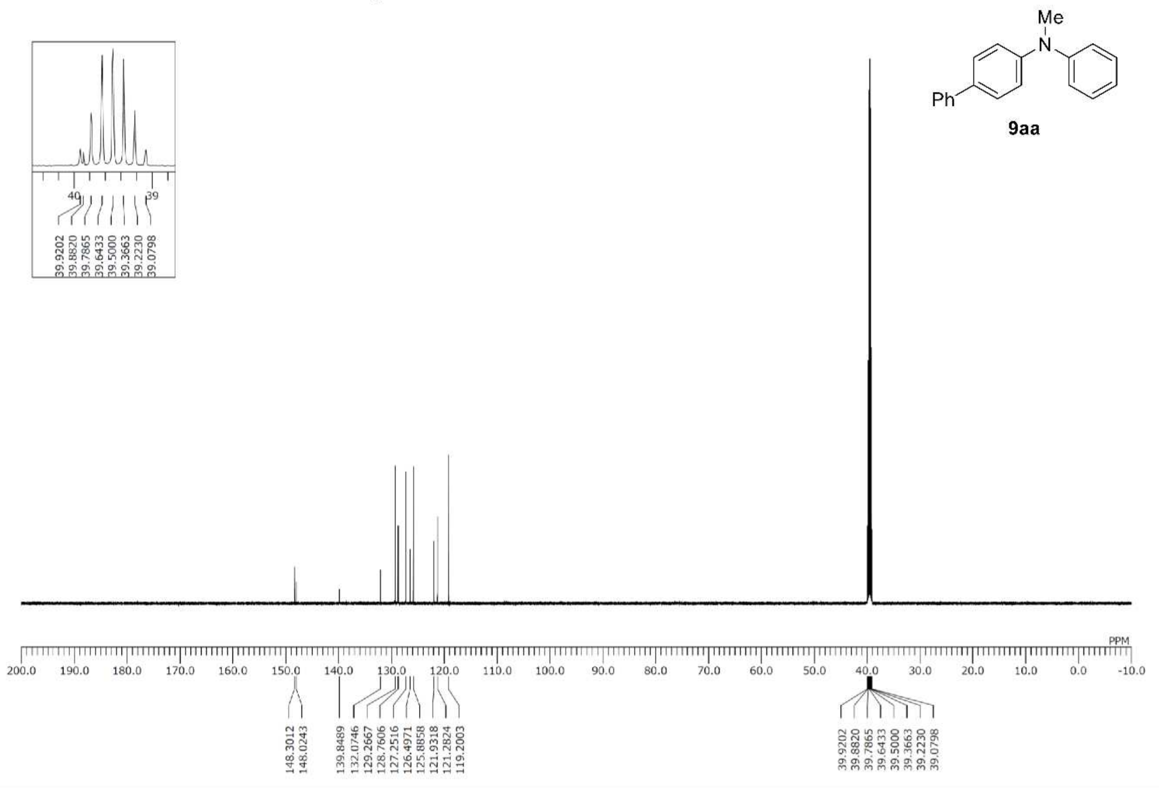

^{1}H NMR spectra of **9ab** ($CDCl_3$, 400 MHz)

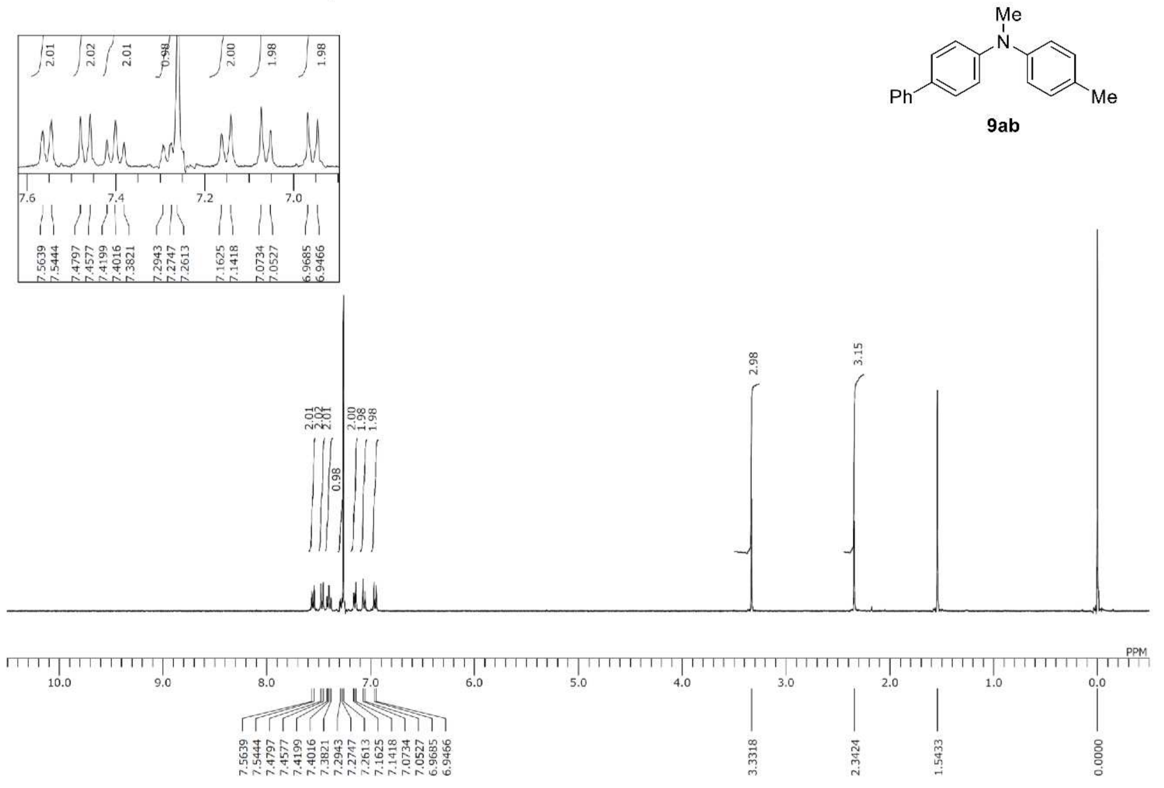

^{13}C NMR spectra of **9ab** ($CDCl_3$, 150 MHz)

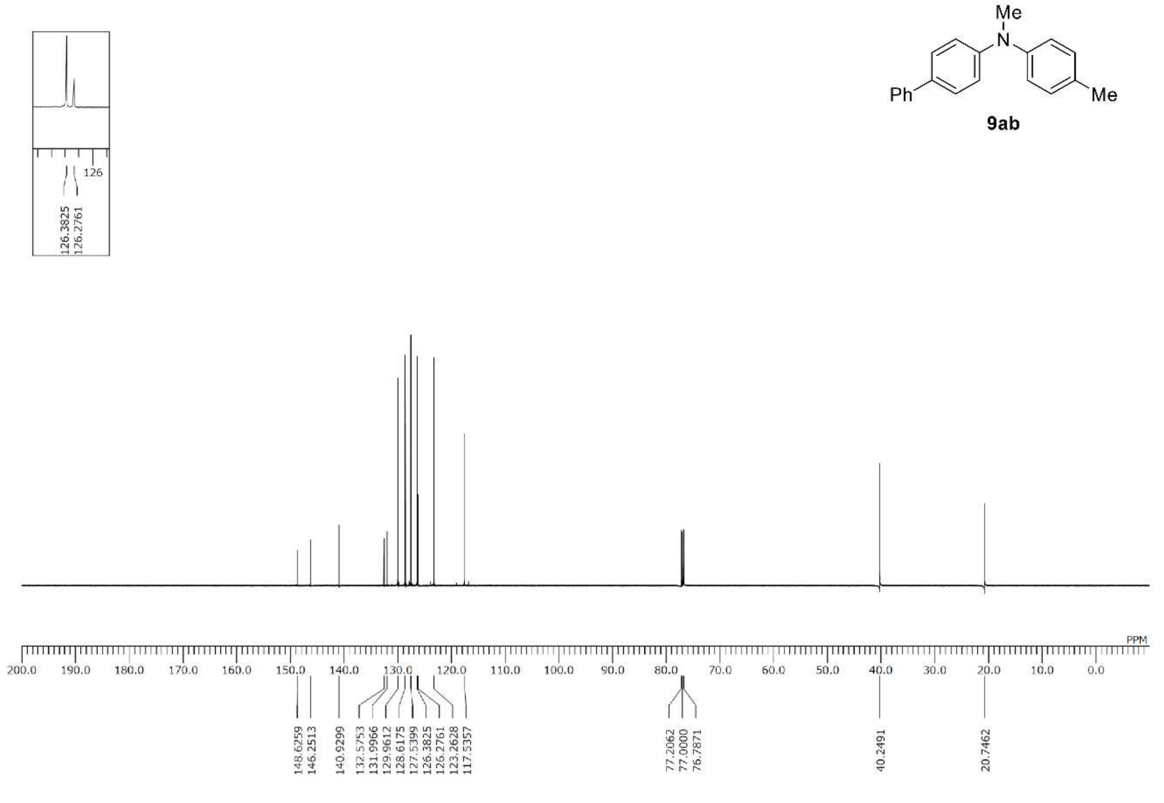

^{1}H NMR spectra of **9ac** ($CDCl_3$, 400 MHz)

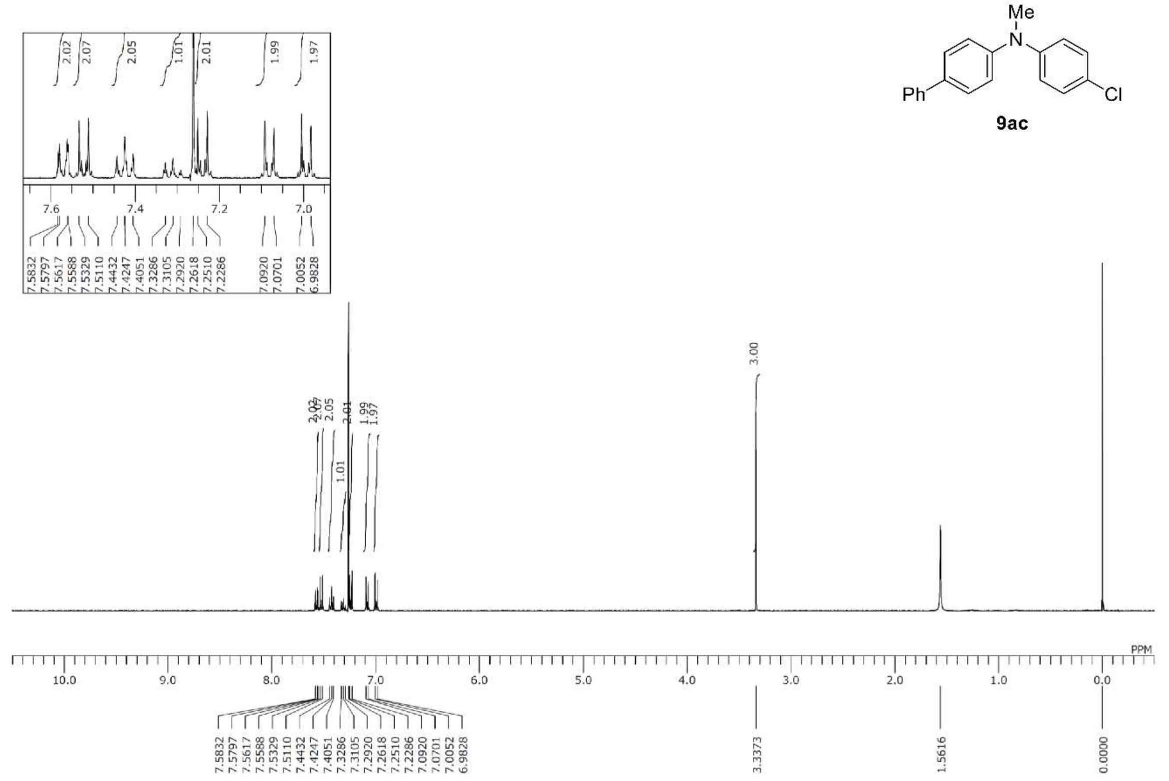

^{13}C NMR spectra of **9ac** ($CDCl_3$, 100 MHz)

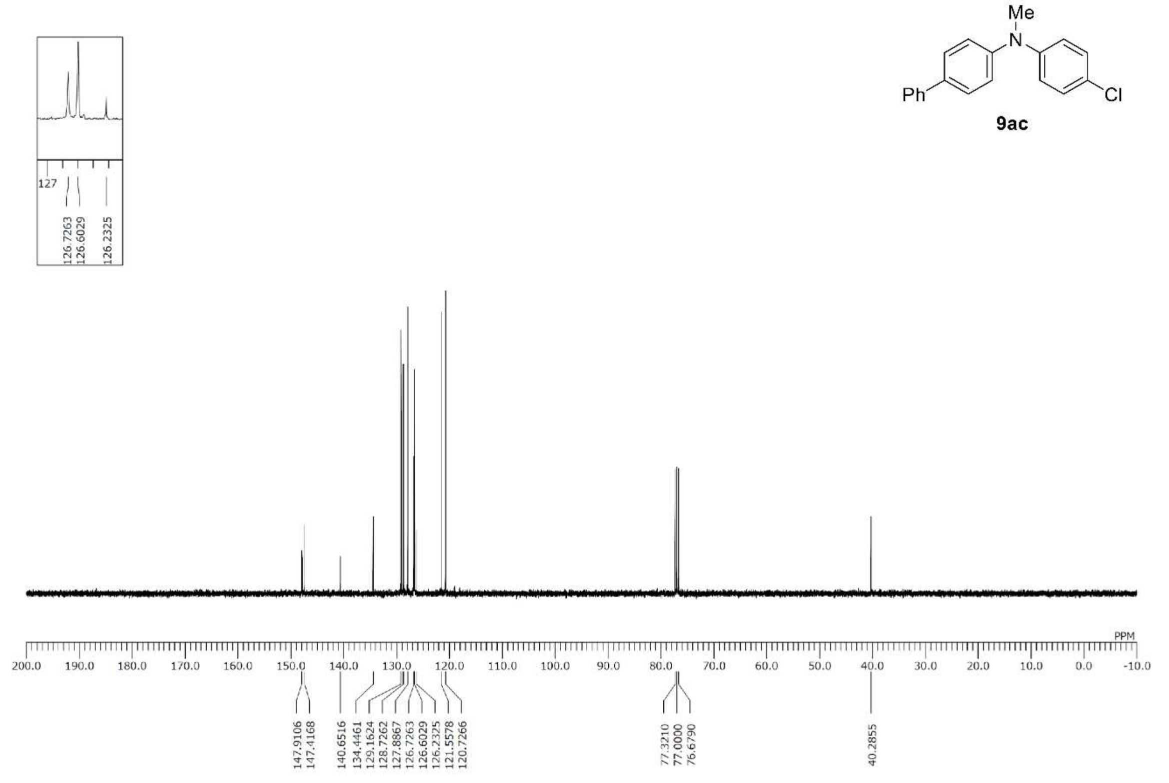

^{1}H NMR spectra of **9ad** ($CDCl_3$, 400 MHz)

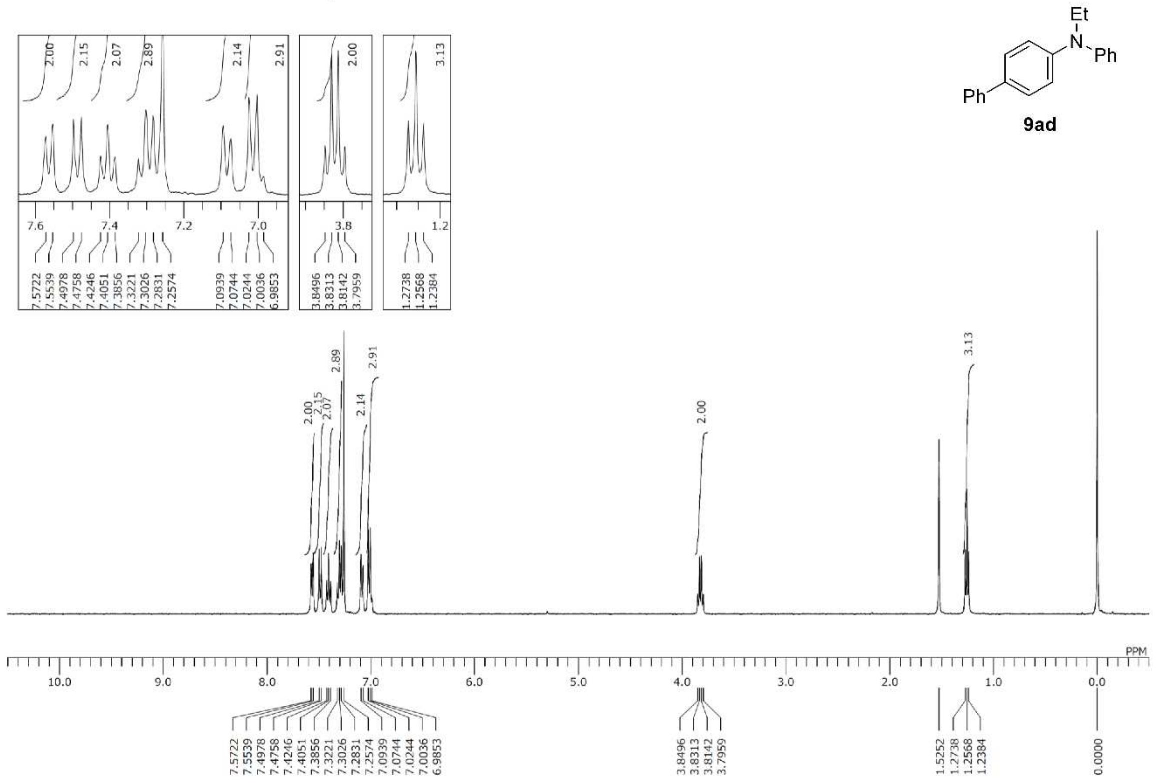

^{13}C NMR spectra of **9ad** ($CDCl_3$, 150 MHz)

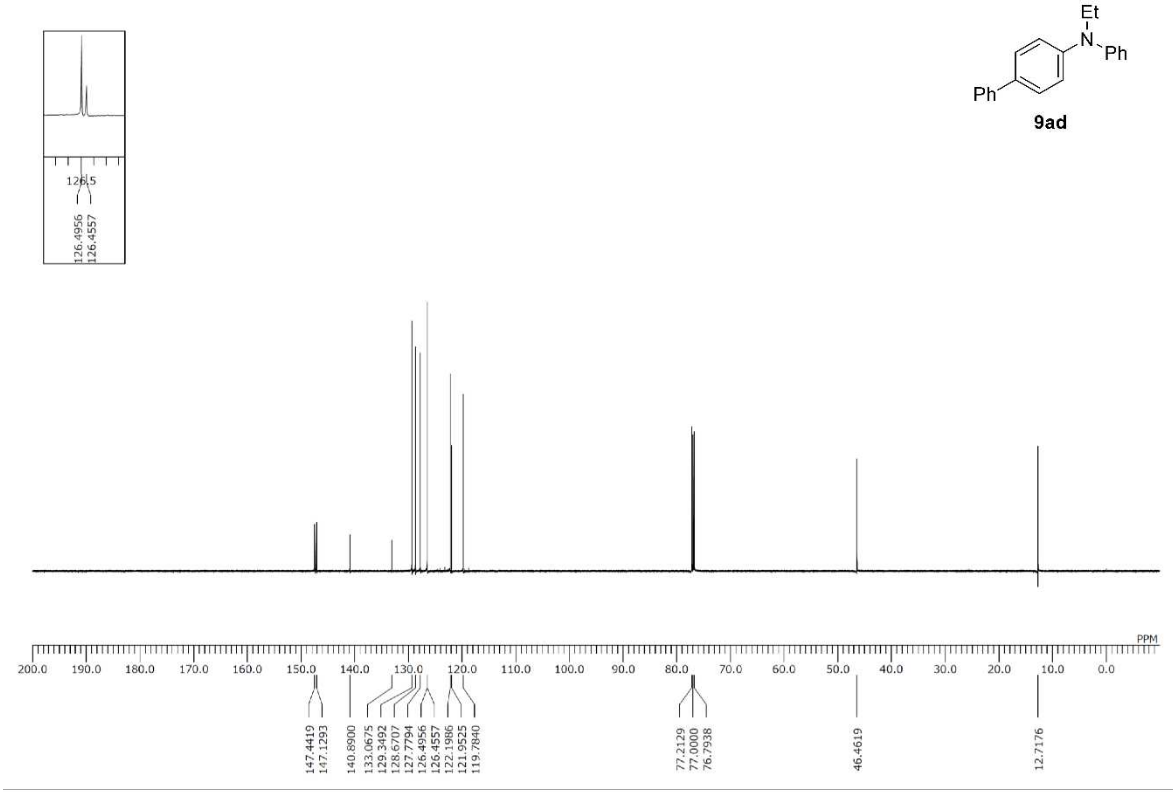

^{1}H NMR spectra of **9ae** ($CDCl_3$, 400 MHz)

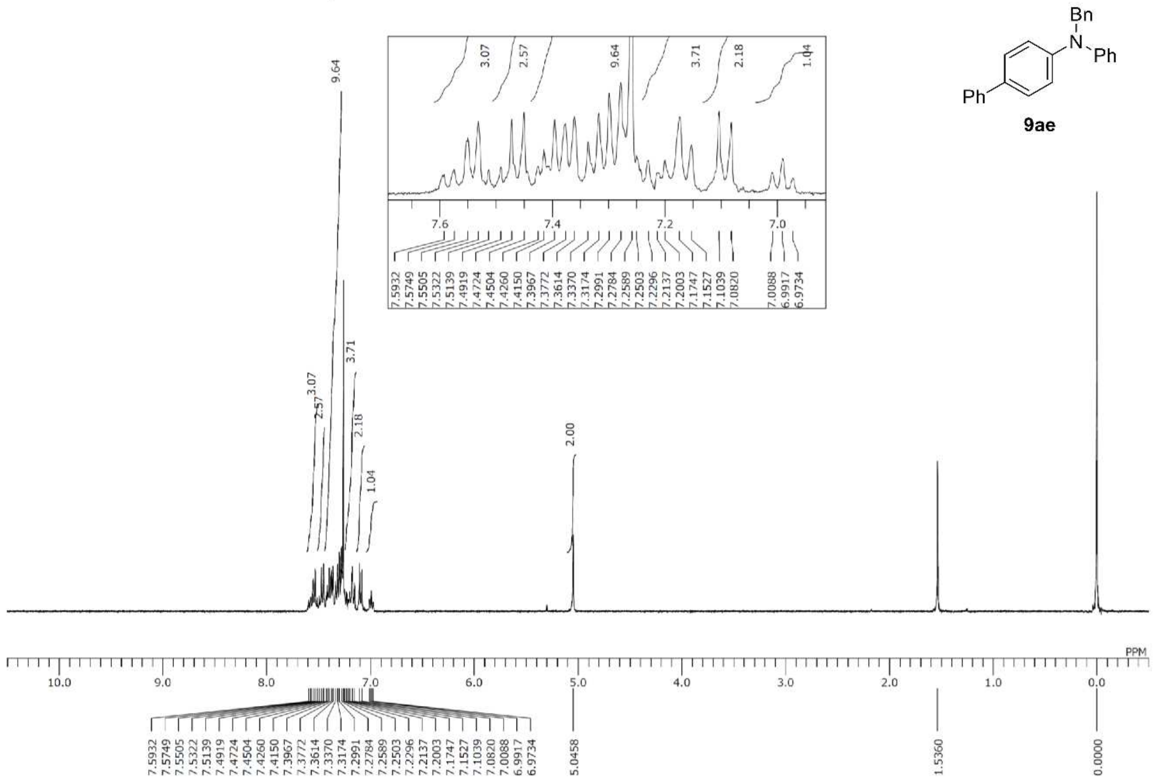

^{13}C NMR spectra of **9ae** ($CDCl_3$, 150 MHz)

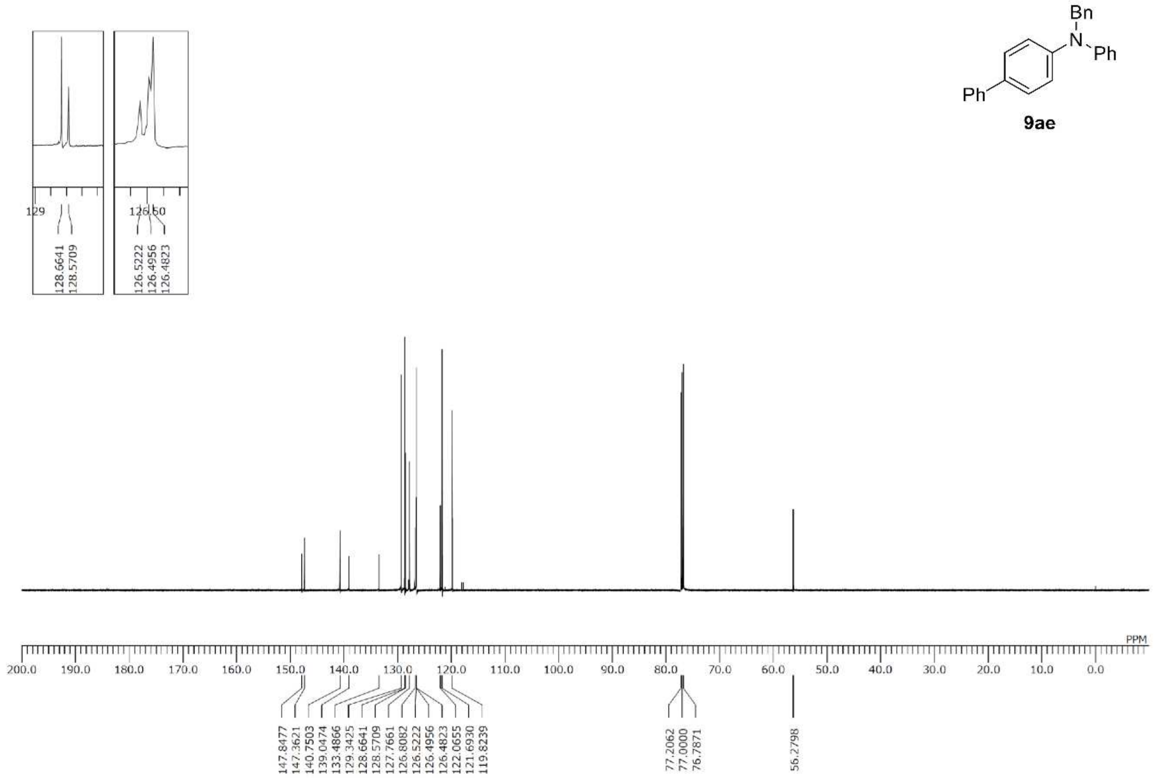

^{1}H NMR spectra of **9af** ($CDCl_3$, 400 MHz)

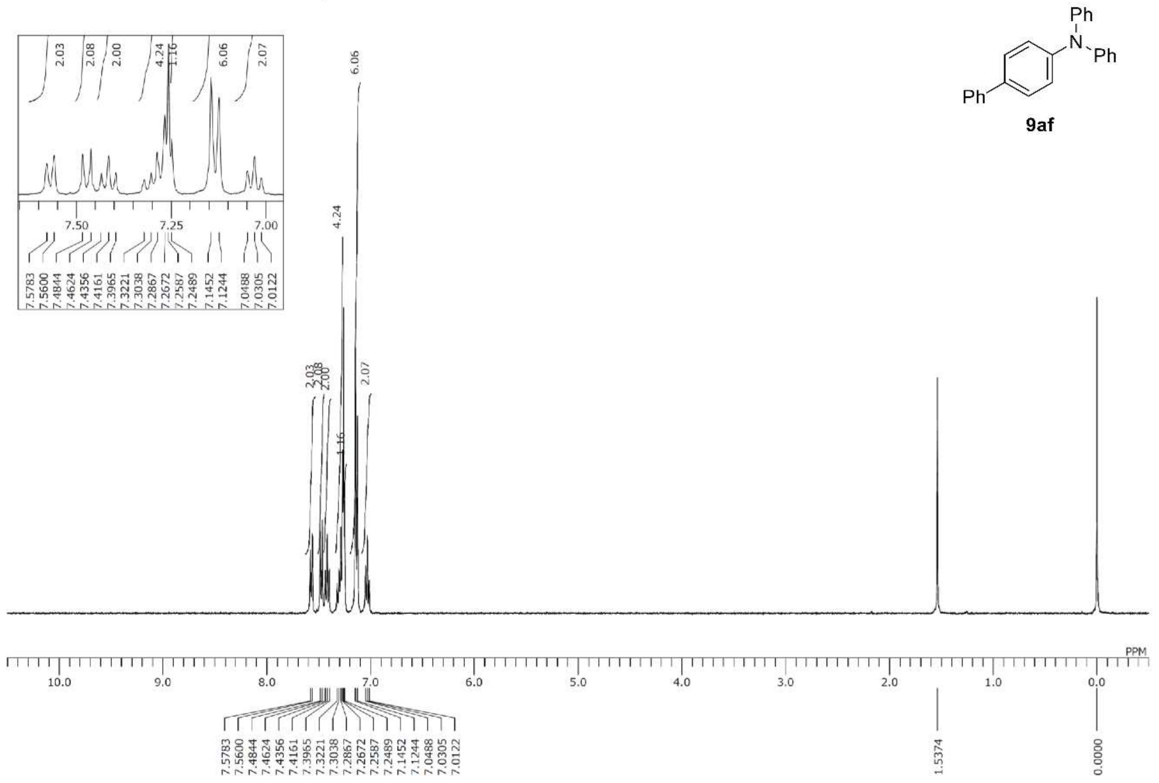

^{13}C NMR spectra of **9af** ($CDCl_3$, 150 MHz)

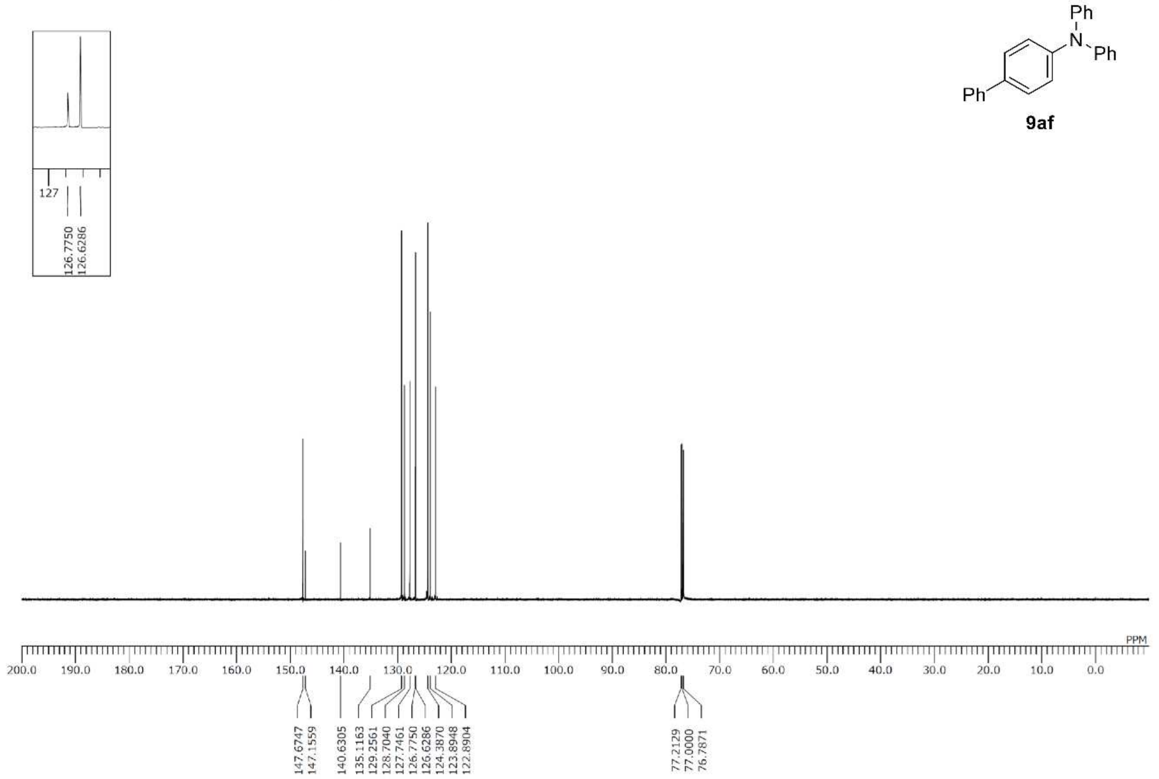

^{1}H NMR spectra of **9ag** ($CDCl_3$, 400 MHz)

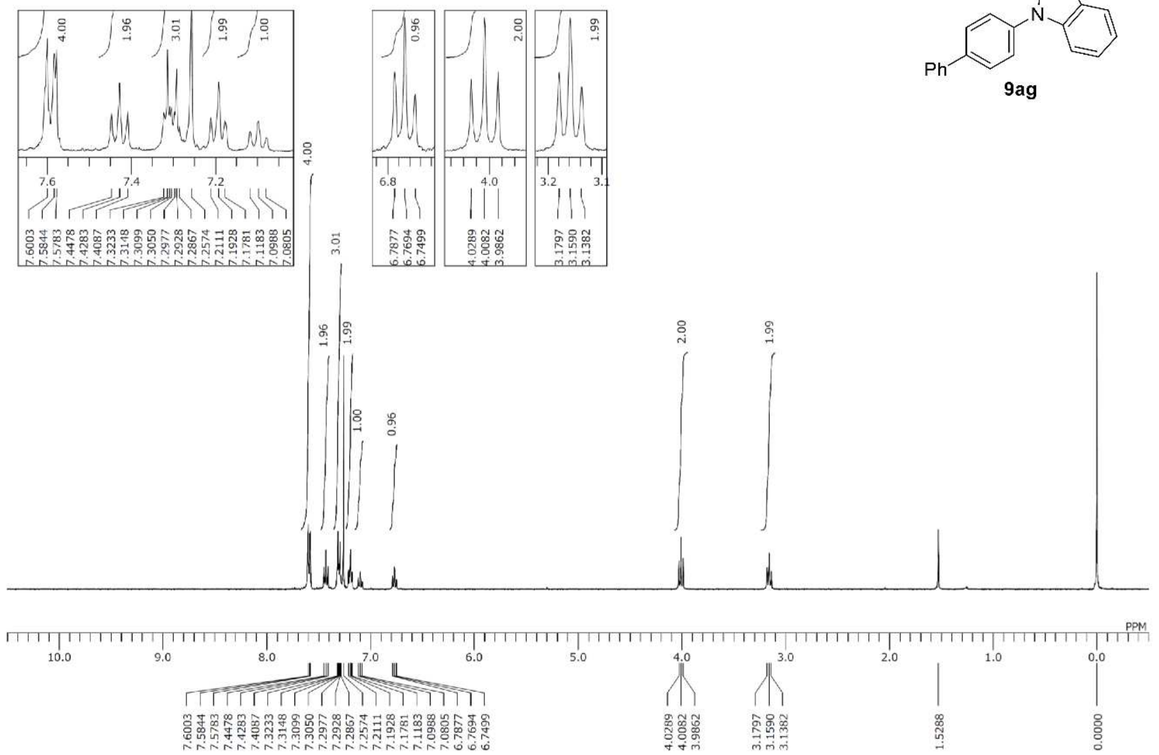

^{13}C NMR spectra of **9ag** ($CDCl_3$, 100 MHz)

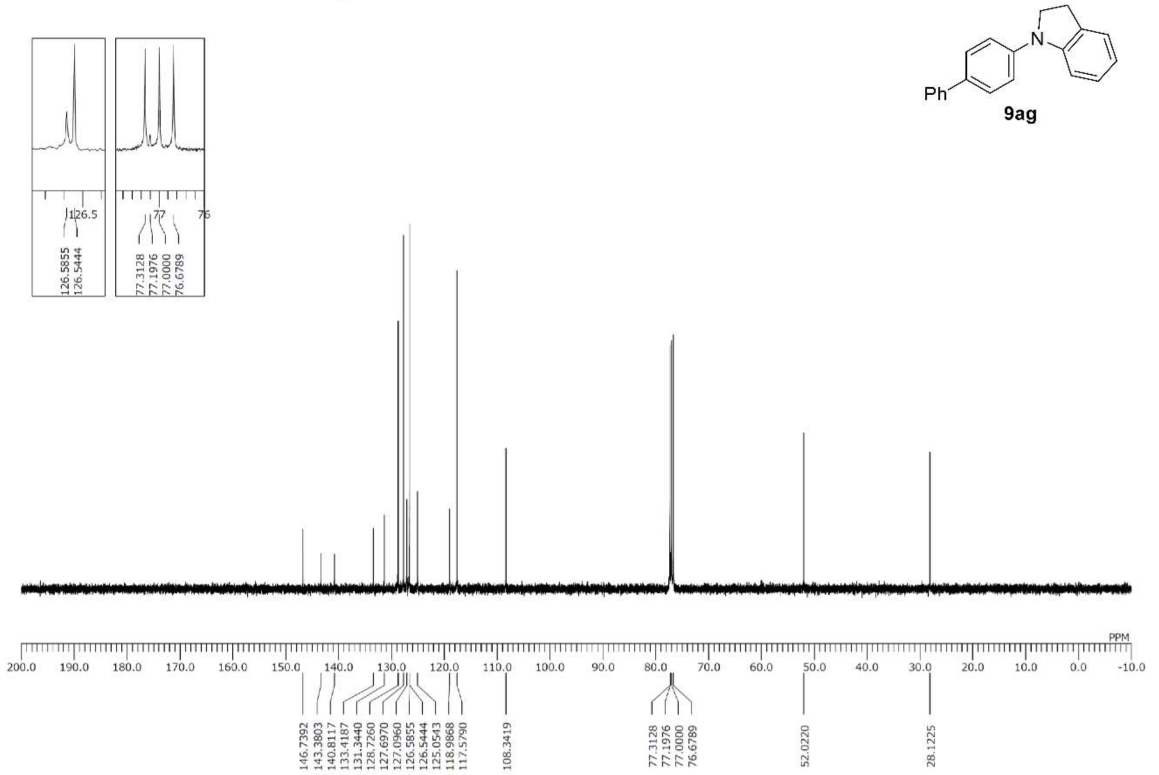

^{1}H NMR spectra of **9ah** ($CDCl_3$, 400 MHz)

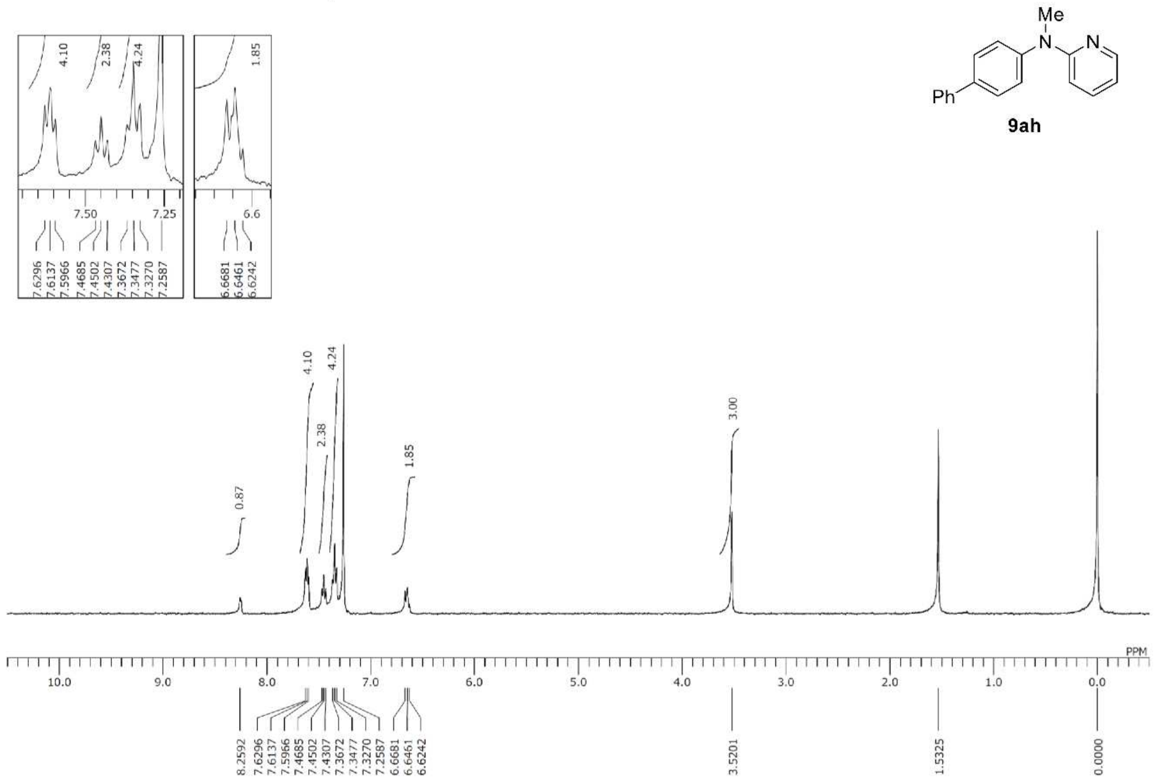

^{13}C NMR spectra of **9ah** ($CDCl_3$, 150 MHz)

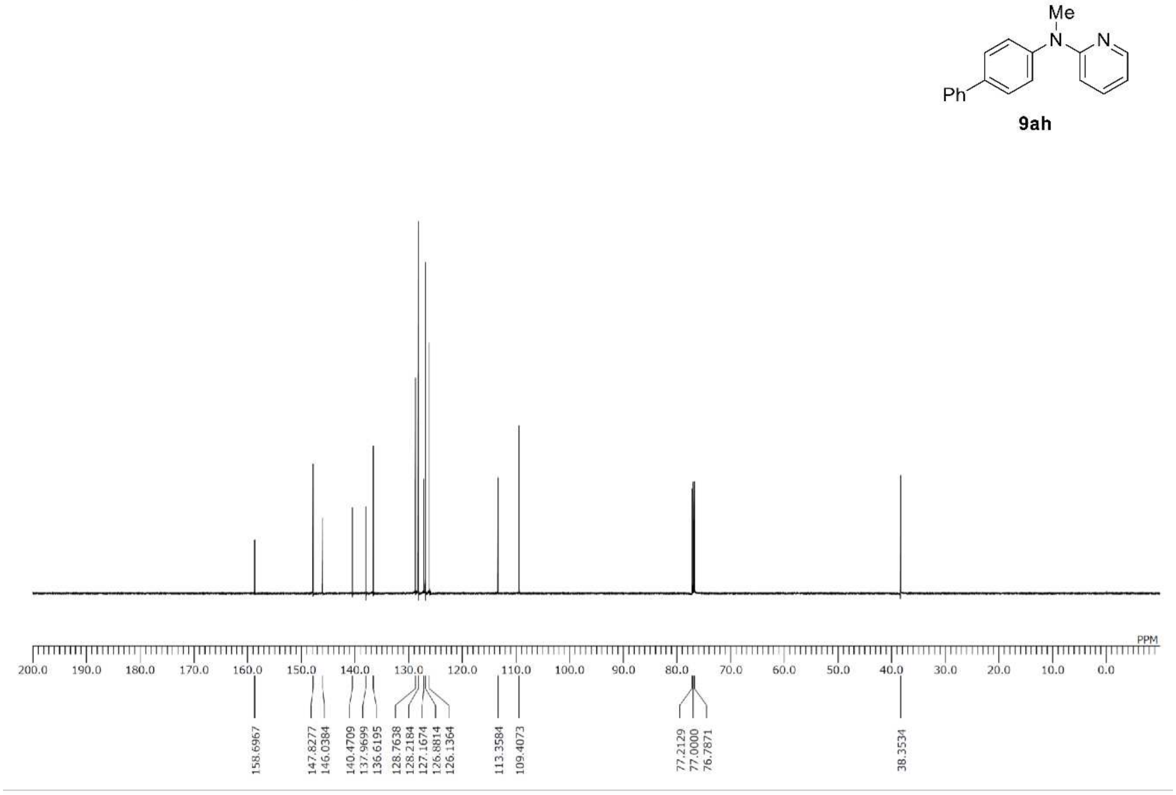

^{1}H NMR spectra of **9ai** ($CDCl_3$, 400 MHz)

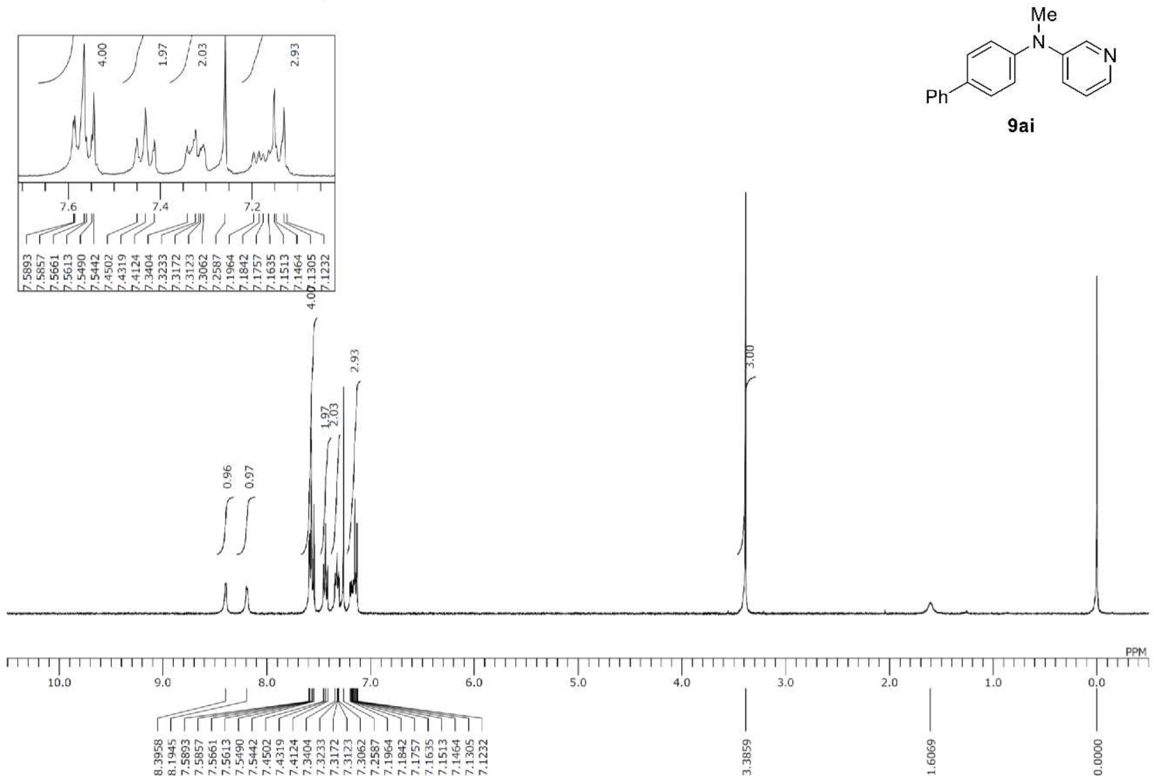

^{13}C NMR spectra of **9ai** ($CDCl_3$, 100 MHz)

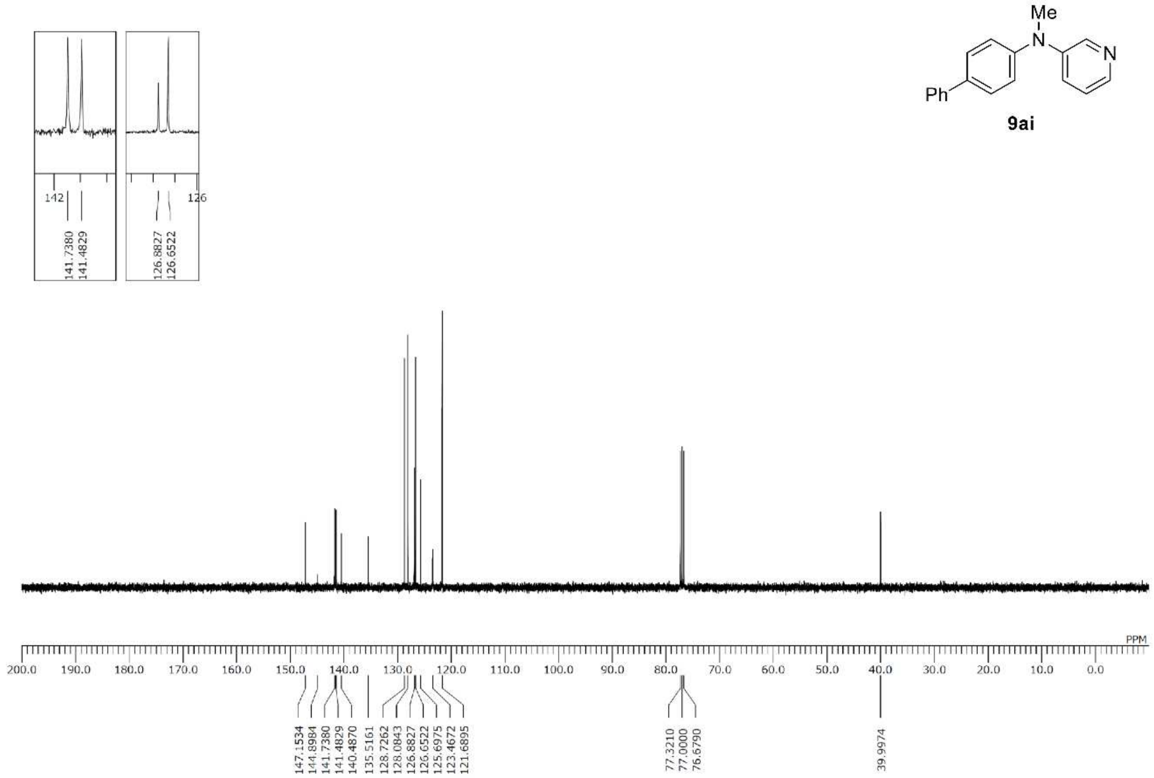

^{1}H NMR spectra of **9aj** ($CDCl_3$, 400 MHz)

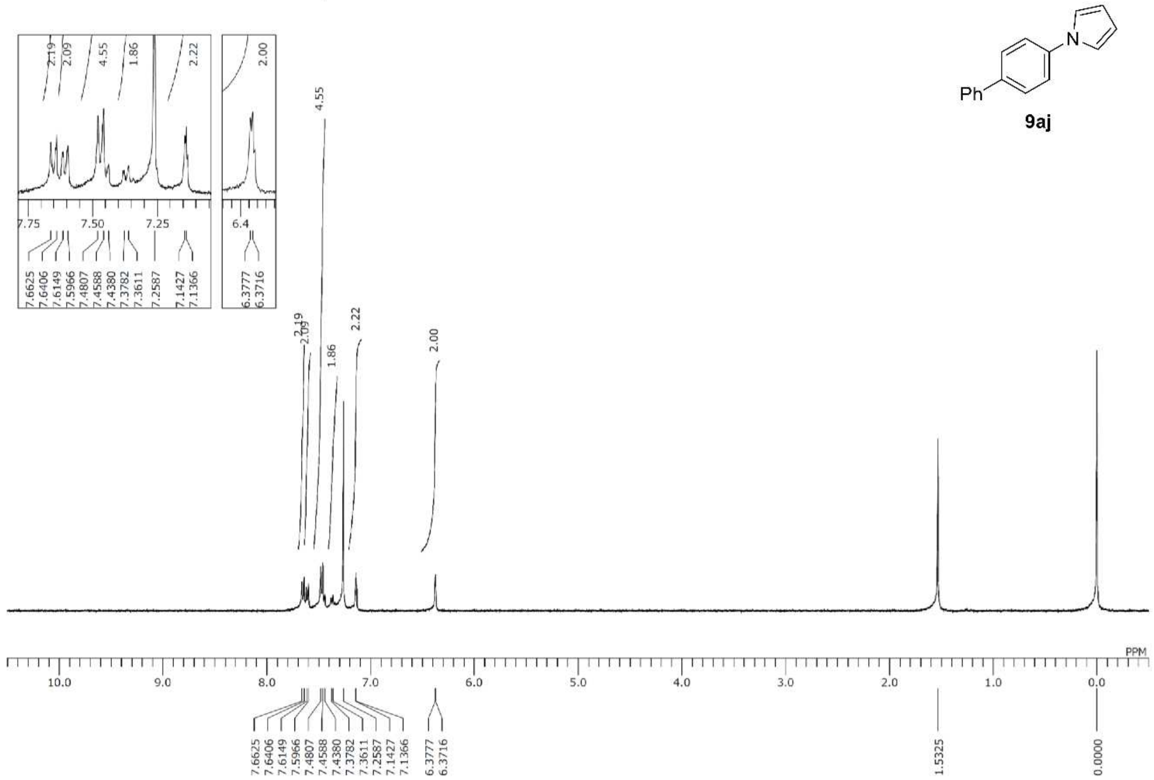

^{13}C NMR spectra of **9aj** ($CDCl_3$, 150 MHz)

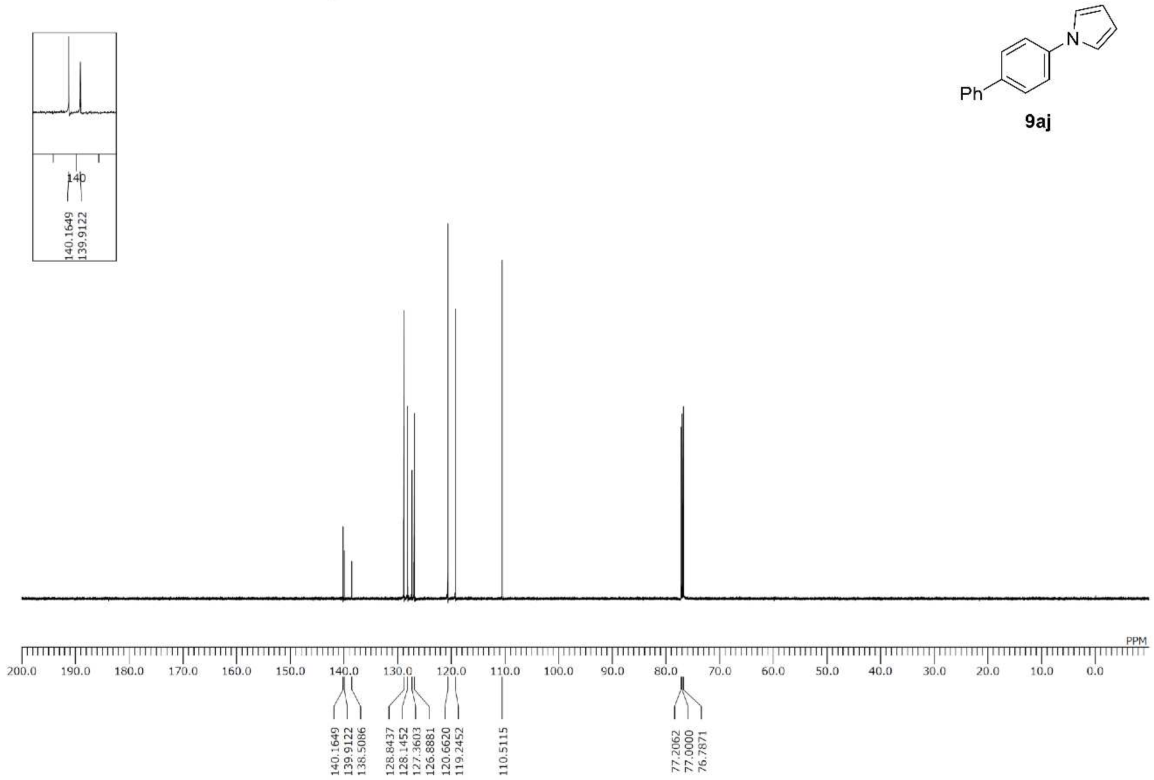

^{1}H NMR spectra of **9ak** ($CDCl_3$, 400 MHz)

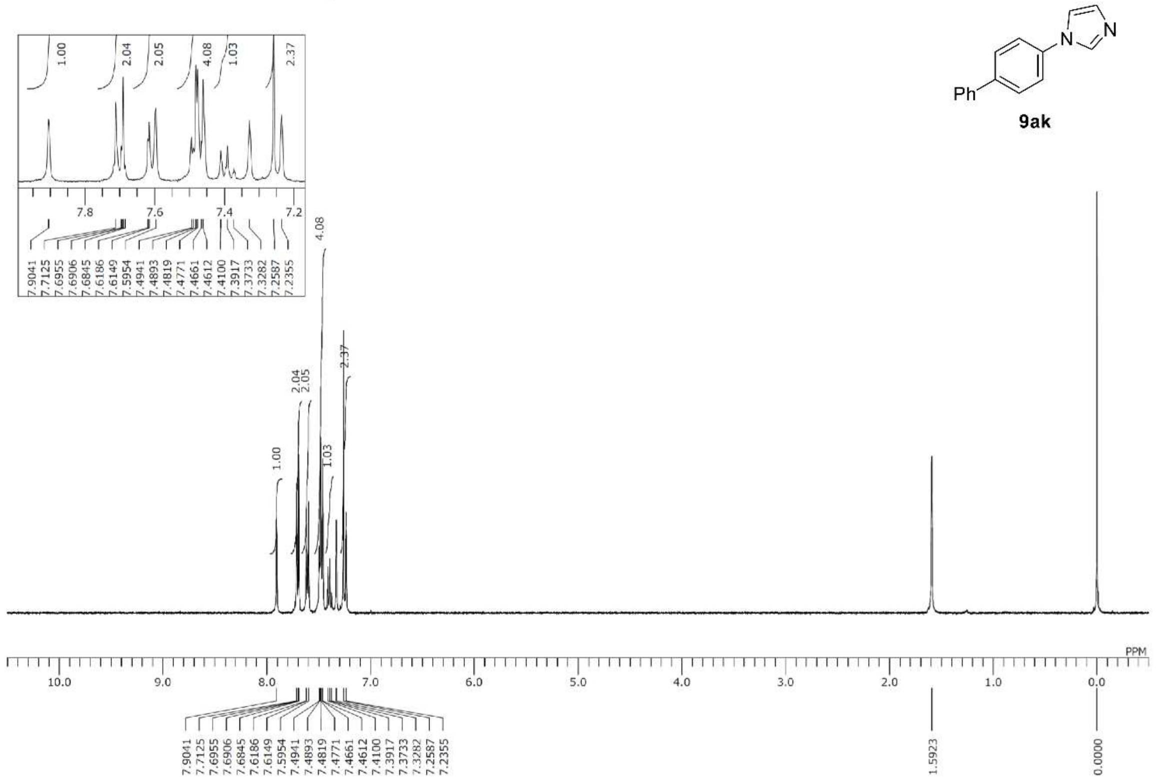

^{13}C NMR spectra of **9ak** ($CDCl_3$, 150 MHz)

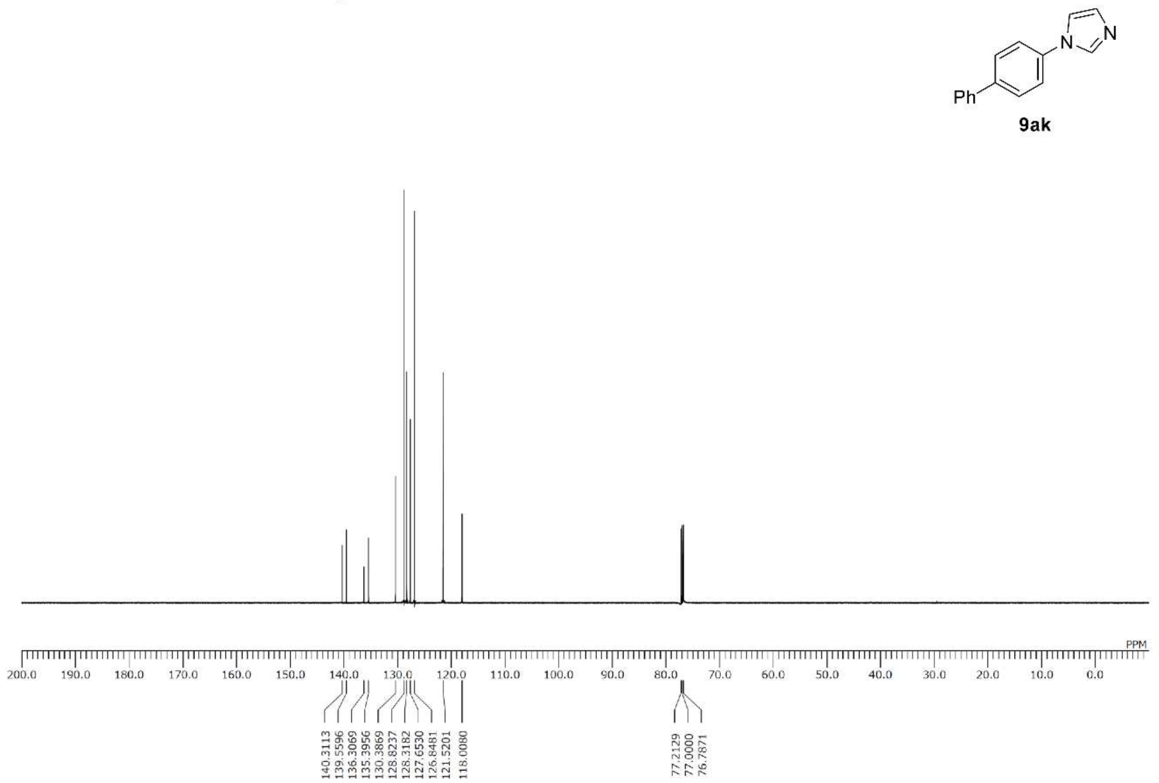

^1H NMR spectra of **9al** ($CDCl_3$, 400 MHz)

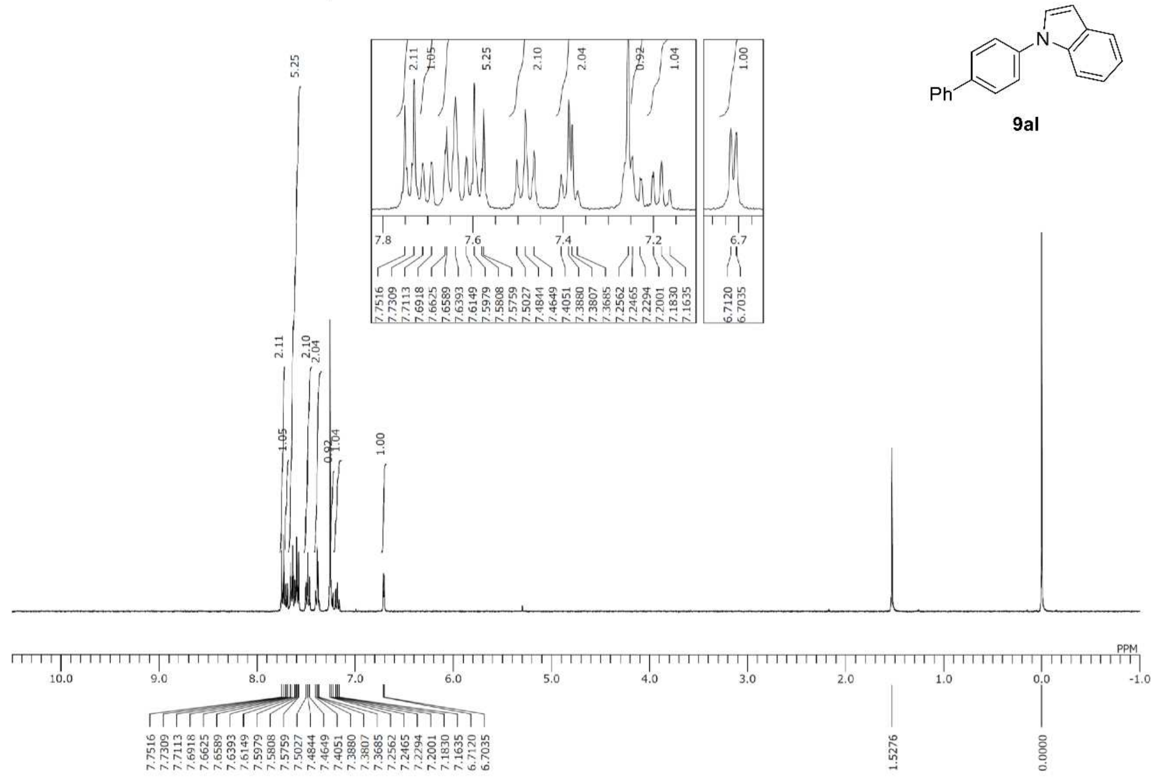

^{13}C NMR spectra of **9al** ($CDCl_3$, 100 MHz)

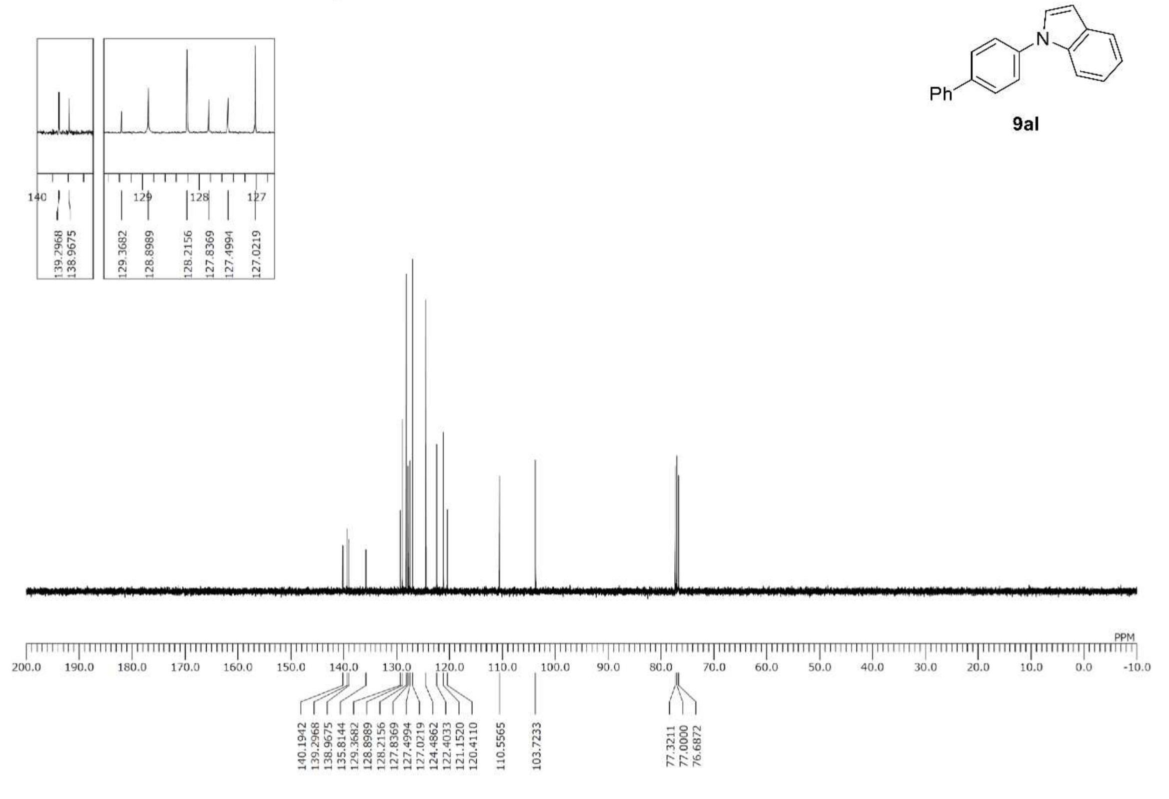

^{1}H NMR spectra of **10aa** ($CDCl_3$, 400 MHz)

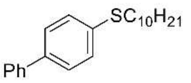

10aa

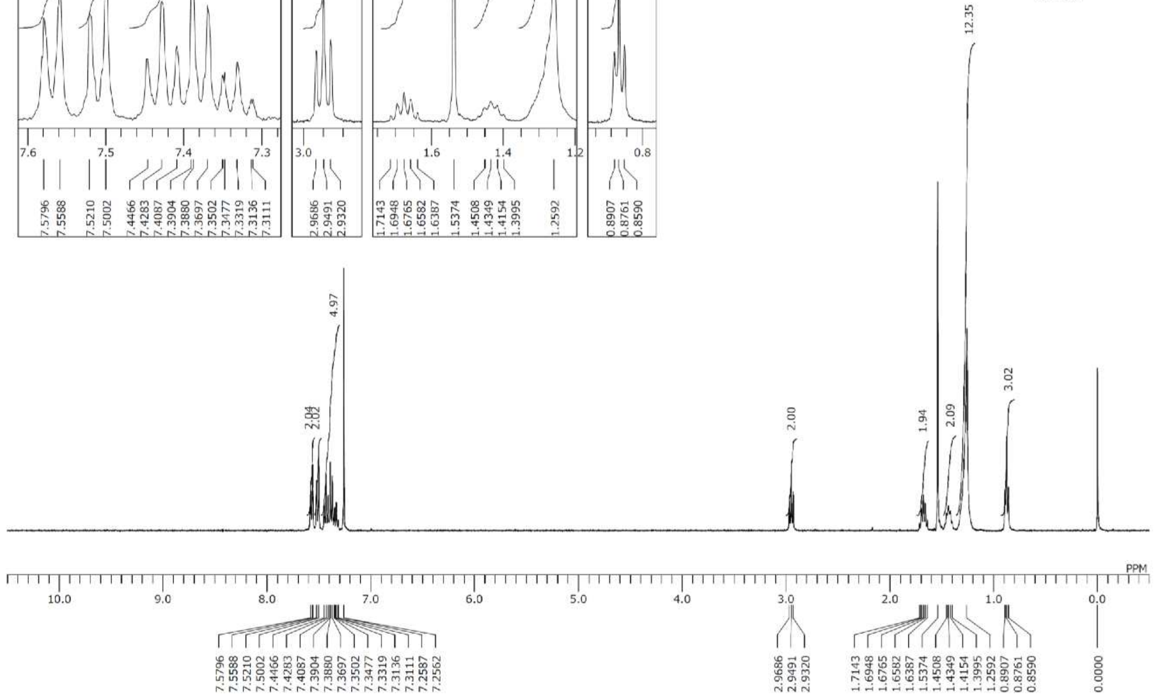

^{13}C NMR spectra of **10aa** (C_6D_6, 150 MHz)

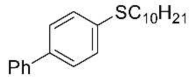

10aa

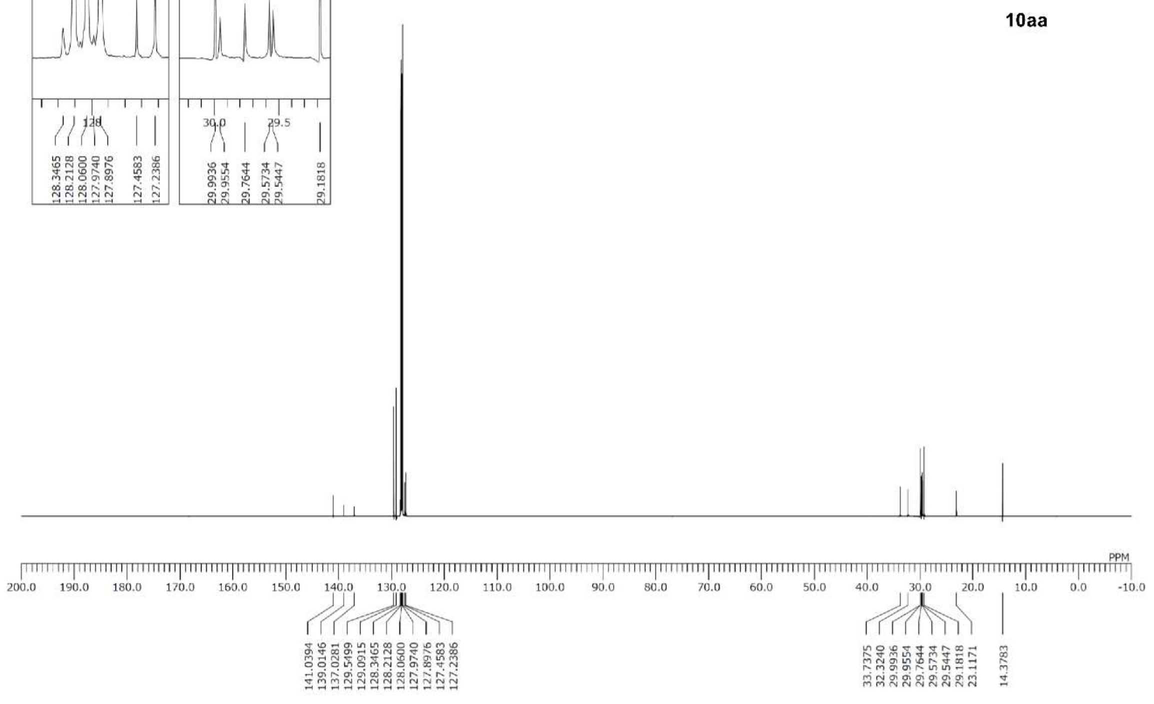

^{1}H NMR spectra of **10ab** ($CDCl_3$, 400 MHz)

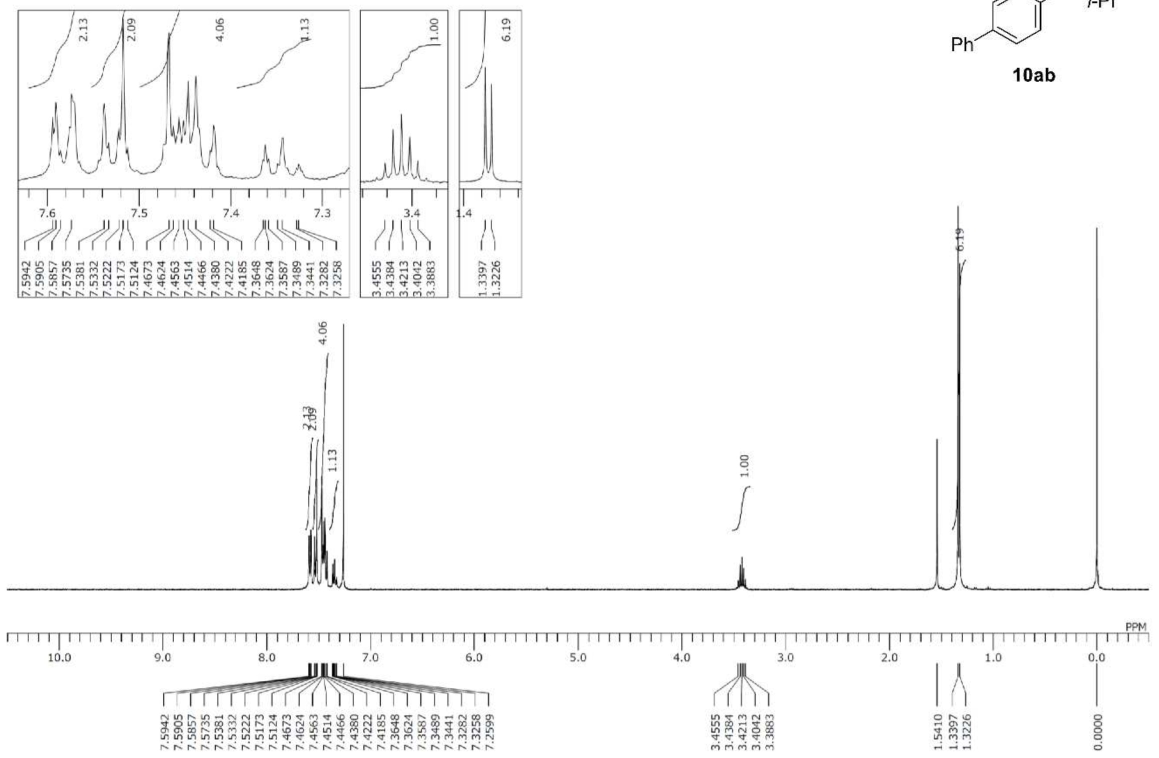

^{13}C NMR spectra of **10ab** ($CDCl_3$, 150 MHz)

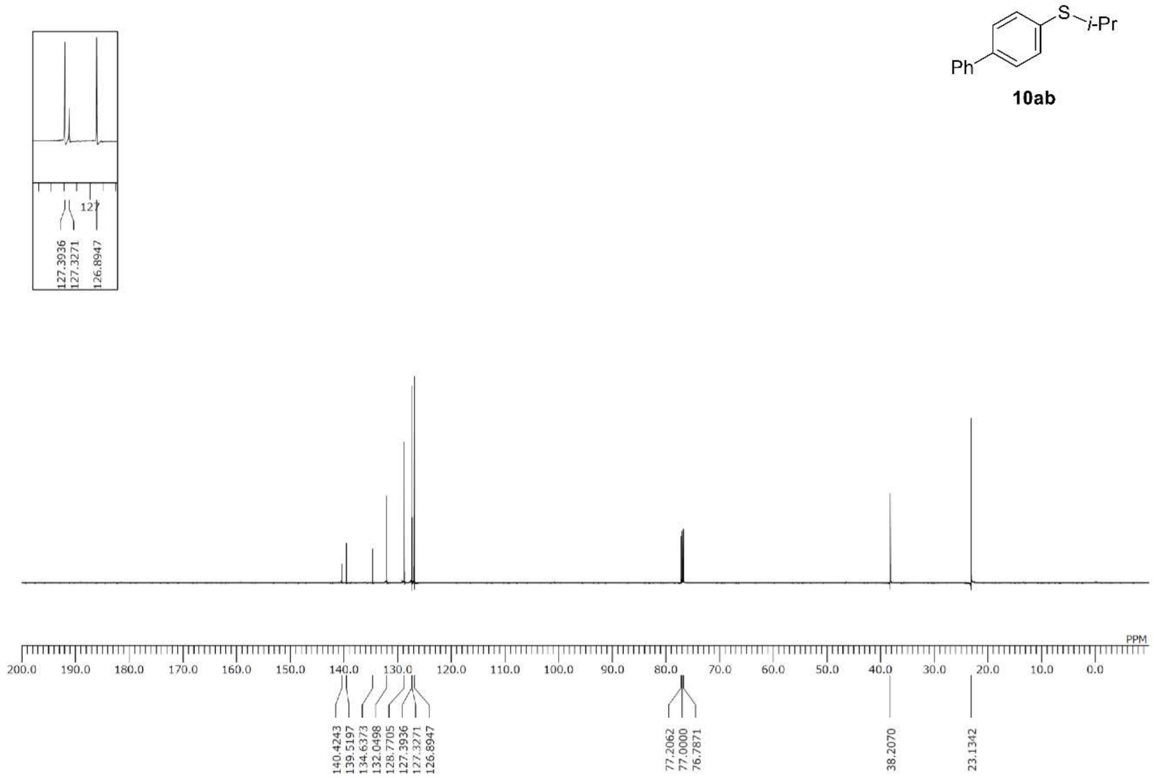

^{1}H NMR spectra of **10ac** ($CDCl_3$, 400 MHz)

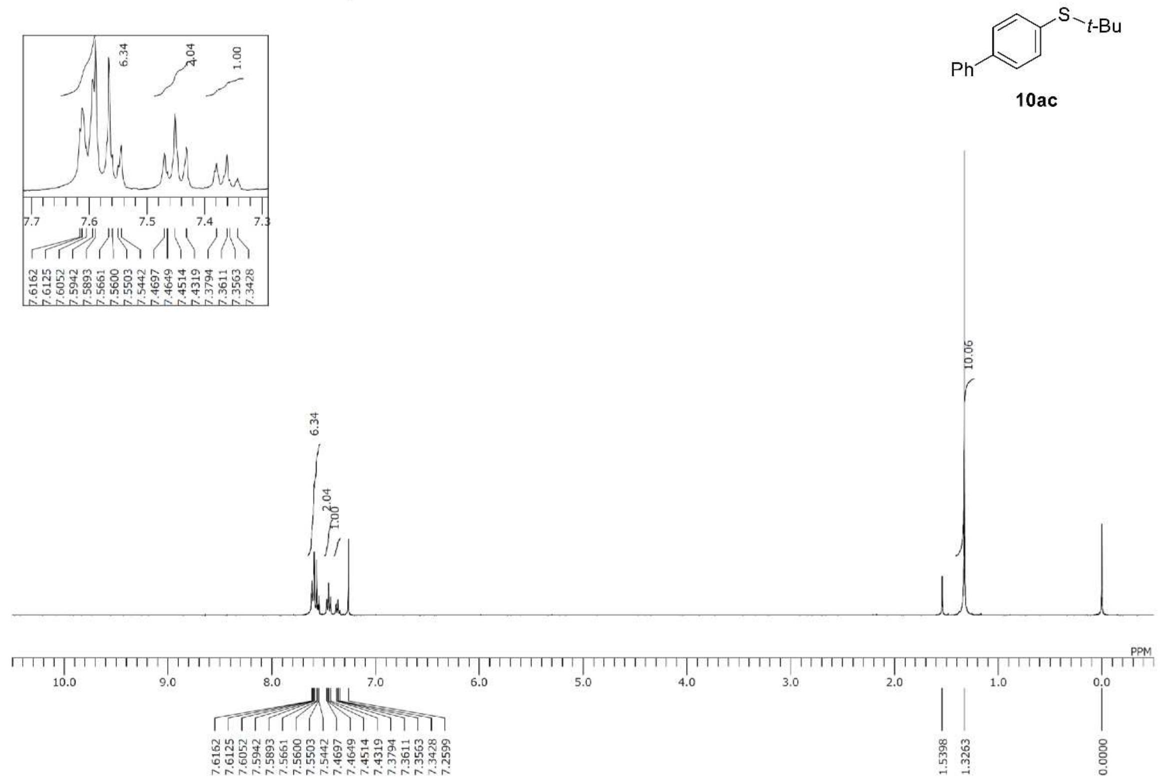

^{13}C NMR spectra of **10ac** ($CDCl_3$, 150 MHz)

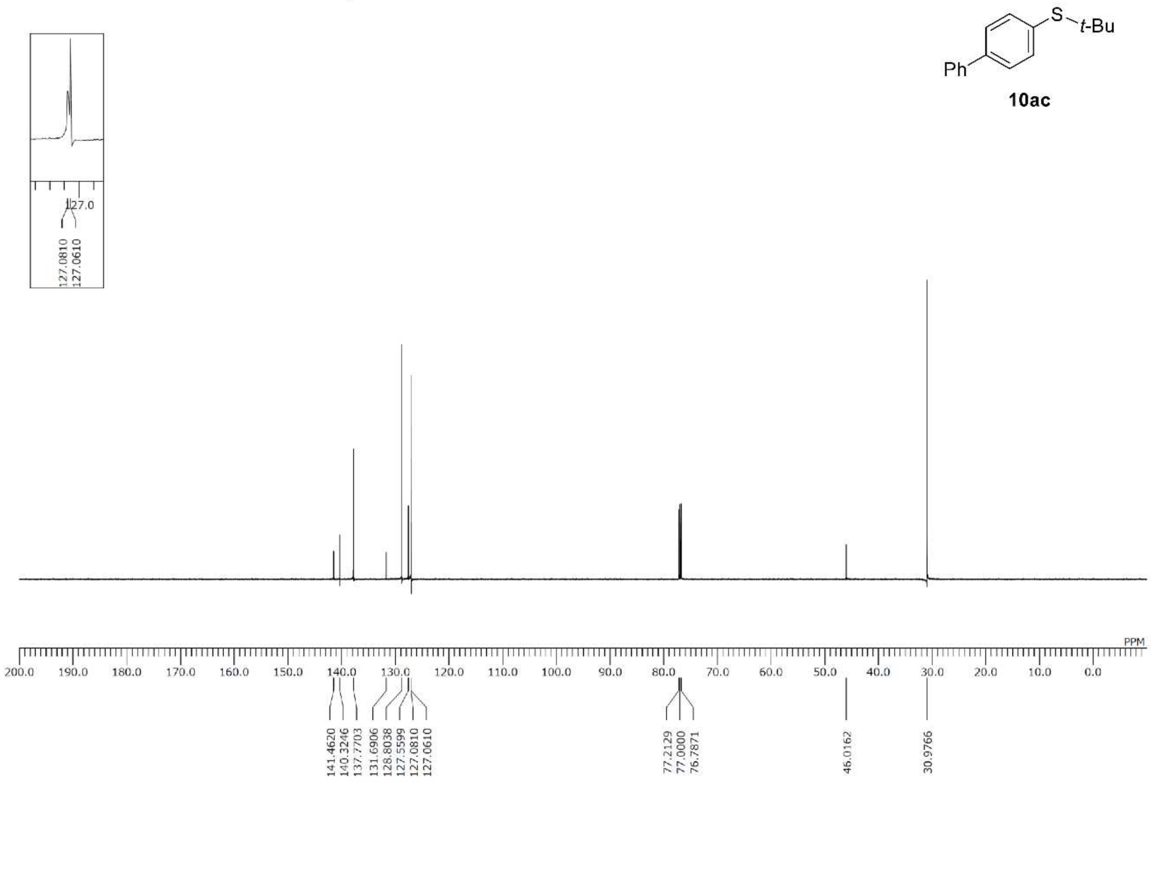

^{1}H NMR spectra of **10ad** ($CDCl_3$, 400 MHz)

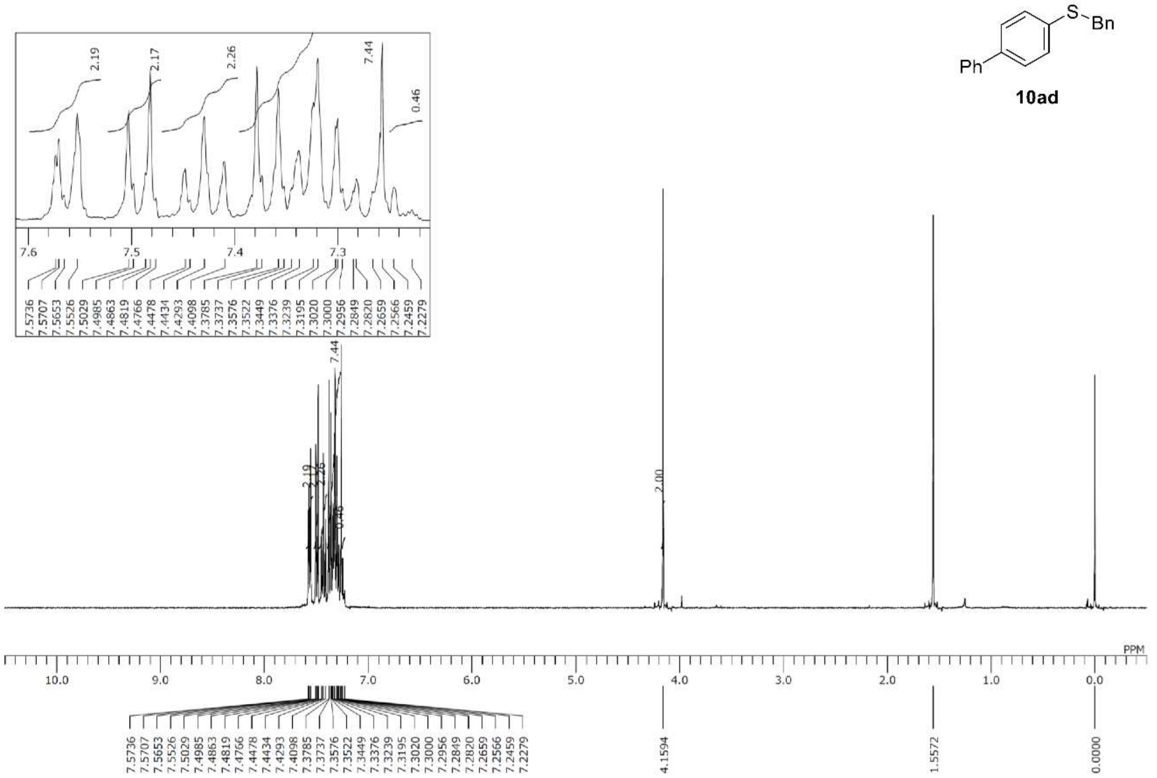

10ad

^{13}C NMR spectra of **10ad** ($CDCl_3$, 100 MHz)

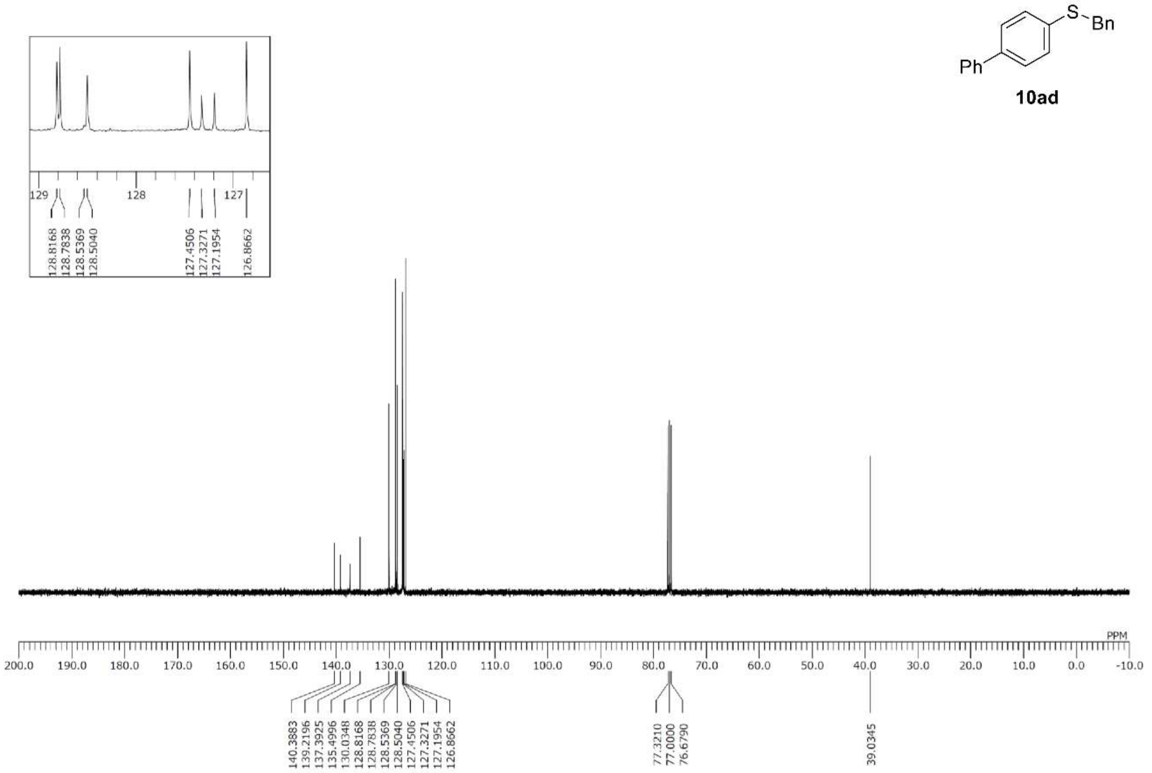

10ad

^{1}H NMR spectra of **10ae** ($CDCl_3$, 400 MHz)

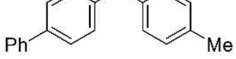

10ae

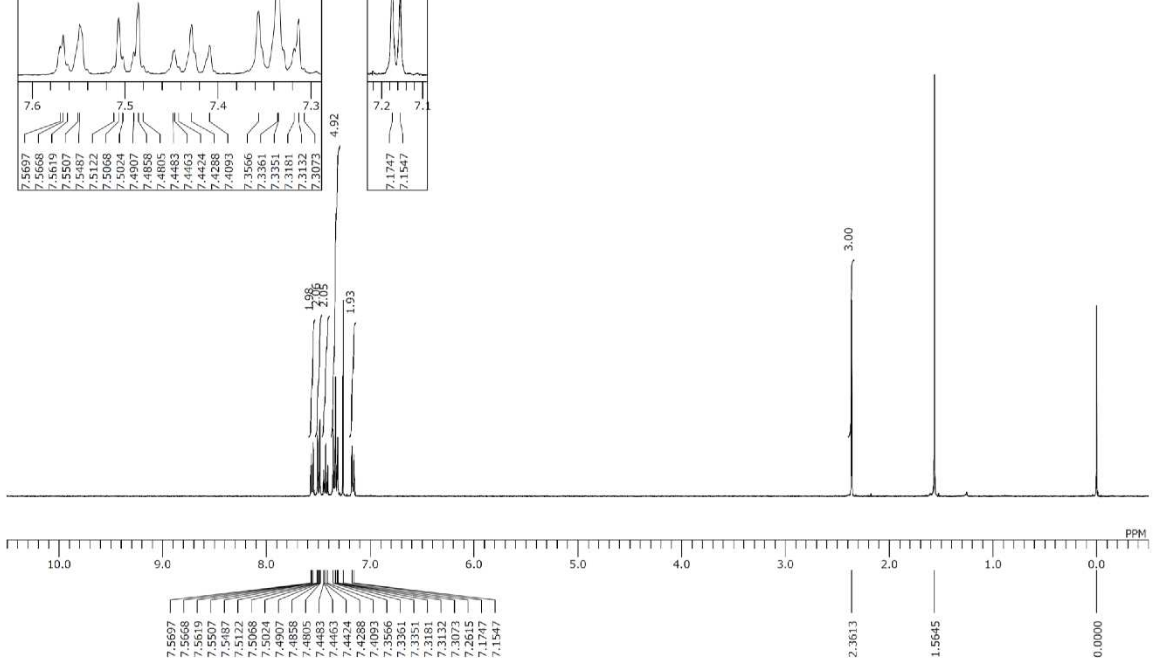

^{13}C NMR spectra of **10ae** ($CDCl_3$, 100 MHz)

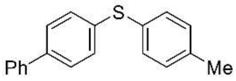

10ae

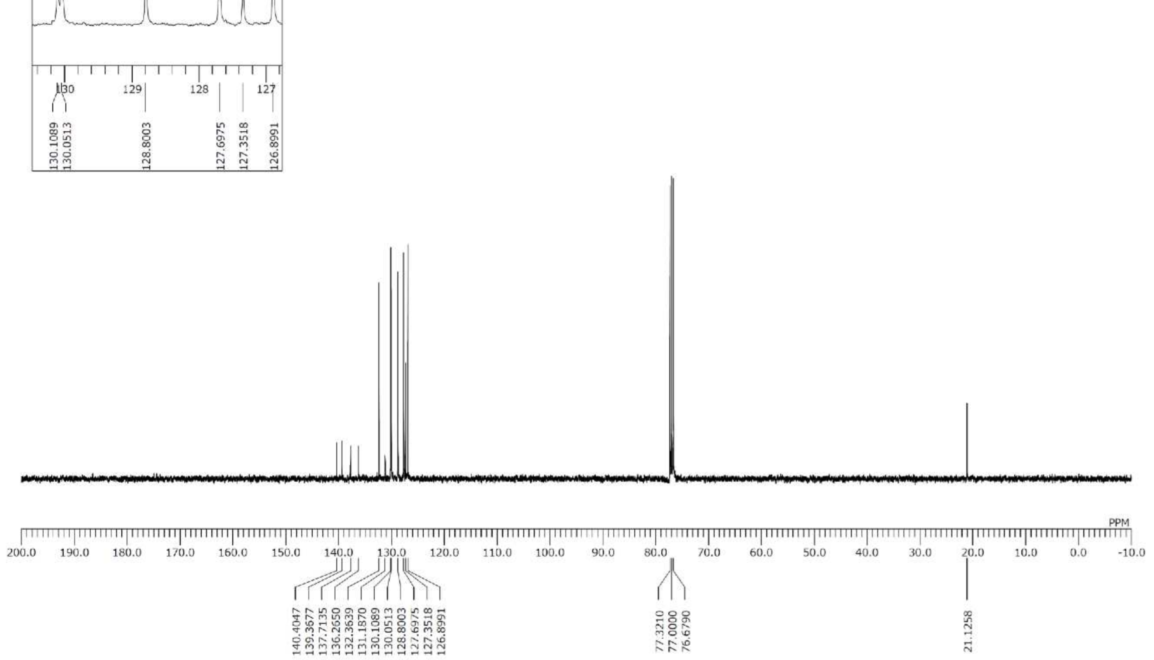

^{1}H NMR spectra of **11aa** ($CDCl_3$, 400 MHz)

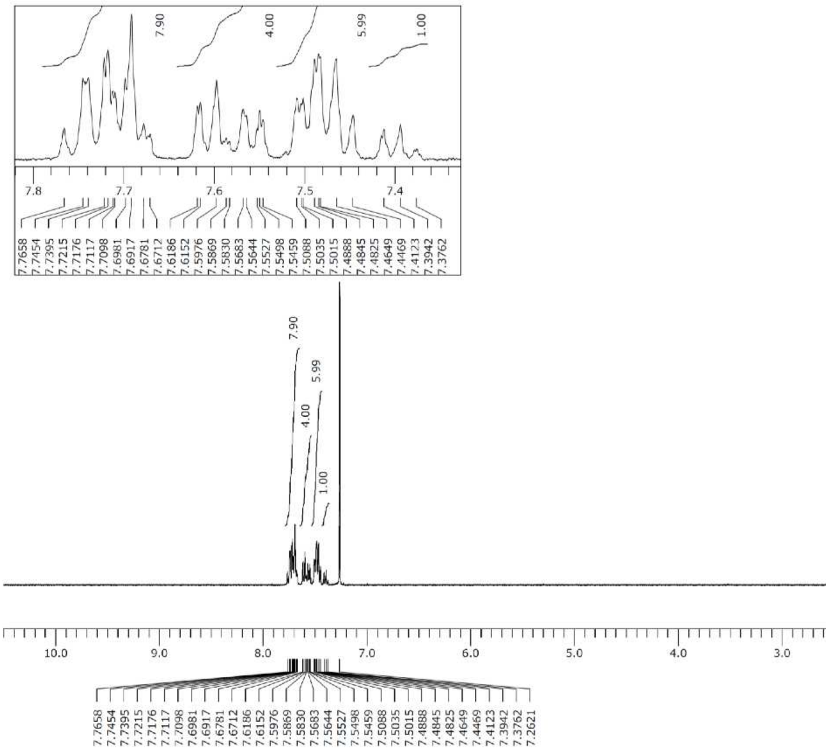

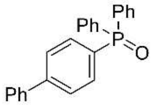

11aa

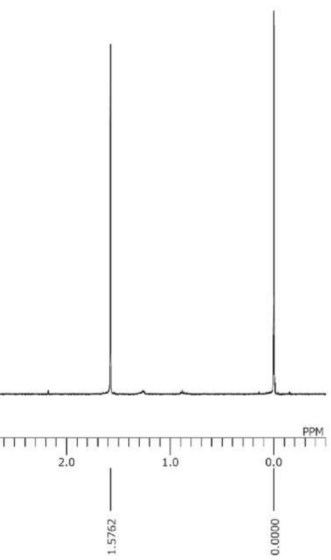

^{13}C NMR spectra of **11aa** ($CDCl_3$, 150 MHz)

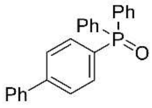

11aa

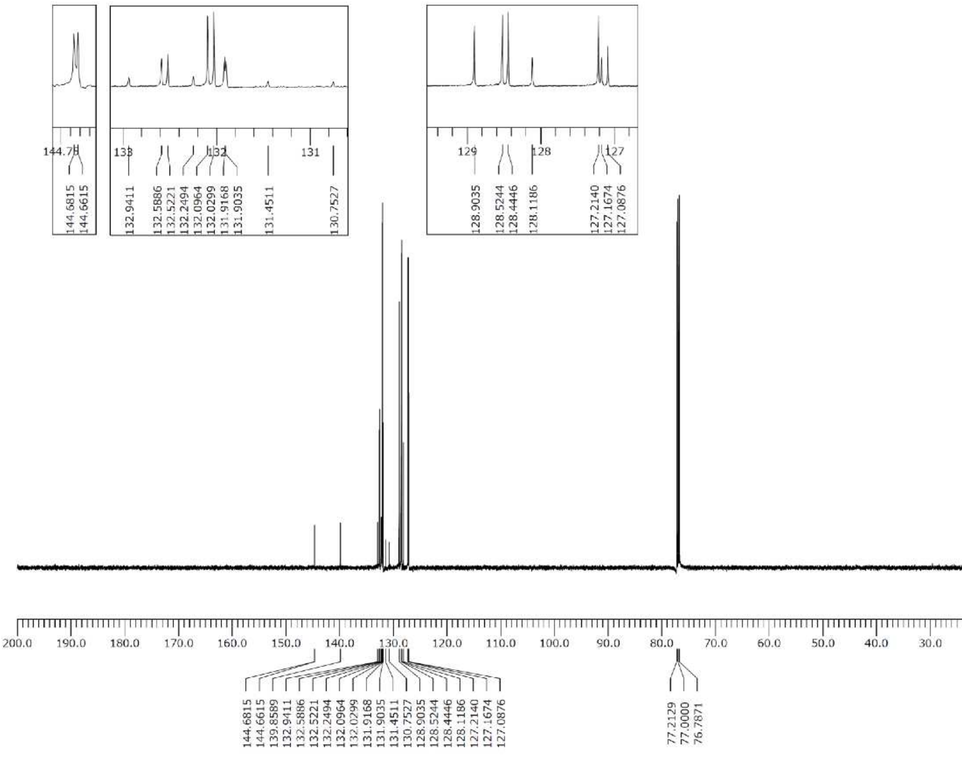

^{31}P NMR spectra of **11aa** ($CDCl_3$, 243 MHz)

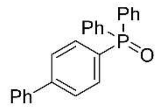

11aa

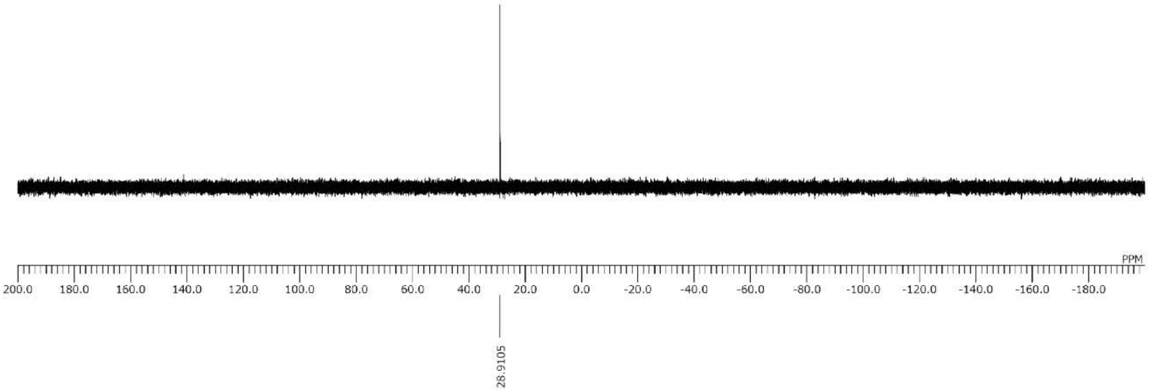

^{1}H NMR spectra of **11ab** ($CDCl_3$, 400 MHz)

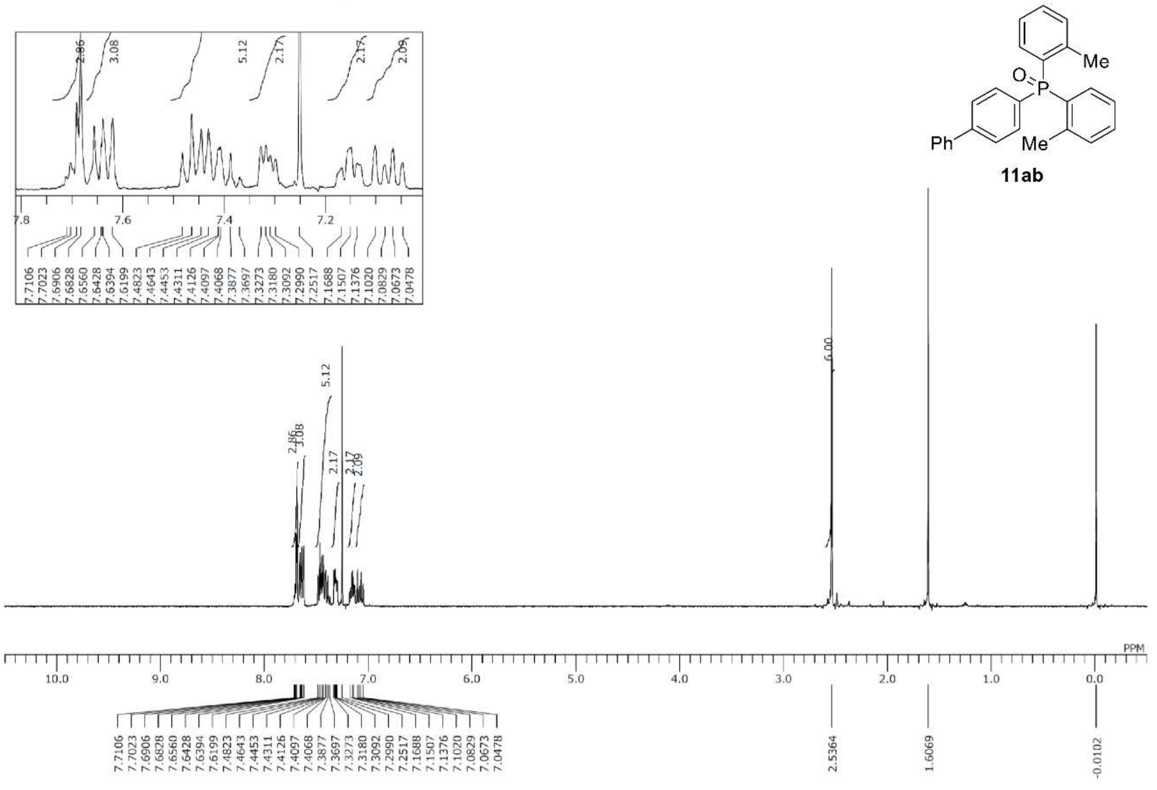

^{13}C NMR spectra of **11ab** ($CDCl_3$, 150 MHz)

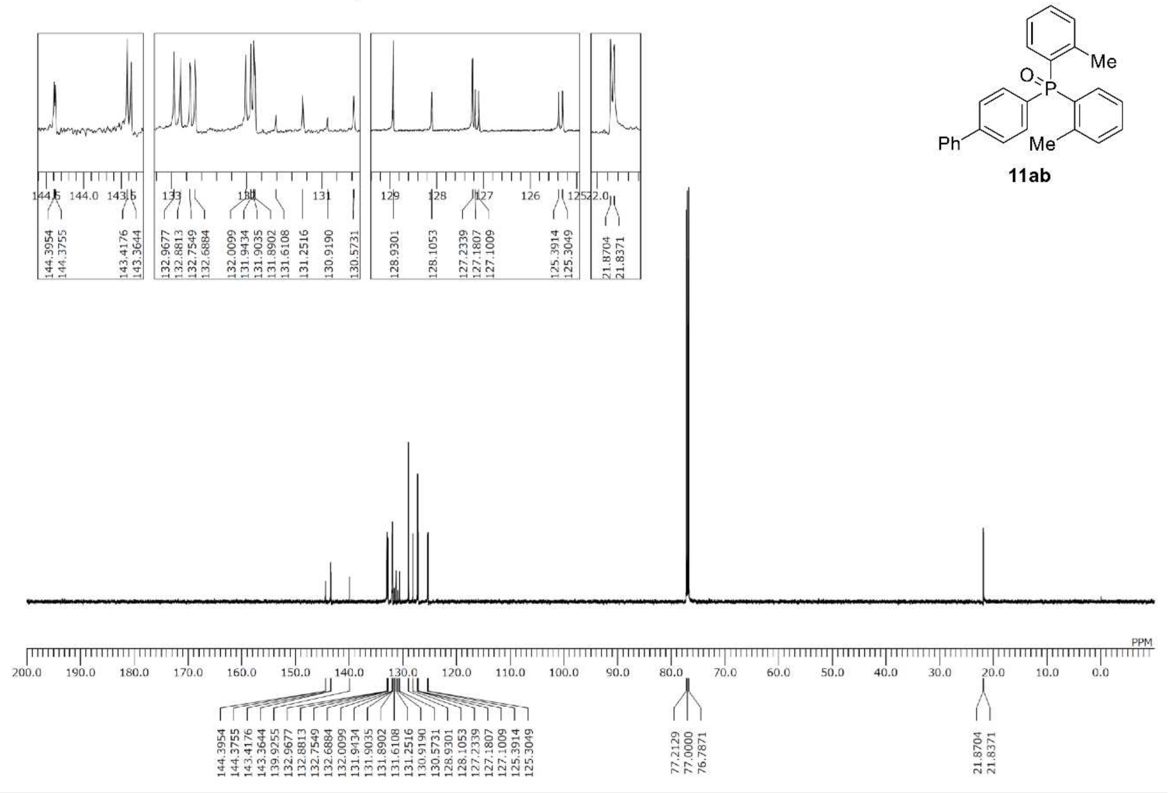

^{31}P NMR spectra of **11ab** ($CDCl_3$, 243 MHz)

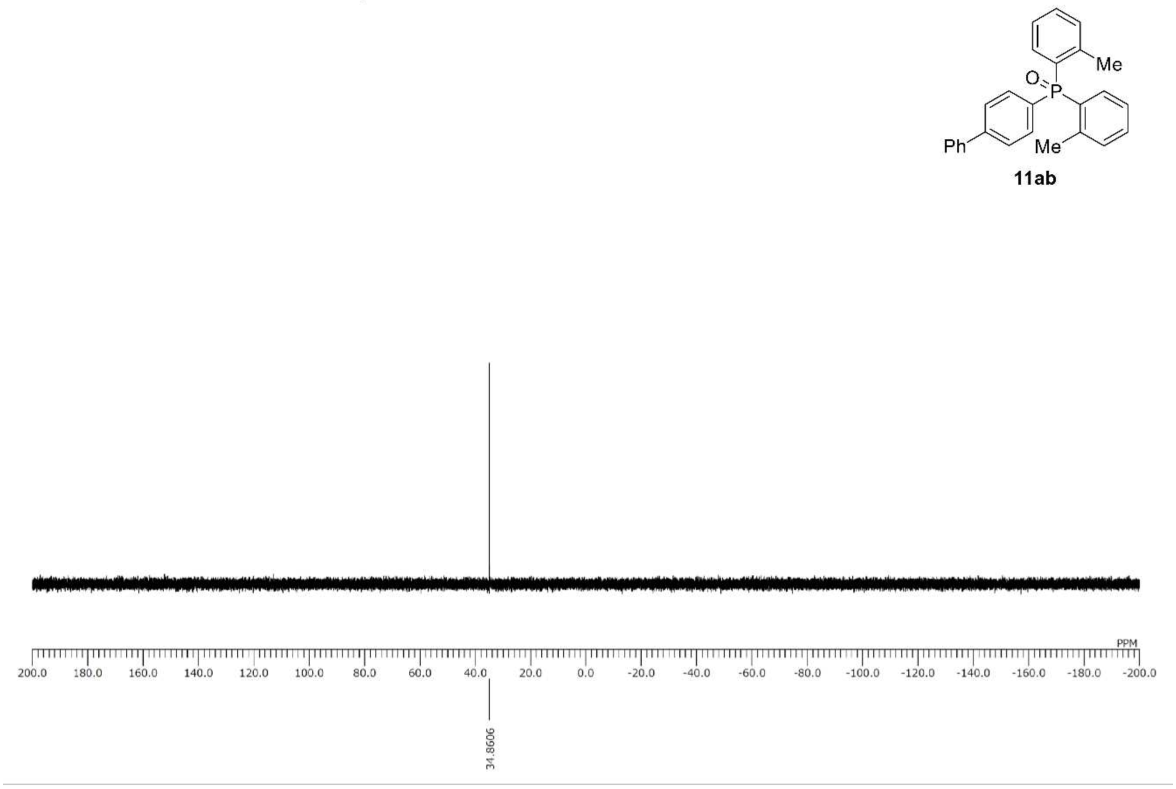

^{1}H NMR spectra of **3Ma** ($CDCl_3$, 400 MHz)

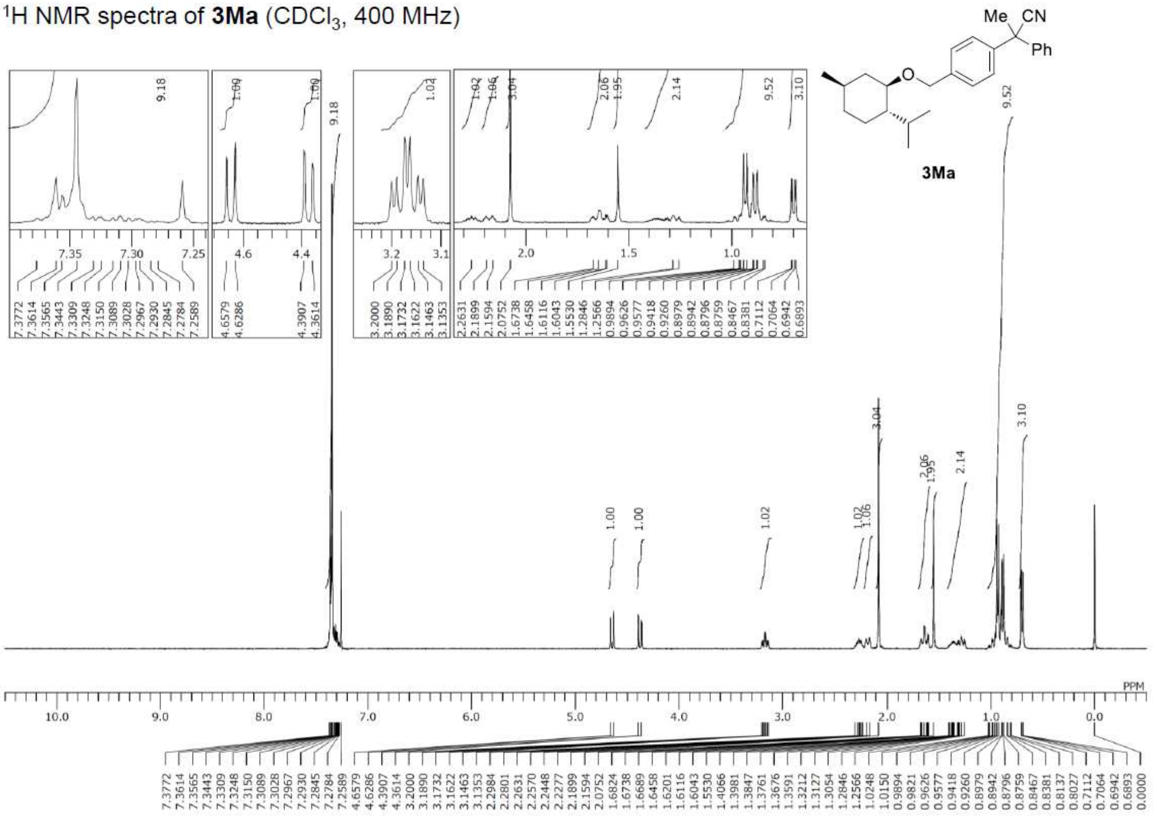

^{13}C NMR spectra of **3Ma** (DMSO-d_6, 100 MHz)

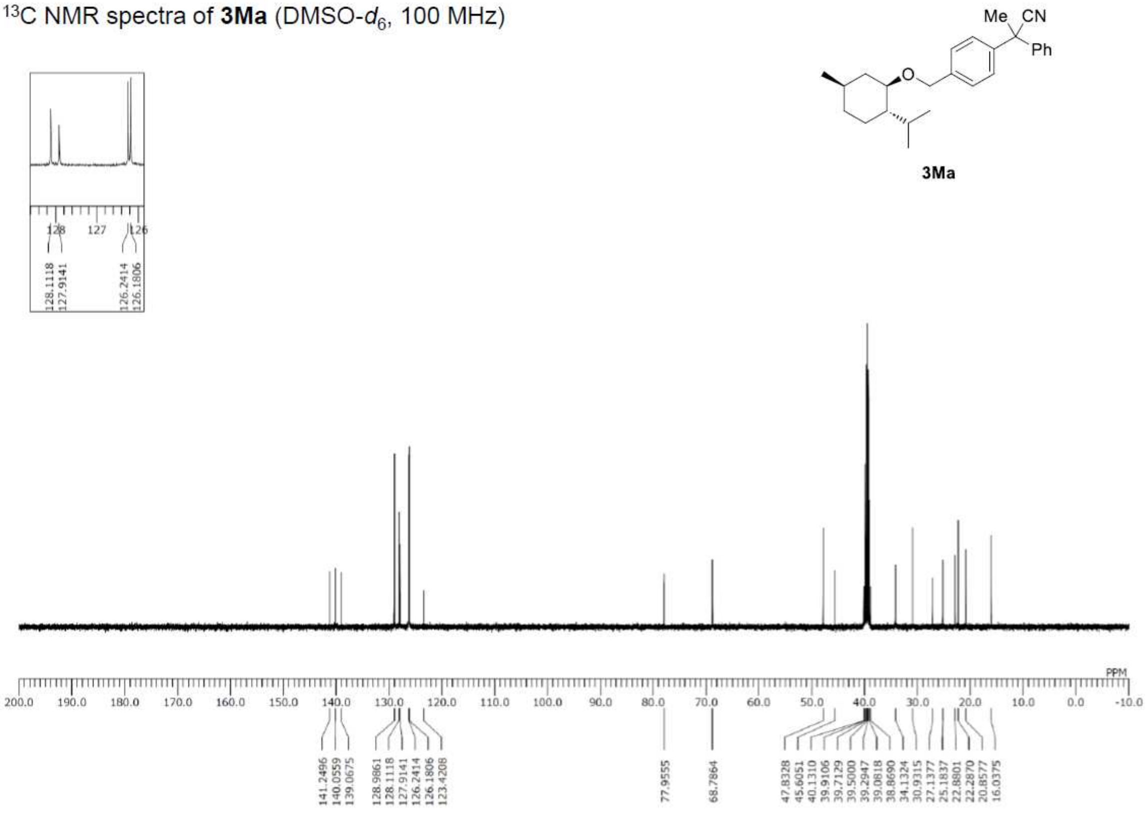

^{1}H NMR spectra of **3Na** ($CDCl_3$, 400 MHz)

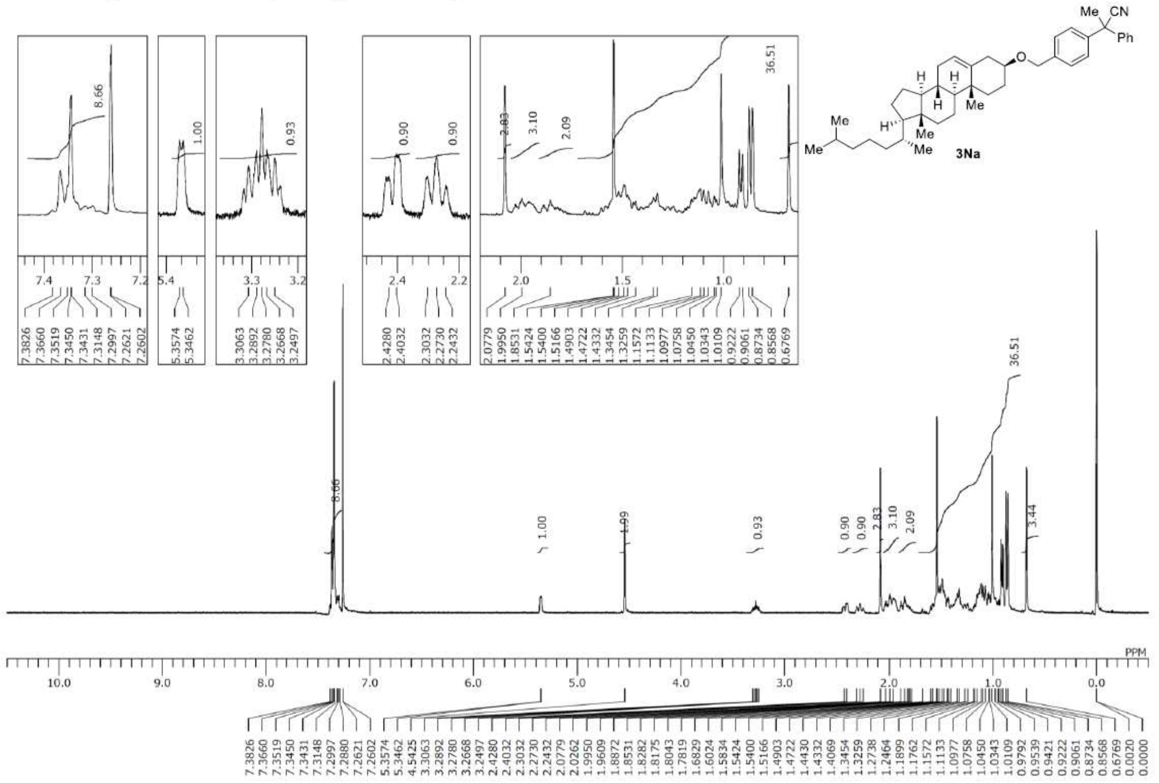

^{13}C NMR spectra of **3Na** ($CDCl_3$, 100 MHz)

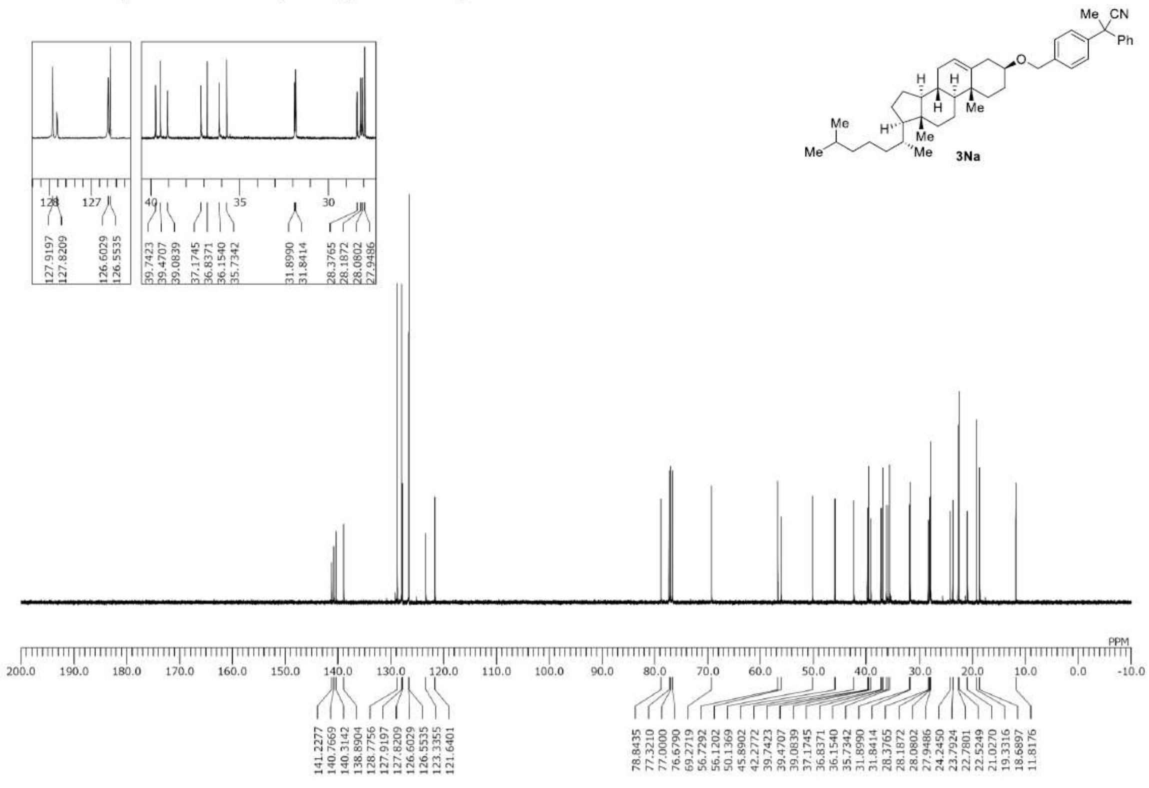

^{1}H NMR spectra of **3Oa** ($CDCl_3$, 400 MHz)

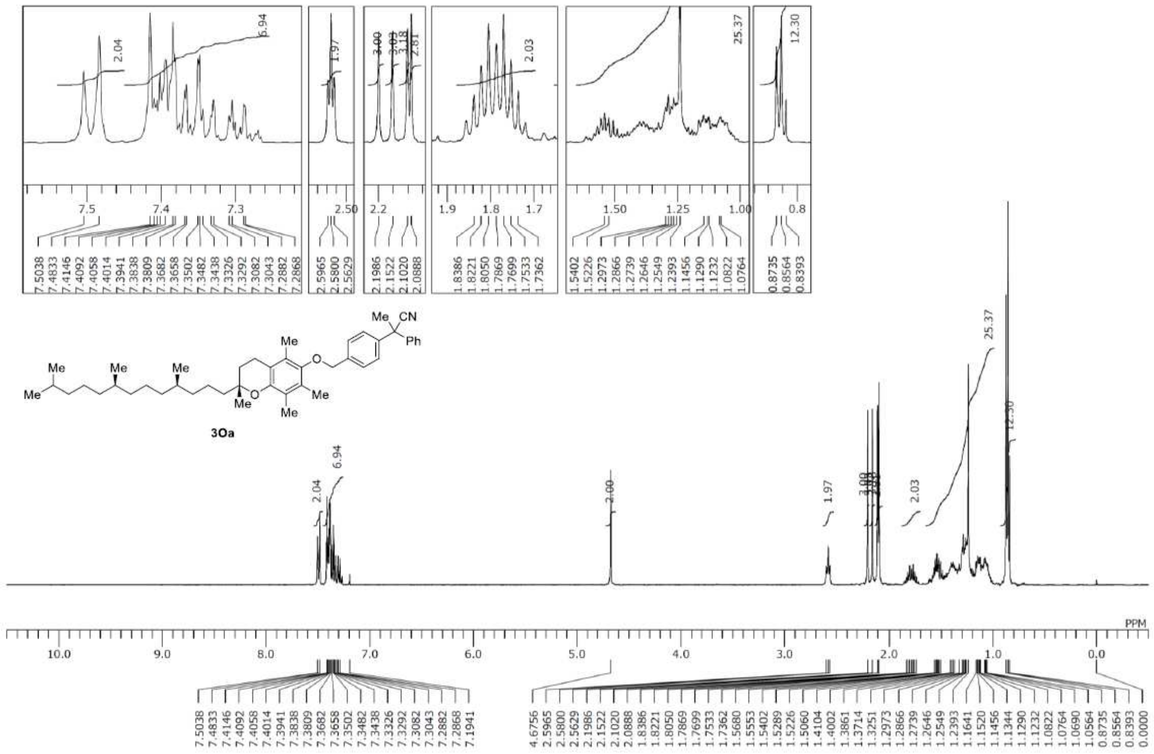

^{13}C NMR spectra of **3Oa** ($CDCl_3$, 100 MHz)

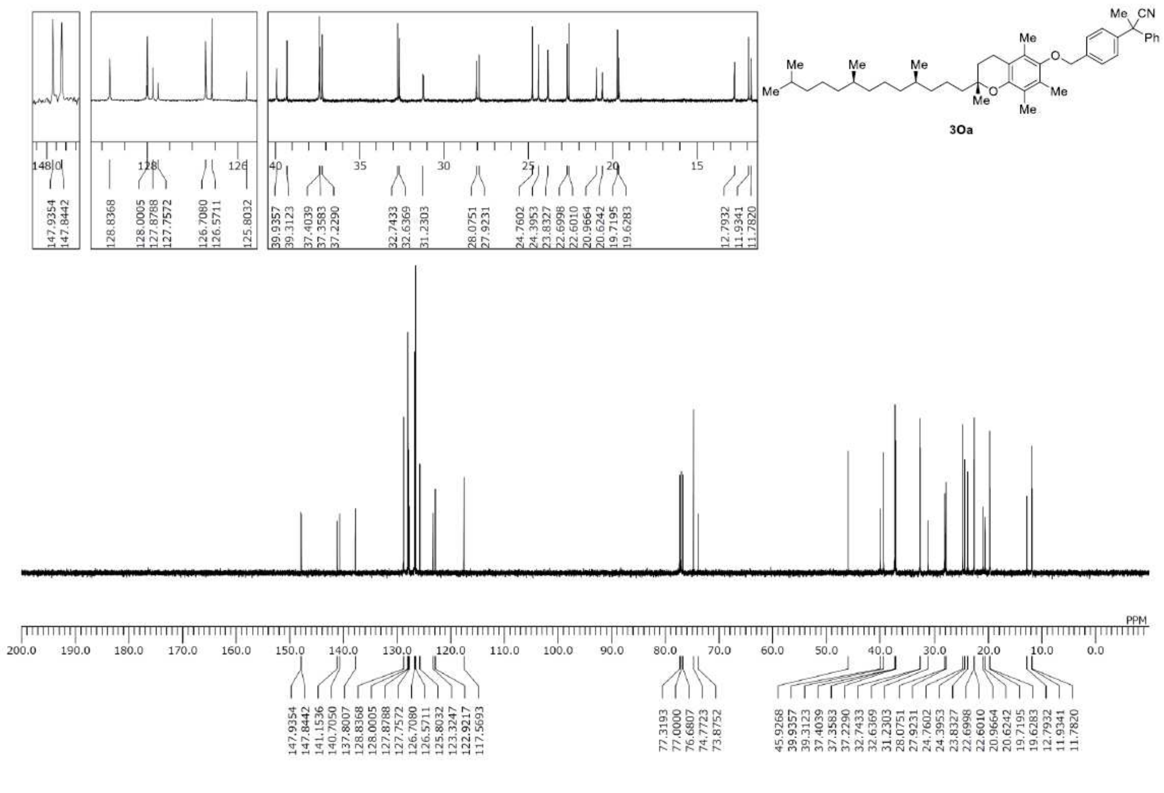

^{1}H NMR spectra of **3Pa** ($CDCl_3$, 600 MHz)

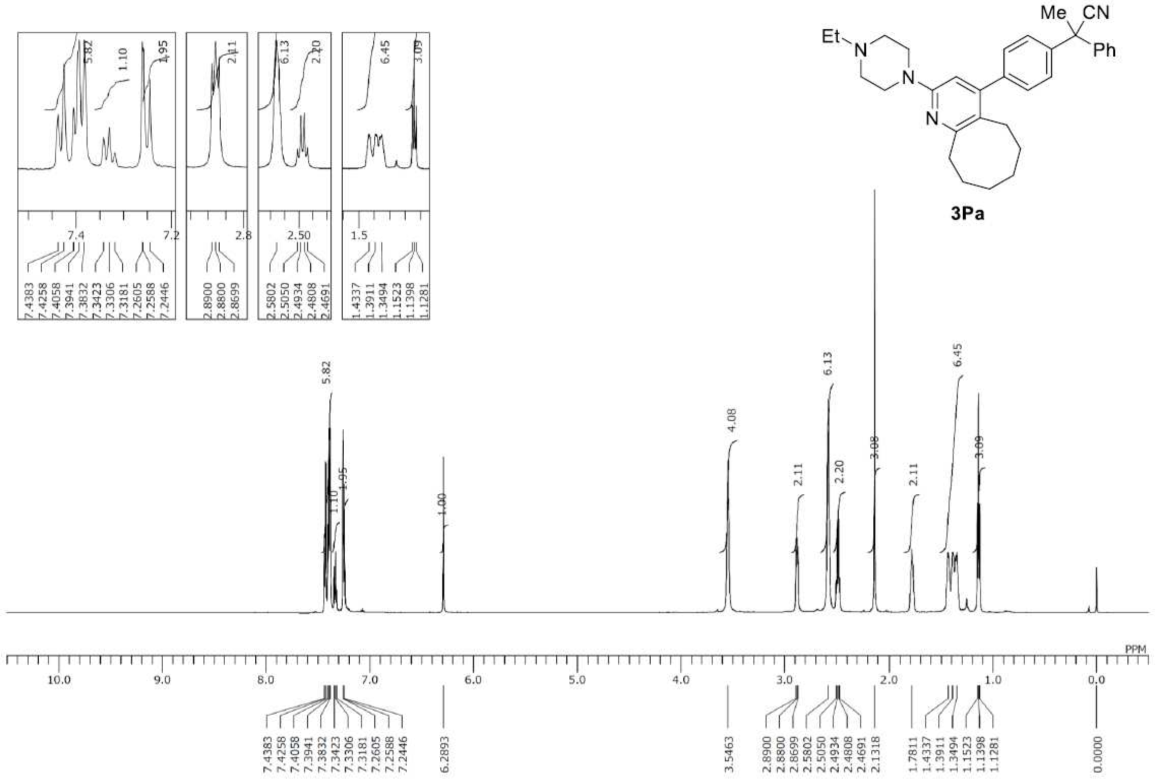

^{13}C NMR spectra of **3Pa** ($CDCl_3$, 100 MHz)

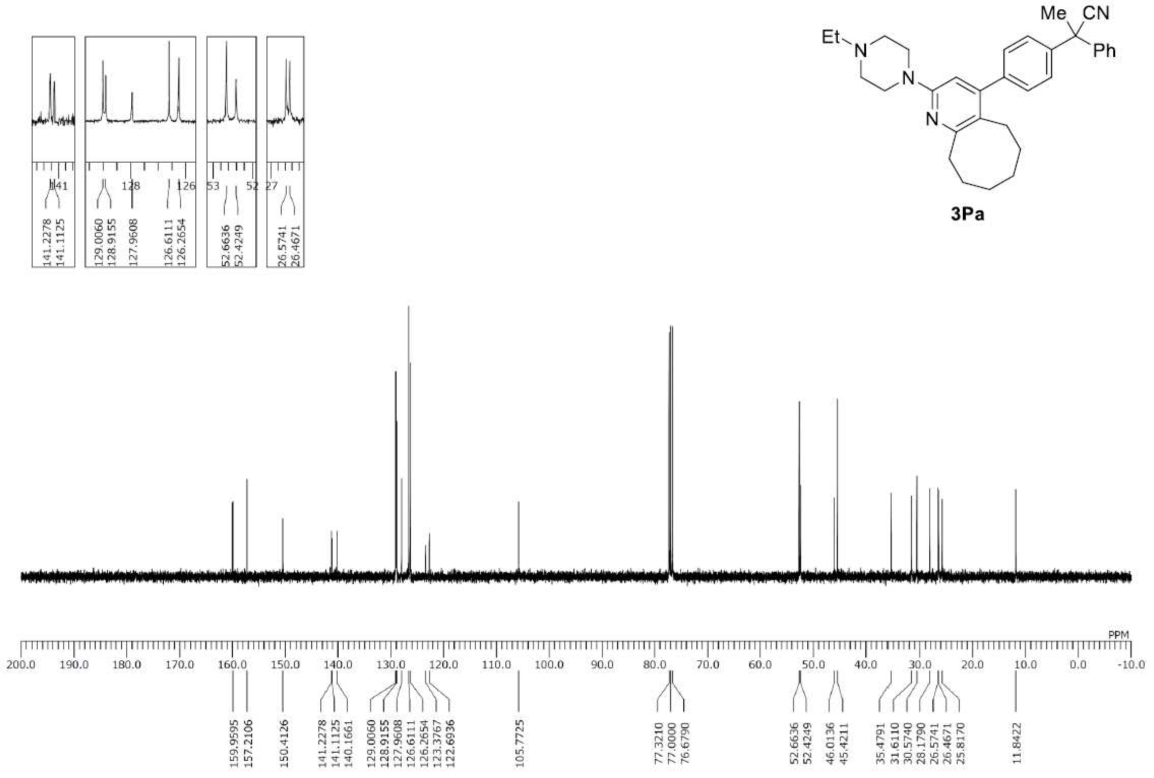

Supplement: Supplementary file 1 — ja4c09042_si_001.pdf [file ja4c09042_si_001.pdf]
